# Potential association between COVID-19 and neurological disorders: analysis of common genes and therapeutics

**DOI:** 10.3389/fneur.2024.1417183

**Published:** 2024-10-14

**Authors:** Wenzhi Chen, Shishi Jiang, Cheng Li, Shu Li, Junling Wang, Renshi Xu

**Affiliations:** ^1^Department of Neurology, Jiangxi Provincial People’s Hospital, The Clinical College of Nanchang Medical College, The First Affiliated Hospital of Nanchang Medical College, National Regional Center for Neurological Diseases, Xiangya Hospital of Central South University Jiangxi Hospital, Nanchang, China; ^2^Department of Neurology, Xiangya Hospital, Central South University, Changsha, China

**Keywords:** COVID-19, neurological disorders, common dysregulated genes, bioinformatics analysis, molecular mechanisms, therapeutic pathways

## Abstract

As the COVID-19 pandemic persists, the increasing evidences suggest that the patients with COVID-19 may face the risks of the neurological complications and sequelae. To address this issue, we conducted a comprehensive study aimed at exploring the relationship between COVID-19 and various neurological disorders, with a particular focus on the shared dysregulated genes and the potential therapeutic targets. We selected six neurological disorders for investigation, including Alzheimer’s disease, epilepsy, stroke, Parkinson’s disease, and the sleep disorders. Through the bioinformatics analysis of the association between these disorders and COVID-19, we aimed to uncover the common molecular mechanisms and the potential treatment pathways. In this study, we utilized the publicly available RNA-Seq and microarray datasets, and employed tools such as Limma and DESeq2 for the differential gene analysis. Through the Gene Ontology and Kyoto Encyclopedia of Genes and Genomes pathway enrichment analysis, we explored the common biological features and pathways. Additionally, we focused on analyzing the regulatory roles of miRNA and transcription factors on the shared differentially expressed genes, and predicted the potential drugs interacting with these genes. These analyses contribute to a better understanding of the relationship between COVID-19 and the neurological disorders, and provide a theoretical basis for the future treatment strategies. Through this research, we aim to offer the deeper insights to the scientific community and present the new perspectives for the clinical practice in addressing the challenges of the neurological complications and sequelae faced by the COVID-19 patients.

## 1 Introduction

The coronavirus disease 2019 (COVID-19) is an infectious disease primarily affecting the respiratory system caused by severe acute respiratory syndrome coronavirus 2 (SARS-CoV-2) (1). As of August 14, 2022, there have been 587 million reported cases globally, with 6.4 million deaths (1). The clinical manifestations of COVID-19 primarily involve respiratory symptoms such as fever, the dry cough and fatigue. Severe cases may lead to the respiratory distress, hypoxemia, the acute respiratory distress syndrome, and even death. Similar to SARS-CoV, SARS-CoV-2 primarily enters the host cells through angiotensin-converting enzyme 2 (ACE2) (2). Given the widespread distribution of ACE2 receptors throughout the body, SARS-CoV-2 can also affect multiple systems beyond the respiratory system, including the digestive, cardiovascular, urinary and nervous systems (3).

With the spread of the COVID-19 pandemic and increasing clinical cases, more and more cases of COVID-19 initially present with the neurological symptoms or have only the neurological symptoms during the mild infections. Among the patients with severe COVID-19, the proportion of those presenting with the neurological symptoms and signs is even higher (4). In a retrospective study by Kacem et al. (5), among 646 included cases out of 1,034 confirmed COVID-19 pneumonia patients, 466 cases (72.1%) had the neurological symptoms (14.5% preceding the respiratory symptoms, 48.1% accompanied by the respiratory symptoms, 27.8% following the respiratory symptoms), and 106 cases (22.7%) had the pure neurological symptoms. The neurological disorders associated with COVID-19 include headaches, anosmia, dysgeusia, delirium, the altered consciousness, the cognitive impairment, seizures, delirium, dizziness, coma, encephalopathy and the potential neurodegenerative diseases (6, 7).

A prospective study in New York City showed that among 4,491 hospitalized COVID-19 patients, 606 (13.5%) developed the new neurological diseases within 2 days of COVID-19 symptom onset, with the most common diagnoses being toxic/metabolic encephalopathy (6.8%), seizures (1.6%), stroke (1.9%) and the hypoxia/ischemic injury (1.4%) (8). A study by the NIH indicated that the novel coronavirus can damage the brain blood vessels without infecting the brain tissue (9). Furthermore, research has confirmed that after the infection with the novel coronavirus, the protein structures in the brain neurons undergo misfolding similar to diseases such as Alzheimer’s disease (AD) and Parkinson’s diseases (PD) (10).

The terrifying aspect of the novel coronavirus lies not only in its rapid spread but also in the various serious sequelae it may bring, known as “long COVID” or “post-acute sequelae of SARS-CoV-2 infection (PASC).” This condition is characterized by the persistence or appearance of new symptoms of COVID-19. Although there is no the formal definition of this condition, PASC is the typically defined as the COVID-19 symptoms persisting for more than 30 days (11–13). The neurological symptoms are a prominent feature of PASC, with the common symptoms including the cognitive impairment, the sleep disorders, the autonomic nervous system disorders, depression, the post-traumatic stress symptoms and the substance use disorders (14, 15).

“Long COVID” may affect any individual with a COVID-19 infection, and it may even develop in the asymptomatic or mildly infected individuals. Even the mildly symptomatic individuals may experience the cognitive impairment syndrome characterized by the impaired attention, the information processing speed, memory and the executive function, a condition often referred to as “COVID-fog” (16). A large study from the Netherlands found that among the adults diagnosed with COVID-19, 21.4% experienced at least one new or worsened symptom within 3 to 5 months after infection, compared to 8.7% in the uninfected individuals during the same period. This suggests that in the general population, one in eight COVID-19 patients (12.7%) may experience the corresponding long-term symptoms due to the COVID-19 infection (17). “Long COVID” can last for 3 months, 6 months, 9 months, or even longer, and this condition has been described as the next emerging public health disaster. However, the mechanisms underlying the neurological symptoms of “long COVID” are still poorly understood.

In conclusion, there is a certain association between COVID-19 and various neurological diseases. The reports of the neurological complications and sequelae during the COVID-19 pandemic suggest that the virus may have the direct or indirect effects on the nervous system. Based on the current various reports about the potential association between COVID-19 and the neurological disorders, it hypothesizes a potential molecular overlap such as a gene- or pathway- level between COVID-19 and the neurodegenerative diseases according to various pieces of evidences. The common occurrence of the cognitive impairment in the COVID-19 patients suggests that the virus might impact the nervous system. Additionally, the neurological complications observed in post-COVID-19, such as the acute encephalopathy, stroke and the long-term cognitive impairment, further support this hypothesis. While these phenomena do not directly prove a genetic or pathway-level overlap between COVID-19 and the neurodegenerative diseases, which suggests a potential connection between COVID-19 and the neurodegenerative diseases. Our research aims to explore this potential molecular overlap through the bioinformatics analysis.

The selection of diseases for our study is not arbitrary but is based on the known clinical associations between COVID-19 and various neurological diseases, such as AD and PD. These diseases share some common features such as neuroinflammation and the oxidative stress, which provide a reasonable basis for our investigation. By comparing the transcriptomic data of these diseases with COVID-19, we aim to uncover their common molecular mechanisms and the potential therapeutic pathways.

In this study, we selected six common COVID-19-related neurological diseases, including the hemorrhagic stroke (HS), the ischemic stroke (IS), PD, AD, epilepsy (EP) and sleep disorders (SD), for the bioinformatics analysis of their interaction with COVID-19. From the perspective of the gene interactions, we aim to elucidate the reasons for the occurrence of the neurological complications during the acute phase of the SARS-CoV-2 infection and the potential pathogenic mechanisms of the long COVID neurological symptoms.

The six neurological diseases we selected (including AD, epilepsy, stroke, PD, and the sleep disorders) may share some commonalities in pathogenesis and pathophysiology, such as the inflammatory responses and apoptosis. Therefore, by analyzing the common dysregulated genes of these diseases and combining the protein–protein interaction network analysis, the gene regulatory network analysis, and the protein-drug interaction network analysis, we can reveal the potential shared biological mechanisms between COVID-19 and the neurological diseases, thereby providing the new insights and strategies for the treatment of the related diseases.

## 2 Methods

We have combined the gene dysregulation across the different diseases and tissue types based on the hypothesis that COVID-19 may cause the neurological complications across multiple systems. Considering that COVID-19 can induce the pathophysiological changes in the nervous system, the immune system and other organ systems, we believe that an overall analysis of the gene dysregulation between the different diseases and tissue types can offer a more comprehensive perspective, revealing the potential common mechanisms. Furthermore, while the different diseases and tissue types may have the unique pathophysiological features, we believe that there are the shared pathophysiological foundations, especially in the immune regulation and the inflammatory response. These commonalities may lead to the co-occurrence of certain gene dysregulations across the different diseases and tissue types. By integrating the data from the different diseases and tissue types, we can identify these commonly dysregulated genes, thereby enhancing our understanding of the mechanisms underlying the SARS-CoV-2’s role in the neurological complications. Our study design aims to explore the commonly dysregulated genes across the different diseases and tissue types rather than simply merge them into a unified model. We will employ the systems biology approaches and the appropriate statistical analyses to elucidate the similarities and differences between the different diseases and tissue types, strive to provide a deeper understanding of the potential mechanisms underlying the SARS-CoV-2 neurological complications.

### 2.1 Data acquisition

We selected the datasets corresponding to six neurological diseases from the GEO database (18) for the subsequent analysis. GSE153873 represents the RNA-Seq datasets for AD, with the brain tissue samples from 12 patients and 10 healthy controls. GSE134697 represents the RNA-Seq datasets for epilepsy, with the brain tissue samples from 17 patients and 2 healthy controls. GSE163256 represents the RNA-Seq datasets for the hemorrhagic stroke, with the peripheral blood samples from 189 patients and 12 healthy controls. GSE16561 represents the microarray datasets for the ischemic stroke, with the peripheral blood samples from 39 patients and 24 healthy controls. GSE54536 represents the microarray datasets for PD, with the peripheral blood samples from 4 patients and 5 healthy controls. GSE37667 represents the microarray datasets for the sleep disorders, with the peripheral blood samples from 9 patients and 9 healthy controls. Additionally, we selected two datasets for COVID-19, wherein GSE183533 represents the RNA-Seq datasets from the COVID-19 lung tissue, with samples from 27 patients and 6 healthy controls. Another COVID-19 datasets, E-MTAB-8871, sourced from the AraayExpress database (19), was used to analyze the immune responses in whole blood cells of the COVID-19 patients and the healthy individuals, with the samples from 22 patients and 10 healthy controls. All datasets applied in this study are from the *Homo sapiens*. The characteristic information for all transcriptome datasets used in this study has been summarized in Table 1.

**Table 1:** Summary table of transcriptome data characteristics.

Disease	Accession	Database	Samples	Source	Experiment type	Years	Organism	Platforms
COVID-19 (immune)	E-MTAB-8871	ArrayExpress	33	Blood	Array assay	2020	*Homo sapiens*	NanoString
COVID-19 (lung)	GSE183533	GEO	41	Lung tissue	RNA-Seq	2022	*Homo sapiens*	GPL24676
Epilepsy	GSE134697	GEO	36	Brain	RNA-Seq	2019	*Homo sapiens*	GPL16791
Intracerebral hemorrhage	GSE163256	GEO	399	Peripheral blood	RNA-Seq	2021	*Homo sapiens*	GPL18573
Ischemic stroke	GSE16561	GEO	63	Whole blood RNA	Array assay	2010	*Homo sapiens*	GPL6883
Parkinson’s disease	GSE54536	GEO	10	Peripheral blood	Array assay	2014	*Homo sapiens*	GPL10558
Sleep disorder	GSE37667	GEO	27	Blood	Array assay	2012	*Homo sapiens*	GPL570
Alzheimer’s disease	GSE153873	GEO	30	Frozen postmortem brain tissue	RNA-Seq	2020	*Homo sapiens*	GPL18573

### 2.2 Identification of differentially expressed genes

R (version 4.1.2) was used for the data organization and statistics. The “Limma” package in R was utilized for the differential analysis of the microarray datasets (20), while the “DESeq2” package was used for the differential analysis of the RNA-Seq data (21). To obtain the significant differentially expressed genes (DEGs), the cutoff criteria were set at |log2(Fold Change)| >1 and the adjusted *p*-value <0.05. The “ggplot2” package was used for the data visualization (22).

### 2.3 Gene Ontology and pathway enrichment analysis

To determine the biological significance of the DEGs obtained from the differential analysis, we conducted the Gene Ontology (GO) and the Kyoto Encyclopedia of Genes and Genomes (KEGG) analyses. The “clusterProfiler” package (23) in R was employed for analysis, with the cutoff criteria requiring adj. *p* < 0.05 and *q*-value <0.2. The “ggplot2” package was used for the data visualization (22).

### 2.4 Protein–protein interaction analysis

Following the differential analysis, to understand the shared pathogenic mechanisms of these DEGs between COVID-19 and the neurological diseases, we constructed the protein–protein interaction (PPI) networks of these DEGs using the STRING database (24). The “igraph” package (version 1.2.6) and the “ggraph” package (version 2.0.5) were used for the visualization of the PPI results (25, 26).

### 2.5 Gene regulatory network analysis

Based on the analyzed DEGs, miRNAs were predicted using the miRTarBase database (27), and the transcription factors (TFs) were predicted using the JASPAR database (28). Finally, using the NetworkAnalyst, the predicted TFs, miRNAs and DEGs were separately used to construct the gene regulatory network (GRN) (29). The GRN analysis reveals the molecular mechanisms of the post-transcriptional regulation of DEGs and is an important method for the mechanistic research.

### 2.6 Protein-drug interaction analysis

In this study, to identify the effective treatments for COVID-19, the DrugBank database was used to predict the drugs interacting with DEGs (30). Additionally, NetworkAnalyst was used to construct the protein-drug interaction network (29), aid in the discovery of more valuable drugs among these predicted drugs.

### 2.7 Age, vaccinated and the rates of COVID-19 infection and neurological disorders incidence analysis

We conducted a retrospective analysis to retrieve and summarize the past literature (31–35) on the relationship between the factors such as age and vaccination, and the incidence rates of COVID-19 and certain neurological diseases.

## 3 Results

### 3.1 Identification of differentially expressed genes reveals links between COVID-19 and neurological complications after SARS-CoV-2 infection

**Figure 1:**
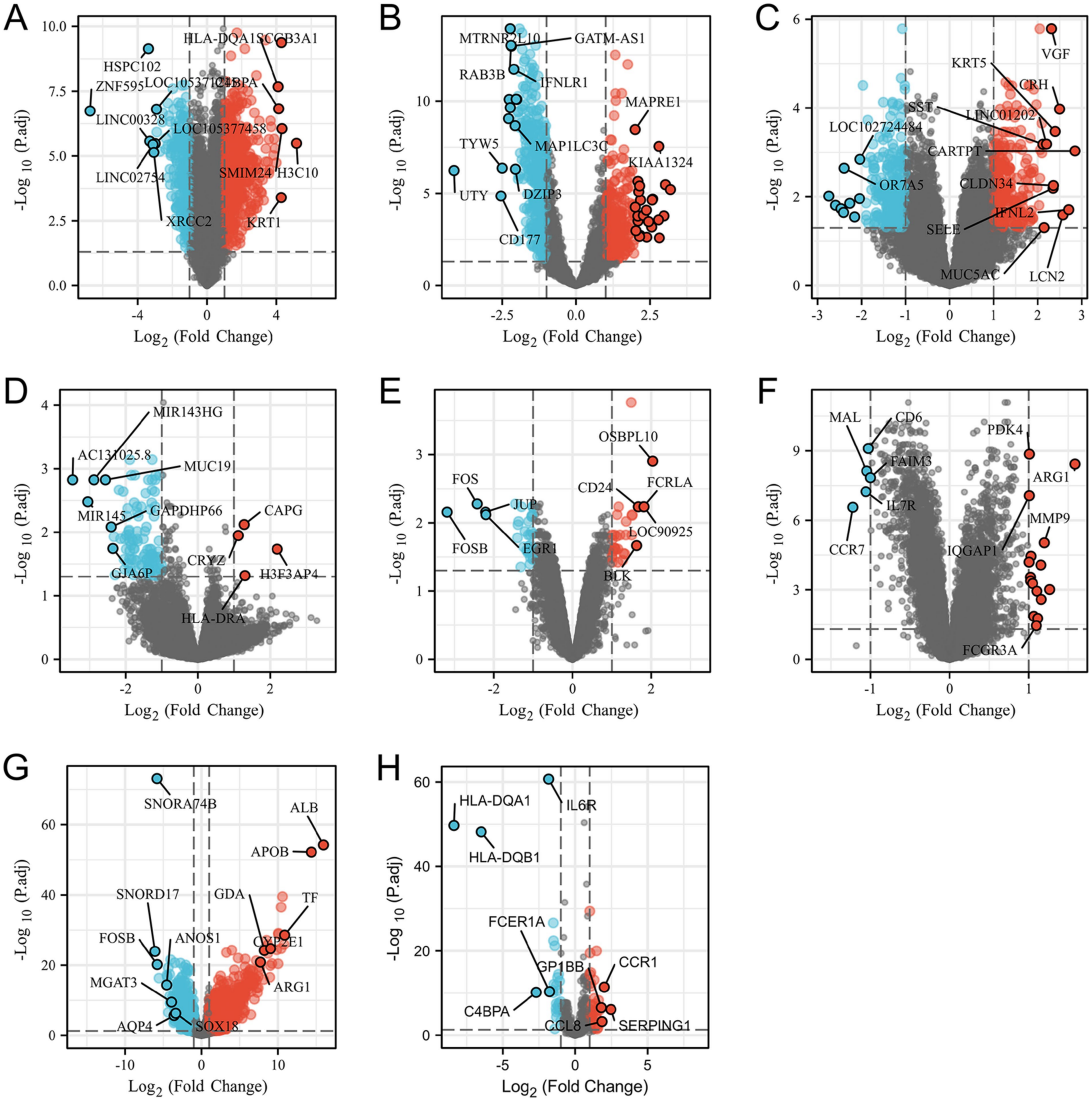
Volcano plots **(A–H)** show the differential genes for SD, HS, AD, ED, PD and IS diseases, and both SARS-CoV-2 and the SARS-CoV-2 immune datasets, respectively. The filtering criteria of adjusted *p*-value <0.05 and |logFC| >1.

**Figure 2:**
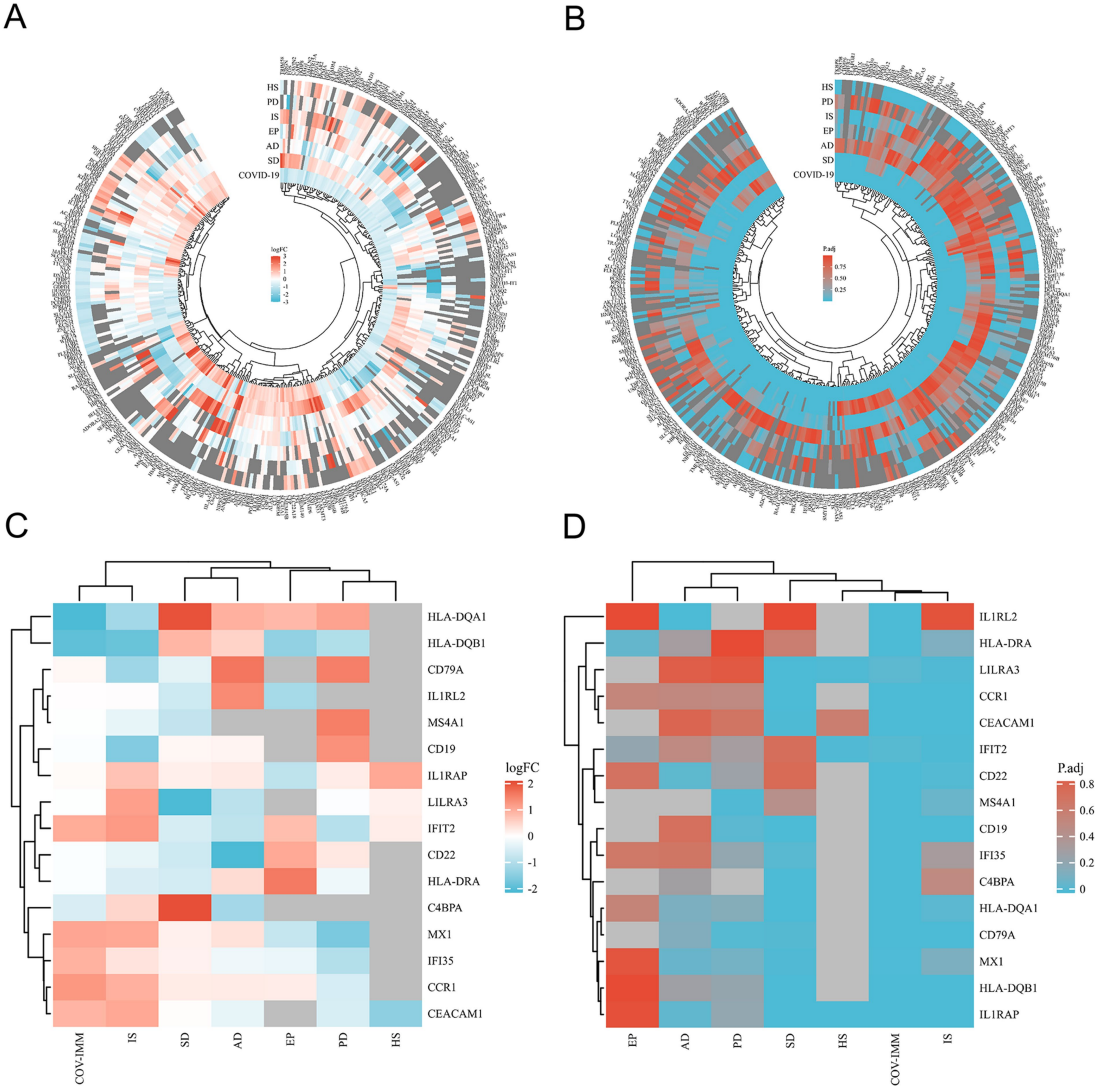
Heatmap shows the differences between SARS-CoV-2 and SARS-CoV-2 immune datasets and the common differential genes of the six neurological diseases in the terms of logFC **(A,C)** and corrected (adjusted) *p*-values **(B,D)** respectively.

**Figure 3:**
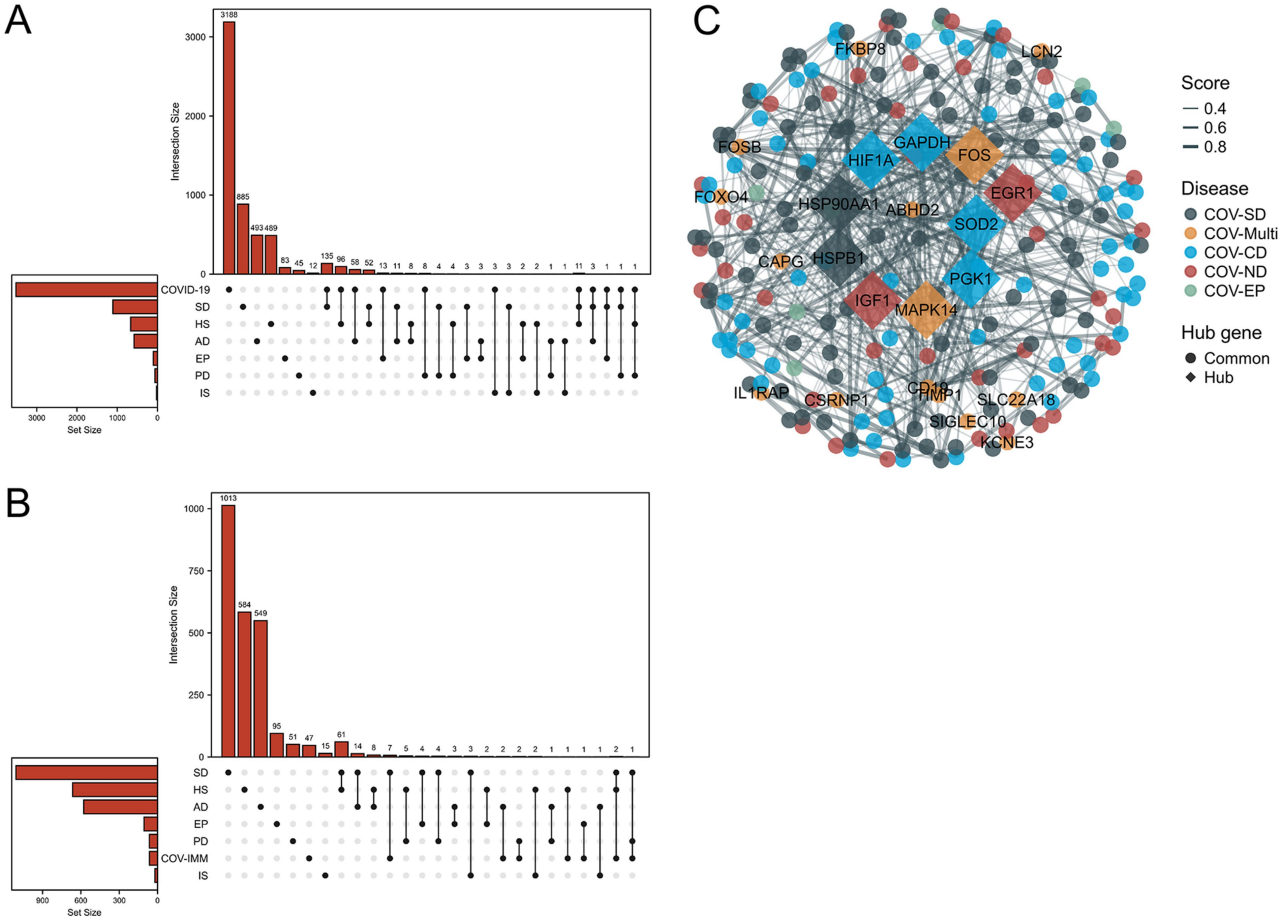
Upset plots depict the distribution of common differential genes between the six neurological diseases and SARS-CoV-2. **(A,B)** Shows the SARS-CoV-2 datasets and the SARS-CoV-2 immune response datasets. **(C)** Shows the PPI of common differential genes between the six neurological diseases and SARS-CoV-2 considering the immune sample, where COV-multi refers to the common differential genes between SARS-CoV-2 and 2 diseases, COVID-neurodegenerative diseases (COV-ND) refers to the common differential genes between SARS-CoV-2 and one of the neurodegenerative diseases (AD and PD), and COVID-CVD (COV-CD) refers to the common differential genes between SARS-CoV-2 and one of the cerebrovascular diseases (HS and IS). COV-SD and COV-EP refers the common differential genes between SARS-CoV-2 and epilepsy (EP), and sleep disorders (SD) respectively. The top 10 of hub genes were excavated through cytoHubba for significant differential genes shared among multiple diseases.

Through the differential analysis, we obtained DEGs from six neurological disease datasets and two COVID-19 datasets. The criteria for selecting the significant DEGs were an adjusted *p*-value (adj. *p*-value) of less than 0.05 and an absolute value of log2 fold change (logFC) greater than 1. As shown in the volcano plot in Figure 1, the significant DEGs are marked with the red or blue circles, where the red represents the upregulated genes and the blue represents the downregulated genes. The number of the significant DEGs varies, with the COVID-19 lung tissue datasets having 3,518 DEGs, the SD blood datasets having 1,109 DEGs, the HS neutrophil datasets having 665 DEGs, the AD brain tissue datasets having 578 DEGs, the EP brain tissue datasets having 105 DEGs, the PD peripheral blood datasets having 64 DEGs, the COVID-19 blood immune response datasets having 63 DEGs and the IS whole blood datasets having 21 DEGs (specific DEGs are referenced in Supplementary Table S1). The Figures 2A,B in heatmap illustrate the association between COVID-19 and other diseases based on logFC values and adj. *p*, showing that COVID-19 shares more DEGs with SD and AD. The further comparison revealed the overlapping DEGs among the different diseases, Figure 3A shows the distribution of the common DEGs between COVID-19 and six common neurological diseases. Notably, COVID-19 shares more genes with SD, HS and AD, with 135, 96 and 58 common genes respectively, while the least common genes are found with IS, with only 3 shared genes.

### 3.2 Immune association between COVID-19 and common neurological diseases

We performed the differential analysis of the COVID-19 blood immune response datasets with six neurological diseases and identified the overlapping DEGs (Supplementary Table S2). The Figures 2C,D in heatmap depict the association between the COVID-19 immune response datasets and the other six diseases based on logFC values and adj. *p*. The heatmap shows that COVID-19 shares fewer immune-related DEGs with the considered diseases. All immune-related DEGs among these diseases include 16 genes: HLA-DQA1, HLA-DQB1, CD19, CCR1, IL1RAP, C4BPA, CEACAM1, IFI35, MX1, LILRA3, IFIT2, CD22, IL1RL2, HLA-DRA, MS4A1, and CD79A. Figure 3B displays the distribution of the common immune-related DEGs between COVID-19 and the six diseases.

To further demonstrate the associations between diseases, we merged the differential genes from the COVID-19 lung tissue and the immune response datasets (refer to Supplementary Table S3) and analyzed the overlap of DEGs among three diseases. COVID-SD (COVID-19 SD) and HS shared 12 common genes: ABHD2, CSRNP1, FKBP8, IL1RAP, KCNE3, LILRA3, MAPK14, SIGLEC10, SLC22A18, TIMP1, TMEM45B and TNFAIP6; COVID-19, SD and AD shared 3 genes: FOXO4, LCN2 and SMIM5; COVID-19, SD and EP shared 1 gene: CAPG; COVID-19, SD and PD shared 2 genes: CD19 and FOS; COVID-19, HS and PD shared 1 gene: FOSB (refer to Supplementary Table S4).

### 3.3 GO and KEGG analysis reveals potential biological processes and shared signaling pathways between COVID-19 and neurological diseases

**Figure 4:**
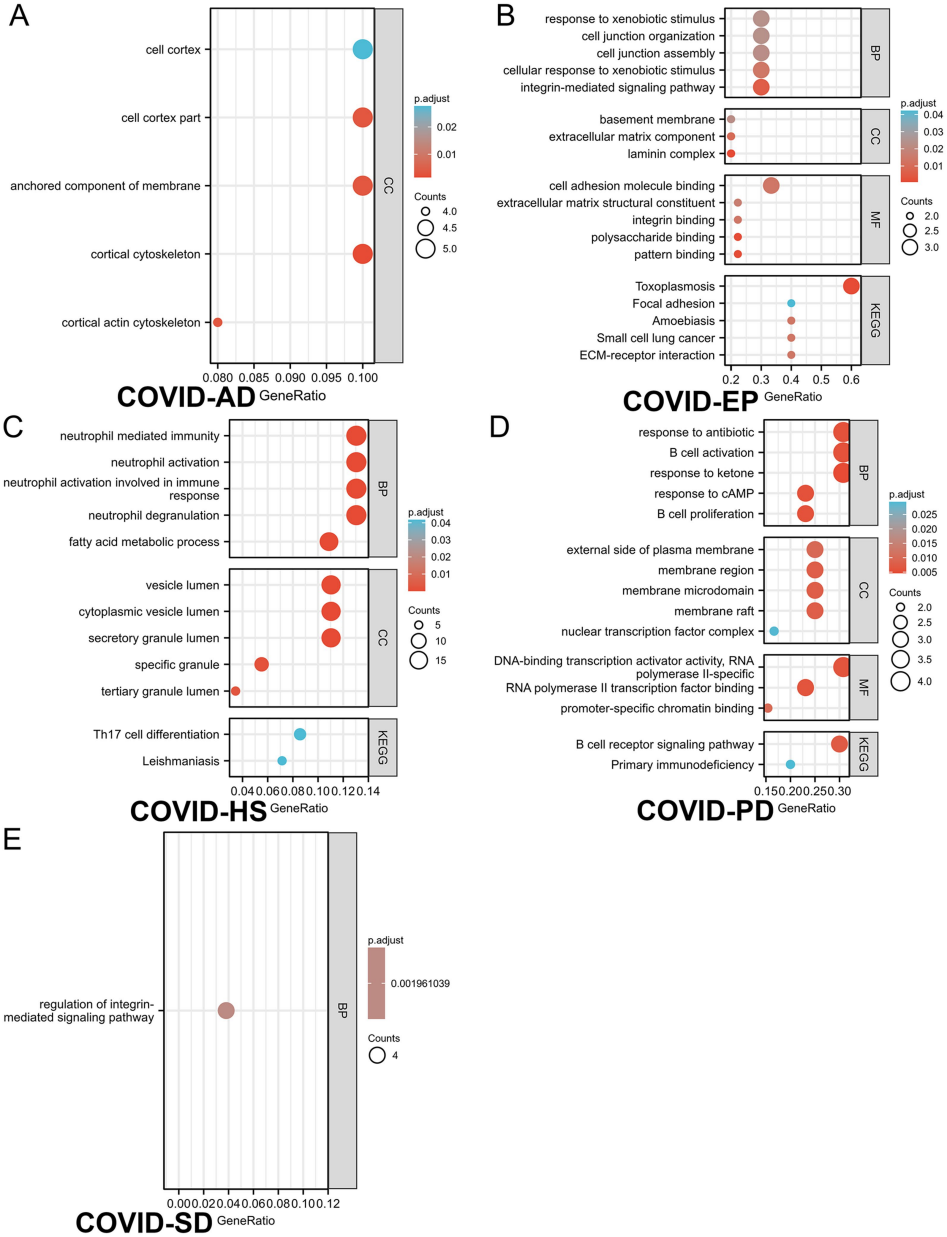
The GO/KEGG analysis of differential genes between 5 neurological diseases and SARS-CoV-2 considering immune samples. **(A–E)** refer to **(A)**: COVID-AD, **(B)**: COVID-EP, **(C)**: COVID-SD, **(D)**: COVID-PD, **(E)**: COVID-HS, respectively. Gene ratio refers to the ratio of the number of genes associated with a specific function or pathway in the enrichment results to the total number of genes provided in the gene list. This ratio can help determine the abundance or significance of a specific function or pathway within the provided gene list. A higher gene ratio may indicate a greater number of genes associated with the specific function or pathway within the provided gene list, which may be relevant to the biological characteristics under study or the functions of interest.

**Figure 5:**
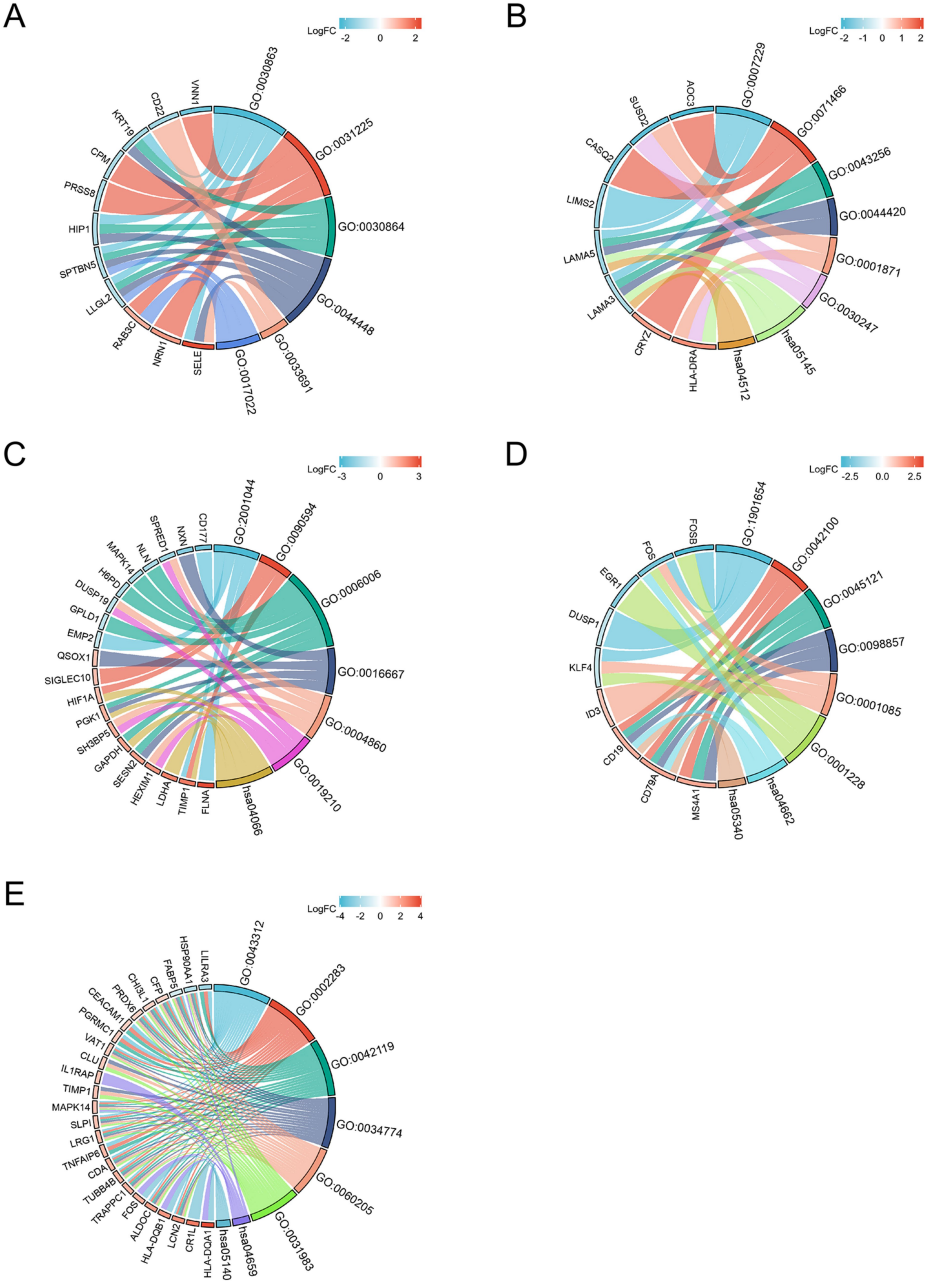
The chord diagram illustrates the relationship between differential genes and the significant enrichment pathway analysis results. **(A)** is COVID-AD, **(B)** is COVID-EP, **(C)** is COVID-HS, **(D)** is COVID-PD and **(E)** is COVID-SD (the width of the connecting line indicates the degree of relationship between the gene and CC, MF, BP or pathway).

Based on the merged differential genes from both COVID-19 lung tissue and immune response datasets, we identified the DEGs shared between COVID-19 and the other six neurological diseases. GO and KEGG analysis was performed on these DEGs, with filtering the criteria of adj. *p* < 0.05 and *q*-value <0.2. The top 5 entries from each GO aspect (molecular function, biological process and cellular component) and pathway analysis results were visualized. Figure 4 presents the enrichment results, where A-E represents COVID-AD, COVID-EP, COVID-HS, COVID-PD and COVID-SD, respectively. From the Figure 4, it is evident that COVID-19 shares more DEGs with EP and PD, with highly enriched pathways associated with the B cell receptors and the immune regulation for COVID-19 and PD, and multiple pathways including the Toxoplasmosis pathway for COVID-19 and EP. The interaction between COVID-19 and SD is also notable, with the enriched pathways including Th17 cell differentiation and Leishmaniasis. COVID-AD only showed results related to the cellular components, lacking the information on the molecular function, pathways and the biological processes. COVID-HS exhibited the fewest enriched results, with only 1 pathway related to the regulation of integrin-mediated signaling pathway. Additionally, we utilized the logFC values of DEGs to construct the chord diagrams (Figure 5), illustrating the interaction between genes and both GO and KEGG analysis results. Figure 5 depicts the interaction between shared DEGs among COVID-19 and the different neurological diseases with their enrichment results.

### 3.4 Protein–protein interaction analysis identifies networks for neurological complications and long COVID sequelae

We constructed a PPI network composed of the DEGs shared between COVID-19 and six neurological diseases, as shown in Figure 3C. We grouped both AD and PD as the neurodegenerative diseases, and both HS and IS as the cerebrovascular diseases (CVD) for the clearer results. The term COV-Multi represents the differential genes shared between COVID-19 and the two neurological diseases, presented as the circular nodes labeled with the gene names in Figure 3C. We utilized STRING (24) to construct the basic PPI network and filtered out the top 10 of hub genes using Cytoscape software and Cytohub plugin (36, 37), represented as the diamond nodes labeled with the gene names in Figure 3C. Through the PPI network, we observed a predominance of the blue nodes, followed by the dark green and the dark red nodes, while the cyan and orange nodes were less abundant. This suggests a broader connection between the cerebrovascular diseases and COVID-19.

### 3.5 Gene regulatory network analysis determines interactions between DEGs-miRNA and TF-DEGs

**Figure 6:**
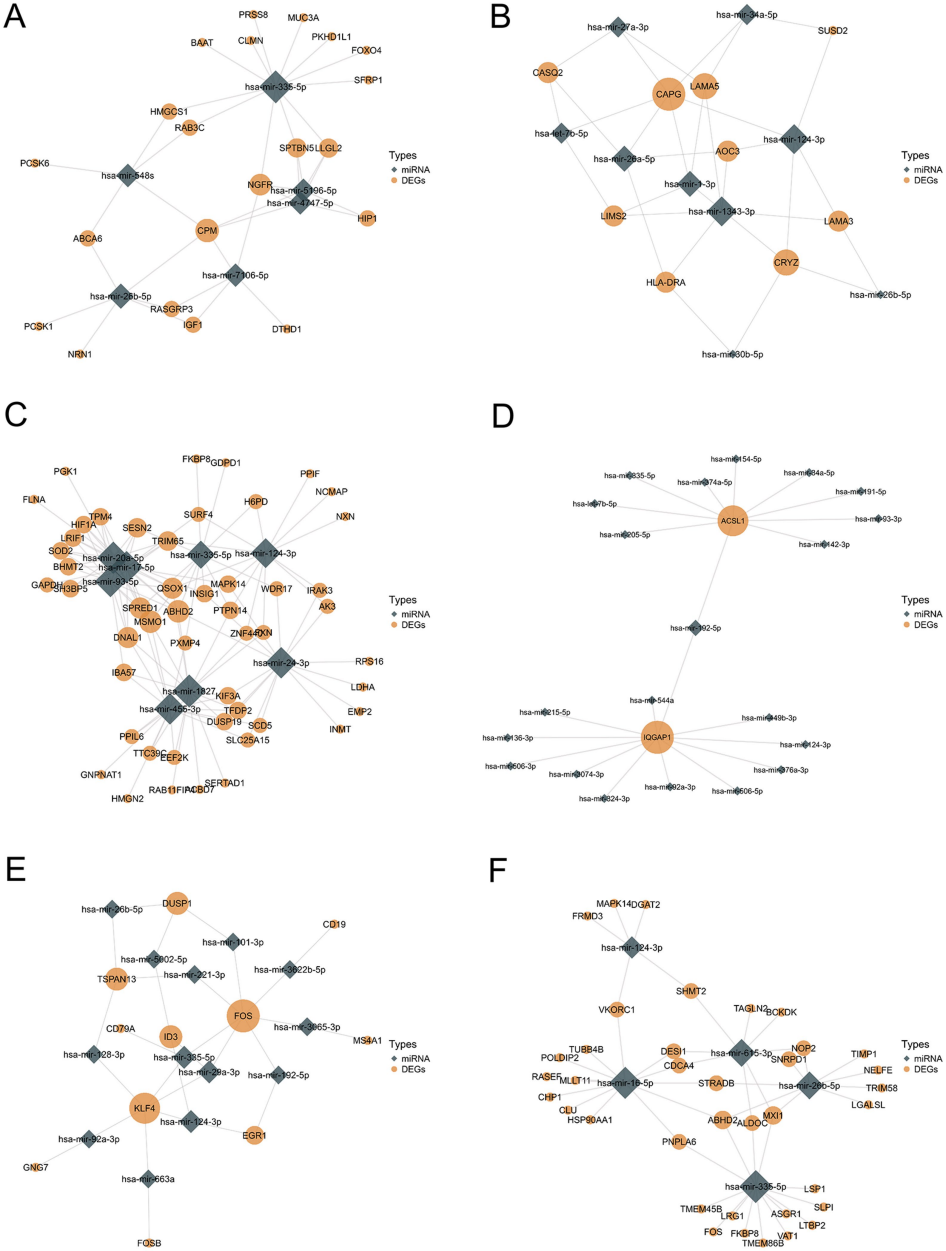
Differentially expressed genes—miRNA interaction network analysis **(A–F)**.

Using the miRTarBase database (27), we predicted the potential miRNAs shared by COVID-19 and six neurological diseases and analyzed their interactions, where the COVID-19 DEGs were derived from the union of lung tissue and immune response datasets. Figure 6 illustrates the DEGs-miRNA interaction network, where the size of the diamond and circular nodes represents the node’s degree, indicating its importance within the network. The nodes CPM, hsa-mir-335-5p (Figure 6A), CAPG, hsa-mir-1343-3p (Figure 6B), MSMO1, hsa-mir-20a-5p (Figure 6C), IQGAP, hsa-mir-192-5p (Figure 6D), FOS, hsa-mir-335-5p (Figure 6E), and ABHD2, hsa-mir-26b-5P (Figure 6F) appear to be more valuable.

**Figure 7:**
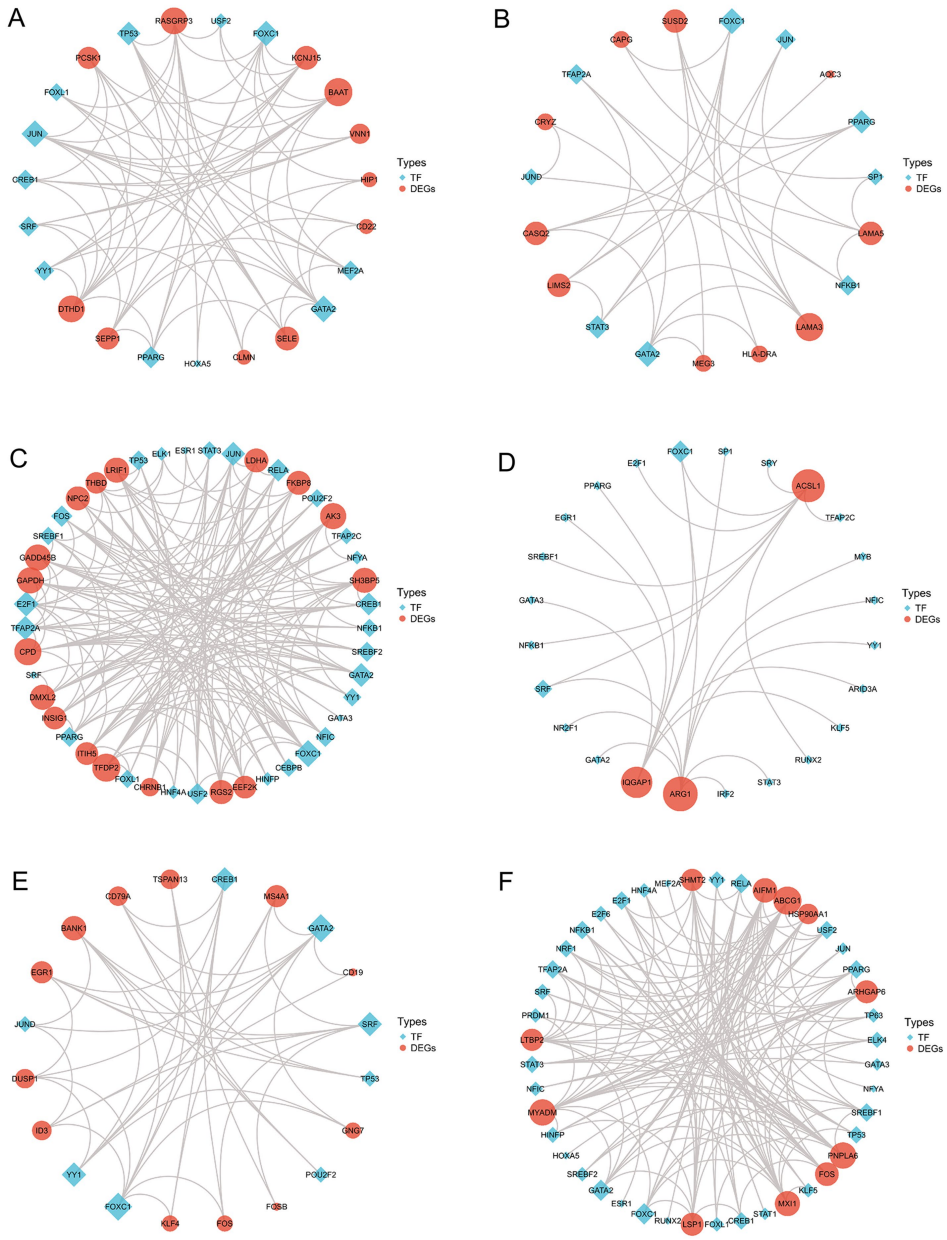
Transcription factors-differentially expressed genes interaction network analysis **(A–F)**.

Furthermore, using the JASPAR database (28), we constructed an interaction network between TFs and DEGs. Figure 7 displays the TFs-DEGs interaction network results, where the red represents DEGs, the blue represents TFs, and the size of nodes represents the node’s degree. From Figures 7C,F, it can be observed that TFs are more active in HS and SD. Some TFs such as FOXC1, FOXL1, GATA2 and PPARG are present in multiple disease interaction networks, indicating their widespread involvement in the regulatory transcription processes of these diseases.

### 3.6 Protein-drug interaction analysis provides insights for treating neurological complications and long COVID sequelae

**Figure 8:**
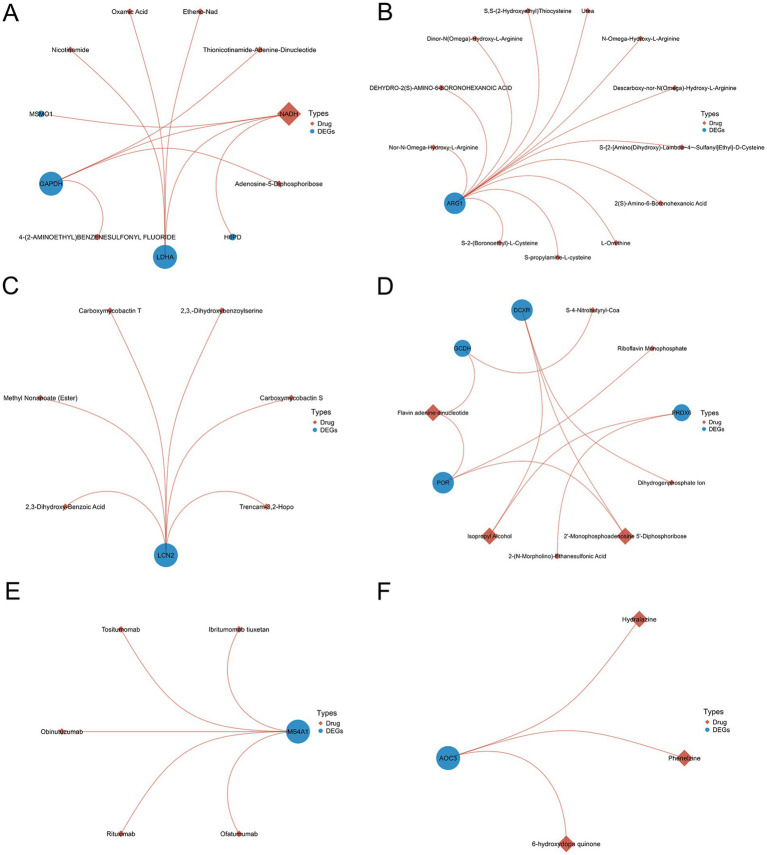
The analysis of protein-drug interactions using differentially expressed genes shared by SARS-CoV-2 and six neurological diseases **(A–F)**.

Utilizing the DrugBank database (30), we conducted the protein-drug interaction analysis of the DEGs shared between COVID-19 and six neurological diseases. The results are presented in Figure 8, where the blue circles represent DEGs and the brown diamonds represent the predicted interacting proteins. The node size reflects the connectivity with other nodes. Through the protein-drug interaction analysis, we identified some potential targets for intervention shown in Figure 8, providing the treatment avenues for the neurological complications caused by COVID-19 and the long COVID sequelae.

### 3.7 Age, vaccinated and the rates of COVID-19 infection and neurological disorders incidence analysis

Indeed, the infection rates of COVID-19 and the incidence rates of the neurological diseases vary significantly across the age groups, which could impact the interpretation of our study results. We attempted to use the meta-analysis to summarize the relationship between age and the incidence of the neurological diseases in post-COVID-19 infection. Unfortunately, due to significant heterogeneity in study design and methods, as well as the variable quality among the studies we retrieved, a robust meta-analysis was not feasible. Therefore, we selected several high-quality studies and constructed to the detail of the relationship between the COVID-19 infection rates and the incidence of certain neurological diseases across the different age groups (Table 2).

**Table 2:** Analyzing of age and vaccination effects on COVID-19 and neurological incidences.

Authors and year	Risk factors	Neurological disorders	Conclusions
Louapre 2020	Age	Multiple sclerosis	Age is an independent risk factor for severe COVID-19
Mao 2020	Age	Acute cerebrovascular disease, consciousness disorders, skeletal muscle injury, epilepsy	Compared to non-severe cases, patients with severe infection are older, and those with more severe infections exhibit neurological manifestations
Ferini-Strambi 2021	Age	Parkinson’s disease, multiple sclerosis	Age, rather than COVID-19 neuropathology, may play a decisive role in neurodegenerative diseases like Parkinson’s disease, while COVID-19 neuropathology, rather than age, may play a decisive role in neuroimmunological diseases like multiple sclerosis
Jeantin 2024	Age, gender, weight	Multiple sclerosis	Age, gender, and obesity are risk factors for the severity of COVID-19
Chen 2024	Vaccine	Cerebrovascular disease	Vaccination can reduce the risk of cerebrovascular disease in COVID-19 survivors

Despite recognizing the importance of the multi-factorial analysis including the vaccination status, the limitations of the data prevented us from conducting such an analysis in our current study. Our research team has been actively collaborating with the data providers, hoping to obtain more comprehensive data in the future studies that would allow us to delve into the specific effects of the vaccination status on the neurological complications after the COVID-19 infection.

We also conducted some current literatures review and found that most of the literature discusses the impact of vaccines themselves on the nervous system, rather than exploring how vaccination status correlates with the incidence of the neurological complications after the COVID-19 infection. However, a very few studies did mention such correlations, for example, Chen et al. (31) found that the prior vaccination against COVID-19 could reduce the risk of the cerebrovascular diseases in the COVID-19 survivors (this conclusion is summarized in the newly added Table 2), which is consistent with our study’s findings of the common activated genes between the COVID-19 infection and the cerebrovascular diseases. Yet, such studies are indeed scarce, and it was difficult to expand such a vast number of subgroup analyses with the limited scope of this manuscript. In the future, we plan to use more comprehensive data for the stratified analysis, considering not only the vaccination status but also the clinical symptoms, age, gender and lifestyle factors, to more accurately assess the impact of these variables on the incidence of the neurological diseases. This will help us gain a more complete understanding of the long-term effects of COVID-19 and provide a scientific basis for the prevention and treatment strategies.

## 4 Discussion

The patients with COVID-19 often report the persistent neurological symptoms. To understand the underlying mechanisms, we conducted a bioinformatics analysis comparing COVID-19 with six representative neurological diseases prone to the neurological complications after the SARS-CoV-2 infection and the “long COVID” neurological diseases (HS, IS, PD, AD, EP and SD). We included an additional COVID-19 immune response datasets to comprehensively assess the changes in the immune microenvironment. The differential analysis revealed the common DEGs between COVID-19 (including immune samples) and the six neurological diseases (SD, HS, AD, EP, PD and IS), with counts of 156, 110, 62, 14, 13 and 3, respectively (details in Supplementary Table S5). The lowest number of the common DEGs was observed between COVID-19 and IS, including ARG1, ACSL1 and IQGAP1. While ARG1 and ACSL1 were upregulated in both diseases, IQGAP1 was downregulated in COVID-19 but upregulated in IS. IQGAP1 plays a crucial role in the vascular homeostasis (38, 39), being upregulated in IS to repair and maintain the vascular function and angiogenesis in the post-ischemic events. In contrast, COVID-19 may deactivate the IQGAP1-mediated vascular maintenance, increasing the risk of stroke. The ARG1 upregulation in COVID-19 (40) and IS (41, 42) is associated with the attenuated inflammation, suggesting its role in mitigating inflammation in both diseases. The role of ACSL1 in COVID-19 and IS remains unclear but may relate to ferroptosis (43).

We further analyzed the common DEGs between COVID-19 and two neurological diseases, revealing 12, 3, 1, 2 and 1 common genes between COVID-19 and SD/HS, SD/AD, SD/EP, SD/PD and HS/PD, respectively (details in Supplementary Table S4). Using Networkanalyst and Cytoscape (29, 37), we conducted the protein network interaction analysis of the shared DEGs in three diseases, identifying the top 10 of hub genes, with MAPK14, FOXO4 and FOS at the core (details in Figure 3C). MAPK14, a member of the MAP kinase family (44), is involved in various neurological diseases, particularly affecting autophagy (45–48). Many viral infections, including SARS-CoV-2, activate the p38 mitogen-activated protein kinase (MAPK) signaling pathway (49). Studies have reported that the sustained immune activation of the AP-1/p38MAPK pathway is a specific feature of the COVID-19 pneumonia (50). The MAPK14 activation disrupts the autophagosome-lysosome fusion, impairing autophagy (47), with implications for the neurodegenerative diseases like AD and PD (45, 46, 48). The MAPK14-mediated inflammation, primarily through the autophagy inhibition (51–53), may compromise the vascular repair (54, 55). The mechanism underlying the MAPK activation in the SARS-CoV-2 infection remains unclear, but considering its neurodestructive role, the MAPK14 inhibitors could be the potential targets for the long COVID treatment. FOS, a proto-oncogene, is widely expressed and implicated in AD, the mechanical and ischemic brain injuries, epilepsy and depression (56). Although the pathogenic mechanism of FOS in COVID-19 is unknown, it has been proposed as a therapeutic target (57). The FOS induction is a reliable marker of the neural activity, suggesting its potential role as a sensitive indicator of the neural damage.

The GO and KEGG analysis identified the shared cellular component features between COVID-19 and AD, notably enrichment in the cell cortex. Interestingly, the recent studies suggest that SARS-CoV-2 induces the tunneling nanotubes (TNTs), facilitating the intercellular spread (58). For the neurons with fewer ACE2 receptors, this transmission mode may weaken the viral dissemination, potentially offering the novel intervention avenues to alleviate the neurological symptoms. Additionally, the EP and HS enrichment in the integrin-mediated signaling pathway suggests a role in the cell adhesion and signaling, critical for the diverse biological functions (59). Integrin, closely linked to ACE2 (60), may underlie some neurological complications (61). The enrichment of the immune-related processes and pathways in COVID-19, SD and PD, such as the neutrophil-mediated immunity and the Th17 cell differentiation, aligns with the previous findings (62), suggesting the immune-mediated neuronal damage in the COVID-19 complications. The chronic inflammation due to the sustained immune activation may contribute to anxiety and the sleep disorders in the long-COVID patients.

To explore the pathogenesis of the COVID-19-associated neurological diseases, we predicted miRNAs and TFs involved in the SARS-CoV-2-induced neurological diseases and sequelae. In the gene-miRNA regulatory networks, the core genes including FOS, IQGAP1, ABHD2 and MAPK-14 interact with the key miRNAs such as hsa-mir-335-5p and hsa-mir-124-3p. Although no the direct evidence links ABHD2 to COVID-19 and the neurological diseases, its role in regulating the male fertility is intriguing (63–65), as COVID-19 has been associated with the male infertility (66, 67). The TFs-gene regulatory networks identified the core TFs including PPARG, FOXC1 and GATA2, known for their roles in the inflammation regulation in the cerebrovascular diseases (68, 69). Furthermore, FOXC1 is associated with the energy metabolism in AD (70). The early studies have found that SRF and GATA2 are involved in regulating the formation of the substantia nigra protein in PD (71).

In addition to the potential targets like the MAPK-14 inhibitors and FOS, we conducted the protein-drug interaction analysis of the shared DEGs between COVID-19 and the neurological diseases, identifying the candidate COVID-19 therapies. Phenelzine, an antidepressant, emerged as a candidate COVID-19 therapy. However, its efficacy remains uncertain, as evidenced by conflicting *in vitro* results (72). NADH and FADH emerged as the candidate treatments (73, 74), with studies suggesting their roles in the COVID-19 recovery and susceptibility reduction (75–77).

In fact, COVID-19 has gradually faded from the public’s view, but the lingering sequelae still trouble some affected individuals. In the silico studies related to the neurological sequelae caused by COVID-19 are gradually being reported.

Verma et al. (78) investigated the post-acute sequelae of COVID-19 (PASC) and highlighted the persistent neurological symptoms such as the olfactory and autonomic dysfunction. Their study showed that the SARS-CoV-2-infected mice displayed the chronic neurological dysfunction similar to that seen in the neurodegenerative diseases, despite the virus not infecting the brain directly. The study emphasized the role of the chronic inflammation in exacerbating the neuronal vulnerability, drawing parallels with the human neurodegenerative conditions. Lee et al. (79) explored the transcriptomic changes in the human brain due to severe COVID-19. Their findings revealed hundreds of the differentially expressed long noncoding RNAs (lncRNAs) associated with the decreased cognitive performance and the inflammatory cytokine response. This study provided the evidence that the severe COVID-19 induces the widespread transcriptomic changes in the brain, potentially regulating the neurocognitive alterations akin to those seen in the neurodegenerative diseases. Griggs et al. (80) examined the neuropathophysiological consequences of the SARS-CoV-2 infection by comparing the transcriptional and cellular signatures in the specific brain regions (Brodmann area 9 and hippocampal formation) of COVID-19, AD and the SARS-CoV-2-infected AD patients. Their study demonstrated the similar neuroinflammation and the blood-brain barrier integrity alterations across these conditions, suggesting the shared pathological mechanisms between COVID-19 and the neurodegenerative diseases. Hu et al. (81) studied the persistent cognitive symptoms (“brain fog”) following COVID-19 in a large prospective cohort. Their single-cell gene expression analysis in the cerebrospinal fluid indicated the monocyte recruitment, the chemokine signaling, the cellular stress and the suppressed interferon response, especially in the myeloid cells. These findings underscored the inflammatory pathways involved in the prolonged cognitive dysfunction in post-COVID-19, echoing the mechanisms seen in the neurodegenerative diseases. Krishna et al. (82) focused on the impact of age and sex on the neuroinflammatory response to the SARS-CoV-2 infection using a mouse model. Their research demonstrated the significant changes in the brain gene expression profiles related to the innate immunity, the defense response to virus, the cerebrovascular and neuronal functions. Despite the absence of the detectable viral RNA in the brain, the study showed that SARS-CoV-2 triggered a neuroinflammatory response, highlighting the intersection of the immune and neurological pathways influenced by age and sex.

These studies collectively illustrate the intersection of COVID-19 and the neurodegenerative diseases through the shared molecular and inflammatory pathways. Our study builds upon this foundation by broadening the scope to include the multiple neurological disorders, aiming to uncover the common deregulated genes and the potential therapeutic targets. This integrative approach not only aligns with the existing research but also extends our understanding of the broader impact of COVID-19 on the neurological health. Based on this research, the purpose of our work and its implications aim to provide the deeper insights into the potential association between COVID-19 and the neurological disorders to the scientific community by analyzing the alterations of common genes between COVID-19 and the neurological disorders, and present new perspectives for the clinical therapeutics in addressing the challenges of the neurological complications and sequelae faced by the COVID-19 patients.

## 5 Summary

Our study covers a variety of neurological disorders, which exhibit the significant heterogeneity in the clinical presentation. Therefore, despite our efforts to integrate the different datasets and the analytical methods, the overlap results between diseases are limited by the diversity of samples. This may result in relatively fewer overlapping results in some cases. The different diseases may lead to changes in the gene expression across the different tissue types. Our research includes the data analysis from various tissue types such as the brain tissue, the peripheral blood and the lung tissue. Since the gene expression is the tissue-specific, we may observe fewer overlapping results between certain diseases, which does not necessarily imply lower biological relevance or significance. On the contrary, even a small number of overlapping results may reveal the key biological processes and pathways shared between diseases. Therefore, we emphasize the importance of the in-depth analysis and biological interpretation of overlapping results in our article, aiming to better understand the pathogenesis and potential therapeutic targets of the SARS-CoV-2-related neurological disorders.

With the increasing number of SARS-CoV-2 infections, more COVID-19 patients are exhibiting the neurological complications and the long-term neurological sequelae. Our study reveals the potential processes through which COVID-19 affects the susceptibility to the neurological diseases. We identified several common DEGs shared between COVID-19 and various common neurological diseases, and identified several hub genes from these DEGs. We found the common genes regulating the vascular homeostasis between COVID-19 and the cerebrovascular diseases. Additionally, we identified genes such as MAPK14 that may participate in the pathogenesis of COVID-19 and the neurological diseases through the modulation of autophagy and inflammation. We speculate that the molecular mimicry mechanisms leading to the immune response errors and the persistent immune activation contribute to the development of the neurological sequelae. Furthermore, we constructed the gene regulatory networks to predict the shared miRNAs and the transcription factors involved in COVID-19 and the neurological diseases. As COVID-19 remains a global pandemic with no effective treatment, we constructed the protein-drug regulatory networks and predicted that NADH, FADH and phenelzine may be potential drugs for treating COVID-19. In summary, we analyzed the relationship between COVID-19 and the common neurological diseases, elucidated some mechanisms underlying comorbidities, and provided the therapeutic insights for treating the COVID-19 complications and the long-term sequelae.

## 6 Limitations

### 6.1 Influence of age on findings

In our study, we explored the relationship between COVID-19 and various neurological disorders by identifying the common deregulated genes. One critical factor that we did not address in our initial analysis is the potential influence of age. Age is a significant determinant in both the incidence and severity of COVID-19 and the neurological disorders. The immune response, comorbidities and the baseline neurological health vary significantly across the different age groups, which can influence the degree and nature of the neurological complications following the COVID-19 infection.

It is well-documented that older adults are at a higher risk for severe COVID-19 outcomes and have a higher prevalence of the neurological disorders such as AD, PD and stroke. In contrast, the younger populations may exhibit the different neurological manifestations and the disease mechanisms. Therefore, the interactions between COVID-19 and the neurological conditions may differ by the age group.

To provide a comprehensive understanding, the future research should include the age-stratified analyses to determine if the deregulated genes we identified have the age-specific relevance. Such analyses could reveal the unique molecular signatures and pathways in the different age cohorts, potentially leading to the age-targeted therapeutic strategies. Recognizing the variability in the disease mechanisms across the age groups is crucial for developing the tailored interventions and improving the patient outcomes.

### 6.2 Influence of vaccination status on findings

Our study did not differentiate between the vaccinated and unvaccinated individuals when analyzing the common deregulated genes associated with COVID-19 and the neurological disorders. This is an important consideration, as the vaccination status significantly impacts the incidence and severity of COVID-19, and consequently, its neurological complications.

COVID-19 vaccines have been shown to reduce the risk of severe disease, hospitalization and the potentially long-term neurological sequelae. The immune response elicited by vaccination could alter the expression of the genes associated with both COVID-19 and the neurological disorders. Therefore, understanding how vaccination modifies the molecular interactions between COVID-19 and the neurological conditions is essential.

In future studies, including the vaccination status as a variable could elucidate the protective mechanisms conferred by vaccines at the molecular level. This could involve comparing the gene expression profiles of the vaccinated and unvaccinated individuals with the neurological disorders in post-COVID-19 infection. Such an analysis might highlight the vaccine-mediated changes in the gene regulation that contribute to reduced the neurological complications.

By integrating the vaccination status, the future research can provide the deeper insights into the molecular underpinnings of COVID-19’s impact on the neurological health and help refine the public health strategies to protect the vulnerable populations. Recognizing the role of vaccination in modulating the disease outcomes will enhance our understanding and inform the development of the comprehensive treatment approaches.

## 7 Future prospects

The future research could expand on the study parameters by incorporating more diverse datasets, which include the different populations, the geographic areas and the socioeconomic backgrounds. This will help understand how these factors influence the incidence and severity of the neurological complications related to COVID-19. Additionally, there is a pressing need for the longitudinal studies to track the long-term effects on the COVID-19 survivors. Such studies would help identify the persistent or delayed neurological issues and are crucial for understanding the comprehensive impact of the disease over time.

The future studies should also focus on the intervention strategies aimed at alleviating the neurological effects observed in the COVID-19 patients. This includes developing the pharmacological treatments targeted at the specific pathways identified in the research, as well as designing the non-pharmacological interventions, such as the rehabilitation programs, to improve the cognitive and motor functions in post-infection. As more data become available, the further research is required to understand the impact of the different COVID-19 vaccines on preventing the virus-related neurological symptoms. This research is vital for distinguishing the roles of various vaccines in preventing the long-term health consequences of COVID-19.

Based on the findings of the ongoing and future research, the policy adjustments may be necessary to address the neurological impacts of COVID-19. The healthcare system might need to adapt by providing more neurological care resources and training the healthcare professionals to better recognize and treat the neurological symptoms in post-COVID-19.

Finally, integrating the research findings into the public health strategies is crucial. This integration would involve educating the public about the risks of the neurological complications from COVID-19 and implementing the prevention strategies to reduce the spread of the virus and its long-term health effects.

## Data Availability

The original contributions presented in the study are included in the article/Supplementary material, further inquiries can be directed to the corresponding author.
