# Supplementary material for: Potential association between COVID-19 and neurological disorders: analysis of common genes and therapeutics
Source: Front Neurol. 2024 Oct 14;15:1417183. doi: 10.3389/fneur.2024.1417183 (PMC11513677; doi:10.3389/fneur.2024.1417183)
Supplement: Supplementary file 1 [file Table_1.docx]

**Supplementary Table 1**

**Significant differential genes**

| Covid-19 | SD | HS | AD | EP | PD | IS | Covid-Imm |
| --- | --- | --- | --- | --- | --- | --- | --- |
| SNORA74B | MLH3 | MTRNR2L10 | KLF15 | FREM1 | TLR10 | CD6 | IL6R |
| ALB | TMEM176A | INMT | VGF | SUSD2 | OSBPL10 | PDK4 | HLA-DQA1 |
| APOB | SCGB3A1 | PPIEL | ADCYAP1 | FRAS1 | SNORA28 | ARG1 | HLA-DQB1 |
| SAA2 | TMEM176B | RAB3B | SLC6A9 | ERICH1-AS1 | FOS | MAL | ITGB2 |
| ORM2 | HSPC102 | IKZF3 | NRN1 | RP11-627G23.1 | FCRLA | FAIM3 | CD19 |
| VTN | CHP1 | GATM-AS1 | SGO1 | CTD-2047H16.4 | PDXDC2P | IL7R | CD22 |
| HPD | NATD1 | PDE6A | NEUROD6 | COLQ | CD24 | IQGAP1 | MS4A1 |
| TF | APOM | MREG | PRKX | MIR143HG | ABCA7 | CCR7 | CCR5 |
| DPYS | LOC101928343 | IDS | PRMT8 | AC131025.8 | PTPRC | MMP9 | ZAP70 |
| APOA1 | PRR11 | LINC00649 | PPEF1 | RBPMS | LOC90925 | CA4 | TUBB |
| HGD | CUTALP | METTL21A | RPH3A | MUC19 | SNX29 | ACSL1 | PAX5 |
| CYP2E1 | HLA-DQA1 | SLC43A2 | PCSK1 | LAMA5 | FOSB | S100A12 | POLR1B |
| APOH | ACSM2A | EEF2K | JADE3 | TTC21A | JUP | FOLR3 | TNFRSF13C |
| BAAT | SMAGP | FXN | C3orf80 | CTD-2349P21.5 | AMFR | LY96 | ZBTB16 |
| CEACAM1 | HLA-DQB1 | ACBD7 | MID1IP1 | LIMS2 | POU2AF1 | BNIP3L | CD79A |
| GDA | PSME3 | IFNLR1 | LOC102724596 | IQCJ | EGR1 | APOBEC3A | IRF5 |
| SNORD17 | CDK5 | ZNF483 | DNAH11 | ANKRD19P | C16orf7 | ORM1 | AICDA |
| IGF1 | EEF1AKMT3 | FGF5 | GNRH1 | MIR145 | MS4A1 | FCGR3B | IL1RL2 |
| ABCB4 | CD99P1 | FBLIM1 | MAS1 | HSPG2 | TBC1D10B | FTHL3 | CX3CR1 |
| RPS2P36 | HNRNPH3 | ATP5E | SOWAHB | COL14A1 | KLF4 | FTHL11 | CCR1 |
| SLC39A14 | INSIG1-DT | MTRNR2L2 | FAM53B-AS1 | AC093642.3 | CXCR4 | FCGR3A | IL12B |
| RN7SKP9 | MICU1 | GNG4 | MSC | MIAT | STAB1 |  | IL1RAP |
| BHMT2 | SIGLEC9 | LOC284260 | MDH1B | FAM118A | AL359560 |  | FCER1A |
| ARG1 | SLC24A3 | CCL5 | CLDN15 | AKT3-IT1 | LOC728153 |  | C4BPA |
| CAV1 | SERPINB9P1 | GLIPR1L2 | CRH | RP4-673D20.3 | RRP12 |  | HLA-DRA |
| EMP2 | CCEPR | PRELP | FOXO4 | CASQ2 | LOC338817 |  | LAG3 |
| TFR2 | LOC100996756 | SIX4 | ABCC12 | RP4-555D20.2 | POU2F2 |  | C2 |
| PROC | SLC22A3 | CSDE1 | TAC1 | MYOCD | BANK1 |  | CD1A |
| FOSB | LINC02802 | C21orf62 | OTOGL | RP11-159D12.2 | E2F5 |  | NOS2 |
| HIRIP3 | SIRT6 | TMEM154 | USP2-AS1 | CAPG | RP2 |  | EBI3 |
| SLC38A4 | ORM1 | LOC286186 | GCNT4 | RP6-99M1.2 | MOV10 |  | STAT1 |
| UPB1 | DEPDC5 | CACNG8 | BEX5 | GAPDHP66 | TSPAN13 |  | PDCD1 |
| SLC51A | TRIB3 | C4orf26 | NPFF | FGF14-IT1 | WARS |  | IL4 |
| LMO7 | SLC10A3 | VSIG1 | CPM | AC144833.1 | CD79A |  | BST2 |
| PLAC9 | SENCR | MOG | ALOX12B | ROCK1P1 | SF3A1 |  | IFIH1 |
| PTPN21 | ATP8B1 | UBE2Q2P1 | LINC01546 | C20orf203 | TNFAIP2 |  | GP1BB |
| CYP2C18 | ELAPOR1 | CCDC122 | GJD2 | HNRNPCP6 | ID3 |  | IL22RA2 |
| LEAP2 | RITA1 | PAK1 | MGC16025 | RP11-989F5.3 | LOC284837 |  | THY1 |
| AK4 | MAX | PPIL6 | FAM222A | CRYZ | BLK |  | LILRA6 |
| PDCL | CASTOR3 | ELMOD1 | LBX2 | COPS8P2 | NAG8 |  | CCL15 |
| TMEM87A | C4BPA | CYP4V2 | HSPB3 | AOC3 | AK129699 |  | SERPING1 |
| C4BPB | KHNYN | NXN | PCDHGC5 | ZNF410 | ATG16L2 |  | KIR_Activating_Subgroup_1 |
| AGMAT | ANKRD36B | ZFP42 | KRT5 | MIR770 | GNG7 |  | CCBP2 |
| LIMCH1 | PKIG | SPRED1 | STAT4 | CEACAM19 | P2RX1 |  | GNLY |
| FNDC4 | LOC105371215 | MIR143HG | ANKRD18DP | FOXL1 | KLHL14 |  | GBP1 |
| CAB39L | CMAS | ZYG11A | MCHR2 | RP11-379B18.5 | MDFIC |  | C8B |
| TDO2 | ZNF595 | TBXA2R | PNMA3 | PVT1 | CD1C |  | SOCS1 |
| SCN4B | ARF3 | KIAA1456 | TNRC6C-AS1 | ADIRF-AS1 | CPT1B |  | KIR_Activating_Subgroup_2 |
| PRSS12 | IL1RAP | ZNF665 | OVOL3 | RP11-981G7.2 | ZNF679 |  | IL18RAP |
| PTPRH | NCR3 | MTRNR2L4 | C10orf62 | RP11-54A4.2 | DUSP1 |  | IL18R1 |
| TPST1 | AGPAT1 | PJA2 | PPFIBP2 | GJA6P | PARP12 |  | KLRB1 |
| GJB2 | DHFR2 | LOC283335 | LINC01561 | SCUBE3 | X69637 |  | CEACAM1 |
| TMSB4X | LRRC42 | HTRA4 | MPO | RP11-768G7.2 | MORC3 |  | IRF7 |
| AKR7A3 | ACTR1B | IRF2BPL | NAP1L2 | SLC25A34 | CD19 |  | IFI35 |
| PON1 | BEND2 | LOC100287314 | LOC100129316 | AC019118.2 | ERAP2 |  | KLRC1 |
| SCARNA5 | ELOA-AS1 | FKBP14 | IL1RL2 | AC011747.4 | STAT2 |  | ITLN2 |
| MTCO3P23 | MIX23 | LOC90834 | NAP1L5 | H3F3AP4 | STAP1 |  | CCL8 |
| F5 | ELP5 | LOC100129269 | STON1 | LINC00599 | TYMP |  | MX1 |
| AMDHD1 | DUBR | AP1S3 | ADRA2B | RP11-509E10.1 | SAMD9L |  | CLEC5A |
| FGG | UGT2B28 | LOC100288069 | LINC01202 | VWCE | VPREB3 |  | CXCL10 |
| AHNAK | LOC100289333 | BVES | THCAT155 | BTBD19 | FCRL5 |  | IFIT2 |
| ONECUT2 | ENTPD1-AS1 | CEP41 | SST | CTD-2281E23.2 | BBS10 |  | CTSG |
| RTKN2 | RAMP2-AS1 | PTPN14 | NAT16 | RP11-535M15.2 | AK024852 |  | LILRA3 |
| PFKFB1 | DYNLL2 | ZNF713 | CHML | RP11-981G7.1 | GABBR1 |  |  |
| SDS | MRPL49 | AFMID | ZBBX | MIR137HG |  |  |  |
| SND1-IT1 | ADAM33 | OR7D2 | MSC-AS1 | RNF139-AS1 |  |  |  |
| SLC39A5 | RAB37 | MAP1LC3C | PAK1 | PTCH2 |  |  |  |
| PPL | LOC105375547 | LOC100128288 | VSNL1 | ADAMTS15 |  |  |  |
| PNPLA3 | ACADVL | ZNF793 | FBLN7 | CTA-407F11.6 |  |  |  |
| SMPDL3A | LOC105369228 | PTK6 | LOC100507194 | SMYD3-IT1 |  |  |  |
| BEX3 | BRD3OS | NEK5 | MCM7 | ELN |  |  |  |
| AQP9 | PRPF38B | TMEM213 | ADCY10P1 | MKRN5P |  |  |  |
| CP | RIC8A | ATCAY | SCG2 | GNB3 |  |  |  |
| SPRING1 | ECRG4 | ATP5I | HIP1 | RP11-282K24.3 |  |  |  |
| AHR | DDX11L2 | C1orf210 | PVRIG | XXbac-BPG154L12.4 |  |  |  |
| TPPP | H2BC21 | MAPRE1 | RGS4 | C1orf132 |  |  |  |
| SLC22A1 | LOC114224 | LRRN4CL | LINC00898 | AF131215.3 |  |  |  |
| SERPINA1 | TMEM185A | LOC100506085 | SH2D5 | RP11-53B2.2 |  |  |  |
| PPIL6 | ARHGEF1 | METTL8 | CARTPT | RP11-981G7.6 |  |  |  |
| GLS2 | TASL | MTRNR2L3 | SAP25 | CORO6 |  |  |  |
| NOP53 | POM121L8P | RNF207 | CCDC184 | MEG3 |  |  |  |
| HEXIM1 | GNG5 | RABL5 | LDLRAP1 | DNAJC5G |  |  |  |
| CLEC3B | ZNF747 | TMEM236 | ZNF652 | LAMA3 |  |  |  |
| AZGP1 | ACTR1A | JPX | KIF1C | RP11-16E23.4 |  |  |  |
| KCND3 | TBC1D25 | ZNF716 | LIN28B-AS1 | FAM153B |  |  |  |
| ENSG00000269688 | ATG13 | TFDP2 | PKMYT1 | GET4 |  |  |  |
| PIPOX | NIPSNAP1 | NCMAP | SH2D6 | LINC00595 |  |  |  |
| ACSL1 | FKBP1A | RAB42 | GAP43 | TPCN2 |  |  |  |
| FABP5 | BRF2 | ZNF818P | BEX1 | RP11-367O10.1 |  |  |  |
| DEPDC7 | TP53I3 | ZC3H12D | DTHD1 | HIF3A |  |  |  |
| VNN1 | PVALB | LOC440300 | SPATC1 | RP11-397O4.1 |  |  |  |
| SERPINF2 | SH3BGRL3 | PXMP4 | SPTSSB | AL132709.8 |  |  |  |
| RIMKLA | CREB3L4 | PAPL | ZBTB20-AS1 | GOLGA8A |  |  |  |
| FOS | NOTCH2NLA | ZFAS1 | LINC00463 | DSCAM-IT1 |  |  |  |
| CYP21A2 | BRAF | PNPO | CRYM | C6orf163 |  |  |  |
| ANOS1 | DHRS1 | PTPRG-AS1 | UCKL1-AS1 | C10orf113 |  |  |  |
| ZFHX4 | ERMAP | WDR1 | BLID | FGF17 |  |  |  |
| RPS12P26 | LOC105378577 | SEPT14 | OLFM3 | MSS51 |  |  |  |
| ADORA2A-AS1 | ELOVL7 | BCAP31 | LOC101926975 | LINC00106 |  |  |  |
| RAB11FIP1 | HIF1AN | ANKRD16 | SERTAD4-AS1 | RP11-572C21.1 |  |  |  |
| CAV2 | H2BC8 | SCD5 | LOC102724484 | RP11-359E3.4 |  |  |  |
| SMOC1 | TNFAIP8L1 | C17orf75 | MORN3 | C1RL-AS1 |  |  |  |
| APOM | SMIM24 | FAM227A | LCN6 | HLA-DRA |  |  |  |
| SPRYD7 | KIR2DL2 | PNMA2 | ZDHHC23 | CATSPER2 |  |  |  |
| DMGDH | ECSIT | ZNF667 | HMGCS1 | GHRLOS |  |  |  |
| HSD11B1 | HOMER1 | EMP2 | SERPINF2 |  |  |  |  |
| PRG4 | CAVIN3 | RFPL1-AS1 | TLDC2 |  |  |  |  |
| PROX1 | SNN | SCAI | NEAT1 |  |  |  |  |
| HAL | TTC7B | KIAA1324 | ITPKB-IT1 |  |  |  |  |
| HAMP | CNN2 | PACS2 | LOC101929715 |  |  |  |  |
| MLIP | SWI5 | EXPH5 | PLA2G4B |  |  |  |  |
| AOX1 | COL9A3 | DYNC1LI1 | LOC285762 |  |  |  |  |
| ENSG00000278896 | ACD | DNAL1 | ATAD3C |  |  |  |  |
| SEMA5A | TMEM109 | TRIM65 | CYP4B1 |  |  |  |  |
| RPLP1 | C8orf82 | PRND | CALY |  |  |  |  |
| CAVIN2 | MRPL51 | CHRNB1 | LOC102723493 |  |  |  |  |
| CNDP1 | MPV17 | ZNF492 | SYP |  |  |  |  |
| ADH1C | VPS72 | PGM5P2 | TMPRSS5 |  |  |  |  |
| ZCCHC3 | LOC105379362 | BRIP1 | KIF19 |  |  |  |  |
| LIPG | MIF4GD | IBA57 | ARRDC2 |  |  |  |  |
| E2F3-IT1 | TMEM212 | POU5F1 | MYOT |  |  |  |  |
| NR5A2 | SNRPN | SLC5A5 | FZD10-AS1 |  |  |  |  |
| CYP3A5 | PPP1R18 | GDPD1 | LLGL2 |  |  |  |  |
| XDH | DUSP14 | PDP2 | TUBB2A |  |  |  |  |
| ANP32B | ZNF721 | KCNJ5 | PCA3 |  |  |  |  |
| PIK3AP1 | SRXN1 | PLEKHH2 | MCHR1 |  |  |  |  |
| CCDC89 | DET1 | LRRC2 | OLMALINC |  |  |  |  |
| C8G | CTDSP1 | LOC284950 | SERTM1 |  |  |  |  |
| ENSG00000280365 | ERAP2 | C2orf91 | CLDN16 |  |  |  |  |
| MTND5P32 | TMEM241 | AK3 | PCP4L1 |  |  |  |  |
| C1R | TKTL1 | GGT6 | SLC10A1 |  |  |  |  |
| ENSG00000275481 | SNAI3 | SEC14L1 | LYRM9 |  |  |  |  |
| ITIH4 | HADHA | PGAM5 | PTOV1-AS2 |  |  |  |  |
| CCDC115 | RSRP1 | KIAA1875 | OPTC |  |  |  |  |
| PLA2G2A | LOC100507642 | SPG21 | CPLX1 |  |  |  |  |
| PBXIP1 | LPIN2 | LOC100128338 | VCAN-AS1 |  |  |  |  |
| COL28A1 | MACF1 | CYFIP2 | FANCB |  |  |  |  |
| SORD | GUSBP14 | UBL5 | HTR3B |  |  |  |  |
| AMIGO2 | PDLIM1 | C1orf56 | STYK1 |  |  |  |  |
| ACTG1P3 | TNFSF12 | CCNI | TUBB3 |  |  |  |  |
| SRD5A1 | SSBP3-AS1 | SGCB | OR7A5 |  |  |  |  |
| PCSK6 | PRSS33 | ARHGEF26-AS1 | LINC01168 |  |  |  |  |
| ALDH8A1 | TAL1 | FPR2 | LOC728084 |  |  |  |  |
| PPP1R14A | GCHFR | ARF1 | ARL4D |  |  |  |  |
| BRF2 | GPS2 | OPA3 | NRON |  |  |  |  |
| ALDH1L1 | MID1IP1 | SLC4A8 | MIR6717 |  |  |  |  |
| MT-RNR2 | POLR2J | AARS2 | PSG8 |  |  |  |  |
| MUC3A | CPTP | LOC283299 | ATOH7 |  |  |  |  |
| ZNF704 | CAPNS1 | LIN28A | MAP4K4 |  |  |  |  |
| RIDA | DEFB108B | MTRNR2L8 | HIF3A |  |  |  |  |
| SEC24D | DNAJB12 | NEK2 | KMO |  |  |  |  |
| LIPC | ZSWIM3 | TMEM136 | BAALC-AS1 |  |  |  |  |
| SERPINA3 | GOLGA8IP | NR1H2 | LINCR-0002 |  |  |  |  |
| MTSS1 | CHID1 | MYLK3 | CXCR1 |  |  |  |  |
| PDSS1 | LIPA | CA5B | KCNB2 |  |  |  |  |
| PATL1 | SPARC | C3orf72 | C6orf223 |  |  |  |  |
| HSD17B2 | RANGRF | CRX | LINC01007 |  |  |  |  |
| ABAT | LOC101927770 | FAM73A | LINC00460 |  |  |  |  |
| PHAF1 | LINC01809 | MANEAL | LOC100288637 |  |  |  |  |
| KANK2 | P2RX1 | LINC00294 | HES5 |  |  |  |  |
| ADAMTS9-AS1 | KPNA1 | WDR92 | LOC100130548 |  |  |  |  |
| MDH1B | SLC29A3 | FOXK1 | SAMD11 |  |  |  |  |
| ENSG00000243696 | POLDIP2 | TSTD3 | GABRA1 |  |  |  |  |
| MYADM | ARSG | SIGLEC8 | GAD1 |  |  |  |  |
| TMEM176A | CHI3L2 | LOC728606 | GFRA2 |  |  |  |  |
| ANXA3 | WIF1 | ZNF714 | SCHLAP1 |  |  |  |  |
| SCARNA21 | LOC729732 | WDR17 | GAD2 |  |  |  |  |
| ASGR1 | MMP24OS | MAP7D3 | RIIAD1 |  |  |  |  |
| CHCHD6 | DESI1 | TYW5 | LRRC73 |  |  |  |  |
| PPP1R1A | GPX7 | AQP6 | FAM222A-AS1 |  |  |  |  |
| CHI3L1 | ASB6 | GLUD1P7 | IGF1 |  |  |  |  |
| ENSG00000259071 | FBXW12 | TMEM130 | ADAMTS3 |  |  |  |  |
| RPL23AP34 | SHISA4 | LARS2-AS1 | SLC23A3 |  |  |  |  |
| HELLPAR | COX2 | PPARA | MRGPRF |  |  |  |  |
| ACSM5 | H2AC11 | DZIP3 | FREM3 |  |  |  |  |
| TBX15 | PI4KB | MTX3 | LINC01164 |  |  |  |  |
| LTBP4 | RETREG2 | LOC728558 | BOK |  |  |  |  |
| CDH2 | SLC9B1 | LOC100287792 | LINC01445 |  |  |  |  |
| TNXB | OR52K3P | C7orf55 | LINC00601 |  |  |  |  |
| UPK3B | ND4 | UTY | LOC105378385 |  |  |  |  |
| ETV5 | MAP1S | IFITM3 | QDPR |  |  |  |  |
| KLKB1 | RILP | PCDHB9 | KIRREL3-AS3 |  |  |  |  |
| PLIN1 | YY1AP1 | CD93 | PRSS8 |  |  |  |  |
| CA11 | PCLAF | NOS1 | PTPRD-AS2 |  |  |  |  |
| LPAL2 | ALDOC | PLEK | LOC339685 |  |  |  |  |
| RIPOR3 | MEN1 | LOC100128531 | SNAP25 |  |  |  |  |
| VEGFD | CIDEB | ANKS4B | CCKBR |  |  |  |  |
| TSHZ2 | RNF185 | ARNTL2 | PLPP2 |  |  |  |  |
| ENSG00000279662 | CMTM5 | SLC7A14 | CDK18 |  |  |  |  |
| INMT | LINC00328 | AKIP1 | MDH1 |  |  |  |  |
| ANXA1 | TSHZ3 | CYP27C1 | COL5A2 |  |  |  |  |
| HTATSF1 | LOC105376486 | FKBP8 | MAMDC4 |  |  |  |  |
| ENSG00000279573 | TRIM26 | RAD23B | FAM81A |  |  |  |  |
| ENSG00000278601 | COPS6 | ORC6 | OLAH |  |  |  |  |
| STX1B | ZNF394 | FBXO45 | SLC12A1 |  |  |  |  |
| PCDHA12 | RBM22 | FAM200B | ABCA6 |  |  |  |  |
| RN7SKP80 | TBC1D10B | NUP43 | ENC1 |  |  |  |  |
| ABCC11 | CNPPD1 | SMAD5 | AMIGO2 |  |  |  |  |
| RPL7AP16 | RAB43 | BMP8A | ELN |  |  |  |  |
| NPAS2 | ORAI3 | HEXIM1 | EGFR-AS1 |  |  |  |  |
| FAM169B | MPST | DNAJB12 | BMP3 |  |  |  |  |
| SNORD89 | ELF4 | S1PR2 | CA3-AS1 |  |  |  |  |
| GATM | CCDC159 | ZNF542 | MAN2A1 |  |  |  |  |
| CYP4F3 | SIRPB1 | ZNF69 | PPP1R2P3 |  |  |  |  |
| GLRX | EFNA4 | KIAA1467 | TRPA1 |  |  |  |  |
| SEC14L2 | XNDC1N | EXOSC6 | CLDN34 |  |  |  |  |
| RASSF9 | CDC42EP2 | DNAJC22 | SMIM5 |  |  |  |  |
| CS | MXRA7 | MTRNR2L1 | C9orf139 |  |  |  |  |
| GGA2 | RCOR3 | VSTM4 | INHBA-AS1 |  |  |  |  |
| CYP7B1 | GMDS-DT | ADAM17 | F3 |  |  |  |  |
| EEF1A1P11 | LYSMD2 | SBF2-AS1 | MIR5690 |  |  |  |  |
| SNORA37 | UTP4 | DUSP19 | CD244 |  |  |  |  |
| FAM110D | CYB561D2 | PCDH11Y | GZMM |  |  |  |  |
| NEK11 | RTL8C | NUDT19 | MDGA1 |  |  |  |  |
| STEAP1 | LOC100505874 | DTD2 | LINC01257 |  |  |  |  |
| RPS6KA5 | H3C10 | BHMT2 | CLEC18A |  |  |  |  |
| OXA1L | KLHDC3 | TMEM241 | DLX6-AS1 |  |  |  |  |
| SERPINA5 | CAPG | CALCOCO2 | OLFM4 |  |  |  |  |
| MGST1 | TBC1D20 | AMPD2 | LOC101929754 |  |  |  |  |
| BAG1 | RELA | FTH1P3 | NPY2R |  |  |  |  |
| RSPH1 | HEXIM2 | EIF4H | MIR3619 |  |  |  |  |
| ENSG00000279500 | LOC105377458 | GNPNAT1 | RAD21L1 |  |  |  |  |
| CAVIN1 | ESF1 | RPS6KA6 | MIR1250 |  |  |  |  |
| PTGR1 | LOC100190986 | FOXRED2 | SELE |  |  |  |  |
| LRG1 | PFAS | PCDHA9 | PRSS35 |  |  |  |  |
| H2BC20P | TRAF3IP3 | ICA1L | SFTPA1 |  |  |  |  |
| FAM184A | SHISA5 | S100A8 | LOC100505912 |  |  |  |  |
| GPAM | RNASEH2A | DNAJB6 | LINC01336 |  |  |  |  |
| RIBC1 | H2AC8 | DSCR6 | TGFBR3L |  |  |  |  |
| C1orf198 | LINC02754 | IL10RB | LOC339529 |  |  |  |  |
| DESI1 | ND2 | THBD | LOC101927043 |  |  |  |  |
| HMGN5 | VPS26B | SLC25A15 | ANO3 |  |  |  |  |
| SLC17A9 | CLK4 | IGFBPL1 | PRKCG |  |  |  |  |
| LINC02362 | ANKRD20A12P | LRIF1 | LOC101929151 |  |  |  |  |
| TMOD3 | LILRA3 | KATNBL1 | LINC01107 |  |  |  |  |
| EFR3B | TOX2 | ZNF566 | SGCA |  |  |  |  |
| ADIRF | TTI2 | ATP8B1 | ABCA8 |  |  |  |  |
| HS3ST3B1 | LRRC75A | C1orf86 | SNORA104 |  |  |  |  |
| CRYZL2P-SEC16B | USP30 | LRRFIP1 | FAAHP1 |  |  |  |  |
| SLC27A5 | ZNF831 | LOC284023 | RAB3A |  |  |  |  |
| GOLT1A | MIR3682 | CCDC113 | LOC100506136 |  |  |  |  |
| P3H2 | RMDN3 | PDIA3 | SNX10 |  |  |  |  |
| CLXN | TAGLN2 | SKAP2 | NGFR |  |  |  |  |
| SURF6 | PRDX6 | BCL2L15 | CCR6 |  |  |  |  |
| CCDC170 | ACTR3BP2 | CTSS | SYT10 |  |  |  |  |
| METTL17 | NDUFB1 | FLNA | DHH |  |  |  |  |
| NECAB1 | SNIP1 | IGBP1 | ANKRD40 |  |  |  |  |
| ITM2A | PPP5D1P | LINC00663 | MIR340 |  |  |  |  |
| WDR72 | DENND4A | DBT | C11orf87 |  |  |  |  |
| LINC01018 | TRIM35 | AKIRIN1 | SMTN |  |  |  |  |
| ABCC3 | C9orf64 | IL1B | MND1 |  |  |  |  |
| DNAH10 | RASEF | PLEKHO2 | IL33 |  |  |  |  |
| TMPRSS6 | CCDC18-AS1 | VAMP3 | LRRC46 |  |  |  |  |
| UNC5CL | ZC3H12A | RAD1 | LGALS2 |  |  |  |  |
| GNMT | TUBA3D | TNFAIP6 | DOCK5 |  |  |  |  |
| SLC25A15 | ACKR4 | TNIP1 | PRR34-AS1 |  |  |  |  |
| ENSG00000261338 | LGALS3BP | COX7B | CXCR2 |  |  |  |  |
| ENSG00000260310 | TSPAN33 | TMF1 | DLX6 |  |  |  |  |
| ANKRD29 | TIPARP | HIST2H2AC | PART1 |  |  |  |  |
| SLC25A18 | DDX41 | HNRNPL | LOC101927139 |  |  |  |  |
| ZNF440 | SULF2 | CHP2 | THRSP |  |  |  |  |
| GOT1 | HLA-DRB4 | PSME3 | SNORD114-21 |  |  |  |  |
| CES3 | S1PR1-DT | PUM2 | CLCA4 |  |  |  |  |
| ZCWPW2 | NOP58 | LOC400891 | CLMN |  |  |  |  |
| IL17RB | ALG8 | MRI1 | ADORA2A |  |  |  |  |
| MGAT3 | PPIG | GP2 | CHRNA7 |  |  |  |  |
| RN7SL4P | LOC389834 | GPLD1 | TCP11 |  |  |  |  |
| MAGI3 | STARD10 | MTRNR2L6 | HILS1 |  |  |  |  |
| CPA3 | TIMM8A | GRM6 | BGLAP |  |  |  |  |
| CRACDL | TUBB4B | CD177 | BOK-AS1 |  |  |  |  |
| C2orf50 | TRA2A | AKAP5 | GADD45A |  |  |  |  |
| C11orf24 | CDK4 | SHISA2 | OVCH2 |  |  |  |  |
| AQP7P1 | SLC25A11 | SYNGR2 | RDH12 |  |  |  |  |
| ENTPD5 | F8 | BAG6 | SH2D3A |  |  |  |  |
| SLC35D1 | TUSC2 | SLC35E2 | LOC100506274 |  |  |  |  |
| MOCOS | PELI3 | ELL | IRX2 |  |  |  |  |
| ENSG00000276980 | MCUR1 | TNFAIP8L3 | GMNC |  |  |  |  |
| SNORD3A | SUV39H1 | NUP98 | MUC3A |  |  |  |  |
| KCND3-IT1 | MIPEPP3 | DKFZP434L187 | KCNV1 |  |  |  |  |
| DRC3 | LOC101927018 | TRIM59 | MIR2467 |  |  |  |  |
| HIC2 | PIP5K1C | ABHD2 | LMNTD2 |  |  |  |  |
| GPIHBP1 | COX3 | OR51E2 | FAM21EP |  |  |  |  |
| SAC3D1 | MAP3K1 | C3orf33 | SALRNA2 |  |  |  |  |
| PPP1R9A | RUNDC3A | ATP6V0C | FOXD2 |  |  |  |  |
| SYT12 | IP6K2 | ATP6AP2 | C11orf94 |  |  |  |  |
| PSAT1 | SIGLEC5 | SF3B2 | LINC00323 |  |  |  |  |
| PTPN14 | TMEM164 | TMEM192 | BMP7-AS1 |  |  |  |  |
| PC | CARM1 | PTGS2 | PRTN3 |  |  |  |  |
| BMS1P10 | FRG1BP | TUBA3FP | GABRG2 |  |  |  |  |
| DNAI4 | FHL3 | PRKX | SLC16A6 |  |  |  |  |
| ABCC2 | MALAT1 | INADL | LOC108783645 |  |  |  |  |
| LRRC23 | MED8 | DBNL | FOXD1 |  |  |  |  |
| OAF | PRAF2 | IL10 | SPDEF |  |  |  |  |
| DGAT2 | GBGT1 | ITIH5 | LINC00484 |  |  |  |  |
| IER2 | TIMP1 | FTH1 | SLC30A3 |  |  |  |  |
| IRX3 | HEY1 | TPM4 | PIK3C2G |  |  |  |  |
| SMYD3-IT1 | FKSG49 | PPP1R15B | LOC101928766 |  |  |  |  |
| ENSG00000280042 | GFI1B | BRAT1 | CPAMD8 |  |  |  |  |
| SLC9B2 | MLLT11 | LINC00598 | NLRP14 |  |  |  |  |
| TMEM45A | ZSCAN22 | PARK2 | LINC01476 |  |  |  |  |
| CFAP43 | CIZ1 | RNF13 | RND1 |  |  |  |  |
| ENSG00000227200 | XKR8 | ZNF260 | FMO3 |  |  |  |  |
| ENSG00000279878 | NKIRAS2 | TPTE2P1 | LOC100288866 |  |  |  |  |
| INSIG1 | CHAMP1 | C14orf2 | LOC101927406 |  |  |  |  |
| ADRB1 | TMEM141 | LOC284551 | TP73 |  |  |  |  |
| MST1P2 | MGLL | DUSP6 | LINC01119 |  |  |  |  |
| MLXIPL | GNAZ | ZNF681 | C1QL3 |  |  |  |  |
| SUGCT | GOLGA6L2 | LPIN3 | FIBCD1 |  |  |  |  |
| ABLIM3 | NEK2-DT | IL1RAP | CLDN9 |  |  |  |  |
| TRIM71 | UCP2 | C9orf85 | LOC105377448 |  |  |  |  |
| LINC01252 | DHRS7B | TMEM181 | UHRF1 |  |  |  |  |
| H2BC19P | TMEM9B | TMEM19 | LINC01296 |  |  |  |  |
| MASP1 | WASHC5 | STX4 | ROR1-AS1 |  |  |  |  |
| ENSG00000261270 | ARFIP2 | LOC151475 | TSG1 |  |  |  |  |
| IRAK2 | LOC100505915 | HCAR2 | GRP |  |  |  |  |
| MT-RNR1 | SSNA1 | TTC39C | NEFL |  |  |  |  |
| RPS16 | OS9 | KDM6A | FFAR4 |  |  |  |  |
| TTC39C | LOC654780 | CD82 | CHGB |  |  |  |  |
| PSTPIP2 | ZBED5-AS1 | CEBPD | HPN-AS1 |  |  |  |  |
| TMIGD3 | VAT1 | KIF3A | FERMT1 |  |  |  |  |
| SLC25A13 | KLF3 | LINC00665 | NEK2 |  |  |  |  |
| TMEM45B | PTDSS1 | ARHGAP15 | LOC101929473 |  |  |  |  |
| EID1 | KAT5 | EIF4A1 | ANLN |  |  |  |  |
| LGALS4 | ZNF793 | ATP1B2 | GSN-AS1 |  |  |  |  |
| LRP4 | XRCC2 | XRCC6 | HPN |  |  |  |  |
| TMEM176B | PNISR | MAPK14 | FHL5 |  |  |  |  |
| H4C4 | BAP1 | DCUN1D2 | CACNG1 |  |  |  |  |
| RAB11FIP4 | MAP2K2 | LOC100130954 | FAM86B3P |  |  |  |  |
| UBTD1 | PRMT5 | RNF40 | TMEM215 |  |  |  |  |
| FAU | TMEM41B | NFKB2 | MIR1914 |  |  |  |  |
| IFT140 | TCTA | FAM157B | LOC149684 |  |  |  |  |
| DCAF8 | PLEK | HCAR3 | VGLL3 |  |  |  |  |
| VSIG2 | ADRM1 | CST7 | SOX8 |  |  |  |  |
| NEURL3 | MICALL1 | AXL | ZNF90 |  |  |  |  |
| ASS1 | DUSP23 | CARD16 | TMC6 |  |  |  |  |
| TOGARAM2 | MZT2B | PTAFR | HIPK2 |  |  |  |  |
| SOD2 | SLC48A1 | METTL2B | LINC00567 |  |  |  |  |
| NRSN2-AS1 | ALOX12 | NFE2L2 | MAGI1-AS1 |  |  |  |  |
| LYZ | LRRC69 | FGL2 | TINAG |  |  |  |  |
| SLC43A3 | TMEM179B | SF3A1 | LOC100505635 |  |  |  |  |
| ENSG00000093100 | SYNGR3 | SNW1 | NSF |  |  |  |  |
| TRIM31 | ZNF213 | SWSAP1 | LOC101927410 |  |  |  |  |
| ARMC12 | BIK | ENTPD3-AS1 | LOC100507616 |  |  |  |  |
| NKD1 | MEIS1 | CNEP1R1 | C10orf128 |  |  |  |  |
| BCAM | IL32 | GLUL | DKFZP434K028 |  |  |  |  |
| ENSG00000241886 | TMEM185B | DNASE1 | SLC26A5 |  |  |  |  |
| TMEM220-AS1 | PTGS1 | SURF4 | INHA |  |  |  |  |
| ENSG00000268573 | SH3GLB2 | C19orf35 | ALG1L |  |  |  |  |
| RPS3 | DEDD2 | LOH12CR2 | KRT19 |  |  |  |  |
| ENSG00000225092 | KCTD21 | P4HB | TFCP2L1 |  |  |  |  |
| SRSF7 | FANCG | ROMO1 | ELMO1-AS1 |  |  |  |  |
| PRDX5 | TCP11L2 | ADAM10 | C5orf38 |  |  |  |  |
| MNS1 | ZFYVE27 | PTMA | ANKRD22 |  |  |  |  |
| WNT9A | HSP90AA1 | GTPBP3 | SHISA2 |  |  |  |  |
| PAK4 | INPP5K | CNNM3 | FAM19A4 |  |  |  |  |
| RN7SKP71 | ASF1B | DPYSL5 | ACP7 |  |  |  |  |
| PPP1R3C | TMEM158 | CHPF2 | C10orf90 |  |  |  |  |
| MT-CO2 | H2BC7 | HSPA8 | LOC100128554 |  |  |  |  |
| CDA | ABHD14A | SGK1 | C7orf61 |  |  |  |  |
| SEMA3B | CHMP6 | LOC727896 | CLCN1 |  |  |  |  |
| BCL7C | SRGAP2B | RAB11FIP4 | LOC441052 |  |  |  |  |
| NGFR | LGALSL | SULT2A1 | KRT81 |  |  |  |  |
| ENSG00000254676 | ZNF296 | ATP2B1 | LOC642943 |  |  |  |  |
| ENSG00000213058 | MGC70870 | DPY19L1P1 | LOC400997 |  |  |  |  |
| H3P31 | GET3 | CIB1 | SGK1 |  |  |  |  |
| TMEM131L | FCGR2B | SCAMP2 | TPTEP1 |  |  |  |  |
| BABAM2 | PPM1M | HDLBP | DEPDC1 |  |  |  |  |
| TSR2 | NEMF | SOD2 | VIP |  |  |  |  |
| HCFC1R1 | BAG6 | TIMP1 | LINCR-0003 |  |  |  |  |
| SERPING1 | LYL1 | EIF3A | PROK2 |  |  |  |  |
| FOXO4 | TRAPPC1 | SLC22A18 | IL16 |  |  |  |  |
| HMGN3 | NDUFAF3 | LPCAT1 | LOC388780 |  |  |  |  |
| PDXDC2P-NPIPB14P | NGDN | GAK | IRX1 |  |  |  |  |
| LCN2 | CAPN12 | LOC100505876 | LOC105376360 |  |  |  |  |
| ENSG00000261553 | SLC35A4 | PPIG | LOC100507472 |  |  |  |  |
| GCLC | BCAS3 | IL1RN | ADAMTS2 |  |  |  |  |
| IL17RE | SH3GL3 | IL8 | SLC45A3 |  |  |  |  |
| SEC24A | SLC38A5 | BCDIN3D-AS1 | PTER |  |  |  |  |
| ITCH-IT1 | KCTD2 | ATP6V0D2 | LOC284933 |  |  |  |  |
| LETMD1 | MAPK14 | BTF3 | MROH5 |  |  |  |  |
| NID1 | PPP1R15A | PPCDC | LINC01108 |  |  |  |  |
| ID4 | DCTN1 | ZC3H12A | IFNL2 |  |  |  |  |
| WDR17 | RNF41 | TNFRSF14 | SPINT1 |  |  |  |  |
| ERBB4 | TANGO2 | CMTM6 | CHADL |  |  |  |  |
| ENSG00000270120 | ZNFX1 | ARSG | CCDC189 |  |  |  |  |
| BLVRA | ORMDL2 | NPC2 | ASB9P1 |  |  |  |  |
| SLC43A1 | NUDT2 | DNAJB14 | H1FNT |  |  |  |  |
| USP27X | NT5M | IRAK1 | MUC16 |  |  |  |  |
| TSTD1 | VKORC1 | RAB2B | CTD-2201E9.1 |  |  |  |  |
| SOCS2 | LINC01949 | CCDC90A | CDR1 |  |  |  |  |
| ITIH5 | CYP27A1 | SNRNP200 | MIR3612 |  |  |  |  |
| POLR3H | LINC00597 | TMCC3 | DLX5 |  |  |  |  |
| LMNTD2-AS1 | H2AW | PAR-SN | PNOC |  |  |  |  |
| ELOVL6 | POLL | HSP90B1 | IGF2 |  |  |  |  |
| ENSG00000265421 | CBX3 | GPCPD1 | ENPP6 |  |  |  |  |
| MTURN | TRIM10 | EPS15L1 | AZGP1 |  |  |  |  |
| DNALI1 | COG7 | HIST1H2BC | RPLP0P2 |  |  |  |  |
| PTMA | ARHGAP6 | CHD1 | KIF2B |  |  |  |  |
| HEY1 | SMIM3 | GBP5 | ZFPM2-AS1 |  |  |  |  |
| PAPSS1 | MORN3 | RRP15 | LOC100507144 |  |  |  |  |
| LINC01852 | POLA1 | INSIG1 | PCDH8 |  |  |  |  |
| SLC16A4-AS1 | MAP2K3 | KDM4A-AS1 | SLED1 |  |  |  |  |
| SLC35G1 | PCGF5 | THAP9-AS1 | LCN10 |  |  |  |  |
| NFS1 | TNFAIP3 | TUBA1A | LMNTD1 |  |  |  |  |
| CDC42BPG | TADA2B | SPRYD7 | SELENOP |  |  |  |  |
| ENSG00000272369 | NRGN | UCKL1-AS1 | LINC01470 |  |  |  |  |
| SLC9A8 | UBAC1 | ESRG | SFRP1 |  |  |  |  |
| SNCG | TSPYL5 | PGM2L1 | CLDN7 |  |  |  |  |
| MTCO3P12 | PODNL1 | NUCB1 | MYBPC2 |  |  |  |  |
| STEAP3 | MCAT | CEBPB | CBX3P2 |  |  |  |  |
| C1orf35 | CDKN1A | CCDC30 | LINC01361 |  |  |  |  |
| COPS9 | CYB5R3 | FAM41C | TESPA1 |  |  |  |  |
| NSUN6 | SLC25A39 | DNAJC27-AS1 | G0S2 |  |  |  |  |
| RPS10 | WDTC1 | ARAF | HECTD2-AS1 |  |  |  |  |
| CYP21A1P | MIR181A2HG | PLAUR | ADORA2A-AS1 |  |  |  |  |
| ENSG00000273451 | RTL6 | SMARCA5 | LINC00390 |  |  |  |  |
| DENND2A | RNF19B | ZNF526 | LINC01378 |  |  |  |  |
| ETNK2 | SHARPIN | TMBIM1 | LOC101929719 |  |  |  |  |
| GAMT | FBXO7 | CANT1 | RAB27B |  |  |  |  |
| BFSP1 | HECTD3 | CNIH4 | RASL11B |  |  |  |  |
| MPHOSPH8 | GYPC | PDE4B | SLC30A8 |  |  |  |  |
| GOT2 | BAK1 | SUPT20H | ZNF341-AS1 |  |  |  |  |
| ENSG00000280384 | HPS1 | BCAS2 | LMX1B |  |  |  |  |
| NTAN1P2 | IMP4 | AZIN1 | IP6K3 |  |  |  |  |
| SPN | PGAP2 | COPB1 | LCN2 |  |  |  |  |
| DNAI7 | TSPAN2 | SERINC3 | RNASE2 |  |  |  |  |
| ZNF486 | CCNI | SLC3A2 | TAC3 |  |  |  |  |
| TMEM92 | POLD4 | MX2 | C17orf102 |  |  |  |  |
| ENSG00000258561 | TNFSF4 | EIF5A2 | LOC101927844 |  |  |  |  |
| EHHADH | ENDOD1 | SQSTM1 | TPH1 |  |  |  |  |
| CROCC | SMIM1 | HIST2H2BF | EGR3 |  |  |  |  |
| TFG | CRTC2 | LOC338758 | TPO |  |  |  |  |
| SNORA7B | DMTN | EIF4A3 | LCN15 |  |  |  |  |
| CETN2 | DHRSX | TPT1 | SPTBN5 |  |  |  |  |
| ST6GAL1 | MARCHF2 | STRN3 | TSPAN10 |  |  |  |  |
| FMO2 | SIGLEC17P | FOSB | VNN1 |  |  |  |  |
| SRPK1 | ZFYVE21 | TM9SF1 | MIR4534 |  |  |  |  |
| SCN8A | STK11 | USP16 | PKHD1L1 |  |  |  |  |
| TSPAN12 | IPO11 | AP2M1 | LENEP |  |  |  |  |
| CGN | IRF2 | HMGN2 | SLC5A11 |  |  |  |  |
| TLE5 | TST | CSRNP1 | MIR3139 |  |  |  |  |
| LRRC1 | GCDH | NLN | DLX4 |  |  |  |  |
| ENSG00000270116 | KRT18 | CASP10 | IQGAP3 |  |  |  |  |
| ZSWIM9 | IP6K1 | ALDOA | LRP2 |  |  |  |  |
| MTCH1P1 | ZBED6CL | SSFA2 | RTKN |  |  |  |  |
| HGFAC | NELFE | CDK5RAP3 | POU2AF1 |  |  |  |  |
| JHY | TUBB3 | SLC4A1 | TPH2 |  |  |  |  |
| MTCH2 | CALCOCO1 | TREM1 | KLHDC7B |  |  |  |  |
| SLC13A3 | CPA3 | HPSE | LOC101059915 |  |  |  |  |
| PON3 | MYG1 | CD44 | LINC01512 |  |  |  |  |
| MT-CO3 | DHPS | RPL10 | HAPLN2 |  |  |  |  |
| FAM110C | TICAM1 | LOC606724 | OR2D3 |  |  |  |  |
| OVGP1 | B9D2 | VPS26A | CR1 |  |  |  |  |
| S100A4 | TOMM40L | CHMP2B | TNN |  |  |  |  |
| SLC7A2-IT1 | POTEM | MED25 | LINC00926 |  |  |  |  |
| HIPK2 | TUBG1 | PPIF | ADGRG5 |  |  |  |  |
| SEC61A2 | MYADM | FCHO2 | LAMA5-AS1 |  |  |  |  |
| LINC01138 | GPR160 | APLP2 | NKX6-2 |  |  |  |  |
| PLD5P1 | CXCR6 | S100A12 | LOC340090 |  |  |  |  |
| ENSG00000276934 | OST4 | ICAM1 | LRRC63 |  |  |  |  |
| ENSG00000279433 | CD19 | PGK1 | MIR657 |  |  |  |  |
| TUBB4BP2 | KIAA0040 | ACOT13 | F2 |  |  |  |  |
| MT-CO1 | TAFA1 | RPL3 | ZIC3 |  |  |  |  |
| PARD6B | NRM | SYTL3 | GRK7 |  |  |  |  |
| NDUFV2 | C2orf88 | HSPA5 | SCUBE1 |  |  |  |  |
| DANCR | PLA2G15 | ZNF780B | GPA33 |  |  |  |  |
| ENSG00000267904 | TPST2 | ATP7A | CCKAR |  |  |  |  |
| HSPE1P26 | CCRL2 | RHBDD2 | LOC100129940 |  |  |  |  |
| MTCO1P12 | TMEM44-AS1 | SIPA1L1 | C4orf26 |  |  |  |  |
| EMCN | STIMATE | PAXBP1-AS1 | SLN |  |  |  |  |
| MAPK14 | HEBP1 | IRS2 | GDNF |  |  |  |  |
| MIR23AHG | TTC14 | ATAD3C | LGR5 |  |  |  |  |
| LRRIQ1 | CYSTM1 | CSTA | MIR4296 |  |  |  |  |
| STX18-IT1 | SLAMF6 | TNFAIP2 | ZBTB46-AS1 |  |  |  |  |
| SNORA12 | DPF2 | LOC100505783 | PTPRQ |  |  |  |  |
| ENSG00000287002 | PKIA | VPS37B | LOC105371789 |  |  |  |  |
| IRAK1BP1 | SURF2 | MATR3 | RAB3C |  |  |  |  |
| SDHAF4 | FAM153B | KDM5B | SFTPA2 |  |  |  |  |
| ZNF578 | TMCO6 | ADPGK | PTF1A |  |  |  |  |
| TNFRSF11B | STING1 | SNX13 | DNAJC5G |  |  |  |  |
| FTLP2 | CCR3 | PSMC4 | MIR4300HG |  |  |  |  |
| TMTC2 | TM7SF2 | H6PD | MIR4311 |  |  |  |  |
| CLDN1 | ARL2BP | ATP6V0A2 | LOC100506271 |  |  |  |  |
| RPS15AP10 | LRRTM2 | ZNF548 | C16orf90 |  |  |  |  |
| PLEKHB1 | CHTF8 | SNN | PRKXP1 |  |  |  |  |
| MYL6 | CDC34 | HSH2D | GDF2 |  |  |  |  |
| ZNF428 | PIN4 | ZFP36L2 | CA9 |  |  |  |  |
| NES | LINC00919 | FXR1 | LINC01088 |  |  |  |  |
| HM13 | BMX | RNASET2 | PRIMA1 |  |  |  |  |
| CHORDC1P4 | CSNK2A1 | KLF10 | ST18 |  |  |  |  |
| LINC01347 | GBAP1 | NUB1 | OR7C1 |  |  |  |  |
| UQCC1 | LINC00342 | GTF2H2B | LOC100287072 |  |  |  |  |
| SLIT2 | DENND1A | ZSWIM1 | OR14I1 |  |  |  |  |
| LINC01290 | FKBP8 | NAIP | CD22 |  |  |  |  |
| SORD2P | NEU1 | MRVI1 | LINC00514 |  |  |  |  |
| SERPINE1 | SNHG16 | ARIH2 | SNRPD2P2 |  |  |  |  |
| SHFL | FAM117A | LDHA | LINC00937 |  |  |  |  |
| SURF4 | TMEM140 | PMAIP1 | KANK4 |  |  |  |  |
| CD52 | LINC02256 | TSPO | FAM95A |  |  |  |  |
| MT1F | ABCD1 | BNIP3L | ARL5C |  |  |  |  |
| PSMA2 | MICOS10P1 | FFAR2 | PIEZO2 |  |  |  |  |
| CD36 | NINJ1 | POLDIP3 | MCCD1 |  |  |  |  |
| RPL7L1P3 | SHMT2 | MASTL | FOXR1 |  |  |  |  |
| CAVIN3 | AGAP9 | NCOA4 | PCSK6-AS1 |  |  |  |  |
| RGS6 | DUXAP8 | TRIM66 | LOC100130331 |  |  |  |  |
| MED14 | GFUS | CD68 | ADAM29 |  |  |  |  |
| UBE4A | PEF1 | MICU1 | DUSP4 |  |  |  |  |
| SLC19A1 | TGFB1I1 | RELA | PCSK6 |  |  |  |  |
| OR7A19P | TREML2 | SERPINB9 | C1orf195 |  |  |  |  |
| SNORD94 | VWCE | ARF4 | MIR6758 |  |  |  |  |
| ACAD8 | GNPDA1 | TMED9 | FAM163A |  |  |  |  |
| RPS3A | TSSC4 | TMEM45B | CT55 |  |  |  |  |
| ENSG00000269967 | C15orf39 | AFTPH | LINC00664 |  |  |  |  |
| IDE | ZFAND3 | MIF | TMEM235 |  |  |  |  |
| MYO1C | RNF10 | SUPT6H | LOC101929613 |  |  |  |  |
| FNDC3B | SH3RF3 | LPIN2 | MIR6871 |  |  |  |  |
| JUND | EPHA1-AS1 | MRPL30 | LINC00320 |  |  |  |  |
| IGSF3 | ADIPOR1 | DNAJB11 | GFRA3 |  |  |  |  |
| FADS1 | UVRAG | PBXIP1 | MIR4479 |  |  |  |  |
| ENSG00000278983 | KAT7 | ZNF527 | LINC01219 |  |  |  |  |
| UBXN1 | FOS | RPL14 | MEIS1-AS2 |  |  |  |  |
| ACVR1B | ZC3HC1 | TYMP | LDHAL6B |  |  |  |  |
| CD177 | COPZ1 | TNFRSF1B | KCNJ15 |  |  |  |  |
| TMEM117 | LINC02076 | RPL11 | LOC101928738 |  |  |  |  |
| LAMA3 | DAD1 | IVNS1ABP | ASB2 |  |  |  |  |
| CHST15 | PINK1 | KDELR2 | LOC102723895 |  |  |  |  |
| GRINA | BCKDK | KLHL2 | LINC01117 |  |  |  |  |
| TMEM140 | FBXW4 | ZNF234 | FCGR3B |  |  |  |  |
| ENSG00000232499 | BCR | RLIM | ELANE |  |  |  |  |
| DECR2 | RBCK1 | CXCL16 | FAM95C |  |  |  |  |
| ZNF570 | EPM2A-DT | SLC25A34 | CALHM3 |  |  |  |  |
| GLT1D1 | MXI1 | OCLN | CHRM5 |  |  |  |  |
| ENSG00000276517 | AIFM1 | METTL6 | FERD3L |  |  |  |  |
| THAP11 | GPKOW | SCN3B | DHRS2 |  |  |  |  |
| TREM2 | MEIS3P1 | POLR2J3 | NFE2 |  |  |  |  |
| TSKU | SMIM5 | SH3BP5 | C1orf94 |  |  |  |  |
| SNCA | TFEB | ARL17A | JAML |  |  |  |  |
| SLC16A2 | ORC6 | ARFIP1 | SNORD128 |  |  |  |  |
| URAHP | SF3A2 | C17orf103 | LPO |  |  |  |  |
| ACO1 | SEM1 | KLHL24 | MIR194-2HG |  |  |  |  |
| ODAD4 | CHST7 | ODF3B | LINC00504 |  |  |  |  |
| SERPINF1 | NR1H2 | CPSF7 | BAAT |  |  |  |  |
| RPL21P65 | BAG1 | RPL6 | LOC100507388 |  |  |  |  |
| RPL36AP33 | MPND | GAPDH | LOC440982 |  |  |  |  |
| MOB3B | GPR146 | MORF4L2 | RASGRP3 |  |  |  |  |
| ENSG00000272941 | ABL1 | GAA | LINC00609 |  |  |  |  |
| RN7SKP16 | MLF2 | NFKBID | LOC101927960 |  |  |  |  |
| RNU6-130P | HSPB1 | PTBP1 | LINC01331 |  |  |  |  |
| RDH11 | STRN4 | PRKACA | LOC101927168 |  |  |  |  |
| SULT1B1 | PCGF1 | TIMP2 | NMRK2 |  |  |  |  |
| F11 | APRT | USMG5 | HOXD1 |  |  |  |  |
| GCH1 | TCL1A | GNB4 | LOC100130698 |  |  |  |  |
| ENSG00000273243 | ATP6V0C | HIAT1 | CLEC18B |  |  |  |  |
| ENSG00000232034 | GDE1 | PER2 | CEL |  |  |  |  |
| PLIN2 | S100A11 | LOC100129250 | ANXA2P3 |  |  |  |  |
| ACADSB | TPRG1L | PHOSPHO1 | LOC101929284 |  |  |  |  |
| SLC22A18 | AK1 | KDM3A | DIO3OS |  |  |  |  |
| IBA57 | LOC441666 | RPS16 | LOC101929341 |  |  |  |  |
| RN7SKP283 | BANF1 | MAB21L3 | FAM151A |  |  |  |  |
| ANG | GLUL | MGC2752 | FAM66E |  |  |  |  |
| CAPN5 | UBE2M | PRKDC | NXPH2 |  |  |  |  |
| CEP112 | CPQ | SYAP1 | LINC01494 |  |  |  |  |
| DNAL1 | PARN | RLF | LOC100130370 |  |  |  |  |
| GK-IT1 | GSEC | AP2A1 | CBLN4 |  |  |  |  |
| SFXN1 | LTBP2 | CANX | LINC00398 |  |  |  |  |
| VAMP1 | GLB1 | SNRPB | GIPR |  |  |  |  |
| NDUFB8 | KIR2DS2 | SLC26A8 | C21orf91-OT1 |  |  |  |  |
| EGR1 | SRM | ETF1 | NMUR2 |  |  |  |  |
| RPS9 | RAB5C | LILRA3 | BCRP3 |  |  |  |  |
| GRB10 | NCAPD2 | HIVEP1 | TSPAN18 |  |  |  |  |
| PDK1 | TMEM86B | RANBP2 | RORC |  |  |  |  |
| ENSG00000267587 | CDK2AP2 | NDUFB3 | CD300LG |  |  |  |  |
| PBLD | PDIA5 | IFIT2 | HIST1H1T |  |  |  |  |
| IFT57 | ST6GALNAC4 | PNPLA8 | MUC5AC |  |  |  |  |
| ITGAE | H2BC12 | CDK5RAP2 | SLC17A6 |  |  |  |  |
| CTH | F13A1 | ENO1 | KRT8P41 |  |  |  |  |
| ENSG00000269044 | SGO2 | RAB22A | CD38 |  |  |  |  |
| ENSG00000271984 | GIMAP7 | NBN | MIR4513 |  |  |  |  |
| PMM1 | CTDSP2 | CPPED1 |  |  |  |  |  |
| POM121L9P | DNAJB6 | EOGT |  |  |  |  |  |
| RPIA | UBXN6 | DHRSX |  |  |  |  |  |
| SPINT2 | MARCKSL1 | UBE2L6 |  |  |  |  |  |
| SS18L1 | RNF182 | CCDC71L |  |  |  |  |  |
| ANPEP | NUDT16L2P | NAPA |  |  |  |  |  |
| RBP5 | RNF123 | HMGN1 |  |  |  |  |  |
| MSR1 | ACRBP | DMXL2 |  |  |  |  |  |
| PDLIM1P4 | C6orf136 | MCOLN1 |  |  |  |  |  |
| CRTAC1 | NFKBIE | RPS29 |  |  |  |  |  |
| MARS2 | CDCA4 | PRPF4 |  |  |  |  |  |
| PARP16 | ATP6 | IFIT3 |  |  |  |  |  |
| ENSG00000267385 | LINC00937 | ARHGDIA |  |  |  |  |  |
| GNG11 | SLC6A8 | ZNF440 |  |  |  |  |  |
| DDB2 | SLC25A1 | ZNF554 |  |  |  |  |  |
| DAPK1 | PPM1G | MAPKAPK3 |  |  |  |  |  |
| HHEX | KTN1 | EMD |  |  |  |  |  |
| PTPRQ | GRAP2 | SERTAD1 |  |  |  |  |  |
| HYDIN | ETHE1 | KCTD20 |  |  |  |  |  |
| CLTCL1 | PGRMC1 | MSMO1 |  |  |  |  |  |
| MORF4L1P1 | PIP4P1 | PECAM1 |  |  |  |  |  |
| ENO1P3 | PIM1 | MGAT4A |  |  |  |  |  |
| MT1G | GPSM3 | SLMO2 |  |  |  |  |  |
| KMO | CTSD | LOC284454 |  |  |  |  |  |
| PHAX | TLR4 | AP2B1 |  |  |  |  |  |
| PDE8A | ARRB2 | FAM210B |  |  |  |  |  |
| HADHB | GYS1 | ATXN2L |  |  |  |  |  |
| TMEM177 | ADA2 | WARS |  |  |  |  |  |
| PON2 | AHSA2P | GGA1 |  |  |  |  |  |
| SERINC2 | PCK2 | ZNF37A |  |  |  |  |  |
| GADD45GIP1 | WDR54 | EMB |  |  |  |  |  |
| ENSG00000267430 | USP39 | LOC100506710 |  |  |  |  |  |
| NIT2 | KIFBP | DNTTIP2 |  |  |  |  |  |
| SLC26A1 | ALAS1 | GZF1 |  |  |  |  |  |
| ID3 | ISG20 | OXSR1 |  |  |  |  |  |
| CCL20 | TCN1 | TPI1 |  |  |  |  |  |
| CRIP2 | AP1M1 | NKIRAS2 |  |  |  |  |  |
| MAB21L4 | MYL4 | DDX21 |  |  |  |  |  |
| RPS19 | TUBB6 | RBM41 |  |  |  |  |  |
| VAMP8 | NPRL3 | GTPBP1 |  |  |  |  |  |
| ENSG00000279250 | ABCG1 | CPD |  |  |  |  |  |
| MPHOSPH9 | ASGR1 | IRAK3 |  |  |  |  |  |
| DNAJC25 | RXRA | GPX4 |  |  |  |  |  |
| LGALSL | ADGRE1 | SIGLEC10 |  |  |  |  |  |
| CLDN12 | CORO1B | ZEB1 |  |  |  |  |  |
| TYW5 | BAZ2B-AS1 | HEXB |  |  |  |  |  |
| HDGFL2 | LOC100310756 | USP14 |  |  |  |  |  |
| ZBTB25 | DPM1 | GPR65 |  |  |  |  |  |
| TRIM27 | PRR5 | ZNF516 |  |  |  |  |  |
| ZNF219 | ABCF1 | EML4 |  |  |  |  |  |
| GPD1L | MPP1 | GBP6 |  |  |  |  |  |
| ENSG00000241489 | ACKR1 | HIF1A |  |  |  |  |  |
| LACTB2 | PLPPR2 | AGPAT6 |  |  |  |  |  |
| MRO | SAC3D1 | FAM174A |  |  |  |  |  |
| CCDC148 | USP7-AS1 | RNF169 |  |  |  |  |  |
| H4C3 | CHPT1 | SESN2 |  |  |  |  |  |
| SOX18 | GAS2L1 | S100A4 |  |  |  |  |  |
| PHKA2 | RBIS | SIN3A |  |  |  |  |  |
| CCN4 | FABP5 | RABGEF1 |  |  |  |  |  |
| ENSG00000281091 | FAXDC2 | RGS2 |  |  |  |  |  |
| CLSTN3 | KIR3DL1 | QSOX1 |  |  |  |  |  |
| ENKD1 | H2BC9 | ATG2A |  |  |  |  |  |
| UBTF | LOC105379250 | HSP90AB1 |  |  |  |  |  |
| ENSG00000279236 | MTX1 | DNAJC14 |  |  |  |  |  |
| PLCD3 | LILRA1 | RHEB |  |  |  |  |  |
| PLOD2 | JAZF1 | KCNE3 |  |  |  |  |  |
| ZNF207 | RNPEPL1 | RPS8 |  |  |  |  |  |
| CPM | R3HDM4 | APH1B |  |  |  |  |  |
| ZNF813 | CBWD2 | STOM |  |  |  |  |  |
| BTG2 | LINC01857 | MOAP1 |  |  |  |  |  |
| CAPS | BRAT1 | CD97 |  |  |  |  |  |
| ENSG00000279838 | DBNL | MOCS3 |  |  |  |  |  |
| MTND2P28 | ATP5ME | SEC16A |  |  |  |  |  |
| MYOM3 | SH2D2A | LINC00476 |  |  |  |  |  |
| ENSG00000270426 | MYOM2 | PLK3 |  |  |  |  |  |
| ARHGAP18 | MTLN | SLC20A1 |  |  |  |  |  |
| CYP4F12 | BTBD11 | SULF2 |  |  |  |  |  |
| ENSG00000266709 | OSGEP | NXPE3 |  |  |  |  |  |
| SYVN1 | CPNE2 | CD83 |  |  |  |  |  |
| ENSG00000256981 | THTPA | TRAF3IP3 |  |  |  |  |  |
| VASH1 | FKBP1B | CLEC2D |  |  |  |  |  |
| PLLP | CHRAC1 | RASA4 |  |  |  |  |  |
| HNRNPA0 | FLJ32255 | POLB |  |  |  |  |  |
| FDPSP3 | TGM2 | MAP3K8 |  |  |  |  |  |
| NHSL1-AS1 | CA2 | PURB |  |  |  |  |  |
| KHK | STMP1 | GADD45B |  |  |  |  |  |
| RPS3AP26 | TRAV13-1 | PLEKHB2 |  |  |  |  |  |
| ENSG00000278266 | TAF15 |  |  |  |  |  |  |
| TUBB4B | TMEM250 |  |  |  |  |  |  |
| PIAS2 | ZNF479 |  |  |  |  |  |  |
| TEDC1 | GALNT6 |  |  |  |  |  |  |
| ZNF14 | TMEM45B |  |  |  |  |  |  |
| PDAP1 | MICB |  |  |  |  |  |  |
| SART1 | FCRL4 |  |  |  |  |  |  |
| ZNF557 | DBN1 |  |  |  |  |  |  |
| ENSG00000266805 | GUCY1B1 |  |  |  |  |  |  |
| PZP | H4C8 |  |  |  |  |  |  |
| FAM174B | HPCAL1 |  |  |  |  |  |  |
| WASF1 | MEST |  |  |  |  |  |  |
| NWD1 | OAZ2 |  |  |  |  |  |  |
| B3GALNT1 | SELP |  |  |  |  |  |  |
| ENSG00000260261 | FBP1 |  |  |  |  |  |  |
| LARP4P | NCF4 |  |  |  |  |  |  |
| ENSG00000224972 | TRAV21 |  |  |  |  |  |  |
| YIF1A | SAP130 |  |  |  |  |  |  |
| SPIN3 | HYMAI |  |  |  |  |  |  |
| ENSG00000276850 | ASPHD2 |  |  |  |  |  |  |
| RRAS | KEAP1 |  |  |  |  |  |  |
| RPS25 | PDZK1IP1 |  |  |  |  |  |  |
| RPS21 | NEURL4 |  |  |  |  |  |  |
| FAM20A | CDA |  |  |  |  |  |  |
| ALDOC | LSP1 |  |  |  |  |  |  |
| USP32 | DNAJB2 |  |  |  |  |  |  |
| HGF | UBALD2 |  |  |  |  |  |  |
| SIAE | SFRP2 |  |  |  |  |  |  |
| LYG1 | HBM |  |  |  |  |  |  |
| LIFR-AS1 | EIF2AK1 |  |  |  |  |  |  |
| METTL21AP1 | TREX1 |  |  |  |  |  |  |
| SYPL2 | ROGDI |  |  |  |  |  |  |
| SEPTIN4-AS1 | MMD |  |  |  |  |  |  |
| MATK | GTSF1 |  |  |  |  |  |  |
| MMRN2 | PDCD6P1 |  |  |  |  |  |  |
| TSPYL1 | SLC20A2 |  |  |  |  |  |  |
| GLRB | ELOF1 |  |  |  |  |  |  |
| TWSG1 | GUCD1 |  |  |  |  |  |  |
| RNF152 | INAFM1 |  |  |  |  |  |  |
| RFX2 | PUDP |  |  |  |  |  |  |
| ENSG00000279814 | RUVBL1 |  |  |  |  |  |  |
| ENSG00000225527 | MSRB1 |  |  |  |  |  |  |
| RNU4ATAC11P | STRADB |  |  |  |  |  |  |
| SRPRA | ANKRD9 |  |  |  |  |  |  |
| GRK5 | LOC105379173 |  |  |  |  |  |  |
| SLC35B1 | PRPF4 |  |  |  |  |  |  |
| ENSG00000279319 | POLR1D |  |  |  |  |  |  |
| AOC2 | ZBTB8OS |  |  |  |  |  |  |
| PPP2R1B | SSB |  |  |  |  |  |  |
| NR1D1 | RHOG |  |  |  |  |  |  |
| YJU2 | NOMO3 |  |  |  |  |  |  |
| ENSG00000285725 | PSMC6 |  |  |  |  |  |  |
| XIRP1 | DCXR |  |  |  |  |  |  |
| DNAAF1 | GPAT2 |  |  |  |  |  |  |
| CEBPB-AS1 | ALDOAP2 |  |  |  |  |  |  |
| DLEC1 | WDR45 |  |  |  |  |  |  |
| ALDH6A1 | PGM1 |  |  |  |  |  |  |
| CDCA7L | CR1L |  |  |  |  |  |  |
| RMDN3 | RBM23 |  |  |  |  |  |  |
| SPTA1 | MAGOH |  |  |  |  |  |  |
| EBLN2 | FGF7P3 |  |  |  |  |  |  |
| PPBP | C12orf43 |  |  |  |  |  |  |
| FAM174A | YIPF3 |  |  |  |  |  |  |
| SHMT2 | STAT6 |  |  |  |  |  |  |
| TOR3A | RASSF10 |  |  |  |  |  |  |
| WIPI1 | PSENEN |  |  |  |  |  |  |
| USHBP1 | U2AF1L4 |  |  |  |  |  |  |
| HAAO | C2orf42 |  |  |  |  |  |  |
| PRPF6 | SLC15A3 |  |  |  |  |  |  |
| PLXNA2 | CRYL1 |  |  |  |  |  |  |
| ENSG00000224967 | HOXC6 |  |  |  |  |  |  |
| PGAM1P8 | KIR3DL3 |  |  |  |  |  |  |
| SNX22 | MST1L |  |  |  |  |  |  |
| PLEKHF1 | UBAP1 |  |  |  |  |  |  |
| RANGRF | STX5 |  |  |  |  |  |  |
| PTS | PLD3 |  |  |  |  |  |  |
| OTULINL | PNP |  |  |  |  |  |  |
| TTC30B | ATIC |  |  |  |  |  |  |
| EEF1A1P19 | ASCC2 |  |  |  |  |  |  |
| ADAT2 | SHKBP1 |  |  |  |  |  |  |
| ICAM5 | ELMO1 |  |  |  |  |  |  |
| C14orf132 | MT1X |  |  |  |  |  |  |
| SLC23A2 | HECW2-AS1 |  |  |  |  |  |  |
| ZCCHC2 | ZNF542P |  |  |  |  |  |  |
| STARD5 | ZC3H15 |  |  |  |  |  |  |
| VKORC1 | MED25 |  |  |  |  |  |  |
| SCYL2P1 | PSMB9 |  |  |  |  |  |  |
| GPT2 | LRFN1 |  |  |  |  |  |  |
| HMGCLL1 | AP2M1 |  |  |  |  |  |  |
| TUBE1 | KBTBD3 |  |  |  |  |  |  |
| SMARCA5 | H2BC10 |  |  |  |  |  |  |
| DNAJB13 | ZNF526 |  |  |  |  |  |  |
| LGALS8 | MAP7D1 |  |  |  |  |  |  |
| SLC35E1P1 | LOC105371967 |  |  |  |  |  |  |
| CEBPZOS | ITGA2B |  |  |  |  |  |  |
| HMGCS1 | H1-2 |  |  |  |  |  |  |
| REEP6 | UQCRB |  |  |  |  |  |  |
| RAPGEF3 | CXCL8 |  |  |  |  |  |  |
| H1-10-AS1 | ZNF628 |  |  |  |  |  |  |
| PI15 | RECQL |  |  |  |  |  |  |
| KIF3A | KLHDC8B |  |  |  |  |  |  |
| KLF4 | EVA1C |  |  |  |  |  |  |
| ENSG00000279159 | PRKAR2B |  |  |  |  |  |  |
| PTPN3 | RRAGD |  |  |  |  |  |  |
| AKAP13 | IPO4 |  |  |  |  |  |  |
| HLA-DPB1 | ROPN1L |  |  |  |  |  |  |
| STOX1 | SIGLEC10 |  |  |  |  |  |  |
| MRPS23 | TIGD3 |  |  |  |  |  |  |
| DHTKD1 | RABAC1 |  |  |  |  |  |  |
| TACC1 | P2RY12 |  |  |  |  |  |  |
| ENSG00000274677 | SRRD |  |  |  |  |  |  |
| MAGEH1 | HBQ1 |  |  |  |  |  |  |
| ACSS2 | SIRPB2 |  |  |  |  |  |  |
| CATIP | CLU |  |  |  |  |  |  |
| FRMD3 | PVRIG |  |  |  |  |  |  |
| SLC7A2 | HTT |  |  |  |  |  |  |
| NOL7 | UBL7 |  |  |  |  |  |  |
| ZSCAN18 | TESC |  |  |  |  |  |  |
| RPL13AP5 | TMEM268 |  |  |  |  |  |  |
| CCDC9 | MCOLN1 |  |  |  |  |  |  |
| WHAMMP3 | KCNE3 |  |  |  |  |  |  |
| ACAT2 | VPS51 |  |  |  |  |  |  |
| CCDC65 | MFSD5 |  |  |  |  |  |  |
| MFAP1 | GBP4 |  |  |  |  |  |  |
| PHLDA3 | CCNJL |  |  |  |  |  |  |
| PCCA | LOC101927166 |  |  |  |  |  |  |
| AQP4 | SLC43A1 |  |  |  |  |  |  |
| SEC31A | PEX6 |  |  |  |  |  |  |
| NCL | FXYD6 |  |  |  |  |  |  |
| SPRY2 | RAD23A |  |  |  |  |  |  |
| MSMO1 | TRAPPC10 |  |  |  |  |  |  |
| THBD | CHI3L1 |  |  |  |  |  |  |
| MRPS10 | HAGH |  |  |  |  |  |  |
| LINC02348 | ANK1 |  |  |  |  |  |  |
| SOX17 | FLOT2 |  |  |  |  |  |  |
| PXMP4 | LBH |  |  |  |  |  |  |
| SCARNA6 | VAMP2 |  |  |  |  |  |  |
| SLC16A7 | RPF2 |  |  |  |  |  |  |
| CLUAP1 | PUSL1 |  |  |  |  |  |  |
| TAS2R3 | SPDYE1 |  |  |  |  |  |  |
| NINJ1 | AP2B1 |  |  |  |  |  |  |
| MPC1 | TRAPPC5 |  |  |  |  |  |  |
| ARMCX1 | SNRPE |  |  |  |  |  |  |
| ENSG00000244313 | TRIM21 |  |  |  |  |  |  |
| TBCC | PPP2R5B |  |  |  |  |  |  |
| OBSL1 | NFE2 |  |  |  |  |  |  |
| PDCL3 | ATP6V1B2 |  |  |  |  |  |  |
| TGDS | DPP4 |  |  |  |  |  |  |
| ENSG00000276649 | SIAH2 |  |  |  |  |  |  |
| ARRB1 | GUK1 |  |  |  |  |  |  |
| FANK1 | FRMD3 |  |  |  |  |  |  |
| MFSD14A | ZNF581 |  |  |  |  |  |  |
| MT-CYB | TMOD1 |  |  |  |  |  |  |
| CLIC5 | ZNF808 |  |  |  |  |  |  |
| FZD1 | ABHD2 |  |  |  |  |  |  |
| ALPK1 | HDGF |  |  |  |  |  |  |
| ZDHHC19 | LINC00339 |  |  |  |  |  |  |
| ENSG00000261051 | CTSA |  |  |  |  |  |  |
| BICD2 | HAUS4 |  |  |  |  |  |  |
| SYT7 | MT1F |  |  |  |  |  |  |
| NCAPH2 | TUBB2A |  |  |  |  |  |  |
| SLC2A10 | DSPP |  |  |  |  |  |  |
| IL1R2 | CD7 |  |  |  |  |  |  |
| SLC38A7 | RANBP10 |  |  |  |  |  |  |
| TRAPPC1 | TRIM58 |  |  |  |  |  |  |
| SRP14 | GATA1 |  |  |  |  |  |  |
| HNRNPCP6 | PNPLA6 |  |  |  |  |  |  |
| FKBP8 | ENO2 |  |  |  |  |  |  |
| AOC4P | PGLYRP1 |  |  |  |  |  |  |
| GRN | ADORA3 |  |  |  |  |  |  |
| ATRN | HLA-DMA |  |  |  |  |  |  |
| VAT1 | TMTC1 |  |  |  |  |  |  |
| CCDC112 | CLEC1B |  |  |  |  |  |  |
| AMOTL2 | HSF5 |  |  |  |  |  |  |
| ENSG00000286314 | LRRC25 |  |  |  |  |  |  |
| ENSG00000260865 | YPEL4 |  |  |  |  |  |  |
| TLE2 | SH3BGRL2 |  |  |  |  |  |  |
| EPS8L1 | POR |  |  |  |  |  |  |
| SPRYD3 | CHURC1 |  |  |  |  |  |  |
| SMAD9 | OCEL1 |  |  |  |  |  |  |
| C7orf50 | AMIGO1 |  |  |  |  |  |  |
| HSPA12B | NDUFS5 |  |  |  |  |  |  |
| FMO3 | SPATC1L |  |  |  |  |  |  |
| MEG3 | GRAMD1C |  |  |  |  |  |  |
| KLF6 | TGM3 |  |  |  |  |  |  |
| ST20 | ACCS |  |  |  |  |  |  |
| VPS37B | BCL2L1 |  |  |  |  |  |  |
| ENSG00000279544 | SLC31A2 |  |  |  |  |  |  |
| STAB2 | FAHD1 |  |  |  |  |  |  |
| SUSD2 | RAD21-AS1 |  |  |  |  |  |  |
| IDH1 | CAVIN2 |  |  |  |  |  |  |
| SLPI | GPX1 |  |  |  |  |  |  |
| MTND4P12 | S100P |  |  |  |  |  |  |
| ADGRV1 | PRDX5 |  |  |  |  |  |  |
| ZNF700 | NAPA |  |  |  |  |  |  |
| CEP89 | TRANK1 |  |  |  |  |  |  |
| FREM2 | WBP2 |  |  |  |  |  |  |
| CBLC | HMBS |  |  |  |  |  |  |
| GAPDHP43 | NOP2 |  |  |  |  |  |  |
| SNORD62B | PRKCD |  |  |  |  |  |  |
| ENSG00000277999 | H2BC6 |  |  |  |  |  |  |
| GLYCTK | CD3E |  |  |  |  |  |  |
| PIKFYVE | GMPPA |  |  |  |  |  |  |
| SECISBP2L | MAF1 |  |  |  |  |  |  |
| PRX | PGM5P2 |  |  |  |  |  |  |
| LARP1B | PSMF1 |  |  |  |  |  |  |
| SPRYD4 | TREML1 |  |  |  |  |  |  |
| GRAMD2B | DPEP3 |  |  |  |  |  |  |
| RPRD1B | CCR1 |  |  |  |  |  |  |
| PLBD1 | DANCR |  |  |  |  |  |  |
| PCDHB9 | TNS1 |  |  |  |  |  |  |
| ENSG00000261560 | H2AC6 |  |  |  |  |  |  |
| DNAJC12 | MFSD13A |  |  |  |  |  |  |
| C4B | KRT1 |  |  |  |  |  |  |
| ZNF653 | PIGU |  |  |  |  |  |  |
| COG3 | TBCA |  |  |  |  |  |  |
| TMEM37 | PLEKHF1 |  |  |  |  |  |  |
| ITGA8 | RAB5B |  |  |  |  |  |  |
| XPNPEP1 | ANKRD55 |  |  |  |  |  |  |
| ENSG00000234405 | IFRD2 |  |  |  |  |  |  |
| ENSG00000230333 | ITPRIPL1 |  |  |  |  |  |  |
| ZC3H4 | GLT1D1 |  |  |  |  |  |  |
| PCDHB12 | CXCR1 |  |  |  |  |  |  |
| TTPAL | DCAF12 |  |  |  |  |  |  |
| SLC46A3 | POLD2 |  |  |  |  |  |  |
| ENSG00000261596 | CD38 |  |  |  |  |  |  |
| ENSG00000225213 | ZER1 |  |  |  |  |  |  |
| GPC6 | SLC27A3 |  |  |  |  |  |  |
| BACH1-IT3 | PLAUR |  |  |  |  |  |  |
| ENSG00000240291 | SAMD12 |  |  |  |  |  |  |
| WWC2 | MICAL2 |  |  |  |  |  |  |
| TSR3 | ITLN1 |  |  |  |  |  |  |
| HBB | MYL6B |  |  |  |  |  |  |
| SNAI2 | BBOF1 |  |  |  |  |  |  |
| SSTR2 | KIR2DS5 |  |  |  |  |  |  |
| NUDT7 | ASPRV1 |  |  |  |  |  |  |
| PFDN4 | IL15RA |  |  |  |  |  |  |
| SYNGR1 | MBNL3 |  |  |  |  |  |  |
| FBXO17 | CSF1R |  |  |  |  |  |  |
| PELI3 | CD274 |  |  |  |  |  |  |
| COLCA1 | EIF2D |  |  |  |  |  |  |
| BEX4 | CSRNP1 |  |  |  |  |  |  |
| NR0B2 | DNTTIP2 |  |  |  |  |  |  |
| TES | KLF1 |  |  |  |  |  |  |
| SNRPD1 | MYH9 |  |  |  |  |  |  |
| SLC35C1 | IL1B |  |  |  |  |  |  |
| ENSG00000258578 | CWC15 |  |  |  |  |  |  |
| ENSG00000279786 | CFP |  |  |  |  |  |  |
| SUPV3L1 | ITPRIP |  |  |  |  |  |  |
| ZNF250 | GPA33 |  |  |  |  |  |  |
| CD1D | SNORA21 |  |  |  |  |  |  |
| ZBTB4 | TENT5C |  |  |  |  |  |  |
| ADGRA3 | GSTM2 |  |  |  |  |  |  |
| SLED1 | EPB42 |  |  |  |  |  |  |
| FAM53B | DHRS3 |  |  |  |  |  |  |
| SPRED1 | LOC389831 |  |  |  |  |  |  |
| IGSF9B | HPSE |  |  |  |  |  |  |
| HMG20B | DHRS13 |  |  |  |  |  |  |
| SPATA41 | NUCB1 |  |  |  |  |  |  |
| WTIP | LOC105377782 |  |  |  |  |  |  |
| SETP6 | DYSF |  |  |  |  |  |  |
| KYAT1 | PFDN4 |  |  |  |  |  |  |
| HNRNPA1 | PDZD11 |  |  |  |  |  |  |
| KLF2 | NDUFA1 |  |  |  |  |  |  |
| PNPO | FAM214B |  |  |  |  |  |  |
| OLFML2A | HMGN3 |  |  |  |  |  |  |
| GPLD1 | FIS1 |  |  |  |  |  |  |
| RN7SKP90 | FCMR |  |  |  |  |  |  |
| CHDH | GTF2H2B |  |  |  |  |  |  |
| TUT7 | ZNF600 |  |  |  |  |  |  |
| KDELR2 | HSPE1 |  |  |  |  |  |  |
| DMRTA1 | ZNF57 |  |  |  |  |  |  |
| MRPL37 | ABHD14B |  |  |  |  |  |  |
| AVPR1A | BPI |  |  |  |  |  |  |
| SLC30A7 | OXTR |  |  |  |  |  |  |
| JUN | HBG2 |  |  |  |  |  |  |
| LRATD1 | EEF1B2 |  |  |  |  |  |  |
| SLC38A9 | CTNNAL1 |  |  |  |  |  |  |
| TSPOAP1 | YBX3 |  |  |  |  |  |  |
| TTC38 | MED22 |  |  |  |  |  |  |
| ENSG00000272140 | DGAT2 |  |  |  |  |  |  |
| KIAA1143 | CLEC4D |  |  |  |  |  |  |
| ABCA5 | LOC101928893 |  |  |  |  |  |  |
| VWA3A | GMPR |  |  |  |  |  |  |
| DLC1 | PCSK1N |  |  |  |  |  |  |
| EIPR1-IT1 | SHCBP1 |  |  |  |  |  |  |
| ENSG00000231181 | BABAM1 |  |  |  |  |  |  |
| RPL21P5 | HLX |  |  |  |  |  |  |
| CLU | BLVRB |  |  |  |  |  |  |
| ENSG00000248794 | STX11 |  |  |  |  |  |  |
| PWWP2B | LRG1 |  |  |  |  |  |  |
| ABCA1 | OSBP2 |  |  |  |  |  |  |
| PPT1 | TATDN1 |  |  |  |  |  |  |
| FGF10 | LSM8 |  |  |  |  |  |  |
| NIPSNAP1 | OPTN |  |  |  |  |  |  |
| SPAG5-AS1 | ABHD15 |  |  |  |  |  |  |
| ENSG00000260064 | PCTP |  |  |  |  |  |  |
| PTMS | SLC4A1 |  |  |  |  |  |  |
| RNF165 | SELENOM |  |  |  |  |  |  |
| LINC00907 | PHOSPHO1 |  |  |  |  |  |  |
| STEEP1 | H2BC5 |  |  |  |  |  |  |
| GAS6 | PDCD4-AS1 |  |  |  |  |  |  |
| ENSG00000280321 | NFU1 |  |  |  |  |  |  |
| GIMAP7 | SMIM30 |  |  |  |  |  |  |
| RPL23AP7 | FOXO4 |  |  |  |  |  |  |
| RPL32P32 | AK6 |  |  |  |  |  |  |
| NIM1K | ADGRE3 |  |  |  |  |  |  |
| MRRF | SLPI |  |  |  |  |  |  |
| HNRNPA1P10 | WLS |  |  |  |  |  |  |
| TYRO3 | CETN3 |  |  |  |  |  |  |
| UBL5 | NME4 |  |  |  |  |  |  |
| RNU4ATAC18P | BATF2 |  |  |  |  |  |  |
| FGD5 | C2orf74 |  |  |  |  |  |  |
| HLA-DRA | LINC01527 |  |  |  |  |  |  |
| PUS3 | ODC1 |  |  |  |  |  |  |
| SP6 | PIP4K2A |  |  |  |  |  |  |
| TRMT112 | HAT1 |  |  |  |  |  |  |
| BMP6 | UBE2L6 |  |  |  |  |  |  |
| BCO2 | RGL4 |  |  |  |  |  |  |
| ZNF821 | KLRG1 |  |  |  |  |  |  |
| DHODH | PCED1B |  |  |  |  |  |  |
| KIAA1328 | TMEM91 |  |  |  |  |  |  |
| YIPF1 | FAM118A |  |  |  |  |  |  |
| CA1 | PTAFR |  |  |  |  |  |  |
| C12orf57 | XK |  |  |  |  |  |  |
| TRIM58 | LINC02481 |  |  |  |  |  |  |
| OLAH | E2F2 |  |  |  |  |  |  |
| MAPK8IP2 | B3GNT8 |  |  |  |  |  |  |
| ENSG00000279196 | PRXL2B |  |  |  |  |  |  |
| WT1 | PLEK2 |  |  |  |  |  |  |
| CBX7 | RELB |  |  |  |  |  |  |
| ZBTB22 | EIF1AY |  |  |  |  |  |  |
| DENND6B | MAP3K7CL |  |  |  |  |  |  |
| PDZD2 | NFKB1 |  |  |  |  |  |  |
| ENSG00000259682 | CLEC12B |  |  |  |  |  |  |
| ANKRD49P2 | FHIT |  |  |  |  |  |  |
| ARPC5 | ADGRG3 |  |  |  |  |  |  |
| ACAA2 | AHSP |  |  |  |  |  |  |
| ENSG00000232545 | FHL2 |  |  |  |  |  |  |
| LBH | FAM104A |  |  |  |  |  |  |
| ENSG00000227615 | CXCL16 |  |  |  |  |  |  |
| ZNF574 | RHBDF2 |  |  |  |  |  |  |
| CCDC71 | TNFAIP6 |  |  |  |  |  |  |
| ZNF784 | LHFPL2 |  |  |  |  |  |  |
| B9D2 | PSMA4 |  |  |  |  |  |  |
| TMEM11-DT | SCN1B |  |  |  |  |  |  |
| PNMA1 | PLVAP |  |  |  |  |  |  |
| ALDH4A1 | DPM2 |  |  |  |  |  |  |
| LINC00607 | ERV3-1 |  |  |  |  |  |  |
| ZNF563 | TSPAN5 |  |  |  |  |  |  |
| SNRPD3 | SNRPD1 |  |  |  |  |  |  |
| ALYREF | OTUD6B-AS1 |  |  |  |  |  |  |
| IGHA2 | TPRKB |  |  |  |  |  |  |
| ZNF431 | GYPB |  |  |  |  |  |  |
| CENPB | MAD2L1BP |  |  |  |  |  |  |
| PRKCH-AS1 | LOC105375492 |  |  |  |  |  |  |
| SOD1-DT | SARNP |  |  |  |  |  |  |
| TRIR | TNFSF13 |  |  |  |  |  |  |
| PLCXD2 | RWDD3 |  |  |  |  |  |  |
| ENSG00000259453 | ARMCX2 |  |  |  |  |  |  |
| COX10 | SELENBP1 |  |  |  |  |  |  |
| AR | RPL26L1 |  |  |  |  |  |  |
| NPTN-IT1 | LRRC4 |  |  |  |  |  |  |
| PPIF | CA4 |  |  |  |  |  |  |
| ENSG00000261578 | RPL22L1 |  |  |  |  |  |  |
| C8orf76 | TMA7 |  |  |  |  |  |  |
| SH3BP5 | USP16 |  |  |  |  |  |  |
| NUDT14 | CASC3 |  |  |  |  |  |  |
| CYB561 | CMC1 |  |  |  |  |  |  |
| MYL3 | UQCRH |  |  |  |  |  |  |
| HMGN2P5 | P2RX7 |  |  |  |  |  |  |
| DAPK2 | CTSB |  |  |  |  |  |  |
| H2AC21 | CEACAM1 |  |  |  |  |  |  |
| CD69 | GBP5 |  |  |  |  |  |  |
| LPGAT1 | SECTM1 |  |  |  |  |  |  |
| VMP1 | ABCC13 |  |  |  |  |  |  |
| MPRIP | P2RY14 |  |  |  |  |  |  |
| ABCB6 | MARCHF8 |  |  |  |  |  |  |
| ADAM20P1 | NINJ2 |  |  |  |  |  |  |
| RNU6-1045P | MRC2 |  |  |  |  |  |  |
| BANF1 | WARS1 |  |  |  |  |  |  |
| CEBPD | OASL |  |  |  |  |  |  |
| ERBB2 | NAPSB |  |  |  |  |  |  |
| GLIPR2 | FBXO6 |  |  |  |  |  |  |
| CLMN | FAM106A |  |  |  |  |  |  |
| RPS14 | RAB2B |  |  |  |  |  |  |
| CMYA5 | KIR3DL2 |  |  |  |  |  |  |
| RAB3B | SLC7A5 |  |  |  |  |  |  |
| NCR3LG1 | RBM38 |  |  |  |  |  |  |
| PITRM1 | CEACAM21 |  |  |  |  |  |  |
| GGH | LCN2 |  |  |  |  |  |  |
| VDAC1 | FAM210B |  |  |  |  |  |  |
| GSDMC | COX16 |  |  |  |  |  |  |
| COL21A1 | SLC22A18 |  |  |  |  |  |  |
| ENSG00000283828 | SNRPG |  |  |  |  |  |  |
| AGRN | MKRN1 |  |  |  |  |  |  |
| RILPL1 | BSG |  |  |  |  |  |  |
| IP6K3 | LTF |  |  |  |  |  |  |
| CCDC124 | COX6C |  |  |  |  |  |  |
| SHROOM1 | RWDD1 |  |  |  |  |  |  |
| HCG11 | CTSL |  |  |  |  |  |  |
| RPSAP6 | PI3 |  |  |  |  |  |  |
| DYRK1B | GLRX5 |  |  |  |  |  |  |
| ACTG1P17 | PF4 |  |  |  |  |  |  |
| ENSG00000281195 | EMC2 |  |  |  |  |  |  |
| LINC01355 | MAL |  |  |  |  |  |  |
| POLDIP2 | MX1 |  |  |  |  |  |  |
| HKDC1 | RSL24D1 |  |  |  |  |  |  |
| DNAL4 | BTNL3 |  |  |  |  |  |  |
| SS18 | LAMP3 |  |  |  |  |  |  |
| FLRT3 | EPHX2 |  |  |  |  |  |  |
| RN7SL141P | RPIA |  |  |  |  |  |  |
| TCF21 | CEACAM8 |  |  |  |  |  |  |
| CLBA1 | SDCBPP2 |  |  |  |  |  |  |
| LRPAP1 | FECH |  |  |  |  |  |  |
| UFD1 | RPS7 |  |  |  |  |  |  |
| ENSG00000253180 | TUBB1 |  |  |  |  |  |  |
| CASZ1 | LY6E |  |  |  |  |  |  |
| ENSG00000258215 | MRPS28 |  |  |  |  |  |  |
| SLAIN1 | BCL2A1 |  |  |  |  |  |  |
| ABRAXAS1 | DPPA3 |  |  |  |  |  |  |
| ZBTB46 | NDUFB3 |  |  |  |  |  |  |
| HSBP1L1 | SNCA |  |  |  |  |  |  |
| F10 | METTL18 |  |  |  |  |  |  |
| WDFY3-AS2 | BPGM |  |  |  |  |  |  |
| PKHD1 | FAM3B |  |  |  |  |  |  |
| SENP3 | OLFM4 |  |  |  |  |  |  |
| SURF2 | SLC1A5 |  |  |  |  |  |  |
| DENND5B | RPL34 |  |  |  |  |  |  |
| CPVL | KDM7A-DT |  |  |  |  |  |  |
| SMAD1 | C9orf78 |  |  |  |  |  |  |
| ZBTB8A | HAUS1 |  |  |  |  |  |  |
| ENSG00000236194 | HEMGN |  |  |  |  |  |  |
| ADCY1 | KANSL1-AS1 |  |  |  |  |  |  |
| H1-3 | CHMP5 |  |  |  |  |  |  |
| FSTL4 | EPSTI1 |  |  |  |  |  |  |
| SPATA17 | SLIRP |  |  |  |  |  |  |
| RPS3AP38 | FCGR1B |  |  |  |  |  |  |
| PPP1R3E | IFI35 |  |  |  |  |  |  |
| HSD3B7 | RSAD2 |  |  |  |  |  |  |
| PLIN5 | FRG1JP |  |  |  |  |  |  |
| SPAG5 | IFIT3 |  |  |  |  |  |  |
| MMADHCP2 | CAMP |  |  |  |  |  |  |
| HSD17B7P2 | MRPL1 |  |  |  |  |  |  |
| SCML1 |  |  |  |  |  |  |  |
| MFSD9 |  |  |  |  |  |  |  |
| RSPH4A |  |  |  |  |  |  |  |
| SOX13 |  |  |  |  |  |  |  |
| GPR137B |  |  |  |  |  |  |  |
| GTF2F1 |  |  |  |  |  |  |  |
| MT-ND1 |  |  |  |  |  |  |  |
| DSTNP2 |  |  |  |  |  |  |  |
| BORCS6 |  |  |  |  |  |  |  |
| ENSG00000286994 |  |  |  |  |  |  |  |
| LIMD1 |  |  |  |  |  |  |  |
| UHRF1BP1L |  |  |  |  |  |  |  |
| CDS1 |  |  |  |  |  |  |  |
| ENSG00000256433 |  |  |  |  |  |  |  |
| SRGAP2C |  |  |  |  |  |  |  |
| LINC02018 |  |  |  |  |  |  |  |
| ENSG00000270558 |  |  |  |  |  |  |  |
| PSMD6 |  |  |  |  |  |  |  |
| POGLUT1 |  |  |  |  |  |  |  |
| LTBR |  |  |  |  |  |  |  |
| KLHL2P1 |  |  |  |  |  |  |  |
| GRPEL1 |  |  |  |  |  |  |  |
| DIPK1B |  |  |  |  |  |  |  |
| SMG1P6 |  |  |  |  |  |  |  |
| ARPIN |  |  |  |  |  |  |  |
| RPS26P18 |  |  |  |  |  |  |  |
| ENSG00000260920 |  |  |  |  |  |  |  |
| GAS8 |  |  |  |  |  |  |  |
| MTND5P11 |  |  |  |  |  |  |  |
| ENSG00000254694 |  |  |  |  |  |  |  |
| MRPS31 |  |  |  |  |  |  |  |
| DCAF13P3 |  |  |  |  |  |  |  |
| ENSG00000257169 |  |  |  |  |  |  |  |
| IL1RL2 |  |  |  |  |  |  |  |
| ENSG00000276334 |  |  |  |  |  |  |  |
| NADK2 |  |  |  |  |  |  |  |
| TM9SF1 |  |  |  |  |  |  |  |
| DHDH |  |  |  |  |  |  |  |
| ENSG00000218175 |  |  |  |  |  |  |  |
| MCTS1 |  |  |  |  |  |  |  |
| CLDN23 |  |  |  |  |  |  |  |
| ENSG00000277558 |  |  |  |  |  |  |  |
| SPIRE2 |  |  |  |  |  |  |  |
| TAMALIN |  |  |  |  |  |  |  |
| STRADB |  |  |  |  |  |  |  |
| EYS |  |  |  |  |  |  |  |
| CES2 |  |  |  |  |  |  |  |
| SNORA73B |  |  |  |  |  |  |  |
| FGF14 |  |  |  |  |  |  |  |
| ENSG00000284946 |  |  |  |  |  |  |  |
| ALG3 |  |  |  |  |  |  |  |
| ZNF611 |  |  |  |  |  |  |  |
| ATP5MF |  |  |  |  |  |  |  |
| ANKRD22 |  |  |  |  |  |  |  |
| ARFGEF3 |  |  |  |  |  |  |  |
| NLN |  |  |  |  |  |  |  |
| CADM4 |  |  |  |  |  |  |  |
| SYNC |  |  |  |  |  |  |  |
| RBM33-DT |  |  |  |  |  |  |  |
| GPR180 |  |  |  |  |  |  |  |
| IGSF22 |  |  |  |  |  |  |  |
| KYNU |  |  |  |  |  |  |  |
| NEGR1 |  |  |  |  |  |  |  |
| GNAO1 |  |  |  |  |  |  |  |
| ERI3-IT1 |  |  |  |  |  |  |  |
| ALAS1 |  |  |  |  |  |  |  |
| ERLIN1 |  |  |  |  |  |  |  |
| JDP2 |  |  |  |  |  |  |  |
| HRG-AS1 |  |  |  |  |  |  |  |
| AKR1C1 |  |  |  |  |  |  |  |
| CFAP54 |  |  |  |  |  |  |  |
| PPP1R26 |  |  |  |  |  |  |  |
| EPCAM |  |  |  |  |  |  |  |
| IQCA1 |  |  |  |  |  |  |  |
| SEC23A |  |  |  |  |  |  |  |
| IQCE |  |  |  |  |  |  |  |
| PGAM1 |  |  |  |  |  |  |  |
| NR4A1 |  |  |  |  |  |  |  |
| ENSG00000253586 |  |  |  |  |  |  |  |
| CFAP53 |  |  |  |  |  |  |  |
| TP53INP1 |  |  |  |  |  |  |  |
| RPL36AP43 |  |  |  |  |  |  |  |
| H3-3B |  |  |  |  |  |  |  |
| NNT |  |  |  |  |  |  |  |
| MCM2 |  |  |  |  |  |  |  |
| FAHD2A |  |  |  |  |  |  |  |
| RHOBTB1 |  |  |  |  |  |  |  |
| POLR1G |  |  |  |  |  |  |  |
| MYRIP |  |  |  |  |  |  |  |
| ELL2 |  |  |  |  |  |  |  |
| ENSG00000255031 |  |  |  |  |  |  |  |
| GBE1 |  |  |  |  |  |  |  |
| AIFM1 |  |  |  |  |  |  |  |
| HPS6 |  |  |  |  |  |  |  |
| DDX55P1 |  |  |  |  |  |  |  |
| DENND10 |  |  |  |  |  |  |  |
| KCTD9P4 |  |  |  |  |  |  |  |
| SLC27A2 |  |  |  |  |  |  |  |
| DDRGK1 |  |  |  |  |  |  |  |
| CBR4 |  |  |  |  |  |  |  |
| PHYKPL |  |  |  |  |  |  |  |
| ENSG00000287771 |  |  |  |  |  |  |  |
| SCD5 |  |  |  |  |  |  |  |
| ADIPOR2 |  |  |  |  |  |  |  |
| EPHB4 |  |  |  |  |  |  |  |
| PDCD5 |  |  |  |  |  |  |  |
| FRRS1 |  |  |  |  |  |  |  |
| HOXB6 |  |  |  |  |  |  |  |
| ENSG00000279133 |  |  |  |  |  |  |  |
| DUS4L |  |  |  |  |  |  |  |
| TCAIM |  |  |  |  |  |  |  |
| CEMIP |  |  |  |  |  |  |  |
| BTBD9 |  |  |  |  |  |  |  |
| ENSG00000288025 |  |  |  |  |  |  |  |
| ACBD7 |  |  |  |  |  |  |  |
| GCNT3 |  |  |  |  |  |  |  |
| ENSG00000271811 |  |  |  |  |  |  |  |
| CHIT1 |  |  |  |  |  |  |  |
| MTATP6P1 |  |  |  |  |  |  |  |
| PRKAG2 |  |  |  |  |  |  |  |
| TBC1D8 |  |  |  |  |  |  |  |
| NPHP1 |  |  |  |  |  |  |  |
| ELP2 |  |  |  |  |  |  |  |
| PYGL |  |  |  |  |  |  |  |
| RASIP1 |  |  |  |  |  |  |  |
| HMGCL |  |  |  |  |  |  |  |
| C20orf27 |  |  |  |  |  |  |  |
| ENSG00000232710 |  |  |  |  |  |  |  |
| TMEM69 |  |  |  |  |  |  |  |
| ENSG00000230001 |  |  |  |  |  |  |  |
| CRIM1 |  |  |  |  |  |  |  |
| LINC00513 |  |  |  |  |  |  |  |
| ASAH2B |  |  |  |  |  |  |  |
| SEC24B |  |  |  |  |  |  |  |
| PDE5A |  |  |  |  |  |  |  |
| CSRNP1 |  |  |  |  |  |  |  |
| CRYL1 |  |  |  |  |  |  |  |
| AKT3-IT1 |  |  |  |  |  |  |  |
| PPIAP31 |  |  |  |  |  |  |  |
| AMOTL1 |  |  |  |  |  |  |  |
| PLPP5 |  |  |  |  |  |  |  |
| WLS |  |  |  |  |  |  |  |
| ENSG00000280190 |  |  |  |  |  |  |  |
| GEN1 |  |  |  |  |  |  |  |
| ZNF501 |  |  |  |  |  |  |  |
| PRDM16 |  |  |  |  |  |  |  |
| RBKS |  |  |  |  |  |  |  |
| PCBD1 |  |  |  |  |  |  |  |
| CCDC40 |  |  |  |  |  |  |  |
| JUNB |  |  |  |  |  |  |  |
| TIMP1 |  |  |  |  |  |  |  |
| PAF1 |  |  |  |  |  |  |  |
| STUB1 |  |  |  |  |  |  |  |
| JPH1 |  |  |  |  |  |  |  |
| SCAI |  |  |  |  |  |  |  |
| ZSWIM5 |  |  |  |  |  |  |  |
| XYLB |  |  |  |  |  |  |  |
| PXMP2 |  |  |  |  |  |  |  |
| NANOGP4 |  |  |  |  |  |  |  |
| LENG1 |  |  |  |  |  |  |  |
| CCDC191 |  |  |  |  |  |  |  |
| ENSG00000254859 |  |  |  |  |  |  |  |
| MT-ATP6 |  |  |  |  |  |  |  |
| R3HCC1 |  |  |  |  |  |  |  |
| SLC4A8 |  |  |  |  |  |  |  |
| ENTPD7 |  |  |  |  |  |  |  |
| EPHB6 |  |  |  |  |  |  |  |
| PRKAR1B |  |  |  |  |  |  |  |
| MITD1 |  |  |  |  |  |  |  |
| RDH5 |  |  |  |  |  |  |  |
| DUSP10 |  |  |  |  |  |  |  |
| ANGPTL4 |  |  |  |  |  |  |  |
| ZNF71 |  |  |  |  |  |  |  |
| CYP4V2 |  |  |  |  |  |  |  |
| SH3GLB2 |  |  |  |  |  |  |  |
| DYNLT2 |  |  |  |  |  |  |  |
| MTRF1 |  |  |  |  |  |  |  |
| TMEM97 |  |  |  |  |  |  |  |
| CDIN1 |  |  |  |  |  |  |  |
| ENSG00000287299 |  |  |  |  |  |  |  |
| PPARGC1A |  |  |  |  |  |  |  |
| OGFOD3 |  |  |  |  |  |  |  |
| HIF1A |  |  |  |  |  |  |  |
| PPIAP72 |  |  |  |  |  |  |  |
| TRIM65 |  |  |  |  |  |  |  |
| RAMP2 |  |  |  |  |  |  |  |
| ENSG00000265943 |  |  |  |  |  |  |  |
| GCAT |  |  |  |  |  |  |  |
| OXSM |  |  |  |  |  |  |  |
| ENSG00000260337 |  |  |  |  |  |  |  |
| FAM167A |  |  |  |  |  |  |  |
| CAPN10-DT |  |  |  |  |  |  |  |
| ERO1A |  |  |  |  |  |  |  |
| CRACR2B |  |  |  |  |  |  |  |
| ATR |  |  |  |  |  |  |  |
| MOCS1 |  |  |  |  |  |  |  |
| IQUB |  |  |  |  |  |  |  |
| ZNF281 |  |  |  |  |  |  |  |
| NIBAN2 |  |  |  |  |  |  |  |
| ATP1B1P1 |  |  |  |  |  |  |  |
| ACAT1 |  |  |  |  |  |  |  |
| RBM42 |  |  |  |  |  |  |  |
| TMEM43 |  |  |  |  |  |  |  |
| LINC01003 |  |  |  |  |  |  |  |
| CDC16 |  |  |  |  |  |  |  |
| LPIN1 |  |  |  |  |  |  |  |
| RABEPK |  |  |  |  |  |  |  |
| SAR1B |  |  |  |  |  |  |  |
| DEPDC1P2 |  |  |  |  |  |  |  |
| SHC3 |  |  |  |  |  |  |  |
| MYO9A |  |  |  |  |  |  |  |
| SLC66A3 |  |  |  |  |  |  |  |
| EFHC2 |  |  |  |  |  |  |  |
| JAG2 |  |  |  |  |  |  |  |
| MIR593 |  |  |  |  |  |  |  |
| ENSG00000233785 |  |  |  |  |  |  |  |
| HLA-E |  |  |  |  |  |  |  |
| EEF1A1 |  |  |  |  |  |  |  |
| PXK |  |  |  |  |  |  |  |
| SLC2A12 |  |  |  |  |  |  |  |
| JCHAIN |  |  |  |  |  |  |  |
| NRBF2 |  |  |  |  |  |  |  |
| AGAP9 |  |  |  |  |  |  |  |
| WHAMMP2 |  |  |  |  |  |  |  |
| SRF |  |  |  |  |  |  |  |
| ATG2B |  |  |  |  |  |  |  |
| MACO1 |  |  |  |  |  |  |  |
| LDHA |  |  |  |  |  |  |  |
| SORL1 |  |  |  |  |  |  |  |
| AMPH |  |  |  |  |  |  |  |
| MVB12B |  |  |  |  |  |  |  |
| NCOA2 |  |  |  |  |  |  |  |
| ZNF425 |  |  |  |  |  |  |  |
| UGDH |  |  |  |  |  |  |  |
| ENSG00000238035 |  |  |  |  |  |  |  |
| ENSG00000286508 |  |  |  |  |  |  |  |
| KL |  |  |  |  |  |  |  |
| CAPN2 |  |  |  |  |  |  |  |
| GPI |  |  |  |  |  |  |  |
| CCDC14 |  |  |  |  |  |  |  |
| LTA4H |  |  |  |  |  |  |  |
| AP4M1 |  |  |  |  |  |  |  |
| C4BPA |  |  |  |  |  |  |  |
| FNDC3A |  |  |  |  |  |  |  |
| ELMO3 |  |  |  |  |  |  |  |
| ADD2 |  |  |  |  |  |  |  |
| ENSG00000276672 |  |  |  |  |  |  |  |
| RN7SKP203 |  |  |  |  |  |  |  |
| CDKN2AIPNL |  |  |  |  |  |  |  |
| NNMT |  |  |  |  |  |  |  |
| TMEM92-AS1 |  |  |  |  |  |  |  |
| HCAR2 |  |  |  |  |  |  |  |
| ENSG00000273295 |  |  |  |  |  |  |  |
| ZNF366 |  |  |  |  |  |  |  |
| HSD17B12 |  |  |  |  |  |  |  |
| LINC01229 |  |  |  |  |  |  |  |
| GLUD2 |  |  |  |  |  |  |  |
| TRPT1 |  |  |  |  |  |  |  |
| COL4A5 |  |  |  |  |  |  |  |
| LNCSRLR |  |  |  |  |  |  |  |
| CLTB |  |  |  |  |  |  |  |
| BMP1 |  |  |  |  |  |  |  |
| ENSG00000238231 |  |  |  |  |  |  |  |
| CPNE8 |  |  |  |  |  |  |  |
| NGLY1 |  |  |  |  |  |  |  |
| ENSG00000243302 |  |  |  |  |  |  |  |
| ENSG00000279598 |  |  |  |  |  |  |  |
| LINC00640 |  |  |  |  |  |  |  |
| TSPAN13 |  |  |  |  |  |  |  |
| SLC26A6 |  |  |  |  |  |  |  |
| USF3 |  |  |  |  |  |  |  |
| ENSG00000270277 |  |  |  |  |  |  |  |
| SPEF2 |  |  |  |  |  |  |  |
| TINAGL1 |  |  |  |  |  |  |  |
| ENSG00000266378 |  |  |  |  |  |  |  |
| ATG4A |  |  |  |  |  |  |  |
| KCTD6 |  |  |  |  |  |  |  |
| PAQR8 |  |  |  |  |  |  |  |
| ENSG00000228886 |  |  |  |  |  |  |  |
| HMGN2 |  |  |  |  |  |  |  |
| NPR1 |  |  |  |  |  |  |  |
| PHYH |  |  |  |  |  |  |  |
| ENSG00000274364 |  |  |  |  |  |  |  |
| GFM2 |  |  |  |  |  |  |  |
| ISYNA1 |  |  |  |  |  |  |  |
| NAP1L5 |  |  |  |  |  |  |  |
| TMEM107 |  |  |  |  |  |  |  |
| SLC2A4 |  |  |  |  |  |  |  |
| EEF1A1P8 |  |  |  |  |  |  |  |
| TMBIM6 |  |  |  |  |  |  |  |
| CCDC97 |  |  |  |  |  |  |  |
| SIGLEC10 |  |  |  |  |  |  |  |
| H1-4 |  |  |  |  |  |  |  |
| NDN |  |  |  |  |  |  |  |
| TPM3 |  |  |  |  |  |  |  |
| BTC |  |  |  |  |  |  |  |
| SLCO5A1 |  |  |  |  |  |  |  |
| ENSG00000275632 |  |  |  |  |  |  |  |
| EEF1AKMT4 |  |  |  |  |  |  |  |
| SCLY |  |  |  |  |  |  |  |
| EPPK1 |  |  |  |  |  |  |  |
| ATRNL1 |  |  |  |  |  |  |  |
| PPP1R12C |  |  |  |  |  |  |  |
| LINC01176 |  |  |  |  |  |  |  |
| ENSG00000254536 |  |  |  |  |  |  |  |
| BMP2 |  |  |  |  |  |  |  |
| DDAH2 |  |  |  |  |  |  |  |
| CCDC34 |  |  |  |  |  |  |  |
| ANKZF1 |  |  |  |  |  |  |  |
| SLC15A4 |  |  |  |  |  |  |  |
| CMTM4 |  |  |  |  |  |  |  |
| ENSG00000258559 |  |  |  |  |  |  |  |
| FGF1 |  |  |  |  |  |  |  |
| CKMT2 |  |  |  |  |  |  |  |
| RN7SL566P |  |  |  |  |  |  |  |
| SLC47A1 |  |  |  |  |  |  |  |
| MBD4 |  |  |  |  |  |  |  |
| ENSG00000288542 |  |  |  |  |  |  |  |
| SUSD3 |  |  |  |  |  |  |  |
| ENSG00000288559 |  |  |  |  |  |  |  |
| PPP1R9B |  |  |  |  |  |  |  |
| TUSC3 |  |  |  |  |  |  |  |
| PKN1 |  |  |  |  |  |  |  |
| MYH11 |  |  |  |  |  |  |  |
| ENPEP |  |  |  |  |  |  |  |
| BRCC3P1 |  |  |  |  |  |  |  |
| RANBP1 |  |  |  |  |  |  |  |
| C1orf21 |  |  |  |  |  |  |  |
| TTLL7-IT1 |  |  |  |  |  |  |  |
| LIN7B |  |  |  |  |  |  |  |
| GDPD1 |  |  |  |  |  |  |  |
| UNC119 |  |  |  |  |  |  |  |
| MAPK8IP1 |  |  |  |  |  |  |  |
| PACSIN3 |  |  |  |  |  |  |  |
| IGFBP6 |  |  |  |  |  |  |  |
| RFFL |  |  |  |  |  |  |  |
| ENSG00000287978 |  |  |  |  |  |  |  |
| NDUFV2P1 |  |  |  |  |  |  |  |
| MT-ND5 |  |  |  |  |  |  |  |
| PGM5 |  |  |  |  |  |  |  |
| ENSG00000279637 |  |  |  |  |  |  |  |
| ZNF714 |  |  |  |  |  |  |  |
| LINC00957 |  |  |  |  |  |  |  |
| TSPAN9-IT1 |  |  |  |  |  |  |  |
| MRPL35 |  |  |  |  |  |  |  |
| HMGB2 |  |  |  |  |  |  |  |
| APLP2 |  |  |  |  |  |  |  |
| DPYSL2 |  |  |  |  |  |  |  |
| MTFR1 |  |  |  |  |  |  |  |
| BDH1 |  |  |  |  |  |  |  |
| TMEM232 |  |  |  |  |  |  |  |
| ICAM4 |  |  |  |  |  |  |  |
| SNORA33 |  |  |  |  |  |  |  |
| TTLL7 |  |  |  |  |  |  |  |
| ZNF713 |  |  |  |  |  |  |  |
| RCAN3 |  |  |  |  |  |  |  |
| GNG7 |  |  |  |  |  |  |  |
| DRAP1 |  |  |  |  |  |  |  |
| CDC37 |  |  |  |  |  |  |  |
| C9orf43 |  |  |  |  |  |  |  |
| MED9 |  |  |  |  |  |  |  |
| MIR4668 |  |  |  |  |  |  |  |
| MT1X |  |  |  |  |  |  |  |
| HEBP2 |  |  |  |  |  |  |  |
| RPL17P50 |  |  |  |  |  |  |  |
| VMO1 |  |  |  |  |  |  |  |
| SLC25A30 |  |  |  |  |  |  |  |
| CALM3 |  |  |  |  |  |  |  |
| ENSG00000254826 |  |  |  |  |  |  |  |
| ENSG00000264112 |  |  |  |  |  |  |  |
| ZFP28 |  |  |  |  |  |  |  |
| HSP90AA1 |  |  |  |  |  |  |  |
| DLG5 |  |  |  |  |  |  |  |
| ZNF43 |  |  |  |  |  |  |  |
| S100A14 |  |  |  |  |  |  |  |
| EGFL8 |  |  |  |  |  |  |  |
| AGFG2 |  |  |  |  |  |  |  |
| MYO1E |  |  |  |  |  |  |  |
| ENSG00000261770 |  |  |  |  |  |  |  |
| SLC39A4 |  |  |  |  |  |  |  |
| ENSG00000287787 |  |  |  |  |  |  |  |
| GABARAP |  |  |  |  |  |  |  |
| IGLV1-40 |  |  |  |  |  |  |  |
| WDFY1 |  |  |  |  |  |  |  |
| SLC20A1 |  |  |  |  |  |  |  |
| NPY1R |  |  |  |  |  |  |  |
| PCID2 |  |  |  |  |  |  |  |
| HYAL2 |  |  |  |  |  |  |  |
| CFAP119 |  |  |  |  |  |  |  |
| TMEM204 |  |  |  |  |  |  |  |
| GDF7 |  |  |  |  |  |  |  |
| SPAAR |  |  |  |  |  |  |  |
| ENSG00000259810 |  |  |  |  |  |  |  |
| SERTAD3 |  |  |  |  |  |  |  |
| RN7SL1 |  |  |  |  |  |  |  |
| RABEP2 |  |  |  |  |  |  |  |
| ZNF414 |  |  |  |  |  |  |  |
| PCDHB10 |  |  |  |  |  |  |  |
| ACP2 |  |  |  |  |  |  |  |
| SNRNP48 |  |  |  |  |  |  |  |
| RPL7AP6 |  |  |  |  |  |  |  |
| TOM1L1 |  |  |  |  |  |  |  |
| ZCCHC9 |  |  |  |  |  |  |  |
| NAMPTP1 |  |  |  |  |  |  |  |
| PANK3 |  |  |  |  |  |  |  |
| XPOT |  |  |  |  |  |  |  |
| SPEN-AS1 |  |  |  |  |  |  |  |
| TSNAXIP1 |  |  |  |  |  |  |  |
| LINC02943 |  |  |  |  |  |  |  |
| PRKAR2B |  |  |  |  |  |  |  |
| ENSG00000263731 |  |  |  |  |  |  |  |
| TLCD4 |  |  |  |  |  |  |  |
| TBX18 |  |  |  |  |  |  |  |
| PLAC8 |  |  |  |  |  |  |  |
| ST8SIA6 |  |  |  |  |  |  |  |
| VEPH1 |  |  |  |  |  |  |  |
| MRPS6 |  |  |  |  |  |  |  |
| TFDP2 |  |  |  |  |  |  |  |
| RNU6-807P |  |  |  |  |  |  |  |
| UBOX5 |  |  |  |  |  |  |  |
| RFTN2 |  |  |  |  |  |  |  |
| TMLHE |  |  |  |  |  |  |  |
| SLC9A3R1 |  |  |  |  |  |  |  |
| WTAP |  |  |  |  |  |  |  |
| ENSG00000267504 |  |  |  |  |  |  |  |
| ERRFI1 |  |  |  |  |  |  |  |
| PUS10 |  |  |  |  |  |  |  |
| MPZ |  |  |  |  |  |  |  |
| HSD11B1L |  |  |  |  |  |  |  |
| DIPK2B |  |  |  |  |  |  |  |
| ZNF423 |  |  |  |  |  |  |  |
| PAQR5 |  |  |  |  |  |  |  |
| RGN |  |  |  |  |  |  |  |
| CPNE7 |  |  |  |  |  |  |  |
| SERTAD1 |  |  |  |  |  |  |  |
| KRTCAP2 |  |  |  |  |  |  |  |
| CERS2 |  |  |  |  |  |  |  |
| RASGRP3 |  |  |  |  |  |  |  |
| PEMT |  |  |  |  |  |  |  |
| LAMB2 |  |  |  |  |  |  |  |
| PREX2 |  |  |  |  |  |  |  |
| PAFAH1B3 |  |  |  |  |  |  |  |
| SLC44A2 |  |  |  |  |  |  |  |
| WWTR1-IT1 |  |  |  |  |  |  |  |
| UBE2FP1 |  |  |  |  |  |  |  |
| KCTD12 |  |  |  |  |  |  |  |
| CDKN2B |  |  |  |  |  |  |  |
| ENSG00000260948 |  |  |  |  |  |  |  |
| MIR27B |  |  |  |  |  |  |  |
| SLC31A1 |  |  |  |  |  |  |  |
| SLC1A2 |  |  |  |  |  |  |  |
| NAGS |  |  |  |  |  |  |  |
| NMT2 |  |  |  |  |  |  |  |
| IRF2BP1 |  |  |  |  |  |  |  |
| AK3 |  |  |  |  |  |  |  |
| CADM1 |  |  |  |  |  |  |  |
| CCDC51 |  |  |  |  |  |  |  |
| CLEC14A |  |  |  |  |  |  |  |
| HSPD1P11 |  |  |  |  |  |  |  |
| TFB1M |  |  |  |  |  |  |  |
| MFAP4 |  |  |  |  |  |  |  |
| NPIPA1 |  |  |  |  |  |  |  |
| RERG |  |  |  |  |  |  |  |
| DPT |  |  |  |  |  |  |  |
| IL1RL1 |  |  |  |  |  |  |  |
| ENSG00000255142 |  |  |  |  |  |  |  |
| H1-5 |  |  |  |  |  |  |  |
| ADSS1 |  |  |  |  |  |  |  |
| FBXO43 |  |  |  |  |  |  |  |
| FLYWCH2 |  |  |  |  |  |  |  |
| FST |  |  |  |  |  |  |  |
| THRA |  |  |  |  |  |  |  |
| CDHR3 |  |  |  |  |  |  |  |
| ENSG00000254484 |  |  |  |  |  |  |  |
| TMSB10 |  |  |  |  |  |  |  |
| FKBPL |  |  |  |  |  |  |  |
| HLA-DRB5 |  |  |  |  |  |  |  |
| A1BG |  |  |  |  |  |  |  |
| ADCY10 |  |  |  |  |  |  |  |
| METAP1D |  |  |  |  |  |  |  |
| CEP126 |  |  |  |  |  |  |  |
| KLHL25 |  |  |  |  |  |  |  |
| RTN4RL2 |  |  |  |  |  |  |  |
| CPD |  |  |  |  |  |  |  |
| ENSG00000254330 |  |  |  |  |  |  |  |
| MIX23 |  |  |  |  |  |  |  |
| SEMA6A |  |  |  |  |  |  |  |
| PCBP2-OT1 |  |  |  |  |  |  |  |
| PDGFA |  |  |  |  |  |  |  |
| GTF2IP23 |  |  |  |  |  |  |  |
| MAMDC2 |  |  |  |  |  |  |  |
| PTPRB |  |  |  |  |  |  |  |
| NPM1P27 |  |  |  |  |  |  |  |
| SHPK |  |  |  |  |  |  |  |
| ENSG00000280339 |  |  |  |  |  |  |  |
| TRPM7 |  |  |  |  |  |  |  |
| ZNF316 |  |  |  |  |  |  |  |
| MYL9 |  |  |  |  |  |  |  |
| CELSR1 |  |  |  |  |  |  |  |
| TRABD2A |  |  |  |  |  |  |  |
| CBX2 |  |  |  |  |  |  |  |
| SCP2 |  |  |  |  |  |  |  |
| EEF2K |  |  |  |  |  |  |  |
| SNORD101 |  |  |  |  |  |  |  |
| ENSG00000278987 |  |  |  |  |  |  |  |
| HLA-DOA |  |  |  |  |  |  |  |
| TOM1L2 |  |  |  |  |  |  |  |
| NOP2 |  |  |  |  |  |  |  |
| MTIF2 |  |  |  |  |  |  |  |
| RN7SKP292 |  |  |  |  |  |  |  |
| RPL10P9 |  |  |  |  |  |  |  |
| ZNF684 |  |  |  |  |  |  |  |
| COQ9 |  |  |  |  |  |  |  |
| ENSG00000276337 |  |  |  |  |  |  |  |
| ENSG00000270574 |  |  |  |  |  |  |  |
| BICDL2 |  |  |  |  |  |  |  |
| SHE |  |  |  |  |  |  |  |
| ENSG00000285517 |  |  |  |  |  |  |  |
| DERL2 |  |  |  |  |  |  |  |
| SUMO4 |  |  |  |  |  |  |  |
| APOL2 |  |  |  |  |  |  |  |
| CHP1 |  |  |  |  |  |  |  |
| GAS5-AS1 |  |  |  |  |  |  |  |
| ENSG00000234584 |  |  |  |  |  |  |  |
| ZNF101 |  |  |  |  |  |  |  |
| SCARNA7 |  |  |  |  |  |  |  |
| CLDN3 |  |  |  |  |  |  |  |
| MT2A |  |  |  |  |  |  |  |
| ARHGAP10 |  |  |  |  |  |  |  |
| ITPR2 |  |  |  |  |  |  |  |
| IBTK |  |  |  |  |  |  |  |
| AATK |  |  |  |  |  |  |  |
| CHMP6 |  |  |  |  |  |  |  |
| ENSG00000261786 |  |  |  |  |  |  |  |
| PSIP1 |  |  |  |  |  |  |  |
| F2RL2 |  |  |  |  |  |  |  |
| WFDC1 |  |  |  |  |  |  |  |
| TANK |  |  |  |  |  |  |  |
| UNC45A |  |  |  |  |  |  |  |
| AAMP |  |  |  |  |  |  |  |
| B3GALT4 |  |  |  |  |  |  |  |
| ENSG00000278600 |  |  |  |  |  |  |  |
| SLC35D2 |  |  |  |  |  |  |  |
| TMEM33 |  |  |  |  |  |  |  |
| SFTPD |  |  |  |  |  |  |  |
| DTHD1 |  |  |  |  |  |  |  |
| SWAP70 |  |  |  |  |  |  |  |
| ENSG00000280376 |  |  |  |  |  |  |  |
| AXL |  |  |  |  |  |  |  |
| MRPL23 |  |  |  |  |  |  |  |
| TAGLN2 |  |  |  |  |  |  |  |
| CHST12 |  |  |  |  |  |  |  |
| GPHN |  |  |  |  |  |  |  |
| COX5A |  |  |  |  |  |  |  |
| BCL6 |  |  |  |  |  |  |  |
| SLC6A13 |  |  |  |  |  |  |  |
| TST |  |  |  |  |  |  |  |
| SFXN5 |  |  |  |  |  |  |  |
| ZBTB7C |  |  |  |  |  |  |  |
| SERPINI1 |  |  |  |  |  |  |  |
| TSPY26P |  |  |  |  |  |  |  |
| CSRP1-AS1 |  |  |  |  |  |  |  |
| NUDT13 |  |  |  |  |  |  |  |
| COLEC10 |  |  |  |  |  |  |  |
| ENSG00000273284 |  |  |  |  |  |  |  |
| OSCP1 |  |  |  |  |  |  |  |
| TMEM115 |  |  |  |  |  |  |  |
| LRIF1 |  |  |  |  |  |  |  |
| ACADVL |  |  |  |  |  |  |  |
| ARHGEF10L |  |  |  |  |  |  |  |
| CARD8-AS1 |  |  |  |  |  |  |  |
| LINC01750 |  |  |  |  |  |  |  |
| PDZD8 |  |  |  |  |  |  |  |
| PCDHB11 |  |  |  |  |  |  |  |
| PRKAB2 |  |  |  |  |  |  |  |
| CYTH3 |  |  |  |  |  |  |  |
| H2AC20 |  |  |  |  |  |  |  |
| CCDC61 |  |  |  |  |  |  |  |
| ENSG00000256325 |  |  |  |  |  |  |  |
| TMEM243 |  |  |  |  |  |  |  |
| RN7SL381P |  |  |  |  |  |  |  |
| NOS1AP |  |  |  |  |  |  |  |
| PCBP2P2 |  |  |  |  |  |  |  |
| NR1H3 |  |  |  |  |  |  |  |
| CASP4 |  |  |  |  |  |  |  |
| MUC20P1 |  |  |  |  |  |  |  |
| LINC01001 |  |  |  |  |  |  |  |
| TMEM230 |  |  |  |  |  |  |  |
| DDX54 |  |  |  |  |  |  |  |
| DCDC2 |  |  |  |  |  |  |  |
| PKIG |  |  |  |  |  |  |  |
| CCNT2 |  |  |  |  |  |  |  |
| ENSG00000236095 |  |  |  |  |  |  |  |
| RPL7P49 |  |  |  |  |  |  |  |
| RETSAT |  |  |  |  |  |  |  |
| CNTN1 |  |  |  |  |  |  |  |
| FUZ |  |  |  |  |  |  |  |
| PCTP |  |  |  |  |  |  |  |
| SOGA1 |  |  |  |  |  |  |  |
| COL8A2 |  |  |  |  |  |  |  |
| DCTPP1 |  |  |  |  |  |  |  |
| LRRC45 |  |  |  |  |  |  |  |
| ACADL |  |  |  |  |  |  |  |
| WDR83 |  |  |  |  |  |  |  |
| CNOT2 |  |  |  |  |  |  |  |
| ADGRG2 |  |  |  |  |  |  |  |
| RN7SKP78 |  |  |  |  |  |  |  |
| SNORA54 |  |  |  |  |  |  |  |
| ENSG00000260144 |  |  |  |  |  |  |  |
| UCHL3 |  |  |  |  |  |  |  |
| CBS |  |  |  |  |  |  |  |
| PRKCZ |  |  |  |  |  |  |  |
| DAAM2-AS1 |  |  |  |  |  |  |  |
| ABHD3 |  |  |  |  |  |  |  |
| ZFP91 |  |  |  |  |  |  |  |
| ARMC2 |  |  |  |  |  |  |  |
| ABHD2 |  |  |  |  |  |  |  |
| ENSG00000225026 |  |  |  |  |  |  |  |
| ENSG00000286406 |  |  |  |  |  |  |  |
| NPTX2 |  |  |  |  |  |  |  |
| ADAM20 |  |  |  |  |  |  |  |
| RN7SKP296 |  |  |  |  |  |  |  |
| ENSG00000277246 |  |  |  |  |  |  |  |
| MPND |  |  |  |  |  |  |  |
| UGP2 |  |  |  |  |  |  |  |
| IL1RAP |  |  |  |  |  |  |  |
| RBMXP2 |  |  |  |  |  |  |  |
| GUF1 |  |  |  |  |  |  |  |
| ZNF66 |  |  |  |  |  |  |  |
| CROCCP3 |  |  |  |  |  |  |  |
| AIG1 |  |  |  |  |  |  |  |
| ATAT1 |  |  |  |  |  |  |  |
| NDUFB7 |  |  |  |  |  |  |  |
| AKR1C3 |  |  |  |  |  |  |  |
| CYBRD1 |  |  |  |  |  |  |  |
| NUDT18 |  |  |  |  |  |  |  |
| NME5 |  |  |  |  |  |  |  |
| CABYR |  |  |  |  |  |  |  |
| CCDC81 |  |  |  |  |  |  |  |
| SNORA80A |  |  |  |  |  |  |  |
| LINC02883 |  |  |  |  |  |  |  |
| ZNF483 |  |  |  |  |  |  |  |
| ENSG00000262140 |  |  |  |  |  |  |  |
| NUGGC |  |  |  |  |  |  |  |
| INTU |  |  |  |  |  |  |  |
| ENSG00000267838 |  |  |  |  |  |  |  |
| SFRP1 |  |  |  |  |  |  |  |
| TRIOBP |  |  |  |  |  |  |  |
| DIPK1A |  |  |  |  |  |  |  |
| TMEM200C |  |  |  |  |  |  |  |
| MRPL46 |  |  |  |  |  |  |  |
| HHIP |  |  |  |  |  |  |  |
| ORAI3 |  |  |  |  |  |  |  |
| GPR89A |  |  |  |  |  |  |  |
| DNAH7 |  |  |  |  |  |  |  |
| CACHD1 |  |  |  |  |  |  |  |
| ENSG00000280332 |  |  |  |  |  |  |  |
| ENSG00000282936 |  |  |  |  |  |  |  |
| GUSBP18 |  |  |  |  |  |  |  |
| CHAF1A |  |  |  |  |  |  |  |
| KCNMA1 |  |  |  |  |  |  |  |
| CFL1P5 |  |  |  |  |  |  |  |
| SMPD1 |  |  |  |  |  |  |  |
| CLSTN1 |  |  |  |  |  |  |  |
| ARMC7 |  |  |  |  |  |  |  |
| LONP2 |  |  |  |  |  |  |  |
| PRDM5 |  |  |  |  |  |  |  |
| RAB23 |  |  |  |  |  |  |  |
| CASKIN2 |  |  |  |  |  |  |  |
| CENPBD2P |  |  |  |  |  |  |  |
| SLC27A3 |  |  |  |  |  |  |  |
| SANBR |  |  |  |  |  |  |  |
| PPP1R3B |  |  |  |  |  |  |  |
| BEX5 |  |  |  |  |  |  |  |
| SERPINE2 |  |  |  |  |  |  |  |
| ETV6 |  |  |  |  |  |  |  |
| SNHG4 |  |  |  |  |  |  |  |
| ENSG00000282221 |  |  |  |  |  |  |  |
| RAB26 |  |  |  |  |  |  |  |
| ZNF282 |  |  |  |  |  |  |  |
| TMOD1 |  |  |  |  |  |  |  |
| SDSL |  |  |  |  |  |  |  |
| C5orf22 |  |  |  |  |  |  |  |
| ZBTB42 |  |  |  |  |  |  |  |
| ENSG00000237927 |  |  |  |  |  |  |  |
| CDR2L |  |  |  |  |  |  |  |
| PROS1 |  |  |  |  |  |  |  |
| ATP5F1B |  |  |  |  |  |  |  |
| RPS3P2 |  |  |  |  |  |  |  |
| SRSF12 |  |  |  |  |  |  |  |
| ENSG00000260196 |  |  |  |  |  |  |  |
| RNF103 |  |  |  |  |  |  |  |
| C17orf100 |  |  |  |  |  |  |  |
| LINC00528 |  |  |  |  |  |  |  |
| ENSG00000261072 |  |  |  |  |  |  |  |
| TMEM86B |  |  |  |  |  |  |  |
| ACOX2 |  |  |  |  |  |  |  |
| HBEGF |  |  |  |  |  |  |  |
| LINC01409 |  |  |  |  |  |  |  |
| MTCO1P11 |  |  |  |  |  |  |  |
| EHD2 |  |  |  |  |  |  |  |
| NXN |  |  |  |  |  |  |  |
| TRAPPC6A |  |  |  |  |  |  |  |
| USP35 |  |  |  |  |  |  |  |
| DPCD |  |  |  |  |  |  |  |
| ENSG00000232546 |  |  |  |  |  |  |  |
| ENSG00000258539 |  |  |  |  |  |  |  |
| RBPMS-AS1 |  |  |  |  |  |  |  |
| CHMP4BP1 |  |  |  |  |  |  |  |
| TSNARE1 |  |  |  |  |  |  |  |
| TREM1 |  |  |  |  |  |  |  |
| ADRA1A |  |  |  |  |  |  |  |
| C1orf226 |  |  |  |  |  |  |  |
| PPT2 |  |  |  |  |  |  |  |
| ZNF28 |  |  |  |  |  |  |  |
| ENSG00000250771 |  |  |  |  |  |  |  |
| CLGN |  |  |  |  |  |  |  |
| NANOGP5 |  |  |  |  |  |  |  |
| G0S2 |  |  |  |  |  |  |  |
| SPSB2 |  |  |  |  |  |  |  |
| WNT3 |  |  |  |  |  |  |  |
| LCN12 |  |  |  |  |  |  |  |
| ZNRF2P1 |  |  |  |  |  |  |  |
| NUDT6 |  |  |  |  |  |  |  |
| MTFR2 |  |  |  |  |  |  |  |
| CASTOR2 |  |  |  |  |  |  |  |
| CACNA1C-IT3 |  |  |  |  |  |  |  |
| CHAC2 |  |  |  |  |  |  |  |
| STMN1 |  |  |  |  |  |  |  |
| CLCN5 |  |  |  |  |  |  |  |
| S100A10 |  |  |  |  |  |  |  |
| RRS1 |  |  |  |  |  |  |  |
| RN7SL735P |  |  |  |  |  |  |  |
| TJP2 |  |  |  |  |  |  |  |
| IDH2 |  |  |  |  |  |  |  |
| SEMA6D |  |  |  |  |  |  |  |
| ICAM2 |  |  |  |  |  |  |  |
| RNF157 |  |  |  |  |  |  |  |
| OSGIN1 |  |  |  |  |  |  |  |
| ZFP69B |  |  |  |  |  |  |  |
| IL22RA1 |  |  |  |  |  |  |  |
| SKI |  |  |  |  |  |  |  |
| GTF2IP5 |  |  |  |  |  |  |  |
| NPEPPSP1 |  |  |  |  |  |  |  |
| ENSG00000226149 |  |  |  |  |  |  |  |
| PTCD2 |  |  |  |  |  |  |  |
| KRTCAP3 |  |  |  |  |  |  |  |
| CCNB1IP1 |  |  |  |  |  |  |  |
| CEP131 |  |  |  |  |  |  |  |
| DNASE1L3 |  |  |  |  |  |  |  |
| CDO1 |  |  |  |  |  |  |  |
| RPS27AP12 |  |  |  |  |  |  |  |
| GCSH |  |  |  |  |  |  |  |
| CCDC15 |  |  |  |  |  |  |  |
| VAMP5 |  |  |  |  |  |  |  |
| ENSG00000248840 |  |  |  |  |  |  |  |
| C3orf86 |  |  |  |  |  |  |  |
| LDHD |  |  |  |  |  |  |  |
| NDNF |  |  |  |  |  |  |  |
| NAV2-AS6 |  |  |  |  |  |  |  |
| PLPP6 |  |  |  |  |  |  |  |
| CDKL5 |  |  |  |  |  |  |  |
| EFNB1 |  |  |  |  |  |  |  |
| BPHL |  |  |  |  |  |  |  |
| TAS2R5 |  |  |  |  |  |  |  |
| PRAG1 |  |  |  |  |  |  |  |
| TMEM182 |  |  |  |  |  |  |  |
| PIWIL2 |  |  |  |  |  |  |  |
| SPTLC1P1 |  |  |  |  |  |  |  |
| MT1A |  |  |  |  |  |  |  |
| C8orf44 |  |  |  |  |  |  |  |
| ANKHD1 |  |  |  |  |  |  |  |
| COX8A |  |  |  |  |  |  |  |
| MVK |  |  |  |  |  |  |  |
| ANKRD10-IT1 |  |  |  |  |  |  |  |
| ENSG00000285791 |  |  |  |  |  |  |  |
| EPAS1 |  |  |  |  |  |  |  |
| HSPA8P15 |  |  |  |  |  |  |  |
| C1orf116 |  |  |  |  |  |  |  |
| MT-ND4 |  |  |  |  |  |  |  |
| TGFB2 |  |  |  |  |  |  |  |
| TNRC18P1 |  |  |  |  |  |  |  |
| LRP2BP-AS1 |  |  |  |  |  |  |  |
| SLC25A25-AS1 |  |  |  |  |  |  |  |
| BNIPL |  |  |  |  |  |  |  |
| BCKDK |  |  |  |  |  |  |  |
| ENSG00000234624 |  |  |  |  |  |  |  |
| IGFBP2 |  |  |  |  |  |  |  |
| RWDD4 |  |  |  |  |  |  |  |
| GPR89B |  |  |  |  |  |  |  |
| STARD9 |  |  |  |  |  |  |  |
| CCN2 |  |  |  |  |  |  |  |
| MAMDC4 |  |  |  |  |  |  |  |
| CCDC168 |  |  |  |  |  |  |  |
| AGL |  |  |  |  |  |  |  |
| ZNF212 |  |  |  |  |  |  |  |
| ENSG00000267682 |  |  |  |  |  |  |  |
| ENSG00000257298 |  |  |  |  |  |  |  |
| FGL2 |  |  |  |  |  |  |  |
| ENSG00000225339 |  |  |  |  |  |  |  |
| ENSG00000286977 |  |  |  |  |  |  |  |
| TAB1 |  |  |  |  |  |  |  |
| RGS2 |  |  |  |  |  |  |  |
| DUSP5-DT |  |  |  |  |  |  |  |
| RABL6 |  |  |  |  |  |  |  |
| NDUFB4 |  |  |  |  |  |  |  |
| ENO3 |  |  |  |  |  |  |  |
| SCAF1 |  |  |  |  |  |  |  |
| FAM151B-DT |  |  |  |  |  |  |  |
| PKD2 |  |  |  |  |  |  |  |
| RPL7AP45 |  |  |  |  |  |  |  |
| TAOK2 |  |  |  |  |  |  |  |
| MICAL3 |  |  |  |  |  |  |  |
| DOHH |  |  |  |  |  |  |  |
| ENSG00000269968 |  |  |  |  |  |  |  |
| CEP68 |  |  |  |  |  |  |  |
| CD9 |  |  |  |  |  |  |  |
| HDAC11 |  |  |  |  |  |  |  |
| AK8 |  |  |  |  |  |  |  |
| TAS2R64P |  |  |  |  |  |  |  |
| BCKDHB |  |  |  |  |  |  |  |
| VPS37A |  |  |  |  |  |  |  |
| RNU1-16P |  |  |  |  |  |  |  |
| DCUN1D4 |  |  |  |  |  |  |  |
| USPL1 |  |  |  |  |  |  |  |
| PRRG1 |  |  |  |  |  |  |  |
| PDCD2L |  |  |  |  |  |  |  |
| CD2BP2 |  |  |  |  |  |  |  |
| RPL19 |  |  |  |  |  |  |  |
| MGST2 |  |  |  |  |  |  |  |
| TACSTD2 |  |  |  |  |  |  |  |
| HLA-DQA1 |  |  |  |  |  |  |  |
| OSBP |  |  |  |  |  |  |  |
| TCEAL3 |  |  |  |  |  |  |  |
| ASCC1 |  |  |  |  |  |  |  |
| RBBP4P1 |  |  |  |  |  |  |  |
| SMIM30 |  |  |  |  |  |  |  |
| ARHGAP26-IT1 |  |  |  |  |  |  |  |
| INCENP |  |  |  |  |  |  |  |
| HDC |  |  |  |  |  |  |  |
| MLPH |  |  |  |  |  |  |  |
| NELFE |  |  |  |  |  |  |  |
| ENSG00000286813 |  |  |  |  |  |  |  |
| MIEF2 |  |  |  |  |  |  |  |
| LARGE2 |  |  |  |  |  |  |  |
| PELP1 |  |  |  |  |  |  |  |
| VPS26BP1 |  |  |  |  |  |  |  |
| SLC5A6 |  |  |  |  |  |  |  |
| ENSG00000280054 |  |  |  |  |  |  |  |
| ZNF605 |  |  |  |  |  |  |  |
| GRTP1 |  |  |  |  |  |  |  |
| ENSG00000288612 |  |  |  |  |  |  |  |
| ENSG00000279819 |  |  |  |  |  |  |  |
| C6orf136 |  |  |  |  |  |  |  |
| KIAA1549 |  |  |  |  |  |  |  |
| TNFAIP8 |  |  |  |  |  |  |  |
| ECHDC2 |  |  |  |  |  |  |  |
| USP30 |  |  |  |  |  |  |  |
| INAFM1 |  |  |  |  |  |  |  |
| NTN4 |  |  |  |  |  |  |  |
| ENSG00000230454 |  |  |  |  |  |  |  |
| HERC3 |  |  |  |  |  |  |  |
| IGF1R |  |  |  |  |  |  |  |
| MIR4435-2HG |  |  |  |  |  |  |  |
| PLCH1 |  |  |  |  |  |  |  |
| LMNB2 |  |  |  |  |  |  |  |
| DNAH6 |  |  |  |  |  |  |  |
| ZNF362 |  |  |  |  |  |  |  |
| ZIK1 |  |  |  |  |  |  |  |
| STRCP1 |  |  |  |  |  |  |  |
| ENSG00000234589 |  |  |  |  |  |  |  |
| SIMC1 |  |  |  |  |  |  |  |
| DAP |  |  |  |  |  |  |  |
| ZNF775 |  |  |  |  |  |  |  |
| ROBO4 |  |  |  |  |  |  |  |
| PCCB |  |  |  |  |  |  |  |
| PAPPA |  |  |  |  |  |  |  |
| HIP1 |  |  |  |  |  |  |  |
| APBB1IP |  |  |  |  |  |  |  |
| ENSG00000255067 |  |  |  |  |  |  |  |
| PKP3 |  |  |  |  |  |  |  |
| DUSP1 |  |  |  |  |  |  |  |
| PTPN13 |  |  |  |  |  |  |  |
| RPS6KA4 |  |  |  |  |  |  |  |
| PMEPA1 |  |  |  |  |  |  |  |
| RESF1 |  |  |  |  |  |  |  |
| C5orf15 |  |  |  |  |  |  |  |
| SCCPDH |  |  |  |  |  |  |  |
| KRI1 |  |  |  |  |  |  |  |
| RPL8 |  |  |  |  |  |  |  |
| DSTN |  |  |  |  |  |  |  |
| MIR2467 |  |  |  |  |  |  |  |
| RTL6 |  |  |  |  |  |  |  |
| ARHGEF2-AS2 |  |  |  |  |  |  |  |
| GRIP2 |  |  |  |  |  |  |  |
| RN7SL517P |  |  |  |  |  |  |  |
| CERS6 |  |  |  |  |  |  |  |
| SLC2A5 |  |  |  |  |  |  |  |
| ID2 |  |  |  |  |  |  |  |
| IQSEC3 |  |  |  |  |  |  |  |
| RHOQ |  |  |  |  |  |  |  |
| CUEDC1 |  |  |  |  |  |  |  |
| ORMDL1 |  |  |  |  |  |  |  |
| RBM24 |  |  |  |  |  |  |  |
| POPDC2 |  |  |  |  |  |  |  |
| HIGD1A |  |  |  |  |  |  |  |
| MAPK10 |  |  |  |  |  |  |  |
| HNRNPA1L3 |  |  |  |  |  |  |  |
| AIF1 |  |  |  |  |  |  |  |
| JCAD |  |  |  |  |  |  |  |
| DMXL2 |  |  |  |  |  |  |  |
| UGCG |  |  |  |  |  |  |  |
| LINC02197 |  |  |  |  |  |  |  |
| FASTKD1 |  |  |  |  |  |  |  |
| CTNNBIP1 |  |  |  |  |  |  |  |
| SNORD3B-1 |  |  |  |  |  |  |  |
| APOO |  |  |  |  |  |  |  |
| SYF2 |  |  |  |  |  |  |  |
| KCNB1 |  |  |  |  |  |  |  |
| IGHA1 |  |  |  |  |  |  |  |
| ENSG00000230896 |  |  |  |  |  |  |  |
| ENSG00000227681 |  |  |  |  |  |  |  |
| RRS1-DT |  |  |  |  |  |  |  |
| RPL18 |  |  |  |  |  |  |  |
| EPB41L1 |  |  |  |  |  |  |  |
| GIPC1 |  |  |  |  |  |  |  |
| PCDHB15 |  |  |  |  |  |  |  |
| FDPS |  |  |  |  |  |  |  |
| SLC9A3R2 |  |  |  |  |  |  |  |
| RRP9 |  |  |  |  |  |  |  |
| DECR1 |  |  |  |  |  |  |  |
| ZBTB40-IT1 |  |  |  |  |  |  |  |
| ENSG00000261026 |  |  |  |  |  |  |  |
| DACH1 |  |  |  |  |  |  |  |
| GHR |  |  |  |  |  |  |  |
| ENSG00000278863 |  |  |  |  |  |  |  |
| REXO5 |  |  |  |  |  |  |  |
| SMOX |  |  |  |  |  |  |  |
| RPL26 |  |  |  |  |  |  |  |
| GALNT6 |  |  |  |  |  |  |  |
| UQCRC2 |  |  |  |  |  |  |  |
| PRKG1 |  |  |  |  |  |  |  |
| OLFML3 |  |  |  |  |  |  |  |
| ECM1 |  |  |  |  |  |  |  |
| CES1 |  |  |  |  |  |  |  |
| MAP9 |  |  |  |  |  |  |  |
| SNORD13E |  |  |  |  |  |  |  |
| LHFPL6 |  |  |  |  |  |  |  |
| SLC25A42 |  |  |  |  |  |  |  |
| MTCO2P11 |  |  |  |  |  |  |  |
| TEKT5 |  |  |  |  |  |  |  |
| ENSG00000278876 |  |  |  |  |  |  |  |
| STK17B |  |  |  |  |  |  |  |
| RPS2P55 |  |  |  |  |  |  |  |
| NACAD |  |  |  |  |  |  |  |
| NAT14 |  |  |  |  |  |  |  |
| ACTG1P20 |  |  |  |  |  |  |  |
| LINC02916 |  |  |  |  |  |  |  |
| TFEB |  |  |  |  |  |  |  |
| NFATC1 |  |  |  |  |  |  |  |
| TRAM2 |  |  |  |  |  |  |  |
| PHF2 |  |  |  |  |  |  |  |
| POR |  |  |  |  |  |  |  |
| FAM229B |  |  |  |  |  |  |  |
| CHKA |  |  |  |  |  |  |  |
| NTHL1 |  |  |  |  |  |  |  |
| APMAP |  |  |  |  |  |  |  |
| ALKBH4 |  |  |  |  |  |  |  |
| ENSG00000269924 |  |  |  |  |  |  |  |
| PDLIM5 |  |  |  |  |  |  |  |
| CYP39A1 |  |  |  |  |  |  |  |
| LOX |  |  |  |  |  |  |  |
| PGAM1P7 |  |  |  |  |  |  |  |
| VDAC1P1 |  |  |  |  |  |  |  |
| ENSG00000283674 |  |  |  |  |  |  |  |
| WAPL-DT |  |  |  |  |  |  |  |
| YPEL1 |  |  |  |  |  |  |  |
| ZNF727 |  |  |  |  |  |  |  |
| GTF3A |  |  |  |  |  |  |  |
| ENSG00000264853 |  |  |  |  |  |  |  |
| MFAP3L |  |  |  |  |  |  |  |
| ENSG00000267096 |  |  |  |  |  |  |  |
| DCAF4L1 |  |  |  |  |  |  |  |
| ENSG00000227304 |  |  |  |  |  |  |  |
| LLGL2 |  |  |  |  |  |  |  |
| MCM3AP-AS1 |  |  |  |  |  |  |  |
| TIMM23 |  |  |  |  |  |  |  |
| NRARP |  |  |  |  |  |  |  |
| STK32C |  |  |  |  |  |  |  |
| FAM131A |  |  |  |  |  |  |  |
| DHRS13 |  |  |  |  |  |  |  |
| ITGA1 |  |  |  |  |  |  |  |
| DUSP3 |  |  |  |  |  |  |  |
| RAB25 |  |  |  |  |  |  |  |
| ENSG00000233967 |  |  |  |  |  |  |  |
| HUNK |  |  |  |  |  |  |  |
| MT1M |  |  |  |  |  |  |  |
| CIRBP |  |  |  |  |  |  |  |
| RPS15AP6 |  |  |  |  |  |  |  |
| FAM43A |  |  |  |  |  |  |  |
| FLT4 |  |  |  |  |  |  |  |
| GIMAP6 |  |  |  |  |  |  |  |
| CRADD |  |  |  |  |  |  |  |
| DUS3L |  |  |  |  |  |  |  |
| ENSG00000236525 |  |  |  |  |  |  |  |
| PCDHA4 |  |  |  |  |  |  |  |
| MT1E |  |  |  |  |  |  |  |
| H1-10 |  |  |  |  |  |  |  |
| ENSG00000257663 |  |  |  |  |  |  |  |
| TMEM54 |  |  |  |  |  |  |  |
| HSPA4L |  |  |  |  |  |  |  |
| IGHV5-51 |  |  |  |  |  |  |  |
| SC5D |  |  |  |  |  |  |  |
| TMEM30B |  |  |  |  |  |  |  |
| FADS2 |  |  |  |  |  |  |  |
| EFCAB6 |  |  |  |  |  |  |  |
| INAVA |  |  |  |  |  |  |  |
| DUSP18 |  |  |  |  |  |  |  |
| RN7SL398P |  |  |  |  |  |  |  |
| CAPG |  |  |  |  |  |  |  |
| SLC5A3 |  |  |  |  |  |  |  |
| LINC00997 |  |  |  |  |  |  |  |
| FMO4 |  |  |  |  |  |  |  |
| RPL4P5 |  |  |  |  |  |  |  |
| SRL |  |  |  |  |  |  |  |
| EFNB2 |  |  |  |  |  |  |  |
| ENSG00000260971 |  |  |  |  |  |  |  |
| ULK2 |  |  |  |  |  |  |  |
| HYKK |  |  |  |  |  |  |  |
| ACSS1 |  |  |  |  |  |  |  |
| BANK1 |  |  |  |  |  |  |  |
| ENSG00000273691 |  |  |  |  |  |  |  |
| RPS15P4 |  |  |  |  |  |  |  |
| SYT17 |  |  |  |  |  |  |  |
| HIBCH |  |  |  |  |  |  |  |
| C1orf220 |  |  |  |  |  |  |  |
| ENSG00000255165 |  |  |  |  |  |  |  |
| USP11 |  |  |  |  |  |  |  |
| GATA2-AS1 |  |  |  |  |  |  |  |
| TMEM38B |  |  |  |  |  |  |  |
| LRRC34 |  |  |  |  |  |  |  |
| NPM1P37 |  |  |  |  |  |  |  |
| ACSS3 |  |  |  |  |  |  |  |
| ENSG00000232626 |  |  |  |  |  |  |  |
| ENSG00000261659 |  |  |  |  |  |  |  |
| EEF1AKMT3 |  |  |  |  |  |  |  |
| GRHPR |  |  |  |  |  |  |  |
| ENSG00000256341 |  |  |  |  |  |  |  |
| HEG1 |  |  |  |  |  |  |  |
| ECHDC3 |  |  |  |  |  |  |  |
| SCARA5 |  |  |  |  |  |  |  |
| ENSG00000285679 |  |  |  |  |  |  |  |
| LRRC56 |  |  |  |  |  |  |  |
| ZC3H18 |  |  |  |  |  |  |  |
| LPCAT1 |  |  |  |  |  |  |  |
| ZDHHC9 |  |  |  |  |  |  |  |
| ARHGAP24 |  |  |  |  |  |  |  |
| ABCC6P2 |  |  |  |  |  |  |  |
| VWA2 |  |  |  |  |  |  |  |
| TPGS1 |  |  |  |  |  |  |  |
| BACH1-IT2 |  |  |  |  |  |  |  |
| RPS14P4 |  |  |  |  |  |  |  |
| TMEM120A |  |  |  |  |  |  |  |
| LCAT |  |  |  |  |  |  |  |
| ENSG00000273270 |  |  |  |  |  |  |  |
| ENSG00000235445 |  |  |  |  |  |  |  |
| CNKSR1 |  |  |  |  |  |  |  |
| ST14 |  |  |  |  |  |  |  |
| TOMM70 |  |  |  |  |  |  |  |
| ENSG00000287306 |  |  |  |  |  |  |  |
| LYPLA1 |  |  |  |  |  |  |  |
| SLFNL1-AS1 |  |  |  |  |  |  |  |
| MGC27382 |  |  |  |  |  |  |  |
| MYO10 |  |  |  |  |  |  |  |
| PPP1R15A |  |  |  |  |  |  |  |
| MAL2 |  |  |  |  |  |  |  |
| SSTR1 |  |  |  |  |  |  |  |
| TAS2R6P |  |  |  |  |  |  |  |
| NELL2 |  |  |  |  |  |  |  |
| UBA6 |  |  |  |  |  |  |  |
| EPHX2 |  |  |  |  |  |  |  |
| RPL35AP2 |  |  |  |  |  |  |  |
| N4BP2L2-IT2 |  |  |  |  |  |  |  |
| ALDH5A1 |  |  |  |  |  |  |  |
| TMEM184A |  |  |  |  |  |  |  |
| PPP1R37 |  |  |  |  |  |  |  |
| SYCE1L |  |  |  |  |  |  |  |
| MTCYBP3 |  |  |  |  |  |  |  |
| MPL |  |  |  |  |  |  |  |
| MTND5P2 |  |  |  |  |  |  |  |
| CYP2S1 |  |  |  |  |  |  |  |
| MPC2 |  |  |  |  |  |  |  |
| ENSG00000274080 |  |  |  |  |  |  |  |
| OLR1 |  |  |  |  |  |  |  |
| CRYZ |  |  |  |  |  |  |  |
| SNORD46 |  |  |  |  |  |  |  |
| SDHB |  |  |  |  |  |  |  |
| DHFR2 |  |  |  |  |  |  |  |
| PCSK5 |  |  |  |  |  |  |  |
| PPP1R13L |  |  |  |  |  |  |  |
| LRRC39 |  |  |  |  |  |  |  |
| ADHFE1 |  |  |  |  |  |  |  |
| ENSG00000273192 |  |  |  |  |  |  |  |
| HSP90B2P |  |  |  |  |  |  |  |
| ENSG00000272754 |  |  |  |  |  |  |  |
| MYO5C |  |  |  |  |  |  |  |
| MRPS31P5 |  |  |  |  |  |  |  |
| KRT19 |  |  |  |  |  |  |  |
| HSD17B13 |  |  |  |  |  |  |  |
| CLIC6 |  |  |  |  |  |  |  |
| C5orf34 |  |  |  |  |  |  |  |
| POLN |  |  |  |  |  |  |  |
| TMEM30BP1 |  |  |  |  |  |  |  |
| CHCHD10 |  |  |  |  |  |  |  |
| LRRC37A15P |  |  |  |  |  |  |  |
| NEXMIF |  |  |  |  |  |  |  |
| CCDC74A |  |  |  |  |  |  |  |
| HARS1 |  |  |  |  |  |  |  |
| LINC02246 |  |  |  |  |  |  |  |
| TMEM125 |  |  |  |  |  |  |  |
| ENSG00000260855 |  |  |  |  |  |  |  |
| ELAPOR1 |  |  |  |  |  |  |  |
| ZNF783 |  |  |  |  |  |  |  |
| CASC2 |  |  |  |  |  |  |  |
| ADRB2 |  |  |  |  |  |  |  |
| H1-1 |  |  |  |  |  |  |  |
| TAS2R4 |  |  |  |  |  |  |  |
| GLIS2 |  |  |  |  |  |  |  |
| DNHD1 |  |  |  |  |  |  |  |
| RAB19 |  |  |  |  |  |  |  |
| ADAMTS17 |  |  |  |  |  |  |  |
| ENSG00000278351 |  |  |  |  |  |  |  |
| PA2G4P4 |  |  |  |  |  |  |  |
| SIPA1L3 |  |  |  |  |  |  |  |
| MANF |  |  |  |  |  |  |  |
| MSRB3 |  |  |  |  |  |  |  |
| PRDM6 |  |  |  |  |  |  |  |
| ASL |  |  |  |  |  |  |  |
| GPM6B |  |  |  |  |  |  |  |
| NDUFAF4 |  |  |  |  |  |  |  |
| ARMCX6 |  |  |  |  |  |  |  |
| PCK2 |  |  |  |  |  |  |  |
| EEF1A1P9 |  |  |  |  |  |  |  |
| LINC00924 |  |  |  |  |  |  |  |
| ENSG00000280120 |  |  |  |  |  |  |  |
| ENSG00000238390 |  |  |  |  |  |  |  |
| APOBEC3C |  |  |  |  |  |  |  |
| KCNN3 |  |  |  |  |  |  |  |
| ENSG00000259692 |  |  |  |  |  |  |  |
| GOLGA6L5P |  |  |  |  |  |  |  |
| RPP25 |  |  |  |  |  |  |  |
| ENSG00000270012 |  |  |  |  |  |  |  |
| PMVK |  |  |  |  |  |  |  |
| RPL24 |  |  |  |  |  |  |  |
| SUCLG2 |  |  |  |  |  |  |  |
| LAMA5 |  |  |  |  |  |  |  |
| GBP2 |  |  |  |  |  |  |  |
| RGS11 |  |  |  |  |  |  |  |
| NFKBIL1 |  |  |  |  |  |  |  |
| GARS1 |  |  |  |  |  |  |  |
| FGF9 |  |  |  |  |  |  |  |
| BCL2L2-PABPN1 |  |  |  |  |  |  |  |
| SCEL |  |  |  |  |  |  |  |
| ENSG00000213703 |  |  |  |  |  |  |  |
| ENSG00000273568 |  |  |  |  |  |  |  |
| ENSG00000280077 |  |  |  |  |  |  |  |
| NPC2 |  |  |  |  |  |  |  |
| RNU6-1157P |  |  |  |  |  |  |  |
| RN7SL430P |  |  |  |  |  |  |  |
| RAB1AP1 |  |  |  |  |  |  |  |
| NHLRC1 |  |  |  |  |  |  |  |
| ZDHHC2 |  |  |  |  |  |  |  |
| EFHD2 |  |  |  |  |  |  |  |
| MSANTD3 |  |  |  |  |  |  |  |
| FDFT1 |  |  |  |  |  |  |  |
| ANP32E |  |  |  |  |  |  |  |
| MCM3 |  |  |  |  |  |  |  |
| DNA2 |  |  |  |  |  |  |  |
| TIMP3 |  |  |  |  |  |  |  |
| COL18A1 |  |  |  |  |  |  |  |
| PPP1R36 |  |  |  |  |  |  |  |
| ENSG00000283057 |  |  |  |  |  |  |  |
| FDXR |  |  |  |  |  |  |  |
| TMEM38A |  |  |  |  |  |  |  |
| RHOF |  |  |  |  |  |  |  |
| TKFC |  |  |  |  |  |  |  |
| TSPAN32 |  |  |  |  |  |  |  |
| OSMR |  |  |  |  |  |  |  |
| NELFB |  |  |  |  |  |  |  |
| RGL2 |  |  |  |  |  |  |  |
| GUCD1 |  |  |  |  |  |  |  |
| ENSG00000257681 |  |  |  |  |  |  |  |
| EIF3C |  |  |  |  |  |  |  |
| PTGER4 |  |  |  |  |  |  |  |
| EDNRA |  |  |  |  |  |  |  |
| EIF4BP7 |  |  |  |  |  |  |  |
| ENSG00000203644 |  |  |  |  |  |  |  |
| ENSG00000239280 |  |  |  |  |  |  |  |
| LRRCC1 |  |  |  |  |  |  |  |
| ENSG00000279539 |  |  |  |  |  |  |  |
| FBL |  |  |  |  |  |  |  |
| LYRM4-AS1 |  |  |  |  |  |  |  |
| ZNF668 |  |  |  |  |  |  |  |
| INSR |  |  |  |  |  |  |  |
| ENSG00000279106 |  |  |  |  |  |  |  |
| GNGT2 |  |  |  |  |  |  |  |
| GCDH |  |  |  |  |  |  |  |
| CPEB3 |  |  |  |  |  |  |  |
| HESX1 |  |  |  |  |  |  |  |
| FUCA2 |  |  |  |  |  |  |  |
| HMGB1P6 |  |  |  |  |  |  |  |
| RPL41 |  |  |  |  |  |  |  |
| RN7SL473P |  |  |  |  |  |  |  |
| GLIPR1-AS1 |  |  |  |  |  |  |  |
| ASB13 |  |  |  |  |  |  |  |
| SLC30A1 |  |  |  |  |  |  |  |
| ARHGAP31 |  |  |  |  |  |  |  |
| HACD4 |  |  |  |  |  |  |  |
| SCN5A |  |  |  |  |  |  |  |
| IFT22 |  |  |  |  |  |  |  |
| TRADD |  |  |  |  |  |  |  |
| ASAP1-IT2 |  |  |  |  |  |  |  |
| ISOC1 |  |  |  |  |  |  |  |
| FAM124B |  |  |  |  |  |  |  |
| FZD6 |  |  |  |  |  |  |  |
| PLTP |  |  |  |  |  |  |  |
| TM2D3 |  |  |  |  |  |  |  |
| SLC25A24 |  |  |  |  |  |  |  |
| ILDR1 |  |  |  |  |  |  |  |
| MTCO3P11 |  |  |  |  |  |  |  |
| MYLIP |  |  |  |  |  |  |  |
| ISLR |  |  |  |  |  |  |  |
| GYG2 |  |  |  |  |  |  |  |
| MPI |  |  |  |  |  |  |  |
| MRC1 |  |  |  |  |  |  |  |
| GABRR2 |  |  |  |  |  |  |  |
| TBX2 |  |  |  |  |  |  |  |
| WDR19 |  |  |  |  |  |  |  |
| TIE1 |  |  |  |  |  |  |  |
| ENSG00000278058 |  |  |  |  |  |  |  |
| CBX6 |  |  |  |  |  |  |  |
| PARD6G |  |  |  |  |  |  |  |
| TPD52L1 |  |  |  |  |  |  |  |
| TMEM123 |  |  |  |  |  |  |  |
| CCDC150 |  |  |  |  |  |  |  |
| MCF2L-AS1 |  |  |  |  |  |  |  |
| ADAMTSL4-AS1 |  |  |  |  |  |  |  |
| ENSG00000244398 |  |  |  |  |  |  |  |
| FAM76B |  |  |  |  |  |  |  |
| LDLRAD3 |  |  |  |  |  |  |  |
| MT-ND4L |  |  |  |  |  |  |  |
| EGOT |  |  |  |  |  |  |  |
| MIR3679 |  |  |  |  |  |  |  |
| IFITM4P |  |  |  |  |  |  |  |
| ADARB2 |  |  |  |  |  |  |  |
| C2orf72 |  |  |  |  |  |  |  |
| STXBP1 |  |  |  |  |  |  |  |
| IGHV4-59 |  |  |  |  |  |  |  |
| ZFP1 |  |  |  |  |  |  |  |
| MEGF10 |  |  |  |  |  |  |  |
| ENSG00000255026 |  |  |  |  |  |  |  |
| RN7SL674P |  |  |  |  |  |  |  |
| PYCARD |  |  |  |  |  |  |  |
| PTX3 |  |  |  |  |  |  |  |
| ENSG00000270072 |  |  |  |  |  |  |  |
| SYTL5 |  |  |  |  |  |  |  |
| COLEC12 |  |  |  |  |  |  |  |
| GPR155 |  |  |  |  |  |  |  |
| C22orf46 |  |  |  |  |  |  |  |
| TMEM17 |  |  |  |  |  |  |  |
| GPR162 |  |  |  |  |  |  |  |
| NBL1 |  |  |  |  |  |  |  |
| SLC26A8 |  |  |  |  |  |  |  |
| SFXN4 |  |  |  |  |  |  |  |
| WWTR1-AS1 |  |  |  |  |  |  |  |
| LINC02028 |  |  |  |  |  |  |  |
| MT-TE |  |  |  |  |  |  |  |
| BACE2-IT1 |  |  |  |  |  |  |  |
| TMC7 |  |  |  |  |  |  |  |
| GIGYF1 |  |  |  |  |  |  |  |
| LRRC28 |  |  |  |  |  |  |  |
| PCSK1 |  |  |  |  |  |  |  |
| SEC61B |  |  |  |  |  |  |  |
| DCN |  |  |  |  |  |  |  |
| CR1L |  |  |  |  |  |  |  |
| GUCY1A1 |  |  |  |  |  |  |  |
| ESRP1 |  |  |  |  |  |  |  |
| CACNB4 |  |  |  |  |  |  |  |
| FSCN1 |  |  |  |  |  |  |  |
| PRORSD1P |  |  |  |  |  |  |  |
| UBE2V1P1 |  |  |  |  |  |  |  |
| Y_RNA |  |  |  |  |  |  |  |
| PCYT2 |  |  |  |  |  |  |  |
| S100A16 |  |  |  |  |  |  |  |
| MRPL2 |  |  |  |  |  |  |  |
| CHST10 |  |  |  |  |  |  |  |
| ROMO1 |  |  |  |  |  |  |  |
| ANGPT1 |  |  |  |  |  |  |  |
| TEX41 |  |  |  |  |  |  |  |
| TSPAN6 |  |  |  |  |  |  |  |
| COL27A1 |  |  |  |  |  |  |  |
| MIA3 |  |  |  |  |  |  |  |
| ENSG00000233461 |  |  |  |  |  |  |  |
| ENSG00000288031 |  |  |  |  |  |  |  |
| MOCS2 |  |  |  |  |  |  |  |
| MGAT4B |  |  |  |  |  |  |  |
| ZMAT3 |  |  |  |  |  |  |  |
| CNTLN |  |  |  |  |  |  |  |
| PIGR |  |  |  |  |  |  |  |
| ENSG00000259392 |  |  |  |  |  |  |  |
| FAM210A |  |  |  |  |  |  |  |
| ENSG00000273724 |  |  |  |  |  |  |  |
| AOC3 |  |  |  |  |  |  |  |
| ACVR1C |  |  |  |  |  |  |  |
| LINC01169 |  |  |  |  |  |  |  |
| THSD1 |  |  |  |  |  |  |  |
| SPATA18 |  |  |  |  |  |  |  |
| ENSG00000276809 |  |  |  |  |  |  |  |
| AIF1L |  |  |  |  |  |  |  |
| RPL13AP25 |  |  |  |  |  |  |  |
| H6PD |  |  |  |  |  |  |  |
| COMMD8 |  |  |  |  |  |  |  |
| LRCH2 |  |  |  |  |  |  |  |
| TRAF1 |  |  |  |  |  |  |  |
| IQSEC2 |  |  |  |  |  |  |  |
| ACE |  |  |  |  |  |  |  |
| ENSG00000273165 |  |  |  |  |  |  |  |
| LRRC8E |  |  |  |  |  |  |  |
| ACOT11 |  |  |  |  |  |  |  |
| HMGA1 |  |  |  |  |  |  |  |
| ALDH1B1 |  |  |  |  |  |  |  |
| SSPN |  |  |  |  |  |  |  |
| C16orf91 |  |  |  |  |  |  |  |
| CYP4Z1 |  |  |  |  |  |  |  |
| MXI1 |  |  |  |  |  |  |  |
| RORA |  |  |  |  |  |  |  |
| ENSG00000288663 |  |  |  |  |  |  |  |
| CNTROB |  |  |  |  |  |  |  |
| PLA2G5 |  |  |  |  |  |  |  |
| MXD4 |  |  |  |  |  |  |  |
| ENSG00000272079 |  |  |  |  |  |  |  |
| ENSG00000261476 |  |  |  |  |  |  |  |
| ENSG00000270190 |  |  |  |  |  |  |  |
| RAB7B |  |  |  |  |  |  |  |
| SNTB1 |  |  |  |  |  |  |  |
| FXN |  |  |  |  |  |  |  |
| MSC-AS1 |  |  |  |  |  |  |  |
| PLA1A |  |  |  |  |  |  |  |
| RRN3P2 |  |  |  |  |  |  |  |
| PALM |  |  |  |  |  |  |  |
| ENSG00000260007 |  |  |  |  |  |  |  |
| HTRA3 |  |  |  |  |  |  |  |
| RAB40C |  |  |  |  |  |  |  |
| SEMA4F |  |  |  |  |  |  |  |
| ENSG00000269189 |  |  |  |  |  |  |  |
| SMIM5 |  |  |  |  |  |  |  |
| COX17 |  |  |  |  |  |  |  |
| HSPA9 |  |  |  |  |  |  |  |
| SH2D3C |  |  |  |  |  |  |  |
| NYNRIN |  |  |  |  |  |  |  |
| ENSG00000257181 |  |  |  |  |  |  |  |
| ECSCR |  |  |  |  |  |  |  |
| ITGB4 |  |  |  |  |  |  |  |
| LNP1 |  |  |  |  |  |  |  |
| ZNF580 |  |  |  |  |  |  |  |
| RPP40 |  |  |  |  |  |  |  |
| FLNA |  |  |  |  |  |  |  |
| ENSG00000256682 |  |  |  |  |  |  |  |
| FRS3 |  |  |  |  |  |  |  |
| CYP2G1P |  |  |  |  |  |  |  |
| LIMS2 |  |  |  |  |  |  |  |
| TMEM205 |  |  |  |  |  |  |  |
| RPS2P5 |  |  |  |  |  |  |  |
| ZBTB45 |  |  |  |  |  |  |  |
| ENSG00000219712 |  |  |  |  |  |  |  |
| TAS2R13 |  |  |  |  |  |  |  |
| KDELR3 |  |  |  |  |  |  |  |
| NDUFAB1 |  |  |  |  |  |  |  |
| IGLV3-1 |  |  |  |  |  |  |  |
| RPL23AP37 |  |  |  |  |  |  |  |
| ENSG00000271011 |  |  |  |  |  |  |  |
| F2RL1 |  |  |  |  |  |  |  |
| RNU7-124P |  |  |  |  |  |  |  |
| SEPHS2 |  |  |  |  |  |  |  |
| MACC1 |  |  |  |  |  |  |  |
| LTB4R |  |  |  |  |  |  |  |
| CPEB4 |  |  |  |  |  |  |  |
| GALNT2 |  |  |  |  |  |  |  |
| PREB |  |  |  |  |  |  |  |
| FGFBP3 |  |  |  |  |  |  |  |
| ACAD11 |  |  |  |  |  |  |  |
| CRISPLD1 |  |  |  |  |  |  |  |
| ENSG00000268707 |  |  |  |  |  |  |  |
| IQGAP1 |  |  |  |  |  |  |  |
| PECR |  |  |  |  |  |  |  |
| ZNF503 |  |  |  |  |  |  |  |
| RCAN1 |  |  |  |  |  |  |  |
| RN7SL57P |  |  |  |  |  |  |  |
| KSR2 |  |  |  |  |  |  |  |
| MIR3142HG |  |  |  |  |  |  |  |
| DCXR |  |  |  |  |  |  |  |
| SLC52A3 |  |  |  |  |  |  |  |
| SPACA9 |  |  |  |  |  |  |  |
| SLC39A10 |  |  |  |  |  |  |  |
| NBPF25P |  |  |  |  |  |  |  |
| ENSG00000261544 |  |  |  |  |  |  |  |
| ENSG00000284428 |  |  |  |  |  |  |  |
| PCDHGA2 |  |  |  |  |  |  |  |
| FAM13C |  |  |  |  |  |  |  |
| LIPH |  |  |  |  |  |  |  |
| E2F8 |  |  |  |  |  |  |  |
| CBFA2T3 |  |  |  |  |  |  |  |
| TGM2 |  |  |  |  |  |  |  |
| GIPR |  |  |  |  |  |  |  |
| LINC00702 |  |  |  |  |  |  |  |
| APOBEC3F |  |  |  |  |  |  |  |
| MALRD1 |  |  |  |  |  |  |  |
| ZNF724 |  |  |  |  |  |  |  |
| IGSF8 |  |  |  |  |  |  |  |
| SNORA22 |  |  |  |  |  |  |  |
| TRPV6 |  |  |  |  |  |  |  |
| RPL23A |  |  |  |  |  |  |  |
| ENSG00000217648 |  |  |  |  |  |  |  |
| NCMAP |  |  |  |  |  |  |  |
| IGHV4-39 |  |  |  |  |  |  |  |
| SOCS3 |  |  |  |  |  |  |  |
| CEBPG |  |  |  |  |  |  |  |
| PSTK |  |  |  |  |  |  |  |
| TMEM98 |  |  |  |  |  |  |  |
| HOMER1 |  |  |  |  |  |  |  |
| GTF2IP1 |  |  |  |  |  |  |  |
| FAM110A |  |  |  |  |  |  |  |
| RGCC |  |  |  |  |  |  |  |
| SLC27A4 |  |  |  |  |  |  |  |
| SPACA6 |  |  |  |  |  |  |  |
| ERG28 |  |  |  |  |  |  |  |
| RHOD |  |  |  |  |  |  |  |
| PPIAP30 |  |  |  |  |  |  |  |
| CD151 |  |  |  |  |  |  |  |
| SELENOP |  |  |  |  |  |  |  |
| GIMAP1 |  |  |  |  |  |  |  |
| EIF2S2P4 |  |  |  |  |  |  |  |
| MICE |  |  |  |  |  |  |  |
| NPM2 |  |  |  |  |  |  |  |
| MIR558 |  |  |  |  |  |  |  |
| ESCO2 |  |  |  |  |  |  |  |
| ENSG00000276603 |  |  |  |  |  |  |  |
| PRRT2 |  |  |  |  |  |  |  |
| TTLL1 |  |  |  |  |  |  |  |
| PLEKHG5 |  |  |  |  |  |  |  |
| SCART1 |  |  |  |  |  |  |  |
| ENSG00000278434 |  |  |  |  |  |  |  |
| GCLM |  |  |  |  |  |  |  |
| ENSG00000266371 |  |  |  |  |  |  |  |
| COTL1 |  |  |  |  |  |  |  |
| ST8SIA5 |  |  |  |  |  |  |  |
| RPS4X |  |  |  |  |  |  |  |
| SLC15A2 |  |  |  |  |  |  |  |
| PLCL1 |  |  |  |  |  |  |  |
| RPS6KL1 |  |  |  |  |  |  |  |
| ZFTA |  |  |  |  |  |  |  |
| PWRN1 |  |  |  |  |  |  |  |
| SLC25A37 |  |  |  |  |  |  |  |
| SCD |  |  |  |  |  |  |  |
| PRDX6 |  |  |  |  |  |  |  |
| TCEA1P4 |  |  |  |  |  |  |  |
| ARL5B |  |  |  |  |  |  |  |
| GPRC5C |  |  |  |  |  |  |  |
| TOB1-AS1 |  |  |  |  |  |  |  |
| H4C1 |  |  |  |  |  |  |  |
| PID1 |  |  |  |  |  |  |  |
| MYO16 |  |  |  |  |  |  |  |
| PEG10 |  |  |  |  |  |  |  |
| RNH1 |  |  |  |  |  |  |  |
| MRPL32 |  |  |  |  |  |  |  |
| RPL9 |  |  |  |  |  |  |  |
| SPA17 |  |  |  |  |  |  |  |
| ABCG1 |  |  |  |  |  |  |  |
| SGSM1 |  |  |  |  |  |  |  |
| IYD |  |  |  |  |  |  |  |
| KLHL29 |  |  |  |  |  |  |  |
| GMFG |  |  |  |  |  |  |  |
| MCM5 |  |  |  |  |  |  |  |
| FRZB |  |  |  |  |  |  |  |
| CADPS |  |  |  |  |  |  |  |
| ADAM19 |  |  |  |  |  |  |  |
| VIM |  |  |  |  |  |  |  |
| ARHGAP23 |  |  |  |  |  |  |  |
| ENSG00000259407 |  |  |  |  |  |  |  |
| CAVIN4 |  |  |  |  |  |  |  |
| RPL13P5 |  |  |  |  |  |  |  |
| RN7SL809P |  |  |  |  |  |  |  |
| CCDC106 |  |  |  |  |  |  |  |
| ADCK2 |  |  |  |  |  |  |  |
| SEMA4C |  |  |  |  |  |  |  |
| ENSG00000273259 |  |  |  |  |  |  |  |
| ITGA3 |  |  |  |  |  |  |  |
| MIR6081 |  |  |  |  |  |  |  |
| RNU6-759P |  |  |  |  |  |  |  |
| WHAMMP1 |  |  |  |  |  |  |  |
| ENSG00000280351 |  |  |  |  |  |  |  |
| ZNF777 |  |  |  |  |  |  |  |
| TMEM220 |  |  |  |  |  |  |  |
| PLCB3 |  |  |  |  |  |  |  |
| CD300LB |  |  |  |  |  |  |  |
| MAGEE1 |  |  |  |  |  |  |  |
| ECI2 |  |  |  |  |  |  |  |
| MGAM |  |  |  |  |  |  |  |
| ADTRP |  |  |  |  |  |  |  |
| LSP1 |  |  |  |  |  |  |  |
| IGHV3-30 |  |  |  |  |  |  |  |
| RFPL3S |  |  |  |  |  |  |  |
| TEX30 |  |  |  |  |  |  |  |
| ENSG00000257531 |  |  |  |  |  |  |  |
| CYP2J2 |  |  |  |  |  |  |  |
| GALT |  |  |  |  |  |  |  |
| FGGY |  |  |  |  |  |  |  |
| PCOLCE2 |  |  |  |  |  |  |  |
| NRGN |  |  |  |  |  |  |  |
| ENSG00000261291 |  |  |  |  |  |  |  |
| ENSG00000280604 |  |  |  |  |  |  |  |
| THBS1-IT1 |  |  |  |  |  |  |  |
| DNAJA3 |  |  |  |  |  |  |  |
| UBE2Q2 |  |  |  |  |  |  |  |
| CD320 |  |  |  |  |  |  |  |
| HIBADH |  |  |  |  |  |  |  |
| RTTN |  |  |  |  |  |  |  |
| WWP1 |  |  |  |  |  |  |  |
| INSC |  |  |  |  |  |  |  |
| RASD1 |  |  |  |  |  |  |  |
| DOK2 |  |  |  |  |  |  |  |
| MPRIPP1 |  |  |  |  |  |  |  |
| CCL21 |  |  |  |  |  |  |  |
| ENSG00000270091 |  |  |  |  |  |  |  |
| ABCC4 |  |  |  |  |  |  |  |
| ANXA2P2 |  |  |  |  |  |  |  |
| TRMT11 |  |  |  |  |  |  |  |
| CCDC17 |  |  |  |  |  |  |  |
| RAD52 |  |  |  |  |  |  |  |
| KCNS3 |  |  |  |  |  |  |  |
| TMEM273 |  |  |  |  |  |  |  |
| ENSG00000275850 |  |  |  |  |  |  |  |
| EMP1 |  |  |  |  |  |  |  |
| RCC2 |  |  |  |  |  |  |  |
| ZNF670 |  |  |  |  |  |  |  |
| TAS2R63P |  |  |  |  |  |  |  |
| HMGA2 |  |  |  |  |  |  |  |
| SCARA3 |  |  |  |  |  |  |  |
| ASPG |  |  |  |  |  |  |  |
| RAB6B |  |  |  |  |  |  |  |
| YBX3 |  |  |  |  |  |  |  |
| ENSG00000274038 |  |  |  |  |  |  |  |
| SLCO2B1 |  |  |  |  |  |  |  |
| PHETA2 |  |  |  |  |  |  |  |
| ARID3A |  |  |  |  |  |  |  |
| MDH2 |  |  |  |  |  |  |  |
| RPL7AP42 |  |  |  |  |  |  |  |
| SHTN1 |  |  |  |  |  |  |  |
| SULT1C4 |  |  |  |  |  |  |  |
| RPL7P9 |  |  |  |  |  |  |  |
| ENSG00000286689 |  |  |  |  |  |  |  |
| TBC1D30 |  |  |  |  |  |  |  |
| GIT1 |  |  |  |  |  |  |  |
| USF1P1 |  |  |  |  |  |  |  |
| ENSG00000288586 |  |  |  |  |  |  |  |
| TMEM26 |  |  |  |  |  |  |  |
| LARGE-IT1 |  |  |  |  |  |  |  |
| ENSG00000251314 |  |  |  |  |  |  |  |
| ENSG00000261056 |  |  |  |  |  |  |  |
| RN7SKP110 |  |  |  |  |  |  |  |
| ENSG00000273328 |  |  |  |  |  |  |  |
| VWA8 |  |  |  |  |  |  |  |
| CDCA4 |  |  |  |  |  |  |  |
| GADD45B |  |  |  |  |  |  |  |
| ENSG00000279453 |  |  |  |  |  |  |  |
| LINC01232 |  |  |  |  |  |  |  |
| WFIKKN1 |  |  |  |  |  |  |  |
| ZNF703 |  |  |  |  |  |  |  |
| DNM1P47 |  |  |  |  |  |  |  |
| TCIM |  |  |  |  |  |  |  |
| GPRC5A |  |  |  |  |  |  |  |
| NPNT |  |  |  |  |  |  |  |
| SIRPA |  |  |  |  |  |  |  |
| ADAMTSL4-AS2 |  |  |  |  |  |  |  |
| CNPY4 |  |  |  |  |  |  |  |
| NOVA2 |  |  |  |  |  |  |  |
| ZNF711 |  |  |  |  |  |  |  |
| MXD1 |  |  |  |  |  |  |  |
| CATSPERG |  |  |  |  |  |  |  |
| ENSG00000274776 |  |  |  |  |  |  |  |
| NDUFB2-AS1 |  |  |  |  |  |  |  |
| PIP5K1B |  |  |  |  |  |  |  |
| BHLHE41 |  |  |  |  |  |  |  |
| ZFP82 |  |  |  |  |  |  |  |
| PEX3 |  |  |  |  |  |  |  |
| ENSG00000261888 |  |  |  |  |  |  |  |
| ENSG00000272505 |  |  |  |  |  |  |  |
| MMUT |  |  |  |  |  |  |  |
| ZNF579 |  |  |  |  |  |  |  |
| NRN1 |  |  |  |  |  |  |  |
| SCIN |  |  |  |  |  |  |  |
| ZNF252P-AS1 |  |  |  |  |  |  |  |
| SMG1P7 |  |  |  |  |  |  |  |
| ANXA2 |  |  |  |  |  |  |  |
| CACTIN |  |  |  |  |  |  |  |
| NBR2 |  |  |  |  |  |  |  |
| CRB3 |  |  |  |  |  |  |  |
| AP1G2-AS1 |  |  |  |  |  |  |  |
| B3GNT7 |  |  |  |  |  |  |  |
| NKD2 |  |  |  |  |  |  |  |
| GNA12 |  |  |  |  |  |  |  |
| PDGFB |  |  |  |  |  |  |  |
| ST7-OT4 |  |  |  |  |  |  |  |
| DBF4 |  |  |  |  |  |  |  |
| FBLN2 |  |  |  |  |  |  |  |
| EXPH5 |  |  |  |  |  |  |  |
| ORMDL3 |  |  |  |  |  |  |  |
| ARHGAP6 |  |  |  |  |  |  |  |
| ENSG00000253372 |  |  |  |  |  |  |  |
| ECHS1 |  |  |  |  |  |  |  |
| PRR4 |  |  |  |  |  |  |  |
| TNFSF14 |  |  |  |  |  |  |  |
| GSTP1 |  |  |  |  |  |  |  |
| ENSG00000272668 |  |  |  |  |  |  |  |
| ENSG00000287925 |  |  |  |  |  |  |  |
| BAIAP2-DT |  |  |  |  |  |  |  |
| ATP5F1C |  |  |  |  |  |  |  |
| USP32P3 |  |  |  |  |  |  |  |
| TNKS1BP1 |  |  |  |  |  |  |  |
| KMT5C |  |  |  |  |  |  |  |
| FAM135B |  |  |  |  |  |  |  |
| NECAB3 |  |  |  |  |  |  |  |
| MAFG-DT |  |  |  |  |  |  |  |
| HDHD5 |  |  |  |  |  |  |  |
| ST3GAL6-AS1 |  |  |  |  |  |  |  |
| ENSG00000288538 |  |  |  |  |  |  |  |
| ENSG00000228280 |  |  |  |  |  |  |  |
| TADA1 |  |  |  |  |  |  |  |
| ZNF385B |  |  |  |  |  |  |  |
| ENSG00000277687 |  |  |  |  |  |  |  |
| SNX21 |  |  |  |  |  |  |  |
| ENSG00000224945 |  |  |  |  |  |  |  |
| ENSG00000234902 |  |  |  |  |  |  |  |
| KLF5 |  |  |  |  |  |  |  |
| MB21D2 |  |  |  |  |  |  |  |
| PFDN2 |  |  |  |  |  |  |  |
| RN7SKP56 |  |  |  |  |  |  |  |
| ENSG00000285744 |  |  |  |  |  |  |  |
| TMEM254 |  |  |  |  |  |  |  |
| FAM151B |  |  |  |  |  |  |  |
| ENSG00000279026 |  |  |  |  |  |  |  |
| ENSG00000259767 |  |  |  |  |  |  |  |
| RNF213-AS1 |  |  |  |  |  |  |  |
| ENSG00000281469 |  |  |  |  |  |  |  |
| FAM78B |  |  |  |  |  |  |  |
| LGR4 |  |  |  |  |  |  |  |
| MAK |  |  |  |  |  |  |  |
| ENSG00000255872 |  |  |  |  |  |  |  |
| KCNJ15 |  |  |  |  |  |  |  |
| ENSG00000280046 |  |  |  |  |  |  |  |
| ZNF865 |  |  |  |  |  |  |  |
| ATOH8 |  |  |  |  |  |  |  |
| FOXQ1 |  |  |  |  |  |  |  |
| ENSG00000270061 |  |  |  |  |  |  |  |
| SCRIB |  |  |  |  |  |  |  |
| ANKRD33B |  |  |  |  |  |  |  |
| LPL |  |  |  |  |  |  |  |
| SLC45A4 |  |  |  |  |  |  |  |
| LRCOL1 |  |  |  |  |  |  |  |
| CPT2 |  |  |  |  |  |  |  |
| MYCBP2-AS2 |  |  |  |  |  |  |  |
| EFS |  |  |  |  |  |  |  |
| ENSG00000275120 |  |  |  |  |  |  |  |
| ZNF331 |  |  |  |  |  |  |  |
| FLVCR2 |  |  |  |  |  |  |  |
| RPLP2 |  |  |  |  |  |  |  |
| BMP5 |  |  |  |  |  |  |  |
| B4GALNT3 |  |  |  |  |  |  |  |
| PROCR |  |  |  |  |  |  |  |
| NIPAL1 |  |  |  |  |  |  |  |
| PAQR7 |  |  |  |  |  |  |  |
| ENSG00000260495 |  |  |  |  |  |  |  |
| ENSG00000273133 |  |  |  |  |  |  |  |
| TG |  |  |  |  |  |  |  |
| ENSG00000277595 |  |  |  |  |  |  |  |
| KIAA1614 |  |  |  |  |  |  |  |
| CIART |  |  |  |  |  |  |  |
| CCDC180 |  |  |  |  |  |  |  |
| GOLGA2P10 |  |  |  |  |  |  |  |
| KCNT2 |  |  |  |  |  |  |  |
| ZNF707 |  |  |  |  |  |  |  |
| TPTEP1 |  |  |  |  |  |  |  |
| JOSD2 |  |  |  |  |  |  |  |
| ENSG00000233674 |  |  |  |  |  |  |  |
| SH3BGRL |  |  |  |  |  |  |  |
| CR1 |  |  |  |  |  |  |  |
| GPSM1 |  |  |  |  |  |  |  |
| MRPL54 |  |  |  |  |  |  |  |
| CNN1 |  |  |  |  |  |  |  |
| MCRIP1 |  |  |  |  |  |  |  |
| ROPN1L |  |  |  |  |  |  |  |
| SMAD6 |  |  |  |  |  |  |  |
| COX6A1 |  |  |  |  |  |  |  |
| IFI27L2 |  |  |  |  |  |  |  |
| ENSG00000225721 |  |  |  |  |  |  |  |
| SALL2 |  |  |  |  |  |  |  |
| TMED1 |  |  |  |  |  |  |  |
| SCML2 |  |  |  |  |  |  |  |
| ENSG00000279811 |  |  |  |  |  |  |  |
| MARCHF9 |  |  |  |  |  |  |  |
| ACADS |  |  |  |  |  |  |  |
| SUCLG2-DT |  |  |  |  |  |  |  |
| MOK |  |  |  |  |  |  |  |
| ENSG00000275236 |  |  |  |  |  |  |  |
| UBXN8 |  |  |  |  |  |  |  |
| ENSG00000278367 |  |  |  |  |  |  |  |
| PRDX3 |  |  |  |  |  |  |  |
| BAALC-AS1 |  |  |  |  |  |  |  |
| INTS6-AS1 |  |  |  |  |  |  |  |
| ENSG00000261537 |  |  |  |  |  |  |  |
| SDCBP2 |  |  |  |  |  |  |  |
| ENSG00000287562 |  |  |  |  |  |  |  |
| GAPDH |  |  |  |  |  |  |  |
| STAP2 |  |  |  |  |  |  |  |
| ENSG00000273381 |  |  |  |  |  |  |  |
| EFEMP1 |  |  |  |  |  |  |  |
| ENSG00000229955 |  |  |  |  |  |  |  |
| RTN2 |  |  |  |  |  |  |  |
| KLHL32 |  |  |  |  |  |  |  |
| TWF2 |  |  |  |  |  |  |  |
| TIFA |  |  |  |  |  |  |  |
| APOE |  |  |  |  |  |  |  |
| ETFB |  |  |  |  |  |  |  |
| ENSG00000223916 |  |  |  |  |  |  |  |
| FOXF1 |  |  |  |  |  |  |  |
| RDH10 |  |  |  |  |  |  |  |
| IGSF6 |  |  |  |  |  |  |  |
| PLCD1 |  |  |  |  |  |  |  |
| ENSG00000250519 |  |  |  |  |  |  |  |
| ENSG00000284292 |  |  |  |  |  |  |  |
| NPIPP1 |  |  |  |  |  |  |  |
| AQP7 |  |  |  |  |  |  |  |
| CEP57 |  |  |  |  |  |  |  |
| NKILA |  |  |  |  |  |  |  |
| RASEF |  |  |  |  |  |  |  |
| RTN4RL1 |  |  |  |  |  |  |  |
| OSTC |  |  |  |  |  |  |  |
| ENSG00000272379 |  |  |  |  |  |  |  |
| AGPAT5 |  |  |  |  |  |  |  |
| NSDHL |  |  |  |  |  |  |  |
| ENSG00000270804 |  |  |  |  |  |  |  |
| UST |  |  |  |  |  |  |  |
| IPMK |  |  |  |  |  |  |  |
| ENSG00000286817 |  |  |  |  |  |  |  |
| ENSG00000224593 |  |  |  |  |  |  |  |
| FBLN1 |  |  |  |  |  |  |  |
| MRPS35 |  |  |  |  |  |  |  |
| ENSG00000240710 |  |  |  |  |  |  |  |
| IL17RD |  |  |  |  |  |  |  |
| RAMP2-AS1 |  |  |  |  |  |  |  |
| ENSG00000261451 |  |  |  |  |  |  |  |
| NUDCP1 |  |  |  |  |  |  |  |
| NCK2 |  |  |  |  |  |  |  |
| C21orf58 |  |  |  |  |  |  |  |
| GSTZ1 |  |  |  |  |  |  |  |
| CCDC88B |  |  |  |  |  |  |  |
| CCL19 |  |  |  |  |  |  |  |
| HILPDA |  |  |  |  |  |  |  |
| CASQ2 |  |  |  |  |  |  |  |
| DKK2 |  |  |  |  |  |  |  |
| DNMT1 |  |  |  |  |  |  |  |
| LMOD3 |  |  |  |  |  |  |  |
| EEPD1 |  |  |  |  |  |  |  |
| ENSG00000279059 |  |  |  |  |  |  |  |
| DDX10P1 |  |  |  |  |  |  |  |
| HIC1 |  |  |  |  |  |  |  |
| ENSG00000286451 |  |  |  |  |  |  |  |
| RGS14 |  |  |  |  |  |  |  |
| CYC1 |  |  |  |  |  |  |  |
| RABGAP1L-DT |  |  |  |  |  |  |  |
| DERA |  |  |  |  |  |  |  |
| ENSG00000266498 |  |  |  |  |  |  |  |
| ENSG00000237094 |  |  |  |  |  |  |  |
| FBXL19 |  |  |  |  |  |  |  |
| TRAPPC4 |  |  |  |  |  |  |  |
| PRR15L |  |  |  |  |  |  |  |
| AP4B1 |  |  |  |  |  |  |  |
| ZEB2-AS1 |  |  |  |  |  |  |  |
| LRRC8D |  |  |  |  |  |  |  |
| ELAPOR2 |  |  |  |  |  |  |  |
| PCF11-AS1 |  |  |  |  |  |  |  |
| RPL30P13 |  |  |  |  |  |  |  |
| ENSG00000274297 |  |  |  |  |  |  |  |
| ENSG00000272033 |  |  |  |  |  |  |  |
| MALL |  |  |  |  |  |  |  |
| CFP |  |  |  |  |  |  |  |
| GRB7 |  |  |  |  |  |  |  |
| MST1L |  |  |  |  |  |  |  |
| BTNL9 |  |  |  |  |  |  |  |
| PCDHB4 |  |  |  |  |  |  |  |
| PLA2G15 |  |  |  |  |  |  |  |
| BCL2L11 |  |  |  |  |  |  |  |
| ADGRG6 |  |  |  |  |  |  |  |
| RAI2 |  |  |  |  |  |  |  |
| KYAT3 |  |  |  |  |  |  |  |
| DIP2A-IT1 |  |  |  |  |  |  |  |
| SYN1 |  |  |  |  |  |  |  |
| GALNT3 |  |  |  |  |  |  |  |
| PTPRU |  |  |  |  |  |  |  |
| ENSG00000229618 |  |  |  |  |  |  |  |
| ZNF524 |  |  |  |  |  |  |  |
| QSOX1 |  |  |  |  |  |  |  |
| YTHDF1P1 |  |  |  |  |  |  |  |
| NANOGP8 |  |  |  |  |  |  |  |
| LINC02918 |  |  |  |  |  |  |  |
| ENSG00000273151 |  |  |  |  |  |  |  |
| PRSS36 |  |  |  |  |  |  |  |
| ARL4AP5 |  |  |  |  |  |  |  |
| ENSG00000258311 |  |  |  |  |  |  |  |
| NT5E |  |  |  |  |  |  |  |
| AP1M2 |  |  |  |  |  |  |  |
| ANGPTL1 |  |  |  |  |  |  |  |
| DPYSL3 |  |  |  |  |  |  |  |
| FUOM |  |  |  |  |  |  |  |
| PKNOX2 |  |  |  |  |  |  |  |
| NCAPGP1 |  |  |  |  |  |  |  |
| PRSS42P |  |  |  |  |  |  |  |
| ENSG00000250899 |  |  |  |  |  |  |  |
| INKA2 |  |  |  |  |  |  |  |
| GIMAP8 |  |  |  |  |  |  |  |
| FEZ1 |  |  |  |  |  |  |  |
| ENSG00000260645 |  |  |  |  |  |  |  |
| ATN1 |  |  |  |  |  |  |  |
| HMOX1 |  |  |  |  |  |  |  |
| TRIM59 |  |  |  |  |  |  |  |
| MMACHC |  |  |  |  |  |  |  |
| DZIP1L |  |  |  |  |  |  |  |
| ENSG00000256361 |  |  |  |  |  |  |  |
| INPP5J |  |  |  |  |  |  |  |
| CHMP4A |  |  |  |  |  |  |  |
| MRPL39 |  |  |  |  |  |  |  |
| FSBP |  |  |  |  |  |  |  |
| SLC28A3 |  |  |  |  |  |  |  |
| ENSG00000233178 |  |  |  |  |  |  |  |
| SOWAHCP2 |  |  |  |  |  |  |  |
| FRMD5 |  |  |  |  |  |  |  |
| FBXW9 |  |  |  |  |  |  |  |
| C1QTNF3 |  |  |  |  |  |  |  |
| ZNF213 |  |  |  |  |  |  |  |
| NOTCH4 |  |  |  |  |  |  |  |
| WHAMMP4 |  |  |  |  |  |  |  |
| IGHV2-5 |  |  |  |  |  |  |  |
| BMP4 |  |  |  |  |  |  |  |
| PARP10 |  |  |  |  |  |  |  |
| B3GAT1 |  |  |  |  |  |  |  |
| RPL7 |  |  |  |  |  |  |  |
| VWA7 |  |  |  |  |  |  |  |
| S100A6 |  |  |  |  |  |  |  |
| ENSG00000270956 |  |  |  |  |  |  |  |
| MSANTD1 |  |  |  |  |  |  |  |
| MECOM |  |  |  |  |  |  |  |
| PCDHB3 |  |  |  |  |  |  |  |
| TCEAL9 |  |  |  |  |  |  |  |
| CPNE5 |  |  |  |  |  |  |  |
| CARS1 |  |  |  |  |  |  |  |
| LINC02482 |  |  |  |  |  |  |  |
| SLC25A36 |  |  |  |  |  |  |  |
| PPM1L |  |  |  |  |  |  |  |
| LGALS3 |  |  |  |  |  |  |  |
| ENSG00000277152 |  |  |  |  |  |  |  |
| LTBP2 |  |  |  |  |  |  |  |
| RPS6P25 |  |  |  |  |  |  |  |
| ENSG00000272465 |  |  |  |  |  |  |  |
| LEPR |  |  |  |  |  |  |  |
| CACNA2D2 |  |  |  |  |  |  |  |
| ENSG00000265625 |  |  |  |  |  |  |  |
| RENBP |  |  |  |  |  |  |  |
| ENSG00000260816 |  |  |  |  |  |  |  |
| SH2D3A |  |  |  |  |  |  |  |
| ENSG00000271009 |  |  |  |  |  |  |  |
| ENSG00000261499 |  |  |  |  |  |  |  |
| MTND4P14 |  |  |  |  |  |  |  |
| ATP13A4 |  |  |  |  |  |  |  |
| UQCRQ |  |  |  |  |  |  |  |
| HOTAIRM1 |  |  |  |  |  |  |  |
| ETS2 |  |  |  |  |  |  |  |
| ENSG00000259915 |  |  |  |  |  |  |  |
| MT-TW |  |  |  |  |  |  |  |
| ENSG00000273437 |  |  |  |  |  |  |  |
| SHLD3 |  |  |  |  |  |  |  |
| C16orf54 |  |  |  |  |  |  |  |
| GNA13 |  |  |  |  |  |  |  |
| ENSG00000260160 |  |  |  |  |  |  |  |
| PEX11A |  |  |  |  |  |  |  |
| METRN |  |  |  |  |  |  |  |
| AK7 |  |  |  |  |  |  |  |
| ENSG00000254165 |  |  |  |  |  |  |  |
| PPP1R16B |  |  |  |  |  |  |  |
| ENSG00000203647 |  |  |  |  |  |  |  |
| ARHGEF17 |  |  |  |  |  |  |  |
| ENSG00000273448 |  |  |  |  |  |  |  |
| ENSG00000232528 |  |  |  |  |  |  |  |
| PPIAP2 |  |  |  |  |  |  |  |
| ENSG00000273230 |  |  |  |  |  |  |  |
| ENSG00000218426 |  |  |  |  |  |  |  |
| MEX3B |  |  |  |  |  |  |  |
| ITGB7 |  |  |  |  |  |  |  |
| BCAT2 |  |  |  |  |  |  |  |
| KDM5C-IT1 |  |  |  |  |  |  |  |
| ENSG00000273893 |  |  |  |  |  |  |  |
| RPL5P30 |  |  |  |  |  |  |  |
| CDC14B |  |  |  |  |  |  |  |
| ZNHIT2 |  |  |  |  |  |  |  |
| LSMEM1 |  |  |  |  |  |  |  |
| HYAL1 |  |  |  |  |  |  |  |
| CFAP61 |  |  |  |  |  |  |  |
| HACD1 |  |  |  |  |  |  |  |
| ENSG00000281530 |  |  |  |  |  |  |  |
| GRHL2 |  |  |  |  |  |  |  |
| KIF3C |  |  |  |  |  |  |  |
| PKHD1L1 |  |  |  |  |  |  |  |
| ENSG00000228463 |  |  |  |  |  |  |  |
| FILIP1 |  |  |  |  |  |  |  |
| ZNF747-DT |  |  |  |  |  |  |  |
| TWF1 |  |  |  |  |  |  |  |
| LNX1 |  |  |  |  |  |  |  |
| ATP5MC3 |  |  |  |  |  |  |  |
| ARHGEF15 |  |  |  |  |  |  |  |
| BMPR1B |  |  |  |  |  |  |  |
| GIPC2 |  |  |  |  |  |  |  |
| MIR17HG |  |  |  |  |  |  |  |
| ATP5PBP1 |  |  |  |  |  |  |  |
| MT-ND6 |  |  |  |  |  |  |  |
| ACKR3 |  |  |  |  |  |  |  |
| DCLRE1A |  |  |  |  |  |  |  |
| PGK1 |  |  |  |  |  |  |  |
| SQLE |  |  |  |  |  |  |  |
| SPTBN5 |  |  |  |  |  |  |  |
| WNT2 |  |  |  |  |  |  |  |
| ARG2 |  |  |  |  |  |  |  |
| LINC02969 |  |  |  |  |  |  |  |
| ENSG00000273792 |  |  |  |  |  |  |  |
| PPM1F |  |  |  |  |  |  |  |
| ENSG00000272529 |  |  |  |  |  |  |  |
| ENSG00000276997 |  |  |  |  |  |  |  |
| TPMT |  |  |  |  |  |  |  |
| KDM8 |  |  |  |  |  |  |  |
| SLC36A1 |  |  |  |  |  |  |  |
| RASL12 |  |  |  |  |  |  |  |
| IQANK1 |  |  |  |  |  |  |  |
| SORCS2 |  |  |  |  |  |  |  |
| EEF1E1 |  |  |  |  |  |  |  |
| HK1 |  |  |  |  |  |  |  |
| GAPDHP1 |  |  |  |  |  |  |  |
| PNPLA6 |  |  |  |  |  |  |  |
| RAB13 |  |  |  |  |  |  |  |
| GRM8 |  |  |  |  |  |  |  |
| RPS23 |  |  |  |  |  |  |  |
| ANKRD1 |  |  |  |  |  |  |  |
| SLC12A7 |  |  |  |  |  |  |  |
| GNPNAT1 |  |  |  |  |  |  |  |
| SUGT1P1 |  |  |  |  |  |  |  |
| CHRNB1 |  |  |  |  |  |  |  |
| ENSG00000224950 |  |  |  |  |  |  |  |
| ENSG00000279348 |  |  |  |  |  |  |  |
| RAB3D |  |  |  |  |  |  |  |
| C18orf15 |  |  |  |  |  |  |  |
| LMOD1 |  |  |  |  |  |  |  |
| ADAMTS9 |  |  |  |  |  |  |  |
| TRPV2 |  |  |  |  |  |  |  |
| TTBK1 |  |  |  |  |  |  |  |
| GLCE |  |  |  |  |  |  |  |
| HSPE1 |  |  |  |  |  |  |  |
| GLDC |  |  |  |  |  |  |  |
| EPHB3 |  |  |  |  |  |  |  |
| CHPT1 |  |  |  |  |  |  |  |
| ADGRE5 |  |  |  |  |  |  |  |
| ITGA11 |  |  |  |  |  |  |  |
| ASPSCR1 |  |  |  |  |  |  |  |
| MAP4K2 |  |  |  |  |  |  |  |
| ENSG00000183171 |  |  |  |  |  |  |  |
| ART4 |  |  |  |  |  |  |  |
| ENSG00000238039 |  |  |  |  |  |  |  |
| SOX4 |  |  |  |  |  |  |  |
| B3GAT1-DT |  |  |  |  |  |  |  |
| PLN |  |  |  |  |  |  |  |
| TUBB6 |  |  |  |  |  |  |  |
| ADIRF-AS1 |  |  |  |  |  |  |  |
| PCDHAC2 |  |  |  |  |  |  |  |
| MAFK |  |  |  |  |  |  |  |
| STRIP2 |  |  |  |  |  |  |  |
| ENSG00000261582 |  |  |  |  |  |  |  |
| H2AC15 |  |  |  |  |  |  |  |
| TMPRSS4 |  |  |  |  |  |  |  |
| B4GALNT1 |  |  |  |  |  |  |  |
| ENSG00000226180 |  |  |  |  |  |  |  |
| ABCG2 |  |  |  |  |  |  |  |
| TAS2R15P |  |  |  |  |  |  |  |
| MAGOH2P |  |  |  |  |  |  |  |
| REM2 |  |  |  |  |  |  |  |
| KITLG |  |  |  |  |  |  |  |
| CXCL14 |  |  |  |  |  |  |  |
| PEBP1 |  |  |  |  |  |  |  |
| HSPB1 |  |  |  |  |  |  |  |
| SOD3 |  |  |  |  |  |  |  |
| TNFRSF19 |  |  |  |  |  |  |  |
| TPM4 |  |  |  |  |  |  |  |
| LILRB5 |  |  |  |  |  |  |  |
| ADH1B |  |  |  |  |  |  |  |
| PCED1B |  |  |  |  |  |  |  |
| ENSG00000262558 |  |  |  |  |  |  |  |
| N4BP3 |  |  |  |  |  |  |  |
| UQCR10 |  |  |  |  |  |  |  |
| PHLDB2 |  |  |  |  |  |  |  |
| ACOX1 |  |  |  |  |  |  |  |
| TARS1 |  |  |  |  |  |  |  |
| DEPTOR |  |  |  |  |  |  |  |
| KLF8 |  |  |  |  |  |  |  |
| ENSG00000268051 |  |  |  |  |  |  |  |
| DRAM1 |  |  |  |  |  |  |  |
| LINC02193 |  |  |  |  |  |  |  |
| TAGAP |  |  |  |  |  |  |  |
| PLEK2 |  |  |  |  |  |  |  |
| MCOLN3 |  |  |  |  |  |  |  |
| ADCY10P1 |  |  |  |  |  |  |  |
| P3H4 |  |  |  |  |  |  |  |
| PNMA2 |  |  |  |  |  |  |  |
| C11orf54 |  |  |  |  |  |  |  |
| CYGB |  |  |  |  |  |  |  |
| ENSG00000270140 |  |  |  |  |  |  |  |
| ARHGEF3 |  |  |  |  |  |  |  |
| ANKDD1B |  |  |  |  |  |  |  |
| THOC6 |  |  |  |  |  |  |  |
| PPP1R3G |  |  |  |  |  |  |  |
| ENSG00000254343 |  |  |  |  |  |  |  |
| CLPX |  |  |  |  |  |  |  |
| ZNF860 |  |  |  |  |  |  |  |
| CERS3-AS1 |  |  |  |  |  |  |  |
| RN7SKP30 |  |  |  |  |  |  |  |
| CDHR1 |  |  |  |  |  |  |  |
| SDHD |  |  |  |  |  |  |  |
| TIMP4 |  |  |  |  |  |  |  |
| HRC |  |  |  |  |  |  |  |
| MARCHF3 |  |  |  |  |  |  |  |
| CCDC183 |  |  |  |  |  |  |  |
| TMEM70 |  |  |  |  |  |  |  |
| IZUMO1 |  |  |  |  |  |  |  |
| LTK |  |  |  |  |  |  |  |
| ENSG00000267672 |  |  |  |  |  |  |  |
| KBTBD11 |  |  |  |  |  |  |  |
| CFAP410 |  |  |  |  |  |  |  |
| VASP |  |  |  |  |  |  |  |
| MIR570 |  |  |  |  |  |  |  |
| SLC25A20 |  |  |  |  |  |  |  |
| GUCY1A2 |  |  |  |  |  |  |  |
| EBP |  |  |  |  |  |  |  |
| BPNT2 |  |  |  |  |  |  |  |
| REX1BD |  |  |  |  |  |  |  |
| CALCB |  |  |  |  |  |  |  |
| ENSG00000262652 |  |  |  |  |  |  |  |
| ENSG00000248015 |  |  |  |  |  |  |  |
| ZNF90 |  |  |  |  |  |  |  |
| TLR2 |  |  |  |  |  |  |  |
| DNAH17 |  |  |  |  |  |  |  |
| DCST2 |  |  |  |  |  |  |  |
| ENSG00000234389 |  |  |  |  |  |  |  |
| TSLP |  |  |  |  |  |  |  |
| SYDE1 |  |  |  |  |  |  |  |
| ENPP2 |  |  |  |  |  |  |  |
| PLCH2 |  |  |  |  |  |  |  |
| RNU7-40P |  |  |  |  |  |  |  |
| SYT15-AS1 |  |  |  |  |  |  |  |
| PRSS8 |  |  |  |  |  |  |  |
| ENSG00000263120 |  |  |  |  |  |  |  |
| FAM83G |  |  |  |  |  |  |  |
| STPG3-AS1 |  |  |  |  |  |  |  |
| ADI1 |  |  |  |  |  |  |  |
| GASK1B-AS1 |  |  |  |  |  |  |  |
| ENSG00000274341 |  |  |  |  |  |  |  |
| IFI35 |  |  |  |  |  |  |  |
| HTR4 |  |  |  |  |  |  |  |
| ENSG00000277879 |  |  |  |  |  |  |  |
| ENSG00000259605 |  |  |  |  |  |  |  |
| MIR7161 |  |  |  |  |  |  |  |
| FENDRR |  |  |  |  |  |  |  |
| ENSG00000285417 |  |  |  |  |  |  |  |
| IRAK3 |  |  |  |  |  |  |  |
| PDE3A |  |  |  |  |  |  |  |
| CD34 |  |  |  |  |  |  |  |
| ENSG00000279880 |  |  |  |  |  |  |  |
| ABCA6 |  |  |  |  |  |  |  |
| STAB1 |  |  |  |  |  |  |  |
| ANKRD55 |  |  |  |  |  |  |  |
| ENSG00000261635 |  |  |  |  |  |  |  |
| ENSG00000279932 |  |  |  |  |  |  |  |
| ZNF566-AS1 |  |  |  |  |  |  |  |
| LINC01622 |  |  |  |  |  |  |  |
| ENSG00000272812 |  |  |  |  |  |  |  |
| THBS1 |  |  |  |  |  |  |  |
| RAB3C |  |  |  |  |  |  |  |
| TAS2R14 |  |  |  |  |  |  |  |
| SUCLG1 |  |  |  |  |  |  |  |
| ENSG00000250280 |  |  |  |  |  |  |  |
| CNIH1 |  |  |  |  |  |  |  |
| HDGFL3 |  |  |  |  |  |  |  |
| RABGAP1L-IT1 |  |  |  |  |  |  |  |
| PAM16 |  |  |  |  |  |  |  |
| COQ10A |  |  |  |  |  |  |  |
| TFCP2L1 |  |  |  |  |  |  |  |
| ENSG00000278668 |  |  |  |  |  |  |  |
| ENSG00000285581 |  |  |  |  |  |  |  |
| TRABD2B |  |  |  |  |  |  |  |
| ZNNT1 |  |  |  |  |  |  |  |
| CYP4X1 |  |  |  |  |  |  |  |
| CRYAB |  |  |  |  |  |  |  |
| HPS5 |  |  |  |  |  |  |  |
| SFTPA2 |  |  |  |  |  |  |  |
| ZNF215 |  |  |  |  |  |  |  |
| MTND2P2 |  |  |  |  |  |  |  |
| PIGAP1 |  |  |  |  |  |  |  |
| APLN |  |  |  |  |  |  |  |
| ENSG00000250138 |  |  |  |  |  |  |  |
| SLC27A1 |  |  |  |  |  |  |  |
| ENSG00000248863 |  |  |  |  |  |  |  |
| BOP1 |  |  |  |  |  |  |  |
| CDNF |  |  |  |  |  |  |  |
| IER5 |  |  |  |  |  |  |  |
| SLC45A2 |  |  |  |  |  |  |  |
| CCL3L1 |  |  |  |  |  |  |  |
| MAPK13 |  |  |  |  |  |  |  |
| TP53I13 |  |  |  |  |  |  |  |
| PDE6B |  |  |  |  |  |  |  |
| CD300C |  |  |  |  |  |  |  |
| ENSG00000238061 |  |  |  |  |  |  |  |
| ENSG00000276248 |  |  |  |  |  |  |  |
| ITGB6 |  |  |  |  |  |  |  |
| DUSP19 |  |  |  |  |  |  |  |
| MEX3A |  |  |  |  |  |  |  |
| MLLT11 |  |  |  |  |  |  |  |
| FAM83H |  |  |  |  |  |  |  |
| TGFB1 |  |  |  |  |  |  |  |
| CTAGE3P |  |  |  |  |  |  |  |
| H3C11 |  |  |  |  |  |  |  |
| CAPN12 |  |  |  |  |  |  |  |
| ENSG00000279836 |  |  |  |  |  |  |  |
| GJC1 |  |  |  |  |  |  |  |
| ENSG00000278236 |  |  |  |  |  |  |  |
| PKD1 |  |  |  |  |  |  |  |
| SCRN1 |  |  |  |  |  |  |  |
| BAIAP3 |  |  |  |  |  |  |  |
| ENSG00000279958 |  |  |  |  |  |  |  |
| FGGY-DT |  |  |  |  |  |  |  |
| MICU3 |  |  |  |  |  |  |  |
| DAGLA |  |  |  |  |  |  |  |
| CDH5 |  |  |  |  |  |  |  |
| CDKL2 |  |  |  |  |  |  |  |
| HBA1 |  |  |  |  |  |  |  |
| HMGB1P31 |  |  |  |  |  |  |  |
| OPLAH |  |  |  |  |  |  |  |
| CCL3 |  |  |  |  |  |  |  |
| STARD4 |  |  |  |  |  |  |  |
| COX6CP17 |  |  |  |  |  |  |  |
| ENSG00000200075 |  |  |  |  |  |  |  |
| ENSG00000285269 |  |  |  |  |  |  |  |
| MGP |  |  |  |  |  |  |  |
| U6 |  |  |  |  |  |  |  |
| ENSG00000287932 |  |  |  |  |  |  |  |
| C17orf97 |  |  |  |  |  |  |  |
| ENSG00000280274 |  |  |  |  |  |  |  |
| FOLH1 |  |  |  |  |  |  |  |
| RGPD3 |  |  |  |  |  |  |  |
| GALNT14 |  |  |  |  |  |  |  |
| DHX34 |  |  |  |  |  |  |  |
| NET1 |  |  |  |  |  |  |  |
| ENSG00000166104 |  |  |  |  |  |  |  |
| ENSG00000269867 |  |  |  |  |  |  |  |
| CYB5D1 |  |  |  |  |  |  |  |
| ENSG00000269938 |  |  |  |  |  |  |  |
| KCNMB4 |  |  |  |  |  |  |  |
| HBA2 |  |  |  |  |  |  |  |
| CHAC1 |  |  |  |  |  |  |  |
| ESYT3 |  |  |  |  |  |  |  |
| PTPRO |  |  |  |  |  |  |  |
| XYLT1 |  |  |  |  |  |  |  |
| FBXO32 |  |  |  |  |  |  |  |
| MT-ND2 |  |  |  |  |  |  |  |
| RASA4CP |  |  |  |  |  |  |  |
| SLC25A4 |  |  |  |  |  |  |  |
| CKAP2L |  |  |  |  |  |  |  |
| GJA1 |  |  |  |  |  |  |  |
| ENSG00000258732 |  |  |  |  |  |  |  |
| SELE |  |  |  |  |  |  |  |
| AMDHD2 |  |  |  |  |  |  |  |
| CHKB-CPT1B |  |  |  |  |  |  |  |
| TSPAN2 |  |  |  |  |  |  |  |
| CBR3 |  |  |  |  |  |  |  |
| HSPB8 |  |  |  |  |  |  |  |
| ABTB1 |  |  |  |  |  |  |  |
| MPV17L |  |  |  |  |  |  |  |
| PIK3CG |  |  |  |  |  |  |  |
| STMN1P1 |  |  |  |  |  |  |  |
| PIM3 |  |  |  |  |  |  |  |
| ZDHHC1 |  |  |  |  |  |  |  |
| RTL8B |  |  |  |  |  |  |  |
| RPLP0P9 |  |  |  |  |  |  |  |
| CCN3 |  |  |  |  |  |  |  |
| PGRMC1 |  |  |  |  |  |  |  |
| TCAP |  |  |  |  |  |  |  |
| ENSG00000270605 |  |  |  |  |  |  |  |
| PLEKHH2 |  |  |  |  |  |  |  |
| PRR5L |  |  |  |  |  |  |  |
| RHPN1 |  |  |  |  |  |  |  |
| USP2-AS1 |  |  |  |  |  |  |  |
| IMPDH1P8 |  |  |  |  |  |  |  |
| SPOCK1 |  |  |  |  |  |  |  |
| ENSG00000260912 |  |  |  |  |  |  |  |
| ENSG00000271141 |  |  |  |  |  |  |  |
| GNAZ |  |  |  |  |  |  |  |
| IGHG1 |  |  |  |  |  |  |  |
| ENSG00000254061 |  |  |  |  |  |  |  |
| RAMP1 |  |  |  |  |  |  |  |
| MUC16 |  |  |  |  |  |  |  |
| SNX29P2 |  |  |  |  |  |  |  |
| TIGD5 |  |  |  |  |  |  |  |
| LAYN |  |  |  |  |  |  |  |
| PRICKLE1 |  |  |  |  |  |  |  |
| KCNJ5 |  |  |  |  |  |  |  |
| EXTL3-AS1 |  |  |  |  |  |  |  |
| NPDC1 |  |  |  |  |  |  |  |
| CFAP91 |  |  |  |  |  |  |  |
| ENSG00000227066 |  |  |  |  |  |  |  |
| SUDS3P1 |  |  |  |  |  |  |  |
| MFSD6 |  |  |  |  |  |  |  |
| A4GALT |  |  |  |  |  |  |  |
| E2F1 |  |  |  |  |  |  |  |
| LINC02970 |  |  |  |  |  |  |  |
| EVC |  |  |  |  |  |  |  |
| SLIT3 |  |  |  |  |  |  |  |
| IL27RA |  |  |  |  |  |  |  |
| ENSG00000187904 |  |  |  |  |  |  |  |
| ARMC5 |  |  |  |  |  |  |  |
| DOK6 |  |  |  |  |  |  |  |
| EFNA1 |  |  |  |  |  |  |  |
| ENSG00000286912 |  |  |  |  |  |  |  |
| PCDHB2 |  |  |  |  |  |  |  |
| PCDH12 |  |  |  |  |  |  |  |
| ENSG00000267152 |  |  |  |  |  |  |  |
| FLT1 |  |  |  |  |  |  |  |
| PKN3 |  |  |  |  |  |  |  |
| TAGLN |  |  |  |  |  |  |  |
| RGPD8 |  |  |  |  |  |  |  |
| LAMC2 |  |  |  |  |  |  |  |
| MYORG |  |  |  |  |  |  |  |
| CELSR3 |  |  |  |  |  |  |  |
| LINC01132 |  |  |  |  |  |  |  |
| ABCA3 |  |  |  |  |  |  |  |
| ENSG00000242299 |  |  |  |  |  |  |  |
| RPS28P7 |  |  |  |  |  |  |  |
| LRFN4 |  |  |  |  |  |  |  |
| CCL14 |  |  |  |  |  |  |  |
| STX1A |  |  |  |  |  |  |  |
| LINC00240 |  |  |  |  |  |  |  |
| ENSG00000250568 |  |  |  |  |  |  |  |
| IGLV1-44 |  |  |  |  |  |  |  |
| ITGB1BP2 |  |  |  |  |  |  |  |
| ACSM3 |  |  |  |  |  |  |  |
| ENSG00000269514 |  |  |  |  |  |  |  |
| HOPX |  |  |  |  |  |  |  |
| ATP2A1 |  |  |  |  |  |  |  |
| COX7B |  |  |  |  |  |  |  |
| RN7SL378P |  |  |  |  |  |  |  |
| ATP7B |  |  |  |  |  |  |  |
| ZNF511 |  |  |  |  |  |  |  |
| SESN2 |  |  |  |  |  |  |  |
| ENSG00000238966 |  |  |  |  |  |  |  |
| HMGB1P41 |  |  |  |  |  |  |  |
| CLDN10 |  |  |  |  |  |  |  |
| NKIRAS1 |  |  |  |  |  |  |  |
| IFIT1 |  |  |  |  |  |  |  |
| FGFR4 |  |  |  |  |  |  |  |
| STEAP1B |  |  |  |  |  |  |  |
| PMS2P6 |  |  |  |  |  |  |  |
| NSMCE1-DT |  |  |  |  |  |  |  |
| ALOX15P1 |  |  |  |  |  |  |  |
| GPR37 |  |  |  |  |  |  |  |
| ENSG00000267681 |  |  |  |  |  |  |  |
| ENSG00000274322 |  |  |  |  |  |  |  |
| KCNE3 |  |  |  |  |  |  |  |
| BEND5 |  |  |  |  |  |  |  |
| SEC61G |  |  |  |  |  |  |  |
| KIF14 |  |  |  |  |  |  |  |
| ENSG00000287074 |  |  |  |  |  |  |  |
| ACAP2-IT1 |  |  |  |  |  |  |  |
| TNFAIP8L2 |  |  |  |  |  |  |  |
| TMEM238 |  |  |  |  |  |  |  |
| UPP1 |  |  |  |  |  |  |  |
| ENSG00000279689 |  |  |  |  |  |  |  |
| KIF21A |  |  |  |  |  |  |  |
| RCSD1 |  |  |  |  |  |  |  |
| LINC01948 |  |  |  |  |  |  |  |
| GPBAR1 |  |  |  |  |  |  |  |
| TEAD4 |  |  |  |  |  |  |  |
| RAMP3 |  |  |  |  |  |  |  |
| MMP24 |  |  |  |  |  |  |  |
| ENSG00000246225 |  |  |  |  |  |  |  |
| COLGALT2 |  |  |  |  |  |  |  |
| ENSG00000266993 |  |  |  |  |  |  |  |
| PLOD1 |  |  |  |  |  |  |  |
| GPC4 |  |  |  |  |  |  |  |
| ENSG00000285867 |  |  |  |  |  |  |  |
| CIT |  |  |  |  |  |  |  |
| HEATR4 |  |  |  |  |  |  |  |
| SNORD117 |  |  |  |  |  |  |  |
| CCN1 |  |  |  |  |  |  |  |
| RGS10 |  |  |  |  |  |  |  |
| ENSG00000261512 |  |  |  |  |  |  |  |
| PROM2 |  |  |  |  |  |  |  |
| SRPX |  |  |  |  |  |  |  |
| MAP1B |  |  |  |  |  |  |  |
| TMC4 |  |  |  |  |  |  |  |
| PLXDC2 |  |  |  |  |  |  |  |
| THNSL1 |  |  |  |  |  |  |  |
| ENSG00000231466 |  |  |  |  |  |  |  |
| MIR3609 |  |  |  |  |  |  |  |
| ARHGAP30 |  |  |  |  |  |  |  |
| FAM107A |  |  |  |  |  |  |  |
| GAS1 |  |  |  |  |  |  |  |
| ENSG00000273156 |  |  |  |  |  |  |  |
| ENSG00000237813 |  |  |  |  |  |  |  |
| XACT |  |  |  |  |  |  |  |
| DEPP1 |  |  |  |  |  |  |  |
| TOB1 |  |  |  |  |  |  |  |
| STEAP2 |  |  |  |  |  |  |  |
| ENSG00000227992 |  |  |  |  |  |  |  |
| RASL10B |  |  |  |  |  |  |  |
| ENSG00000254325 |  |  |  |  |  |  |  |
| ARHGEF4 |  |  |  |  |  |  |  |
| BIRC3 |  |  |  |  |  |  |  |
| DOCK7-DT |  |  |  |  |  |  |  |
| LILRA1 |  |  |  |  |  |  |  |
| PABPC1L |  |  |  |  |  |  |  |
| TLNRD1 |  |  |  |  |  |  |  |
| SPATA2L |  |  |  |  |  |  |  |
| HORMAD1 |  |  |  |  |  |  |  |
| GNB1L |  |  |  |  |  |  |  |
| FAM133CP |  |  |  |  |  |  |  |
| LST1 |  |  |  |  |  |  |  |
| ADAMTS1 |  |  |  |  |  |  |  |
| ANKRD37 |  |  |  |  |  |  |  |
| ENSG00000275367 |  |  |  |  |  |  |  |
| ARHGEF19 |  |  |  |  |  |  |  |
| ENSG00000272638 |  |  |  |  |  |  |  |
| ADAMTS14 |  |  |  |  |  |  |  |
| TSPAN33 |  |  |  |  |  |  |  |
| ENSG00000274964 |  |  |  |  |  |  |  |
| PRELP |  |  |  |  |  |  |  |
| SNORD15B |  |  |  |  |  |  |  |
| ZNF385A |  |  |  |  |  |  |  |
| CXCL1 |  |  |  |  |  |  |  |
| PTPRN2 |  |  |  |  |  |  |  |
| LIF |  |  |  |  |  |  |  |
| MT-TQ |  |  |  |  |  |  |  |
| ENSG00000283415 |  |  |  |  |  |  |  |
| RGS5 |  |  |  |  |  |  |  |
| ENSG00000260711 |  |  |  |  |  |  |  |
| EDIL3 |  |  |  |  |  |  |  |
| TMED7 |  |  |  |  |  |  |  |
| MUC5B |  |  |  |  |  |  |  |
| IGKV1-5 |  |  |  |  |  |  |  |
| PNPLA7 |  |  |  |  |  |  |  |
| DPEP2 |  |  |  |  |  |  |  |
| PYGO1 |  |  |  |  |  |  |  |
| TNFAIP6 |  |  |  |  |  |  |  |
| ABHD6 |  |  |  |  |  |  |  |
| NAV3 |  |  |  |  |  |  |  |
| VNN2 |  |  |  |  |  |  |  |
| SPESP1 |  |  |  |  |  |  |  |
| PIK3R6 |  |  |  |  |  |  |  |
| MAN1B1-DT |  |  |  |  |  |  |  |
| TPM2 |  |  |  |  |  |  |  |
| RIMS3 |  |  |  |  |  |  |  |
| STAT4 |  |  |  |  |  |  |  |
| DUSP5 |  |  |  |  |  |  |  |
| MBD3 |  |  |  |  |  |  |  |
| SYTL1 |  |  |  |  |  |  |  |
| LOXHD1 |  |  |  |  |  |  |  |
| ENSG00000286129 |  |  |  |  |  |  |  |
| MINAR1 |  |  |  |  |  |  |  |
| GREM2 |  |  |  |  |  |  |  |
| NEXN |  |  |  |  |  |  |  |
| NMRAL2P |  |  |  |  |  |  |  |
| TBC1D10C |  |  |  |  |  |  |  |
| VGLL3 |  |  |  |  |  |  |  |
| ADGRG1 |  |  |  |  |  |  |  |
| ATF3 |  |  |  |  |  |  |  |
| ID1 |  |  |  |  |  |  |  |
| PPM1M |  |  |  |  |  |  |  |
| ENSG00000279328 |  |  |  |  |  |  |  |
| FHOD1 |  |  |  |  |  |  |  |
| AADAT |  |  |  |  |  |  |  |
| ENSG00000284602 |  |  |  |  |  |  |  |
| SAMD5 |  |  |  |  |  |  |  |
| SOX7 |  |  |  |  |  |  |  |
| MAPK4 |  |  |  |  |  |  |  |
| ENSG00000232334 |  |  |  |  |  |  |  |
| SCTR |  |  |  |  |  |  |  |
| THRB-IT1 |  |  |  |  |  |  |  |
| ADAMTS7 |  |  |  |  |  |  |  |
| SPOPL-DT |  |  |  |  |  |  |  |
