# Supplementary material for: Potential association between COVID-19 and neurological disorders: analysis of common genes and therapeutics
Source: Front Neurol. 2024 Oct 14;15:1417183. doi: 10.3389/fneur.2024.1417183 (PMC11513677; doi:10.3389/fneur.2024.1417183)
Supplement: Supplementary file 2 [file Table_2.docx]

**Supplementary Table 2**

**Intersection Distribution**

| Genes | IS | PD | EP | AD | HS | SD | COVID-19 |
| --- | --- | --- | --- | --- | --- | --- | --- |
| CD6 | 1 | 0 | 0 | 0 | 0 | 0 | 0 |
| PDK4 | 1 | 0 | 0 | 0 | 0 | 0 | 0 |
| ARG1 | 1 | 0 | 0 | 0 | 0 | 0 | 1 |
| MAL | 1 | 0 | 0 | 0 | 0 | 1 | 0 |
| FAIM3 | 1 | 0 | 0 | 0 | 0 | 0 | 0 |
| IL7R | 1 | 0 | 0 | 0 | 0 | 0 | 0 |
| IQGAP1 | 1 | 0 | 0 | 0 | 0 | 0 | 1 |
| CCR7 | 1 | 0 | 0 | 0 | 0 | 0 | 0 |
| MMP9 | 1 | 0 | 0 | 0 | 0 | 0 | 0 |
| CA4 | 1 | 0 | 0 | 0 | 0 | 1 | 0 |
| ACSL1 | 1 | 0 | 0 | 0 | 0 | 0 | 1 |
| S100A12 | 1 | 0 | 0 | 0 | 1 | 0 | 0 |
| FOLR3 | 1 | 0 | 0 | 0 | 0 | 0 | 0 |
| LY96 | 1 | 0 | 0 | 0 | 0 | 0 | 0 |
| BNIP3L | 1 | 0 | 0 | 0 | 1 | 0 | 0 |
| APOBEC3A | 1 | 0 | 0 | 0 | 0 | 0 | 0 |
| ORM1 | 1 | 0 | 0 | 0 | 0 | 1 | 0 |
| FCGR3B | 1 | 0 | 0 | 1 | 0 | 0 | 0 |
| FTHL3 | 1 | 0 | 0 | 0 | 0 | 0 | 0 |
| FTHL11 | 1 | 0 | 0 | 0 | 0 | 0 | 0 |
| FCGR3A | 1 | 0 | 0 | 0 | 0 | 0 | 0 |
| TLR10 | 0 | 1 | 0 | 0 | 0 | 0 | 0 |
| OSBPL10 | 0 | 1 | 0 | 0 | 0 | 0 | 0 |
| SNORA28 | 0 | 1 | 0 | 0 | 0 | 0 | 0 |
| FOS | 0 | 1 | 0 | 0 | 0 | 1 | 1 |
| FCRLA | 0 | 1 | 0 | 0 | 0 | 0 | 0 |
| PDXDC2P | 0 | 1 | 0 | 0 | 0 | 0 | 0 |
| CD24 | 0 | 1 | 0 | 0 | 0 | 0 | 0 |
| ABCA7 | 0 | 1 | 0 | 0 | 0 | 0 | 0 |
| PTPRC | 0 | 1 | 0 | 0 | 0 | 0 | 0 |
| LOC90925 | 0 | 1 | 0 | 0 | 0 | 0 | 0 |
| SNX29 | 0 | 1 | 0 | 0 | 0 | 0 | 0 |
| FOSB | 0 | 1 | 0 | 0 | 1 | 0 | 1 |
| JUP | 0 | 1 | 0 | 0 | 0 | 0 | 0 |
| AMFR | 0 | 1 | 0 | 0 | 0 | 0 | 0 |
| POU2AF1 | 0 | 1 | 0 | 1 | 0 | 0 | 0 |
| EGR1 | 0 | 1 | 0 | 0 | 0 | 0 | 1 |
| C16orf7 | 0 | 1 | 0 | 0 | 0 | 0 | 0 |
| MS4A1 | 0 | 1 | 0 | 0 | 0 | 0 | 0 |
| TBC1D10B | 0 | 1 | 0 | 0 | 0 | 1 | 0 |
| KLF4 | 0 | 1 | 0 | 0 | 0 | 0 | 1 |
| CXCR4 | 0 | 1 | 0 | 0 | 0 | 0 | 0 |
| STAB1 | 0 | 1 | 0 | 0 | 0 | 0 | 1 |
| AL359560 | 0 | 1 | 0 | 0 | 0 | 0 | 0 |
| LOC728153 | 0 | 1 | 0 | 0 | 0 | 0 | 0 |
| RRP12 | 0 | 1 | 0 | 0 | 0 | 0 | 0 |
| LOC338817 | 0 | 1 | 0 | 0 | 0 | 0 | 0 |
| POU2F2 | 0 | 1 | 0 | 0 | 0 | 0 | 0 |
| BANK1 | 0 | 1 | 0 | 0 | 0 | 0 | 1 |
| E2F5 | 0 | 1 | 0 | 0 | 0 | 0 | 0 |
| RP2 | 0 | 1 | 0 | 0 | 0 | 0 | 0 |
| MOV10 | 0 | 1 | 0 | 0 | 0 | 0 | 0 |
| TSPAN13 | 0 | 1 | 0 | 0 | 0 | 0 | 1 |
| WARS | 0 | 1 | 0 | 0 | 1 | 0 | 0 |
| CD79A | 0 | 1 | 0 | 0 | 0 | 0 | 0 |
| SF3A1 | 0 | 1 | 0 | 0 | 1 | 0 | 0 |
| TNFAIP2 | 0 | 1 | 0 | 0 | 1 | 0 | 0 |
| ID3 | 0 | 1 | 0 | 0 | 0 | 0 | 1 |
| LOC284837 | 0 | 1 | 0 | 0 | 0 | 0 | 0 |
| BLK | 0 | 1 | 0 | 0 | 0 | 0 | 0 |
| NAG8 | 0 | 1 | 0 | 0 | 0 | 0 | 0 |
| AK129699 | 0 | 1 | 0 | 0 | 0 | 0 | 0 |
| ATG16L2 | 0 | 1 | 0 | 0 | 0 | 0 | 0 |
| GNG7 | 0 | 1 | 0 | 0 | 0 | 0 | 1 |
| P2RX1 | 0 | 1 | 0 | 0 | 0 | 1 | 0 |
| KLHL14 | 0 | 1 | 0 | 0 | 0 | 0 | 0 |
| MDFIC | 0 | 1 | 0 | 0 | 0 | 0 | 0 |
| CD1C | 0 | 1 | 0 | 0 | 0 | 0 | 0 |
| CPT1B | 0 | 1 | 0 | 0 | 0 | 0 | 0 |
| ZNF679 | 0 | 1 | 0 | 0 | 0 | 0 | 0 |
| DUSP1 | 0 | 1 | 0 | 0 | 0 | 0 | 1 |
| PARP12 | 0 | 1 | 0 | 0 | 0 | 0 | 0 |
| X69637 | 0 | 1 | 0 | 0 | 0 | 0 | 0 |
| MORC3 | 0 | 1 | 0 | 0 | 0 | 0 | 0 |
| CD19 | 0 | 1 | 0 | 0 | 0 | 1 | 0 |
| ERAP2 | 0 | 1 | 0 | 0 | 0 | 1 | 0 |
| STAT2 | 0 | 1 | 0 | 0 | 0 | 0 | 0 |
| STAP1 | 0 | 1 | 0 | 0 | 0 | 0 | 0 |
| TYMP | 0 | 1 | 0 | 0 | 1 | 0 | 0 |
| SAMD9L | 0 | 1 | 0 | 0 | 0 | 0 | 0 |
| VPREB3 | 0 | 1 | 0 | 0 | 0 | 0 | 0 |
| FCRL5 | 0 | 1 | 0 | 0 | 0 | 0 | 0 |
| BBS10 | 0 | 1 | 0 | 0 | 0 | 0 | 0 |
| AK024852 | 0 | 1 | 0 | 0 | 0 | 0 | 0 |
| GABBR1 | 0 | 1 | 0 | 0 | 0 | 0 | 0 |
| FREM1 | 0 | 0 | 1 | 0 | 0 | 0 | 0 |
| SUSD2 | 0 | 0 | 1 | 0 | 0 | 0 | 1 |
| FRAS1 | 0 | 0 | 1 | 0 | 0 | 0 | 0 |
| ERICH1-AS1 | 0 | 0 | 1 | 0 | 0 | 0 | 0 |
| RP11-627G23.1 | 0 | 0 | 1 | 0 | 0 | 0 | 0 |
| CTD-2047H16.4 | 0 | 0 | 1 | 0 | 0 | 0 | 0 |
| COLQ | 0 | 0 | 1 | 0 | 0 | 0 | 0 |
| MIR143HG | 0 | 0 | 1 | 0 | 1 | 0 | 0 |
| AC131025.8 | 0 | 0 | 1 | 0 | 0 | 0 | 0 |
| RBPMS | 0 | 0 | 1 | 0 | 0 | 0 | 0 |
| MUC19 | 0 | 0 | 1 | 0 | 0 | 0 | 0 |
| LAMA5 | 0 | 0 | 1 | 0 | 0 | 0 | 1 |
| TTC21A | 0 | 0 | 1 | 0 | 0 | 0 | 0 |
| CTD-2349P21.5 | 0 | 0 | 1 | 0 | 0 | 0 | 0 |
| LIMS2 | 0 | 0 | 1 | 0 | 0 | 0 | 1 |
| IQCJ | 0 | 0 | 1 | 0 | 0 | 0 | 0 |
| ANKRD19P | 0 | 0 | 1 | 0 | 0 | 0 | 0 |
| MIR145 | 0 | 0 | 1 | 0 | 0 | 0 | 0 |
| HSPG2 | 0 | 0 | 1 | 0 | 0 | 0 | 0 |
| COL14A1 | 0 | 0 | 1 | 0 | 0 | 0 | 0 |
| AC093642.3 | 0 | 0 | 1 | 0 | 0 | 0 | 0 |
| MIAT | 0 | 0 | 1 | 0 | 0 | 0 | 0 |
| FAM118A | 0 | 0 | 1 | 0 | 0 | 1 | 0 |
| AKT3-IT1 | 0 | 0 | 1 | 0 | 0 | 0 | 1 |
| RP4-673D20.3 | 0 | 0 | 1 | 0 | 0 | 0 | 0 |
| CASQ2 | 0 | 0 | 1 | 0 | 0 | 0 | 1 |
| RP4-555D20.2 | 0 | 0 | 1 | 0 | 0 | 0 | 0 |
| MYOCD | 0 | 0 | 1 | 0 | 0 | 0 | 0 |
| RP11-159D12.2 | 0 | 0 | 1 | 0 | 0 | 0 | 0 |
| CAPG | 0 | 0 | 1 | 0 | 0 | 1 | 1 |
| RP6-99M1.2 | 0 | 0 | 1 | 0 | 0 | 0 | 0 |
| GAPDHP66 | 0 | 0 | 1 | 0 | 0 | 0 | 0 |
| FGF14-IT1 | 0 | 0 | 1 | 0 | 0 | 0 | 0 |
| AC144833.1 | 0 | 0 | 1 | 0 | 0 | 0 | 0 |
| ROCK1P1 | 0 | 0 | 1 | 0 | 0 | 0 | 0 |
| C20orf203 | 0 | 0 | 1 | 0 | 0 | 0 | 0 |
| HNRNPCP6 | 0 | 0 | 1 | 0 | 0 | 0 | 1 |
| RP11-989F5.3 | 0 | 0 | 1 | 0 | 0 | 0 | 0 |
| CRYZ | 0 | 0 | 1 | 0 | 0 | 0 | 1 |
| COPS8P2 | 0 | 0 | 1 | 0 | 0 | 0 | 0 |
| AOC3 | 0 | 0 | 1 | 0 | 0 | 0 | 1 |
| ZNF410 | 0 | 0 | 1 | 0 | 0 | 0 | 0 |
| MIR770 | 0 | 0 | 1 | 0 | 0 | 0 | 0 |
| CEACAM19 | 0 | 0 | 1 | 0 | 0 | 0 | 0 |
| FOXL1 | 0 | 0 | 1 | 0 | 0 | 0 | 0 |
| RP11-379B18.5 | 0 | 0 | 1 | 0 | 0 | 0 | 0 |
| PVT1 | 0 | 0 | 1 | 0 | 0 | 0 | 0 |
| ADIRF-AS1 | 0 | 0 | 1 | 0 | 0 | 0 | 1 |
| RP11-981G7.2 | 0 | 0 | 1 | 0 | 0 | 0 | 0 |
| RP11-54A4.2 | 0 | 0 | 1 | 0 | 0 | 0 | 0 |
| GJA6P | 0 | 0 | 1 | 0 | 0 | 0 | 0 |
| SCUBE3 | 0 | 0 | 1 | 0 | 0 | 0 | 0 |
| RP11-768G7.2 | 0 | 0 | 1 | 0 | 0 | 0 | 0 |
| SLC25A34 | 0 | 0 | 1 | 0 | 1 | 0 | 0 |
| AC019118.2 | 0 | 0 | 1 | 0 | 0 | 0 | 0 |
| AC011747.4 | 0 | 0 | 1 | 0 | 0 | 0 | 0 |
| H3F3AP4 | 0 | 0 | 1 | 0 | 0 | 0 | 0 |
| LINC00599 | 0 | 0 | 1 | 0 | 0 | 0 | 0 |
| RP11-509E10.1 | 0 | 0 | 1 | 0 | 0 | 0 | 0 |
| VWCE | 0 | 0 | 1 | 0 | 0 | 1 | 0 |
| BTBD19 | 0 | 0 | 1 | 0 | 0 | 0 | 0 |
| CTD-2281E23.2 | 0 | 0 | 1 | 0 | 0 | 0 | 0 |
| RP11-535M15.2 | 0 | 0 | 1 | 0 | 0 | 0 | 0 |
| RP11-981G7.1 | 0 | 0 | 1 | 0 | 0 | 0 | 0 |
| MIR137HG | 0 | 0 | 1 | 0 | 0 | 0 | 0 |
| RNF139-AS1 | 0 | 0 | 1 | 0 | 0 | 0 | 0 |
| PTCH2 | 0 | 0 | 1 | 0 | 0 | 0 | 0 |
| ADAMTS15 | 0 | 0 | 1 | 0 | 0 | 0 | 0 |
| CTA-407F11.6 | 0 | 0 | 1 | 0 | 0 | 0 | 0 |
| SMYD3-IT1 | 0 | 0 | 1 | 0 | 0 | 0 | 1 |
| ELN | 0 | 0 | 1 | 1 | 0 | 0 | 0 |
| MKRN5P | 0 | 0 | 1 | 0 | 0 | 0 | 0 |
| GNB3 | 0 | 0 | 1 | 0 | 0 | 0 | 0 |
| RP11-282K24.3 | 0 | 0 | 1 | 0 | 0 | 0 | 0 |
| XXbac-BPG154L12.4 | 0 | 0 | 1 | 0 | 0 | 0 | 0 |
| C1orf132 | 0 | 0 | 1 | 0 | 0 | 0 | 0 |
| AF131215.3 | 0 | 0 | 1 | 0 | 0 | 0 | 0 |
| RP11-53B2.2 | 0 | 0 | 1 | 0 | 0 | 0 | 0 |
| RP11-981G7.6 | 0 | 0 | 1 | 0 | 0 | 0 | 0 |
| CORO6 | 0 | 0 | 1 | 0 | 0 | 0 | 0 |
| MEG3 | 0 | 0 | 1 | 0 | 0 | 0 | 1 |
| DNAJC5G | 0 | 0 | 1 | 1 | 0 | 0 | 0 |
| LAMA3 | 0 | 0 | 1 | 0 | 0 | 0 | 1 |
| RP11-16E23.4 | 0 | 0 | 1 | 0 | 0 | 0 | 0 |
| FAM153B | 0 | 0 | 1 | 0 | 0 | 1 | 0 |
| GET4 | 0 | 0 | 1 | 0 | 0 | 0 | 0 |
| LINC00595 | 0 | 0 | 1 | 0 | 0 | 0 | 0 |
| TPCN2 | 0 | 0 | 1 | 0 | 0 | 0 | 0 |
| RP11-367O10.1 | 0 | 0 | 1 | 0 | 0 | 0 | 0 |
| HIF3A | 0 | 0 | 1 | 1 | 0 | 0 | 0 |
| RP11-397O4.1 | 0 | 0 | 1 | 0 | 0 | 0 | 0 |
| AL132709.8 | 0 | 0 | 1 | 0 | 0 | 0 | 0 |
| GOLGA8A | 0 | 0 | 1 | 0 | 0 | 0 | 0 |
| DSCAM-IT1 | 0 | 0 | 1 | 0 | 0 | 0 | 0 |
| C6orf163 | 0 | 0 | 1 | 0 | 0 | 0 | 0 |
| C10orf113 | 0 | 0 | 1 | 0 | 0 | 0 | 0 |
| FGF17 | 0 | 0 | 1 | 0 | 0 | 0 | 0 |
| MSS51 | 0 | 0 | 1 | 0 | 0 | 0 | 0 |
| LINC00106 | 0 | 0 | 1 | 0 | 0 | 0 | 0 |
| RP11-572C21.1 | 0 | 0 | 1 | 0 | 0 | 0 | 0 |
| RP11-359E3.4 | 0 | 0 | 1 | 0 | 0 | 0 | 0 |
| C1RL-AS1 | 0 | 0 | 1 | 0 | 0 | 0 | 0 |
| HLA-DRA | 0 | 0 | 1 | 0 | 0 | 0 | 1 |
| CATSPER2 | 0 | 0 | 1 | 0 | 0 | 0 | 0 |
| GHRLOS | 0 | 0 | 1 | 0 | 0 | 0 | 0 |
| KLF15 | 0 | 0 | 0 | 1 | 0 | 0 | 0 |
| VGF | 0 | 0 | 0 | 1 | 0 | 0 | 0 |
| ADCYAP1 | 0 | 0 | 0 | 1 | 0 | 0 | 0 |
| SLC6A9 | 0 | 0 | 0 | 1 | 0 | 0 | 0 |
| NRN1 | 0 | 0 | 0 | 1 | 0 | 0 | 1 |
| SGO1 | 0 | 0 | 0 | 1 | 0 | 0 | 0 |
| NEUROD6 | 0 | 0 | 0 | 1 | 0 | 0 | 0 |
| PRKX | 0 | 0 | 0 | 1 | 1 | 0 | 0 |
| PRMT8 | 0 | 0 | 0 | 1 | 0 | 0 | 0 |
| PPEF1 | 0 | 0 | 0 | 1 | 0 | 0 | 0 |
| RPH3A | 0 | 0 | 0 | 1 | 0 | 0 | 0 |
| PCSK1 | 0 | 0 | 0 | 1 | 0 | 0 | 1 |
| JADE3 | 0 | 0 | 0 | 1 | 0 | 0 | 0 |
| C3orf80 | 0 | 0 | 0 | 1 | 0 | 0 | 0 |
| MID1IP1 | 0 | 0 | 0 | 1 | 0 | 1 | 0 |
| LOC102724596 | 0 | 0 | 0 | 1 | 0 | 0 | 0 |
| DNAH11 | 0 | 0 | 0 | 1 | 0 | 0 | 0 |
| GNRH1 | 0 | 0 | 0 | 1 | 0 | 0 | 0 |
| MAS1 | 0 | 0 | 0 | 1 | 0 | 0 | 0 |
| SOWAHB | 0 | 0 | 0 | 1 | 0 | 0 | 0 |
| FAM53B-AS1 | 0 | 0 | 0 | 1 | 0 | 0 | 0 |
| MSC | 0 | 0 | 0 | 1 | 0 | 0 | 0 |
| MDH1B | 0 | 0 | 0 | 1 | 0 | 0 | 1 |
| CLDN15 | 0 | 0 | 0 | 1 | 0 | 0 | 0 |
| CRH | 0 | 0 | 0 | 1 | 0 | 0 | 0 |
| FOXO4 | 0 | 0 | 0 | 1 | 0 | 1 | 1 |
| ABCC12 | 0 | 0 | 0 | 1 | 0 | 0 | 0 |
| TAC1 | 0 | 0 | 0 | 1 | 0 | 0 | 0 |
| OTOGL | 0 | 0 | 0 | 1 | 0 | 0 | 0 |
| USP2-AS1 | 0 | 0 | 0 | 1 | 0 | 0 | 1 |
| GCNT4 | 0 | 0 | 0 | 1 | 0 | 0 | 0 |
| BEX5 | 0 | 0 | 0 | 1 | 0 | 0 | 1 |
| NPFF | 0 | 0 | 0 | 1 | 0 | 0 | 0 |
| CPM | 0 | 0 | 0 | 1 | 0 | 0 | 1 |
| ALOX12B | 0 | 0 | 0 | 1 | 0 | 0 | 0 |
| LINC01546 | 0 | 0 | 0 | 1 | 0 | 0 | 0 |
| GJD2 | 0 | 0 | 0 | 1 | 0 | 0 | 0 |
| MGC16025 | 0 | 0 | 0 | 1 | 0 | 0 | 0 |
| FAM222A | 0 | 0 | 0 | 1 | 0 | 0 | 0 |
| LBX2 | 0 | 0 | 0 | 1 | 0 | 0 | 0 |
| HSPB3 | 0 | 0 | 0 | 1 | 0 | 0 | 0 |
| PCDHGC5 | 0 | 0 | 0 | 1 | 0 | 0 | 0 |
| KRT5 | 0 | 0 | 0 | 1 | 0 | 0 | 0 |
| STAT4 | 0 | 0 | 0 | 1 | 0 | 0 | 1 |
| ANKRD18DP | 0 | 0 | 0 | 1 | 0 | 0 | 0 |
| MCHR2 | 0 | 0 | 0 | 1 | 0 | 0 | 0 |
| PNMA3 | 0 | 0 | 0 | 1 | 0 | 0 | 0 |
| TNRC6C-AS1 | 0 | 0 | 0 | 1 | 0 | 0 | 0 |
| OVOL3 | 0 | 0 | 0 | 1 | 0 | 0 | 0 |
| C10orf62 | 0 | 0 | 0 | 1 | 0 | 0 | 0 |
| PPFIBP2 | 0 | 0 | 0 | 1 | 0 | 0 | 0 |
| LINC01561 | 0 | 0 | 0 | 1 | 0 | 0 | 0 |
| MPO | 0 | 0 | 0 | 1 | 0 | 0 | 0 |
| NAP1L2 | 0 | 0 | 0 | 1 | 0 | 0 | 0 |
| LOC100129316 | 0 | 0 | 0 | 1 | 0 | 0 | 0 |
| IL1RL2 | 0 | 0 | 0 | 1 | 0 | 0 | 1 |
| NAP1L5 | 0 | 0 | 0 | 1 | 0 | 0 | 1 |
| STON1 | 0 | 0 | 0 | 1 | 0 | 0 | 0 |
| ADRA2B | 0 | 0 | 0 | 1 | 0 | 0 | 0 |
| LINC01202 | 0 | 0 | 0 | 1 | 0 | 0 | 0 |
| THCAT155 | 0 | 0 | 0 | 1 | 0 | 0 | 0 |
| SST | 0 | 0 | 0 | 1 | 0 | 0 | 0 |
| NAT16 | 0 | 0 | 0 | 1 | 0 | 0 | 0 |
| CHML | 0 | 0 | 0 | 1 | 0 | 0 | 0 |
| ZBBX | 0 | 0 | 0 | 1 | 0 | 0 | 0 |
| MSC-AS1 | 0 | 0 | 0 | 1 | 0 | 0 | 1 |
| PAK1 | 0 | 0 | 0 | 1 | 1 | 0 | 0 |
| VSNL1 | 0 | 0 | 0 | 1 | 0 | 0 | 0 |
| FBLN7 | 0 | 0 | 0 | 1 | 0 | 0 | 0 |
| LOC100507194 | 0 | 0 | 0 | 1 | 0 | 0 | 0 |
| MCM7 | 0 | 0 | 0 | 1 | 0 | 0 | 0 |
| ADCY10P1 | 0 | 0 | 0 | 1 | 0 | 0 | 1 |
| SCG2 | 0 | 0 | 0 | 1 | 0 | 0 | 0 |
| HIP1 | 0 | 0 | 0 | 1 | 0 | 0 | 1 |
| PVRIG | 0 | 0 | 0 | 1 | 0 | 1 | 0 |
| RGS4 | 0 | 0 | 0 | 1 | 0 | 0 | 0 |
| LINC00898 | 0 | 0 | 0 | 1 | 0 | 0 | 0 |
| SH2D5 | 0 | 0 | 0 | 1 | 0 | 0 | 0 |
| CARTPT | 0 | 0 | 0 | 1 | 0 | 0 | 0 |
| SAP25 | 0 | 0 | 0 | 1 | 0 | 0 | 0 |
| CCDC184 | 0 | 0 | 0 | 1 | 0 | 0 | 0 |
| LDLRAP1 | 0 | 0 | 0 | 1 | 0 | 0 | 0 |
| ZNF652 | 0 | 0 | 0 | 1 | 0 | 0 | 0 |
| KIF1C | 0 | 0 | 0 | 1 | 0 | 0 | 0 |
| LIN28B-AS1 | 0 | 0 | 0 | 1 | 0 | 0 | 0 |
| PKMYT1 | 0 | 0 | 0 | 1 | 0 | 0 | 0 |
| SH2D6 | 0 | 0 | 0 | 1 | 0 | 0 | 0 |
| GAP43 | 0 | 0 | 0 | 1 | 0 | 0 | 0 |
| BEX1 | 0 | 0 | 0 | 1 | 0 | 0 | 0 |
| DTHD1 | 0 | 0 | 0 | 1 | 0 | 0 | 1 |
| SPATC1 | 0 | 0 | 0 | 1 | 0 | 0 | 0 |
| SPTSSB | 0 | 0 | 0 | 1 | 0 | 0 | 0 |
| ZBTB20-AS1 | 0 | 0 | 0 | 1 | 0 | 0 | 0 |
| LINC00463 | 0 | 0 | 0 | 1 | 0 | 0 | 0 |
| CRYM | 0 | 0 | 0 | 1 | 0 | 0 | 0 |
| UCKL1-AS1 | 0 | 0 | 0 | 1 | 1 | 0 | 0 |
| BLID | 0 | 0 | 0 | 1 | 0 | 0 | 0 |
| OLFM3 | 0 | 0 | 0 | 1 | 0 | 0 | 0 |
| LOC101926975 | 0 | 0 | 0 | 1 | 0 | 0 | 0 |
| SERTAD4-AS1 | 0 | 0 | 0 | 1 | 0 | 0 | 0 |
| LOC102724484 | 0 | 0 | 0 | 1 | 0 | 0 | 0 |
| MORN3 | 0 | 0 | 0 | 1 | 0 | 1 | 0 |
| LCN6 | 0 | 0 | 0 | 1 | 0 | 0 | 0 |
| ZDHHC23 | 0 | 0 | 0 | 1 | 0 | 0 | 0 |
| HMGCS1 | 0 | 0 | 0 | 1 | 0 | 0 | 1 |
| SERPINF2 | 0 | 0 | 0 | 1 | 0 | 0 | 1 |
| TLDC2 | 0 | 0 | 0 | 1 | 0 | 0 | 0 |
| NEAT1 | 0 | 0 | 0 | 1 | 0 | 0 | 0 |
| ITPKB-IT1 | 0 | 0 | 0 | 1 | 0 | 0 | 0 |
| LOC101929715 | 0 | 0 | 0 | 1 | 0 | 0 | 0 |
| PLA2G4B | 0 | 0 | 0 | 1 | 0 | 0 | 0 |
| LOC285762 | 0 | 0 | 0 | 1 | 0 | 0 | 0 |
| ATAD3C | 0 | 0 | 0 | 1 | 1 | 0 | 0 |
| CYP4B1 | 0 | 0 | 0 | 1 | 0 | 0 | 0 |
| CALY | 0 | 0 | 0 | 1 | 0 | 0 | 0 |
| LOC102723493 | 0 | 0 | 0 | 1 | 0 | 0 | 0 |
| SYP | 0 | 0 | 0 | 1 | 0 | 0 | 0 |
| TMPRSS5 | 0 | 0 | 0 | 1 | 0 | 0 | 0 |
| KIF19 | 0 | 0 | 0 | 1 | 0 | 0 | 0 |
| ARRDC2 | 0 | 0 | 0 | 1 | 0 | 0 | 0 |
| MYOT | 0 | 0 | 0 | 1 | 0 | 0 | 0 |
| FZD10-AS1 | 0 | 0 | 0 | 1 | 0 | 0 | 0 |
| LLGL2 | 0 | 0 | 0 | 1 | 0 | 0 | 1 |
| TUBB2A | 0 | 0 | 0 | 1 | 0 | 1 | 0 |
| PCA3 | 0 | 0 | 0 | 1 | 0 | 0 | 0 |
| MCHR1 | 0 | 0 | 0 | 1 | 0 | 0 | 0 |
| OLMALINC | 0 | 0 | 0 | 1 | 0 | 0 | 0 |
| SERTM1 | 0 | 0 | 0 | 1 | 0 | 0 | 0 |
| CLDN16 | 0 | 0 | 0 | 1 | 0 | 0 | 0 |
| PCP4L1 | 0 | 0 | 0 | 1 | 0 | 0 | 0 |
| SLC10A1 | 0 | 0 | 0 | 1 | 0 | 0 | 0 |
| LYRM9 | 0 | 0 | 0 | 1 | 0 | 0 | 0 |
| PTOV1-AS2 | 0 | 0 | 0 | 1 | 0 | 0 | 0 |
| OPTC | 0 | 0 | 0 | 1 | 0 | 0 | 0 |
| CPLX1 | 0 | 0 | 0 | 1 | 0 | 0 | 0 |
| VCAN-AS1 | 0 | 0 | 0 | 1 | 0 | 0 | 0 |
| FANCB | 0 | 0 | 0 | 1 | 0 | 0 | 0 |
| HTR3B | 0 | 0 | 0 | 1 | 0 | 0 | 0 |
| STYK1 | 0 | 0 | 0 | 1 | 0 | 0 | 0 |
| TUBB3 | 0 | 0 | 0 | 1 | 0 | 1 | 0 |
| OR7A5 | 0 | 0 | 0 | 1 | 0 | 0 | 0 |
| LINC01168 | 0 | 0 | 0 | 1 | 0 | 0 | 0 |
| LOC728084 | 0 | 0 | 0 | 1 | 0 | 0 | 0 |
| ARL4D | 0 | 0 | 0 | 1 | 0 | 0 | 0 |
| NRON | 0 | 0 | 0 | 1 | 0 | 0 | 0 |
| MIR6717 | 0 | 0 | 0 | 1 | 0 | 0 | 0 |
| PSG8 | 0 | 0 | 0 | 1 | 0 | 0 | 0 |
| ATOH7 | 0 | 0 | 0 | 1 | 0 | 0 | 0 |
| MAP4K4 | 0 | 0 | 0 | 1 | 0 | 0 | 0 |
| KMO | 0 | 0 | 0 | 1 | 0 | 0 | 1 |
| BAALC-AS1 | 0 | 0 | 0 | 1 | 0 | 0 | 1 |
| LINCR-0002 | 0 | 0 | 0 | 1 | 0 | 0 | 0 |
| CXCR1 | 0 | 0 | 0 | 1 | 0 | 1 | 0 |
| KCNB2 | 0 | 0 | 0 | 1 | 0 | 0 | 0 |
| C6orf223 | 0 | 0 | 0 | 1 | 0 | 0 | 0 |
| LINC01007 | 0 | 0 | 0 | 1 | 0 | 0 | 0 |
| LINC00460 | 0 | 0 | 0 | 1 | 0 | 0 | 0 |
| LOC100288637 | 0 | 0 | 0 | 1 | 0 | 0 | 0 |
| HES5 | 0 | 0 | 0 | 1 | 0 | 0 | 0 |
| LOC100130548 | 0 | 0 | 0 | 1 | 0 | 0 | 0 |
| SAMD11 | 0 | 0 | 0 | 1 | 0 | 0 | 0 |
| GABRA1 | 0 | 0 | 0 | 1 | 0 | 0 | 0 |
| GAD1 | 0 | 0 | 0 | 1 | 0 | 0 | 0 |
| GFRA2 | 0 | 0 | 0 | 1 | 0 | 0 | 0 |
| SCHLAP1 | 0 | 0 | 0 | 1 | 0 | 0 | 0 |
| GAD2 | 0 | 0 | 0 | 1 | 0 | 0 | 0 |
| RIIAD1 | 0 | 0 | 0 | 1 | 0 | 0 | 0 |
| LRRC73 | 0 | 0 | 0 | 1 | 0 | 0 | 0 |
| FAM222A-AS1 | 0 | 0 | 0 | 1 | 0 | 0 | 0 |
| IGF1 | 0 | 0 | 0 | 1 | 0 | 0 | 1 |
| ADAMTS3 | 0 | 0 | 0 | 1 | 0 | 0 | 0 |
| SLC23A3 | 0 | 0 | 0 | 1 | 0 | 0 | 0 |
| MRGPRF | 0 | 0 | 0 | 1 | 0 | 0 | 0 |
| FREM3 | 0 | 0 | 0 | 1 | 0 | 0 | 0 |
| LINC01164 | 0 | 0 | 0 | 1 | 0 | 0 | 0 |
| BOK | 0 | 0 | 0 | 1 | 0 | 0 | 0 |
| LINC01445 | 0 | 0 | 0 | 1 | 0 | 0 | 0 |
| LINC00601 | 0 | 0 | 0 | 1 | 0 | 0 | 0 |
| LOC105378385 | 0 | 0 | 0 | 1 | 0 | 0 | 0 |
| QDPR | 0 | 0 | 0 | 1 | 0 | 0 | 0 |
| KIRREL3-AS3 | 0 | 0 | 0 | 1 | 0 | 0 | 0 |
| PRSS8 | 0 | 0 | 0 | 1 | 0 | 0 | 1 |
| PTPRD-AS2 | 0 | 0 | 0 | 1 | 0 | 0 | 0 |
| LOC339685 | 0 | 0 | 0 | 1 | 0 | 0 | 0 |
| SNAP25 | 0 | 0 | 0 | 1 | 0 | 0 | 0 |
| CCKBR | 0 | 0 | 0 | 1 | 0 | 0 | 0 |
| PLPP2 | 0 | 0 | 0 | 1 | 0 | 0 | 0 |
| CDK18 | 0 | 0 | 0 | 1 | 0 | 0 | 0 |
| MDH1 | 0 | 0 | 0 | 1 | 0 | 0 | 0 |
| COL5A2 | 0 | 0 | 0 | 1 | 0 | 0 | 0 |
| MAMDC4 | 0 | 0 | 0 | 1 | 0 | 0 | 1 |
| FAM81A | 0 | 0 | 0 | 1 | 0 | 0 | 0 |
| OLAH | 0 | 0 | 0 | 1 | 0 | 0 | 1 |
| SLC12A1 | 0 | 0 | 0 | 1 | 0 | 0 | 0 |
| ABCA6 | 0 | 0 | 0 | 1 | 0 | 0 | 1 |
| ENC1 | 0 | 0 | 0 | 1 | 0 | 0 | 0 |
| AMIGO2 | 0 | 0 | 0 | 1 | 0 | 0 | 1 |
| EGFR-AS1 | 0 | 0 | 0 | 1 | 0 | 0 | 0 |
| BMP3 | 0 | 0 | 0 | 1 | 0 | 0 | 0 |
| CA3-AS1 | 0 | 0 | 0 | 1 | 0 | 0 | 0 |
| MAN2A1 | 0 | 0 | 0 | 1 | 0 | 0 | 0 |
| PPP1R2P3 | 0 | 0 | 0 | 1 | 0 | 0 | 0 |
| TRPA1 | 0 | 0 | 0 | 1 | 0 | 0 | 0 |
| CLDN34 | 0 | 0 | 0 | 1 | 0 | 0 | 0 |
| SMIM5 | 0 | 0 | 0 | 1 | 0 | 1 | 1 |
| C9orf139 | 0 | 0 | 0 | 1 | 0 | 0 | 0 |
| INHBA-AS1 | 0 | 0 | 0 | 1 | 0 | 0 | 0 |
| F3 | 0 | 0 | 0 | 1 | 0 | 0 | 0 |
| MIR5690 | 0 | 0 | 0 | 1 | 0 | 0 | 0 |
| CD244 | 0 | 0 | 0 | 1 | 0 | 0 | 0 |
| GZMM | 0 | 0 | 0 | 1 | 0 | 0 | 0 |
| MDGA1 | 0 | 0 | 0 | 1 | 0 | 0 | 0 |
| LINC01257 | 0 | 0 | 0 | 1 | 0 | 0 | 0 |
| CLEC18A | 0 | 0 | 0 | 1 | 0 | 0 | 0 |
| DLX6-AS1 | 0 | 0 | 0 | 1 | 0 | 0 | 0 |
| OLFM4 | 0 | 0 | 0 | 1 | 0 | 1 | 0 |
| LOC101929754 | 0 | 0 | 0 | 1 | 0 | 0 | 0 |
| NPY2R | 0 | 0 | 0 | 1 | 0 | 0 | 0 |
| MIR3619 | 0 | 0 | 0 | 1 | 0 | 0 | 0 |
| RAD21L1 | 0 | 0 | 0 | 1 | 0 | 0 | 0 |
| MIR1250 | 0 | 0 | 0 | 1 | 0 | 0 | 0 |
| SELE | 0 | 0 | 0 | 1 | 0 | 0 | 1 |
| PRSS35 | 0 | 0 | 0 | 1 | 0 | 0 | 0 |
| SFTPA1 | 0 | 0 | 0 | 1 | 0 | 0 | 0 |
| LOC100505912 | 0 | 0 | 0 | 1 | 0 | 0 | 0 |
| LINC01336 | 0 | 0 | 0 | 1 | 0 | 0 | 0 |
| TGFBR3L | 0 | 0 | 0 | 1 | 0 | 0 | 0 |
| LOC339529 | 0 | 0 | 0 | 1 | 0 | 0 | 0 |
| LOC101927043 | 0 | 0 | 0 | 1 | 0 | 0 | 0 |
| ANO3 | 0 | 0 | 0 | 1 | 0 | 0 | 0 |
| PRKCG | 0 | 0 | 0 | 1 | 0 | 0 | 0 |
| LOC101929151 | 0 | 0 | 0 | 1 | 0 | 0 | 0 |
| LINC01107 | 0 | 0 | 0 | 1 | 0 | 0 | 0 |
| SGCA | 0 | 0 | 0 | 1 | 0 | 0 | 0 |
| ABCA8 | 0 | 0 | 0 | 1 | 0 | 0 | 0 |
| SNORA104 | 0 | 0 | 0 | 1 | 0 | 0 | 0 |
| FAAHP1 | 0 | 0 | 0 | 1 | 0 | 0 | 0 |
| RAB3A | 0 | 0 | 0 | 1 | 0 | 0 | 0 |
| LOC100506136 | 0 | 0 | 0 | 1 | 0 | 0 | 0 |
| SNX10 | 0 | 0 | 0 | 1 | 0 | 0 | 0 |
| NGFR | 0 | 0 | 0 | 1 | 0 | 0 | 1 |
| CCR6 | 0 | 0 | 0 | 1 | 0 | 0 | 0 |
| SYT10 | 0 | 0 | 0 | 1 | 0 | 0 | 0 |
| DHH | 0 | 0 | 0 | 1 | 0 | 0 | 0 |
| ANKRD40 | 0 | 0 | 0 | 1 | 0 | 0 | 0 |
| MIR340 | 0 | 0 | 0 | 1 | 0 | 0 | 0 |
| C11orf87 | 0 | 0 | 0 | 1 | 0 | 0 | 0 |
| SMTN | 0 | 0 | 0 | 1 | 0 | 0 | 0 |
| MND1 | 0 | 0 | 0 | 1 | 0 | 0 | 0 |
| IL33 | 0 | 0 | 0 | 1 | 0 | 0 | 0 |
| LRRC46 | 0 | 0 | 0 | 1 | 0 | 0 | 0 |
| LGALS2 | 0 | 0 | 0 | 1 | 0 | 0 | 0 |
| DOCK5 | 0 | 0 | 0 | 1 | 0 | 0 | 0 |
| PRR34-AS1 | 0 | 0 | 0 | 1 | 0 | 0 | 0 |
| CXCR2 | 0 | 0 | 0 | 1 | 0 | 0 | 0 |
| DLX6 | 0 | 0 | 0 | 1 | 0 | 0 | 0 |
| PART1 | 0 | 0 | 0 | 1 | 0 | 0 | 0 |
| LOC101927139 | 0 | 0 | 0 | 1 | 0 | 0 | 0 |
| THRSP | 0 | 0 | 0 | 1 | 0 | 0 | 0 |
| SNORD114-21 | 0 | 0 | 0 | 1 | 0 | 0 | 0 |
| CLCA4 | 0 | 0 | 0 | 1 | 0 | 0 | 0 |
| CLMN | 0 | 0 | 0 | 1 | 0 | 0 | 1 |
| ADORA2A | 0 | 0 | 0 | 1 | 0 | 0 | 0 |
| CHRNA7 | 0 | 0 | 0 | 1 | 0 | 0 | 0 |
| TCP11 | 0 | 0 | 0 | 1 | 0 | 0 | 0 |
| HILS1 | 0 | 0 | 0 | 1 | 0 | 0 | 0 |
| BGLAP | 0 | 0 | 0 | 1 | 0 | 0 | 0 |
| BOK-AS1 | 0 | 0 | 0 | 1 | 0 | 0 | 0 |
| GADD45A | 0 | 0 | 0 | 1 | 0 | 0 | 0 |
| OVCH2 | 0 | 0 | 0 | 1 | 0 | 0 | 0 |
| RDH12 | 0 | 0 | 0 | 1 | 0 | 0 | 0 |
| SH2D3A | 0 | 0 | 0 | 1 | 0 | 0 | 1 |
| LOC100506274 | 0 | 0 | 0 | 1 | 0 | 0 | 0 |
| IRX2 | 0 | 0 | 0 | 1 | 0 | 0 | 0 |
| GMNC | 0 | 0 | 0 | 1 | 0 | 0 | 0 |
| MUC3A | 0 | 0 | 0 | 1 | 0 | 0 | 1 |
| KCNV1 | 0 | 0 | 0 | 1 | 0 | 0 | 0 |
| MIR2467 | 0 | 0 | 0 | 1 | 0 | 0 | 1 |
| LMNTD2 | 0 | 0 | 0 | 1 | 0 | 0 | 0 |
| FAM21EP | 0 | 0 | 0 | 1 | 0 | 0 | 0 |
| SALRNA2 | 0 | 0 | 0 | 1 | 0 | 0 | 0 |
| FOXD2 | 0 | 0 | 0 | 1 | 0 | 0 | 0 |
| C11orf94 | 0 | 0 | 0 | 1 | 0 | 0 | 0 |
| LINC00323 | 0 | 0 | 0 | 1 | 0 | 0 | 0 |
| BMP7-AS1 | 0 | 0 | 0 | 1 | 0 | 0 | 0 |
| PRTN3 | 0 | 0 | 0 | 1 | 0 | 0 | 0 |
| GABRG2 | 0 | 0 | 0 | 1 | 0 | 0 | 0 |
| SLC16A6 | 0 | 0 | 0 | 1 | 0 | 0 | 0 |
| LOC108783645 | 0 | 0 | 0 | 1 | 0 | 0 | 0 |
| FOXD1 | 0 | 0 | 0 | 1 | 0 | 0 | 0 |
| SPDEF | 0 | 0 | 0 | 1 | 0 | 0 | 0 |
| LINC00484 | 0 | 0 | 0 | 1 | 0 | 0 | 0 |
| SLC30A3 | 0 | 0 | 0 | 1 | 0 | 0 | 0 |
| PIK3C2G | 0 | 0 | 0 | 1 | 0 | 0 | 0 |
| LOC101928766 | 0 | 0 | 0 | 1 | 0 | 0 | 0 |
| CPAMD8 | 0 | 0 | 0 | 1 | 0 | 0 | 0 |
| NLRP14 | 0 | 0 | 0 | 1 | 0 | 0 | 0 |
| LINC01476 | 0 | 0 | 0 | 1 | 0 | 0 | 0 |
| RND1 | 0 | 0 | 0 | 1 | 0 | 0 | 0 |
| FMO3 | 0 | 0 | 0 | 1 | 0 | 0 | 1 |
| LOC100288866 | 0 | 0 | 0 | 1 | 0 | 0 | 0 |
| LOC101927406 | 0 | 0 | 0 | 1 | 0 | 0 | 0 |
| TP73 | 0 | 0 | 0 | 1 | 0 | 0 | 0 |
| LINC01119 | 0 | 0 | 0 | 1 | 0 | 0 | 0 |
| C1QL3 | 0 | 0 | 0 | 1 | 0 | 0 | 0 |
| FIBCD1 | 0 | 0 | 0 | 1 | 0 | 0 | 0 |
| CLDN9 | 0 | 0 | 0 | 1 | 0 | 0 | 0 |
| LOC105377448 | 0 | 0 | 0 | 1 | 0 | 0 | 0 |
| UHRF1 | 0 | 0 | 0 | 1 | 0 | 0 | 0 |
| LINC01296 | 0 | 0 | 0 | 1 | 0 | 0 | 0 |
| ROR1-AS1 | 0 | 0 | 0 | 1 | 0 | 0 | 0 |
| TSG1 | 0 | 0 | 0 | 1 | 0 | 0 | 0 |
| GRP | 0 | 0 | 0 | 1 | 0 | 0 | 0 |
| NEFL | 0 | 0 | 0 | 1 | 0 | 0 | 0 |
| FFAR4 | 0 | 0 | 0 | 1 | 0 | 0 | 0 |
| CHGB | 0 | 0 | 0 | 1 | 0 | 0 | 0 |
| HPN-AS1 | 0 | 0 | 0 | 1 | 0 | 0 | 0 |
| FERMT1 | 0 | 0 | 0 | 1 | 0 | 0 | 0 |
| NEK2 | 0 | 0 | 0 | 1 | 1 | 0 | 0 |
| LOC101929473 | 0 | 0 | 0 | 1 | 0 | 0 | 0 |
| ANLN | 0 | 0 | 0 | 1 | 0 | 0 | 0 |
| GSN-AS1 | 0 | 0 | 0 | 1 | 0 | 0 | 0 |
| HPN | 0 | 0 | 0 | 1 | 0 | 0 | 0 |
| FHL5 | 0 | 0 | 0 | 1 | 0 | 0 | 0 |
| CACNG1 | 0 | 0 | 0 | 1 | 0 | 0 | 0 |
| FAM86B3P | 0 | 0 | 0 | 1 | 0 | 0 | 0 |
| TMEM215 | 0 | 0 | 0 | 1 | 0 | 0 | 0 |
| MIR1914 | 0 | 0 | 0 | 1 | 0 | 0 | 0 |
| LOC149684 | 0 | 0 | 0 | 1 | 0 | 0 | 0 |
| VGLL3 | 0 | 0 | 0 | 1 | 0 | 0 | 1 |
| SOX8 | 0 | 0 | 0 | 1 | 0 | 0 | 0 |
| ZNF90 | 0 | 0 | 0 | 1 | 0 | 0 | 1 |
| TMC6 | 0 | 0 | 0 | 1 | 0 | 0 | 0 |
| HIPK2 | 0 | 0 | 0 | 1 | 0 | 0 | 1 |
| LINC00567 | 0 | 0 | 0 | 1 | 0 | 0 | 0 |
| MAGI1-AS1 | 0 | 0 | 0 | 1 | 0 | 0 | 0 |
| TINAG | 0 | 0 | 0 | 1 | 0 | 0 | 0 |
| LOC100505635 | 0 | 0 | 0 | 1 | 0 | 0 | 0 |
| NSF | 0 | 0 | 0 | 1 | 0 | 0 | 0 |
| LOC101927410 | 0 | 0 | 0 | 1 | 0 | 0 | 0 |
| LOC100507616 | 0 | 0 | 0 | 1 | 0 | 0 | 0 |
| C10orf128 | 0 | 0 | 0 | 1 | 0 | 0 | 0 |
| DKFZP434K028 | 0 | 0 | 0 | 1 | 0 | 0 | 0 |
| SLC26A5 | 0 | 0 | 0 | 1 | 0 | 0 | 0 |
| INHA | 0 | 0 | 0 | 1 | 0 | 0 | 0 |
| ALG1L | 0 | 0 | 0 | 1 | 0 | 0 | 0 |
| KRT19 | 0 | 0 | 0 | 1 | 0 | 0 | 1 |
| TFCP2L1 | 0 | 0 | 0 | 1 | 0 | 0 | 1 |
| ELMO1-AS1 | 0 | 0 | 0 | 1 | 0 | 0 | 0 |
| C5orf38 | 0 | 0 | 0 | 1 | 0 | 0 | 0 |
| ANKRD22 | 0 | 0 | 0 | 1 | 0 | 0 | 1 |
| SHISA2 | 0 | 0 | 0 | 1 | 1 | 0 | 0 |
| FAM19A4 | 0 | 0 | 0 | 1 | 0 | 0 | 0 |
| ACP7 | 0 | 0 | 0 | 1 | 0 | 0 | 0 |
| C10orf90 | 0 | 0 | 0 | 1 | 0 | 0 | 0 |
| LOC100128554 | 0 | 0 | 0 | 1 | 0 | 0 | 0 |
| C7orf61 | 0 | 0 | 0 | 1 | 0 | 0 | 0 |
| CLCN1 | 0 | 0 | 0 | 1 | 0 | 0 | 0 |
| LOC441052 | 0 | 0 | 0 | 1 | 0 | 0 | 0 |
| KRT81 | 0 | 0 | 0 | 1 | 0 | 0 | 0 |
| LOC642943 | 0 | 0 | 0 | 1 | 0 | 0 | 0 |
| LOC400997 | 0 | 0 | 0 | 1 | 0 | 0 | 0 |
| SGK1 | 0 | 0 | 0 | 1 | 1 | 0 | 0 |
| TPTEP1 | 0 | 0 | 0 | 1 | 0 | 0 | 1 |
| DEPDC1 | 0 | 0 | 0 | 1 | 0 | 0 | 0 |
| VIP | 0 | 0 | 0 | 1 | 0 | 0 | 0 |
| LINCR-0003 | 0 | 0 | 0 | 1 | 0 | 0 | 0 |
| PROK2 | 0 | 0 | 0 | 1 | 0 | 0 | 0 |
| IL16 | 0 | 0 | 0 | 1 | 0 | 0 | 0 |
| LOC388780 | 0 | 0 | 0 | 1 | 0 | 0 | 0 |
| IRX1 | 0 | 0 | 0 | 1 | 0 | 0 | 0 |
| LOC105376360 | 0 | 0 | 0 | 1 | 0 | 0 | 0 |
| LOC100507472 | 0 | 0 | 0 | 1 | 0 | 0 | 0 |
| ADAMTS2 | 0 | 0 | 0 | 1 | 0 | 0 | 0 |
| SLC45A3 | 0 | 0 | 0 | 1 | 0 | 0 | 0 |
| PTER | 0 | 0 | 0 | 1 | 0 | 0 | 0 |
| LOC284933 | 0 | 0 | 0 | 1 | 0 | 0 | 0 |
| MROH5 | 0 | 0 | 0 | 1 | 0 | 0 | 0 |
| LINC01108 | 0 | 0 | 0 | 1 | 0 | 0 | 0 |
| IFNL2 | 0 | 0 | 0 | 1 | 0 | 0 | 0 |
| SPINT1 | 0 | 0 | 0 | 1 | 0 | 0 | 0 |
| CHADL | 0 | 0 | 0 | 1 | 0 | 0 | 0 |
| CCDC189 | 0 | 0 | 0 | 1 | 0 | 0 | 0 |
| ASB9P1 | 0 | 0 | 0 | 1 | 0 | 0 | 0 |
| H1FNT | 0 | 0 | 0 | 1 | 0 | 0 | 0 |
| MUC16 | 0 | 0 | 0 | 1 | 0 | 0 | 1 |
| CTD-2201E9.1 | 0 | 0 | 0 | 1 | 0 | 0 | 0 |
| CDR1 | 0 | 0 | 0 | 1 | 0 | 0 | 0 |
| MIR3612 | 0 | 0 | 0 | 1 | 0 | 0 | 0 |
| DLX5 | 0 | 0 | 0 | 1 | 0 | 0 | 0 |
| PNOC | 0 | 0 | 0 | 1 | 0 | 0 | 0 |
| IGF2 | 0 | 0 | 0 | 1 | 0 | 0 | 0 |
| ENPP6 | 0 | 0 | 0 | 1 | 0 | 0 | 0 |
| AZGP1 | 0 | 0 | 0 | 1 | 0 | 0 | 1 |
| RPLP0P2 | 0 | 0 | 0 | 1 | 0 | 0 | 0 |
| KIF2B | 0 | 0 | 0 | 1 | 0 | 0 | 0 |
| ZFPM2-AS1 | 0 | 0 | 0 | 1 | 0 | 0 | 0 |
| LOC100507144 | 0 | 0 | 0 | 1 | 0 | 0 | 0 |
| PCDH8 | 0 | 0 | 0 | 1 | 0 | 0 | 0 |
| SLED1 | 0 | 0 | 0 | 1 | 0 | 0 | 1 |
| LCN10 | 0 | 0 | 0 | 1 | 0 | 0 | 0 |
| LMNTD1 | 0 | 0 | 0 | 1 | 0 | 0 | 0 |
| SELENOP | 0 | 0 | 0 | 1 | 0 | 0 | 1 |
| LINC01470 | 0 | 0 | 0 | 1 | 0 | 0 | 0 |
| SFRP1 | 0 | 0 | 0 | 1 | 0 | 0 | 1 |
| CLDN7 | 0 | 0 | 0 | 1 | 0 | 0 | 0 |
| MYBPC2 | 0 | 0 | 0 | 1 | 0 | 0 | 0 |
| CBX3P2 | 0 | 0 | 0 | 1 | 0 | 0 | 0 |
| LINC01361 | 0 | 0 | 0 | 1 | 0 | 0 | 0 |
| TESPA1 | 0 | 0 | 0 | 1 | 0 | 0 | 0 |
| G0S2 | 0 | 0 | 0 | 1 | 0 | 0 | 1 |
| HECTD2-AS1 | 0 | 0 | 0 | 1 | 0 | 0 | 0 |
| ADORA2A-AS1 | 0 | 0 | 0 | 1 | 0 | 0 | 1 |
| LINC00390 | 0 | 0 | 0 | 1 | 0 | 0 | 0 |
| LINC01378 | 0 | 0 | 0 | 1 | 0 | 0 | 0 |
| LOC101929719 | 0 | 0 | 0 | 1 | 0 | 0 | 0 |
| RAB27B | 0 | 0 | 0 | 1 | 0 | 0 | 0 |
| RASL11B | 0 | 0 | 0 | 1 | 0 | 0 | 0 |
| SLC30A8 | 0 | 0 | 0 | 1 | 0 | 0 | 0 |
| ZNF341-AS1 | 0 | 0 | 0 | 1 | 0 | 0 | 0 |
| LMX1B | 0 | 0 | 0 | 1 | 0 | 0 | 0 |
| IP6K3 | 0 | 0 | 0 | 1 | 0 | 0 | 1 |
| LCN2 | 0 | 0 | 0 | 1 | 0 | 1 | 1 |
| RNASE2 | 0 | 0 | 0 | 1 | 0 | 0 | 0 |
| TAC3 | 0 | 0 | 0 | 1 | 0 | 0 | 0 |
| C17orf102 | 0 | 0 | 0 | 1 | 0 | 0 | 0 |
| LOC101927844 | 0 | 0 | 0 | 1 | 0 | 0 | 0 |
| TPH1 | 0 | 0 | 0 | 1 | 0 | 0 | 0 |
| EGR3 | 0 | 0 | 0 | 1 | 0 | 0 | 0 |
| TPO | 0 | 0 | 0 | 1 | 0 | 0 | 0 |
| LCN15 | 0 | 0 | 0 | 1 | 0 | 0 | 0 |
| SPTBN5 | 0 | 0 | 0 | 1 | 0 | 0 | 1 |
| TSPAN10 | 0 | 0 | 0 | 1 | 0 | 0 | 0 |
| VNN1 | 0 | 0 | 0 | 1 | 0 | 0 | 1 |
| MIR4534 | 0 | 0 | 0 | 1 | 0 | 0 | 0 |
| PKHD1L1 | 0 | 0 | 0 | 1 | 0 | 0 | 1 |
| LENEP | 0 | 0 | 0 | 1 | 0 | 0 | 0 |
| SLC5A11 | 0 | 0 | 0 | 1 | 0 | 0 | 0 |
| MIR3139 | 0 | 0 | 0 | 1 | 0 | 0 | 0 |
| DLX4 | 0 | 0 | 0 | 1 | 0 | 0 | 0 |
| IQGAP3 | 0 | 0 | 0 | 1 | 0 | 0 | 0 |
| LRP2 | 0 | 0 | 0 | 1 | 0 | 0 | 0 |
| RTKN | 0 | 0 | 0 | 1 | 0 | 0 | 0 |
| TPH2 | 0 | 0 | 0 | 1 | 0 | 0 | 0 |
| KLHDC7B | 0 | 0 | 0 | 1 | 0 | 0 | 0 |
| LOC101059915 | 0 | 0 | 0 | 1 | 0 | 0 | 0 |
| LINC01512 | 0 | 0 | 0 | 1 | 0 | 0 | 0 |
| HAPLN2 | 0 | 0 | 0 | 1 | 0 | 0 | 0 |
| OR2D3 | 0 | 0 | 0 | 1 | 0 | 0 | 0 |
| CR1 | 0 | 0 | 0 | 1 | 0 | 0 | 1 |
| TNN | 0 | 0 | 0 | 1 | 0 | 0 | 0 |
| LINC00926 | 0 | 0 | 0 | 1 | 0 | 0 | 0 |
| ADGRG5 | 0 | 0 | 0 | 1 | 0 | 0 | 0 |
| LAMA5-AS1 | 0 | 0 | 0 | 1 | 0 | 0 | 0 |
| NKX6-2 | 0 | 0 | 0 | 1 | 0 | 0 | 0 |
| LOC340090 | 0 | 0 | 0 | 1 | 0 | 0 | 0 |
| LRRC63 | 0 | 0 | 0 | 1 | 0 | 0 | 0 |
| MIR657 | 0 | 0 | 0 | 1 | 0 | 0 | 0 |
| F2 | 0 | 0 | 0 | 1 | 0 | 0 | 0 |
| ZIC3 | 0 | 0 | 0 | 1 | 0 | 0 | 0 |
| GRK7 | 0 | 0 | 0 | 1 | 0 | 0 | 0 |
| SCUBE1 | 0 | 0 | 0 | 1 | 0 | 0 | 0 |
| GPA33 | 0 | 0 | 0 | 1 | 0 | 1 | 0 |
| CCKAR | 0 | 0 | 0 | 1 | 0 | 0 | 0 |
| LOC100129940 | 0 | 0 | 0 | 1 | 0 | 0 | 0 |
| C4orf26 | 0 | 0 | 0 | 1 | 1 | 0 | 0 |
| SLN | 0 | 0 | 0 | 1 | 0 | 0 | 0 |
| GDNF | 0 | 0 | 0 | 1 | 0 | 0 | 0 |
| LGR5 | 0 | 0 | 0 | 1 | 0 | 0 | 0 |
| MIR4296 | 0 | 0 | 0 | 1 | 0 | 0 | 0 |
| ZBTB46-AS1 | 0 | 0 | 0 | 1 | 0 | 0 | 0 |
| PTPRQ | 0 | 0 | 0 | 1 | 0 | 0 | 1 |
| LOC105371789 | 0 | 0 | 0 | 1 | 0 | 0 | 0 |
| RAB3C | 0 | 0 | 0 | 1 | 0 | 0 | 1 |
| SFTPA2 | 0 | 0 | 0 | 1 | 0 | 0 | 1 |
| PTF1A | 0 | 0 | 0 | 1 | 0 | 0 | 0 |
| MIR4300HG | 0 | 0 | 0 | 1 | 0 | 0 | 0 |
| MIR4311 | 0 | 0 | 0 | 1 | 0 | 0 | 0 |
| LOC100506271 | 0 | 0 | 0 | 1 | 0 | 0 | 0 |
| C16orf90 | 0 | 0 | 0 | 1 | 0 | 0 | 0 |
| PRKXP1 | 0 | 0 | 0 | 1 | 0 | 0 | 0 |
| GDF2 | 0 | 0 | 0 | 1 | 0 | 0 | 0 |
| CA9 | 0 | 0 | 0 | 1 | 0 | 0 | 0 |
| LINC01088 | 0 | 0 | 0 | 1 | 0 | 0 | 0 |
| PRIMA1 | 0 | 0 | 0 | 1 | 0 | 0 | 0 |
| ST18 | 0 | 0 | 0 | 1 | 0 | 0 | 0 |
| OR7C1 | 0 | 0 | 0 | 1 | 0 | 0 | 0 |
| LOC100287072 | 0 | 0 | 0 | 1 | 0 | 0 | 0 |
| OR14I1 | 0 | 0 | 0 | 1 | 0 | 0 | 0 |
| CD22 | 0 | 0 | 0 | 1 | 0 | 0 | 0 |
| LINC00514 | 0 | 0 | 0 | 1 | 0 | 0 | 0 |
| SNRPD2P2 | 0 | 0 | 0 | 1 | 0 | 0 | 0 |
| LINC00937 | 0 | 0 | 0 | 1 | 0 | 1 | 0 |
| KANK4 | 0 | 0 | 0 | 1 | 0 | 0 | 0 |
| FAM95A | 0 | 0 | 0 | 1 | 0 | 0 | 0 |
| ARL5C | 0 | 0 | 0 | 1 | 0 | 0 | 0 |
| PIEZO2 | 0 | 0 | 0 | 1 | 0 | 0 | 0 |
| MCCD1 | 0 | 0 | 0 | 1 | 0 | 0 | 0 |
| FOXR1 | 0 | 0 | 0 | 1 | 0 | 0 | 0 |
| PCSK6-AS1 | 0 | 0 | 0 | 1 | 0 | 0 | 0 |
| LOC100130331 | 0 | 0 | 0 | 1 | 0 | 0 | 0 |
| ADAM29 | 0 | 0 | 0 | 1 | 0 | 0 | 0 |
| DUSP4 | 0 | 0 | 0 | 1 | 0 | 0 | 0 |
| PCSK6 | 0 | 0 | 0 | 1 | 0 | 0 | 1 |
| C1orf195 | 0 | 0 | 0 | 1 | 0 | 0 | 0 |
| MIR6758 | 0 | 0 | 0 | 1 | 0 | 0 | 0 |
| FAM163A | 0 | 0 | 0 | 1 | 0 | 0 | 0 |
| CT55 | 0 | 0 | 0 | 1 | 0 | 0 | 0 |
| LINC00664 | 0 | 0 | 0 | 1 | 0 | 0 | 0 |
| TMEM235 | 0 | 0 | 0 | 1 | 0 | 0 | 0 |
| LOC101929613 | 0 | 0 | 0 | 1 | 0 | 0 | 0 |
| MIR6871 | 0 | 0 | 0 | 1 | 0 | 0 | 0 |
| LINC00320 | 0 | 0 | 0 | 1 | 0 | 0 | 0 |
| GFRA3 | 0 | 0 | 0 | 1 | 0 | 0 | 0 |
| MIR4479 | 0 | 0 | 0 | 1 | 0 | 0 | 0 |
| LINC01219 | 0 | 0 | 0 | 1 | 0 | 0 | 0 |
| MEIS1-AS2 | 0 | 0 | 0 | 1 | 0 | 0 | 0 |
| LDHAL6B | 0 | 0 | 0 | 1 | 0 | 0 | 0 |
| KCNJ15 | 0 | 0 | 0 | 1 | 0 | 0 | 1 |
| LOC101928738 | 0 | 0 | 0 | 1 | 0 | 0 | 0 |
| ASB2 | 0 | 0 | 0 | 1 | 0 | 0 | 0 |
| LOC102723895 | 0 | 0 | 0 | 1 | 0 | 0 | 0 |
| LINC01117 | 0 | 0 | 0 | 1 | 0 | 0 | 0 |
| ELANE | 0 | 0 | 0 | 1 | 0 | 0 | 0 |
| FAM95C | 0 | 0 | 0 | 1 | 0 | 0 | 0 |
| CALHM3 | 0 | 0 | 0 | 1 | 0 | 0 | 0 |
| CHRM5 | 0 | 0 | 0 | 1 | 0 | 0 | 0 |
| FERD3L | 0 | 0 | 0 | 1 | 0 | 0 | 0 |
| DHRS2 | 0 | 0 | 0 | 1 | 0 | 0 | 0 |
| NFE2 | 0 | 0 | 0 | 1 | 0 | 1 | 0 |
| C1orf94 | 0 | 0 | 0 | 1 | 0 | 0 | 0 |
| JAML | 0 | 0 | 0 | 1 | 0 | 0 | 0 |
| SNORD128 | 0 | 0 | 0 | 1 | 0 | 0 | 0 |
| LPO | 0 | 0 | 0 | 1 | 0 | 0 | 0 |
| MIR194-2HG | 0 | 0 | 0 | 1 | 0 | 0 | 0 |
| LINC00504 | 0 | 0 | 0 | 1 | 0 | 0 | 0 |
| BAAT | 0 | 0 | 0 | 1 | 0 | 0 | 1 |
| LOC100507388 | 0 | 0 | 0 | 1 | 0 | 0 | 0 |
| LOC440982 | 0 | 0 | 0 | 1 | 0 | 0 | 0 |
| RASGRP3 | 0 | 0 | 0 | 1 | 0 | 0 | 1 |
| LINC00609 | 0 | 0 | 0 | 1 | 0 | 0 | 0 |
| LOC101927960 | 0 | 0 | 0 | 1 | 0 | 0 | 0 |
| LINC01331 | 0 | 0 | 0 | 1 | 0 | 0 | 0 |
| LOC101927168 | 0 | 0 | 0 | 1 | 0 | 0 | 0 |
| NMRK2 | 0 | 0 | 0 | 1 | 0 | 0 | 0 |
| HOXD1 | 0 | 0 | 0 | 1 | 0 | 0 | 0 |
| LOC100130698 | 0 | 0 | 0 | 1 | 0 | 0 | 0 |
| CLEC18B | 0 | 0 | 0 | 1 | 0 | 0 | 0 |
| CEL | 0 | 0 | 0 | 1 | 0 | 0 | 0 |
| ANXA2P3 | 0 | 0 | 0 | 1 | 0 | 0 | 0 |
| LOC101929284 | 0 | 0 | 0 | 1 | 0 | 0 | 0 |
| DIO3OS | 0 | 0 | 0 | 1 | 0 | 0 | 0 |
| LOC101929341 | 0 | 0 | 0 | 1 | 0 | 0 | 0 |
| FAM151A | 0 | 0 | 0 | 1 | 0 | 0 | 0 |
| FAM66E | 0 | 0 | 0 | 1 | 0 | 0 | 0 |
| NXPH2 | 0 | 0 | 0 | 1 | 0 | 0 | 0 |
| LINC01494 | 0 | 0 | 0 | 1 | 0 | 0 | 0 |
| LOC100130370 | 0 | 0 | 0 | 1 | 0 | 0 | 0 |
| CBLN4 | 0 | 0 | 0 | 1 | 0 | 0 | 0 |
| LINC00398 | 0 | 0 | 0 | 1 | 0 | 0 | 0 |
| GIPR | 0 | 0 | 0 | 1 | 0 | 0 | 1 |
| C21orf91-OT1 | 0 | 0 | 0 | 1 | 0 | 0 | 0 |
| NMUR2 | 0 | 0 | 0 | 1 | 0 | 0 | 0 |
| BCRP3 | 0 | 0 | 0 | 1 | 0 | 0 | 0 |
| TSPAN18 | 0 | 0 | 0 | 1 | 0 | 0 | 0 |
| RORC | 0 | 0 | 0 | 1 | 0 | 0 | 0 |
| CD300LG | 0 | 0 | 0 | 1 | 0 | 0 | 0 |
| HIST1H1T | 0 | 0 | 0 | 1 | 0 | 0 | 0 |
| MUC5AC | 0 | 0 | 0 | 1 | 0 | 0 | 0 |
| SLC17A6 | 0 | 0 | 0 | 1 | 0 | 0 | 0 |
| KRT8P41 | 0 | 0 | 0 | 1 | 0 | 0 | 0 |
| CD38 | 0 | 0 | 0 | 1 | 0 | 1 | 0 |
| MIR4513 | 0 | 0 | 0 | 1 | 0 | 0 | 0 |
| MTRNR2L10 | 0 | 0 | 0 | 0 | 1 | 0 | 0 |
| INMT | 0 | 0 | 0 | 0 | 1 | 0 | 1 |
| PPIEL | 0 | 0 | 0 | 0 | 1 | 0 | 0 |
| RAB3B | 0 | 0 | 0 | 0 | 1 | 0 | 1 |
| IKZF3 | 0 | 0 | 0 | 0 | 1 | 0 | 0 |
| GATM-AS1 | 0 | 0 | 0 | 0 | 1 | 0 | 0 |
| PDE6A | 0 | 0 | 0 | 0 | 1 | 0 | 0 |
| MREG | 0 | 0 | 0 | 0 | 1 | 0 | 0 |
| IDS | 0 | 0 | 0 | 0 | 1 | 0 | 0 |
| LINC00649 | 0 | 0 | 0 | 0 | 1 | 0 | 0 |
| METTL21A | 0 | 0 | 0 | 0 | 1 | 0 | 0 |
| SLC43A2 | 0 | 0 | 0 | 0 | 1 | 0 | 0 |
| EEF2K | 0 | 0 | 0 | 0 | 1 | 0 | 1 |
| FXN | 0 | 0 | 0 | 0 | 1 | 0 | 1 |
| ACBD7 | 0 | 0 | 0 | 0 | 1 | 0 | 1 |
| IFNLR1 | 0 | 0 | 0 | 0 | 1 | 0 | 0 |
| ZNF483 | 0 | 0 | 0 | 0 | 1 | 0 | 1 |
| FGF5 | 0 | 0 | 0 | 0 | 1 | 0 | 0 |
| FBLIM1 | 0 | 0 | 0 | 0 | 1 | 0 | 0 |
| ATP5E | 0 | 0 | 0 | 0 | 1 | 0 | 0 |
| MTRNR2L2 | 0 | 0 | 0 | 0 | 1 | 0 | 0 |
| GNG4 | 0 | 0 | 0 | 0 | 1 | 0 | 0 |
| LOC284260 | 0 | 0 | 0 | 0 | 1 | 0 | 0 |
| CCL5 | 0 | 0 | 0 | 0 | 1 | 0 | 0 |
| GLIPR1L2 | 0 | 0 | 0 | 0 | 1 | 0 | 0 |
| PRELP | 0 | 0 | 0 | 0 | 1 | 0 | 1 |
| SIX4 | 0 | 0 | 0 | 0 | 1 | 0 | 0 |
| CSDE1 | 0 | 0 | 0 | 0 | 1 | 0 | 0 |
| C21orf62 | 0 | 0 | 0 | 0 | 1 | 0 | 0 |
| TMEM154 | 0 | 0 | 0 | 0 | 1 | 0 | 0 |
| LOC286186 | 0 | 0 | 0 | 0 | 1 | 0 | 0 |
| CACNG8 | 0 | 0 | 0 | 0 | 1 | 0 | 0 |
| VSIG1 | 0 | 0 | 0 | 0 | 1 | 0 | 0 |
| MOG | 0 | 0 | 0 | 0 | 1 | 0 | 0 |
| UBE2Q2P1 | 0 | 0 | 0 | 0 | 1 | 0 | 0 |
| CCDC122 | 0 | 0 | 0 | 0 | 1 | 0 | 0 |
| PPIL6 | 0 | 0 | 0 | 0 | 1 | 0 | 1 |
| ELMOD1 | 0 | 0 | 0 | 0 | 1 | 0 | 0 |
| CYP4V2 | 0 | 0 | 0 | 0 | 1 | 0 | 1 |
| NXN | 0 | 0 | 0 | 0 | 1 | 0 | 1 |
| ZFP42 | 0 | 0 | 0 | 0 | 1 | 0 | 0 |
| SPRED1 | 0 | 0 | 0 | 0 | 1 | 0 | 1 |
| ZYG11A | 0 | 0 | 0 | 0 | 1 | 0 | 0 |
| TBXA2R | 0 | 0 | 0 | 0 | 1 | 0 | 0 |
| KIAA1456 | 0 | 0 | 0 | 0 | 1 | 0 | 0 |
| ZNF665 | 0 | 0 | 0 | 0 | 1 | 0 | 0 |
| MTRNR2L4 | 0 | 0 | 0 | 0 | 1 | 0 | 0 |
| PJA2 | 0 | 0 | 0 | 0 | 1 | 0 | 0 |
| LOC283335 | 0 | 0 | 0 | 0 | 1 | 0 | 0 |
| HTRA4 | 0 | 0 | 0 | 0 | 1 | 0 | 0 |
| IRF2BPL | 0 | 0 | 0 | 0 | 1 | 0 | 0 |
| LOC100287314 | 0 | 0 | 0 | 0 | 1 | 0 | 0 |
| FKBP14 | 0 | 0 | 0 | 0 | 1 | 0 | 0 |
| LOC90834 | 0 | 0 | 0 | 0 | 1 | 0 | 0 |
| LOC100129269 | 0 | 0 | 0 | 0 | 1 | 0 | 0 |
| AP1S3 | 0 | 0 | 0 | 0 | 1 | 0 | 0 |
| LOC100288069 | 0 | 0 | 0 | 0 | 1 | 0 | 0 |
| BVES | 0 | 0 | 0 | 0 | 1 | 0 | 0 |
| CEP41 | 0 | 0 | 0 | 0 | 1 | 0 | 0 |
| PTPN14 | 0 | 0 | 0 | 0 | 1 | 0 | 1 |
| ZNF713 | 0 | 0 | 0 | 0 | 1 | 0 | 1 |
| AFMID | 0 | 0 | 0 | 0 | 1 | 0 | 0 |
| OR7D2 | 0 | 0 | 0 | 0 | 1 | 0 | 0 |
| MAP1LC3C | 0 | 0 | 0 | 0 | 1 | 0 | 0 |
| LOC100128288 | 0 | 0 | 0 | 0 | 1 | 0 | 0 |
| ZNF793 | 0 | 0 | 0 | 0 | 1 | 1 | 0 |
| PTK6 | 0 | 0 | 0 | 0 | 1 | 0 | 0 |
| NEK5 | 0 | 0 | 0 | 0 | 1 | 0 | 0 |
| TMEM213 | 0 | 0 | 0 | 0 | 1 | 0 | 0 |
| ATCAY | 0 | 0 | 0 | 0 | 1 | 0 | 0 |
| ATP5I | 0 | 0 | 0 | 0 | 1 | 0 | 0 |
| C1orf210 | 0 | 0 | 0 | 0 | 1 | 0 | 0 |
| MAPRE1 | 0 | 0 | 0 | 0 | 1 | 0 | 0 |
| LRRN4CL | 0 | 0 | 0 | 0 | 1 | 0 | 0 |
| LOC100506085 | 0 | 0 | 0 | 0 | 1 | 0 | 0 |
| METTL8 | 0 | 0 | 0 | 0 | 1 | 0 | 0 |
| MTRNR2L3 | 0 | 0 | 0 | 0 | 1 | 0 | 0 |
| RNF207 | 0 | 0 | 0 | 0 | 1 | 0 | 0 |
| RABL5 | 0 | 0 | 0 | 0 | 1 | 0 | 0 |
| TMEM236 | 0 | 0 | 0 | 0 | 1 | 0 | 0 |
| JPX | 0 | 0 | 0 | 0 | 1 | 0 | 0 |
| ZNF716 | 0 | 0 | 0 | 0 | 1 | 0 | 0 |
| TFDP2 | 0 | 0 | 0 | 0 | 1 | 0 | 1 |
| NCMAP | 0 | 0 | 0 | 0 | 1 | 0 | 1 |
| RAB42 | 0 | 0 | 0 | 0 | 1 | 0 | 0 |
| ZNF818P | 0 | 0 | 0 | 0 | 1 | 0 | 0 |
| ZC3H12D | 0 | 0 | 0 | 0 | 1 | 0 | 0 |
| LOC440300 | 0 | 0 | 0 | 0 | 1 | 0 | 0 |
| PXMP4 | 0 | 0 | 0 | 0 | 1 | 0 | 1 |
| PAPL | 0 | 0 | 0 | 0 | 1 | 0 | 0 |
| ZFAS1 | 0 | 0 | 0 | 0 | 1 | 0 | 0 |
| PNPO | 0 | 0 | 0 | 0 | 1 | 0 | 1 |
| PTPRG-AS1 | 0 | 0 | 0 | 0 | 1 | 0 | 0 |
| WDR1 | 0 | 0 | 0 | 0 | 1 | 0 | 0 |
| SEPT14 | 0 | 0 | 0 | 0 | 1 | 0 | 0 |
| BCAP31 | 0 | 0 | 0 | 0 | 1 | 0 | 0 |
| ANKRD16 | 0 | 0 | 0 | 0 | 1 | 0 | 0 |
| SCD5 | 0 | 0 | 0 | 0 | 1 | 0 | 1 |
| C17orf75 | 0 | 0 | 0 | 0 | 1 | 0 | 0 |
| FAM227A | 0 | 0 | 0 | 0 | 1 | 0 | 0 |
| PNMA2 | 0 | 0 | 0 | 0 | 1 | 0 | 1 |
| ZNF667 | 0 | 0 | 0 | 0 | 1 | 0 | 0 |
| EMP2 | 0 | 0 | 0 | 0 | 1 | 0 | 1 |
| RFPL1-AS1 | 0 | 0 | 0 | 0 | 1 | 0 | 0 |
| SCAI | 0 | 0 | 0 | 0 | 1 | 0 | 1 |
| KIAA1324 | 0 | 0 | 0 | 0 | 1 | 0 | 0 |
| PACS2 | 0 | 0 | 0 | 0 | 1 | 0 | 0 |
| EXPH5 | 0 | 0 | 0 | 0 | 1 | 0 | 1 |
| DYNC1LI1 | 0 | 0 | 0 | 0 | 1 | 0 | 0 |
| DNAL1 | 0 | 0 | 0 | 0 | 1 | 0 | 1 |
| TRIM65 | 0 | 0 | 0 | 0 | 1 | 0 | 1 |
| PRND | 0 | 0 | 0 | 0 | 1 | 0 | 0 |
| CHRNB1 | 0 | 0 | 0 | 0 | 1 | 0 | 1 |
| ZNF492 | 0 | 0 | 0 | 0 | 1 | 0 | 0 |
| PGM5P2 | 0 | 0 | 0 | 0 | 1 | 1 | 0 |
| BRIP1 | 0 | 0 | 0 | 0 | 1 | 0 | 0 |
| IBA57 | 0 | 0 | 0 | 0 | 1 | 0 | 1 |
| POU5F1 | 0 | 0 | 0 | 0 | 1 | 0 | 0 |
| SLC5A5 | 0 | 0 | 0 | 0 | 1 | 0 | 0 |
| GDPD1 | 0 | 0 | 0 | 0 | 1 | 0 | 1 |
| PDP2 | 0 | 0 | 0 | 0 | 1 | 0 | 0 |
| KCNJ5 | 0 | 0 | 0 | 0 | 1 | 0 | 1 |
| PLEKHH2 | 0 | 0 | 0 | 0 | 1 | 0 | 1 |
| LRRC2 | 0 | 0 | 0 | 0 | 1 | 0 | 0 |
| LOC284950 | 0 | 0 | 0 | 0 | 1 | 0 | 0 |
| C2orf91 | 0 | 0 | 0 | 0 | 1 | 0 | 0 |
| AK3 | 0 | 0 | 0 | 0 | 1 | 0 | 1 |
| GGT6 | 0 | 0 | 0 | 0 | 1 | 0 | 0 |
| SEC14L1 | 0 | 0 | 0 | 0 | 1 | 0 | 0 |
| PGAM5 | 0 | 0 | 0 | 0 | 1 | 0 | 0 |
| KIAA1875 | 0 | 0 | 0 | 0 | 1 | 0 | 0 |
| SPG21 | 0 | 0 | 0 | 0 | 1 | 0 | 0 |
| LOC100128338 | 0 | 0 | 0 | 0 | 1 | 0 | 0 |
| CYFIP2 | 0 | 0 | 0 | 0 | 1 | 0 | 0 |
| UBL5 | 0 | 0 | 0 | 0 | 1 | 0 | 1 |
| C1orf56 | 0 | 0 | 0 | 0 | 1 | 0 | 0 |
| CCNI | 0 | 0 | 0 | 0 | 1 | 1 | 0 |
| SGCB | 0 | 0 | 0 | 0 | 1 | 0 | 0 |
| ARHGEF26-AS1 | 0 | 0 | 0 | 0 | 1 | 0 | 0 |
| FPR2 | 0 | 0 | 0 | 0 | 1 | 0 | 0 |
| ARF1 | 0 | 0 | 0 | 0 | 1 | 0 | 0 |
| OPA3 | 0 | 0 | 0 | 0 | 1 | 0 | 0 |
| SLC4A8 | 0 | 0 | 0 | 0 | 1 | 0 | 1 |
| AARS2 | 0 | 0 | 0 | 0 | 1 | 0 | 0 |
| LOC283299 | 0 | 0 | 0 | 0 | 1 | 0 | 0 |
| LIN28A | 0 | 0 | 0 | 0 | 1 | 0 | 0 |
| MTRNR2L8 | 0 | 0 | 0 | 0 | 1 | 0 | 0 |
| TMEM136 | 0 | 0 | 0 | 0 | 1 | 0 | 0 |
| NR1H2 | 0 | 0 | 0 | 0 | 1 | 1 | 0 |
| MYLK3 | 0 | 0 | 0 | 0 | 1 | 0 | 0 |
| CA5B | 0 | 0 | 0 | 0 | 1 | 0 | 0 |
| C3orf72 | 0 | 0 | 0 | 0 | 1 | 0 | 0 |
| CRX | 0 | 0 | 0 | 0 | 1 | 0 | 0 |
| FAM73A | 0 | 0 | 0 | 0 | 1 | 0 | 0 |
| MANEAL | 0 | 0 | 0 | 0 | 1 | 0 | 0 |
| LINC00294 | 0 | 0 | 0 | 0 | 1 | 0 | 0 |
| WDR92 | 0 | 0 | 0 | 0 | 1 | 0 | 0 |
| FOXK1 | 0 | 0 | 0 | 0 | 1 | 0 | 0 |
| TSTD3 | 0 | 0 | 0 | 0 | 1 | 0 | 0 |
| SIGLEC8 | 0 | 0 | 0 | 0 | 1 | 0 | 0 |
| LOC728606 | 0 | 0 | 0 | 0 | 1 | 0 | 0 |
| ZNF714 | 0 | 0 | 0 | 0 | 1 | 0 | 1 |
| WDR17 | 0 | 0 | 0 | 0 | 1 | 0 | 1 |
| MAP7D3 | 0 | 0 | 0 | 0 | 1 | 0 | 0 |
| TYW5 | 0 | 0 | 0 | 0 | 1 | 0 | 1 |
| AQP6 | 0 | 0 | 0 | 0 | 1 | 0 | 0 |
| GLUD1P7 | 0 | 0 | 0 | 0 | 1 | 0 | 0 |
| TMEM130 | 0 | 0 | 0 | 0 | 1 | 0 | 0 |
| LARS2-AS1 | 0 | 0 | 0 | 0 | 1 | 0 | 0 |
| PPARA | 0 | 0 | 0 | 0 | 1 | 0 | 0 |
| DZIP3 | 0 | 0 | 0 | 0 | 1 | 0 | 0 |
| MTX3 | 0 | 0 | 0 | 0 | 1 | 0 | 0 |
| LOC728558 | 0 | 0 | 0 | 0 | 1 | 0 | 0 |
| LOC100287792 | 0 | 0 | 0 | 0 | 1 | 0 | 0 |
| C7orf55 | 0 | 0 | 0 | 0 | 1 | 0 | 0 |
| UTY | 0 | 0 | 0 | 0 | 1 | 0 | 0 |
| IFITM3 | 0 | 0 | 0 | 0 | 1 | 0 | 0 |
| PCDHB9 | 0 | 0 | 0 | 0 | 1 | 0 | 1 |
| CD93 | 0 | 0 | 0 | 0 | 1 | 0 | 0 |
| NOS1 | 0 | 0 | 0 | 0 | 1 | 0 | 0 |
| PLEK | 0 | 0 | 0 | 0 | 1 | 1 | 0 |
| LOC100128531 | 0 | 0 | 0 | 0 | 1 | 0 | 0 |
| ANKS4B | 0 | 0 | 0 | 0 | 1 | 0 | 0 |
| ARNTL2 | 0 | 0 | 0 | 0 | 1 | 0 | 0 |
| SLC7A14 | 0 | 0 | 0 | 0 | 1 | 0 | 0 |
| AKIP1 | 0 | 0 | 0 | 0 | 1 | 0 | 0 |
| CYP27C1 | 0 | 0 | 0 | 0 | 1 | 0 | 0 |
| FKBP8 | 0 | 0 | 0 | 0 | 1 | 1 | 1 |
| RAD23B | 0 | 0 | 0 | 0 | 1 | 0 | 0 |
| ORC6 | 0 | 0 | 0 | 0 | 1 | 1 | 0 |
| FBXO45 | 0 | 0 | 0 | 0 | 1 | 0 | 0 |
| FAM200B | 0 | 0 | 0 | 0 | 1 | 0 | 0 |
| NUP43 | 0 | 0 | 0 | 0 | 1 | 0 | 0 |
| SMAD5 | 0 | 0 | 0 | 0 | 1 | 0 | 0 |
| BMP8A | 0 | 0 | 0 | 0 | 1 | 0 | 0 |
| HEXIM1 | 0 | 0 | 0 | 0 | 1 | 0 | 1 |
| DNAJB12 | 0 | 0 | 0 | 0 | 1 | 1 | 0 |
| S1PR2 | 0 | 0 | 0 | 0 | 1 | 0 | 0 |
| ZNF542 | 0 | 0 | 0 | 0 | 1 | 0 | 0 |
| ZNF69 | 0 | 0 | 0 | 0 | 1 | 0 | 0 |
| KIAA1467 | 0 | 0 | 0 | 0 | 1 | 0 | 0 |
| EXOSC6 | 0 | 0 | 0 | 0 | 1 | 0 | 0 |
| DNAJC22 | 0 | 0 | 0 | 0 | 1 | 0 | 0 |
| MTRNR2L1 | 0 | 0 | 0 | 0 | 1 | 0 | 0 |
| VSTM4 | 0 | 0 | 0 | 0 | 1 | 0 | 0 |
| ADAM17 | 0 | 0 | 0 | 0 | 1 | 0 | 0 |
| SBF2-AS1 | 0 | 0 | 0 | 0 | 1 | 0 | 0 |
| DUSP19 | 0 | 0 | 0 | 0 | 1 | 0 | 1 |
| PCDH11Y | 0 | 0 | 0 | 0 | 1 | 0 | 0 |
| NUDT19 | 0 | 0 | 0 | 0 | 1 | 0 | 0 |
| DTD2 | 0 | 0 | 0 | 0 | 1 | 0 | 0 |
| BHMT2 | 0 | 0 | 0 | 0 | 1 | 0 | 1 |
| TMEM241 | 0 | 0 | 0 | 0 | 1 | 1 | 0 |
| CALCOCO2 | 0 | 0 | 0 | 0 | 1 | 0 | 0 |
| AMPD2 | 0 | 0 | 0 | 0 | 1 | 0 | 0 |
| FTH1P3 | 0 | 0 | 0 | 0 | 1 | 0 | 0 |
| EIF4H | 0 | 0 | 0 | 0 | 1 | 0 | 0 |
| GNPNAT1 | 0 | 0 | 0 | 0 | 1 | 0 | 1 |
| RPS6KA6 | 0 | 0 | 0 | 0 | 1 | 0 | 0 |
| FOXRED2 | 0 | 0 | 0 | 0 | 1 | 0 | 0 |
| PCDHA9 | 0 | 0 | 0 | 0 | 1 | 0 | 0 |
| ICA1L | 0 | 0 | 0 | 0 | 1 | 0 | 0 |
| S100A8 | 0 | 0 | 0 | 0 | 1 | 0 | 0 |
| DNAJB6 | 0 | 0 | 0 | 0 | 1 | 1 | 0 |
| DSCR6 | 0 | 0 | 0 | 0 | 1 | 0 | 0 |
| IL10RB | 0 | 0 | 0 | 0 | 1 | 0 | 0 |
| THBD | 0 | 0 | 0 | 0 | 1 | 0 | 1 |
| SLC25A15 | 0 | 0 | 0 | 0 | 1 | 0 | 1 |
| IGFBPL1 | 0 | 0 | 0 | 0 | 1 | 0 | 0 |
| LRIF1 | 0 | 0 | 0 | 0 | 1 | 0 | 1 |
| KATNBL1 | 0 | 0 | 0 | 0 | 1 | 0 | 0 |
| ZNF566 | 0 | 0 | 0 | 0 | 1 | 0 | 0 |
| ATP8B1 | 0 | 0 | 0 | 0 | 1 | 1 | 0 |
| C1orf86 | 0 | 0 | 0 | 0 | 1 | 0 | 0 |
| LRRFIP1 | 0 | 0 | 0 | 0 | 1 | 0 | 0 |
| LOC284023 | 0 | 0 | 0 | 0 | 1 | 0 | 0 |
| CCDC113 | 0 | 0 | 0 | 0 | 1 | 0 | 0 |
| PDIA3 | 0 | 0 | 0 | 0 | 1 | 0 | 0 |
| SKAP2 | 0 | 0 | 0 | 0 | 1 | 0 | 0 |
| BCL2L15 | 0 | 0 | 0 | 0 | 1 | 0 | 0 |
| CTSS | 0 | 0 | 0 | 0 | 1 | 0 | 0 |
| FLNA | 0 | 0 | 0 | 0 | 1 | 0 | 1 |
| IGBP1 | 0 | 0 | 0 | 0 | 1 | 0 | 0 |
| LINC00663 | 0 | 0 | 0 | 0 | 1 | 0 | 0 |
| DBT | 0 | 0 | 0 | 0 | 1 | 0 | 0 |
| AKIRIN1 | 0 | 0 | 0 | 0 | 1 | 0 | 0 |
| IL1B | 0 | 0 | 0 | 0 | 1 | 1 | 0 |
| PLEKHO2 | 0 | 0 | 0 | 0 | 1 | 0 | 0 |
| VAMP3 | 0 | 0 | 0 | 0 | 1 | 0 | 0 |
| RAD1 | 0 | 0 | 0 | 0 | 1 | 0 | 0 |
| TNFAIP6 | 0 | 0 | 0 | 0 | 1 | 1 | 1 |
| TNIP1 | 0 | 0 | 0 | 0 | 1 | 0 | 0 |
| COX7B | 0 | 0 | 0 | 0 | 1 | 0 | 1 |
| TMF1 | 0 | 0 | 0 | 0 | 1 | 0 | 0 |
| HIST2H2AC | 0 | 0 | 0 | 0 | 1 | 0 | 0 |
| HNRNPL | 0 | 0 | 0 | 0 | 1 | 0 | 0 |
| CHP2 | 0 | 0 | 0 | 0 | 1 | 0 | 0 |
| PSME3 | 0 | 0 | 0 | 0 | 1 | 1 | 0 |
| PUM2 | 0 | 0 | 0 | 0 | 1 | 0 | 0 |
| LOC400891 | 0 | 0 | 0 | 0 | 1 | 0 | 0 |
| MRI1 | 0 | 0 | 0 | 0 | 1 | 0 | 0 |
| GP2 | 0 | 0 | 0 | 0 | 1 | 0 | 0 |
| GPLD1 | 0 | 0 | 0 | 0 | 1 | 0 | 1 |
| MTRNR2L6 | 0 | 0 | 0 | 0 | 1 | 0 | 0 |
| GRM6 | 0 | 0 | 0 | 0 | 1 | 0 | 0 |
| CD177 | 0 | 0 | 0 | 0 | 1 | 0 | 1 |
| AKAP5 | 0 | 0 | 0 | 0 | 1 | 0 | 0 |
| SYNGR2 | 0 | 0 | 0 | 0 | 1 | 0 | 0 |
| BAG6 | 0 | 0 | 0 | 0 | 1 | 1 | 0 |
| SLC35E2 | 0 | 0 | 0 | 0 | 1 | 0 | 0 |
| ELL | 0 | 0 | 0 | 0 | 1 | 0 | 0 |
| TNFAIP8L3 | 0 | 0 | 0 | 0 | 1 | 0 | 0 |
| NUP98 | 0 | 0 | 0 | 0 | 1 | 0 | 0 |
| DKFZP434L187 | 0 | 0 | 0 | 0 | 1 | 0 | 0 |
| TRIM59 | 0 | 0 | 0 | 0 | 1 | 0 | 1 |
| ABHD2 | 0 | 0 | 0 | 0 | 1 | 1 | 1 |
| OR51E2 | 0 | 0 | 0 | 0 | 1 | 0 | 0 |
| C3orf33 | 0 | 0 | 0 | 0 | 1 | 0 | 0 |
| ATP6V0C | 0 | 0 | 0 | 0 | 1 | 1 | 0 |
| ATP6AP2 | 0 | 0 | 0 | 0 | 1 | 0 | 0 |
| SF3B2 | 0 | 0 | 0 | 0 | 1 | 0 | 0 |
| TMEM192 | 0 | 0 | 0 | 0 | 1 | 0 | 0 |
| PTGS2 | 0 | 0 | 0 | 0 | 1 | 0 | 0 |
| TUBA3FP | 0 | 0 | 0 | 0 | 1 | 0 | 0 |
| INADL | 0 | 0 | 0 | 0 | 1 | 0 | 0 |
| DBNL | 0 | 0 | 0 | 0 | 1 | 1 | 0 |
| IL10 | 0 | 0 | 0 | 0 | 1 | 0 | 0 |
| ITIH5 | 0 | 0 | 0 | 0 | 1 | 0 | 1 |
| FTH1 | 0 | 0 | 0 | 0 | 1 | 0 | 0 |
| TPM4 | 0 | 0 | 0 | 0 | 1 | 0 | 1 |
| PPP1R15B | 0 | 0 | 0 | 0 | 1 | 0 | 0 |
| BRAT1 | 0 | 0 | 0 | 0 | 1 | 1 | 0 |
| LINC00598 | 0 | 0 | 0 | 0 | 1 | 0 | 0 |
| PARK2 | 0 | 0 | 0 | 0 | 1 | 0 | 0 |
| RNF13 | 0 | 0 | 0 | 0 | 1 | 0 | 0 |
| ZNF260 | 0 | 0 | 0 | 0 | 1 | 0 | 0 |
| TPTE2P1 | 0 | 0 | 0 | 0 | 1 | 0 | 0 |
| C14orf2 | 0 | 0 | 0 | 0 | 1 | 0 | 0 |
| LOC284551 | 0 | 0 | 0 | 0 | 1 | 0 | 0 |
| DUSP6 | 0 | 0 | 0 | 0 | 1 | 0 | 0 |
| ZNF681 | 0 | 0 | 0 | 0 | 1 | 0 | 0 |
| LPIN3 | 0 | 0 | 0 | 0 | 1 | 0 | 0 |
| IL1RAP | 0 | 0 | 0 | 0 | 1 | 1 | 1 |
| C9orf85 | 0 | 0 | 0 | 0 | 1 | 0 | 0 |
| TMEM181 | 0 | 0 | 0 | 0 | 1 | 0 | 0 |
| TMEM19 | 0 | 0 | 0 | 0 | 1 | 0 | 0 |
| STX4 | 0 | 0 | 0 | 0 | 1 | 0 | 0 |
| LOC151475 | 0 | 0 | 0 | 0 | 1 | 0 | 0 |
| HCAR2 | 0 | 0 | 0 | 0 | 1 | 0 | 1 |
| TTC39C | 0 | 0 | 0 | 0 | 1 | 0 | 1 |
| KDM6A | 0 | 0 | 0 | 0 | 1 | 0 | 0 |
| CD82 | 0 | 0 | 0 | 0 | 1 | 0 | 0 |
| CEBPD | 0 | 0 | 0 | 0 | 1 | 0 | 1 |
| KIF3A | 0 | 0 | 0 | 0 | 1 | 0 | 1 |
| LINC00665 | 0 | 0 | 0 | 0 | 1 | 0 | 0 |
| ARHGAP15 | 0 | 0 | 0 | 0 | 1 | 0 | 0 |
| EIF4A1 | 0 | 0 | 0 | 0 | 1 | 0 | 0 |
| ATP1B2 | 0 | 0 | 0 | 0 | 1 | 0 | 0 |
| XRCC6 | 0 | 0 | 0 | 0 | 1 | 0 | 0 |
| MAPK14 | 0 | 0 | 0 | 0 | 1 | 1 | 1 |
| DCUN1D2 | 0 | 0 | 0 | 0 | 1 | 0 | 0 |
| LOC100130954 | 0 | 0 | 0 | 0 | 1 | 0 | 0 |
| RNF40 | 0 | 0 | 0 | 0 | 1 | 0 | 0 |
| NFKB2 | 0 | 0 | 0 | 0 | 1 | 0 | 0 |
| FAM157B | 0 | 0 | 0 | 0 | 1 | 0 | 0 |
| HCAR3 | 0 | 0 | 0 | 0 | 1 | 0 | 0 |
| CST7 | 0 | 0 | 0 | 0 | 1 | 0 | 0 |
| AXL | 0 | 0 | 0 | 0 | 1 | 0 | 1 |
| CARD16 | 0 | 0 | 0 | 0 | 1 | 0 | 0 |
| PTAFR | 0 | 0 | 0 | 0 | 1 | 1 | 0 |
| METTL2B | 0 | 0 | 0 | 0 | 1 | 0 | 0 |
| NFE2L2 | 0 | 0 | 0 | 0 | 1 | 0 | 0 |
| FGL2 | 0 | 0 | 0 | 0 | 1 | 0 | 1 |
| SNW1 | 0 | 0 | 0 | 0 | 1 | 0 | 0 |
| SWSAP1 | 0 | 0 | 0 | 0 | 1 | 0 | 0 |
| ENTPD3-AS1 | 0 | 0 | 0 | 0 | 1 | 0 | 0 |
| CNEP1R1 | 0 | 0 | 0 | 0 | 1 | 0 | 0 |
| GLUL | 0 | 0 | 0 | 0 | 1 | 1 | 0 |
| DNASE1 | 0 | 0 | 0 | 0 | 1 | 0 | 0 |
| SURF4 | 0 | 0 | 0 | 0 | 1 | 0 | 1 |
| C19orf35 | 0 | 0 | 0 | 0 | 1 | 0 | 0 |
| LOH12CR2 | 0 | 0 | 0 | 0 | 1 | 0 | 0 |
| P4HB | 0 | 0 | 0 | 0 | 1 | 0 | 0 |
| ROMO1 | 0 | 0 | 0 | 0 | 1 | 0 | 1 |
| ADAM10 | 0 | 0 | 0 | 0 | 1 | 0 | 0 |
| PTMA | 0 | 0 | 0 | 0 | 1 | 0 | 1 |
| GTPBP3 | 0 | 0 | 0 | 0 | 1 | 0 | 0 |
| CNNM3 | 0 | 0 | 0 | 0 | 1 | 0 | 0 |
| DPYSL5 | 0 | 0 | 0 | 0 | 1 | 0 | 0 |
| CHPF2 | 0 | 0 | 0 | 0 | 1 | 0 | 0 |
| HSPA8 | 0 | 0 | 0 | 0 | 1 | 0 | 0 |
| LOC727896 | 0 | 0 | 0 | 0 | 1 | 0 | 0 |
| RAB11FIP4 | 0 | 0 | 0 | 0 | 1 | 0 | 1 |
| SULT2A1 | 0 | 0 | 0 | 0 | 1 | 0 | 0 |
| ATP2B1 | 0 | 0 | 0 | 0 | 1 | 0 | 0 |
| DPY19L1P1 | 0 | 0 | 0 | 0 | 1 | 0 | 0 |
| CIB1 | 0 | 0 | 0 | 0 | 1 | 0 | 0 |
| SCAMP2 | 0 | 0 | 0 | 0 | 1 | 0 | 0 |
| HDLBP | 0 | 0 | 0 | 0 | 1 | 0 | 0 |
| SOD2 | 0 | 0 | 0 | 0 | 1 | 0 | 1 |
| TIMP1 | 0 | 0 | 0 | 0 | 1 | 1 | 1 |
| EIF3A | 0 | 0 | 0 | 0 | 1 | 0 | 0 |
| SLC22A18 | 0 | 0 | 0 | 0 | 1 | 1 | 1 |
| LPCAT1 | 0 | 0 | 0 | 0 | 1 | 0 | 1 |
| GAK | 0 | 0 | 0 | 0 | 1 | 0 | 0 |
| LOC100505876 | 0 | 0 | 0 | 0 | 1 | 0 | 0 |
| PPIG | 0 | 0 | 0 | 0 | 1 | 1 | 0 |
| IL1RN | 0 | 0 | 0 | 0 | 1 | 0 | 0 |
| IL8 | 0 | 0 | 0 | 0 | 1 | 0 | 0 |
| BCDIN3D-AS1 | 0 | 0 | 0 | 0 | 1 | 0 | 0 |
| ATP6V0D2 | 0 | 0 | 0 | 0 | 1 | 0 | 0 |
| BTF3 | 0 | 0 | 0 | 0 | 1 | 0 | 0 |
| PPCDC | 0 | 0 | 0 | 0 | 1 | 0 | 0 |
| ZC3H12A | 0 | 0 | 0 | 0 | 1 | 1 | 0 |
| TNFRSF14 | 0 | 0 | 0 | 0 | 1 | 0 | 0 |
| CMTM6 | 0 | 0 | 0 | 0 | 1 | 0 | 0 |
| ARSG | 0 | 0 | 0 | 0 | 1 | 1 | 0 |
| NPC2 | 0 | 0 | 0 | 0 | 1 | 0 | 1 |
| DNAJB14 | 0 | 0 | 0 | 0 | 1 | 0 | 0 |
| IRAK1 | 0 | 0 | 0 | 0 | 1 | 0 | 0 |
| RAB2B | 0 | 0 | 0 | 0 | 1 | 1 | 0 |
| CCDC90A | 0 | 0 | 0 | 0 | 1 | 0 | 0 |
| SNRNP200 | 0 | 0 | 0 | 0 | 1 | 0 | 0 |
| TMCC3 | 0 | 0 | 0 | 0 | 1 | 0 | 0 |
| PAR-SN | 0 | 0 | 0 | 0 | 1 | 0 | 0 |
| HSP90B1 | 0 | 0 | 0 | 0 | 1 | 0 | 0 |
| GPCPD1 | 0 | 0 | 0 | 0 | 1 | 0 | 0 |
| EPS15L1 | 0 | 0 | 0 | 0 | 1 | 0 | 0 |
| HIST1H2BC | 0 | 0 | 0 | 0 | 1 | 0 | 0 |
| CHD1 | 0 | 0 | 0 | 0 | 1 | 0 | 0 |
| GBP5 | 0 | 0 | 0 | 0 | 1 | 1 | 0 |
| RRP15 | 0 | 0 | 0 | 0 | 1 | 0 | 0 |
| INSIG1 | 0 | 0 | 0 | 0 | 1 | 0 | 1 |
| KDM4A-AS1 | 0 | 0 | 0 | 0 | 1 | 0 | 0 |
| THAP9-AS1 | 0 | 0 | 0 | 0 | 1 | 0 | 0 |
| TUBA1A | 0 | 0 | 0 | 0 | 1 | 0 | 0 |
| SPRYD7 | 0 | 0 | 0 | 0 | 1 | 0 | 1 |
| ESRG | 0 | 0 | 0 | 0 | 1 | 0 | 0 |
| PGM2L1 | 0 | 0 | 0 | 0 | 1 | 0 | 0 |
| NUCB1 | 0 | 0 | 0 | 0 | 1 | 1 | 0 |
| CEBPB | 0 | 0 | 0 | 0 | 1 | 0 | 0 |
| CCDC30 | 0 | 0 | 0 | 0 | 1 | 0 | 0 |
| FAM41C | 0 | 0 | 0 | 0 | 1 | 0 | 0 |
| DNAJC27-AS1 | 0 | 0 | 0 | 0 | 1 | 0 | 0 |
| ARAF | 0 | 0 | 0 | 0 | 1 | 0 | 0 |
| PLAUR | 0 | 0 | 0 | 0 | 1 | 1 | 0 |
| SMARCA5 | 0 | 0 | 0 | 0 | 1 | 0 | 1 |
| ZNF526 | 0 | 0 | 0 | 0 | 1 | 1 | 0 |
| TMBIM1 | 0 | 0 | 0 | 0 | 1 | 0 | 0 |
| CANT1 | 0 | 0 | 0 | 0 | 1 | 0 | 0 |
| CNIH4 | 0 | 0 | 0 | 0 | 1 | 0 | 0 |
| PDE4B | 0 | 0 | 0 | 0 | 1 | 0 | 0 |
| SUPT20H | 0 | 0 | 0 | 0 | 1 | 0 | 0 |
| BCAS2 | 0 | 0 | 0 | 0 | 1 | 0 | 0 |
| AZIN1 | 0 | 0 | 0 | 0 | 1 | 0 | 0 |
| COPB1 | 0 | 0 | 0 | 0 | 1 | 0 | 0 |
| SERINC3 | 0 | 0 | 0 | 0 | 1 | 0 | 0 |
| SLC3A2 | 0 | 0 | 0 | 0 | 1 | 0 | 0 |
| MX2 | 0 | 0 | 0 | 0 | 1 | 0 | 0 |
| EIF5A2 | 0 | 0 | 0 | 0 | 1 | 0 | 0 |
| SQSTM1 | 0 | 0 | 0 | 0 | 1 | 0 | 0 |
| HIST2H2BF | 0 | 0 | 0 | 0 | 1 | 0 | 0 |
| LOC338758 | 0 | 0 | 0 | 0 | 1 | 0 | 0 |
| EIF4A3 | 0 | 0 | 0 | 0 | 1 | 0 | 0 |
| TPT1 | 0 | 0 | 0 | 0 | 1 | 0 | 0 |
| STRN3 | 0 | 0 | 0 | 0 | 1 | 0 | 0 |
| TM9SF1 | 0 | 0 | 0 | 0 | 1 | 0 | 1 |
| USP16 | 0 | 0 | 0 | 0 | 1 | 1 | 0 |
| AP2M1 | 0 | 0 | 0 | 0 | 1 | 1 | 0 |
| HMGN2 | 0 | 0 | 0 | 0 | 1 | 0 | 1 |
| CSRNP1 | 0 | 0 | 0 | 0 | 1 | 1 | 1 |
| NLN | 0 | 0 | 0 | 0 | 1 | 0 | 1 |
| CASP10 | 0 | 0 | 0 | 0 | 1 | 0 | 0 |
| ALDOA | 0 | 0 | 0 | 0 | 1 | 0 | 0 |
| SSFA2 | 0 | 0 | 0 | 0 | 1 | 0 | 0 |
| CDK5RAP3 | 0 | 0 | 0 | 0 | 1 | 0 | 0 |
| SLC4A1 | 0 | 0 | 0 | 0 | 1 | 1 | 0 |
| TREM1 | 0 | 0 | 0 | 0 | 1 | 0 | 1 |
| HPSE | 0 | 0 | 0 | 0 | 1 | 1 | 0 |
| CD44 | 0 | 0 | 0 | 0 | 1 | 0 | 0 |
| RPL10 | 0 | 0 | 0 | 0 | 1 | 0 | 0 |
| LOC606724 | 0 | 0 | 0 | 0 | 1 | 0 | 0 |
| VPS26A | 0 | 0 | 0 | 0 | 1 | 0 | 0 |
| CHMP2B | 0 | 0 | 0 | 0 | 1 | 0 | 0 |
| MED25 | 0 | 0 | 0 | 0 | 1 | 1 | 0 |
| PPIF | 0 | 0 | 0 | 0 | 1 | 0 | 1 |
| FCHO2 | 0 | 0 | 0 | 0 | 1 | 0 | 0 |
| APLP2 | 0 | 0 | 0 | 0 | 1 | 0 | 1 |
| ICAM1 | 0 | 0 | 0 | 0 | 1 | 0 | 0 |
| PGK1 | 0 | 0 | 0 | 0 | 1 | 0 | 1 |
| ACOT13 | 0 | 0 | 0 | 0 | 1 | 0 | 0 |
| RPL3 | 0 | 0 | 0 | 0 | 1 | 0 | 0 |
| SYTL3 | 0 | 0 | 0 | 0 | 1 | 0 | 0 |
| HSPA5 | 0 | 0 | 0 | 0 | 1 | 0 | 0 |
| ZNF780B | 0 | 0 | 0 | 0 | 1 | 0 | 0 |
| ATP7A | 0 | 0 | 0 | 0 | 1 | 0 | 0 |
| RHBDD2 | 0 | 0 | 0 | 0 | 1 | 0 | 0 |
| SIPA1L1 | 0 | 0 | 0 | 0 | 1 | 0 | 0 |
| PAXBP1-AS1 | 0 | 0 | 0 | 0 | 1 | 0 | 0 |
| IRS2 | 0 | 0 | 0 | 0 | 1 | 0 | 0 |
| CSTA | 0 | 0 | 0 | 0 | 1 | 0 | 0 |
| LOC100505783 | 0 | 0 | 0 | 0 | 1 | 0 | 0 |
| VPS37B | 0 | 0 | 0 | 0 | 1 | 0 | 1 |
| MATR3 | 0 | 0 | 0 | 0 | 1 | 0 | 0 |
| KDM5B | 0 | 0 | 0 | 0 | 1 | 0 | 0 |
| ADPGK | 0 | 0 | 0 | 0 | 1 | 0 | 0 |
| SNX13 | 0 | 0 | 0 | 0 | 1 | 0 | 0 |
| PSMC4 | 0 | 0 | 0 | 0 | 1 | 0 | 0 |
| H6PD | 0 | 0 | 0 | 0 | 1 | 0 | 1 |
| ATP6V0A2 | 0 | 0 | 0 | 0 | 1 | 0 | 0 |
| ZNF548 | 0 | 0 | 0 | 0 | 1 | 0 | 0 |
| SNN | 0 | 0 | 0 | 0 | 1 | 1 | 0 |
| HSH2D | 0 | 0 | 0 | 0 | 1 | 0 | 0 |
| ZFP36L2 | 0 | 0 | 0 | 0 | 1 | 0 | 0 |
| FXR1 | 0 | 0 | 0 | 0 | 1 | 0 | 0 |
| RNASET2 | 0 | 0 | 0 | 0 | 1 | 0 | 0 |
| KLF10 | 0 | 0 | 0 | 0 | 1 | 0 | 0 |
| NUB1 | 0 | 0 | 0 | 0 | 1 | 0 | 0 |
| GTF2H2B | 0 | 0 | 0 | 0 | 1 | 1 | 0 |
| ZSWIM1 | 0 | 0 | 0 | 0 | 1 | 0 | 0 |
| NAIP | 0 | 0 | 0 | 0 | 1 | 0 | 0 |
| MRVI1 | 0 | 0 | 0 | 0 | 1 | 0 | 0 |
| ARIH2 | 0 | 0 | 0 | 0 | 1 | 0 | 0 |
| LDHA | 0 | 0 | 0 | 0 | 1 | 0 | 1 |
| PMAIP1 | 0 | 0 | 0 | 0 | 1 | 0 | 0 |
| TSPO | 0 | 0 | 0 | 0 | 1 | 0 | 0 |
| FFAR2 | 0 | 0 | 0 | 0 | 1 | 0 | 0 |
| POLDIP3 | 0 | 0 | 0 | 0 | 1 | 0 | 0 |
| MASTL | 0 | 0 | 0 | 0 | 1 | 0 | 0 |
| NCOA4 | 0 | 0 | 0 | 0 | 1 | 0 | 0 |
| TRIM66 | 0 | 0 | 0 | 0 | 1 | 0 | 0 |
| CD68 | 0 | 0 | 0 | 0 | 1 | 0 | 0 |
| MICU1 | 0 | 0 | 0 | 0 | 1 | 1 | 0 |
| RELA | 0 | 0 | 0 | 0 | 1 | 1 | 0 |
| SERPINB9 | 0 | 0 | 0 | 0 | 1 | 0 | 0 |
| ARF4 | 0 | 0 | 0 | 0 | 1 | 0 | 0 |
| TMED9 | 0 | 0 | 0 | 0 | 1 | 0 | 0 |
| TMEM45B | 0 | 0 | 0 | 0 | 1 | 1 | 1 |
| AFTPH | 0 | 0 | 0 | 0 | 1 | 0 | 0 |
| MIF | 0 | 0 | 0 | 0 | 1 | 0 | 0 |
| SUPT6H | 0 | 0 | 0 | 0 | 1 | 0 | 0 |
| LPIN2 | 0 | 0 | 0 | 0 | 1 | 1 | 0 |
| MRPL30 | 0 | 0 | 0 | 0 | 1 | 0 | 0 |
| DNAJB11 | 0 | 0 | 0 | 0 | 1 | 0 | 0 |
| PBXIP1 | 0 | 0 | 0 | 0 | 1 | 0 | 1 |
| ZNF527 | 0 | 0 | 0 | 0 | 1 | 0 | 0 |
| RPL14 | 0 | 0 | 0 | 0 | 1 | 0 | 0 |
| TNFRSF1B | 0 | 0 | 0 | 0 | 1 | 0 | 0 |
| RPL11 | 0 | 0 | 0 | 0 | 1 | 0 | 0 |
| IVNS1ABP | 0 | 0 | 0 | 0 | 1 | 0 | 0 |
| KDELR2 | 0 | 0 | 0 | 0 | 1 | 0 | 1 |
| KLHL2 | 0 | 0 | 0 | 0 | 1 | 0 | 0 |
| ZNF234 | 0 | 0 | 0 | 0 | 1 | 0 | 0 |
| RLIM | 0 | 0 | 0 | 0 | 1 | 0 | 0 |
| CXCL16 | 0 | 0 | 0 | 0 | 1 | 1 | 0 |
| OCLN | 0 | 0 | 0 | 0 | 1 | 0 | 0 |
| METTL6 | 0 | 0 | 0 | 0 | 1 | 0 | 0 |
| SCN3B | 0 | 0 | 0 | 0 | 1 | 0 | 0 |
| POLR2J3 | 0 | 0 | 0 | 0 | 1 | 0 | 0 |
| SH3BP5 | 0 | 0 | 0 | 0 | 1 | 0 | 1 |
| ARL17A | 0 | 0 | 0 | 0 | 1 | 0 | 0 |
| ARFIP1 | 0 | 0 | 0 | 0 | 1 | 0 | 0 |
| C17orf103 | 0 | 0 | 0 | 0 | 1 | 0 | 0 |
| KLHL24 | 0 | 0 | 0 | 0 | 1 | 0 | 0 |
| ODF3B | 0 | 0 | 0 | 0 | 1 | 0 | 0 |
| CPSF7 | 0 | 0 | 0 | 0 | 1 | 0 | 0 |
| RPL6 | 0 | 0 | 0 | 0 | 1 | 0 | 0 |
| GAPDH | 0 | 0 | 0 | 0 | 1 | 0 | 1 |
| MORF4L2 | 0 | 0 | 0 | 0 | 1 | 0 | 0 |
| GAA | 0 | 0 | 0 | 0 | 1 | 0 | 0 |
| NFKBID | 0 | 0 | 0 | 0 | 1 | 0 | 0 |
| PTBP1 | 0 | 0 | 0 | 0 | 1 | 0 | 0 |
| PRKACA | 0 | 0 | 0 | 0 | 1 | 0 | 0 |
| TIMP2 | 0 | 0 | 0 | 0 | 1 | 0 | 0 |
| USMG5 | 0 | 0 | 0 | 0 | 1 | 0 | 0 |
| GNB4 | 0 | 0 | 0 | 0 | 1 | 0 | 0 |
| HIAT1 | 0 | 0 | 0 | 0 | 1 | 0 | 0 |
| PER2 | 0 | 0 | 0 | 0 | 1 | 0 | 0 |
| LOC100129250 | 0 | 0 | 0 | 0 | 1 | 0 | 0 |
| PHOSPHO1 | 0 | 0 | 0 | 0 | 1 | 1 | 0 |
| KDM3A | 0 | 0 | 0 | 0 | 1 | 0 | 0 |
| RPS16 | 0 | 0 | 0 | 0 | 1 | 0 | 1 |
| MAB21L3 | 0 | 0 | 0 | 0 | 1 | 0 | 0 |
| MGC2752 | 0 | 0 | 0 | 0 | 1 | 0 | 0 |
| PRKDC | 0 | 0 | 0 | 0 | 1 | 0 | 0 |
| SYAP1 | 0 | 0 | 0 | 0 | 1 | 0 | 0 |
| RLF | 0 | 0 | 0 | 0 | 1 | 0 | 0 |
| AP2A1 | 0 | 0 | 0 | 0 | 1 | 0 | 0 |
| CANX | 0 | 0 | 0 | 0 | 1 | 0 | 0 |
| SNRPB | 0 | 0 | 0 | 0 | 1 | 0 | 0 |
| SLC26A8 | 0 | 0 | 0 | 0 | 1 | 0 | 1 |
| ETF1 | 0 | 0 | 0 | 0 | 1 | 0 | 0 |
| LILRA3 | 0 | 0 | 0 | 0 | 1 | 1 | 0 |
| HIVEP1 | 0 | 0 | 0 | 0 | 1 | 0 | 0 |
| RANBP2 | 0 | 0 | 0 | 0 | 1 | 0 | 0 |
| NDUFB3 | 0 | 0 | 0 | 0 | 1 | 1 | 0 |
| IFIT2 | 0 | 0 | 0 | 0 | 1 | 0 | 0 |
| PNPLA8 | 0 | 0 | 0 | 0 | 1 | 0 | 0 |
| CDK5RAP2 | 0 | 0 | 0 | 0 | 1 | 0 | 0 |
| ENO1 | 0 | 0 | 0 | 0 | 1 | 0 | 0 |
| RAB22A | 0 | 0 | 0 | 0 | 1 | 0 | 0 |
| NBN | 0 | 0 | 0 | 0 | 1 | 0 | 0 |
| CPPED1 | 0 | 0 | 0 | 0 | 1 | 0 | 0 |
| EOGT | 0 | 0 | 0 | 0 | 1 | 0 | 0 |
| DHRSX | 0 | 0 | 0 | 0 | 1 | 1 | 0 |
| UBE2L6 | 0 | 0 | 0 | 0 | 1 | 1 | 0 |
| CCDC71L | 0 | 0 | 0 | 0 | 1 | 0 | 0 |
| NAPA | 0 | 0 | 0 | 0 | 1 | 1 | 0 |
| HMGN1 | 0 | 0 | 0 | 0 | 1 | 0 | 0 |
| DMXL2 | 0 | 0 | 0 | 0 | 1 | 0 | 1 |
| MCOLN1 | 0 | 0 | 0 | 0 | 1 | 1 | 0 |
| RPS29 | 0 | 0 | 0 | 0 | 1 | 0 | 0 |
| PRPF4 | 0 | 0 | 0 | 0 | 1 | 1 | 0 |
| IFIT3 | 0 | 0 | 0 | 0 | 1 | 1 | 0 |
| ARHGDIA | 0 | 0 | 0 | 0 | 1 | 0 | 0 |
| ZNF440 | 0 | 0 | 0 | 0 | 1 | 0 | 1 |
| ZNF554 | 0 | 0 | 0 | 0 | 1 | 0 | 0 |
| MAPKAPK3 | 0 | 0 | 0 | 0 | 1 | 0 | 0 |
| EMD | 0 | 0 | 0 | 0 | 1 | 0 | 0 |
| SERTAD1 | 0 | 0 | 0 | 0 | 1 | 0 | 1 |
| KCTD20 | 0 | 0 | 0 | 0 | 1 | 0 | 0 |
| MSMO1 | 0 | 0 | 0 | 0 | 1 | 0 | 1 |
| PECAM1 | 0 | 0 | 0 | 0 | 1 | 0 | 0 |
| MGAT4A | 0 | 0 | 0 | 0 | 1 | 0 | 0 |
| SLMO2 | 0 | 0 | 0 | 0 | 1 | 0 | 0 |
| LOC284454 | 0 | 0 | 0 | 0 | 1 | 0 | 0 |
| AP2B1 | 0 | 0 | 0 | 0 | 1 | 1 | 0 |
| FAM210B | 0 | 0 | 0 | 0 | 1 | 1 | 0 |
| ATXN2L | 0 | 0 | 0 | 0 | 1 | 0 | 0 |
| GGA1 | 0 | 0 | 0 | 0 | 1 | 0 | 0 |
| ZNF37A | 0 | 0 | 0 | 0 | 1 | 0 | 0 |
| EMB | 0 | 0 | 0 | 0 | 1 | 0 | 0 |
| LOC100506710 | 0 | 0 | 0 | 0 | 1 | 0 | 0 |
| DNTTIP2 | 0 | 0 | 0 | 0 | 1 | 1 | 0 |
| GZF1 | 0 | 0 | 0 | 0 | 1 | 0 | 0 |
| OXSR1 | 0 | 0 | 0 | 0 | 1 | 0 | 0 |
| TPI1 | 0 | 0 | 0 | 0 | 1 | 0 | 0 |
| NKIRAS2 | 0 | 0 | 0 | 0 | 1 | 1 | 0 |
| DDX21 | 0 | 0 | 0 | 0 | 1 | 0 | 0 |
| RBM41 | 0 | 0 | 0 | 0 | 1 | 0 | 0 |
| GTPBP1 | 0 | 0 | 0 | 0 | 1 | 0 | 0 |
| CPD | 0 | 0 | 0 | 0 | 1 | 0 | 1 |
| IRAK3 | 0 | 0 | 0 | 0 | 1 | 0 | 1 |
| GPX4 | 0 | 0 | 0 | 0 | 1 | 0 | 0 |
| SIGLEC10 | 0 | 0 | 0 | 0 | 1 | 1 | 1 |
| ZEB1 | 0 | 0 | 0 | 0 | 1 | 0 | 0 |
| HEXB | 0 | 0 | 0 | 0 | 1 | 0 | 0 |
| USP14 | 0 | 0 | 0 | 0 | 1 | 0 | 0 |
| GPR65 | 0 | 0 | 0 | 0 | 1 | 0 | 0 |
| ZNF516 | 0 | 0 | 0 | 0 | 1 | 0 | 0 |
| EML4 | 0 | 0 | 0 | 0 | 1 | 0 | 0 |
| GBP6 | 0 | 0 | 0 | 0 | 1 | 0 | 0 |
| HIF1A | 0 | 0 | 0 | 0 | 1 | 0 | 1 |
| AGPAT6 | 0 | 0 | 0 | 0 | 1 | 0 | 0 |
| FAM174A | 0 | 0 | 0 | 0 | 1 | 0 | 1 |
| RNF169 | 0 | 0 | 0 | 0 | 1 | 0 | 0 |
| SESN2 | 0 | 0 | 0 | 0 | 1 | 0 | 1 |
| S100A4 | 0 | 0 | 0 | 0 | 1 | 0 | 1 |
| SIN3A | 0 | 0 | 0 | 0 | 1 | 0 | 0 |
| RABGEF1 | 0 | 0 | 0 | 0 | 1 | 0 | 0 |
| RGS2 | 0 | 0 | 0 | 0 | 1 | 0 | 1 |
| QSOX1 | 0 | 0 | 0 | 0 | 1 | 0 | 1 |
| ATG2A | 0 | 0 | 0 | 0 | 1 | 0 | 0 |
| HSP90AB1 | 0 | 0 | 0 | 0 | 1 | 0 | 0 |
| DNAJC14 | 0 | 0 | 0 | 0 | 1 | 0 | 0 |
| RHEB | 0 | 0 | 0 | 0 | 1 | 0 | 0 |
| KCNE3 | 0 | 0 | 0 | 0 | 1 | 1 | 1 |
| RPS8 | 0 | 0 | 0 | 0 | 1 | 0 | 0 |
| APH1B | 0 | 0 | 0 | 0 | 1 | 0 | 0 |
| STOM | 0 | 0 | 0 | 0 | 1 | 0 | 0 |
| MOAP1 | 0 | 0 | 0 | 0 | 1 | 0 | 0 |
| CD97 | 0 | 0 | 0 | 0 | 1 | 0 | 0 |
| MOCS3 | 0 | 0 | 0 | 0 | 1 | 0 | 0 |
| SEC16A | 0 | 0 | 0 | 0 | 1 | 0 | 0 |
| LINC00476 | 0 | 0 | 0 | 0 | 1 | 0 | 0 |
| PLK3 | 0 | 0 | 0 | 0 | 1 | 0 | 0 |
| SLC20A1 | 0 | 0 | 0 | 0 | 1 | 0 | 1 |
| SULF2 | 0 | 0 | 0 | 0 | 1 | 1 | 0 |
| NXPE3 | 0 | 0 | 0 | 0 | 1 | 0 | 0 |
| CD83 | 0 | 0 | 0 | 0 | 1 | 0 | 0 |
| TRAF3IP3 | 0 | 0 | 0 | 0 | 1 | 1 | 0 |
| CLEC2D | 0 | 0 | 0 | 0 | 1 | 0 | 0 |
| RASA4 | 0 | 0 | 0 | 0 | 1 | 0 | 0 |
| POLB | 0 | 0 | 0 | 0 | 1 | 0 | 0 |
| MAP3K8 | 0 | 0 | 0 | 0 | 1 | 0 | 0 |
| PURB | 0 | 0 | 0 | 0 | 1 | 0 | 0 |
| GADD45B | 0 | 0 | 0 | 0 | 1 | 0 | 1 |
| PLEKHB2 | 0 | 0 | 0 | 0 | 1 | 0 | 0 |
| MLH3 | 0 | 0 | 0 | 0 | 0 | 1 | 0 |
| TMEM176A | 0 | 0 | 0 | 0 | 0 | 1 | 1 |
| SCGB3A1 | 0 | 0 | 0 | 0 | 0 | 1 | 0 |
| TMEM176B | 0 | 0 | 0 | 0 | 0 | 1 | 1 |
| HSPC102 | 0 | 0 | 0 | 0 | 0 | 1 | 0 |
| CHP1 | 0 | 0 | 0 | 0 | 0 | 1 | 1 |
| NATD1 | 0 | 0 | 0 | 0 | 0 | 1 | 0 |
| APOM | 0 | 0 | 0 | 0 | 0 | 1 | 1 |
| LOC101928343 | 0 | 0 | 0 | 0 | 0 | 1 | 0 |
| PRR11 | 0 | 0 | 0 | 0 | 0 | 1 | 0 |
| CUTALP | 0 | 0 | 0 | 0 | 0 | 1 | 0 |
| HLA-DQA1 | 0 | 0 | 0 | 0 | 0 | 1 | 1 |
| ACSM2A | 0 | 0 | 0 | 0 | 0 | 1 | 0 |
| SMAGP | 0 | 0 | 0 | 0 | 0 | 1 | 0 |
| HLA-DQB1 | 0 | 0 | 0 | 0 | 0 | 1 | 0 |
| CDK5 | 0 | 0 | 0 | 0 | 0 | 1 | 0 |
| EEF1AKMT3 | 0 | 0 | 0 | 0 | 0 | 1 | 1 |
| CD99P1 | 0 | 0 | 0 | 0 | 0 | 1 | 0 |
| HNRNPH3 | 0 | 0 | 0 | 0 | 0 | 1 | 0 |
| INSIG1-DT | 0 | 0 | 0 | 0 | 0 | 1 | 0 |
| SIGLEC9 | 0 | 0 | 0 | 0 | 0 | 1 | 0 |
| SLC24A3 | 0 | 0 | 0 | 0 | 0 | 1 | 0 |
| SERPINB9P1 | 0 | 0 | 0 | 0 | 0 | 1 | 0 |
| CCEPR | 0 | 0 | 0 | 0 | 0 | 1 | 0 |
| LOC100996756 | 0 | 0 | 0 | 0 | 0 | 1 | 0 |
| SLC22A3 | 0 | 0 | 0 | 0 | 0 | 1 | 0 |
| LINC02802 | 0 | 0 | 0 | 0 | 0 | 1 | 0 |
| SIRT6 | 0 | 0 | 0 | 0 | 0 | 1 | 0 |
| DEPDC5 | 0 | 0 | 0 | 0 | 0 | 1 | 0 |
| TRIB3 | 0 | 0 | 0 | 0 | 0 | 1 | 0 |
| SLC10A3 | 0 | 0 | 0 | 0 | 0 | 1 | 0 |
| SENCR | 0 | 0 | 0 | 0 | 0 | 1 | 0 |
| ELAPOR1 | 0 | 0 | 0 | 0 | 0 | 1 | 1 |
| RITA1 | 0 | 0 | 0 | 0 | 0 | 1 | 0 |
| MAX | 0 | 0 | 0 | 0 | 0 | 1 | 0 |
| CASTOR3 | 0 | 0 | 0 | 0 | 0 | 1 | 0 |
| C4BPA | 0 | 0 | 0 | 0 | 0 | 1 | 1 |
| KHNYN | 0 | 0 | 0 | 0 | 0 | 1 | 0 |
| ANKRD36B | 0 | 0 | 0 | 0 | 0 | 1 | 0 |
| PKIG | 0 | 0 | 0 | 0 | 0 | 1 | 1 |
| LOC105371215 | 0 | 0 | 0 | 0 | 0 | 1 | 0 |
| CMAS | 0 | 0 | 0 | 0 | 0 | 1 | 0 |
| ZNF595 | 0 | 0 | 0 | 0 | 0 | 1 | 0 |
| ARF3 | 0 | 0 | 0 | 0 | 0 | 1 | 0 |
| NCR3 | 0 | 0 | 0 | 0 | 0 | 1 | 0 |
| AGPAT1 | 0 | 0 | 0 | 0 | 0 | 1 | 0 |
| DHFR2 | 0 | 0 | 0 | 0 | 0 | 1 | 1 |
| LRRC42 | 0 | 0 | 0 | 0 | 0 | 1 | 0 |
| ACTR1B | 0 | 0 | 0 | 0 | 0 | 1 | 0 |
| BEND2 | 0 | 0 | 0 | 0 | 0 | 1 | 0 |
| ELOA-AS1 | 0 | 0 | 0 | 0 | 0 | 1 | 0 |
| MIX23 | 0 | 0 | 0 | 0 | 0 | 1 | 1 |
| ELP5 | 0 | 0 | 0 | 0 | 0 | 1 | 0 |
| DUBR | 0 | 0 | 0 | 0 | 0 | 1 | 0 |
| UGT2B28 | 0 | 0 | 0 | 0 | 0 | 1 | 0 |
| LOC100289333 | 0 | 0 | 0 | 0 | 0 | 1 | 0 |
| ENTPD1-AS1 | 0 | 0 | 0 | 0 | 0 | 1 | 0 |
| RAMP2-AS1 | 0 | 0 | 0 | 0 | 0 | 1 | 1 |
| DYNLL2 | 0 | 0 | 0 | 0 | 0 | 1 | 0 |
| MRPL49 | 0 | 0 | 0 | 0 | 0 | 1 | 0 |
| ADAM33 | 0 | 0 | 0 | 0 | 0 | 1 | 0 |
| RAB37 | 0 | 0 | 0 | 0 | 0 | 1 | 0 |
| LOC105375547 | 0 | 0 | 0 | 0 | 0 | 1 | 0 |
| ACADVL | 0 | 0 | 0 | 0 | 0 | 1 | 1 |
| LOC105369228 | 0 | 0 | 0 | 0 | 0 | 1 | 0 |
| BRD3OS | 0 | 0 | 0 | 0 | 0 | 1 | 0 |
| PRPF38B | 0 | 0 | 0 | 0 | 0 | 1 | 0 |
| RIC8A | 0 | 0 | 0 | 0 | 0 | 1 | 0 |
| ECRG4 | 0 | 0 | 0 | 0 | 0 | 1 | 0 |
| DDX11L2 | 0 | 0 | 0 | 0 | 0 | 1 | 0 |
| H2BC21 | 0 | 0 | 0 | 0 | 0 | 1 | 0 |
| LOC114224 | 0 | 0 | 0 | 0 | 0 | 1 | 0 |
| TMEM185A | 0 | 0 | 0 | 0 | 0 | 1 | 0 |
| ARHGEF1 | 0 | 0 | 0 | 0 | 0 | 1 | 0 |
| TASL | 0 | 0 | 0 | 0 | 0 | 1 | 0 |
| POM121L8P | 0 | 0 | 0 | 0 | 0 | 1 | 0 |
| GNG5 | 0 | 0 | 0 | 0 | 0 | 1 | 0 |
| ZNF747 | 0 | 0 | 0 | 0 | 0 | 1 | 0 |
| ACTR1A | 0 | 0 | 0 | 0 | 0 | 1 | 0 |
| TBC1D25 | 0 | 0 | 0 | 0 | 0 | 1 | 0 |
| ATG13 | 0 | 0 | 0 | 0 | 0 | 1 | 0 |
| NIPSNAP1 | 0 | 0 | 0 | 0 | 0 | 1 | 1 |
| FKBP1A | 0 | 0 | 0 | 0 | 0 | 1 | 0 |
| BRF2 | 0 | 0 | 0 | 0 | 0 | 1 | 1 |
| TP53I3 | 0 | 0 | 0 | 0 | 0 | 1 | 0 |
| PVALB | 0 | 0 | 0 | 0 | 0 | 1 | 0 |
| SH3BGRL3 | 0 | 0 | 0 | 0 | 0 | 1 | 0 |
| CREB3L4 | 0 | 0 | 0 | 0 | 0 | 1 | 0 |
| NOTCH2NLA | 0 | 0 | 0 | 0 | 0 | 1 | 0 |
| BRAF | 0 | 0 | 0 | 0 | 0 | 1 | 0 |
| DHRS1 | 0 | 0 | 0 | 0 | 0 | 1 | 0 |
| ERMAP | 0 | 0 | 0 | 0 | 0 | 1 | 0 |
| LOC105378577 | 0 | 0 | 0 | 0 | 0 | 1 | 0 |
| ELOVL7 | 0 | 0 | 0 | 0 | 0 | 1 | 0 |
| HIF1AN | 0 | 0 | 0 | 0 | 0 | 1 | 0 |
| H2BC8 | 0 | 0 | 0 | 0 | 0 | 1 | 0 |
| TNFAIP8L1 | 0 | 0 | 0 | 0 | 0 | 1 | 0 |
| SMIM24 | 0 | 0 | 0 | 0 | 0 | 1 | 0 |
| KIR2DL2 | 0 | 0 | 0 | 0 | 0 | 1 | 0 |
| ECSIT | 0 | 0 | 0 | 0 | 0 | 1 | 0 |
| HOMER1 | 0 | 0 | 0 | 0 | 0 | 1 | 1 |
| CAVIN3 | 0 | 0 | 0 | 0 | 0 | 1 | 1 |
| TTC7B | 0 | 0 | 0 | 0 | 0 | 1 | 0 |
| CNN2 | 0 | 0 | 0 | 0 | 0 | 1 | 0 |
| SWI5 | 0 | 0 | 0 | 0 | 0 | 1 | 0 |
| COL9A3 | 0 | 0 | 0 | 0 | 0 | 1 | 0 |
| ACD | 0 | 0 | 0 | 0 | 0 | 1 | 0 |
| TMEM109 | 0 | 0 | 0 | 0 | 0 | 1 | 0 |
| C8orf82 | 0 | 0 | 0 | 0 | 0 | 1 | 0 |
| MRPL51 | 0 | 0 | 0 | 0 | 0 | 1 | 0 |
| MPV17 | 0 | 0 | 0 | 0 | 0 | 1 | 0 |
| VPS72 | 0 | 0 | 0 | 0 | 0 | 1 | 0 |
| LOC105379362 | 0 | 0 | 0 | 0 | 0 | 1 | 0 |
| MIF4GD | 0 | 0 | 0 | 0 | 0 | 1 | 0 |
| TMEM212 | 0 | 0 | 0 | 0 | 0 | 1 | 0 |
| SNRPN | 0 | 0 | 0 | 0 | 0 | 1 | 0 |
| PPP1R18 | 0 | 0 | 0 | 0 | 0 | 1 | 0 |
| DUSP14 | 0 | 0 | 0 | 0 | 0 | 1 | 0 |
| ZNF721 | 0 | 0 | 0 | 0 | 0 | 1 | 0 |
| SRXN1 | 0 | 0 | 0 | 0 | 0 | 1 | 0 |
| DET1 | 0 | 0 | 0 | 0 | 0 | 1 | 0 |
| CTDSP1 | 0 | 0 | 0 | 0 | 0 | 1 | 0 |
| TKTL1 | 0 | 0 | 0 | 0 | 0 | 1 | 0 |
| SNAI3 | 0 | 0 | 0 | 0 | 0 | 1 | 0 |
| HADHA | 0 | 0 | 0 | 0 | 0 | 1 | 0 |
| RSRP1 | 0 | 0 | 0 | 0 | 0 | 1 | 0 |
| LOC100507642 | 0 | 0 | 0 | 0 | 0 | 1 | 0 |
| MACF1 | 0 | 0 | 0 | 0 | 0 | 1 | 0 |
| GUSBP14 | 0 | 0 | 0 | 0 | 0 | 1 | 0 |
| PDLIM1 | 0 | 0 | 0 | 0 | 0 | 1 | 0 |
| TNFSF12 | 0 | 0 | 0 | 0 | 0 | 1 | 0 |
| SSBP3-AS1 | 0 | 0 | 0 | 0 | 0 | 1 | 0 |
| PRSS33 | 0 | 0 | 0 | 0 | 0 | 1 | 0 |
| TAL1 | 0 | 0 | 0 | 0 | 0 | 1 | 0 |
| GCHFR | 0 | 0 | 0 | 0 | 0 | 1 | 0 |
| GPS2 | 0 | 0 | 0 | 0 | 0 | 1 | 0 |
| POLR2J | 0 | 0 | 0 | 0 | 0 | 1 | 0 |
| CPTP | 0 | 0 | 0 | 0 | 0 | 1 | 0 |
| CAPNS1 | 0 | 0 | 0 | 0 | 0 | 1 | 0 |
| DEFB108B | 0 | 0 | 0 | 0 | 0 | 1 | 0 |
| ZSWIM3 | 0 | 0 | 0 | 0 | 0 | 1 | 0 |
| GOLGA8IP | 0 | 0 | 0 | 0 | 0 | 1 | 0 |
| CHID1 | 0 | 0 | 0 | 0 | 0 | 1 | 0 |
| LIPA | 0 | 0 | 0 | 0 | 0 | 1 | 0 |
| SPARC | 0 | 0 | 0 | 0 | 0 | 1 | 0 |
| RANGRF | 0 | 0 | 0 | 0 | 0 | 1 | 1 |
| LOC101927770 | 0 | 0 | 0 | 0 | 0 | 1 | 0 |
| LINC01809 | 0 | 0 | 0 | 0 | 0 | 1 | 0 |
| KPNA1 | 0 | 0 | 0 | 0 | 0 | 1 | 0 |
| SLC29A3 | 0 | 0 | 0 | 0 | 0 | 1 | 0 |
| POLDIP2 | 0 | 0 | 0 | 0 | 0 | 1 | 1 |
| CHI3L2 | 0 | 0 | 0 | 0 | 0 | 1 | 0 |
| WIF1 | 0 | 0 | 0 | 0 | 0 | 1 | 0 |
| LOC729732 | 0 | 0 | 0 | 0 | 0 | 1 | 0 |
| MMP24OS | 0 | 0 | 0 | 0 | 0 | 1 | 0 |
| DESI1 | 0 | 0 | 0 | 0 | 0 | 1 | 1 |
| GPX7 | 0 | 0 | 0 | 0 | 0 | 1 | 0 |
| ASB6 | 0 | 0 | 0 | 0 | 0 | 1 | 0 |
| FBXW12 | 0 | 0 | 0 | 0 | 0 | 1 | 0 |
| SHISA4 | 0 | 0 | 0 | 0 | 0 | 1 | 0 |
| COX2 | 0 | 0 | 0 | 0 | 0 | 1 | 0 |
| H2AC11 | 0 | 0 | 0 | 0 | 0 | 1 | 0 |
| PI4KB | 0 | 0 | 0 | 0 | 0 | 1 | 0 |
| RETREG2 | 0 | 0 | 0 | 0 | 0 | 1 | 0 |
| SLC9B1 | 0 | 0 | 0 | 0 | 0 | 1 | 0 |
| OR52K3P | 0 | 0 | 0 | 0 | 0 | 1 | 0 |
| ND4 | 0 | 0 | 0 | 0 | 0 | 1 | 0 |
| MAP1S | 0 | 0 | 0 | 0 | 0 | 1 | 0 |
| RILP | 0 | 0 | 0 | 0 | 0 | 1 | 0 |
| YY1AP1 | 0 | 0 | 0 | 0 | 0 | 1 | 0 |
| PCLAF | 0 | 0 | 0 | 0 | 0 | 1 | 0 |
| ALDOC | 0 | 0 | 0 | 0 | 0 | 1 | 1 |
| MEN1 | 0 | 0 | 0 | 0 | 0 | 1 | 0 |
| CIDEB | 0 | 0 | 0 | 0 | 0 | 1 | 0 |
| RNF185 | 0 | 0 | 0 | 0 | 0 | 1 | 0 |
| CMTM5 | 0 | 0 | 0 | 0 | 0 | 1 | 0 |
| LINC00328 | 0 | 0 | 0 | 0 | 0 | 1 | 0 |
| TSHZ3 | 0 | 0 | 0 | 0 | 0 | 1 | 0 |
| LOC105376486 | 0 | 0 | 0 | 0 | 0 | 1 | 0 |
| TRIM26 | 0 | 0 | 0 | 0 | 0 | 1 | 0 |
| COPS6 | 0 | 0 | 0 | 0 | 0 | 1 | 0 |
| ZNF394 | 0 | 0 | 0 | 0 | 0 | 1 | 0 |
| RBM22 | 0 | 0 | 0 | 0 | 0 | 1 | 0 |
| CNPPD1 | 0 | 0 | 0 | 0 | 0 | 1 | 0 |
| RAB43 | 0 | 0 | 0 | 0 | 0 | 1 | 0 |
| ORAI3 | 0 | 0 | 0 | 0 | 0 | 1 | 1 |
| MPST | 0 | 0 | 0 | 0 | 0 | 1 | 0 |
| ELF4 | 0 | 0 | 0 | 0 | 0 | 1 | 0 |
| CCDC159 | 0 | 0 | 0 | 0 | 0 | 1 | 0 |
| SIRPB1 | 0 | 0 | 0 | 0 | 0 | 1 | 0 |
| EFNA4 | 0 | 0 | 0 | 0 | 0 | 1 | 0 |
| XNDC1N | 0 | 0 | 0 | 0 | 0 | 1 | 0 |
| CDC42EP2 | 0 | 0 | 0 | 0 | 0 | 1 | 0 |
| MXRA7 | 0 | 0 | 0 | 0 | 0 | 1 | 0 |
| RCOR3 | 0 | 0 | 0 | 0 | 0 | 1 | 0 |
| GMDS-DT | 0 | 0 | 0 | 0 | 0 | 1 | 0 |
| LYSMD2 | 0 | 0 | 0 | 0 | 0 | 1 | 0 |
| UTP4 | 0 | 0 | 0 | 0 | 0 | 1 | 0 |
| CYB561D2 | 0 | 0 | 0 | 0 | 0 | 1 | 0 |
| RTL8C | 0 | 0 | 0 | 0 | 0 | 1 | 0 |
| LOC100505874 | 0 | 0 | 0 | 0 | 0 | 1 | 0 |
| H3C10 | 0 | 0 | 0 | 0 | 0 | 1 | 0 |
| KLHDC3 | 0 | 0 | 0 | 0 | 0 | 1 | 0 |
| TBC1D20 | 0 | 0 | 0 | 0 | 0 | 1 | 0 |
| HEXIM2 | 0 | 0 | 0 | 0 | 0 | 1 | 0 |
| LOC105377458 | 0 | 0 | 0 | 0 | 0 | 1 | 0 |
| ESF1 | 0 | 0 | 0 | 0 | 0 | 1 | 0 |
| LOC100190986 | 0 | 0 | 0 | 0 | 0 | 1 | 0 |
| PFAS | 0 | 0 | 0 | 0 | 0 | 1 | 0 |
| SHISA5 | 0 | 0 | 0 | 0 | 0 | 1 | 0 |
| RNASEH2A | 0 | 0 | 0 | 0 | 0 | 1 | 0 |
| H2AC8 | 0 | 0 | 0 | 0 | 0 | 1 | 0 |
| LINC02754 | 0 | 0 | 0 | 0 | 0 | 1 | 0 |
| ND2 | 0 | 0 | 0 | 0 | 0 | 1 | 0 |
| VPS26B | 0 | 0 | 0 | 0 | 0 | 1 | 0 |
| CLK4 | 0 | 0 | 0 | 0 | 0 | 1 | 0 |
| ANKRD20A12P | 0 | 0 | 0 | 0 | 0 | 1 | 0 |
| TOX2 | 0 | 0 | 0 | 0 | 0 | 1 | 0 |
| TTI2 | 0 | 0 | 0 | 0 | 0 | 1 | 0 |
| LRRC75A | 0 | 0 | 0 | 0 | 0 | 1 | 0 |
| USP30 | 0 | 0 | 0 | 0 | 0 | 1 | 1 |
| ZNF831 | 0 | 0 | 0 | 0 | 0 | 1 | 0 |
| MIR3682 | 0 | 0 | 0 | 0 | 0 | 1 | 0 |
| RMDN3 | 0 | 0 | 0 | 0 | 0 | 1 | 1 |
| TAGLN2 | 0 | 0 | 0 | 0 | 0 | 1 | 1 |
| PRDX6 | 0 | 0 | 0 | 0 | 0 | 1 | 1 |
| ACTR3BP2 | 0 | 0 | 0 | 0 | 0 | 1 | 0 |
| NDUFB1 | 0 | 0 | 0 | 0 | 0 | 1 | 0 |
| SNIP1 | 0 | 0 | 0 | 0 | 0 | 1 | 0 |
| PPP5D1P | 0 | 0 | 0 | 0 | 0 | 1 | 0 |
| DENND4A | 0 | 0 | 0 | 0 | 0 | 1 | 0 |
| TRIM35 | 0 | 0 | 0 | 0 | 0 | 1 | 0 |
| C9orf64 | 0 | 0 | 0 | 0 | 0 | 1 | 0 |
| RASEF | 0 | 0 | 0 | 0 | 0 | 1 | 1 |
| CCDC18-AS1 | 0 | 0 | 0 | 0 | 0 | 1 | 0 |
| TUBA3D | 0 | 0 | 0 | 0 | 0 | 1 | 0 |
| ACKR4 | 0 | 0 | 0 | 0 | 0 | 1 | 0 |
| LGALS3BP | 0 | 0 | 0 | 0 | 0 | 1 | 0 |
| TSPAN33 | 0 | 0 | 0 | 0 | 0 | 1 | 1 |
| TIPARP | 0 | 0 | 0 | 0 | 0 | 1 | 0 |
| DDX41 | 0 | 0 | 0 | 0 | 0 | 1 | 0 |
| HLA-DRB4 | 0 | 0 | 0 | 0 | 0 | 1 | 0 |
| S1PR1-DT | 0 | 0 | 0 | 0 | 0 | 1 | 0 |
| NOP58 | 0 | 0 | 0 | 0 | 0 | 1 | 0 |
| ALG8 | 0 | 0 | 0 | 0 | 0 | 1 | 0 |
| LOC389834 | 0 | 0 | 0 | 0 | 0 | 1 | 0 |
| STARD10 | 0 | 0 | 0 | 0 | 0 | 1 | 0 |
| TIMM8A | 0 | 0 | 0 | 0 | 0 | 1 | 0 |
| TUBB4B | 0 | 0 | 0 | 0 | 0 | 1 | 1 |
| TRA2A | 0 | 0 | 0 | 0 | 0 | 1 | 0 |
| CDK4 | 0 | 0 | 0 | 0 | 0 | 1 | 0 |
| SLC25A11 | 0 | 0 | 0 | 0 | 0 | 1 | 0 |
| F8 | 0 | 0 | 0 | 0 | 0 | 1 | 0 |
| TUSC2 | 0 | 0 | 0 | 0 | 0 | 1 | 0 |
| PELI3 | 0 | 0 | 0 | 0 | 0 | 1 | 1 |
| MCUR1 | 0 | 0 | 0 | 0 | 0 | 1 | 0 |
| SUV39H1 | 0 | 0 | 0 | 0 | 0 | 1 | 0 |
| MIPEPP3 | 0 | 0 | 0 | 0 | 0 | 1 | 0 |
| LOC101927018 | 0 | 0 | 0 | 0 | 0 | 1 | 0 |
| PIP5K1C | 0 | 0 | 0 | 0 | 0 | 1 | 0 |
| COX3 | 0 | 0 | 0 | 0 | 0 | 1 | 0 |
| MAP3K1 | 0 | 0 | 0 | 0 | 0 | 1 | 0 |
| RUNDC3A | 0 | 0 | 0 | 0 | 0 | 1 | 0 |
| IP6K2 | 0 | 0 | 0 | 0 | 0 | 1 | 0 |
| SIGLEC5 | 0 | 0 | 0 | 0 | 0 | 1 | 0 |
| TMEM164 | 0 | 0 | 0 | 0 | 0 | 1 | 0 |
| CARM1 | 0 | 0 | 0 | 0 | 0 | 1 | 0 |
| FRG1BP | 0 | 0 | 0 | 0 | 0 | 1 | 0 |
| FHL3 | 0 | 0 | 0 | 0 | 0 | 1 | 0 |
| MALAT1 | 0 | 0 | 0 | 0 | 0 | 1 | 0 |
| MED8 | 0 | 0 | 0 | 0 | 0 | 1 | 0 |
| PRAF2 | 0 | 0 | 0 | 0 | 0 | 1 | 0 |
| GBGT1 | 0 | 0 | 0 | 0 | 0 | 1 | 0 |
| HEY1 | 0 | 0 | 0 | 0 | 0 | 1 | 1 |
| FKSG49 | 0 | 0 | 0 | 0 | 0 | 1 | 0 |
| GFI1B | 0 | 0 | 0 | 0 | 0 | 1 | 0 |
| MLLT11 | 0 | 0 | 0 | 0 | 0 | 1 | 1 |
| ZSCAN22 | 0 | 0 | 0 | 0 | 0 | 1 | 0 |
| CIZ1 | 0 | 0 | 0 | 0 | 0 | 1 | 0 |
| XKR8 | 0 | 0 | 0 | 0 | 0 | 1 | 0 |
| CHAMP1 | 0 | 0 | 0 | 0 | 0 | 1 | 0 |
| TMEM141 | 0 | 0 | 0 | 0 | 0 | 1 | 0 |
| MGLL | 0 | 0 | 0 | 0 | 0 | 1 | 0 |
| GNAZ | 0 | 0 | 0 | 0 | 0 | 1 | 1 |
| GOLGA6L2 | 0 | 0 | 0 | 0 | 0 | 1 | 0 |
| NEK2-DT | 0 | 0 | 0 | 0 | 0 | 1 | 0 |
| UCP2 | 0 | 0 | 0 | 0 | 0 | 1 | 0 |
| DHRS7B | 0 | 0 | 0 | 0 | 0 | 1 | 0 |
| TMEM9B | 0 | 0 | 0 | 0 | 0 | 1 | 0 |
| WASHC5 | 0 | 0 | 0 | 0 | 0 | 1 | 0 |
| ARFIP2 | 0 | 0 | 0 | 0 | 0 | 1 | 0 |
| LOC100505915 | 0 | 0 | 0 | 0 | 0 | 1 | 0 |
| SSNA1 | 0 | 0 | 0 | 0 | 0 | 1 | 0 |
| OS9 | 0 | 0 | 0 | 0 | 0 | 1 | 0 |
| LOC654780 | 0 | 0 | 0 | 0 | 0 | 1 | 0 |
| ZBED5-AS1 | 0 | 0 | 0 | 0 | 0 | 1 | 0 |
| VAT1 | 0 | 0 | 0 | 0 | 0 | 1 | 1 |
| KLF3 | 0 | 0 | 0 | 0 | 0 | 1 | 0 |
| PTDSS1 | 0 | 0 | 0 | 0 | 0 | 1 | 0 |
| KAT5 | 0 | 0 | 0 | 0 | 0 | 1 | 0 |
| XRCC2 | 0 | 0 | 0 | 0 | 0 | 1 | 0 |
| PNISR | 0 | 0 | 0 | 0 | 0 | 1 | 0 |
| BAP1 | 0 | 0 | 0 | 0 | 0 | 1 | 0 |
| MAP2K2 | 0 | 0 | 0 | 0 | 0 | 1 | 0 |
| PRMT5 | 0 | 0 | 0 | 0 | 0 | 1 | 0 |
| TMEM41B | 0 | 0 | 0 | 0 | 0 | 1 | 0 |
| TCTA | 0 | 0 | 0 | 0 | 0 | 1 | 0 |
| ADRM1 | 0 | 0 | 0 | 0 | 0 | 1 | 0 |
| MICALL1 | 0 | 0 | 0 | 0 | 0 | 1 | 0 |
| DUSP23 | 0 | 0 | 0 | 0 | 0 | 1 | 0 |
| MZT2B | 0 | 0 | 0 | 0 | 0 | 1 | 0 |
| SLC48A1 | 0 | 0 | 0 | 0 | 0 | 1 | 0 |
| ALOX12 | 0 | 0 | 0 | 0 | 0 | 1 | 0 |
| LRRC69 | 0 | 0 | 0 | 0 | 0 | 1 | 0 |
| TMEM179B | 0 | 0 | 0 | 0 | 0 | 1 | 0 |
| SYNGR3 | 0 | 0 | 0 | 0 | 0 | 1 | 0 |
| ZNF213 | 0 | 0 | 0 | 0 | 0 | 1 | 1 |
| BIK | 0 | 0 | 0 | 0 | 0 | 1 | 0 |
| MEIS1 | 0 | 0 | 0 | 0 | 0 | 1 | 0 |
| IL32 | 0 | 0 | 0 | 0 | 0 | 1 | 0 |
| TMEM185B | 0 | 0 | 0 | 0 | 0 | 1 | 0 |
| PTGS1 | 0 | 0 | 0 | 0 | 0 | 1 | 0 |
| SH3GLB2 | 0 | 0 | 0 | 0 | 0 | 1 | 1 |
| DEDD2 | 0 | 0 | 0 | 0 | 0 | 1 | 0 |
| KCTD21 | 0 | 0 | 0 | 0 | 0 | 1 | 0 |
| FANCG | 0 | 0 | 0 | 0 | 0 | 1 | 0 |
| TCP11L2 | 0 | 0 | 0 | 0 | 0 | 1 | 0 |
| ZFYVE27 | 0 | 0 | 0 | 0 | 0 | 1 | 0 |
| HSP90AA1 | 0 | 0 | 0 | 0 | 0 | 1 | 1 |
| INPP5K | 0 | 0 | 0 | 0 | 0 | 1 | 0 |
| ASF1B | 0 | 0 | 0 | 0 | 0 | 1 | 0 |
| TMEM158 | 0 | 0 | 0 | 0 | 0 | 1 | 0 |
| H2BC7 | 0 | 0 | 0 | 0 | 0 | 1 | 0 |
| ABHD14A | 0 | 0 | 0 | 0 | 0 | 1 | 0 |
| CHMP6 | 0 | 0 | 0 | 0 | 0 | 1 | 1 |
| SRGAP2B | 0 | 0 | 0 | 0 | 0 | 1 | 0 |
| LGALSL | 0 | 0 | 0 | 0 | 0 | 1 | 1 |
| ZNF296 | 0 | 0 | 0 | 0 | 0 | 1 | 0 |
| MGC70870 | 0 | 0 | 0 | 0 | 0 | 1 | 0 |
| GET3 | 0 | 0 | 0 | 0 | 0 | 1 | 0 |
| FCGR2B | 0 | 0 | 0 | 0 | 0 | 1 | 0 |
| PPM1M | 0 | 0 | 0 | 0 | 0 | 1 | 1 |
| NEMF | 0 | 0 | 0 | 0 | 0 | 1 | 0 |
| LYL1 | 0 | 0 | 0 | 0 | 0 | 1 | 0 |
| TRAPPC1 | 0 | 0 | 0 | 0 | 0 | 1 | 1 |
| NDUFAF3 | 0 | 0 | 0 | 0 | 0 | 1 | 0 |
| NGDN | 0 | 0 | 0 | 0 | 0 | 1 | 0 |
| CAPN12 | 0 | 0 | 0 | 0 | 0 | 1 | 1 |
| SLC35A4 | 0 | 0 | 0 | 0 | 0 | 1 | 0 |
| BCAS3 | 0 | 0 | 0 | 0 | 0 | 1 | 0 |
| SH3GL3 | 0 | 0 | 0 | 0 | 0 | 1 | 0 |
| SLC38A5 | 0 | 0 | 0 | 0 | 0 | 1 | 0 |
| KCTD2 | 0 | 0 | 0 | 0 | 0 | 1 | 0 |
| PPP1R15A | 0 | 0 | 0 | 0 | 0 | 1 | 1 |
| DCTN1 | 0 | 0 | 0 | 0 | 0 | 1 | 0 |
| RNF41 | 0 | 0 | 0 | 0 | 0 | 1 | 0 |
| TANGO2 | 0 | 0 | 0 | 0 | 0 | 1 | 0 |
| ZNFX1 | 0 | 0 | 0 | 0 | 0 | 1 | 0 |
| ORMDL2 | 0 | 0 | 0 | 0 | 0 | 1 | 0 |
| NUDT2 | 0 | 0 | 0 | 0 | 0 | 1 | 0 |
| NT5M | 0 | 0 | 0 | 0 | 0 | 1 | 0 |
| VKORC1 | 0 | 0 | 0 | 0 | 0 | 1 | 1 |
| LINC01949 | 0 | 0 | 0 | 0 | 0 | 1 | 0 |
| CYP27A1 | 0 | 0 | 0 | 0 | 0 | 1 | 0 |
| LINC00597 | 0 | 0 | 0 | 0 | 0 | 1 | 0 |
| H2AW | 0 | 0 | 0 | 0 | 0 | 1 | 0 |
| POLL | 0 | 0 | 0 | 0 | 0 | 1 | 0 |
| CBX3 | 0 | 0 | 0 | 0 | 0 | 1 | 0 |
| TRIM10 | 0 | 0 | 0 | 0 | 0 | 1 | 0 |
| COG7 | 0 | 0 | 0 | 0 | 0 | 1 | 0 |
| ARHGAP6 | 0 | 0 | 0 | 0 | 0 | 1 | 1 |
| SMIM3 | 0 | 0 | 0 | 0 | 0 | 1 | 0 |
| POLA1 | 0 | 0 | 0 | 0 | 0 | 1 | 0 |
| MAP2K3 | 0 | 0 | 0 | 0 | 0 | 1 | 0 |
| PCGF5 | 0 | 0 | 0 | 0 | 0 | 1 | 0 |
| TNFAIP3 | 0 | 0 | 0 | 0 | 0 | 1 | 0 |
| TADA2B | 0 | 0 | 0 | 0 | 0 | 1 | 0 |
| NRGN | 0 | 0 | 0 | 0 | 0 | 1 | 1 |
| UBAC1 | 0 | 0 | 0 | 0 | 0 | 1 | 0 |
| TSPYL5 | 0 | 0 | 0 | 0 | 0 | 1 | 0 |
| PODNL1 | 0 | 0 | 0 | 0 | 0 | 1 | 0 |
| MCAT | 0 | 0 | 0 | 0 | 0 | 1 | 0 |
| CDKN1A | 0 | 0 | 0 | 0 | 0 | 1 | 0 |
| CYB5R3 | 0 | 0 | 0 | 0 | 0 | 1 | 0 |
| SLC25A39 | 0 | 0 | 0 | 0 | 0 | 1 | 0 |
| WDTC1 | 0 | 0 | 0 | 0 | 0 | 1 | 0 |
| MIR181A2HG | 0 | 0 | 0 | 0 | 0 | 1 | 0 |
| RTL6 | 0 | 0 | 0 | 0 | 0 | 1 | 1 |
| RNF19B | 0 | 0 | 0 | 0 | 0 | 1 | 0 |
| SHARPIN | 0 | 0 | 0 | 0 | 0 | 1 | 0 |
| FBXO7 | 0 | 0 | 0 | 0 | 0 | 1 | 0 |
| HECTD3 | 0 | 0 | 0 | 0 | 0 | 1 | 0 |
| GYPC | 0 | 0 | 0 | 0 | 0 | 1 | 0 |
| BAK1 | 0 | 0 | 0 | 0 | 0 | 1 | 0 |
| HPS1 | 0 | 0 | 0 | 0 | 0 | 1 | 0 |
| IMP4 | 0 | 0 | 0 | 0 | 0 | 1 | 0 |
| PGAP2 | 0 | 0 | 0 | 0 | 0 | 1 | 0 |
| TSPAN2 | 0 | 0 | 0 | 0 | 0 | 1 | 1 |
| POLD4 | 0 | 0 | 0 | 0 | 0 | 1 | 0 |
| TNFSF4 | 0 | 0 | 0 | 0 | 0 | 1 | 0 |
| ENDOD1 | 0 | 0 | 0 | 0 | 0 | 1 | 0 |
| SMIM1 | 0 | 0 | 0 | 0 | 0 | 1 | 0 |
| CRTC2 | 0 | 0 | 0 | 0 | 0 | 1 | 0 |
| DMTN | 0 | 0 | 0 | 0 | 0 | 1 | 0 |
| MARCHF2 | 0 | 0 | 0 | 0 | 0 | 1 | 0 |
| SIGLEC17P | 0 | 0 | 0 | 0 | 0 | 1 | 0 |
| ZFYVE21 | 0 | 0 | 0 | 0 | 0 | 1 | 0 |
| STK11 | 0 | 0 | 0 | 0 | 0 | 1 | 0 |
| IPO11 | 0 | 0 | 0 | 0 | 0 | 1 | 0 |
| IRF2 | 0 | 0 | 0 | 0 | 0 | 1 | 0 |
| TST | 0 | 0 | 0 | 0 | 0 | 1 | 1 |
| GCDH | 0 | 0 | 0 | 0 | 0 | 1 | 1 |
| KRT18 | 0 | 0 | 0 | 0 | 0 | 1 | 0 |
| IP6K1 | 0 | 0 | 0 | 0 | 0 | 1 | 0 |
| ZBED6CL | 0 | 0 | 0 | 0 | 0 | 1 | 0 |
| NELFE | 0 | 0 | 0 | 0 | 0 | 1 | 1 |
| CALCOCO1 | 0 | 0 | 0 | 0 | 0 | 1 | 0 |
| CPA3 | 0 | 0 | 0 | 0 | 0 | 1 | 1 |
| MYG1 | 0 | 0 | 0 | 0 | 0 | 1 | 0 |
| DHPS | 0 | 0 | 0 | 0 | 0 | 1 | 0 |
| TICAM1 | 0 | 0 | 0 | 0 | 0 | 1 | 0 |
| B9D2 | 0 | 0 | 0 | 0 | 0 | 1 | 1 |
| TOMM40L | 0 | 0 | 0 | 0 | 0 | 1 | 0 |
| POTEM | 0 | 0 | 0 | 0 | 0 | 1 | 0 |
| TUBG1 | 0 | 0 | 0 | 0 | 0 | 1 | 0 |
| MYADM | 0 | 0 | 0 | 0 | 0 | 1 | 1 |
| GPR160 | 0 | 0 | 0 | 0 | 0 | 1 | 0 |
| CXCR6 | 0 | 0 | 0 | 0 | 0 | 1 | 0 |
| OST4 | 0 | 0 | 0 | 0 | 0 | 1 | 0 |
| KIAA0040 | 0 | 0 | 0 | 0 | 0 | 1 | 0 |
| TAFA1 | 0 | 0 | 0 | 0 | 0 | 1 | 0 |
| NRM | 0 | 0 | 0 | 0 | 0 | 1 | 0 |
| C2orf88 | 0 | 0 | 0 | 0 | 0 | 1 | 0 |
| PLA2G15 | 0 | 0 | 0 | 0 | 0 | 1 | 1 |
| TPST2 | 0 | 0 | 0 | 0 | 0 | 1 | 0 |
| CCRL2 | 0 | 0 | 0 | 0 | 0 | 1 | 0 |
| TMEM44-AS1 | 0 | 0 | 0 | 0 | 0 | 1 | 0 |
| STIMATE | 0 | 0 | 0 | 0 | 0 | 1 | 0 |
| HEBP1 | 0 | 0 | 0 | 0 | 0 | 1 | 0 |
| TTC14 | 0 | 0 | 0 | 0 | 0 | 1 | 0 |
| CYSTM1 | 0 | 0 | 0 | 0 | 0 | 1 | 0 |
| SLAMF6 | 0 | 0 | 0 | 0 | 0 | 1 | 0 |
| DPF2 | 0 | 0 | 0 | 0 | 0 | 1 | 0 |
| PKIA | 0 | 0 | 0 | 0 | 0 | 1 | 0 |
| SURF2 | 0 | 0 | 0 | 0 | 0 | 1 | 1 |
| TMCO6 | 0 | 0 | 0 | 0 | 0 | 1 | 0 |
| STING1 | 0 | 0 | 0 | 0 | 0 | 1 | 0 |
| CCR3 | 0 | 0 | 0 | 0 | 0 | 1 | 0 |
| TM7SF2 | 0 | 0 | 0 | 0 | 0 | 1 | 0 |
| ARL2BP | 0 | 0 | 0 | 0 | 0 | 1 | 0 |
| LRRTM2 | 0 | 0 | 0 | 0 | 0 | 1 | 0 |
| CHTF8 | 0 | 0 | 0 | 0 | 0 | 1 | 0 |
| CDC34 | 0 | 0 | 0 | 0 | 0 | 1 | 0 |
| PIN4 | 0 | 0 | 0 | 0 | 0 | 1 | 0 |
| LINC00919 | 0 | 0 | 0 | 0 | 0 | 1 | 0 |
| BMX | 0 | 0 | 0 | 0 | 0 | 1 | 0 |
| CSNK2A1 | 0 | 0 | 0 | 0 | 0 | 1 | 0 |
| GBAP1 | 0 | 0 | 0 | 0 | 0 | 1 | 0 |
| LINC00342 | 0 | 0 | 0 | 0 | 0 | 1 | 0 |
| DENND1A | 0 | 0 | 0 | 0 | 0 | 1 | 0 |
| NEU1 | 0 | 0 | 0 | 0 | 0 | 1 | 0 |
| SNHG16 | 0 | 0 | 0 | 0 | 0 | 1 | 0 |
| FAM117A | 0 | 0 | 0 | 0 | 0 | 1 | 0 |
| TMEM140 | 0 | 0 | 0 | 0 | 0 | 1 | 1 |
| LINC02256 | 0 | 0 | 0 | 0 | 0 | 1 | 0 |
| ABCD1 | 0 | 0 | 0 | 0 | 0 | 1 | 0 |
| MICOS10P1 | 0 | 0 | 0 | 0 | 0 | 1 | 0 |
| NINJ1 | 0 | 0 | 0 | 0 | 0 | 1 | 1 |
| SHMT2 | 0 | 0 | 0 | 0 | 0 | 1 | 1 |
| AGAP9 | 0 | 0 | 0 | 0 | 0 | 1 | 1 |
| DUXAP8 | 0 | 0 | 0 | 0 | 0 | 1 | 0 |
| GFUS | 0 | 0 | 0 | 0 | 0 | 1 | 0 |
| PEF1 | 0 | 0 | 0 | 0 | 0 | 1 | 0 |
| TGFB1I1 | 0 | 0 | 0 | 0 | 0 | 1 | 0 |
| TREML2 | 0 | 0 | 0 | 0 | 0 | 1 | 0 |
| GNPDA1 | 0 | 0 | 0 | 0 | 0 | 1 | 0 |
| TSSC4 | 0 | 0 | 0 | 0 | 0 | 1 | 0 |
| C15orf39 | 0 | 0 | 0 | 0 | 0 | 1 | 0 |
| ZFAND3 | 0 | 0 | 0 | 0 | 0 | 1 | 0 |
| RNF10 | 0 | 0 | 0 | 0 | 0 | 1 | 0 |
| SH3RF3 | 0 | 0 | 0 | 0 | 0 | 1 | 0 |
| EPHA1-AS1 | 0 | 0 | 0 | 0 | 0 | 1 | 0 |
| ADIPOR1 | 0 | 0 | 0 | 0 | 0 | 1 | 0 |
| UVRAG | 0 | 0 | 0 | 0 | 0 | 1 | 0 |
| KAT7 | 0 | 0 | 0 | 0 | 0 | 1 | 0 |
| ZC3HC1 | 0 | 0 | 0 | 0 | 0 | 1 | 0 |
| COPZ1 | 0 | 0 | 0 | 0 | 0 | 1 | 0 |
| LINC02076 | 0 | 0 | 0 | 0 | 0 | 1 | 0 |
| DAD1 | 0 | 0 | 0 | 0 | 0 | 1 | 0 |
| PINK1 | 0 | 0 | 0 | 0 | 0 | 1 | 0 |
| BCKDK | 0 | 0 | 0 | 0 | 0 | 1 | 1 |
| FBXW4 | 0 | 0 | 0 | 0 | 0 | 1 | 0 |
| BCR | 0 | 0 | 0 | 0 | 0 | 1 | 0 |
| RBCK1 | 0 | 0 | 0 | 0 | 0 | 1 | 0 |
| EPM2A-DT | 0 | 0 | 0 | 0 | 0 | 1 | 0 |
| MXI1 | 0 | 0 | 0 | 0 | 0 | 1 | 1 |
| AIFM1 | 0 | 0 | 0 | 0 | 0 | 1 | 1 |
| GPKOW | 0 | 0 | 0 | 0 | 0 | 1 | 0 |
| MEIS3P1 | 0 | 0 | 0 | 0 | 0 | 1 | 0 |
| TFEB | 0 | 0 | 0 | 0 | 0 | 1 | 1 |
| SF3A2 | 0 | 0 | 0 | 0 | 0 | 1 | 0 |
| SEM1 | 0 | 0 | 0 | 0 | 0 | 1 | 0 |
| CHST7 | 0 | 0 | 0 | 0 | 0 | 1 | 0 |
| BAG1 | 0 | 0 | 0 | 0 | 0 | 1 | 1 |
| MPND | 0 | 0 | 0 | 0 | 0 | 1 | 1 |
| GPR146 | 0 | 0 | 0 | 0 | 0 | 1 | 0 |
| ABL1 | 0 | 0 | 0 | 0 | 0 | 1 | 0 |
| MLF2 | 0 | 0 | 0 | 0 | 0 | 1 | 0 |
| HSPB1 | 0 | 0 | 0 | 0 | 0 | 1 | 1 |
| STRN4 | 0 | 0 | 0 | 0 | 0 | 1 | 0 |
| PCGF1 | 0 | 0 | 0 | 0 | 0 | 1 | 0 |
| APRT | 0 | 0 | 0 | 0 | 0 | 1 | 0 |
| TCL1A | 0 | 0 | 0 | 0 | 0 | 1 | 0 |
| GDE1 | 0 | 0 | 0 | 0 | 0 | 1 | 0 |
| S100A11 | 0 | 0 | 0 | 0 | 0 | 1 | 0 |
| TPRG1L | 0 | 0 | 0 | 0 | 0 | 1 | 0 |
| AK1 | 0 | 0 | 0 | 0 | 0 | 1 | 0 |
| LOC441666 | 0 | 0 | 0 | 0 | 0 | 1 | 0 |
| BANF1 | 0 | 0 | 0 | 0 | 0 | 1 | 1 |
| UBE2M | 0 | 0 | 0 | 0 | 0 | 1 | 0 |
| CPQ | 0 | 0 | 0 | 0 | 0 | 1 | 0 |
| PARN | 0 | 0 | 0 | 0 | 0 | 1 | 0 |
| GSEC | 0 | 0 | 0 | 0 | 0 | 1 | 0 |
| LTBP2 | 0 | 0 | 0 | 0 | 0 | 1 | 1 |
| GLB1 | 0 | 0 | 0 | 0 | 0 | 1 | 0 |
| KIR2DS2 | 0 | 0 | 0 | 0 | 0 | 1 | 0 |
| SRM | 0 | 0 | 0 | 0 | 0 | 1 | 0 |
| RAB5C | 0 | 0 | 0 | 0 | 0 | 1 | 0 |
| NCAPD2 | 0 | 0 | 0 | 0 | 0 | 1 | 0 |
| TMEM86B | 0 | 0 | 0 | 0 | 0 | 1 | 1 |
| CDK2AP2 | 0 | 0 | 0 | 0 | 0 | 1 | 0 |
| PDIA5 | 0 | 0 | 0 | 0 | 0 | 1 | 0 |
| ST6GALNAC4 | 0 | 0 | 0 | 0 | 0 | 1 | 0 |
| H2BC12 | 0 | 0 | 0 | 0 | 0 | 1 | 0 |
| F13A1 | 0 | 0 | 0 | 0 | 0 | 1 | 0 |
| SGO2 | 0 | 0 | 0 | 0 | 0 | 1 | 0 |
| GIMAP7 | 0 | 0 | 0 | 0 | 0 | 1 | 1 |
| CTDSP2 | 0 | 0 | 0 | 0 | 0 | 1 | 0 |
| UBXN6 | 0 | 0 | 0 | 0 | 0 | 1 | 0 |
| MARCKSL1 | 0 | 0 | 0 | 0 | 0 | 1 | 0 |
| RNF182 | 0 | 0 | 0 | 0 | 0 | 1 | 0 |
| NUDT16L2P | 0 | 0 | 0 | 0 | 0 | 1 | 0 |
| RNF123 | 0 | 0 | 0 | 0 | 0 | 1 | 0 |
| ACRBP | 0 | 0 | 0 | 0 | 0 | 1 | 0 |
| C6orf136 | 0 | 0 | 0 | 0 | 0 | 1 | 1 |
| NFKBIE | 0 | 0 | 0 | 0 | 0 | 1 | 0 |
| CDCA4 | 0 | 0 | 0 | 0 | 0 | 1 | 1 |
| ATP6 | 0 | 0 | 0 | 0 | 0 | 1 | 0 |
| SLC6A8 | 0 | 0 | 0 | 0 | 0 | 1 | 0 |
| SLC25A1 | 0 | 0 | 0 | 0 | 0 | 1 | 0 |
| PPM1G | 0 | 0 | 0 | 0 | 0 | 1 | 0 |
| KTN1 | 0 | 0 | 0 | 0 | 0 | 1 | 0 |
| GRAP2 | 0 | 0 | 0 | 0 | 0 | 1 | 0 |
| ETHE1 | 0 | 0 | 0 | 0 | 0 | 1 | 0 |
| PGRMC1 | 0 | 0 | 0 | 0 | 0 | 1 | 1 |
| PIP4P1 | 0 | 0 | 0 | 0 | 0 | 1 | 0 |
| PIM1 | 0 | 0 | 0 | 0 | 0 | 1 | 0 |
| GPSM3 | 0 | 0 | 0 | 0 | 0 | 1 | 0 |
| CTSD | 0 | 0 | 0 | 0 | 0 | 1 | 0 |
| TLR4 | 0 | 0 | 0 | 0 | 0 | 1 | 0 |
| ARRB2 | 0 | 0 | 0 | 0 | 0 | 1 | 0 |
| GYS1 | 0 | 0 | 0 | 0 | 0 | 1 | 0 |
| ADA2 | 0 | 0 | 0 | 0 | 0 | 1 | 0 |
| AHSA2P | 0 | 0 | 0 | 0 | 0 | 1 | 0 |
| PCK2 | 0 | 0 | 0 | 0 | 0 | 1 | 1 |
| WDR54 | 0 | 0 | 0 | 0 | 0 | 1 | 0 |
| USP39 | 0 | 0 | 0 | 0 | 0 | 1 | 0 |
| KIFBP | 0 | 0 | 0 | 0 | 0 | 1 | 0 |
| ALAS1 | 0 | 0 | 0 | 0 | 0 | 1 | 1 |
| ISG20 | 0 | 0 | 0 | 0 | 0 | 1 | 0 |
| TCN1 | 0 | 0 | 0 | 0 | 0 | 1 | 0 |
| AP1M1 | 0 | 0 | 0 | 0 | 0 | 1 | 0 |
| MYL4 | 0 | 0 | 0 | 0 | 0 | 1 | 0 |
| TUBB6 | 0 | 0 | 0 | 0 | 0 | 1 | 1 |
| NPRL3 | 0 | 0 | 0 | 0 | 0 | 1 | 0 |
| ABCG1 | 0 | 0 | 0 | 0 | 0 | 1 | 1 |
| ASGR1 | 0 | 0 | 0 | 0 | 0 | 1 | 1 |
| RXRA | 0 | 0 | 0 | 0 | 0 | 1 | 0 |
| ADGRE1 | 0 | 0 | 0 | 0 | 0 | 1 | 0 |
| CORO1B | 0 | 0 | 0 | 0 | 0 | 1 | 0 |
| BAZ2B-AS1 | 0 | 0 | 0 | 0 | 0 | 1 | 0 |
| LOC100310756 | 0 | 0 | 0 | 0 | 0 | 1 | 0 |
| DPM1 | 0 | 0 | 0 | 0 | 0 | 1 | 0 |
| PRR5 | 0 | 0 | 0 | 0 | 0 | 1 | 0 |
| ABCF1 | 0 | 0 | 0 | 0 | 0 | 1 | 0 |
| MPP1 | 0 | 0 | 0 | 0 | 0 | 1 | 0 |
| ACKR1 | 0 | 0 | 0 | 0 | 0 | 1 | 0 |
| PLPPR2 | 0 | 0 | 0 | 0 | 0 | 1 | 0 |
| SAC3D1 | 0 | 0 | 0 | 0 | 0 | 1 | 1 |
| USP7-AS1 | 0 | 0 | 0 | 0 | 0 | 1 | 0 |
| CHPT1 | 0 | 0 | 0 | 0 | 0 | 1 | 1 |
| GAS2L1 | 0 | 0 | 0 | 0 | 0 | 1 | 0 |
| RBIS | 0 | 0 | 0 | 0 | 0 | 1 | 0 |
| FABP5 | 0 | 0 | 0 | 0 | 0 | 1 | 1 |
| FAXDC2 | 0 | 0 | 0 | 0 | 0 | 1 | 0 |
| KIR3DL1 | 0 | 0 | 0 | 0 | 0 | 1 | 0 |
| H2BC9 | 0 | 0 | 0 | 0 | 0 | 1 | 0 |
| LOC105379250 | 0 | 0 | 0 | 0 | 0 | 1 | 0 |
| MTX1 | 0 | 0 | 0 | 0 | 0 | 1 | 0 |
| LILRA1 | 0 | 0 | 0 | 0 | 0 | 1 | 1 |
| JAZF1 | 0 | 0 | 0 | 0 | 0 | 1 | 0 |
| RNPEPL1 | 0 | 0 | 0 | 0 | 0 | 1 | 0 |
| R3HDM4 | 0 | 0 | 0 | 0 | 0 | 1 | 0 |
| CBWD2 | 0 | 0 | 0 | 0 | 0 | 1 | 0 |
| LINC01857 | 0 | 0 | 0 | 0 | 0 | 1 | 0 |
| ATP5ME | 0 | 0 | 0 | 0 | 0 | 1 | 0 |
| SH2D2A | 0 | 0 | 0 | 0 | 0 | 1 | 0 |
| MYOM2 | 0 | 0 | 0 | 0 | 0 | 1 | 0 |
| MTLN | 0 | 0 | 0 | 0 | 0 | 1 | 0 |
| BTBD11 | 0 | 0 | 0 | 0 | 0 | 1 | 0 |
| OSGEP | 0 | 0 | 0 | 0 | 0 | 1 | 0 |
| CPNE2 | 0 | 0 | 0 | 0 | 0 | 1 | 0 |
| THTPA | 0 | 0 | 0 | 0 | 0 | 1 | 0 |
| FKBP1B | 0 | 0 | 0 | 0 | 0 | 1 | 0 |
| CHRAC1 | 0 | 0 | 0 | 0 | 0 | 1 | 0 |
| FLJ32255 | 0 | 0 | 0 | 0 | 0 | 1 | 0 |
| TGM2 | 0 | 0 | 0 | 0 | 0 | 1 | 1 |
| CA2 | 0 | 0 | 0 | 0 | 0 | 1 | 0 |
| STMP1 | 0 | 0 | 0 | 0 | 0 | 1 | 0 |
| TRAV13-1 | 0 | 0 | 0 | 0 | 0 | 1 | 0 |
| TAF15 | 0 | 0 | 0 | 0 | 0 | 1 | 0 |
| TMEM250 | 0 | 0 | 0 | 0 | 0 | 1 | 0 |
| ZNF479 | 0 | 0 | 0 | 0 | 0 | 1 | 0 |
| GALNT6 | 0 | 0 | 0 | 0 | 0 | 1 | 1 |
| MICB | 0 | 0 | 0 | 0 | 0 | 1 | 0 |
| FCRL4 | 0 | 0 | 0 | 0 | 0 | 1 | 0 |
| DBN1 | 0 | 0 | 0 | 0 | 0 | 1 | 0 |
| GUCY1B1 | 0 | 0 | 0 | 0 | 0 | 1 | 0 |
| H4C8 | 0 | 0 | 0 | 0 | 0 | 1 | 0 |
| HPCAL1 | 0 | 0 | 0 | 0 | 0 | 1 | 0 |
| MEST | 0 | 0 | 0 | 0 | 0 | 1 | 0 |
| OAZ2 | 0 | 0 | 0 | 0 | 0 | 1 | 0 |
| SELP | 0 | 0 | 0 | 0 | 0 | 1 | 0 |
| FBP1 | 0 | 0 | 0 | 0 | 0 | 1 | 0 |
| NCF4 | 0 | 0 | 0 | 0 | 0 | 1 | 0 |
| TRAV21 | 0 | 0 | 0 | 0 | 0 | 1 | 0 |
| SAP130 | 0 | 0 | 0 | 0 | 0 | 1 | 0 |
| HYMAI | 0 | 0 | 0 | 0 | 0 | 1 | 0 |
| ASPHD2 | 0 | 0 | 0 | 0 | 0 | 1 | 0 |
| KEAP1 | 0 | 0 | 0 | 0 | 0 | 1 | 0 |
| PDZK1IP1 | 0 | 0 | 0 | 0 | 0 | 1 | 0 |
| NEURL4 | 0 | 0 | 0 | 0 | 0 | 1 | 0 |
| CDA | 0 | 0 | 0 | 0 | 0 | 1 | 1 |
| LSP1 | 0 | 0 | 0 | 0 | 0 | 1 | 1 |
| DNAJB2 | 0 | 0 | 0 | 0 | 0 | 1 | 0 |
| UBALD2 | 0 | 0 | 0 | 0 | 0 | 1 | 0 |
| SFRP2 | 0 | 0 | 0 | 0 | 0 | 1 | 0 |
| HBM | 0 | 0 | 0 | 0 | 0 | 1 | 0 |
| EIF2AK1 | 0 | 0 | 0 | 0 | 0 | 1 | 0 |
| TREX1 | 0 | 0 | 0 | 0 | 0 | 1 | 0 |
| ROGDI | 0 | 0 | 0 | 0 | 0 | 1 | 0 |
| MMD | 0 | 0 | 0 | 0 | 0 | 1 | 0 |
| GTSF1 | 0 | 0 | 0 | 0 | 0 | 1 | 0 |
| PDCD6P1 | 0 | 0 | 0 | 0 | 0 | 1 | 0 |
| SLC20A2 | 0 | 0 | 0 | 0 | 0 | 1 | 0 |
| ELOF1 | 0 | 0 | 0 | 0 | 0 | 1 | 0 |
| GUCD1 | 0 | 0 | 0 | 0 | 0 | 1 | 1 |
| INAFM1 | 0 | 0 | 0 | 0 | 0 | 1 | 1 |
| PUDP | 0 | 0 | 0 | 0 | 0 | 1 | 0 |
| RUVBL1 | 0 | 0 | 0 | 0 | 0 | 1 | 0 |
| MSRB1 | 0 | 0 | 0 | 0 | 0 | 1 | 0 |
| STRADB | 0 | 0 | 0 | 0 | 0 | 1 | 1 |
| ANKRD9 | 0 | 0 | 0 | 0 | 0 | 1 | 0 |
| LOC105379173 | 0 | 0 | 0 | 0 | 0 | 1 | 0 |
| POLR1D | 0 | 0 | 0 | 0 | 0 | 1 | 0 |
| ZBTB8OS | 0 | 0 | 0 | 0 | 0 | 1 | 0 |
| SSB | 0 | 0 | 0 | 0 | 0 | 1 | 0 |
| RHOG | 0 | 0 | 0 | 0 | 0 | 1 | 0 |
| NOMO3 | 0 | 0 | 0 | 0 | 0 | 1 | 0 |
| PSMC6 | 0 | 0 | 0 | 0 | 0 | 1 | 0 |
| DCXR | 0 | 0 | 0 | 0 | 0 | 1 | 1 |
| GPAT2 | 0 | 0 | 0 | 0 | 0 | 1 | 0 |
| ALDOAP2 | 0 | 0 | 0 | 0 | 0 | 1 | 0 |
| WDR45 | 0 | 0 | 0 | 0 | 0 | 1 | 0 |
| PGM1 | 0 | 0 | 0 | 0 | 0 | 1 | 0 |
| CR1L | 0 | 0 | 0 | 0 | 0 | 1 | 1 |
| RBM23 | 0 | 0 | 0 | 0 | 0 | 1 | 0 |
| MAGOH | 0 | 0 | 0 | 0 | 0 | 1 | 0 |
| FGF7P3 | 0 | 0 | 0 | 0 | 0 | 1 | 0 |
| C12orf43 | 0 | 0 | 0 | 0 | 0 | 1 | 0 |
| YIPF3 | 0 | 0 | 0 | 0 | 0 | 1 | 0 |
| STAT6 | 0 | 0 | 0 | 0 | 0 | 1 | 0 |
| RASSF10 | 0 | 0 | 0 | 0 | 0 | 1 | 0 |
| PSENEN | 0 | 0 | 0 | 0 | 0 | 1 | 0 |
| U2AF1L4 | 0 | 0 | 0 | 0 | 0 | 1 | 0 |
| C2orf42 | 0 | 0 | 0 | 0 | 0 | 1 | 0 |
| SLC15A3 | 0 | 0 | 0 | 0 | 0 | 1 | 0 |
| CRYL1 | 0 | 0 | 0 | 0 | 0 | 1 | 1 |
| HOXC6 | 0 | 0 | 0 | 0 | 0 | 1 | 0 |
| KIR3DL3 | 0 | 0 | 0 | 0 | 0 | 1 | 0 |
| MST1L | 0 | 0 | 0 | 0 | 0 | 1 | 1 |
| UBAP1 | 0 | 0 | 0 | 0 | 0 | 1 | 0 |
| STX5 | 0 | 0 | 0 | 0 | 0 | 1 | 0 |
| PLD3 | 0 | 0 | 0 | 0 | 0 | 1 | 0 |
| PNP | 0 | 0 | 0 | 0 | 0 | 1 | 0 |
| ATIC | 0 | 0 | 0 | 0 | 0 | 1 | 0 |
| ASCC2 | 0 | 0 | 0 | 0 | 0 | 1 | 0 |
| SHKBP1 | 0 | 0 | 0 | 0 | 0 | 1 | 0 |
| ELMO1 | 0 | 0 | 0 | 0 | 0 | 1 | 0 |
| MT1X | 0 | 0 | 0 | 0 | 0 | 1 | 1 |
| HECW2-AS1 | 0 | 0 | 0 | 0 | 0 | 1 | 0 |
| ZNF542P | 0 | 0 | 0 | 0 | 0 | 1 | 0 |
| ZC3H15 | 0 | 0 | 0 | 0 | 0 | 1 | 0 |
| PSMB9 | 0 | 0 | 0 | 0 | 0 | 1 | 0 |
| LRFN1 | 0 | 0 | 0 | 0 | 0 | 1 | 0 |
| KBTBD3 | 0 | 0 | 0 | 0 | 0 | 1 | 0 |
| H2BC10 | 0 | 0 | 0 | 0 | 0 | 1 | 0 |
| MAP7D1 | 0 | 0 | 0 | 0 | 0 | 1 | 0 |
| LOC105371967 | 0 | 0 | 0 | 0 | 0 | 1 | 0 |
| ITGA2B | 0 | 0 | 0 | 0 | 0 | 1 | 0 |
| H1-2 | 0 | 0 | 0 | 0 | 0 | 1 | 0 |
| UQCRB | 0 | 0 | 0 | 0 | 0 | 1 | 0 |
| CXCL8 | 0 | 0 | 0 | 0 | 0 | 1 | 0 |
| ZNF628 | 0 | 0 | 0 | 0 | 0 | 1 | 0 |
| RECQL | 0 | 0 | 0 | 0 | 0 | 1 | 0 |
| KLHDC8B | 0 | 0 | 0 | 0 | 0 | 1 | 0 |
| EVA1C | 0 | 0 | 0 | 0 | 0 | 1 | 0 |
| PRKAR2B | 0 | 0 | 0 | 0 | 0 | 1 | 1 |
| RRAGD | 0 | 0 | 0 | 0 | 0 | 1 | 0 |
| IPO4 | 0 | 0 | 0 | 0 | 0 | 1 | 0 |
| ROPN1L | 0 | 0 | 0 | 0 | 0 | 1 | 1 |
| TIGD3 | 0 | 0 | 0 | 0 | 0 | 1 | 0 |
| RABAC1 | 0 | 0 | 0 | 0 | 0 | 1 | 0 |
| P2RY12 | 0 | 0 | 0 | 0 | 0 | 1 | 0 |
| SRRD | 0 | 0 | 0 | 0 | 0 | 1 | 0 |
| HBQ1 | 0 | 0 | 0 | 0 | 0 | 1 | 0 |
| SIRPB2 | 0 | 0 | 0 | 0 | 0 | 1 | 0 |
| CLU | 0 | 0 | 0 | 0 | 0 | 1 | 1 |
| HTT | 0 | 0 | 0 | 0 | 0 | 1 | 0 |
| UBL7 | 0 | 0 | 0 | 0 | 0 | 1 | 0 |
| TESC | 0 | 0 | 0 | 0 | 0 | 1 | 0 |
| TMEM268 | 0 | 0 | 0 | 0 | 0 | 1 | 0 |
| VPS51 | 0 | 0 | 0 | 0 | 0 | 1 | 0 |
| MFSD5 | 0 | 0 | 0 | 0 | 0 | 1 | 0 |
| GBP4 | 0 | 0 | 0 | 0 | 0 | 1 | 0 |
| CCNJL | 0 | 0 | 0 | 0 | 0 | 1 | 0 |
| LOC101927166 | 0 | 0 | 0 | 0 | 0 | 1 | 0 |
| SLC43A1 | 0 | 0 | 0 | 0 | 0 | 1 | 1 |
| PEX6 | 0 | 0 | 0 | 0 | 0 | 1 | 0 |
| FXYD6 | 0 | 0 | 0 | 0 | 0 | 1 | 0 |
| RAD23A | 0 | 0 | 0 | 0 | 0 | 1 | 0 |
| TRAPPC10 | 0 | 0 | 0 | 0 | 0 | 1 | 0 |
| CHI3L1 | 0 | 0 | 0 | 0 | 0 | 1 | 1 |
| HAGH | 0 | 0 | 0 | 0 | 0 | 1 | 0 |
| ANK1 | 0 | 0 | 0 | 0 | 0 | 1 | 0 |
| FLOT2 | 0 | 0 | 0 | 0 | 0 | 1 | 0 |
| LBH | 0 | 0 | 0 | 0 | 0 | 1 | 1 |
| VAMP2 | 0 | 0 | 0 | 0 | 0 | 1 | 0 |
| RPF2 | 0 | 0 | 0 | 0 | 0 | 1 | 0 |
| PUSL1 | 0 | 0 | 0 | 0 | 0 | 1 | 0 |
| SPDYE1 | 0 | 0 | 0 | 0 | 0 | 1 | 0 |
| TRAPPC5 | 0 | 0 | 0 | 0 | 0 | 1 | 0 |
| SNRPE | 0 | 0 | 0 | 0 | 0 | 1 | 0 |
| TRIM21 | 0 | 0 | 0 | 0 | 0 | 1 | 0 |
| PPP2R5B | 0 | 0 | 0 | 0 | 0 | 1 | 0 |
| ATP6V1B2 | 0 | 0 | 0 | 0 | 0 | 1 | 0 |
| DPP4 | 0 | 0 | 0 | 0 | 0 | 1 | 0 |
| SIAH2 | 0 | 0 | 0 | 0 | 0 | 1 | 0 |
| GUK1 | 0 | 0 | 0 | 0 | 0 | 1 | 0 |
| FRMD3 | 0 | 0 | 0 | 0 | 0 | 1 | 1 |
| ZNF581 | 0 | 0 | 0 | 0 | 0 | 1 | 0 |
| TMOD1 | 0 | 0 | 0 | 0 | 0 | 1 | 1 |
| ZNF808 | 0 | 0 | 0 | 0 | 0 | 1 | 0 |
| HDGF | 0 | 0 | 0 | 0 | 0 | 1 | 0 |
| LINC00339 | 0 | 0 | 0 | 0 | 0 | 1 | 0 |
| CTSA | 0 | 0 | 0 | 0 | 0 | 1 | 0 |
| HAUS4 | 0 | 0 | 0 | 0 | 0 | 1 | 0 |
| MT1F | 0 | 0 | 0 | 0 | 0 | 1 | 1 |
| DSPP | 0 | 0 | 0 | 0 | 0 | 1 | 0 |
| CD7 | 0 | 0 | 0 | 0 | 0 | 1 | 0 |
| RANBP10 | 0 | 0 | 0 | 0 | 0 | 1 | 0 |
| TRIM58 | 0 | 0 | 0 | 0 | 0 | 1 | 1 |
| GATA1 | 0 | 0 | 0 | 0 | 0 | 1 | 0 |
| PNPLA6 | 0 | 0 | 0 | 0 | 0 | 1 | 1 |
| ENO2 | 0 | 0 | 0 | 0 | 0 | 1 | 0 |
| PGLYRP1 | 0 | 0 | 0 | 0 | 0 | 1 | 0 |
| ADORA3 | 0 | 0 | 0 | 0 | 0 | 1 | 0 |
| HLA-DMA | 0 | 0 | 0 | 0 | 0 | 1 | 0 |
| TMTC1 | 0 | 0 | 0 | 0 | 0 | 1 | 0 |
| CLEC1B | 0 | 0 | 0 | 0 | 0 | 1 | 0 |
| HSF5 | 0 | 0 | 0 | 0 | 0 | 1 | 0 |
| LRRC25 | 0 | 0 | 0 | 0 | 0 | 1 | 0 |
| YPEL4 | 0 | 0 | 0 | 0 | 0 | 1 | 0 |
| SH3BGRL2 | 0 | 0 | 0 | 0 | 0 | 1 | 0 |
| POR | 0 | 0 | 0 | 0 | 0 | 1 | 1 |
| CHURC1 | 0 | 0 | 0 | 0 | 0 | 1 | 0 |
| OCEL1 | 0 | 0 | 0 | 0 | 0 | 1 | 0 |
| AMIGO1 | 0 | 0 | 0 | 0 | 0 | 1 | 0 |
| NDUFS5 | 0 | 0 | 0 | 0 | 0 | 1 | 0 |
| SPATC1L | 0 | 0 | 0 | 0 | 0 | 1 | 0 |
| GRAMD1C | 0 | 0 | 0 | 0 | 0 | 1 | 0 |
| TGM3 | 0 | 0 | 0 | 0 | 0 | 1 | 0 |
| ACCS | 0 | 0 | 0 | 0 | 0 | 1 | 0 |
| BCL2L1 | 0 | 0 | 0 | 0 | 0 | 1 | 0 |
| SLC31A2 | 0 | 0 | 0 | 0 | 0 | 1 | 0 |
| FAHD1 | 0 | 0 | 0 | 0 | 0 | 1 | 0 |
| RAD21-AS1 | 0 | 0 | 0 | 0 | 0 | 1 | 0 |
| CAVIN2 | 0 | 0 | 0 | 0 | 0 | 1 | 1 |
| GPX1 | 0 | 0 | 0 | 0 | 0 | 1 | 0 |
| S100P | 0 | 0 | 0 | 0 | 0 | 1 | 0 |
| PRDX5 | 0 | 0 | 0 | 0 | 0 | 1 | 1 |
| TRANK1 | 0 | 0 | 0 | 0 | 0 | 1 | 0 |
| WBP2 | 0 | 0 | 0 | 0 | 0 | 1 | 0 |
| HMBS | 0 | 0 | 0 | 0 | 0 | 1 | 0 |
| NOP2 | 0 | 0 | 0 | 0 | 0 | 1 | 1 |
| PRKCD | 0 | 0 | 0 | 0 | 0 | 1 | 0 |
| H2BC6 | 0 | 0 | 0 | 0 | 0 | 1 | 0 |
| CD3E | 0 | 0 | 0 | 0 | 0 | 1 | 0 |
| GMPPA | 0 | 0 | 0 | 0 | 0 | 1 | 0 |
| MAF1 | 0 | 0 | 0 | 0 | 0 | 1 | 0 |
| PSMF1 | 0 | 0 | 0 | 0 | 0 | 1 | 0 |
| TREML1 | 0 | 0 | 0 | 0 | 0 | 1 | 0 |
| DPEP3 | 0 | 0 | 0 | 0 | 0 | 1 | 0 |
| CCR1 | 0 | 0 | 0 | 0 | 0 | 1 | 0 |
| DANCR | 0 | 0 | 0 | 0 | 0 | 1 | 1 |
| TNS1 | 0 | 0 | 0 | 0 | 0 | 1 | 0 |
| H2AC6 | 0 | 0 | 0 | 0 | 0 | 1 | 0 |
| MFSD13A | 0 | 0 | 0 | 0 | 0 | 1 | 0 |
| KRT1 | 0 | 0 | 0 | 0 | 0 | 1 | 0 |
| PIGU | 0 | 0 | 0 | 0 | 0 | 1 | 0 |
| TBCA | 0 | 0 | 0 | 0 | 0 | 1 | 0 |
| PLEKHF1 | 0 | 0 | 0 | 0 | 0 | 1 | 1 |
| RAB5B | 0 | 0 | 0 | 0 | 0 | 1 | 0 |
| ANKRD55 | 0 | 0 | 0 | 0 | 0 | 1 | 1 |
| IFRD2 | 0 | 0 | 0 | 0 | 0 | 1 | 0 |
| ITPRIPL1 | 0 | 0 | 0 | 0 | 0 | 1 | 0 |
| GLT1D1 | 0 | 0 | 0 | 0 | 0 | 1 | 1 |
| DCAF12 | 0 | 0 | 0 | 0 | 0 | 1 | 0 |
| POLD2 | 0 | 0 | 0 | 0 | 0 | 1 | 0 |
| ZER1 | 0 | 0 | 0 | 0 | 0 | 1 | 0 |
| SLC27A3 | 0 | 0 | 0 | 0 | 0 | 1 | 1 |
| SAMD12 | 0 | 0 | 0 | 0 | 0 | 1 | 0 |
| MICAL2 | 0 | 0 | 0 | 0 | 0 | 1 | 0 |
| ITLN1 | 0 | 0 | 0 | 0 | 0 | 1 | 0 |
| MYL6B | 0 | 0 | 0 | 0 | 0 | 1 | 0 |
| BBOF1 | 0 | 0 | 0 | 0 | 0 | 1 | 0 |
| KIR2DS5 | 0 | 0 | 0 | 0 | 0 | 1 | 0 |
| ASPRV1 | 0 | 0 | 0 | 0 | 0 | 1 | 0 |
| IL15RA | 0 | 0 | 0 | 0 | 0 | 1 | 0 |
| MBNL3 | 0 | 0 | 0 | 0 | 0 | 1 | 0 |
| CSF1R | 0 | 0 | 0 | 0 | 0 | 1 | 0 |
| CD274 | 0 | 0 | 0 | 0 | 0 | 1 | 0 |
| EIF2D | 0 | 0 | 0 | 0 | 0 | 1 | 0 |
| KLF1 | 0 | 0 | 0 | 0 | 0 | 1 | 0 |
| MYH9 | 0 | 0 | 0 | 0 | 0 | 1 | 0 |
| CWC15 | 0 | 0 | 0 | 0 | 0 | 1 | 0 |
| CFP | 0 | 0 | 0 | 0 | 0 | 1 | 1 |
| ITPRIP | 0 | 0 | 0 | 0 | 0 | 1 | 0 |
| SNORA21 | 0 | 0 | 0 | 0 | 0 | 1 | 0 |
| TENT5C | 0 | 0 | 0 | 0 | 0 | 1 | 0 |
| GSTM2 | 0 | 0 | 0 | 0 | 0 | 1 | 0 |
| EPB42 | 0 | 0 | 0 | 0 | 0 | 1 | 0 |
| DHRS3 | 0 | 0 | 0 | 0 | 0 | 1 | 0 |
| LOC389831 | 0 | 0 | 0 | 0 | 0 | 1 | 0 |
| DHRS13 | 0 | 0 | 0 | 0 | 0 | 1 | 1 |
| LOC105377782 | 0 | 0 | 0 | 0 | 0 | 1 | 0 |
| DYSF | 0 | 0 | 0 | 0 | 0 | 1 | 0 |
| PFDN4 | 0 | 0 | 0 | 0 | 0 | 1 | 1 |
| PDZD11 | 0 | 0 | 0 | 0 | 0 | 1 | 0 |
| NDUFA1 | 0 | 0 | 0 | 0 | 0 | 1 | 0 |
| FAM214B | 0 | 0 | 0 | 0 | 0 | 1 | 0 |
| HMGN3 | 0 | 0 | 0 | 0 | 0 | 1 | 1 |
| FIS1 | 0 | 0 | 0 | 0 | 0 | 1 | 0 |
| FCMR | 0 | 0 | 0 | 0 | 0 | 1 | 0 |
| ZNF600 | 0 | 0 | 0 | 0 | 0 | 1 | 0 |
| HSPE1 | 0 | 0 | 0 | 0 | 0 | 1 | 1 |
| ZNF57 | 0 | 0 | 0 | 0 | 0 | 1 | 0 |
| ABHD14B | 0 | 0 | 0 | 0 | 0 | 1 | 0 |
| BPI | 0 | 0 | 0 | 0 | 0 | 1 | 0 |
| OXTR | 0 | 0 | 0 | 0 | 0 | 1 | 0 |
| HBG2 | 0 | 0 | 0 | 0 | 0 | 1 | 0 |
| EEF1B2 | 0 | 0 | 0 | 0 | 0 | 1 | 0 |
| CTNNAL1 | 0 | 0 | 0 | 0 | 0 | 1 | 0 |
| YBX3 | 0 | 0 | 0 | 0 | 0 | 1 | 1 |
| MED22 | 0 | 0 | 0 | 0 | 0 | 1 | 0 |
| DGAT2 | 0 | 0 | 0 | 0 | 0 | 1 | 1 |
| CLEC4D | 0 | 0 | 0 | 0 | 0 | 1 | 0 |
| LOC101928893 | 0 | 0 | 0 | 0 | 0 | 1 | 0 |
| GMPR | 0 | 0 | 0 | 0 | 0 | 1 | 0 |
| PCSK1N | 0 | 0 | 0 | 0 | 0 | 1 | 0 |
| SHCBP1 | 0 | 0 | 0 | 0 | 0 | 1 | 0 |
| BABAM1 | 0 | 0 | 0 | 0 | 0 | 1 | 0 |
| HLX | 0 | 0 | 0 | 0 | 0 | 1 | 0 |
| BLVRB | 0 | 0 | 0 | 0 | 0 | 1 | 0 |
| STX11 | 0 | 0 | 0 | 0 | 0 | 1 | 0 |
| LRG1 | 0 | 0 | 0 | 0 | 0 | 1 | 1 |
| OSBP2 | 0 | 0 | 0 | 0 | 0 | 1 | 0 |
| TATDN1 | 0 | 0 | 0 | 0 | 0 | 1 | 0 |
| LSM8 | 0 | 0 | 0 | 0 | 0 | 1 | 0 |
| OPTN | 0 | 0 | 0 | 0 | 0 | 1 | 0 |
| ABHD15 | 0 | 0 | 0 | 0 | 0 | 1 | 0 |
| PCTP | 0 | 0 | 0 | 0 | 0 | 1 | 1 |
| SELENOM | 0 | 0 | 0 | 0 | 0 | 1 | 0 |
| H2BC5 | 0 | 0 | 0 | 0 | 0 | 1 | 0 |
| PDCD4-AS1 | 0 | 0 | 0 | 0 | 0 | 1 | 0 |
| NFU1 | 0 | 0 | 0 | 0 | 0 | 1 | 0 |
| SMIM30 | 0 | 0 | 0 | 0 | 0 | 1 | 1 |
| AK6 | 0 | 0 | 0 | 0 | 0 | 1 | 0 |
| ADGRE3 | 0 | 0 | 0 | 0 | 0 | 1 | 0 |
| SLPI | 0 | 0 | 0 | 0 | 0 | 1 | 1 |
| WLS | 0 | 0 | 0 | 0 | 0 | 1 | 1 |
| CETN3 | 0 | 0 | 0 | 0 | 0 | 1 | 0 |
| NME4 | 0 | 0 | 0 | 0 | 0 | 1 | 0 |
| BATF2 | 0 | 0 | 0 | 0 | 0 | 1 | 0 |
| C2orf74 | 0 | 0 | 0 | 0 | 0 | 1 | 0 |
| LINC01527 | 0 | 0 | 0 | 0 | 0 | 1 | 0 |
| ODC1 | 0 | 0 | 0 | 0 | 0 | 1 | 0 |
| PIP4K2A | 0 | 0 | 0 | 0 | 0 | 1 | 0 |
| HAT1 | 0 | 0 | 0 | 0 | 0 | 1 | 0 |
| RGL4 | 0 | 0 | 0 | 0 | 0 | 1 | 0 |
| KLRG1 | 0 | 0 | 0 | 0 | 0 | 1 | 0 |
| PCED1B | 0 | 0 | 0 | 0 | 0 | 1 | 1 |
| TMEM91 | 0 | 0 | 0 | 0 | 0 | 1 | 0 |
| XK | 0 | 0 | 0 | 0 | 0 | 1 | 0 |
| LINC02481 | 0 | 0 | 0 | 0 | 0 | 1 | 0 |
| E2F2 | 0 | 0 | 0 | 0 | 0 | 1 | 0 |
| B3GNT8 | 0 | 0 | 0 | 0 | 0 | 1 | 0 |
| PRXL2B | 0 | 0 | 0 | 0 | 0 | 1 | 0 |
| PLEK2 | 0 | 0 | 0 | 0 | 0 | 1 | 1 |
| RELB | 0 | 0 | 0 | 0 | 0 | 1 | 0 |
| EIF1AY | 0 | 0 | 0 | 0 | 0 | 1 | 0 |
| MAP3K7CL | 0 | 0 | 0 | 0 | 0 | 1 | 0 |
| NFKB1 | 0 | 0 | 0 | 0 | 0 | 1 | 0 |
| CLEC12B | 0 | 0 | 0 | 0 | 0 | 1 | 0 |
| FHIT | 0 | 0 | 0 | 0 | 0 | 1 | 0 |
| ADGRG3 | 0 | 0 | 0 | 0 | 0 | 1 | 0 |
| AHSP | 0 | 0 | 0 | 0 | 0 | 1 | 0 |
| FHL2 | 0 | 0 | 0 | 0 | 0 | 1 | 0 |
| FAM104A | 0 | 0 | 0 | 0 | 0 | 1 | 0 |
| RHBDF2 | 0 | 0 | 0 | 0 | 0 | 1 | 0 |
| LHFPL2 | 0 | 0 | 0 | 0 | 0 | 1 | 0 |
| PSMA4 | 0 | 0 | 0 | 0 | 0 | 1 | 0 |
| SCN1B | 0 | 0 | 0 | 0 | 0 | 1 | 0 |
| PLVAP | 0 | 0 | 0 | 0 | 0 | 1 | 0 |
| DPM2 | 0 | 0 | 0 | 0 | 0 | 1 | 0 |
| ERV3-1 | 0 | 0 | 0 | 0 | 0 | 1 | 0 |
| TSPAN5 | 0 | 0 | 0 | 0 | 0 | 1 | 0 |
| SNRPD1 | 0 | 0 | 0 | 0 | 0 | 1 | 1 |
| OTUD6B-AS1 | 0 | 0 | 0 | 0 | 0 | 1 | 0 |
| TPRKB | 0 | 0 | 0 | 0 | 0 | 1 | 0 |
| GYPB | 0 | 0 | 0 | 0 | 0 | 1 | 0 |
| MAD2L1BP | 0 | 0 | 0 | 0 | 0 | 1 | 0 |
| LOC105375492 | 0 | 0 | 0 | 0 | 0 | 1 | 0 |
| SARNP | 0 | 0 | 0 | 0 | 0 | 1 | 0 |
| TNFSF13 | 0 | 0 | 0 | 0 | 0 | 1 | 0 |
| RWDD3 | 0 | 0 | 0 | 0 | 0 | 1 | 0 |
| ARMCX2 | 0 | 0 | 0 | 0 | 0 | 1 | 0 |
| SELENBP1 | 0 | 0 | 0 | 0 | 0 | 1 | 0 |
| RPL26L1 | 0 | 0 | 0 | 0 | 0 | 1 | 0 |
| LRRC4 | 0 | 0 | 0 | 0 | 0 | 1 | 0 |
| RPL22L1 | 0 | 0 | 0 | 0 | 0 | 1 | 0 |
| TMA7 | 0 | 0 | 0 | 0 | 0 | 1 | 0 |
| CASC3 | 0 | 0 | 0 | 0 | 0 | 1 | 0 |
| CMC1 | 0 | 0 | 0 | 0 | 0 | 1 | 0 |
| UQCRH | 0 | 0 | 0 | 0 | 0 | 1 | 0 |
| P2RX7 | 0 | 0 | 0 | 0 | 0 | 1 | 0 |
| CTSB | 0 | 0 | 0 | 0 | 0 | 1 | 0 |
| CEACAM1 | 0 | 0 | 0 | 0 | 0 | 1 | 1 |
| SECTM1 | 0 | 0 | 0 | 0 | 0 | 1 | 0 |
| ABCC13 | 0 | 0 | 0 | 0 | 0 | 1 | 0 |
| P2RY14 | 0 | 0 | 0 | 0 | 0 | 1 | 0 |
| MARCHF8 | 0 | 0 | 0 | 0 | 0 | 1 | 0 |
| NINJ2 | 0 | 0 | 0 | 0 | 0 | 1 | 0 |
| MRC2 | 0 | 0 | 0 | 0 | 0 | 1 | 0 |
| WARS1 | 0 | 0 | 0 | 0 | 0 | 1 | 0 |
| OASL | 0 | 0 | 0 | 0 | 0 | 1 | 0 |
| NAPSB | 0 | 0 | 0 | 0 | 0 | 1 | 0 |
| FBXO6 | 0 | 0 | 0 | 0 | 0 | 1 | 0 |
| FAM106A | 0 | 0 | 0 | 0 | 0 | 1 | 0 |
| KIR3DL2 | 0 | 0 | 0 | 0 | 0 | 1 | 0 |
| SLC7A5 | 0 | 0 | 0 | 0 | 0 | 1 | 0 |
| RBM38 | 0 | 0 | 0 | 0 | 0 | 1 | 0 |
| CEACAM21 | 0 | 0 | 0 | 0 | 0 | 1 | 0 |
| COX16 | 0 | 0 | 0 | 0 | 0 | 1 | 0 |
| SNRPG | 0 | 0 | 0 | 0 | 0 | 1 | 0 |
| MKRN1 | 0 | 0 | 0 | 0 | 0 | 1 | 0 |
| BSG | 0 | 0 | 0 | 0 | 0 | 1 | 0 |
| LTF | 0 | 0 | 0 | 0 | 0 | 1 | 0 |
| COX6C | 0 | 0 | 0 | 0 | 0 | 1 | 0 |
| RWDD1 | 0 | 0 | 0 | 0 | 0 | 1 | 0 |
| CTSL | 0 | 0 | 0 | 0 | 0 | 1 | 0 |
| PI3 | 0 | 0 | 0 | 0 | 0 | 1 | 0 |
| GLRX5 | 0 | 0 | 0 | 0 | 0 | 1 | 0 |
| PF4 | 0 | 0 | 0 | 0 | 0 | 1 | 0 |
| EMC2 | 0 | 0 | 0 | 0 | 0 | 1 | 0 |
| MX1 | 0 | 0 | 0 | 0 | 0 | 1 | 0 |
| RSL24D1 | 0 | 0 | 0 | 0 | 0 | 1 | 0 |
| BTNL3 | 0 | 0 | 0 | 0 | 0 | 1 | 0 |
| LAMP3 | 0 | 0 | 0 | 0 | 0 | 1 | 0 |
| EPHX2 | 0 | 0 | 0 | 0 | 0 | 1 | 1 |
| RPIA | 0 | 0 | 0 | 0 | 0 | 1 | 1 |
| CEACAM8 | 0 | 0 | 0 | 0 | 0 | 1 | 0 |
| SDCBPP2 | 0 | 0 | 0 | 0 | 0 | 1 | 0 |
| FECH | 0 | 0 | 0 | 0 | 0 | 1 | 0 |
| RPS7 | 0 | 0 | 0 | 0 | 0 | 1 | 0 |
| TUBB1 | 0 | 0 | 0 | 0 | 0 | 1 | 0 |
| LY6E | 0 | 0 | 0 | 0 | 0 | 1 | 0 |
| MRPS28 | 0 | 0 | 0 | 0 | 0 | 1 | 0 |
| BCL2A1 | 0 | 0 | 0 | 0 | 0 | 1 | 0 |
| DPPA3 | 0 | 0 | 0 | 0 | 0 | 1 | 0 |
| SNCA | 0 | 0 | 0 | 0 | 0 | 1 | 1 |
| METTL18 | 0 | 0 | 0 | 0 | 0 | 1 | 0 |
| BPGM | 0 | 0 | 0 | 0 | 0 | 1 | 0 |
| FAM3B | 0 | 0 | 0 | 0 | 0 | 1 | 0 |
| SLC1A5 | 0 | 0 | 0 | 0 | 0 | 1 | 0 |
| RPL34 | 0 | 0 | 0 | 0 | 0 | 1 | 0 |
| KDM7A-DT | 0 | 0 | 0 | 0 | 0 | 1 | 0 |
| C9orf78 | 0 | 0 | 0 | 0 | 0 | 1 | 0 |
| HAUS1 | 0 | 0 | 0 | 0 | 0 | 1 | 0 |
| HEMGN | 0 | 0 | 0 | 0 | 0 | 1 | 0 |
| KANSL1-AS1 | 0 | 0 | 0 | 0 | 0 | 1 | 0 |
| CHMP5 | 0 | 0 | 0 | 0 | 0 | 1 | 0 |
| EPSTI1 | 0 | 0 | 0 | 0 | 0 | 1 | 0 |
| SLIRP | 0 | 0 | 0 | 0 | 0 | 1 | 0 |
| FCGR1B | 0 | 0 | 0 | 0 | 0 | 1 | 0 |
| IFI35 | 0 | 0 | 0 | 0 | 0 | 1 | 1 |
| RSAD2 | 0 | 0 | 0 | 0 | 0 | 1 | 0 |
| FRG1JP | 0 | 0 | 0 | 0 | 0 | 1 | 0 |
| CAMP | 0 | 0 | 0 | 0 | 0 | 1 | 0 |
| MRPL1 | 0 | 0 | 0 | 0 | 0 | 1 | 0 |
| SNORA74B | 0 | 0 | 0 | 0 | 0 | 0 | 1 |
| ALB | 0 | 0 | 0 | 0 | 0 | 0 | 1 |
| APOB | 0 | 0 | 0 | 0 | 0 | 0 | 1 |
| SAA2 | 0 | 0 | 0 | 0 | 0 | 0 | 1 |
| ORM2 | 0 | 0 | 0 | 0 | 0 | 0 | 1 |
| VTN | 0 | 0 | 0 | 0 | 0 | 0 | 1 |
| HPD | 0 | 0 | 0 | 0 | 0 | 0 | 1 |
| TF | 0 | 0 | 0 | 0 | 0 | 0 | 1 |
| DPYS | 0 | 0 | 0 | 0 | 0 | 0 | 1 |
| APOA1 | 0 | 0 | 0 | 0 | 0 | 0 | 1 |
| HGD | 0 | 0 | 0 | 0 | 0 | 0 | 1 |
| CYP2E1 | 0 | 0 | 0 | 0 | 0 | 0 | 1 |
| APOH | 0 | 0 | 0 | 0 | 0 | 0 | 1 |
| GDA | 0 | 0 | 0 | 0 | 0 | 0 | 1 |
| SNORD17 | 0 | 0 | 0 | 0 | 0 | 0 | 1 |
| ABCB4 | 0 | 0 | 0 | 0 | 0 | 0 | 1 |
| RPS2P36 | 0 | 0 | 0 | 0 | 0 | 0 | 1 |
| SLC39A14 | 0 | 0 | 0 | 0 | 0 | 0 | 1 |
| RN7SKP9 | 0 | 0 | 0 | 0 | 0 | 0 | 1 |
| CAV1 | 0 | 0 | 0 | 0 | 0 | 0 | 1 |
| TFR2 | 0 | 0 | 0 | 0 | 0 | 0 | 1 |
| PROC | 0 | 0 | 0 | 0 | 0 | 0 | 1 |
| HIRIP3 | 0 | 0 | 0 | 0 | 0 | 0 | 1 |
| SLC38A4 | 0 | 0 | 0 | 0 | 0 | 0 | 1 |
| UPB1 | 0 | 0 | 0 | 0 | 0 | 0 | 1 |
| SLC51A | 0 | 0 | 0 | 0 | 0 | 0 | 1 |
| LMO7 | 0 | 0 | 0 | 0 | 0 | 0 | 1 |
| PLAC9 | 0 | 0 | 0 | 0 | 0 | 0 | 1 |
| PTPN21 | 0 | 0 | 0 | 0 | 0 | 0 | 1 |
| CYP2C18 | 0 | 0 | 0 | 0 | 0 | 0 | 1 |
| LEAP2 | 0 | 0 | 0 | 0 | 0 | 0 | 1 |
| AK4 | 0 | 0 | 0 | 0 | 0 | 0 | 1 |
| PDCL | 0 | 0 | 0 | 0 | 0 | 0 | 1 |
| TMEM87A | 0 | 0 | 0 | 0 | 0 | 0 | 1 |
| C4BPB | 0 | 0 | 0 | 0 | 0 | 0 | 1 |
| AGMAT | 0 | 0 | 0 | 0 | 0 | 0 | 1 |
| LIMCH1 | 0 | 0 | 0 | 0 | 0 | 0 | 1 |
| FNDC4 | 0 | 0 | 0 | 0 | 0 | 0 | 1 |
| CAB39L | 0 | 0 | 0 | 0 | 0 | 0 | 1 |
| TDO2 | 0 | 0 | 0 | 0 | 0 | 0 | 1 |
| SCN4B | 0 | 0 | 0 | 0 | 0 | 0 | 1 |
| PRSS12 | 0 | 0 | 0 | 0 | 0 | 0 | 1 |
| PTPRH | 0 | 0 | 0 | 0 | 0 | 0 | 1 |
| TPST1 | 0 | 0 | 0 | 0 | 0 | 0 | 1 |
| GJB2 | 0 | 0 | 0 | 0 | 0 | 0 | 1 |
| TMSB4X | 0 | 0 | 0 | 0 | 0 | 0 | 1 |
| AKR7A3 | 0 | 0 | 0 | 0 | 0 | 0 | 1 |
| PON1 | 0 | 0 | 0 | 0 | 0 | 0 | 1 |
| SCARNA5 | 0 | 0 | 0 | 0 | 0 | 0 | 1 |
| MTCO3P23 | 0 | 0 | 0 | 0 | 0 | 0 | 1 |
| F5 | 0 | 0 | 0 | 0 | 0 | 0 | 1 |
| AMDHD1 | 0 | 0 | 0 | 0 | 0 | 0 | 1 |
| FGG | 0 | 0 | 0 | 0 | 0 | 0 | 1 |
| AHNAK | 0 | 0 | 0 | 0 | 0 | 0 | 1 |
| ONECUT2 | 0 | 0 | 0 | 0 | 0 | 0 | 1 |
| RTKN2 | 0 | 0 | 0 | 0 | 0 | 0 | 1 |
| PFKFB1 | 0 | 0 | 0 | 0 | 0 | 0 | 1 |
| SDS | 0 | 0 | 0 | 0 | 0 | 0 | 1 |
| SND1-IT1 | 0 | 0 | 0 | 0 | 0 | 0 | 1 |
| SLC39A5 | 0 | 0 | 0 | 0 | 0 | 0 | 1 |
| PPL | 0 | 0 | 0 | 0 | 0 | 0 | 1 |
| PNPLA3 | 0 | 0 | 0 | 0 | 0 | 0 | 1 |
| SMPDL3A | 0 | 0 | 0 | 0 | 0 | 0 | 1 |
| BEX3 | 0 | 0 | 0 | 0 | 0 | 0 | 1 |
| AQP9 | 0 | 0 | 0 | 0 | 0 | 0 | 1 |
| CP | 0 | 0 | 0 | 0 | 0 | 0 | 1 |
| SPRING1 | 0 | 0 | 0 | 0 | 0 | 0 | 1 |
| AHR | 0 | 0 | 0 | 0 | 0 | 0 | 1 |
| TPPP | 0 | 0 | 0 | 0 | 0 | 0 | 1 |
| SLC22A1 | 0 | 0 | 0 | 0 | 0 | 0 | 1 |
| SERPINA1 | 0 | 0 | 0 | 0 | 0 | 0 | 1 |
| GLS2 | 0 | 0 | 0 | 0 | 0 | 0 | 1 |
| NOP53 | 0 | 0 | 0 | 0 | 0 | 0 | 1 |
| CLEC3B | 0 | 0 | 0 | 0 | 0 | 0 | 1 |
| KCND3 | 0 | 0 | 0 | 0 | 0 | 0 | 1 |
| ENSG00000269688 | 0 | 0 | 0 | 0 | 0 | 0 | 1 |
| PIPOX | 0 | 0 | 0 | 0 | 0 | 0 | 1 |
| DEPDC7 | 0 | 0 | 0 | 0 | 0 | 0 | 1 |
| RIMKLA | 0 | 0 | 0 | 0 | 0 | 0 | 1 |
| CYP21A2 | 0 | 0 | 0 | 0 | 0 | 0 | 1 |
| ANOS1 | 0 | 0 | 0 | 0 | 0 | 0 | 1 |
| ZFHX4 | 0 | 0 | 0 | 0 | 0 | 0 | 1 |
| RPS12P26 | 0 | 0 | 0 | 0 | 0 | 0 | 1 |
| RAB11FIP1 | 0 | 0 | 0 | 0 | 0 | 0 | 1 |
| CAV2 | 0 | 0 | 0 | 0 | 0 | 0 | 1 |
| SMOC1 | 0 | 0 | 0 | 0 | 0 | 0 | 1 |
| DMGDH | 0 | 0 | 0 | 0 | 0 | 0 | 1 |
| HSD11B1 | 0 | 0 | 0 | 0 | 0 | 0 | 1 |
| PRG4 | 0 | 0 | 0 | 0 | 0 | 0 | 1 |
| PROX1 | 0 | 0 | 0 | 0 | 0 | 0 | 1 |
| HAL | 0 | 0 | 0 | 0 | 0 | 0 | 1 |
| HAMP | 0 | 0 | 0 | 0 | 0 | 0 | 1 |
| MLIP | 0 | 0 | 0 | 0 | 0 | 0 | 1 |
| AOX1 | 0 | 0 | 0 | 0 | 0 | 0 | 1 |
| ENSG00000278896 | 0 | 0 | 0 | 0 | 0 | 0 | 1 |
| SEMA5A | 0 | 0 | 0 | 0 | 0 | 0 | 1 |
| RPLP1 | 0 | 0 | 0 | 0 | 0 | 0 | 1 |
| CNDP1 | 0 | 0 | 0 | 0 | 0 | 0 | 1 |
| ADH1C | 0 | 0 | 0 | 0 | 0 | 0 | 1 |
| ZCCHC3 | 0 | 0 | 0 | 0 | 0 | 0 | 1 |
| LIPG | 0 | 0 | 0 | 0 | 0 | 0 | 1 |
| E2F3-IT1 | 0 | 0 | 0 | 0 | 0 | 0 | 1 |
| NR5A2 | 0 | 0 | 0 | 0 | 0 | 0 | 1 |
| CYP3A5 | 0 | 0 | 0 | 0 | 0 | 0 | 1 |
| XDH | 0 | 0 | 0 | 0 | 0 | 0 | 1 |
| ANP32B | 0 | 0 | 0 | 0 | 0 | 0 | 1 |
| PIK3AP1 | 0 | 0 | 0 | 0 | 0 | 0 | 1 |
| CCDC89 | 0 | 0 | 0 | 0 | 0 | 0 | 1 |
| C8G | 0 | 0 | 0 | 0 | 0 | 0 | 1 |
| ENSG00000280365 | 0 | 0 | 0 | 0 | 0 | 0 | 1 |
| MTND5P32 | 0 | 0 | 0 | 0 | 0 | 0 | 1 |
| C1R | 0 | 0 | 0 | 0 | 0 | 0 | 1 |
| ENSG00000275481 | 0 | 0 | 0 | 0 | 0 | 0 | 1 |
| ITIH4 | 0 | 0 | 0 | 0 | 0 | 0 | 1 |
| CCDC115 | 0 | 0 | 0 | 0 | 0 | 0 | 1 |
| PLA2G2A | 0 | 0 | 0 | 0 | 0 | 0 | 1 |
| COL28A1 | 0 | 0 | 0 | 0 | 0 | 0 | 1 |
| SORD | 0 | 0 | 0 | 0 | 0 | 0 | 1 |
| ACTG1P3 | 0 | 0 | 0 | 0 | 0 | 0 | 1 |
| SRD5A1 | 0 | 0 | 0 | 0 | 0 | 0 | 1 |
| ALDH8A1 | 0 | 0 | 0 | 0 | 0 | 0 | 1 |
| PPP1R14A | 0 | 0 | 0 | 0 | 0 | 0 | 1 |
| ALDH1L1 | 0 | 0 | 0 | 0 | 0 | 0 | 1 |
| MT-RNR2 | 0 | 0 | 0 | 0 | 0 | 0 | 1 |
| ZNF704 | 0 | 0 | 0 | 0 | 0 | 0 | 1 |
| RIDA | 0 | 0 | 0 | 0 | 0 | 0 | 1 |
| SEC24D | 0 | 0 | 0 | 0 | 0 | 0 | 1 |
| LIPC | 0 | 0 | 0 | 0 | 0 | 0 | 1 |
| SERPINA3 | 0 | 0 | 0 | 0 | 0 | 0 | 1 |
| MTSS1 | 0 | 0 | 0 | 0 | 0 | 0 | 1 |
| PDSS1 | 0 | 0 | 0 | 0 | 0 | 0 | 1 |
| PATL1 | 0 | 0 | 0 | 0 | 0 | 0 | 1 |
| HSD17B2 | 0 | 0 | 0 | 0 | 0 | 0 | 1 |
| ABAT | 0 | 0 | 0 | 0 | 0 | 0 | 1 |
| PHAF1 | 0 | 0 | 0 | 0 | 0 | 0 | 1 |
| KANK2 | 0 | 0 | 0 | 0 | 0 | 0 | 1 |
| ADAMTS9-AS1 | 0 | 0 | 0 | 0 | 0 | 0 | 1 |
| ENSG00000243696 | 0 | 0 | 0 | 0 | 0 | 0 | 1 |
| ANXA3 | 0 | 0 | 0 | 0 | 0 | 0 | 1 |
| SCARNA21 | 0 | 0 | 0 | 0 | 0 | 0 | 1 |
| CHCHD6 | 0 | 0 | 0 | 0 | 0 | 0 | 1 |
| PPP1R1A | 0 | 0 | 0 | 0 | 0 | 0 | 1 |
| ENSG00000259071 | 0 | 0 | 0 | 0 | 0 | 0 | 1 |
| RPL23AP34 | 0 | 0 | 0 | 0 | 0 | 0 | 1 |
| HELLPAR | 0 | 0 | 0 | 0 | 0 | 0 | 1 |
| ACSM5 | 0 | 0 | 0 | 0 | 0 | 0 | 1 |
| TBX15 | 0 | 0 | 0 | 0 | 0 | 0 | 1 |
| LTBP4 | 0 | 0 | 0 | 0 | 0 | 0 | 1 |
| CDH2 | 0 | 0 | 0 | 0 | 0 | 0 | 1 |
| TNXB | 0 | 0 | 0 | 0 | 0 | 0 | 1 |
| UPK3B | 0 | 0 | 0 | 0 | 0 | 0 | 1 |
| ETV5 | 0 | 0 | 0 | 0 | 0 | 0 | 1 |
| KLKB1 | 0 | 0 | 0 | 0 | 0 | 0 | 1 |
| PLIN1 | 0 | 0 | 0 | 0 | 0 | 0 | 1 |
| CA11 | 0 | 0 | 0 | 0 | 0 | 0 | 1 |
| LPAL2 | 0 | 0 | 0 | 0 | 0 | 0 | 1 |
| RIPOR3 | 0 | 0 | 0 | 0 | 0 | 0 | 1 |
| VEGFD | 0 | 0 | 0 | 0 | 0 | 0 | 1 |
| TSHZ2 | 0 | 0 | 0 | 0 | 0 | 0 | 1 |
| ENSG00000279662 | 0 | 0 | 0 | 0 | 0 | 0 | 1 |
| ANXA1 | 0 | 0 | 0 | 0 | 0 | 0 | 1 |
| HTATSF1 | 0 | 0 | 0 | 0 | 0 | 0 | 1 |
| ENSG00000279573 | 0 | 0 | 0 | 0 | 0 | 0 | 1 |
| ENSG00000278601 | 0 | 0 | 0 | 0 | 0 | 0 | 1 |
| STX1B | 0 | 0 | 0 | 0 | 0 | 0 | 1 |
| PCDHA12 | 0 | 0 | 0 | 0 | 0 | 0 | 1 |
| RN7SKP80 | 0 | 0 | 0 | 0 | 0 | 0 | 1 |
| ABCC11 | 0 | 0 | 0 | 0 | 0 | 0 | 1 |
| RPL7AP16 | 0 | 0 | 0 | 0 | 0 | 0 | 1 |
| NPAS2 | 0 | 0 | 0 | 0 | 0 | 0 | 1 |
| FAM169B | 0 | 0 | 0 | 0 | 0 | 0 | 1 |
| SNORD89 | 0 | 0 | 0 | 0 | 0 | 0 | 1 |
| GATM | 0 | 0 | 0 | 0 | 0 | 0 | 1 |
| CYP4F3 | 0 | 0 | 0 | 0 | 0 | 0 | 1 |
| GLRX | 0 | 0 | 0 | 0 | 0 | 0 | 1 |
| SEC14L2 | 0 | 0 | 0 | 0 | 0 | 0 | 1 |
| RASSF9 | 0 | 0 | 0 | 0 | 0 | 0 | 1 |
| CS | 0 | 0 | 0 | 0 | 0 | 0 | 1 |
| GGA2 | 0 | 0 | 0 | 0 | 0 | 0 | 1 |
| CYP7B1 | 0 | 0 | 0 | 0 | 0 | 0 | 1 |
| EEF1A1P11 | 0 | 0 | 0 | 0 | 0 | 0 | 1 |
| SNORA37 | 0 | 0 | 0 | 0 | 0 | 0 | 1 |
| FAM110D | 0 | 0 | 0 | 0 | 0 | 0 | 1 |
| NEK11 | 0 | 0 | 0 | 0 | 0 | 0 | 1 |
| STEAP1 | 0 | 0 | 0 | 0 | 0 | 0 | 1 |
| RPS6KA5 | 0 | 0 | 0 | 0 | 0 | 0 | 1 |
| OXA1L | 0 | 0 | 0 | 0 | 0 | 0 | 1 |
| SERPINA5 | 0 | 0 | 0 | 0 | 0 | 0 | 1 |
| MGST1 | 0 | 0 | 0 | 0 | 0 | 0 | 1 |
| RSPH1 | 0 | 0 | 0 | 0 | 0 | 0 | 1 |
| ENSG00000279500 | 0 | 0 | 0 | 0 | 0 | 0 | 1 |
| CAVIN1 | 0 | 0 | 0 | 0 | 0 | 0 | 1 |
| PTGR1 | 0 | 0 | 0 | 0 | 0 | 0 | 1 |
| H2BC20P | 0 | 0 | 0 | 0 | 0 | 0 | 1 |
| FAM184A | 0 | 0 | 0 | 0 | 0 | 0 | 1 |
| GPAM | 0 | 0 | 0 | 0 | 0 | 0 | 1 |
| RIBC1 | 0 | 0 | 0 | 0 | 0 | 0 | 1 |
| C1orf198 | 0 | 0 | 0 | 0 | 0 | 0 | 1 |
| HMGN5 | 0 | 0 | 0 | 0 | 0 | 0 | 1 |
| SLC17A9 | 0 | 0 | 0 | 0 | 0 | 0 | 1 |
| LINC02362 | 0 | 0 | 0 | 0 | 0 | 0 | 1 |
| TMOD3 | 0 | 0 | 0 | 0 | 0 | 0 | 1 |
| EFR3B | 0 | 0 | 0 | 0 | 0 | 0 | 1 |
| ADIRF | 0 | 0 | 0 | 0 | 0 | 0 | 1 |
| HS3ST3B1 | 0 | 0 | 0 | 0 | 0 | 0 | 1 |
| CRYZL2P-SEC16B | 0 | 0 | 0 | 0 | 0 | 0 | 1 |
| SLC27A5 | 0 | 0 | 0 | 0 | 0 | 0 | 1 |
| GOLT1A | 0 | 0 | 0 | 0 | 0 | 0 | 1 |
| P3H2 | 0 | 0 | 0 | 0 | 0 | 0 | 1 |
| CLXN | 0 | 0 | 0 | 0 | 0 | 0 | 1 |
| SURF6 | 0 | 0 | 0 | 0 | 0 | 0 | 1 |
| CCDC170 | 0 | 0 | 0 | 0 | 0 | 0 | 1 |
| METTL17 | 0 | 0 | 0 | 0 | 0 | 0 | 1 |
| NECAB1 | 0 | 0 | 0 | 0 | 0 | 0 | 1 |
| ITM2A | 0 | 0 | 0 | 0 | 0 | 0 | 1 |
| WDR72 | 0 | 0 | 0 | 0 | 0 | 0 | 1 |
| LINC01018 | 0 | 0 | 0 | 0 | 0 | 0 | 1 |
| ABCC3 | 0 | 0 | 0 | 0 | 0 | 0 | 1 |
| DNAH10 | 0 | 0 | 0 | 0 | 0 | 0 | 1 |
| TMPRSS6 | 0 | 0 | 0 | 0 | 0 | 0 | 1 |
| UNC5CL | 0 | 0 | 0 | 0 | 0 | 0 | 1 |
| GNMT | 0 | 0 | 0 | 0 | 0 | 0 | 1 |
| ENSG00000261338 | 0 | 0 | 0 | 0 | 0 | 0 | 1 |
| ENSG00000260310 | 0 | 0 | 0 | 0 | 0 | 0 | 1 |
| ANKRD29 | 0 | 0 | 0 | 0 | 0 | 0 | 1 |
| SLC25A18 | 0 | 0 | 0 | 0 | 0 | 0 | 1 |
| GOT1 | 0 | 0 | 0 | 0 | 0 | 0 | 1 |
| CES3 | 0 | 0 | 0 | 0 | 0 | 0 | 1 |
| ZCWPW2 | 0 | 0 | 0 | 0 | 0 | 0 | 1 |
| IL17RB | 0 | 0 | 0 | 0 | 0 | 0 | 1 |
| MGAT3 | 0 | 0 | 0 | 0 | 0 | 0 | 1 |
| RN7SL4P | 0 | 0 | 0 | 0 | 0 | 0 | 1 |
| MAGI3 | 0 | 0 | 0 | 0 | 0 | 0 | 1 |
| CRACDL | 0 | 0 | 0 | 0 | 0 | 0 | 1 |
| C2orf50 | 0 | 0 | 0 | 0 | 0 | 0 | 1 |
| C11orf24 | 0 | 0 | 0 | 0 | 0 | 0 | 1 |
| AQP7P1 | 0 | 0 | 0 | 0 | 0 | 0 | 1 |
| ENTPD5 | 0 | 0 | 0 | 0 | 0 | 0 | 1 |
| SLC35D1 | 0 | 0 | 0 | 0 | 0 | 0 | 1 |
| MOCOS | 0 | 0 | 0 | 0 | 0 | 0 | 1 |
| ENSG00000276980 | 0 | 0 | 0 | 0 | 0 | 0 | 1 |
| SNORD3A | 0 | 0 | 0 | 0 | 0 | 0 | 1 |
| KCND3-IT1 | 0 | 0 | 0 | 0 | 0 | 0 | 1 |
| DRC3 | 0 | 0 | 0 | 0 | 0 | 0 | 1 |
| HIC2 | 0 | 0 | 0 | 0 | 0 | 0 | 1 |
| GPIHBP1 | 0 | 0 | 0 | 0 | 0 | 0 | 1 |
| PPP1R9A | 0 | 0 | 0 | 0 | 0 | 0 | 1 |
| SYT12 | 0 | 0 | 0 | 0 | 0 | 0 | 1 |
| PSAT1 | 0 | 0 | 0 | 0 | 0 | 0 | 1 |
| PC | 0 | 0 | 0 | 0 | 0 | 0 | 1 |
| BMS1P10 | 0 | 0 | 0 | 0 | 0 | 0 | 1 |
| DNAI4 | 0 | 0 | 0 | 0 | 0 | 0 | 1 |
| ABCC2 | 0 | 0 | 0 | 0 | 0 | 0 | 1 |
| LRRC23 | 0 | 0 | 0 | 0 | 0 | 0 | 1 |
| OAF | 0 | 0 | 0 | 0 | 0 | 0 | 1 |
| IER2 | 0 | 0 | 0 | 0 | 0 | 0 | 1 |
| IRX3 | 0 | 0 | 0 | 0 | 0 | 0 | 1 |
| ENSG00000280042 | 0 | 0 | 0 | 0 | 0 | 0 | 1 |
| SLC9B2 | 0 | 0 | 0 | 0 | 0 | 0 | 1 |
| TMEM45A | 0 | 0 | 0 | 0 | 0 | 0 | 1 |
| CFAP43 | 0 | 0 | 0 | 0 | 0 | 0 | 1 |
| ENSG00000227200 | 0 | 0 | 0 | 0 | 0 | 0 | 1 |
| ENSG00000279878 | 0 | 0 | 0 | 0 | 0 | 0 | 1 |
| ADRB1 | 0 | 0 | 0 | 0 | 0 | 0 | 1 |
| MST1P2 | 0 | 0 | 0 | 0 | 0 | 0 | 1 |
| MLXIPL | 0 | 0 | 0 | 0 | 0 | 0 | 1 |
| SUGCT | 0 | 0 | 0 | 0 | 0 | 0 | 1 |
| ABLIM3 | 0 | 0 | 0 | 0 | 0 | 0 | 1 |
| TRIM71 | 0 | 0 | 0 | 0 | 0 | 0 | 1 |
| LINC01252 | 0 | 0 | 0 | 0 | 0 | 0 | 1 |
| H2BC19P | 0 | 0 | 0 | 0 | 0 | 0 | 1 |
| MASP1 | 0 | 0 | 0 | 0 | 0 | 0 | 1 |
| ENSG00000261270 | 0 | 0 | 0 | 0 | 0 | 0 | 1 |
| IRAK2 | 0 | 0 | 0 | 0 | 0 | 0 | 1 |
| MT-RNR1 | 0 | 0 | 0 | 0 | 0 | 0 | 1 |
| PSTPIP2 | 0 | 0 | 0 | 0 | 0 | 0 | 1 |
| TMIGD3 | 0 | 0 | 0 | 0 | 0 | 0 | 1 |
| SLC25A13 | 0 | 0 | 0 | 0 | 0 | 0 | 1 |
| EID1 | 0 | 0 | 0 | 0 | 0 | 0 | 1 |
| LGALS4 | 0 | 0 | 0 | 0 | 0 | 0 | 1 |
| LRP4 | 0 | 0 | 0 | 0 | 0 | 0 | 1 |
| H4C4 | 0 | 0 | 0 | 0 | 0 | 0 | 1 |
| UBTD1 | 0 | 0 | 0 | 0 | 0 | 0 | 1 |
| FAU | 0 | 0 | 0 | 0 | 0 | 0 | 1 |
| IFT140 | 0 | 0 | 0 | 0 | 0 | 0 | 1 |
| DCAF8 | 0 | 0 | 0 | 0 | 0 | 0 | 1 |
| VSIG2 | 0 | 0 | 0 | 0 | 0 | 0 | 1 |
| NEURL3 | 0 | 0 | 0 | 0 | 0 | 0 | 1 |
| ASS1 | 0 | 0 | 0 | 0 | 0 | 0 | 1 |
| TOGARAM2 | 0 | 0 | 0 | 0 | 0 | 0 | 1 |
| NRSN2-AS1 | 0 | 0 | 0 | 0 | 0 | 0 | 1 |
| LYZ | 0 | 0 | 0 | 0 | 0 | 0 | 1 |
| SLC43A3 | 0 | 0 | 0 | 0 | 0 | 0 | 1 |
| ENSG00000093100 | 0 | 0 | 0 | 0 | 0 | 0 | 1 |
| TRIM31 | 0 | 0 | 0 | 0 | 0 | 0 | 1 |
| ARMC12 | 0 | 0 | 0 | 0 | 0 | 0 | 1 |
| NKD1 | 0 | 0 | 0 | 0 | 0 | 0 | 1 |
| BCAM | 0 | 0 | 0 | 0 | 0 | 0 | 1 |
| ENSG00000241886 | 0 | 0 | 0 | 0 | 0 | 0 | 1 |
| TMEM220-AS1 | 0 | 0 | 0 | 0 | 0 | 0 | 1 |
| ENSG00000268573 | 0 | 0 | 0 | 0 | 0 | 0 | 1 |
| RPS3 | 0 | 0 | 0 | 0 | 0 | 0 | 1 |
| ENSG00000225092 | 0 | 0 | 0 | 0 | 0 | 0 | 1 |
| SRSF7 | 0 | 0 | 0 | 0 | 0 | 0 | 1 |
| MNS1 | 0 | 0 | 0 | 0 | 0 | 0 | 1 |
| WNT9A | 0 | 0 | 0 | 0 | 0 | 0 | 1 |
| PAK4 | 0 | 0 | 0 | 0 | 0 | 0 | 1 |
| RN7SKP71 | 0 | 0 | 0 | 0 | 0 | 0 | 1 |
| PPP1R3C | 0 | 0 | 0 | 0 | 0 | 0 | 1 |
| MT-CO2 | 0 | 0 | 0 | 0 | 0 | 0 | 1 |
| SEMA3B | 0 | 0 | 0 | 0 | 0 | 0 | 1 |
| BCL7C | 0 | 0 | 0 | 0 | 0 | 0 | 1 |
| ENSG00000254676 | 0 | 0 | 0 | 0 | 0 | 0 | 1 |
| ENSG00000213058 | 0 | 0 | 0 | 0 | 0 | 0 | 1 |
| H3P31 | 0 | 0 | 0 | 0 | 0 | 0 | 1 |
| TMEM131L | 0 | 0 | 0 | 0 | 0 | 0 | 1 |
| BABAM2 | 0 | 0 | 0 | 0 | 0 | 0 | 1 |
| TSR2 | 0 | 0 | 0 | 0 | 0 | 0 | 1 |
| HCFC1R1 | 0 | 0 | 0 | 0 | 0 | 0 | 1 |
| SERPING1 | 0 | 0 | 0 | 0 | 0 | 0 | 1 |
| PDXDC2P-NPIPB14P | 0 | 0 | 0 | 0 | 0 | 0 | 1 |
| ENSG00000261553 | 0 | 0 | 0 | 0 | 0 | 0 | 1 |
| GCLC | 0 | 0 | 0 | 0 | 0 | 0 | 1 |
| IL17RE | 0 | 0 | 0 | 0 | 0 | 0 | 1 |
| SEC24A | 0 | 0 | 0 | 0 | 0 | 0 | 1 |
| ITCH-IT1 | 0 | 0 | 0 | 0 | 0 | 0 | 1 |
| LETMD1 | 0 | 0 | 0 | 0 | 0 | 0 | 1 |
| NID1 | 0 | 0 | 0 | 0 | 0 | 0 | 1 |
| ID4 | 0 | 0 | 0 | 0 | 0 | 0 | 1 |
| ERBB4 | 0 | 0 | 0 | 0 | 0 | 0 | 1 |
| ENSG00000270120 | 0 | 0 | 0 | 0 | 0 | 0 | 1 |
| BLVRA | 0 | 0 | 0 | 0 | 0 | 0 | 1 |
| USP27X | 0 | 0 | 0 | 0 | 0 | 0 | 1 |
| TSTD1 | 0 | 0 | 0 | 0 | 0 | 0 | 1 |
| SOCS2 | 0 | 0 | 0 | 0 | 0 | 0 | 1 |
| POLR3H | 0 | 0 | 0 | 0 | 0 | 0 | 1 |
| LMNTD2-AS1 | 0 | 0 | 0 | 0 | 0 | 0 | 1 |
| ELOVL6 | 0 | 0 | 0 | 0 | 0 | 0 | 1 |
| ENSG00000265421 | 0 | 0 | 0 | 0 | 0 | 0 | 1 |
| MTURN | 0 | 0 | 0 | 0 | 0 | 0 | 1 |
| DNALI1 | 0 | 0 | 0 | 0 | 0 | 0 | 1 |
| PAPSS1 | 0 | 0 | 0 | 0 | 0 | 0 | 1 |
| LINC01852 | 0 | 0 | 0 | 0 | 0 | 0 | 1 |
| SLC16A4-AS1 | 0 | 0 | 0 | 0 | 0 | 0 | 1 |
| SLC35G1 | 0 | 0 | 0 | 0 | 0 | 0 | 1 |
| NFS1 | 0 | 0 | 0 | 0 | 0 | 0 | 1 |
| CDC42BPG | 0 | 0 | 0 | 0 | 0 | 0 | 1 |
| ENSG00000272369 | 0 | 0 | 0 | 0 | 0 | 0 | 1 |
| SLC9A8 | 0 | 0 | 0 | 0 | 0 | 0 | 1 |
| SNCG | 0 | 0 | 0 | 0 | 0 | 0 | 1 |
| MTCO3P12 | 0 | 0 | 0 | 0 | 0 | 0 | 1 |
| STEAP3 | 0 | 0 | 0 | 0 | 0 | 0 | 1 |
| C1orf35 | 0 | 0 | 0 | 0 | 0 | 0 | 1 |
| COPS9 | 0 | 0 | 0 | 0 | 0 | 0 | 1 |
| NSUN6 | 0 | 0 | 0 | 0 | 0 | 0 | 1 |
| RPS10 | 0 | 0 | 0 | 0 | 0 | 0 | 1 |
| CYP21A1P | 0 | 0 | 0 | 0 | 0 | 0 | 1 |
| ENSG00000273451 | 0 | 0 | 0 | 0 | 0 | 0 | 1 |
| DENND2A | 0 | 0 | 0 | 0 | 0 | 0 | 1 |
| ETNK2 | 0 | 0 | 0 | 0 | 0 | 0 | 1 |
| GAMT | 0 | 0 | 0 | 0 | 0 | 0 | 1 |
| BFSP1 | 0 | 0 | 0 | 0 | 0 | 0 | 1 |
| MPHOSPH8 | 0 | 0 | 0 | 0 | 0 | 0 | 1 |
| GOT2 | 0 | 0 | 0 | 0 | 0 | 0 | 1 |
| ENSG00000280384 | 0 | 0 | 0 | 0 | 0 | 0 | 1 |
| NTAN1P2 | 0 | 0 | 0 | 0 | 0 | 0 | 1 |
| SPN | 0 | 0 | 0 | 0 | 0 | 0 | 1 |
| DNAI7 | 0 | 0 | 0 | 0 | 0 | 0 | 1 |
| ZNF486 | 0 | 0 | 0 | 0 | 0 | 0 | 1 |
| TMEM92 | 0 | 0 | 0 | 0 | 0 | 0 | 1 |
| ENSG00000258561 | 0 | 0 | 0 | 0 | 0 | 0 | 1 |
| EHHADH | 0 | 0 | 0 | 0 | 0 | 0 | 1 |
| CROCC | 0 | 0 | 0 | 0 | 0 | 0 | 1 |
| TFG | 0 | 0 | 0 | 0 | 0 | 0 | 1 |
| SNORA7B | 0 | 0 | 0 | 0 | 0 | 0 | 1 |
| CETN2 | 0 | 0 | 0 | 0 | 0 | 0 | 1 |
| ST6GAL1 | 0 | 0 | 0 | 0 | 0 | 0 | 1 |
| FMO2 | 0 | 0 | 0 | 0 | 0 | 0 | 1 |
| SRPK1 | 0 | 0 | 0 | 0 | 0 | 0 | 1 |
| SCN8A | 0 | 0 | 0 | 0 | 0 | 0 | 1 |
| TSPAN12 | 0 | 0 | 0 | 0 | 0 | 0 | 1 |
| CGN | 0 | 0 | 0 | 0 | 0 | 0 | 1 |
| TLE5 | 0 | 0 | 0 | 0 | 0 | 0 | 1 |
| LRRC1 | 0 | 0 | 0 | 0 | 0 | 0 | 1 |
| ENSG00000270116 | 0 | 0 | 0 | 0 | 0 | 0 | 1 |
| ZSWIM9 | 0 | 0 | 0 | 0 | 0 | 0 | 1 |
| MTCH1P1 | 0 | 0 | 0 | 0 | 0 | 0 | 1 |
| HGFAC | 0 | 0 | 0 | 0 | 0 | 0 | 1 |
| JHY | 0 | 0 | 0 | 0 | 0 | 0 | 1 |
| MTCH2 | 0 | 0 | 0 | 0 | 0 | 0 | 1 |
| SLC13A3 | 0 | 0 | 0 | 0 | 0 | 0 | 1 |
| PON3 | 0 | 0 | 0 | 0 | 0 | 0 | 1 |
| MT-CO3 | 0 | 0 | 0 | 0 | 0 | 0 | 1 |
| FAM110C | 0 | 0 | 0 | 0 | 0 | 0 | 1 |
| OVGP1 | 0 | 0 | 0 | 0 | 0 | 0 | 1 |
| SLC7A2-IT1 | 0 | 0 | 0 | 0 | 0 | 0 | 1 |
| SEC61A2 | 0 | 0 | 0 | 0 | 0 | 0 | 1 |
| LINC01138 | 0 | 0 | 0 | 0 | 0 | 0 | 1 |
| PLD5P1 | 0 | 0 | 0 | 0 | 0 | 0 | 1 |
| ENSG00000276934 | 0 | 0 | 0 | 0 | 0 | 0 | 1 |
| ENSG00000279433 | 0 | 0 | 0 | 0 | 0 | 0 | 1 |
| TUBB4BP2 | 0 | 0 | 0 | 0 | 0 | 0 | 1 |
| MT-CO1 | 0 | 0 | 0 | 0 | 0 | 0 | 1 |
| PARD6B | 0 | 0 | 0 | 0 | 0 | 0 | 1 |
| NDUFV2 | 0 | 0 | 0 | 0 | 0 | 0 | 1 |
| ENSG00000267904 | 0 | 0 | 0 | 0 | 0 | 0 | 1 |
| HSPE1P26 | 0 | 0 | 0 | 0 | 0 | 0 | 1 |
| MTCO1P12 | 0 | 0 | 0 | 0 | 0 | 0 | 1 |
| EMCN | 0 | 0 | 0 | 0 | 0 | 0 | 1 |
| MIR23AHG | 0 | 0 | 0 | 0 | 0 | 0 | 1 |
| LRRIQ1 | 0 | 0 | 0 | 0 | 0 | 0 | 1 |
| STX18-IT1 | 0 | 0 | 0 | 0 | 0 | 0 | 1 |
| SNORA12 | 0 | 0 | 0 | 0 | 0 | 0 | 1 |
| ENSG00000287002 | 0 | 0 | 0 | 0 | 0 | 0 | 1 |
| IRAK1BP1 | 0 | 0 | 0 | 0 | 0 | 0 | 1 |
| SDHAF4 | 0 | 0 | 0 | 0 | 0 | 0 | 1 |
| ZNF578 | 0 | 0 | 0 | 0 | 0 | 0 | 1 |
| TNFRSF11B | 0 | 0 | 0 | 0 | 0 | 0 | 1 |
| FTLP2 | 0 | 0 | 0 | 0 | 0 | 0 | 1 |
| TMTC2 | 0 | 0 | 0 | 0 | 0 | 0 | 1 |
| CLDN1 | 0 | 0 | 0 | 0 | 0 | 0 | 1 |
| RPS15AP10 | 0 | 0 | 0 | 0 | 0 | 0 | 1 |
| PLEKHB1 | 0 | 0 | 0 | 0 | 0 | 0 | 1 |
| MYL6 | 0 | 0 | 0 | 0 | 0 | 0 | 1 |
| ZNF428 | 0 | 0 | 0 | 0 | 0 | 0 | 1 |
| NES | 0 | 0 | 0 | 0 | 0 | 0 | 1 |
| HM13 | 0 | 0 | 0 | 0 | 0 | 0 | 1 |
| CHORDC1P4 | 0 | 0 | 0 | 0 | 0 | 0 | 1 |
| LINC01347 | 0 | 0 | 0 | 0 | 0 | 0 | 1 |
| UQCC1 | 0 | 0 | 0 | 0 | 0 | 0 | 1 |
| SLIT2 | 0 | 0 | 0 | 0 | 0 | 0 | 1 |
| LINC01290 | 0 | 0 | 0 | 0 | 0 | 0 | 1 |
| SORD2P | 0 | 0 | 0 | 0 | 0 | 0 | 1 |
| SERPINE1 | 0 | 0 | 0 | 0 | 0 | 0 | 1 |
| SHFL | 0 | 0 | 0 | 0 | 0 | 0 | 1 |
| CD52 | 0 | 0 | 0 | 0 | 0 | 0 | 1 |
| PSMA2 | 0 | 0 | 0 | 0 | 0 | 0 | 1 |
| CD36 | 0 | 0 | 0 | 0 | 0 | 0 | 1 |
| RPL7L1P3 | 0 | 0 | 0 | 0 | 0 | 0 | 1 |
| RGS6 | 0 | 0 | 0 | 0 | 0 | 0 | 1 |
| MED14 | 0 | 0 | 0 | 0 | 0 | 0 | 1 |
| UBE4A | 0 | 0 | 0 | 0 | 0 | 0 | 1 |
| SLC19A1 | 0 | 0 | 0 | 0 | 0 | 0 | 1 |
| OR7A19P | 0 | 0 | 0 | 0 | 0 | 0 | 1 |
| SNORD94 | 0 | 0 | 0 | 0 | 0 | 0 | 1 |
| ACAD8 | 0 | 0 | 0 | 0 | 0 | 0 | 1 |
| RPS3A | 0 | 0 | 0 | 0 | 0 | 0 | 1 |
| ENSG00000269967 | 0 | 0 | 0 | 0 | 0 | 0 | 1 |
| IDE | 0 | 0 | 0 | 0 | 0 | 0 | 1 |
| MYO1C | 0 | 0 | 0 | 0 | 0 | 0 | 1 |
| FNDC3B | 0 | 0 | 0 | 0 | 0 | 0 | 1 |
| JUND | 0 | 0 | 0 | 0 | 0 | 0 | 1 |
| IGSF3 | 0 | 0 | 0 | 0 | 0 | 0 | 1 |
| FADS1 | 0 | 0 | 0 | 0 | 0 | 0 | 1 |
| ENSG00000278983 | 0 | 0 | 0 | 0 | 0 | 0 | 1 |
| UBXN1 | 0 | 0 | 0 | 0 | 0 | 0 | 1 |
| ACVR1B | 0 | 0 | 0 | 0 | 0 | 0 | 1 |
| TMEM117 | 0 | 0 | 0 | 0 | 0 | 0 | 1 |
| CHST15 | 0 | 0 | 0 | 0 | 0 | 0 | 1 |
| GRINA | 0 | 0 | 0 | 0 | 0 | 0 | 1 |
| ENSG00000232499 | 0 | 0 | 0 | 0 | 0 | 0 | 1 |
| DECR2 | 0 | 0 | 0 | 0 | 0 | 0 | 1 |
| ZNF570 | 0 | 0 | 0 | 0 | 0 | 0 | 1 |
| ENSG00000276517 | 0 | 0 | 0 | 0 | 0 | 0 | 1 |
| THAP11 | 0 | 0 | 0 | 0 | 0 | 0 | 1 |
| TREM2 | 0 | 0 | 0 | 0 | 0 | 0 | 1 |
| TSKU | 0 | 0 | 0 | 0 | 0 | 0 | 1 |
| SLC16A2 | 0 | 0 | 0 | 0 | 0 | 0 | 1 |
| URAHP | 0 | 0 | 0 | 0 | 0 | 0 | 1 |
| ACO1 | 0 | 0 | 0 | 0 | 0 | 0 | 1 |
| ODAD4 | 0 | 0 | 0 | 0 | 0 | 0 | 1 |
| SERPINF1 | 0 | 0 | 0 | 0 | 0 | 0 | 1 |
| RPL21P65 | 0 | 0 | 0 | 0 | 0 | 0 | 1 |
| RPL36AP33 | 0 | 0 | 0 | 0 | 0 | 0 | 1 |
| MOB3B | 0 | 0 | 0 | 0 | 0 | 0 | 1 |
| ENSG00000272941 | 0 | 0 | 0 | 0 | 0 | 0 | 1 |
| RN7SKP16 | 0 | 0 | 0 | 0 | 0 | 0 | 1 |
| RNU6-130P | 0 | 0 | 0 | 0 | 0 | 0 | 1 |
| RDH11 | 0 | 0 | 0 | 0 | 0 | 0 | 1 |
| SULT1B1 | 0 | 0 | 0 | 0 | 0 | 0 | 1 |
| F11 | 0 | 0 | 0 | 0 | 0 | 0 | 1 |
| GCH1 | 0 | 0 | 0 | 0 | 0 | 0 | 1 |
| ENSG00000273243 | 0 | 0 | 0 | 0 | 0 | 0 | 1 |
| ENSG00000232034 | 0 | 0 | 0 | 0 | 0 | 0 | 1 |
| PLIN2 | 0 | 0 | 0 | 0 | 0 | 0 | 1 |
| ACADSB | 0 | 0 | 0 | 0 | 0 | 0 | 1 |
| RN7SKP283 | 0 | 0 | 0 | 0 | 0 | 0 | 1 |
| ANG | 0 | 0 | 0 | 0 | 0 | 0 | 1 |
| CAPN5 | 0 | 0 | 0 | 0 | 0 | 0 | 1 |
| CEP112 | 0 | 0 | 0 | 0 | 0 | 0 | 1 |
| GK-IT1 | 0 | 0 | 0 | 0 | 0 | 0 | 1 |
| SFXN1 | 0 | 0 | 0 | 0 | 0 | 0 | 1 |
| VAMP1 | 0 | 0 | 0 | 0 | 0 | 0 | 1 |
| NDUFB8 | 0 | 0 | 0 | 0 | 0 | 0 | 1 |
| RPS9 | 0 | 0 | 0 | 0 | 0 | 0 | 1 |
| GRB10 | 0 | 0 | 0 | 0 | 0 | 0 | 1 |
| PDK1 | 0 | 0 | 0 | 0 | 0 | 0 | 1 |
| ENSG00000267587 | 0 | 0 | 0 | 0 | 0 | 0 | 1 |
| PBLD | 0 | 0 | 0 | 0 | 0 | 0 | 1 |
| IFT57 | 0 | 0 | 0 | 0 | 0 | 0 | 1 |
| ITGAE | 0 | 0 | 0 | 0 | 0 | 0 | 1 |
| CTH | 0 | 0 | 0 | 0 | 0 | 0 | 1 |
| ENSG00000269044 | 0 | 0 | 0 | 0 | 0 | 0 | 1 |
| ENSG00000271984 | 0 | 0 | 0 | 0 | 0 | 0 | 1 |
| PMM1 | 0 | 0 | 0 | 0 | 0 | 0 | 1 |
| POM121L9P | 0 | 0 | 0 | 0 | 0 | 0 | 1 |
| SPINT2 | 0 | 0 | 0 | 0 | 0 | 0 | 1 |
| SS18L1 | 0 | 0 | 0 | 0 | 0 | 0 | 1 |
| ANPEP | 0 | 0 | 0 | 0 | 0 | 0 | 1 |
| RBP5 | 0 | 0 | 0 | 0 | 0 | 0 | 1 |
| MSR1 | 0 | 0 | 0 | 0 | 0 | 0 | 1 |
| PDLIM1P4 | 0 | 0 | 0 | 0 | 0 | 0 | 1 |
| CRTAC1 | 0 | 0 | 0 | 0 | 0 | 0 | 1 |
| MARS2 | 0 | 0 | 0 | 0 | 0 | 0 | 1 |
| PARP16 | 0 | 0 | 0 | 0 | 0 | 0 | 1 |
| ENSG00000267385 | 0 | 0 | 0 | 0 | 0 | 0 | 1 |
| GNG11 | 0 | 0 | 0 | 0 | 0 | 0 | 1 |
| DDB2 | 0 | 0 | 0 | 0 | 0 | 0 | 1 |
| DAPK1 | 0 | 0 | 0 | 0 | 0 | 0 | 1 |
| HHEX | 0 | 0 | 0 | 0 | 0 | 0 | 1 |
| HYDIN | 0 | 0 | 0 | 0 | 0 | 0 | 1 |
| CLTCL1 | 0 | 0 | 0 | 0 | 0 | 0 | 1 |
| MORF4L1P1 | 0 | 0 | 0 | 0 | 0 | 0 | 1 |
| ENO1P3 | 0 | 0 | 0 | 0 | 0 | 0 | 1 |
| MT1G | 0 | 0 | 0 | 0 | 0 | 0 | 1 |
| PHAX | 0 | 0 | 0 | 0 | 0 | 0 | 1 |
| PDE8A | 0 | 0 | 0 | 0 | 0 | 0 | 1 |
| HADHB | 0 | 0 | 0 | 0 | 0 | 0 | 1 |
| TMEM177 | 0 | 0 | 0 | 0 | 0 | 0 | 1 |
| PON2 | 0 | 0 | 0 | 0 | 0 | 0 | 1 |
| SERINC2 | 0 | 0 | 0 | 0 | 0 | 0 | 1 |
| GADD45GIP1 | 0 | 0 | 0 | 0 | 0 | 0 | 1 |
| ENSG00000267430 | 0 | 0 | 0 | 0 | 0 | 0 | 1 |
| NIT2 | 0 | 0 | 0 | 0 | 0 | 0 | 1 |
| SLC26A1 | 0 | 0 | 0 | 0 | 0 | 0 | 1 |
| CCL20 | 0 | 0 | 0 | 0 | 0 | 0 | 1 |
| CRIP2 | 0 | 0 | 0 | 0 | 0 | 0 | 1 |
| MAB21L4 | 0 | 0 | 0 | 0 | 0 | 0 | 1 |
| RPS19 | 0 | 0 | 0 | 0 | 0 | 0 | 1 |
| VAMP8 | 0 | 0 | 0 | 0 | 0 | 0 | 1 |
| ENSG00000279250 | 0 | 0 | 0 | 0 | 0 | 0 | 1 |
| MPHOSPH9 | 0 | 0 | 0 | 0 | 0 | 0 | 1 |
| DNAJC25 | 0 | 0 | 0 | 0 | 0 | 0 | 1 |
| CLDN12 | 0 | 0 | 0 | 0 | 0 | 0 | 1 |
| HDGFL2 | 0 | 0 | 0 | 0 | 0 | 0 | 1 |
| ZBTB25 | 0 | 0 | 0 | 0 | 0 | 0 | 1 |
| TRIM27 | 0 | 0 | 0 | 0 | 0 | 0 | 1 |
| ZNF219 | 0 | 0 | 0 | 0 | 0 | 0 | 1 |
| GPD1L | 0 | 0 | 0 | 0 | 0 | 0 | 1 |
| ENSG00000241489 | 0 | 0 | 0 | 0 | 0 | 0 | 1 |
| LACTB2 | 0 | 0 | 0 | 0 | 0 | 0 | 1 |
| MRO | 0 | 0 | 0 | 0 | 0 | 0 | 1 |
| CCDC148 | 0 | 0 | 0 | 0 | 0 | 0 | 1 |
| H4C3 | 0 | 0 | 0 | 0 | 0 | 0 | 1 |
| SOX18 | 0 | 0 | 0 | 0 | 0 | 0 | 1 |
| PHKA2 | 0 | 0 | 0 | 0 | 0 | 0 | 1 |
| CCN4 | 0 | 0 | 0 | 0 | 0 | 0 | 1 |
| ENSG00000281091 | 0 | 0 | 0 | 0 | 0 | 0 | 1 |
| CLSTN3 | 0 | 0 | 0 | 0 | 0 | 0 | 1 |
| ENKD1 | 0 | 0 | 0 | 0 | 0 | 0 | 1 |
| UBTF | 0 | 0 | 0 | 0 | 0 | 0 | 1 |
| ENSG00000279236 | 0 | 0 | 0 | 0 | 0 | 0 | 1 |
| PLCD3 | 0 | 0 | 0 | 0 | 0 | 0 | 1 |
| PLOD2 | 0 | 0 | 0 | 0 | 0 | 0 | 1 |
| ZNF207 | 0 | 0 | 0 | 0 | 0 | 0 | 1 |
| ZNF813 | 0 | 0 | 0 | 0 | 0 | 0 | 1 |
| BTG2 | 0 | 0 | 0 | 0 | 0 | 0 | 1 |
| CAPS | 0 | 0 | 0 | 0 | 0 | 0 | 1 |
| ENSG00000279838 | 0 | 0 | 0 | 0 | 0 | 0 | 1 |
| MTND2P28 | 0 | 0 | 0 | 0 | 0 | 0 | 1 |
| MYOM3 | 0 | 0 | 0 | 0 | 0 | 0 | 1 |
| ENSG00000270426 | 0 | 0 | 0 | 0 | 0 | 0 | 1 |
| ARHGAP18 | 0 | 0 | 0 | 0 | 0 | 0 | 1 |
| CYP4F12 | 0 | 0 | 0 | 0 | 0 | 0 | 1 |
| ENSG00000266709 | 0 | 0 | 0 | 0 | 0 | 0 | 1 |
| SYVN1 | 0 | 0 | 0 | 0 | 0 | 0 | 1 |
| ENSG00000256981 | 0 | 0 | 0 | 0 | 0 | 0 | 1 |
| VASH1 | 0 | 0 | 0 | 0 | 0 | 0 | 1 |
| PLLP | 0 | 0 | 0 | 0 | 0 | 0 | 1 |
| HNRNPA0 | 0 | 0 | 0 | 0 | 0 | 0 | 1 |
| FDPSP3 | 0 | 0 | 0 | 0 | 0 | 0 | 1 |
| NHSL1-AS1 | 0 | 0 | 0 | 0 | 0 | 0 | 1 |
| KHK | 0 | 0 | 0 | 0 | 0 | 0 | 1 |
| RPS3AP26 | 0 | 0 | 0 | 0 | 0 | 0 | 1 |
| ENSG00000278266 | 0 | 0 | 0 | 0 | 0 | 0 | 1 |
| PIAS2 | 0 | 0 | 0 | 0 | 0 | 0 | 1 |
| TEDC1 | 0 | 0 | 0 | 0 | 0 | 0 | 1 |
| ZNF14 | 0 | 0 | 0 | 0 | 0 | 0 | 1 |
| PDAP1 | 0 | 0 | 0 | 0 | 0 | 0 | 1 |
| SART1 | 0 | 0 | 0 | 0 | 0 | 0 | 1 |
| ZNF557 | 0 | 0 | 0 | 0 | 0 | 0 | 1 |
| ENSG00000266805 | 0 | 0 | 0 | 0 | 0 | 0 | 1 |
| PZP | 0 | 0 | 0 | 0 | 0 | 0 | 1 |
| FAM174B | 0 | 0 | 0 | 0 | 0 | 0 | 1 |
| WASF1 | 0 | 0 | 0 | 0 | 0 | 0 | 1 |
| NWD1 | 0 | 0 | 0 | 0 | 0 | 0 | 1 |
| B3GALNT1 | 0 | 0 | 0 | 0 | 0 | 0 | 1 |
| ENSG00000260261 | 0 | 0 | 0 | 0 | 0 | 0 | 1 |
| LARP4P | 0 | 0 | 0 | 0 | 0 | 0 | 1 |
| ENSG00000224972 | 0 | 0 | 0 | 0 | 0 | 0 | 1 |
| YIF1A | 0 | 0 | 0 | 0 | 0 | 0 | 1 |
| SPIN3 | 0 | 0 | 0 | 0 | 0 | 0 | 1 |
| ENSG00000276850 | 0 | 0 | 0 | 0 | 0 | 0 | 1 |
| RRAS | 0 | 0 | 0 | 0 | 0 | 0 | 1 |
| RPS25 | 0 | 0 | 0 | 0 | 0 | 0 | 1 |
| RPS21 | 0 | 0 | 0 | 0 | 0 | 0 | 1 |
| FAM20A | 0 | 0 | 0 | 0 | 0 | 0 | 1 |
| USP32 | 0 | 0 | 0 | 0 | 0 | 0 | 1 |
| HGF | 0 | 0 | 0 | 0 | 0 | 0 | 1 |
| SIAE | 0 | 0 | 0 | 0 | 0 | 0 | 1 |
| LYG1 | 0 | 0 | 0 | 0 | 0 | 0 | 1 |
| LIFR-AS1 | 0 | 0 | 0 | 0 | 0 | 0 | 1 |
| METTL21AP1 | 0 | 0 | 0 | 0 | 0 | 0 | 1 |
| SYPL2 | 0 | 0 | 0 | 0 | 0 | 0 | 1 |
| SEPTIN4-AS1 | 0 | 0 | 0 | 0 | 0 | 0 | 1 |
| MATK | 0 | 0 | 0 | 0 | 0 | 0 | 1 |
| MMRN2 | 0 | 0 | 0 | 0 | 0 | 0 | 1 |
| TSPYL1 | 0 | 0 | 0 | 0 | 0 | 0 | 1 |
| GLRB | 0 | 0 | 0 | 0 | 0 | 0 | 1 |
| TWSG1 | 0 | 0 | 0 | 0 | 0 | 0 | 1 |
| RNF152 | 0 | 0 | 0 | 0 | 0 | 0 | 1 |
| RFX2 | 0 | 0 | 0 | 0 | 0 | 0 | 1 |
| ENSG00000279814 | 0 | 0 | 0 | 0 | 0 | 0 | 1 |
| ENSG00000225527 | 0 | 0 | 0 | 0 | 0 | 0 | 1 |
| RNU4ATAC11P | 0 | 0 | 0 | 0 | 0 | 0 | 1 |
| SRPRA | 0 | 0 | 0 | 0 | 0 | 0 | 1 |
| GRK5 | 0 | 0 | 0 | 0 | 0 | 0 | 1 |
| SLC35B1 | 0 | 0 | 0 | 0 | 0 | 0 | 1 |
| ENSG00000279319 | 0 | 0 | 0 | 0 | 0 | 0 | 1 |
| AOC2 | 0 | 0 | 0 | 0 | 0 | 0 | 1 |
| PPP2R1B | 0 | 0 | 0 | 0 | 0 | 0 | 1 |
| NR1D1 | 0 | 0 | 0 | 0 | 0 | 0 | 1 |
| YJU2 | 0 | 0 | 0 | 0 | 0 | 0 | 1 |
| ENSG00000285725 | 0 | 0 | 0 | 0 | 0 | 0 | 1 |
| XIRP1 | 0 | 0 | 0 | 0 | 0 | 0 | 1 |
| DNAAF1 | 0 | 0 | 0 | 0 | 0 | 0 | 1 |
| CEBPB-AS1 | 0 | 0 | 0 | 0 | 0 | 0 | 1 |
| DLEC1 | 0 | 0 | 0 | 0 | 0 | 0 | 1 |
| ALDH6A1 | 0 | 0 | 0 | 0 | 0 | 0 | 1 |
| CDCA7L | 0 | 0 | 0 | 0 | 0 | 0 | 1 |
| SPTA1 | 0 | 0 | 0 | 0 | 0 | 0 | 1 |
| EBLN2 | 0 | 0 | 0 | 0 | 0 | 0 | 1 |
| PPBP | 0 | 0 | 0 | 0 | 0 | 0 | 1 |
| TOR3A | 0 | 0 | 0 | 0 | 0 | 0 | 1 |
| WIPI1 | 0 | 0 | 0 | 0 | 0 | 0 | 1 |
| USHBP1 | 0 | 0 | 0 | 0 | 0 | 0 | 1 |
| HAAO | 0 | 0 | 0 | 0 | 0 | 0 | 1 |
| PRPF6 | 0 | 0 | 0 | 0 | 0 | 0 | 1 |
| PLXNA2 | 0 | 0 | 0 | 0 | 0 | 0 | 1 |
| ENSG00000224967 | 0 | 0 | 0 | 0 | 0 | 0 | 1 |
| PGAM1P8 | 0 | 0 | 0 | 0 | 0 | 0 | 1 |
| SNX22 | 0 | 0 | 0 | 0 | 0 | 0 | 1 |
| PTS | 0 | 0 | 0 | 0 | 0 | 0 | 1 |
| OTULINL | 0 | 0 | 0 | 0 | 0 | 0 | 1 |
| TTC30B | 0 | 0 | 0 | 0 | 0 | 0 | 1 |
| EEF1A1P19 | 0 | 0 | 0 | 0 | 0 | 0 | 1 |
| ADAT2 | 0 | 0 | 0 | 0 | 0 | 0 | 1 |
| ICAM5 | 0 | 0 | 0 | 0 | 0 | 0 | 1 |
| C14orf132 | 0 | 0 | 0 | 0 | 0 | 0 | 1 |
| SLC23A2 | 0 | 0 | 0 | 0 | 0 | 0 | 1 |
| ZCCHC2 | 0 | 0 | 0 | 0 | 0 | 0 | 1 |
| STARD5 | 0 | 0 | 0 | 0 | 0 | 0 | 1 |
| SCYL2P1 | 0 | 0 | 0 | 0 | 0 | 0 | 1 |
| GPT2 | 0 | 0 | 0 | 0 | 0 | 0 | 1 |
| HMGCLL1 | 0 | 0 | 0 | 0 | 0 | 0 | 1 |
| TUBE1 | 0 | 0 | 0 | 0 | 0 | 0 | 1 |
| DNAJB13 | 0 | 0 | 0 | 0 | 0 | 0 | 1 |
| LGALS8 | 0 | 0 | 0 | 0 | 0 | 0 | 1 |
| SLC35E1P1 | 0 | 0 | 0 | 0 | 0 | 0 | 1 |
| CEBPZOS | 0 | 0 | 0 | 0 | 0 | 0 | 1 |
| REEP6 | 0 | 0 | 0 | 0 | 0 | 0 | 1 |
| RAPGEF3 | 0 | 0 | 0 | 0 | 0 | 0 | 1 |
| H1-10-AS1 | 0 | 0 | 0 | 0 | 0 | 0 | 1 |
| PI15 | 0 | 0 | 0 | 0 | 0 | 0 | 1 |
| ENSG00000279159 | 0 | 0 | 0 | 0 | 0 | 0 | 1 |
| PTPN3 | 0 | 0 | 0 | 0 | 0 | 0 | 1 |
| AKAP13 | 0 | 0 | 0 | 0 | 0 | 0 | 1 |
| HLA-DPB1 | 0 | 0 | 0 | 0 | 0 | 0 | 1 |
| STOX1 | 0 | 0 | 0 | 0 | 0 | 0 | 1 |
| MRPS23 | 0 | 0 | 0 | 0 | 0 | 0 | 1 |
| DHTKD1 | 0 | 0 | 0 | 0 | 0 | 0 | 1 |
| TACC1 | 0 | 0 | 0 | 0 | 0 | 0 | 1 |
| ENSG00000274677 | 0 | 0 | 0 | 0 | 0 | 0 | 1 |
| MAGEH1 | 0 | 0 | 0 | 0 | 0 | 0 | 1 |
| ACSS2 | 0 | 0 | 0 | 0 | 0 | 0 | 1 |
| CATIP | 0 | 0 | 0 | 0 | 0 | 0 | 1 |
| SLC7A2 | 0 | 0 | 0 | 0 | 0 | 0 | 1 |
| NOL7 | 0 | 0 | 0 | 0 | 0 | 0 | 1 |
| ZSCAN18 | 0 | 0 | 0 | 0 | 0 | 0 | 1 |
| RPL13AP5 | 0 | 0 | 0 | 0 | 0 | 0 | 1 |
| CCDC9 | 0 | 0 | 0 | 0 | 0 | 0 | 1 |
| WHAMMP3 | 0 | 0 | 0 | 0 | 0 | 0 | 1 |
| ACAT2 | 0 | 0 | 0 | 0 | 0 | 0 | 1 |
| CCDC65 | 0 | 0 | 0 | 0 | 0 | 0 | 1 |
| MFAP1 | 0 | 0 | 0 | 0 | 0 | 0 | 1 |
| PHLDA3 | 0 | 0 | 0 | 0 | 0 | 0 | 1 |
| PCCA | 0 | 0 | 0 | 0 | 0 | 0 | 1 |
| AQP4 | 0 | 0 | 0 | 0 | 0 | 0 | 1 |
| SEC31A | 0 | 0 | 0 | 0 | 0 | 0 | 1 |
| NCL | 0 | 0 | 0 | 0 | 0 | 0 | 1 |
| SPRY2 | 0 | 0 | 0 | 0 | 0 | 0 | 1 |
| MRPS10 | 0 | 0 | 0 | 0 | 0 | 0 | 1 |
| LINC02348 | 0 | 0 | 0 | 0 | 0 | 0 | 1 |
| SOX17 | 0 | 0 | 0 | 0 | 0 | 0 | 1 |
| SCARNA6 | 0 | 0 | 0 | 0 | 0 | 0 | 1 |
| SLC16A7 | 0 | 0 | 0 | 0 | 0 | 0 | 1 |
| CLUAP1 | 0 | 0 | 0 | 0 | 0 | 0 | 1 |
| TAS2R3 | 0 | 0 | 0 | 0 | 0 | 0 | 1 |
| MPC1 | 0 | 0 | 0 | 0 | 0 | 0 | 1 |
| ARMCX1 | 0 | 0 | 0 | 0 | 0 | 0 | 1 |
| ENSG00000244313 | 0 | 0 | 0 | 0 | 0 | 0 | 1 |
| TBCC | 0 | 0 | 0 | 0 | 0 | 0 | 1 |
| OBSL1 | 0 | 0 | 0 | 0 | 0 | 0 | 1 |
| PDCL3 | 0 | 0 | 0 | 0 | 0 | 0 | 1 |
| TGDS | 0 | 0 | 0 | 0 | 0 | 0 | 1 |
| ENSG00000276649 | 0 | 0 | 0 | 0 | 0 | 0 | 1 |
| ARRB1 | 0 | 0 | 0 | 0 | 0 | 0 | 1 |
| FANK1 | 0 | 0 | 0 | 0 | 0 | 0 | 1 |
| MFSD14A | 0 | 0 | 0 | 0 | 0 | 0 | 1 |
| MT-CYB | 0 | 0 | 0 | 0 | 0 | 0 | 1 |
| CLIC5 | 0 | 0 | 0 | 0 | 0 | 0 | 1 |
| FZD1 | 0 | 0 | 0 | 0 | 0 | 0 | 1 |
| ALPK1 | 0 | 0 | 0 | 0 | 0 | 0 | 1 |
| ZDHHC19 | 0 | 0 | 0 | 0 | 0 | 0 | 1 |
| ENSG00000261051 | 0 | 0 | 0 | 0 | 0 | 0 | 1 |
| BICD2 | 0 | 0 | 0 | 0 | 0 | 0 | 1 |
| SYT7 | 0 | 0 | 0 | 0 | 0 | 0 | 1 |
| NCAPH2 | 0 | 0 | 0 | 0 | 0 | 0 | 1 |
| SLC2A10 | 0 | 0 | 0 | 0 | 0 | 0 | 1 |
| IL1R2 | 0 | 0 | 0 | 0 | 0 | 0 | 1 |
| SLC38A7 | 0 | 0 | 0 | 0 | 0 | 0 | 1 |
| SRP14 | 0 | 0 | 0 | 0 | 0 | 0 | 1 |
| AOC4P | 0 | 0 | 0 | 0 | 0 | 0 | 1 |
| GRN | 0 | 0 | 0 | 0 | 0 | 0 | 1 |
| ATRN | 0 | 0 | 0 | 0 | 0 | 0 | 1 |
| CCDC112 | 0 | 0 | 0 | 0 | 0 | 0 | 1 |
| AMOTL2 | 0 | 0 | 0 | 0 | 0 | 0 | 1 |
| ENSG00000286314 | 0 | 0 | 0 | 0 | 0 | 0 | 1 |
| ENSG00000260865 | 0 | 0 | 0 | 0 | 0 | 0 | 1 |
| TLE2 | 0 | 0 | 0 | 0 | 0 | 0 | 1 |
| EPS8L1 | 0 | 0 | 0 | 0 | 0 | 0 | 1 |
| SPRYD3 | 0 | 0 | 0 | 0 | 0 | 0 | 1 |
| SMAD9 | 0 | 0 | 0 | 0 | 0 | 0 | 1 |
| C7orf50 | 0 | 0 | 0 | 0 | 0 | 0 | 1 |
| HSPA12B | 0 | 0 | 0 | 0 | 0 | 0 | 1 |
| KLF6 | 0 | 0 | 0 | 0 | 0 | 0 | 1 |
| ST20 | 0 | 0 | 0 | 0 | 0 | 0 | 1 |
| ENSG00000279544 | 0 | 0 | 0 | 0 | 0 | 0 | 1 |
| STAB2 | 0 | 0 | 0 | 0 | 0 | 0 | 1 |
| IDH1 | 0 | 0 | 0 | 0 | 0 | 0 | 1 |
| MTND4P12 | 0 | 0 | 0 | 0 | 0 | 0 | 1 |
| ADGRV1 | 0 | 0 | 0 | 0 | 0 | 0 | 1 |
| ZNF700 | 0 | 0 | 0 | 0 | 0 | 0 | 1 |
| CEP89 | 0 | 0 | 0 | 0 | 0 | 0 | 1 |
| FREM2 | 0 | 0 | 0 | 0 | 0 | 0 | 1 |
| CBLC | 0 | 0 | 0 | 0 | 0 | 0 | 1 |
| GAPDHP43 | 0 | 0 | 0 | 0 | 0 | 0 | 1 |
| SNORD62B | 0 | 0 | 0 | 0 | 0 | 0 | 1 |
| ENSG00000277999 | 0 | 0 | 0 | 0 | 0 | 0 | 1 |
| GLYCTK | 0 | 0 | 0 | 0 | 0 | 0 | 1 |
| PIKFYVE | 0 | 0 | 0 | 0 | 0 | 0 | 1 |
| SECISBP2L | 0 | 0 | 0 | 0 | 0 | 0 | 1 |
| PRX | 0 | 0 | 0 | 0 | 0 | 0 | 1 |
| LARP1B | 0 | 0 | 0 | 0 | 0 | 0 | 1 |
| SPRYD4 | 0 | 0 | 0 | 0 | 0 | 0 | 1 |
| GRAMD2B | 0 | 0 | 0 | 0 | 0 | 0 | 1 |
| RPRD1B | 0 | 0 | 0 | 0 | 0 | 0 | 1 |
| PLBD1 | 0 | 0 | 0 | 0 | 0 | 0 | 1 |
| ENSG00000261560 | 0 | 0 | 0 | 0 | 0 | 0 | 1 |
| DNAJC12 | 0 | 0 | 0 | 0 | 0 | 0 | 1 |
| C4B | 0 | 0 | 0 | 0 | 0 | 0 | 1 |
| ZNF653 | 0 | 0 | 0 | 0 | 0 | 0 | 1 |
| COG3 | 0 | 0 | 0 | 0 | 0 | 0 | 1 |
| TMEM37 | 0 | 0 | 0 | 0 | 0 | 0 | 1 |
| ITGA8 | 0 | 0 | 0 | 0 | 0 | 0 | 1 |
| XPNPEP1 | 0 | 0 | 0 | 0 | 0 | 0 | 1 |
| ENSG00000234405 | 0 | 0 | 0 | 0 | 0 | 0 | 1 |
| ENSG00000230333 | 0 | 0 | 0 | 0 | 0 | 0 | 1 |
| ZC3H4 | 0 | 0 | 0 | 0 | 0 | 0 | 1 |
| PCDHB12 | 0 | 0 | 0 | 0 | 0 | 0 | 1 |
| TTPAL | 0 | 0 | 0 | 0 | 0 | 0 | 1 |
| SLC46A3 | 0 | 0 | 0 | 0 | 0 | 0 | 1 |
| ENSG00000261596 | 0 | 0 | 0 | 0 | 0 | 0 | 1 |
| ENSG00000225213 | 0 | 0 | 0 | 0 | 0 | 0 | 1 |
| GPC6 | 0 | 0 | 0 | 0 | 0 | 0 | 1 |
| BACH1-IT3 | 0 | 0 | 0 | 0 | 0 | 0 | 1 |
| ENSG00000240291 | 0 | 0 | 0 | 0 | 0 | 0 | 1 |
| WWC2 | 0 | 0 | 0 | 0 | 0 | 0 | 1 |
| TSR3 | 0 | 0 | 0 | 0 | 0 | 0 | 1 |
| HBB | 0 | 0 | 0 | 0 | 0 | 0 | 1 |
| SNAI2 | 0 | 0 | 0 | 0 | 0 | 0 | 1 |
| SSTR2 | 0 | 0 | 0 | 0 | 0 | 0 | 1 |
| NUDT7 | 0 | 0 | 0 | 0 | 0 | 0 | 1 |
| SYNGR1 | 0 | 0 | 0 | 0 | 0 | 0 | 1 |
| FBXO17 | 0 | 0 | 0 | 0 | 0 | 0 | 1 |
| COLCA1 | 0 | 0 | 0 | 0 | 0 | 0 | 1 |
| BEX4 | 0 | 0 | 0 | 0 | 0 | 0 | 1 |
| NR0B2 | 0 | 0 | 0 | 0 | 0 | 0 | 1 |
| TES | 0 | 0 | 0 | 0 | 0 | 0 | 1 |
| SLC35C1 | 0 | 0 | 0 | 0 | 0 | 0 | 1 |
| ENSG00000258578 | 0 | 0 | 0 | 0 | 0 | 0 | 1 |
| ENSG00000279786 | 0 | 0 | 0 | 0 | 0 | 0 | 1 |
| SUPV3L1 | 0 | 0 | 0 | 0 | 0 | 0 | 1 |
| ZNF250 | 0 | 0 | 0 | 0 | 0 | 0 | 1 |
| CD1D | 0 | 0 | 0 | 0 | 0 | 0 | 1 |
| ZBTB4 | 0 | 0 | 0 | 0 | 0 | 0 | 1 |
| ADGRA3 | 0 | 0 | 0 | 0 | 0 | 0 | 1 |
| FAM53B | 0 | 0 | 0 | 0 | 0 | 0 | 1 |
| IGSF9B | 0 | 0 | 0 | 0 | 0 | 0 | 1 |
| HMG20B | 0 | 0 | 0 | 0 | 0 | 0 | 1 |
| SPATA41 | 0 | 0 | 0 | 0 | 0 | 0 | 1 |
| WTIP | 0 | 0 | 0 | 0 | 0 | 0 | 1 |
| SETP6 | 0 | 0 | 0 | 0 | 0 | 0 | 1 |
| KYAT1 | 0 | 0 | 0 | 0 | 0 | 0 | 1 |
| HNRNPA1 | 0 | 0 | 0 | 0 | 0 | 0 | 1 |
| KLF2 | 0 | 0 | 0 | 0 | 0 | 0 | 1 |
| OLFML2A | 0 | 0 | 0 | 0 | 0 | 0 | 1 |
| RN7SKP90 | 0 | 0 | 0 | 0 | 0 | 0 | 1 |
| CHDH | 0 | 0 | 0 | 0 | 0 | 0 | 1 |
| TUT7 | 0 | 0 | 0 | 0 | 0 | 0 | 1 |
| DMRTA1 | 0 | 0 | 0 | 0 | 0 | 0 | 1 |
| MRPL37 | 0 | 0 | 0 | 0 | 0 | 0 | 1 |
| AVPR1A | 0 | 0 | 0 | 0 | 0 | 0 | 1 |
| SLC30A7 | 0 | 0 | 0 | 0 | 0 | 0 | 1 |
| JUN | 0 | 0 | 0 | 0 | 0 | 0 | 1 |
| LRATD1 | 0 | 0 | 0 | 0 | 0 | 0 | 1 |
| SLC38A9 | 0 | 0 | 0 | 0 | 0 | 0 | 1 |
| TSPOAP1 | 0 | 0 | 0 | 0 | 0 | 0 | 1 |
| TTC38 | 0 | 0 | 0 | 0 | 0 | 0 | 1 |
| ENSG00000272140 | 0 | 0 | 0 | 0 | 0 | 0 | 1 |
| KIAA1143 | 0 | 0 | 0 | 0 | 0 | 0 | 1 |
| ABCA5 | 0 | 0 | 0 | 0 | 0 | 0 | 1 |
| VWA3A | 0 | 0 | 0 | 0 | 0 | 0 | 1 |
| DLC1 | 0 | 0 | 0 | 0 | 0 | 0 | 1 |
| EIPR1-IT1 | 0 | 0 | 0 | 0 | 0 | 0 | 1 |
| ENSG00000231181 | 0 | 0 | 0 | 0 | 0 | 0 | 1 |
| RPL21P5 | 0 | 0 | 0 | 0 | 0 | 0 | 1 |
| ENSG00000248794 | 0 | 0 | 0 | 0 | 0 | 0 | 1 |
| PWWP2B | 0 | 0 | 0 | 0 | 0 | 0 | 1 |
| ABCA1 | 0 | 0 | 0 | 0 | 0 | 0 | 1 |
| PPT1 | 0 | 0 | 0 | 0 | 0 | 0 | 1 |
| FGF10 | 0 | 0 | 0 | 0 | 0 | 0 | 1 |
| SPAG5-AS1 | 0 | 0 | 0 | 0 | 0 | 0 | 1 |
| ENSG00000260064 | 0 | 0 | 0 | 0 | 0 | 0 | 1 |
| PTMS | 0 | 0 | 0 | 0 | 0 | 0 | 1 |
| RNF165 | 0 | 0 | 0 | 0 | 0 | 0 | 1 |
| LINC00907 | 0 | 0 | 0 | 0 | 0 | 0 | 1 |
| STEEP1 | 0 | 0 | 0 | 0 | 0 | 0 | 1 |
| GAS6 | 0 | 0 | 0 | 0 | 0 | 0 | 1 |
| ENSG00000280321 | 0 | 0 | 0 | 0 | 0 | 0 | 1 |
| RPL23AP7 | 0 | 0 | 0 | 0 | 0 | 0 | 1 |
| RPL32P32 | 0 | 0 | 0 | 0 | 0 | 0 | 1 |
| NIM1K | 0 | 0 | 0 | 0 | 0 | 0 | 1 |
| MRRF | 0 | 0 | 0 | 0 | 0 | 0 | 1 |
| HNRNPA1P10 | 0 | 0 | 0 | 0 | 0 | 0 | 1 |
| TYRO3 | 0 | 0 | 0 | 0 | 0 | 0 | 1 |
| RNU4ATAC18P | 0 | 0 | 0 | 0 | 0 | 0 | 1 |
| FGD5 | 0 | 0 | 0 | 0 | 0 | 0 | 1 |
| PUS3 | 0 | 0 | 0 | 0 | 0 | 0 | 1 |
| SP6 | 0 | 0 | 0 | 0 | 0 | 0 | 1 |
| TRMT112 | 0 | 0 | 0 | 0 | 0 | 0 | 1 |
| BMP6 | 0 | 0 | 0 | 0 | 0 | 0 | 1 |
| BCO2 | 0 | 0 | 0 | 0 | 0 | 0 | 1 |
| ZNF821 | 0 | 0 | 0 | 0 | 0 | 0 | 1 |
| DHODH | 0 | 0 | 0 | 0 | 0 | 0 | 1 |
| KIAA1328 | 0 | 0 | 0 | 0 | 0 | 0 | 1 |
| YIPF1 | 0 | 0 | 0 | 0 | 0 | 0 | 1 |
| CA1 | 0 | 0 | 0 | 0 | 0 | 0 | 1 |
| C12orf57 | 0 | 0 | 0 | 0 | 0 | 0 | 1 |
| MAPK8IP2 | 0 | 0 | 0 | 0 | 0 | 0 | 1 |
| ENSG00000279196 | 0 | 0 | 0 | 0 | 0 | 0 | 1 |
| WT1 | 0 | 0 | 0 | 0 | 0 | 0 | 1 |
| CBX7 | 0 | 0 | 0 | 0 | 0 | 0 | 1 |
| ZBTB22 | 0 | 0 | 0 | 0 | 0 | 0 | 1 |
| DENND6B | 0 | 0 | 0 | 0 | 0 | 0 | 1 |
| PDZD2 | 0 | 0 | 0 | 0 | 0 | 0 | 1 |
| ENSG00000259682 | 0 | 0 | 0 | 0 | 0 | 0 | 1 |
| ANKRD49P2 | 0 | 0 | 0 | 0 | 0 | 0 | 1 |
| ARPC5 | 0 | 0 | 0 | 0 | 0 | 0 | 1 |
| ACAA2 | 0 | 0 | 0 | 0 | 0 | 0 | 1 |
| ENSG00000232545 | 0 | 0 | 0 | 0 | 0 | 0 | 1 |
| ENSG00000227615 | 0 | 0 | 0 | 0 | 0 | 0 | 1 |
| ZNF574 | 0 | 0 | 0 | 0 | 0 | 0 | 1 |
| CCDC71 | 0 | 0 | 0 | 0 | 0 | 0 | 1 |
| ZNF784 | 0 | 0 | 0 | 0 | 0 | 0 | 1 |
| TMEM11-DT | 0 | 0 | 0 | 0 | 0 | 0 | 1 |
| PNMA1 | 0 | 0 | 0 | 0 | 0 | 0 | 1 |
| ALDH4A1 | 0 | 0 | 0 | 0 | 0 | 0 | 1 |
| LINC00607 | 0 | 0 | 0 | 0 | 0 | 0 | 1 |
| ZNF563 | 0 | 0 | 0 | 0 | 0 | 0 | 1 |
| SNRPD3 | 0 | 0 | 0 | 0 | 0 | 0 | 1 |
| ALYREF | 0 | 0 | 0 | 0 | 0 | 0 | 1 |
| IGHA2 | 0 | 0 | 0 | 0 | 0 | 0 | 1 |
| ZNF431 | 0 | 0 | 0 | 0 | 0 | 0 | 1 |
| CENPB | 0 | 0 | 0 | 0 | 0 | 0 | 1 |
| PRKCH-AS1 | 0 | 0 | 0 | 0 | 0 | 0 | 1 |
| SOD1-DT | 0 | 0 | 0 | 0 | 0 | 0 | 1 |
| TRIR | 0 | 0 | 0 | 0 | 0 | 0 | 1 |
| PLCXD2 | 0 | 0 | 0 | 0 | 0 | 0 | 1 |
| ENSG00000259453 | 0 | 0 | 0 | 0 | 0 | 0 | 1 |
| COX10 | 0 | 0 | 0 | 0 | 0 | 0 | 1 |
| AR | 0 | 0 | 0 | 0 | 0 | 0 | 1 |
| NPTN-IT1 | 0 | 0 | 0 | 0 | 0 | 0 | 1 |
| ENSG00000261578 | 0 | 0 | 0 | 0 | 0 | 0 | 1 |
| C8orf76 | 0 | 0 | 0 | 0 | 0 | 0 | 1 |
| NUDT14 | 0 | 0 | 0 | 0 | 0 | 0 | 1 |
| CYB561 | 0 | 0 | 0 | 0 | 0 | 0 | 1 |
| MYL3 | 0 | 0 | 0 | 0 | 0 | 0 | 1 |
| HMGN2P5 | 0 | 0 | 0 | 0 | 0 | 0 | 1 |
| DAPK2 | 0 | 0 | 0 | 0 | 0 | 0 | 1 |
| H2AC21 | 0 | 0 | 0 | 0 | 0 | 0 | 1 |
| CD69 | 0 | 0 | 0 | 0 | 0 | 0 | 1 |
| LPGAT1 | 0 | 0 | 0 | 0 | 0 | 0 | 1 |
| VMP1 | 0 | 0 | 0 | 0 | 0 | 0 | 1 |
| MPRIP | 0 | 0 | 0 | 0 | 0 | 0 | 1 |
| ABCB6 | 0 | 0 | 0 | 0 | 0 | 0 | 1 |
| ADAM20P1 | 0 | 0 | 0 | 0 | 0 | 0 | 1 |
| RNU6-1045P | 0 | 0 | 0 | 0 | 0 | 0 | 1 |
| ERBB2 | 0 | 0 | 0 | 0 | 0 | 0 | 1 |
| GLIPR2 | 0 | 0 | 0 | 0 | 0 | 0 | 1 |
| RPS14 | 0 | 0 | 0 | 0 | 0 | 0 | 1 |
| CMYA5 | 0 | 0 | 0 | 0 | 0 | 0 | 1 |
| NCR3LG1 | 0 | 0 | 0 | 0 | 0 | 0 | 1 |
| PITRM1 | 0 | 0 | 0 | 0 | 0 | 0 | 1 |
| GGH | 0 | 0 | 0 | 0 | 0 | 0 | 1 |
| VDAC1 | 0 | 0 | 0 | 0 | 0 | 0 | 1 |
| GSDMC | 0 | 0 | 0 | 0 | 0 | 0 | 1 |
| COL21A1 | 0 | 0 | 0 | 0 | 0 | 0 | 1 |
| ENSG00000283828 | 0 | 0 | 0 | 0 | 0 | 0 | 1 |
| AGRN | 0 | 0 | 0 | 0 | 0 | 0 | 1 |
| RILPL1 | 0 | 0 | 0 | 0 | 0 | 0 | 1 |
| CCDC124 | 0 | 0 | 0 | 0 | 0 | 0 | 1 |
| SHROOM1 | 0 | 0 | 0 | 0 | 0 | 0 | 1 |
| HCG11 | 0 | 0 | 0 | 0 | 0 | 0 | 1 |
| RPSAP6 | 0 | 0 | 0 | 0 | 0 | 0 | 1 |
| DYRK1B | 0 | 0 | 0 | 0 | 0 | 0 | 1 |
| ACTG1P17 | 0 | 0 | 0 | 0 | 0 | 0 | 1 |
| ENSG00000281195 | 0 | 0 | 0 | 0 | 0 | 0 | 1 |
| LINC01355 | 0 | 0 | 0 | 0 | 0 | 0 | 1 |
| HKDC1 | 0 | 0 | 0 | 0 | 0 | 0 | 1 |
| DNAL4 | 0 | 0 | 0 | 0 | 0 | 0 | 1 |
| SS18 | 0 | 0 | 0 | 0 | 0 | 0 | 1 |
| FLRT3 | 0 | 0 | 0 | 0 | 0 | 0 | 1 |
| RN7SL141P | 0 | 0 | 0 | 0 | 0 | 0 | 1 |
| TCF21 | 0 | 0 | 0 | 0 | 0 | 0 | 1 |
| CLBA1 | 0 | 0 | 0 | 0 | 0 | 0 | 1 |
| LRPAP1 | 0 | 0 | 0 | 0 | 0 | 0 | 1 |
| UFD1 | 0 | 0 | 0 | 0 | 0 | 0 | 1 |
| ENSG00000253180 | 0 | 0 | 0 | 0 | 0 | 0 | 1 |
| CASZ1 | 0 | 0 | 0 | 0 | 0 | 0 | 1 |
| ENSG00000258215 | 0 | 0 | 0 | 0 | 0 | 0 | 1 |
| SLAIN1 | 0 | 0 | 0 | 0 | 0 | 0 | 1 |
| ABRAXAS1 | 0 | 0 | 0 | 0 | 0 | 0 | 1 |
| ZBTB46 | 0 | 0 | 0 | 0 | 0 | 0 | 1 |
| HSBP1L1 | 0 | 0 | 0 | 0 | 0 | 0 | 1 |
| F10 | 0 | 0 | 0 | 0 | 0 | 0 | 1 |
| WDFY3-AS2 | 0 | 0 | 0 | 0 | 0 | 0 | 1 |
| PKHD1 | 0 | 0 | 0 | 0 | 0 | 0 | 1 |
| SENP3 | 0 | 0 | 0 | 0 | 0 | 0 | 1 |
| DENND5B | 0 | 0 | 0 | 0 | 0 | 0 | 1 |
| CPVL | 0 | 0 | 0 | 0 | 0 | 0 | 1 |
| SMAD1 | 0 | 0 | 0 | 0 | 0 | 0 | 1 |
| ZBTB8A | 0 | 0 | 0 | 0 | 0 | 0 | 1 |
| ENSG00000236194 | 0 | 0 | 0 | 0 | 0 | 0 | 1 |
| ADCY1 | 0 | 0 | 0 | 0 | 0 | 0 | 1 |
| H1-3 | 0 | 0 | 0 | 0 | 0 | 0 | 1 |
| FSTL4 | 0 | 0 | 0 | 0 | 0 | 0 | 1 |
| SPATA17 | 0 | 0 | 0 | 0 | 0 | 0 | 1 |
| RPS3AP38 | 0 | 0 | 0 | 0 | 0 | 0 | 1 |
| PPP1R3E | 0 | 0 | 0 | 0 | 0 | 0 | 1 |
| HSD3B7 | 0 | 0 | 0 | 0 | 0 | 0 | 1 |
| PLIN5 | 0 | 0 | 0 | 0 | 0 | 0 | 1 |
| SPAG5 | 0 | 0 | 0 | 0 | 0 | 0 | 1 |
| MMADHCP2 | 0 | 0 | 0 | 0 | 0 | 0 | 1 |
| HSD17B7P2 | 0 | 0 | 0 | 0 | 0 | 0 | 1 |
| SCML1 | 0 | 0 | 0 | 0 | 0 | 0 | 1 |
| MFSD9 | 0 | 0 | 0 | 0 | 0 | 0 | 1 |
| RSPH4A | 0 | 0 | 0 | 0 | 0 | 0 | 1 |
| SOX13 | 0 | 0 | 0 | 0 | 0 | 0 | 1 |
| GPR137B | 0 | 0 | 0 | 0 | 0 | 0 | 1 |
| GTF2F1 | 0 | 0 | 0 | 0 | 0 | 0 | 1 |
| MT-ND1 | 0 | 0 | 0 | 0 | 0 | 0 | 1 |
| DSTNP2 | 0 | 0 | 0 | 0 | 0 | 0 | 1 |
| BORCS6 | 0 | 0 | 0 | 0 | 0 | 0 | 1 |
| ENSG00000286994 | 0 | 0 | 0 | 0 | 0 | 0 | 1 |
| LIMD1 | 0 | 0 | 0 | 0 | 0 | 0 | 1 |
| UHRF1BP1L | 0 | 0 | 0 | 0 | 0 | 0 | 1 |
| CDS1 | 0 | 0 | 0 | 0 | 0 | 0 | 1 |
| ENSG00000256433 | 0 | 0 | 0 | 0 | 0 | 0 | 1 |
| SRGAP2C | 0 | 0 | 0 | 0 | 0 | 0 | 1 |
| LINC02018 | 0 | 0 | 0 | 0 | 0 | 0 | 1 |
| ENSG00000270558 | 0 | 0 | 0 | 0 | 0 | 0 | 1 |
| PSMD6 | 0 | 0 | 0 | 0 | 0 | 0 | 1 |
| POGLUT1 | 0 | 0 | 0 | 0 | 0 | 0 | 1 |
| LTBR | 0 | 0 | 0 | 0 | 0 | 0 | 1 |
| KLHL2P1 | 0 | 0 | 0 | 0 | 0 | 0 | 1 |
| GRPEL1 | 0 | 0 | 0 | 0 | 0 | 0 | 1 |
| DIPK1B | 0 | 0 | 0 | 0 | 0 | 0 | 1 |
| SMG1P6 | 0 | 0 | 0 | 0 | 0 | 0 | 1 |
| ARPIN | 0 | 0 | 0 | 0 | 0 | 0 | 1 |
| RPS26P18 | 0 | 0 | 0 | 0 | 0 | 0 | 1 |
| ENSG00000260920 | 0 | 0 | 0 | 0 | 0 | 0 | 1 |
| GAS8 | 0 | 0 | 0 | 0 | 0 | 0 | 1 |
| MTND5P11 | 0 | 0 | 0 | 0 | 0 | 0 | 1 |
| ENSG00000254694 | 0 | 0 | 0 | 0 | 0 | 0 | 1 |
| MRPS31 | 0 | 0 | 0 | 0 | 0 | 0 | 1 |
| DCAF13P3 | 0 | 0 | 0 | 0 | 0 | 0 | 1 |
| ENSG00000257169 | 0 | 0 | 0 | 0 | 0 | 0 | 1 |
| ENSG00000276334 | 0 | 0 | 0 | 0 | 0 | 0 | 1 |
| NADK2 | 0 | 0 | 0 | 0 | 0 | 0 | 1 |
| DHDH | 0 | 0 | 0 | 0 | 0 | 0 | 1 |
| ENSG00000218175 | 0 | 0 | 0 | 0 | 0 | 0 | 1 |
| MCTS1 | 0 | 0 | 0 | 0 | 0 | 0 | 1 |
| CLDN23 | 0 | 0 | 0 | 0 | 0 | 0 | 1 |
| ENSG00000277558 | 0 | 0 | 0 | 0 | 0 | 0 | 1 |
| SPIRE2 | 0 | 0 | 0 | 0 | 0 | 0 | 1 |
| TAMALIN | 0 | 0 | 0 | 0 | 0 | 0 | 1 |
| EYS | 0 | 0 | 0 | 0 | 0 | 0 | 1 |
| CES2 | 0 | 0 | 0 | 0 | 0 | 0 | 1 |
| SNORA73B | 0 | 0 | 0 | 0 | 0 | 0 | 1 |
| FGF14 | 0 | 0 | 0 | 0 | 0 | 0 | 1 |
| ENSG00000284946 | 0 | 0 | 0 | 0 | 0 | 0 | 1 |
| ALG3 | 0 | 0 | 0 | 0 | 0 | 0 | 1 |
| ZNF611 | 0 | 0 | 0 | 0 | 0 | 0 | 1 |
| ATP5MF | 0 | 0 | 0 | 0 | 0 | 0 | 1 |
| ARFGEF3 | 0 | 0 | 0 | 0 | 0 | 0 | 1 |
| CADM4 | 0 | 0 | 0 | 0 | 0 | 0 | 1 |
| SYNC | 0 | 0 | 0 | 0 | 0 | 0 | 1 |
| RBM33-DT | 0 | 0 | 0 | 0 | 0 | 0 | 1 |
| GPR180 | 0 | 0 | 0 | 0 | 0 | 0 | 1 |
| IGSF22 | 0 | 0 | 0 | 0 | 0 | 0 | 1 |
| KYNU | 0 | 0 | 0 | 0 | 0 | 0 | 1 |
| NEGR1 | 0 | 0 | 0 | 0 | 0 | 0 | 1 |
| GNAO1 | 0 | 0 | 0 | 0 | 0 | 0 | 1 |
| ERI3-IT1 | 0 | 0 | 0 | 0 | 0 | 0 | 1 |
| ERLIN1 | 0 | 0 | 0 | 0 | 0 | 0 | 1 |
| JDP2 | 0 | 0 | 0 | 0 | 0 | 0 | 1 |
| HRG-AS1 | 0 | 0 | 0 | 0 | 0 | 0 | 1 |
| AKR1C1 | 0 | 0 | 0 | 0 | 0 | 0 | 1 |
| CFAP54 | 0 | 0 | 0 | 0 | 0 | 0 | 1 |
| PPP1R26 | 0 | 0 | 0 | 0 | 0 | 0 | 1 |
| EPCAM | 0 | 0 | 0 | 0 | 0 | 0 | 1 |
| IQCA1 | 0 | 0 | 0 | 0 | 0 | 0 | 1 |
| SEC23A | 0 | 0 | 0 | 0 | 0 | 0 | 1 |
| IQCE | 0 | 0 | 0 | 0 | 0 | 0 | 1 |
| PGAM1 | 0 | 0 | 0 | 0 | 0 | 0 | 1 |
| NR4A1 | 0 | 0 | 0 | 0 | 0 | 0 | 1 |
| ENSG00000253586 | 0 | 0 | 0 | 0 | 0 | 0 | 1 |
| CFAP53 | 0 | 0 | 0 | 0 | 0 | 0 | 1 |
| TP53INP1 | 0 | 0 | 0 | 0 | 0 | 0 | 1 |
| RPL36AP43 | 0 | 0 | 0 | 0 | 0 | 0 | 1 |
| H3-3B | 0 | 0 | 0 | 0 | 0 | 0 | 1 |
| NNT | 0 | 0 | 0 | 0 | 0 | 0 | 1 |
| MCM2 | 0 | 0 | 0 | 0 | 0 | 0 | 1 |
| FAHD2A | 0 | 0 | 0 | 0 | 0 | 0 | 1 |
| RHOBTB1 | 0 | 0 | 0 | 0 | 0 | 0 | 1 |
| POLR1G | 0 | 0 | 0 | 0 | 0 | 0 | 1 |
| MYRIP | 0 | 0 | 0 | 0 | 0 | 0 | 1 |
| ELL2 | 0 | 0 | 0 | 0 | 0 | 0 | 1 |
| ENSG00000255031 | 0 | 0 | 0 | 0 | 0 | 0 | 1 |
| GBE1 | 0 | 0 | 0 | 0 | 0 | 0 | 1 |
| HPS6 | 0 | 0 | 0 | 0 | 0 | 0 | 1 |
| DDX55P1 | 0 | 0 | 0 | 0 | 0 | 0 | 1 |
| DENND10 | 0 | 0 | 0 | 0 | 0 | 0 | 1 |
| KCTD9P4 | 0 | 0 | 0 | 0 | 0 | 0 | 1 |
| SLC27A2 | 0 | 0 | 0 | 0 | 0 | 0 | 1 |
| DDRGK1 | 0 | 0 | 0 | 0 | 0 | 0 | 1 |
| CBR4 | 0 | 0 | 0 | 0 | 0 | 0 | 1 |
| PHYKPL | 0 | 0 | 0 | 0 | 0 | 0 | 1 |
| ENSG00000287771 | 0 | 0 | 0 | 0 | 0 | 0 | 1 |
| ADIPOR2 | 0 | 0 | 0 | 0 | 0 | 0 | 1 |
| EPHB4 | 0 | 0 | 0 | 0 | 0 | 0 | 1 |
| PDCD5 | 0 | 0 | 0 | 0 | 0 | 0 | 1 |
| FRRS1 | 0 | 0 | 0 | 0 | 0 | 0 | 1 |
| HOXB6 | 0 | 0 | 0 | 0 | 0 | 0 | 1 |
| ENSG00000279133 | 0 | 0 | 0 | 0 | 0 | 0 | 1 |
| DUS4L | 0 | 0 | 0 | 0 | 0 | 0 | 1 |
| TCAIM | 0 | 0 | 0 | 0 | 0 | 0 | 1 |
| CEMIP | 0 | 0 | 0 | 0 | 0 | 0 | 1 |
| BTBD9 | 0 | 0 | 0 | 0 | 0 | 0 | 1 |
| ENSG00000288025 | 0 | 0 | 0 | 0 | 0 | 0 | 1 |
| GCNT3 | 0 | 0 | 0 | 0 | 0 | 0 | 1 |
| ENSG00000271811 | 0 | 0 | 0 | 0 | 0 | 0 | 1 |
| CHIT1 | 0 | 0 | 0 | 0 | 0 | 0 | 1 |
| MTATP6P1 | 0 | 0 | 0 | 0 | 0 | 0 | 1 |
| PRKAG2 | 0 | 0 | 0 | 0 | 0 | 0 | 1 |
| TBC1D8 | 0 | 0 | 0 | 0 | 0 | 0 | 1 |
| NPHP1 | 0 | 0 | 0 | 0 | 0 | 0 | 1 |
| ELP2 | 0 | 0 | 0 | 0 | 0 | 0 | 1 |
| PYGL | 0 | 0 | 0 | 0 | 0 | 0 | 1 |
| RASIP1 | 0 | 0 | 0 | 0 | 0 | 0 | 1 |
| HMGCL | 0 | 0 | 0 | 0 | 0 | 0 | 1 |
| C20orf27 | 0 | 0 | 0 | 0 | 0 | 0 | 1 |
| ENSG00000232710 | 0 | 0 | 0 | 0 | 0 | 0 | 1 |
| TMEM69 | 0 | 0 | 0 | 0 | 0 | 0 | 1 |
| ENSG00000230001 | 0 | 0 | 0 | 0 | 0 | 0 | 1 |
| CRIM1 | 0 | 0 | 0 | 0 | 0 | 0 | 1 |
| LINC00513 | 0 | 0 | 0 | 0 | 0 | 0 | 1 |
| ASAH2B | 0 | 0 | 0 | 0 | 0 | 0 | 1 |
| SEC24B | 0 | 0 | 0 | 0 | 0 | 0 | 1 |
| PDE5A | 0 | 0 | 0 | 0 | 0 | 0 | 1 |
| PPIAP31 | 0 | 0 | 0 | 0 | 0 | 0 | 1 |
| AMOTL1 | 0 | 0 | 0 | 0 | 0 | 0 | 1 |
| PLPP5 | 0 | 0 | 0 | 0 | 0 | 0 | 1 |
| ENSG00000280190 | 0 | 0 | 0 | 0 | 0 | 0 | 1 |
| GEN1 | 0 | 0 | 0 | 0 | 0 | 0 | 1 |
| ZNF501 | 0 | 0 | 0 | 0 | 0 | 0 | 1 |
| PRDM16 | 0 | 0 | 0 | 0 | 0 | 0 | 1 |
| RBKS | 0 | 0 | 0 | 0 | 0 | 0 | 1 |
| PCBD1 | 0 | 0 | 0 | 0 | 0 | 0 | 1 |
| CCDC40 | 0 | 0 | 0 | 0 | 0 | 0 | 1 |
| JUNB | 0 | 0 | 0 | 0 | 0 | 0 | 1 |
| PAF1 | 0 | 0 | 0 | 0 | 0 | 0 | 1 |
| STUB1 | 0 | 0 | 0 | 0 | 0 | 0 | 1 |
| JPH1 | 0 | 0 | 0 | 0 | 0 | 0 | 1 |
| ZSWIM5 | 0 | 0 | 0 | 0 | 0 | 0 | 1 |
| XYLB | 0 | 0 | 0 | 0 | 0 | 0 | 1 |
| PXMP2 | 0 | 0 | 0 | 0 | 0 | 0 | 1 |
| NANOGP4 | 0 | 0 | 0 | 0 | 0 | 0 | 1 |
| LENG1 | 0 | 0 | 0 | 0 | 0 | 0 | 1 |
| CCDC191 | 0 | 0 | 0 | 0 | 0 | 0 | 1 |
| ENSG00000254859 | 0 | 0 | 0 | 0 | 0 | 0 | 1 |
| MT-ATP6 | 0 | 0 | 0 | 0 | 0 | 0 | 1 |
| R3HCC1 | 0 | 0 | 0 | 0 | 0 | 0 | 1 |
| ENTPD7 | 0 | 0 | 0 | 0 | 0 | 0 | 1 |
| EPHB6 | 0 | 0 | 0 | 0 | 0 | 0 | 1 |
| PRKAR1B | 0 | 0 | 0 | 0 | 0 | 0 | 1 |
| MITD1 | 0 | 0 | 0 | 0 | 0 | 0 | 1 |
| RDH5 | 0 | 0 | 0 | 0 | 0 | 0 | 1 |
| DUSP10 | 0 | 0 | 0 | 0 | 0 | 0 | 1 |
| ANGPTL4 | 0 | 0 | 0 | 0 | 0 | 0 | 1 |
| ZNF71 | 0 | 0 | 0 | 0 | 0 | 0 | 1 |
| DYNLT2 | 0 | 0 | 0 | 0 | 0 | 0 | 1 |
| MTRF1 | 0 | 0 | 0 | 0 | 0 | 0 | 1 |
| TMEM97 | 0 | 0 | 0 | 0 | 0 | 0 | 1 |
| CDIN1 | 0 | 0 | 0 | 0 | 0 | 0 | 1 |
| ENSG00000287299 | 0 | 0 | 0 | 0 | 0 | 0 | 1 |
| PPARGC1A | 0 | 0 | 0 | 0 | 0 | 0 | 1 |
| OGFOD3 | 0 | 0 | 0 | 0 | 0 | 0 | 1 |
| PPIAP72 | 0 | 0 | 0 | 0 | 0 | 0 | 1 |
| RAMP2 | 0 | 0 | 0 | 0 | 0 | 0 | 1 |
| ENSG00000265943 | 0 | 0 | 0 | 0 | 0 | 0 | 1 |
| GCAT | 0 | 0 | 0 | 0 | 0 | 0 | 1 |
| OXSM | 0 | 0 | 0 | 0 | 0 | 0 | 1 |
| ENSG00000260337 | 0 | 0 | 0 | 0 | 0 | 0 | 1 |
| FAM167A | 0 | 0 | 0 | 0 | 0 | 0 | 1 |
| CAPN10-DT | 0 | 0 | 0 | 0 | 0 | 0 | 1 |
| ERO1A | 0 | 0 | 0 | 0 | 0 | 0 | 1 |
| CRACR2B | 0 | 0 | 0 | 0 | 0 | 0 | 1 |
| ATR | 0 | 0 | 0 | 0 | 0 | 0 | 1 |
| MOCS1 | 0 | 0 | 0 | 0 | 0 | 0 | 1 |
| IQUB | 0 | 0 | 0 | 0 | 0 | 0 | 1 |
| ZNF281 | 0 | 0 | 0 | 0 | 0 | 0 | 1 |
| NIBAN2 | 0 | 0 | 0 | 0 | 0 | 0 | 1 |
| ATP1B1P1 | 0 | 0 | 0 | 0 | 0 | 0 | 1 |
| ACAT1 | 0 | 0 | 0 | 0 | 0 | 0 | 1 |
| RBM42 | 0 | 0 | 0 | 0 | 0 | 0 | 1 |
| TMEM43 | 0 | 0 | 0 | 0 | 0 | 0 | 1 |
| LINC01003 | 0 | 0 | 0 | 0 | 0 | 0 | 1 |
| CDC16 | 0 | 0 | 0 | 0 | 0 | 0 | 1 |
| LPIN1 | 0 | 0 | 0 | 0 | 0 | 0 | 1 |
| RABEPK | 0 | 0 | 0 | 0 | 0 | 0 | 1 |
| SAR1B | 0 | 0 | 0 | 0 | 0 | 0 | 1 |
| DEPDC1P2 | 0 | 0 | 0 | 0 | 0 | 0 | 1 |
| SHC3 | 0 | 0 | 0 | 0 | 0 | 0 | 1 |
| MYO9A | 0 | 0 | 0 | 0 | 0 | 0 | 1 |
| SLC66A3 | 0 | 0 | 0 | 0 | 0 | 0 | 1 |
| EFHC2 | 0 | 0 | 0 | 0 | 0 | 0 | 1 |
| JAG2 | 0 | 0 | 0 | 0 | 0 | 0 | 1 |
| MIR593 | 0 | 0 | 0 | 0 | 0 | 0 | 1 |
| ENSG00000233785 | 0 | 0 | 0 | 0 | 0 | 0 | 1 |
| HLA-E | 0 | 0 | 0 | 0 | 0 | 0 | 1 |
| EEF1A1 | 0 | 0 | 0 | 0 | 0 | 0 | 1 |
| PXK | 0 | 0 | 0 | 0 | 0 | 0 | 1 |
| SLC2A12 | 0 | 0 | 0 | 0 | 0 | 0 | 1 |
| JCHAIN | 0 | 0 | 0 | 0 | 0 | 0 | 1 |
| NRBF2 | 0 | 0 | 0 | 0 | 0 | 0 | 1 |
| WHAMMP2 | 0 | 0 | 0 | 0 | 0 | 0 | 1 |
| SRF | 0 | 0 | 0 | 0 | 0 | 0 | 1 |
| ATG2B | 0 | 0 | 0 | 0 | 0 | 0 | 1 |
| MACO1 | 0 | 0 | 0 | 0 | 0 | 0 | 1 |
| SORL1 | 0 | 0 | 0 | 0 | 0 | 0 | 1 |
| AMPH | 0 | 0 | 0 | 0 | 0 | 0 | 1 |
| MVB12B | 0 | 0 | 0 | 0 | 0 | 0 | 1 |
| NCOA2 | 0 | 0 | 0 | 0 | 0 | 0 | 1 |
| ZNF425 | 0 | 0 | 0 | 0 | 0 | 0 | 1 |
| UGDH | 0 | 0 | 0 | 0 | 0 | 0 | 1 |
| ENSG00000238035 | 0 | 0 | 0 | 0 | 0 | 0 | 1 |
| ENSG00000286508 | 0 | 0 | 0 | 0 | 0 | 0 | 1 |
| KL | 0 | 0 | 0 | 0 | 0 | 0 | 1 |
| CAPN2 | 0 | 0 | 0 | 0 | 0 | 0 | 1 |
| GPI | 0 | 0 | 0 | 0 | 0 | 0 | 1 |
| CCDC14 | 0 | 0 | 0 | 0 | 0 | 0 | 1 |
| LTA4H | 0 | 0 | 0 | 0 | 0 | 0 | 1 |
| AP4M1 | 0 | 0 | 0 | 0 | 0 | 0 | 1 |
| FNDC3A | 0 | 0 | 0 | 0 | 0 | 0 | 1 |
| ELMO3 | 0 | 0 | 0 | 0 | 0 | 0 | 1 |
| ADD2 | 0 | 0 | 0 | 0 | 0 | 0 | 1 |
| ENSG00000276672 | 0 | 0 | 0 | 0 | 0 | 0 | 1 |
| RN7SKP203 | 0 | 0 | 0 | 0 | 0 | 0 | 1 |
| CDKN2AIPNL | 0 | 0 | 0 | 0 | 0 | 0 | 1 |
| NNMT | 0 | 0 | 0 | 0 | 0 | 0 | 1 |
| TMEM92-AS1 | 0 | 0 | 0 | 0 | 0 | 0 | 1 |
| ENSG00000273295 | 0 | 0 | 0 | 0 | 0 | 0 | 1 |
| ZNF366 | 0 | 0 | 0 | 0 | 0 | 0 | 1 |
| HSD17B12 | 0 | 0 | 0 | 0 | 0 | 0 | 1 |
| LINC01229 | 0 | 0 | 0 | 0 | 0 | 0 | 1 |
| GLUD2 | 0 | 0 | 0 | 0 | 0 | 0 | 1 |
| TRPT1 | 0 | 0 | 0 | 0 | 0 | 0 | 1 |
| COL4A5 | 0 | 0 | 0 | 0 | 0 | 0 | 1 |
| LNCSRLR | 0 | 0 | 0 | 0 | 0 | 0 | 1 |
| CLTB | 0 | 0 | 0 | 0 | 0 | 0 | 1 |
| BMP1 | 0 | 0 | 0 | 0 | 0 | 0 | 1 |
| ENSG00000238231 | 0 | 0 | 0 | 0 | 0 | 0 | 1 |
| CPNE8 | 0 | 0 | 0 | 0 | 0 | 0 | 1 |
| NGLY1 | 0 | 0 | 0 | 0 | 0 | 0 | 1 |
| ENSG00000243302 | 0 | 0 | 0 | 0 | 0 | 0 | 1 |
| ENSG00000279598 | 0 | 0 | 0 | 0 | 0 | 0 | 1 |
| LINC00640 | 0 | 0 | 0 | 0 | 0 | 0 | 1 |
| SLC26A6 | 0 | 0 | 0 | 0 | 0 | 0 | 1 |
| USF3 | 0 | 0 | 0 | 0 | 0 | 0 | 1 |
| ENSG00000270277 | 0 | 0 | 0 | 0 | 0 | 0 | 1 |
| SPEF2 | 0 | 0 | 0 | 0 | 0 | 0 | 1 |
| TINAGL1 | 0 | 0 | 0 | 0 | 0 | 0 | 1 |
| ENSG00000266378 | 0 | 0 | 0 | 0 | 0 | 0 | 1 |
| ATG4A | 0 | 0 | 0 | 0 | 0 | 0 | 1 |
| KCTD6 | 0 | 0 | 0 | 0 | 0 | 0 | 1 |
| PAQR8 | 0 | 0 | 0 | 0 | 0 | 0 | 1 |
| ENSG00000228886 | 0 | 0 | 0 | 0 | 0 | 0 | 1 |
| NPR1 | 0 | 0 | 0 | 0 | 0 | 0 | 1 |
| PHYH | 0 | 0 | 0 | 0 | 0 | 0 | 1 |
| ENSG00000274364 | 0 | 0 | 0 | 0 | 0 | 0 | 1 |
| GFM2 | 0 | 0 | 0 | 0 | 0 | 0 | 1 |
| ISYNA1 | 0 | 0 | 0 | 0 | 0 | 0 | 1 |
| TMEM107 | 0 | 0 | 0 | 0 | 0 | 0 | 1 |
| SLC2A4 | 0 | 0 | 0 | 0 | 0 | 0 | 1 |
| EEF1A1P8 | 0 | 0 | 0 | 0 | 0 | 0 | 1 |
| TMBIM6 | 0 | 0 | 0 | 0 | 0 | 0 | 1 |
| CCDC97 | 0 | 0 | 0 | 0 | 0 | 0 | 1 |
| H1-4 | 0 | 0 | 0 | 0 | 0 | 0 | 1 |
| NDN | 0 | 0 | 0 | 0 | 0 | 0 | 1 |
| TPM3 | 0 | 0 | 0 | 0 | 0 | 0 | 1 |
| BTC | 0 | 0 | 0 | 0 | 0 | 0 | 1 |
| SLCO5A1 | 0 | 0 | 0 | 0 | 0 | 0 | 1 |
| ENSG00000275632 | 0 | 0 | 0 | 0 | 0 | 0 | 1 |
| EEF1AKMT4 | 0 | 0 | 0 | 0 | 0 | 0 | 1 |
| SCLY | 0 | 0 | 0 | 0 | 0 | 0 | 1 |
| EPPK1 | 0 | 0 | 0 | 0 | 0 | 0 | 1 |
| ATRNL1 | 0 | 0 | 0 | 0 | 0 | 0 | 1 |
| PPP1R12C | 0 | 0 | 0 | 0 | 0 | 0 | 1 |
| LINC01176 | 0 | 0 | 0 | 0 | 0 | 0 | 1 |
| ENSG00000254536 | 0 | 0 | 0 | 0 | 0 | 0 | 1 |
| BMP2 | 0 | 0 | 0 | 0 | 0 | 0 | 1 |
| DDAH2 | 0 | 0 | 0 | 0 | 0 | 0 | 1 |
| CCDC34 | 0 | 0 | 0 | 0 | 0 | 0 | 1 |
| ANKZF1 | 0 | 0 | 0 | 0 | 0 | 0 | 1 |
| SLC15A4 | 0 | 0 | 0 | 0 | 0 | 0 | 1 |
| CMTM4 | 0 | 0 | 0 | 0 | 0 | 0 | 1 |
| ENSG00000258559 | 0 | 0 | 0 | 0 | 0 | 0 | 1 |
| FGF1 | 0 | 0 | 0 | 0 | 0 | 0 | 1 |
| CKMT2 | 0 | 0 | 0 | 0 | 0 | 0 | 1 |
| RN7SL566P | 0 | 0 | 0 | 0 | 0 | 0 | 1 |
| SLC47A1 | 0 | 0 | 0 | 0 | 0 | 0 | 1 |
| MBD4 | 0 | 0 | 0 | 0 | 0 | 0 | 1 |
| ENSG00000288542 | 0 | 0 | 0 | 0 | 0 | 0 | 1 |
| SUSD3 | 0 | 0 | 0 | 0 | 0 | 0 | 1 |
| ENSG00000288559 | 0 | 0 | 0 | 0 | 0 | 0 | 1 |
| PPP1R9B | 0 | 0 | 0 | 0 | 0 | 0 | 1 |
| TUSC3 | 0 | 0 | 0 | 0 | 0 | 0 | 1 |
| PKN1 | 0 | 0 | 0 | 0 | 0 | 0 | 1 |
| MYH11 | 0 | 0 | 0 | 0 | 0 | 0 | 1 |
| ENPEP | 0 | 0 | 0 | 0 | 0 | 0 | 1 |
| BRCC3P1 | 0 | 0 | 0 | 0 | 0 | 0 | 1 |
| RANBP1 | 0 | 0 | 0 | 0 | 0 | 0 | 1 |
| C1orf21 | 0 | 0 | 0 | 0 | 0 | 0 | 1 |
| TTLL7-IT1 | 0 | 0 | 0 | 0 | 0 | 0 | 1 |
| LIN7B | 0 | 0 | 0 | 0 | 0 | 0 | 1 |
| UNC119 | 0 | 0 | 0 | 0 | 0 | 0 | 1 |
| MAPK8IP1 | 0 | 0 | 0 | 0 | 0 | 0 | 1 |
| PACSIN3 | 0 | 0 | 0 | 0 | 0 | 0 | 1 |
| IGFBP6 | 0 | 0 | 0 | 0 | 0 | 0 | 1 |
| RFFL | 0 | 0 | 0 | 0 | 0 | 0 | 1 |
| ENSG00000287978 | 0 | 0 | 0 | 0 | 0 | 0 | 1 |
| NDUFV2P1 | 0 | 0 | 0 | 0 | 0 | 0 | 1 |
| MT-ND5 | 0 | 0 | 0 | 0 | 0 | 0 | 1 |
| PGM5 | 0 | 0 | 0 | 0 | 0 | 0 | 1 |
| ENSG00000279637 | 0 | 0 | 0 | 0 | 0 | 0 | 1 |
| LINC00957 | 0 | 0 | 0 | 0 | 0 | 0 | 1 |
| TSPAN9-IT1 | 0 | 0 | 0 | 0 | 0 | 0 | 1 |
| MRPL35 | 0 | 0 | 0 | 0 | 0 | 0 | 1 |
| HMGB2 | 0 | 0 | 0 | 0 | 0 | 0 | 1 |
| DPYSL2 | 0 | 0 | 0 | 0 | 0 | 0 | 1 |
| MTFR1 | 0 | 0 | 0 | 0 | 0 | 0 | 1 |
| BDH1 | 0 | 0 | 0 | 0 | 0 | 0 | 1 |
| TMEM232 | 0 | 0 | 0 | 0 | 0 | 0 | 1 |
| ICAM4 | 0 | 0 | 0 | 0 | 0 | 0 | 1 |
| SNORA33 | 0 | 0 | 0 | 0 | 0 | 0 | 1 |
| TTLL7 | 0 | 0 | 0 | 0 | 0 | 0 | 1 |
| RCAN3 | 0 | 0 | 0 | 0 | 0 | 0 | 1 |
| DRAP1 | 0 | 0 | 0 | 0 | 0 | 0 | 1 |
| CDC37 | 0 | 0 | 0 | 0 | 0 | 0 | 1 |
| C9orf43 | 0 | 0 | 0 | 0 | 0 | 0 | 1 |
| MED9 | 0 | 0 | 0 | 0 | 0 | 0 | 1 |
| MIR4668 | 0 | 0 | 0 | 0 | 0 | 0 | 1 |
| HEBP2 | 0 | 0 | 0 | 0 | 0 | 0 | 1 |
| RPL17P50 | 0 | 0 | 0 | 0 | 0 | 0 | 1 |
| VMO1 | 0 | 0 | 0 | 0 | 0 | 0 | 1 |
| SLC25A30 | 0 | 0 | 0 | 0 | 0 | 0 | 1 |
| CALM3 | 0 | 0 | 0 | 0 | 0 | 0 | 1 |
| ENSG00000254826 | 0 | 0 | 0 | 0 | 0 | 0 | 1 |
| ENSG00000264112 | 0 | 0 | 0 | 0 | 0 | 0 | 1 |
| ZFP28 | 0 | 0 | 0 | 0 | 0 | 0 | 1 |
| DLG5 | 0 | 0 | 0 | 0 | 0 | 0 | 1 |
| ZNF43 | 0 | 0 | 0 | 0 | 0 | 0 | 1 |
| S100A14 | 0 | 0 | 0 | 0 | 0 | 0 | 1 |
| EGFL8 | 0 | 0 | 0 | 0 | 0 | 0 | 1 |
| AGFG2 | 0 | 0 | 0 | 0 | 0 | 0 | 1 |
| MYO1E | 0 | 0 | 0 | 0 | 0 | 0 | 1 |
| ENSG00000261770 | 0 | 0 | 0 | 0 | 0 | 0 | 1 |
| SLC39A4 | 0 | 0 | 0 | 0 | 0 | 0 | 1 |
| ENSG00000287787 | 0 | 0 | 0 | 0 | 0 | 0 | 1 |
| GABARAP | 0 | 0 | 0 | 0 | 0 | 0 | 1 |
| IGLV1-40 | 0 | 0 | 0 | 0 | 0 | 0 | 1 |
| WDFY1 | 0 | 0 | 0 | 0 | 0 | 0 | 1 |
| NPY1R | 0 | 0 | 0 | 0 | 0 | 0 | 1 |
| PCID2 | 0 | 0 | 0 | 0 | 0 | 0 | 1 |
| HYAL2 | 0 | 0 | 0 | 0 | 0 | 0 | 1 |
| CFAP119 | 0 | 0 | 0 | 0 | 0 | 0 | 1 |
| TMEM204 | 0 | 0 | 0 | 0 | 0 | 0 | 1 |
| GDF7 | 0 | 0 | 0 | 0 | 0 | 0 | 1 |
| SPAAR | 0 | 0 | 0 | 0 | 0 | 0 | 1 |
| ENSG00000259810 | 0 | 0 | 0 | 0 | 0 | 0 | 1 |
| SERTAD3 | 0 | 0 | 0 | 0 | 0 | 0 | 1 |
| RN7SL1 | 0 | 0 | 0 | 0 | 0 | 0 | 1 |
| RABEP2 | 0 | 0 | 0 | 0 | 0 | 0 | 1 |
| ZNF414 | 0 | 0 | 0 | 0 | 0 | 0 | 1 |
| PCDHB10 | 0 | 0 | 0 | 0 | 0 | 0 | 1 |
| ACP2 | 0 | 0 | 0 | 0 | 0 | 0 | 1 |
| SNRNP48 | 0 | 0 | 0 | 0 | 0 | 0 | 1 |
| RPL7AP6 | 0 | 0 | 0 | 0 | 0 | 0 | 1 |
| TOM1L1 | 0 | 0 | 0 | 0 | 0 | 0 | 1 |
| ZCCHC9 | 0 | 0 | 0 | 0 | 0 | 0 | 1 |
| NAMPTP1 | 0 | 0 | 0 | 0 | 0 | 0 | 1 |
| PANK3 | 0 | 0 | 0 | 0 | 0 | 0 | 1 |
| XPOT | 0 | 0 | 0 | 0 | 0 | 0 | 1 |
| SPEN-AS1 | 0 | 0 | 0 | 0 | 0 | 0 | 1 |
| TSNAXIP1 | 0 | 0 | 0 | 0 | 0 | 0 | 1 |
| LINC02943 | 0 | 0 | 0 | 0 | 0 | 0 | 1 |
| ENSG00000263731 | 0 | 0 | 0 | 0 | 0 | 0 | 1 |
| TLCD4 | 0 | 0 | 0 | 0 | 0 | 0 | 1 |
| TBX18 | 0 | 0 | 0 | 0 | 0 | 0 | 1 |
| PLAC8 | 0 | 0 | 0 | 0 | 0 | 0 | 1 |
| ST8SIA6 | 0 | 0 | 0 | 0 | 0 | 0 | 1 |
| VEPH1 | 0 | 0 | 0 | 0 | 0 | 0 | 1 |
| MRPS6 | 0 | 0 | 0 | 0 | 0 | 0 | 1 |
| RNU6-807P | 0 | 0 | 0 | 0 | 0 | 0 | 1 |
| UBOX5 | 0 | 0 | 0 | 0 | 0 | 0 | 1 |
| RFTN2 | 0 | 0 | 0 | 0 | 0 | 0 | 1 |
| TMLHE | 0 | 0 | 0 | 0 | 0 | 0 | 1 |
| SLC9A3R1 | 0 | 0 | 0 | 0 | 0 | 0 | 1 |
| WTAP | 0 | 0 | 0 | 0 | 0 | 0 | 1 |
| ENSG00000267504 | 0 | 0 | 0 | 0 | 0 | 0 | 1 |
| ERRFI1 | 0 | 0 | 0 | 0 | 0 | 0 | 1 |
| PUS10 | 0 | 0 | 0 | 0 | 0 | 0 | 1 |
| MPZ | 0 | 0 | 0 | 0 | 0 | 0 | 1 |
| HSD11B1L | 0 | 0 | 0 | 0 | 0 | 0 | 1 |
| DIPK2B | 0 | 0 | 0 | 0 | 0 | 0 | 1 |
| ZNF423 | 0 | 0 | 0 | 0 | 0 | 0 | 1 |
| PAQR5 | 0 | 0 | 0 | 0 | 0 | 0 | 1 |
| RGN | 0 | 0 | 0 | 0 | 0 | 0 | 1 |
| CPNE7 | 0 | 0 | 0 | 0 | 0 | 0 | 1 |
| KRTCAP2 | 0 | 0 | 0 | 0 | 0 | 0 | 1 |
| CERS2 | 0 | 0 | 0 | 0 | 0 | 0 | 1 |
| PEMT | 0 | 0 | 0 | 0 | 0 | 0 | 1 |
| LAMB2 | 0 | 0 | 0 | 0 | 0 | 0 | 1 |
| PREX2 | 0 | 0 | 0 | 0 | 0 | 0 | 1 |
| PAFAH1B3 | 0 | 0 | 0 | 0 | 0 | 0 | 1 |
| SLC44A2 | 0 | 0 | 0 | 0 | 0 | 0 | 1 |
| WWTR1-IT1 | 0 | 0 | 0 | 0 | 0 | 0 | 1 |
| UBE2FP1 | 0 | 0 | 0 | 0 | 0 | 0 | 1 |
| KCTD12 | 0 | 0 | 0 | 0 | 0 | 0 | 1 |
| CDKN2B | 0 | 0 | 0 | 0 | 0 | 0 | 1 |
| ENSG00000260948 | 0 | 0 | 0 | 0 | 0 | 0 | 1 |
| MIR27B | 0 | 0 | 0 | 0 | 0 | 0 | 1 |
| SLC31A1 | 0 | 0 | 0 | 0 | 0 | 0 | 1 |
| SLC1A2 | 0 | 0 | 0 | 0 | 0 | 0 | 1 |
| NAGS | 0 | 0 | 0 | 0 | 0 | 0 | 1 |
| NMT2 | 0 | 0 | 0 | 0 | 0 | 0 | 1 |
| IRF2BP1 | 0 | 0 | 0 | 0 | 0 | 0 | 1 |
| CADM1 | 0 | 0 | 0 | 0 | 0 | 0 | 1 |
| CCDC51 | 0 | 0 | 0 | 0 | 0 | 0 | 1 |
| CLEC14A | 0 | 0 | 0 | 0 | 0 | 0 | 1 |
| HSPD1P11 | 0 | 0 | 0 | 0 | 0 | 0 | 1 |
| TFB1M | 0 | 0 | 0 | 0 | 0 | 0 | 1 |
| MFAP4 | 0 | 0 | 0 | 0 | 0 | 0 | 1 |
| NPIPA1 | 0 | 0 | 0 | 0 | 0 | 0 | 1 |
| RERG | 0 | 0 | 0 | 0 | 0 | 0 | 1 |
| DPT | 0 | 0 | 0 | 0 | 0 | 0 | 1 |
| IL1RL1 | 0 | 0 | 0 | 0 | 0 | 0 | 1 |
| ENSG00000255142 | 0 | 0 | 0 | 0 | 0 | 0 | 1 |
| H1-5 | 0 | 0 | 0 | 0 | 0 | 0 | 1 |
| ADSS1 | 0 | 0 | 0 | 0 | 0 | 0 | 1 |
| FBXO43 | 0 | 0 | 0 | 0 | 0 | 0 | 1 |
| FLYWCH2 | 0 | 0 | 0 | 0 | 0 | 0 | 1 |
| FST | 0 | 0 | 0 | 0 | 0 | 0 | 1 |
| THRA | 0 | 0 | 0 | 0 | 0 | 0 | 1 |
| CDHR3 | 0 | 0 | 0 | 0 | 0 | 0 | 1 |
| ENSG00000254484 | 0 | 0 | 0 | 0 | 0 | 0 | 1 |
| TMSB10 | 0 | 0 | 0 | 0 | 0 | 0 | 1 |
| FKBPL | 0 | 0 | 0 | 0 | 0 | 0 | 1 |
| HLA-DRB5 | 0 | 0 | 0 | 0 | 0 | 0 | 1 |
| A1BG | 0 | 0 | 0 | 0 | 0 | 0 | 1 |
| ADCY10 | 0 | 0 | 0 | 0 | 0 | 0 | 1 |
| METAP1D | 0 | 0 | 0 | 0 | 0 | 0 | 1 |
| CEP126 | 0 | 0 | 0 | 0 | 0 | 0 | 1 |
| KLHL25 | 0 | 0 | 0 | 0 | 0 | 0 | 1 |
| RTN4RL2 | 0 | 0 | 0 | 0 | 0 | 0 | 1 |
| ENSG00000254330 | 0 | 0 | 0 | 0 | 0 | 0 | 1 |
| SEMA6A | 0 | 0 | 0 | 0 | 0 | 0 | 1 |
| PCBP2-OT1 | 0 | 0 | 0 | 0 | 0 | 0 | 1 |
| PDGFA | 0 | 0 | 0 | 0 | 0 | 0 | 1 |
| GTF2IP23 | 0 | 0 | 0 | 0 | 0 | 0 | 1 |
| MAMDC2 | 0 | 0 | 0 | 0 | 0 | 0 | 1 |
| PTPRB | 0 | 0 | 0 | 0 | 0 | 0 | 1 |
| NPM1P27 | 0 | 0 | 0 | 0 | 0 | 0 | 1 |
| SHPK | 0 | 0 | 0 | 0 | 0 | 0 | 1 |
| ENSG00000280339 | 0 | 0 | 0 | 0 | 0 | 0 | 1 |
| TRPM7 | 0 | 0 | 0 | 0 | 0 | 0 | 1 |
| ZNF316 | 0 | 0 | 0 | 0 | 0 | 0 | 1 |
| MYL9 | 0 | 0 | 0 | 0 | 0 | 0 | 1 |
| CELSR1 | 0 | 0 | 0 | 0 | 0 | 0 | 1 |
| TRABD2A | 0 | 0 | 0 | 0 | 0 | 0 | 1 |
| CBX2 | 0 | 0 | 0 | 0 | 0 | 0 | 1 |
| SCP2 | 0 | 0 | 0 | 0 | 0 | 0 | 1 |
| SNORD101 | 0 | 0 | 0 | 0 | 0 | 0 | 1 |
| ENSG00000278987 | 0 | 0 | 0 | 0 | 0 | 0 | 1 |
| HLA-DOA | 0 | 0 | 0 | 0 | 0 | 0 | 1 |
| TOM1L2 | 0 | 0 | 0 | 0 | 0 | 0 | 1 |
| MTIF2 | 0 | 0 | 0 | 0 | 0 | 0 | 1 |
| RN7SKP292 | 0 | 0 | 0 | 0 | 0 | 0 | 1 |
| RPL10P9 | 0 | 0 | 0 | 0 | 0 | 0 | 1 |
| ZNF684 | 0 | 0 | 0 | 0 | 0 | 0 | 1 |
| COQ9 | 0 | 0 | 0 | 0 | 0 | 0 | 1 |
| ENSG00000276337 | 0 | 0 | 0 | 0 | 0 | 0 | 1 |
| ENSG00000270574 | 0 | 0 | 0 | 0 | 0 | 0 | 1 |
| BICDL2 | 0 | 0 | 0 | 0 | 0 | 0 | 1 |
| SHE | 0 | 0 | 0 | 0 | 0 | 0 | 1 |
| ENSG00000285517 | 0 | 0 | 0 | 0 | 0 | 0 | 1 |
| DERL2 | 0 | 0 | 0 | 0 | 0 | 0 | 1 |
| SUMO4 | 0 | 0 | 0 | 0 | 0 | 0 | 1 |
| APOL2 | 0 | 0 | 0 | 0 | 0 | 0 | 1 |
| GAS5-AS1 | 0 | 0 | 0 | 0 | 0 | 0 | 1 |
| ENSG00000234584 | 0 | 0 | 0 | 0 | 0 | 0 | 1 |
| ZNF101 | 0 | 0 | 0 | 0 | 0 | 0 | 1 |
| SCARNA7 | 0 | 0 | 0 | 0 | 0 | 0 | 1 |
| CLDN3 | 0 | 0 | 0 | 0 | 0 | 0 | 1 |
| MT2A | 0 | 0 | 0 | 0 | 0 | 0 | 1 |
| ARHGAP10 | 0 | 0 | 0 | 0 | 0 | 0 | 1 |
| ITPR2 | 0 | 0 | 0 | 0 | 0 | 0 | 1 |
| IBTK | 0 | 0 | 0 | 0 | 0 | 0 | 1 |
| AATK | 0 | 0 | 0 | 0 | 0 | 0 | 1 |
| ENSG00000261786 | 0 | 0 | 0 | 0 | 0 | 0 | 1 |
| PSIP1 | 0 | 0 | 0 | 0 | 0 | 0 | 1 |
| F2RL2 | 0 | 0 | 0 | 0 | 0 | 0 | 1 |
| WFDC1 | 0 | 0 | 0 | 0 | 0 | 0 | 1 |
| TANK | 0 | 0 | 0 | 0 | 0 | 0 | 1 |
| UNC45A | 0 | 0 | 0 | 0 | 0 | 0 | 1 |
| AAMP | 0 | 0 | 0 | 0 | 0 | 0 | 1 |
| B3GALT4 | 0 | 0 | 0 | 0 | 0 | 0 | 1 |
| ENSG00000278600 | 0 | 0 | 0 | 0 | 0 | 0 | 1 |
| SLC35D2 | 0 | 0 | 0 | 0 | 0 | 0 | 1 |
| TMEM33 | 0 | 0 | 0 | 0 | 0 | 0 | 1 |
| SFTPD | 0 | 0 | 0 | 0 | 0 | 0 | 1 |
| SWAP70 | 0 | 0 | 0 | 0 | 0 | 0 | 1 |
| ENSG00000280376 | 0 | 0 | 0 | 0 | 0 | 0 | 1 |
| MRPL23 | 0 | 0 | 0 | 0 | 0 | 0 | 1 |
| CHST12 | 0 | 0 | 0 | 0 | 0 | 0 | 1 |
| GPHN | 0 | 0 | 0 | 0 | 0 | 0 | 1 |
| COX5A | 0 | 0 | 0 | 0 | 0 | 0 | 1 |
| BCL6 | 0 | 0 | 0 | 0 | 0 | 0 | 1 |
| SLC6A13 | 0 | 0 | 0 | 0 | 0 | 0 | 1 |
| SFXN5 | 0 | 0 | 0 | 0 | 0 | 0 | 1 |
| ZBTB7C | 0 | 0 | 0 | 0 | 0 | 0 | 1 |
| SERPINI1 | 0 | 0 | 0 | 0 | 0 | 0 | 1 |
| TSPY26P | 0 | 0 | 0 | 0 | 0 | 0 | 1 |
| CSRP1-AS1 | 0 | 0 | 0 | 0 | 0 | 0 | 1 |
| NUDT13 | 0 | 0 | 0 | 0 | 0 | 0 | 1 |
| COLEC10 | 0 | 0 | 0 | 0 | 0 | 0 | 1 |
| ENSG00000273284 | 0 | 0 | 0 | 0 | 0 | 0 | 1 |
| OSCP1 | 0 | 0 | 0 | 0 | 0 | 0 | 1 |
| TMEM115 | 0 | 0 | 0 | 0 | 0 | 0 | 1 |
| ARHGEF10L | 0 | 0 | 0 | 0 | 0 | 0 | 1 |
| CARD8-AS1 | 0 | 0 | 0 | 0 | 0 | 0 | 1 |
| LINC01750 | 0 | 0 | 0 | 0 | 0 | 0 | 1 |
| PDZD8 | 0 | 0 | 0 | 0 | 0 | 0 | 1 |
| PCDHB11 | 0 | 0 | 0 | 0 | 0 | 0 | 1 |
| PRKAB2 | 0 | 0 | 0 | 0 | 0 | 0 | 1 |
| CYTH3 | 0 | 0 | 0 | 0 | 0 | 0 | 1 |
| H2AC20 | 0 | 0 | 0 | 0 | 0 | 0 | 1 |
| CCDC61 | 0 | 0 | 0 | 0 | 0 | 0 | 1 |
| ENSG00000256325 | 0 | 0 | 0 | 0 | 0 | 0 | 1 |
| TMEM243 | 0 | 0 | 0 | 0 | 0 | 0 | 1 |
| RN7SL381P | 0 | 0 | 0 | 0 | 0 | 0 | 1 |
| NOS1AP | 0 | 0 | 0 | 0 | 0 | 0 | 1 |
| PCBP2P2 | 0 | 0 | 0 | 0 | 0 | 0 | 1 |
| NR1H3 | 0 | 0 | 0 | 0 | 0 | 0 | 1 |
| CASP4 | 0 | 0 | 0 | 0 | 0 | 0 | 1 |
| MUC20P1 | 0 | 0 | 0 | 0 | 0 | 0 | 1 |
| LINC01001 | 0 | 0 | 0 | 0 | 0 | 0 | 1 |
| TMEM230 | 0 | 0 | 0 | 0 | 0 | 0 | 1 |
| DDX54 | 0 | 0 | 0 | 0 | 0 | 0 | 1 |
| DCDC2 | 0 | 0 | 0 | 0 | 0 | 0 | 1 |
| CCNT2 | 0 | 0 | 0 | 0 | 0 | 0 | 1 |
| ENSG00000236095 | 0 | 0 | 0 | 0 | 0 | 0 | 1 |
| RPL7P49 | 0 | 0 | 0 | 0 | 0 | 0 | 1 |
| RETSAT | 0 | 0 | 0 | 0 | 0 | 0 | 1 |
| CNTN1 | 0 | 0 | 0 | 0 | 0 | 0 | 1 |
| FUZ | 0 | 0 | 0 | 0 | 0 | 0 | 1 |
| SOGA1 | 0 | 0 | 0 | 0 | 0 | 0 | 1 |
| COL8A2 | 0 | 0 | 0 | 0 | 0 | 0 | 1 |
| DCTPP1 | 0 | 0 | 0 | 0 | 0 | 0 | 1 |
| LRRC45 | 0 | 0 | 0 | 0 | 0 | 0 | 1 |
| ACADL | 0 | 0 | 0 | 0 | 0 | 0 | 1 |
| WDR83 | 0 | 0 | 0 | 0 | 0 | 0 | 1 |
| CNOT2 | 0 | 0 | 0 | 0 | 0 | 0 | 1 |
| ADGRG2 | 0 | 0 | 0 | 0 | 0 | 0 | 1 |
| RN7SKP78 | 0 | 0 | 0 | 0 | 0 | 0 | 1 |
| SNORA54 | 0 | 0 | 0 | 0 | 0 | 0 | 1 |
| ENSG00000260144 | 0 | 0 | 0 | 0 | 0 | 0 | 1 |
| UCHL3 | 0 | 0 | 0 | 0 | 0 | 0 | 1 |
| CBS | 0 | 0 | 0 | 0 | 0 | 0 | 1 |
| PRKCZ | 0 | 0 | 0 | 0 | 0 | 0 | 1 |
| DAAM2-AS1 | 0 | 0 | 0 | 0 | 0 | 0 | 1 |
| ABHD3 | 0 | 0 | 0 | 0 | 0 | 0 | 1 |
| ZFP91 | 0 | 0 | 0 | 0 | 0 | 0 | 1 |
| ARMC2 | 0 | 0 | 0 | 0 | 0 | 0 | 1 |
| ENSG00000225026 | 0 | 0 | 0 | 0 | 0 | 0 | 1 |
| ENSG00000286406 | 0 | 0 | 0 | 0 | 0 | 0 | 1 |
| NPTX2 | 0 | 0 | 0 | 0 | 0 | 0 | 1 |
| ADAM20 | 0 | 0 | 0 | 0 | 0 | 0 | 1 |
| RN7SKP296 | 0 | 0 | 0 | 0 | 0 | 0 | 1 |
| ENSG00000277246 | 0 | 0 | 0 | 0 | 0 | 0 | 1 |
| UGP2 | 0 | 0 | 0 | 0 | 0 | 0 | 1 |
| RBMXP2 | 0 | 0 | 0 | 0 | 0 | 0 | 1 |
| GUF1 | 0 | 0 | 0 | 0 | 0 | 0 | 1 |
| ZNF66 | 0 | 0 | 0 | 0 | 0 | 0 | 1 |
| CROCCP3 | 0 | 0 | 0 | 0 | 0 | 0 | 1 |
| AIG1 | 0 | 0 | 0 | 0 | 0 | 0 | 1 |
| ATAT1 | 0 | 0 | 0 | 0 | 0 | 0 | 1 |
| NDUFB7 | 0 | 0 | 0 | 0 | 0 | 0 | 1 |
| AKR1C3 | 0 | 0 | 0 | 0 | 0 | 0 | 1 |
| CYBRD1 | 0 | 0 | 0 | 0 | 0 | 0 | 1 |
| NUDT18 | 0 | 0 | 0 | 0 | 0 | 0 | 1 |
| NME5 | 0 | 0 | 0 | 0 | 0 | 0 | 1 |
| CABYR | 0 | 0 | 0 | 0 | 0 | 0 | 1 |
| CCDC81 | 0 | 0 | 0 | 0 | 0 | 0 | 1 |
| SNORA80A | 0 | 0 | 0 | 0 | 0 | 0 | 1 |
| LINC02883 | 0 | 0 | 0 | 0 | 0 | 0 | 1 |
| ENSG00000262140 | 0 | 0 | 0 | 0 | 0 | 0 | 1 |
| NUGGC | 0 | 0 | 0 | 0 | 0 | 0 | 1 |
| INTU | 0 | 0 | 0 | 0 | 0 | 0 | 1 |
| ENSG00000267838 | 0 | 0 | 0 | 0 | 0 | 0 | 1 |
| TRIOBP | 0 | 0 | 0 | 0 | 0 | 0 | 1 |
| DIPK1A | 0 | 0 | 0 | 0 | 0 | 0 | 1 |
| TMEM200C | 0 | 0 | 0 | 0 | 0 | 0 | 1 |
| MRPL46 | 0 | 0 | 0 | 0 | 0 | 0 | 1 |
| HHIP | 0 | 0 | 0 | 0 | 0 | 0 | 1 |
| GPR89A | 0 | 0 | 0 | 0 | 0 | 0 | 1 |
| DNAH7 | 0 | 0 | 0 | 0 | 0 | 0 | 1 |
| CACHD1 | 0 | 0 | 0 | 0 | 0 | 0 | 1 |
| ENSG00000280332 | 0 | 0 | 0 | 0 | 0 | 0 | 1 |
| ENSG00000282936 | 0 | 0 | 0 | 0 | 0 | 0 | 1 |
| GUSBP18 | 0 | 0 | 0 | 0 | 0 | 0 | 1 |
| CHAF1A | 0 | 0 | 0 | 0 | 0 | 0 | 1 |
| KCNMA1 | 0 | 0 | 0 | 0 | 0 | 0 | 1 |
| CFL1P5 | 0 | 0 | 0 | 0 | 0 | 0 | 1 |
| SMPD1 | 0 | 0 | 0 | 0 | 0 | 0 | 1 |
| CLSTN1 | 0 | 0 | 0 | 0 | 0 | 0 | 1 |
| ARMC7 | 0 | 0 | 0 | 0 | 0 | 0 | 1 |
| LONP2 | 0 | 0 | 0 | 0 | 0 | 0 | 1 |
| PRDM5 | 0 | 0 | 0 | 0 | 0 | 0 | 1 |
| RAB23 | 0 | 0 | 0 | 0 | 0 | 0 | 1 |
| CASKIN2 | 0 | 0 | 0 | 0 | 0 | 0 | 1 |
| CENPBD2P | 0 | 0 | 0 | 0 | 0 | 0 | 1 |
| SANBR | 0 | 0 | 0 | 0 | 0 | 0 | 1 |
| PPP1R3B | 0 | 0 | 0 | 0 | 0 | 0 | 1 |
| SERPINE2 | 0 | 0 | 0 | 0 | 0 | 0 | 1 |
| ETV6 | 0 | 0 | 0 | 0 | 0 | 0 | 1 |
| SNHG4 | 0 | 0 | 0 | 0 | 0 | 0 | 1 |
| ENSG00000282221 | 0 | 0 | 0 | 0 | 0 | 0 | 1 |
| RAB26 | 0 | 0 | 0 | 0 | 0 | 0 | 1 |
| ZNF282 | 0 | 0 | 0 | 0 | 0 | 0 | 1 |
| SDSL | 0 | 0 | 0 | 0 | 0 | 0 | 1 |
| C5orf22 | 0 | 0 | 0 | 0 | 0 | 0 | 1 |
| ZBTB42 | 0 | 0 | 0 | 0 | 0 | 0 | 1 |
| ENSG00000237927 | 0 | 0 | 0 | 0 | 0 | 0 | 1 |
| CDR2L | 0 | 0 | 0 | 0 | 0 | 0 | 1 |
| PROS1 | 0 | 0 | 0 | 0 | 0 | 0 | 1 |
| ATP5F1B | 0 | 0 | 0 | 0 | 0 | 0 | 1 |
| RPS3P2 | 0 | 0 | 0 | 0 | 0 | 0 | 1 |
| SRSF12 | 0 | 0 | 0 | 0 | 0 | 0 | 1 |
| ENSG00000260196 | 0 | 0 | 0 | 0 | 0 | 0 | 1 |
| RNF103 | 0 | 0 | 0 | 0 | 0 | 0 | 1 |
| C17orf100 | 0 | 0 | 0 | 0 | 0 | 0 | 1 |
| LINC00528 | 0 | 0 | 0 | 0 | 0 | 0 | 1 |
| ENSG00000261072 | 0 | 0 | 0 | 0 | 0 | 0 | 1 |
| ACOX2 | 0 | 0 | 0 | 0 | 0 | 0 | 1 |
| HBEGF | 0 | 0 | 0 | 0 | 0 | 0 | 1 |
| LINC01409 | 0 | 0 | 0 | 0 | 0 | 0 | 1 |
| MTCO1P11 | 0 | 0 | 0 | 0 | 0 | 0 | 1 |
| EHD2 | 0 | 0 | 0 | 0 | 0 | 0 | 1 |
| TRAPPC6A | 0 | 0 | 0 | 0 | 0 | 0 | 1 |
| USP35 | 0 | 0 | 0 | 0 | 0 | 0 | 1 |
| DPCD | 0 | 0 | 0 | 0 | 0 | 0 | 1 |
| ENSG00000232546 | 0 | 0 | 0 | 0 | 0 | 0 | 1 |
| ENSG00000258539 | 0 | 0 | 0 | 0 | 0 | 0 | 1 |
| RBPMS-AS1 | 0 | 0 | 0 | 0 | 0 | 0 | 1 |
| CHMP4BP1 | 0 | 0 | 0 | 0 | 0 | 0 | 1 |
| TSNARE1 | 0 | 0 | 0 | 0 | 0 | 0 | 1 |
| ADRA1A | 0 | 0 | 0 | 0 | 0 | 0 | 1 |
| C1orf226 | 0 | 0 | 0 | 0 | 0 | 0 | 1 |
| PPT2 | 0 | 0 | 0 | 0 | 0 | 0 | 1 |
| ZNF28 | 0 | 0 | 0 | 0 | 0 | 0 | 1 |
| ENSG00000250771 | 0 | 0 | 0 | 0 | 0 | 0 | 1 |
| CLGN | 0 | 0 | 0 | 0 | 0 | 0 | 1 |
| NANOGP5 | 0 | 0 | 0 | 0 | 0 | 0 | 1 |
| SPSB2 | 0 | 0 | 0 | 0 | 0 | 0 | 1 |
| WNT3 | 0 | 0 | 0 | 0 | 0 | 0 | 1 |
| LCN12 | 0 | 0 | 0 | 0 | 0 | 0 | 1 |
| ZNRF2P1 | 0 | 0 | 0 | 0 | 0 | 0 | 1 |
| NUDT6 | 0 | 0 | 0 | 0 | 0 | 0 | 1 |
| MTFR2 | 0 | 0 | 0 | 0 | 0 | 0 | 1 |
| CASTOR2 | 0 | 0 | 0 | 0 | 0 | 0 | 1 |
| CACNA1C-IT3 | 0 | 0 | 0 | 0 | 0 | 0 | 1 |
| CHAC2 | 0 | 0 | 0 | 0 | 0 | 0 | 1 |
| STMN1 | 0 | 0 | 0 | 0 | 0 | 0 | 1 |
| CLCN5 | 0 | 0 | 0 | 0 | 0 | 0 | 1 |
| S100A10 | 0 | 0 | 0 | 0 | 0 | 0 | 1 |
| RRS1 | 0 | 0 | 0 | 0 | 0 | 0 | 1 |
| RN7SL735P | 0 | 0 | 0 | 0 | 0 | 0 | 1 |
| TJP2 | 0 | 0 | 0 | 0 | 0 | 0 | 1 |
| IDH2 | 0 | 0 | 0 | 0 | 0 | 0 | 1 |
| SEMA6D | 0 | 0 | 0 | 0 | 0 | 0 | 1 |
| ICAM2 | 0 | 0 | 0 | 0 | 0 | 0 | 1 |
| RNF157 | 0 | 0 | 0 | 0 | 0 | 0 | 1 |
| OSGIN1 | 0 | 0 | 0 | 0 | 0 | 0 | 1 |
| ZFP69B | 0 | 0 | 0 | 0 | 0 | 0 | 1 |
| IL22RA1 | 0 | 0 | 0 | 0 | 0 | 0 | 1 |
| SKI | 0 | 0 | 0 | 0 | 0 | 0 | 1 |
| GTF2IP5 | 0 | 0 | 0 | 0 | 0 | 0 | 1 |
| NPEPPSP1 | 0 | 0 | 0 | 0 | 0 | 0 | 1 |
| ENSG00000226149 | 0 | 0 | 0 | 0 | 0 | 0 | 1 |
| PTCD2 | 0 | 0 | 0 | 0 | 0 | 0 | 1 |
| KRTCAP3 | 0 | 0 | 0 | 0 | 0 | 0 | 1 |
| CCNB1IP1 | 0 | 0 | 0 | 0 | 0 | 0 | 1 |
| CEP131 | 0 | 0 | 0 | 0 | 0 | 0 | 1 |
| DNASE1L3 | 0 | 0 | 0 | 0 | 0 | 0 | 1 |
| CDO1 | 0 | 0 | 0 | 0 | 0 | 0 | 1 |
| RPS27AP12 | 0 | 0 | 0 | 0 | 0 | 0 | 1 |
| GCSH | 0 | 0 | 0 | 0 | 0 | 0 | 1 |
| CCDC15 | 0 | 0 | 0 | 0 | 0 | 0 | 1 |
| VAMP5 | 0 | 0 | 0 | 0 | 0 | 0 | 1 |
| ENSG00000248840 | 0 | 0 | 0 | 0 | 0 | 0 | 1 |
| C3orf86 | 0 | 0 | 0 | 0 | 0 | 0 | 1 |
| LDHD | 0 | 0 | 0 | 0 | 0 | 0 | 1 |
| NDNF | 0 | 0 | 0 | 0 | 0 | 0 | 1 |
| NAV2-AS6 | 0 | 0 | 0 | 0 | 0 | 0 | 1 |
| PLPP6 | 0 | 0 | 0 | 0 | 0 | 0 | 1 |
| CDKL5 | 0 | 0 | 0 | 0 | 0 | 0 | 1 |
| EFNB1 | 0 | 0 | 0 | 0 | 0 | 0 | 1 |
| BPHL | 0 | 0 | 0 | 0 | 0 | 0 | 1 |
| TAS2R5 | 0 | 0 | 0 | 0 | 0 | 0 | 1 |
| PRAG1 | 0 | 0 | 0 | 0 | 0 | 0 | 1 |
| TMEM182 | 0 | 0 | 0 | 0 | 0 | 0 | 1 |
| PIWIL2 | 0 | 0 | 0 | 0 | 0 | 0 | 1 |
| SPTLC1P1 | 0 | 0 | 0 | 0 | 0 | 0 | 1 |
| MT1A | 0 | 0 | 0 | 0 | 0 | 0 | 1 |
| C8orf44 | 0 | 0 | 0 | 0 | 0 | 0 | 1 |
| ANKHD1 | 0 | 0 | 0 | 0 | 0 | 0 | 1 |
| COX8A | 0 | 0 | 0 | 0 | 0 | 0 | 1 |
| MVK | 0 | 0 | 0 | 0 | 0 | 0 | 1 |
| ANKRD10-IT1 | 0 | 0 | 0 | 0 | 0 | 0 | 1 |
| ENSG00000285791 | 0 | 0 | 0 | 0 | 0 | 0 | 1 |
| EPAS1 | 0 | 0 | 0 | 0 | 0 | 0 | 1 |
| HSPA8P15 | 0 | 0 | 0 | 0 | 0 | 0 | 1 |
| C1orf116 | 0 | 0 | 0 | 0 | 0 | 0 | 1 |
| MT-ND4 | 0 | 0 | 0 | 0 | 0 | 0 | 1 |
| TGFB2 | 0 | 0 | 0 | 0 | 0 | 0 | 1 |
| TNRC18P1 | 0 | 0 | 0 | 0 | 0 | 0 | 1 |
| LRP2BP-AS1 | 0 | 0 | 0 | 0 | 0 | 0 | 1 |
| SLC25A25-AS1 | 0 | 0 | 0 | 0 | 0 | 0 | 1 |
| BNIPL | 0 | 0 | 0 | 0 | 0 | 0 | 1 |
| ENSG00000234624 | 0 | 0 | 0 | 0 | 0 | 0 | 1 |
| IGFBP2 | 0 | 0 | 0 | 0 | 0 | 0 | 1 |
| RWDD4 | 0 | 0 | 0 | 0 | 0 | 0 | 1 |
| GPR89B | 0 | 0 | 0 | 0 | 0 | 0 | 1 |
| STARD9 | 0 | 0 | 0 | 0 | 0 | 0 | 1 |
| CCN2 | 0 | 0 | 0 | 0 | 0 | 0 | 1 |
| CCDC168 | 0 | 0 | 0 | 0 | 0 | 0 | 1 |
| AGL | 0 | 0 | 0 | 0 | 0 | 0 | 1 |
| ZNF212 | 0 | 0 | 0 | 0 | 0 | 0 | 1 |
| ENSG00000267682 | 0 | 0 | 0 | 0 | 0 | 0 | 1 |
| ENSG00000257298 | 0 | 0 | 0 | 0 | 0 | 0 | 1 |
| ENSG00000225339 | 0 | 0 | 0 | 0 | 0 | 0 | 1 |
| ENSG00000286977 | 0 | 0 | 0 | 0 | 0 | 0 | 1 |
| TAB1 | 0 | 0 | 0 | 0 | 0 | 0 | 1 |
| DUSP5-DT | 0 | 0 | 0 | 0 | 0 | 0 | 1 |
| RABL6 | 0 | 0 | 0 | 0 | 0 | 0 | 1 |
| NDUFB4 | 0 | 0 | 0 | 0 | 0 | 0 | 1 |
| ENO3 | 0 | 0 | 0 | 0 | 0 | 0 | 1 |
| SCAF1 | 0 | 0 | 0 | 0 | 0 | 0 | 1 |
| FAM151B-DT | 0 | 0 | 0 | 0 | 0 | 0 | 1 |
| PKD2 | 0 | 0 | 0 | 0 | 0 | 0 | 1 |
| RPL7AP45 | 0 | 0 | 0 | 0 | 0 | 0 | 1 |
| TAOK2 | 0 | 0 | 0 | 0 | 0 | 0 | 1 |
| MICAL3 | 0 | 0 | 0 | 0 | 0 | 0 | 1 |
| DOHH | 0 | 0 | 0 | 0 | 0 | 0 | 1 |
| ENSG00000269968 | 0 | 0 | 0 | 0 | 0 | 0 | 1 |
| CEP68 | 0 | 0 | 0 | 0 | 0 | 0 | 1 |
| CD9 | 0 | 0 | 0 | 0 | 0 | 0 | 1 |
| HDAC11 | 0 | 0 | 0 | 0 | 0 | 0 | 1 |
| AK8 | 0 | 0 | 0 | 0 | 0 | 0 | 1 |
| TAS2R64P | 0 | 0 | 0 | 0 | 0 | 0 | 1 |
| BCKDHB | 0 | 0 | 0 | 0 | 0 | 0 | 1 |
| VPS37A | 0 | 0 | 0 | 0 | 0 | 0 | 1 |
| RNU1-16P | 0 | 0 | 0 | 0 | 0 | 0 | 1 |
| DCUN1D4 | 0 | 0 | 0 | 0 | 0 | 0 | 1 |
| USPL1 | 0 | 0 | 0 | 0 | 0 | 0 | 1 |
| PRRG1 | 0 | 0 | 0 | 0 | 0 | 0 | 1 |
| PDCD2L | 0 | 0 | 0 | 0 | 0 | 0 | 1 |
| CD2BP2 | 0 | 0 | 0 | 0 | 0 | 0 | 1 |
| RPL19 | 0 | 0 | 0 | 0 | 0 | 0 | 1 |
| MGST2 | 0 | 0 | 0 | 0 | 0 | 0 | 1 |
| TACSTD2 | 0 | 0 | 0 | 0 | 0 | 0 | 1 |
| OSBP | 0 | 0 | 0 | 0 | 0 | 0 | 1 |
| TCEAL3 | 0 | 0 | 0 | 0 | 0 | 0 | 1 |
| ASCC1 | 0 | 0 | 0 | 0 | 0 | 0 | 1 |
| RBBP4P1 | 0 | 0 | 0 | 0 | 0 | 0 | 1 |
| ARHGAP26-IT1 | 0 | 0 | 0 | 0 | 0 | 0 | 1 |
| INCENP | 0 | 0 | 0 | 0 | 0 | 0 | 1 |
| HDC | 0 | 0 | 0 | 0 | 0 | 0 | 1 |
| MLPH | 0 | 0 | 0 | 0 | 0 | 0 | 1 |
| ENSG00000286813 | 0 | 0 | 0 | 0 | 0 | 0 | 1 |
| MIEF2 | 0 | 0 | 0 | 0 | 0 | 0 | 1 |
| LARGE2 | 0 | 0 | 0 | 0 | 0 | 0 | 1 |
| PELP1 | 0 | 0 | 0 | 0 | 0 | 0 | 1 |
| VPS26BP1 | 0 | 0 | 0 | 0 | 0 | 0 | 1 |
| SLC5A6 | 0 | 0 | 0 | 0 | 0 | 0 | 1 |
| ENSG00000280054 | 0 | 0 | 0 | 0 | 0 | 0 | 1 |
| ZNF605 | 0 | 0 | 0 | 0 | 0 | 0 | 1 |
| GRTP1 | 0 | 0 | 0 | 0 | 0 | 0 | 1 |
| ENSG00000288612 | 0 | 0 | 0 | 0 | 0 | 0 | 1 |
| ENSG00000279819 | 0 | 0 | 0 | 0 | 0 | 0 | 1 |
| KIAA1549 | 0 | 0 | 0 | 0 | 0 | 0 | 1 |
| TNFAIP8 | 0 | 0 | 0 | 0 | 0 | 0 | 1 |
| ECHDC2 | 0 | 0 | 0 | 0 | 0 | 0 | 1 |
| NTN4 | 0 | 0 | 0 | 0 | 0 | 0 | 1 |
| ENSG00000230454 | 0 | 0 | 0 | 0 | 0 | 0 | 1 |
| HERC3 | 0 | 0 | 0 | 0 | 0 | 0 | 1 |
| IGF1R | 0 | 0 | 0 | 0 | 0 | 0 | 1 |
| MIR4435-2HG | 0 | 0 | 0 | 0 | 0 | 0 | 1 |
| PLCH1 | 0 | 0 | 0 | 0 | 0 | 0 | 1 |
| LMNB2 | 0 | 0 | 0 | 0 | 0 | 0 | 1 |
| DNAH6 | 0 | 0 | 0 | 0 | 0 | 0 | 1 |
| ZNF362 | 0 | 0 | 0 | 0 | 0 | 0 | 1 |
| ZIK1 | 0 | 0 | 0 | 0 | 0 | 0 | 1 |
| STRCP1 | 0 | 0 | 0 | 0 | 0 | 0 | 1 |
| ENSG00000234589 | 0 | 0 | 0 | 0 | 0 | 0 | 1 |
| SIMC1 | 0 | 0 | 0 | 0 | 0 | 0 | 1 |
| DAP | 0 | 0 | 0 | 0 | 0 | 0 | 1 |
| ZNF775 | 0 | 0 | 0 | 0 | 0 | 0 | 1 |
| ROBO4 | 0 | 0 | 0 | 0 | 0 | 0 | 1 |
| PCCB | 0 | 0 | 0 | 0 | 0 | 0 | 1 |
| PAPPA | 0 | 0 | 0 | 0 | 0 | 0 | 1 |
| APBB1IP | 0 | 0 | 0 | 0 | 0 | 0 | 1 |
| ENSG00000255067 | 0 | 0 | 0 | 0 | 0 | 0 | 1 |
| PKP3 | 0 | 0 | 0 | 0 | 0 | 0 | 1 |
| PTPN13 | 0 | 0 | 0 | 0 | 0 | 0 | 1 |
| RPS6KA4 | 0 | 0 | 0 | 0 | 0 | 0 | 1 |
| PMEPA1 | 0 | 0 | 0 | 0 | 0 | 0 | 1 |
| RESF1 | 0 | 0 | 0 | 0 | 0 | 0 | 1 |
| C5orf15 | 0 | 0 | 0 | 0 | 0 | 0 | 1 |
| SCCPDH | 0 | 0 | 0 | 0 | 0 | 0 | 1 |
| KRI1 | 0 | 0 | 0 | 0 | 0 | 0 | 1 |
| RPL8 | 0 | 0 | 0 | 0 | 0 | 0 | 1 |
| DSTN | 0 | 0 | 0 | 0 | 0 | 0 | 1 |
| ARHGEF2-AS2 | 0 | 0 | 0 | 0 | 0 | 0 | 1 |
| GRIP2 | 0 | 0 | 0 | 0 | 0 | 0 | 1 |
| RN7SL517P | 0 | 0 | 0 | 0 | 0 | 0 | 1 |
| CERS6 | 0 | 0 | 0 | 0 | 0 | 0 | 1 |
| SLC2A5 | 0 | 0 | 0 | 0 | 0 | 0 | 1 |
| ID2 | 0 | 0 | 0 | 0 | 0 | 0 | 1 |
| IQSEC3 | 0 | 0 | 0 | 0 | 0 | 0 | 1 |
| RHOQ | 0 | 0 | 0 | 0 | 0 | 0 | 1 |
| CUEDC1 | 0 | 0 | 0 | 0 | 0 | 0 | 1 |
| ORMDL1 | 0 | 0 | 0 | 0 | 0 | 0 | 1 |
| RBM24 | 0 | 0 | 0 | 0 | 0 | 0 | 1 |
| POPDC2 | 0 | 0 | 0 | 0 | 0 | 0 | 1 |
| HIGD1A | 0 | 0 | 0 | 0 | 0 | 0 | 1 |
| MAPK10 | 0 | 0 | 0 | 0 | 0 | 0 | 1 |
| HNRNPA1L3 | 0 | 0 | 0 | 0 | 0 | 0 | 1 |
| AIF1 | 0 | 0 | 0 | 0 | 0 | 0 | 1 |
| JCAD | 0 | 0 | 0 | 0 | 0 | 0 | 1 |
| UGCG | 0 | 0 | 0 | 0 | 0 | 0 | 1 |
| LINC02197 | 0 | 0 | 0 | 0 | 0 | 0 | 1 |
| FASTKD1 | 0 | 0 | 0 | 0 | 0 | 0 | 1 |
| CTNNBIP1 | 0 | 0 | 0 | 0 | 0 | 0 | 1 |
| SNORD3B-1 | 0 | 0 | 0 | 0 | 0 | 0 | 1 |
| APOO | 0 | 0 | 0 | 0 | 0 | 0 | 1 |
| SYF2 | 0 | 0 | 0 | 0 | 0 | 0 | 1 |
| KCNB1 | 0 | 0 | 0 | 0 | 0 | 0 | 1 |
| IGHA1 | 0 | 0 | 0 | 0 | 0 | 0 | 1 |
| ENSG00000230896 | 0 | 0 | 0 | 0 | 0 | 0 | 1 |
| ENSG00000227681 | 0 | 0 | 0 | 0 | 0 | 0 | 1 |
| RRS1-DT | 0 | 0 | 0 | 0 | 0 | 0 | 1 |
| RPL18 | 0 | 0 | 0 | 0 | 0 | 0 | 1 |
| EPB41L1 | 0 | 0 | 0 | 0 | 0 | 0 | 1 |
| GIPC1 | 0 | 0 | 0 | 0 | 0 | 0 | 1 |
| PCDHB15 | 0 | 0 | 0 | 0 | 0 | 0 | 1 |
| FDPS | 0 | 0 | 0 | 0 | 0 | 0 | 1 |
| SLC9A3R2 | 0 | 0 | 0 | 0 | 0 | 0 | 1 |
| RRP9 | 0 | 0 | 0 | 0 | 0 | 0 | 1 |
| DECR1 | 0 | 0 | 0 | 0 | 0 | 0 | 1 |
| ZBTB40-IT1 | 0 | 0 | 0 | 0 | 0 | 0 | 1 |
| ENSG00000261026 | 0 | 0 | 0 | 0 | 0 | 0 | 1 |
| DACH1 | 0 | 0 | 0 | 0 | 0 | 0 | 1 |
| GHR | 0 | 0 | 0 | 0 | 0 | 0 | 1 |
| ENSG00000278863 | 0 | 0 | 0 | 0 | 0 | 0 | 1 |
| REXO5 | 0 | 0 | 0 | 0 | 0 | 0 | 1 |
| SMOX | 0 | 0 | 0 | 0 | 0 | 0 | 1 |
| RPL26 | 0 | 0 | 0 | 0 | 0 | 0 | 1 |
| UQCRC2 | 0 | 0 | 0 | 0 | 0 | 0 | 1 |
| PRKG1 | 0 | 0 | 0 | 0 | 0 | 0 | 1 |
| OLFML3 | 0 | 0 | 0 | 0 | 0 | 0 | 1 |
| ECM1 | 0 | 0 | 0 | 0 | 0 | 0 | 1 |
| CES1 | 0 | 0 | 0 | 0 | 0 | 0 | 1 |
| MAP9 | 0 | 0 | 0 | 0 | 0 | 0 | 1 |
| SNORD13E | 0 | 0 | 0 | 0 | 0 | 0 | 1 |
| LHFPL6 | 0 | 0 | 0 | 0 | 0 | 0 | 1 |
| SLC25A42 | 0 | 0 | 0 | 0 | 0 | 0 | 1 |
| MTCO2P11 | 0 | 0 | 0 | 0 | 0 | 0 | 1 |
| TEKT5 | 0 | 0 | 0 | 0 | 0 | 0 | 1 |
| ENSG00000278876 | 0 | 0 | 0 | 0 | 0 | 0 | 1 |
| STK17B | 0 | 0 | 0 | 0 | 0 | 0 | 1 |
| RPS2P55 | 0 | 0 | 0 | 0 | 0 | 0 | 1 |
| NACAD | 0 | 0 | 0 | 0 | 0 | 0 | 1 |
| NAT14 | 0 | 0 | 0 | 0 | 0 | 0 | 1 |
| ACTG1P20 | 0 | 0 | 0 | 0 | 0 | 0 | 1 |
| LINC02916 | 0 | 0 | 0 | 0 | 0 | 0 | 1 |
| NFATC1 | 0 | 0 | 0 | 0 | 0 | 0 | 1 |
| TRAM2 | 0 | 0 | 0 | 0 | 0 | 0 | 1 |
| PHF2 | 0 | 0 | 0 | 0 | 0 | 0 | 1 |
| FAM229B | 0 | 0 | 0 | 0 | 0 | 0 | 1 |
| CHKA | 0 | 0 | 0 | 0 | 0 | 0 | 1 |
| NTHL1 | 0 | 0 | 0 | 0 | 0 | 0 | 1 |
| APMAP | 0 | 0 | 0 | 0 | 0 | 0 | 1 |
| ALKBH4 | 0 | 0 | 0 | 0 | 0 | 0 | 1 |
| ENSG00000269924 | 0 | 0 | 0 | 0 | 0 | 0 | 1 |
| PDLIM5 | 0 | 0 | 0 | 0 | 0 | 0 | 1 |
| CYP39A1 | 0 | 0 | 0 | 0 | 0 | 0 | 1 |
| LOX | 0 | 0 | 0 | 0 | 0 | 0 | 1 |
| PGAM1P7 | 0 | 0 | 0 | 0 | 0 | 0 | 1 |
| VDAC1P1 | 0 | 0 | 0 | 0 | 0 | 0 | 1 |
| ENSG00000283674 | 0 | 0 | 0 | 0 | 0 | 0 | 1 |
| WAPL-DT | 0 | 0 | 0 | 0 | 0 | 0 | 1 |
| YPEL1 | 0 | 0 | 0 | 0 | 0 | 0 | 1 |
| ZNF727 | 0 | 0 | 0 | 0 | 0 | 0 | 1 |
| GTF3A | 0 | 0 | 0 | 0 | 0 | 0 | 1 |
| ENSG00000264853 | 0 | 0 | 0 | 0 | 0 | 0 | 1 |
| MFAP3L | 0 | 0 | 0 | 0 | 0 | 0 | 1 |
| ENSG00000267096 | 0 | 0 | 0 | 0 | 0 | 0 | 1 |
| DCAF4L1 | 0 | 0 | 0 | 0 | 0 | 0 | 1 |
| ENSG00000227304 | 0 | 0 | 0 | 0 | 0 | 0 | 1 |
| MCM3AP-AS1 | 0 | 0 | 0 | 0 | 0 | 0 | 1 |
| TIMM23 | 0 | 0 | 0 | 0 | 0 | 0 | 1 |
| NRARP | 0 | 0 | 0 | 0 | 0 | 0 | 1 |
| STK32C | 0 | 0 | 0 | 0 | 0 | 0 | 1 |
| FAM131A | 0 | 0 | 0 | 0 | 0 | 0 | 1 |
| ITGA1 | 0 | 0 | 0 | 0 | 0 | 0 | 1 |
| DUSP3 | 0 | 0 | 0 | 0 | 0 | 0 | 1 |
| RAB25 | 0 | 0 | 0 | 0 | 0 | 0 | 1 |
| ENSG00000233967 | 0 | 0 | 0 | 0 | 0 | 0 | 1 |
| HUNK | 0 | 0 | 0 | 0 | 0 | 0 | 1 |
| MT1M | 0 | 0 | 0 | 0 | 0 | 0 | 1 |
| CIRBP | 0 | 0 | 0 | 0 | 0 | 0 | 1 |
| RPS15AP6 | 0 | 0 | 0 | 0 | 0 | 0 | 1 |
| FAM43A | 0 | 0 | 0 | 0 | 0 | 0 | 1 |
| FLT4 | 0 | 0 | 0 | 0 | 0 | 0 | 1 |
| GIMAP6 | 0 | 0 | 0 | 0 | 0 | 0 | 1 |
| CRADD | 0 | 0 | 0 | 0 | 0 | 0 | 1 |
| DUS3L | 0 | 0 | 0 | 0 | 0 | 0 | 1 |
| ENSG00000236525 | 0 | 0 | 0 | 0 | 0 | 0 | 1 |
| PCDHA4 | 0 | 0 | 0 | 0 | 0 | 0 | 1 |
| MT1E | 0 | 0 | 0 | 0 | 0 | 0 | 1 |
| H1-10 | 0 | 0 | 0 | 0 | 0 | 0 | 1 |
| ENSG00000257663 | 0 | 0 | 0 | 0 | 0 | 0 | 1 |
| TMEM54 | 0 | 0 | 0 | 0 | 0 | 0 | 1 |
| HSPA4L | 0 | 0 | 0 | 0 | 0 | 0 | 1 |
| IGHV5-51 | 0 | 0 | 0 | 0 | 0 | 0 | 1 |
| SC5D | 0 | 0 | 0 | 0 | 0 | 0 | 1 |
| TMEM30B | 0 | 0 | 0 | 0 | 0 | 0 | 1 |
| FADS2 | 0 | 0 | 0 | 0 | 0 | 0 | 1 |
| EFCAB6 | 0 | 0 | 0 | 0 | 0 | 0 | 1 |
| INAVA | 0 | 0 | 0 | 0 | 0 | 0 | 1 |
| DUSP18 | 0 | 0 | 0 | 0 | 0 | 0 | 1 |
| RN7SL398P | 0 | 0 | 0 | 0 | 0 | 0 | 1 |
| SLC5A3 | 0 | 0 | 0 | 0 | 0 | 0 | 1 |
| LINC00997 | 0 | 0 | 0 | 0 | 0 | 0 | 1 |
| FMO4 | 0 | 0 | 0 | 0 | 0 | 0 | 1 |
| RPL4P5 | 0 | 0 | 0 | 0 | 0 | 0 | 1 |
| SRL | 0 | 0 | 0 | 0 | 0 | 0 | 1 |
| EFNB2 | 0 | 0 | 0 | 0 | 0 | 0 | 1 |
| ENSG00000260971 | 0 | 0 | 0 | 0 | 0 | 0 | 1 |
| ULK2 | 0 | 0 | 0 | 0 | 0 | 0 | 1 |
| HYKK | 0 | 0 | 0 | 0 | 0 | 0 | 1 |
| ACSS1 | 0 | 0 | 0 | 0 | 0 | 0 | 1 |
| ENSG00000273691 | 0 | 0 | 0 | 0 | 0 | 0 | 1 |
| RPS15P4 | 0 | 0 | 0 | 0 | 0 | 0 | 1 |
| SYT17 | 0 | 0 | 0 | 0 | 0 | 0 | 1 |
| HIBCH | 0 | 0 | 0 | 0 | 0 | 0 | 1 |
| C1orf220 | 0 | 0 | 0 | 0 | 0 | 0 | 1 |
| ENSG00000255165 | 0 | 0 | 0 | 0 | 0 | 0 | 1 |
| USP11 | 0 | 0 | 0 | 0 | 0 | 0 | 1 |
| GATA2-AS1 | 0 | 0 | 0 | 0 | 0 | 0 | 1 |
| TMEM38B | 0 | 0 | 0 | 0 | 0 | 0 | 1 |
| LRRC34 | 0 | 0 | 0 | 0 | 0 | 0 | 1 |
| NPM1P37 | 0 | 0 | 0 | 0 | 0 | 0 | 1 |
| ACSS3 | 0 | 0 | 0 | 0 | 0 | 0 | 1 |
| ENSG00000232626 | 0 | 0 | 0 | 0 | 0 | 0 | 1 |
| ENSG00000261659 | 0 | 0 | 0 | 0 | 0 | 0 | 1 |
| GRHPR | 0 | 0 | 0 | 0 | 0 | 0 | 1 |
| ENSG00000256341 | 0 | 0 | 0 | 0 | 0 | 0 | 1 |
| HEG1 | 0 | 0 | 0 | 0 | 0 | 0 | 1 |
| ECHDC3 | 0 | 0 | 0 | 0 | 0 | 0 | 1 |
| SCARA5 | 0 | 0 | 0 | 0 | 0 | 0 | 1 |
| ENSG00000285679 | 0 | 0 | 0 | 0 | 0 | 0 | 1 |
| LRRC56 | 0 | 0 | 0 | 0 | 0 | 0 | 1 |
| ZC3H18 | 0 | 0 | 0 | 0 | 0 | 0 | 1 |
| ZDHHC9 | 0 | 0 | 0 | 0 | 0 | 0 | 1 |
| ARHGAP24 | 0 | 0 | 0 | 0 | 0 | 0 | 1 |
| ABCC6P2 | 0 | 0 | 0 | 0 | 0 | 0 | 1 |
| VWA2 | 0 | 0 | 0 | 0 | 0 | 0 | 1 |
| TPGS1 | 0 | 0 | 0 | 0 | 0 | 0 | 1 |
| BACH1-IT2 | 0 | 0 | 0 | 0 | 0 | 0 | 1 |
| RPS14P4 | 0 | 0 | 0 | 0 | 0 | 0 | 1 |
| TMEM120A | 0 | 0 | 0 | 0 | 0 | 0 | 1 |
| LCAT | 0 | 0 | 0 | 0 | 0 | 0 | 1 |
| ENSG00000273270 | 0 | 0 | 0 | 0 | 0 | 0 | 1 |
| ENSG00000235445 | 0 | 0 | 0 | 0 | 0 | 0 | 1 |
| CNKSR1 | 0 | 0 | 0 | 0 | 0 | 0 | 1 |
| ST14 | 0 | 0 | 0 | 0 | 0 | 0 | 1 |
| TOMM70 | 0 | 0 | 0 | 0 | 0 | 0 | 1 |
| ENSG00000287306 | 0 | 0 | 0 | 0 | 0 | 0 | 1 |
| LYPLA1 | 0 | 0 | 0 | 0 | 0 | 0 | 1 |
| SLFNL1-AS1 | 0 | 0 | 0 | 0 | 0 | 0 | 1 |
| MGC27382 | 0 | 0 | 0 | 0 | 0 | 0 | 1 |
| MYO10 | 0 | 0 | 0 | 0 | 0 | 0 | 1 |
| MAL2 | 0 | 0 | 0 | 0 | 0 | 0 | 1 |
| SSTR1 | 0 | 0 | 0 | 0 | 0 | 0 | 1 |
| TAS2R6P | 0 | 0 | 0 | 0 | 0 | 0 | 1 |
| NELL2 | 0 | 0 | 0 | 0 | 0 | 0 | 1 |
| UBA6 | 0 | 0 | 0 | 0 | 0 | 0 | 1 |
| RPL35AP2 | 0 | 0 | 0 | 0 | 0 | 0 | 1 |
| N4BP2L2-IT2 | 0 | 0 | 0 | 0 | 0 | 0 | 1 |
| ALDH5A1 | 0 | 0 | 0 | 0 | 0 | 0 | 1 |
| TMEM184A | 0 | 0 | 0 | 0 | 0 | 0 | 1 |
| PPP1R37 | 0 | 0 | 0 | 0 | 0 | 0 | 1 |
| SYCE1L | 0 | 0 | 0 | 0 | 0 | 0 | 1 |
| MTCYBP3 | 0 | 0 | 0 | 0 | 0 | 0 | 1 |
| MPL | 0 | 0 | 0 | 0 | 0 | 0 | 1 |
| MTND5P2 | 0 | 0 | 0 | 0 | 0 | 0 | 1 |
| CYP2S1 | 0 | 0 | 0 | 0 | 0 | 0 | 1 |
| MPC2 | 0 | 0 | 0 | 0 | 0 | 0 | 1 |
| ENSG00000274080 | 0 | 0 | 0 | 0 | 0 | 0 | 1 |
| OLR1 | 0 | 0 | 0 | 0 | 0 | 0 | 1 |
| SNORD46 | 0 | 0 | 0 | 0 | 0 | 0 | 1 |
| SDHB | 0 | 0 | 0 | 0 | 0 | 0 | 1 |
| PCSK5 | 0 | 0 | 0 | 0 | 0 | 0 | 1 |
| PPP1R13L | 0 | 0 | 0 | 0 | 0 | 0 | 1 |
| LRRC39 | 0 | 0 | 0 | 0 | 0 | 0 | 1 |
| ADHFE1 | 0 | 0 | 0 | 0 | 0 | 0 | 1 |
| ENSG00000273192 | 0 | 0 | 0 | 0 | 0 | 0 | 1 |
| HSP90B2P | 0 | 0 | 0 | 0 | 0 | 0 | 1 |
| ENSG00000272754 | 0 | 0 | 0 | 0 | 0 | 0 | 1 |
| MYO5C | 0 | 0 | 0 | 0 | 0 | 0 | 1 |
| MRPS31P5 | 0 | 0 | 0 | 0 | 0 | 0 | 1 |
| HSD17B13 | 0 | 0 | 0 | 0 | 0 | 0 | 1 |
| CLIC6 | 0 | 0 | 0 | 0 | 0 | 0 | 1 |
| C5orf34 | 0 | 0 | 0 | 0 | 0 | 0 | 1 |
| POLN | 0 | 0 | 0 | 0 | 0 | 0 | 1 |
| TMEM30BP1 | 0 | 0 | 0 | 0 | 0 | 0 | 1 |
| CHCHD10 | 0 | 0 | 0 | 0 | 0 | 0 | 1 |
| LRRC37A15P | 0 | 0 | 0 | 0 | 0 | 0 | 1 |
| NEXMIF | 0 | 0 | 0 | 0 | 0 | 0 | 1 |
| CCDC74A | 0 | 0 | 0 | 0 | 0 | 0 | 1 |
| HARS1 | 0 | 0 | 0 | 0 | 0 | 0 | 1 |
| LINC02246 | 0 | 0 | 0 | 0 | 0 | 0 | 1 |
| TMEM125 | 0 | 0 | 0 | 0 | 0 | 0 | 1 |
| ENSG00000260855 | 0 | 0 | 0 | 0 | 0 | 0 | 1 |
| ZNF783 | 0 | 0 | 0 | 0 | 0 | 0 | 1 |
| CASC2 | 0 | 0 | 0 | 0 | 0 | 0 | 1 |
| ADRB2 | 0 | 0 | 0 | 0 | 0 | 0 | 1 |
| H1-1 | 0 | 0 | 0 | 0 | 0 | 0 | 1 |
| TAS2R4 | 0 | 0 | 0 | 0 | 0 | 0 | 1 |
| GLIS2 | 0 | 0 | 0 | 0 | 0 | 0 | 1 |
| DNHD1 | 0 | 0 | 0 | 0 | 0 | 0 | 1 |
| RAB19 | 0 | 0 | 0 | 0 | 0 | 0 | 1 |
| ADAMTS17 | 0 | 0 | 0 | 0 | 0 | 0 | 1 |
| ENSG00000278351 | 0 | 0 | 0 | 0 | 0 | 0 | 1 |
| PA2G4P4 | 0 | 0 | 0 | 0 | 0 | 0 | 1 |
| SIPA1L3 | 0 | 0 | 0 | 0 | 0 | 0 | 1 |
| MANF | 0 | 0 | 0 | 0 | 0 | 0 | 1 |
| MSRB3 | 0 | 0 | 0 | 0 | 0 | 0 | 1 |
| PRDM6 | 0 | 0 | 0 | 0 | 0 | 0 | 1 |
| ASL | 0 | 0 | 0 | 0 | 0 | 0 | 1 |
| GPM6B | 0 | 0 | 0 | 0 | 0 | 0 | 1 |
| NDUFAF4 | 0 | 0 | 0 | 0 | 0 | 0 | 1 |
| ARMCX6 | 0 | 0 | 0 | 0 | 0 | 0 | 1 |
| EEF1A1P9 | 0 | 0 | 0 | 0 | 0 | 0 | 1 |
| LINC00924 | 0 | 0 | 0 | 0 | 0 | 0 | 1 |
| ENSG00000280120 | 0 | 0 | 0 | 0 | 0 | 0 | 1 |
| ENSG00000238390 | 0 | 0 | 0 | 0 | 0 | 0 | 1 |
| APOBEC3C | 0 | 0 | 0 | 0 | 0 | 0 | 1 |
| KCNN3 | 0 | 0 | 0 | 0 | 0 | 0 | 1 |
| ENSG00000259692 | 0 | 0 | 0 | 0 | 0 | 0 | 1 |
| GOLGA6L5P | 0 | 0 | 0 | 0 | 0 | 0 | 1 |
| RPP25 | 0 | 0 | 0 | 0 | 0 | 0 | 1 |
| ENSG00000270012 | 0 | 0 | 0 | 0 | 0 | 0 | 1 |
| PMVK | 0 | 0 | 0 | 0 | 0 | 0 | 1 |
| RPL24 | 0 | 0 | 0 | 0 | 0 | 0 | 1 |
| SUCLG2 | 0 | 0 | 0 | 0 | 0 | 0 | 1 |
| GBP2 | 0 | 0 | 0 | 0 | 0 | 0 | 1 |
| RGS11 | 0 | 0 | 0 | 0 | 0 | 0 | 1 |
| NFKBIL1 | 0 | 0 | 0 | 0 | 0 | 0 | 1 |
| GARS1 | 0 | 0 | 0 | 0 | 0 | 0 | 1 |
| FGF9 | 0 | 0 | 0 | 0 | 0 | 0 | 1 |
| BCL2L2-PABPN1 | 0 | 0 | 0 | 0 | 0 | 0 | 1 |
| SCEL | 0 | 0 | 0 | 0 | 0 | 0 | 1 |
| ENSG00000213703 | 0 | 0 | 0 | 0 | 0 | 0 | 1 |
| ENSG00000273568 | 0 | 0 | 0 | 0 | 0 | 0 | 1 |
| ENSG00000280077 | 0 | 0 | 0 | 0 | 0 | 0 | 1 |
| RNU6-1157P | 0 | 0 | 0 | 0 | 0 | 0 | 1 |
| RN7SL430P | 0 | 0 | 0 | 0 | 0 | 0 | 1 |
| RAB1AP1 | 0 | 0 | 0 | 0 | 0 | 0 | 1 |
| NHLRC1 | 0 | 0 | 0 | 0 | 0 | 0 | 1 |
| ZDHHC2 | 0 | 0 | 0 | 0 | 0 | 0 | 1 |
| EFHD2 | 0 | 0 | 0 | 0 | 0 | 0 | 1 |
| MSANTD3 | 0 | 0 | 0 | 0 | 0 | 0 | 1 |
| FDFT1 | 0 | 0 | 0 | 0 | 0 | 0 | 1 |
| ANP32E | 0 | 0 | 0 | 0 | 0 | 0 | 1 |
| MCM3 | 0 | 0 | 0 | 0 | 0 | 0 | 1 |
| DNA2 | 0 | 0 | 0 | 0 | 0 | 0 | 1 |
| TIMP3 | 0 | 0 | 0 | 0 | 0 | 0 | 1 |
| COL18A1 | 0 | 0 | 0 | 0 | 0 | 0 | 1 |
| PPP1R36 | 0 | 0 | 0 | 0 | 0 | 0 | 1 |
| ENSG00000283057 | 0 | 0 | 0 | 0 | 0 | 0 | 1 |
| FDXR | 0 | 0 | 0 | 0 | 0 | 0 | 1 |
| TMEM38A | 0 | 0 | 0 | 0 | 0 | 0 | 1 |
| RHOF | 0 | 0 | 0 | 0 | 0 | 0 | 1 |
| TKFC | 0 | 0 | 0 | 0 | 0 | 0 | 1 |
| TSPAN32 | 0 | 0 | 0 | 0 | 0 | 0 | 1 |
| OSMR | 0 | 0 | 0 | 0 | 0 | 0 | 1 |
| NELFB | 0 | 0 | 0 | 0 | 0 | 0 | 1 |
| RGL2 | 0 | 0 | 0 | 0 | 0 | 0 | 1 |
| ENSG00000257681 | 0 | 0 | 0 | 0 | 0 | 0 | 1 |
| EIF3C | 0 | 0 | 0 | 0 | 0 | 0 | 1 |
| PTGER4 | 0 | 0 | 0 | 0 | 0 | 0 | 1 |
| EDNRA | 0 | 0 | 0 | 0 | 0 | 0 | 1 |
| EIF4BP7 | 0 | 0 | 0 | 0 | 0 | 0 | 1 |
| ENSG00000203644 | 0 | 0 | 0 | 0 | 0 | 0 | 1 |
| ENSG00000239280 | 0 | 0 | 0 | 0 | 0 | 0 | 1 |
| LRRCC1 | 0 | 0 | 0 | 0 | 0 | 0 | 1 |
| ENSG00000279539 | 0 | 0 | 0 | 0 | 0 | 0 | 1 |
| FBL | 0 | 0 | 0 | 0 | 0 | 0 | 1 |
| LYRM4-AS1 | 0 | 0 | 0 | 0 | 0 | 0 | 1 |
| ZNF668 | 0 | 0 | 0 | 0 | 0 | 0 | 1 |
| INSR | 0 | 0 | 0 | 0 | 0 | 0 | 1 |
| ENSG00000279106 | 0 | 0 | 0 | 0 | 0 | 0 | 1 |
| GNGT2 | 0 | 0 | 0 | 0 | 0 | 0 | 1 |
| CPEB3 | 0 | 0 | 0 | 0 | 0 | 0 | 1 |
| HESX1 | 0 | 0 | 0 | 0 | 0 | 0 | 1 |
| FUCA2 | 0 | 0 | 0 | 0 | 0 | 0 | 1 |
| HMGB1P6 | 0 | 0 | 0 | 0 | 0 | 0 | 1 |
| RPL41 | 0 | 0 | 0 | 0 | 0 | 0 | 1 |
| RN7SL473P | 0 | 0 | 0 | 0 | 0 | 0 | 1 |
| GLIPR1-AS1 | 0 | 0 | 0 | 0 | 0 | 0 | 1 |
| ASB13 | 0 | 0 | 0 | 0 | 0 | 0 | 1 |
| SLC30A1 | 0 | 0 | 0 | 0 | 0 | 0 | 1 |
| ARHGAP31 | 0 | 0 | 0 | 0 | 0 | 0 | 1 |
| HACD4 | 0 | 0 | 0 | 0 | 0 | 0 | 1 |
| SCN5A | 0 | 0 | 0 | 0 | 0 | 0 | 1 |
| IFT22 | 0 | 0 | 0 | 0 | 0 | 0 | 1 |
| TRADD | 0 | 0 | 0 | 0 | 0 | 0 | 1 |
| ASAP1-IT2 | 0 | 0 | 0 | 0 | 0 | 0 | 1 |
| ISOC1 | 0 | 0 | 0 | 0 | 0 | 0 | 1 |
| FAM124B | 0 | 0 | 0 | 0 | 0 | 0 | 1 |
| FZD6 | 0 | 0 | 0 | 0 | 0 | 0 | 1 |
| PLTP | 0 | 0 | 0 | 0 | 0 | 0 | 1 |
| TM2D3 | 0 | 0 | 0 | 0 | 0 | 0 | 1 |
| SLC25A24 | 0 | 0 | 0 | 0 | 0 | 0 | 1 |
| ILDR1 | 0 | 0 | 0 | 0 | 0 | 0 | 1 |
| MTCO3P11 | 0 | 0 | 0 | 0 | 0 | 0 | 1 |
| MYLIP | 0 | 0 | 0 | 0 | 0 | 0 | 1 |
| ISLR | 0 | 0 | 0 | 0 | 0 | 0 | 1 |
| GYG2 | 0 | 0 | 0 | 0 | 0 | 0 | 1 |
| MPI | 0 | 0 | 0 | 0 | 0 | 0 | 1 |
| MRC1 | 0 | 0 | 0 | 0 | 0 | 0 | 1 |
| GABRR2 | 0 | 0 | 0 | 0 | 0 | 0 | 1 |
| TBX2 | 0 | 0 | 0 | 0 | 0 | 0 | 1 |
| WDR19 | 0 | 0 | 0 | 0 | 0 | 0 | 1 |
| TIE1 | 0 | 0 | 0 | 0 | 0 | 0 | 1 |
| ENSG00000278058 | 0 | 0 | 0 | 0 | 0 | 0 | 1 |
| CBX6 | 0 | 0 | 0 | 0 | 0 | 0 | 1 |
| PARD6G | 0 | 0 | 0 | 0 | 0 | 0 | 1 |
| TPD52L1 | 0 | 0 | 0 | 0 | 0 | 0 | 1 |
| TMEM123 | 0 | 0 | 0 | 0 | 0 | 0 | 1 |
| CCDC150 | 0 | 0 | 0 | 0 | 0 | 0 | 1 |
| MCF2L-AS1 | 0 | 0 | 0 | 0 | 0 | 0 | 1 |
| ADAMTSL4-AS1 | 0 | 0 | 0 | 0 | 0 | 0 | 1 |
| ENSG00000244398 | 0 | 0 | 0 | 0 | 0 | 0 | 1 |
| FAM76B | 0 | 0 | 0 | 0 | 0 | 0 | 1 |
| LDLRAD3 | 0 | 0 | 0 | 0 | 0 | 0 | 1 |
| MT-ND4L | 0 | 0 | 0 | 0 | 0 | 0 | 1 |
| EGOT | 0 | 0 | 0 | 0 | 0 | 0 | 1 |
| MIR3679 | 0 | 0 | 0 | 0 | 0 | 0 | 1 |
| IFITM4P | 0 | 0 | 0 | 0 | 0 | 0 | 1 |
| ADARB2 | 0 | 0 | 0 | 0 | 0 | 0 | 1 |
| C2orf72 | 0 | 0 | 0 | 0 | 0 | 0 | 1 |
| STXBP1 | 0 | 0 | 0 | 0 | 0 | 0 | 1 |
| IGHV4-59 | 0 | 0 | 0 | 0 | 0 | 0 | 1 |
| ZFP1 | 0 | 0 | 0 | 0 | 0 | 0 | 1 |
| MEGF10 | 0 | 0 | 0 | 0 | 0 | 0 | 1 |
| ENSG00000255026 | 0 | 0 | 0 | 0 | 0 | 0 | 1 |
| RN7SL674P | 0 | 0 | 0 | 0 | 0 | 0 | 1 |
| PYCARD | 0 | 0 | 0 | 0 | 0 | 0 | 1 |
| PTX3 | 0 | 0 | 0 | 0 | 0 | 0 | 1 |
| ENSG00000270072 | 0 | 0 | 0 | 0 | 0 | 0 | 1 |
| SYTL5 | 0 | 0 | 0 | 0 | 0 | 0 | 1 |
| COLEC12 | 0 | 0 | 0 | 0 | 0 | 0 | 1 |
| GPR155 | 0 | 0 | 0 | 0 | 0 | 0 | 1 |
| C22orf46 | 0 | 0 | 0 | 0 | 0 | 0 | 1 |
| TMEM17 | 0 | 0 | 0 | 0 | 0 | 0 | 1 |
| GPR162 | 0 | 0 | 0 | 0 | 0 | 0 | 1 |
| NBL1 | 0 | 0 | 0 | 0 | 0 | 0 | 1 |
| SFXN4 | 0 | 0 | 0 | 0 | 0 | 0 | 1 |
| WWTR1-AS1 | 0 | 0 | 0 | 0 | 0 | 0 | 1 |
| LINC02028 | 0 | 0 | 0 | 0 | 0 | 0 | 1 |
| MT-TE | 0 | 0 | 0 | 0 | 0 | 0 | 1 |
| BACE2-IT1 | 0 | 0 | 0 | 0 | 0 | 0 | 1 |
| TMC7 | 0 | 0 | 0 | 0 | 0 | 0 | 1 |
| GIGYF1 | 0 | 0 | 0 | 0 | 0 | 0 | 1 |
| LRRC28 | 0 | 0 | 0 | 0 | 0 | 0 | 1 |
| SEC61B | 0 | 0 | 0 | 0 | 0 | 0 | 1 |
| DCN | 0 | 0 | 0 | 0 | 0 | 0 | 1 |
| GUCY1A1 | 0 | 0 | 0 | 0 | 0 | 0 | 1 |
| ESRP1 | 0 | 0 | 0 | 0 | 0 | 0 | 1 |
| CACNB4 | 0 | 0 | 0 | 0 | 0 | 0 | 1 |
| FSCN1 | 0 | 0 | 0 | 0 | 0 | 0 | 1 |
| PRORSD1P | 0 | 0 | 0 | 0 | 0 | 0 | 1 |
| UBE2V1P1 | 0 | 0 | 0 | 0 | 0 | 0 | 1 |
| Y_RNA | 0 | 0 | 0 | 0 | 0 | 0 | 1 |
| PCYT2 | 0 | 0 | 0 | 0 | 0 | 0 | 1 |
| S100A16 | 0 | 0 | 0 | 0 | 0 | 0 | 1 |
| MRPL2 | 0 | 0 | 0 | 0 | 0 | 0 | 1 |
| CHST10 | 0 | 0 | 0 | 0 | 0 | 0 | 1 |
| ANGPT1 | 0 | 0 | 0 | 0 | 0 | 0 | 1 |
| TEX41 | 0 | 0 | 0 | 0 | 0 | 0 | 1 |
| TSPAN6 | 0 | 0 | 0 | 0 | 0 | 0 | 1 |
| COL27A1 | 0 | 0 | 0 | 0 | 0 | 0 | 1 |
| MIA3 | 0 | 0 | 0 | 0 | 0 | 0 | 1 |
| ENSG00000233461 | 0 | 0 | 0 | 0 | 0 | 0 | 1 |
| ENSG00000288031 | 0 | 0 | 0 | 0 | 0 | 0 | 1 |
| MOCS2 | 0 | 0 | 0 | 0 | 0 | 0 | 1 |
| MGAT4B | 0 | 0 | 0 | 0 | 0 | 0 | 1 |
| ZMAT3 | 0 | 0 | 0 | 0 | 0 | 0 | 1 |
| CNTLN | 0 | 0 | 0 | 0 | 0 | 0 | 1 |
| PIGR | 0 | 0 | 0 | 0 | 0 | 0 | 1 |
| ENSG00000259392 | 0 | 0 | 0 | 0 | 0 | 0 | 1 |
| FAM210A | 0 | 0 | 0 | 0 | 0 | 0 | 1 |
| ENSG00000273724 | 0 | 0 | 0 | 0 | 0 | 0 | 1 |
| ACVR1C | 0 | 0 | 0 | 0 | 0 | 0 | 1 |
| LINC01169 | 0 | 0 | 0 | 0 | 0 | 0 | 1 |
| THSD1 | 0 | 0 | 0 | 0 | 0 | 0 | 1 |
| SPATA18 | 0 | 0 | 0 | 0 | 0 | 0 | 1 |
| ENSG00000276809 | 0 | 0 | 0 | 0 | 0 | 0 | 1 |
| AIF1L | 0 | 0 | 0 | 0 | 0 | 0 | 1 |
| RPL13AP25 | 0 | 0 | 0 | 0 | 0 | 0 | 1 |
| COMMD8 | 0 | 0 | 0 | 0 | 0 | 0 | 1 |
| LRCH2 | 0 | 0 | 0 | 0 | 0 | 0 | 1 |
| TRAF1 | 0 | 0 | 0 | 0 | 0 | 0 | 1 |
| IQSEC2 | 0 | 0 | 0 | 0 | 0 | 0 | 1 |
| ACE | 0 | 0 | 0 | 0 | 0 | 0 | 1 |
| ENSG00000273165 | 0 | 0 | 0 | 0 | 0 | 0 | 1 |
| LRRC8E | 0 | 0 | 0 | 0 | 0 | 0 | 1 |
| ACOT11 | 0 | 0 | 0 | 0 | 0 | 0 | 1 |
| HMGA1 | 0 | 0 | 0 | 0 | 0 | 0 | 1 |
| ALDH1B1 | 0 | 0 | 0 | 0 | 0 | 0 | 1 |
| SSPN | 0 | 0 | 0 | 0 | 0 | 0 | 1 |
| C16orf91 | 0 | 0 | 0 | 0 | 0 | 0 | 1 |
| CYP4Z1 | 0 | 0 | 0 | 0 | 0 | 0 | 1 |
| RORA | 0 | 0 | 0 | 0 | 0 | 0 | 1 |
| ENSG00000288663 | 0 | 0 | 0 | 0 | 0 | 0 | 1 |
| CNTROB | 0 | 0 | 0 | 0 | 0 | 0 | 1 |
| PLA2G5 | 0 | 0 | 0 | 0 | 0 | 0 | 1 |
| MXD4 | 0 | 0 | 0 | 0 | 0 | 0 | 1 |
| ENSG00000272079 | 0 | 0 | 0 | 0 | 0 | 0 | 1 |
| ENSG00000261476 | 0 | 0 | 0 | 0 | 0 | 0 | 1 |
| ENSG00000270190 | 0 | 0 | 0 | 0 | 0 | 0 | 1 |
| RAB7B | 0 | 0 | 0 | 0 | 0 | 0 | 1 |
| SNTB1 | 0 | 0 | 0 | 0 | 0 | 0 | 1 |
| PLA1A | 0 | 0 | 0 | 0 | 0 | 0 | 1 |
| RRN3P2 | 0 | 0 | 0 | 0 | 0 | 0 | 1 |
| PALM | 0 | 0 | 0 | 0 | 0 | 0 | 1 |
| ENSG00000260007 | 0 | 0 | 0 | 0 | 0 | 0 | 1 |
| HTRA3 | 0 | 0 | 0 | 0 | 0 | 0 | 1 |
| RAB40C | 0 | 0 | 0 | 0 | 0 | 0 | 1 |
| SEMA4F | 0 | 0 | 0 | 0 | 0 | 0 | 1 |
| ENSG00000269189 | 0 | 0 | 0 | 0 | 0 | 0 | 1 |
| COX17 | 0 | 0 | 0 | 0 | 0 | 0 | 1 |
| HSPA9 | 0 | 0 | 0 | 0 | 0 | 0 | 1 |
| SH2D3C | 0 | 0 | 0 | 0 | 0 | 0 | 1 |
| NYNRIN | 0 | 0 | 0 | 0 | 0 | 0 | 1 |
| ENSG00000257181 | 0 | 0 | 0 | 0 | 0 | 0 | 1 |
| ECSCR | 0 | 0 | 0 | 0 | 0 | 0 | 1 |
| ITGB4 | 0 | 0 | 0 | 0 | 0 | 0 | 1 |
| LNP1 | 0 | 0 | 0 | 0 | 0 | 0 | 1 |
| ZNF580 | 0 | 0 | 0 | 0 | 0 | 0 | 1 |
| RPP40 | 0 | 0 | 0 | 0 | 0 | 0 | 1 |
| ENSG00000256682 | 0 | 0 | 0 | 0 | 0 | 0 | 1 |
| FRS3 | 0 | 0 | 0 | 0 | 0 | 0 | 1 |
| CYP2G1P | 0 | 0 | 0 | 0 | 0 | 0 | 1 |
| TMEM205 | 0 | 0 | 0 | 0 | 0 | 0 | 1 |
| RPS2P5 | 0 | 0 | 0 | 0 | 0 | 0 | 1 |
| ZBTB45 | 0 | 0 | 0 | 0 | 0 | 0 | 1 |
| ENSG00000219712 | 0 | 0 | 0 | 0 | 0 | 0 | 1 |
| TAS2R13 | 0 | 0 | 0 | 0 | 0 | 0 | 1 |
| KDELR3 | 0 | 0 | 0 | 0 | 0 | 0 | 1 |
| NDUFAB1 | 0 | 0 | 0 | 0 | 0 | 0 | 1 |
| IGLV3-1 | 0 | 0 | 0 | 0 | 0 | 0 | 1 |
| RPL23AP37 | 0 | 0 | 0 | 0 | 0 | 0 | 1 |
| ENSG00000271011 | 0 | 0 | 0 | 0 | 0 | 0 | 1 |
| F2RL1 | 0 | 0 | 0 | 0 | 0 | 0 | 1 |
| RNU7-124P | 0 | 0 | 0 | 0 | 0 | 0 | 1 |
| SEPHS2 | 0 | 0 | 0 | 0 | 0 | 0 | 1 |
| MACC1 | 0 | 0 | 0 | 0 | 0 | 0 | 1 |
| LTB4R | 0 | 0 | 0 | 0 | 0 | 0 | 1 |
| CPEB4 | 0 | 0 | 0 | 0 | 0 | 0 | 1 |
| GALNT2 | 0 | 0 | 0 | 0 | 0 | 0 | 1 |
| PREB | 0 | 0 | 0 | 0 | 0 | 0 | 1 |
| FGFBP3 | 0 | 0 | 0 | 0 | 0 | 0 | 1 |
| ACAD11 | 0 | 0 | 0 | 0 | 0 | 0 | 1 |
| CRISPLD1 | 0 | 0 | 0 | 0 | 0 | 0 | 1 |
| ENSG00000268707 | 0 | 0 | 0 | 0 | 0 | 0 | 1 |
| PECR | 0 | 0 | 0 | 0 | 0 | 0 | 1 |
| ZNF503 | 0 | 0 | 0 | 0 | 0 | 0 | 1 |
| RCAN1 | 0 | 0 | 0 | 0 | 0 | 0 | 1 |
| RN7SL57P | 0 | 0 | 0 | 0 | 0 | 0 | 1 |
| KSR2 | 0 | 0 | 0 | 0 | 0 | 0 | 1 |
| MIR3142HG | 0 | 0 | 0 | 0 | 0 | 0 | 1 |
| SLC52A3 | 0 | 0 | 0 | 0 | 0 | 0 | 1 |
| SPACA9 | 0 | 0 | 0 | 0 | 0 | 0 | 1 |
| SLC39A10 | 0 | 0 | 0 | 0 | 0 | 0 | 1 |
| NBPF25P | 0 | 0 | 0 | 0 | 0 | 0 | 1 |
| ENSG00000261544 | 0 | 0 | 0 | 0 | 0 | 0 | 1 |
| ENSG00000284428 | 0 | 0 | 0 | 0 | 0 | 0 | 1 |
| PCDHGA2 | 0 | 0 | 0 | 0 | 0 | 0 | 1 |
| FAM13C | 0 | 0 | 0 | 0 | 0 | 0 | 1 |
| LIPH | 0 | 0 | 0 | 0 | 0 | 0 | 1 |
| E2F8 | 0 | 0 | 0 | 0 | 0 | 0 | 1 |
| CBFA2T3 | 0 | 0 | 0 | 0 | 0 | 0 | 1 |
| LINC00702 | 0 | 0 | 0 | 0 | 0 | 0 | 1 |
| APOBEC3F | 0 | 0 | 0 | 0 | 0 | 0 | 1 |
| MALRD1 | 0 | 0 | 0 | 0 | 0 | 0 | 1 |
| ZNF724 | 0 | 0 | 0 | 0 | 0 | 0 | 1 |
| IGSF8 | 0 | 0 | 0 | 0 | 0 | 0 | 1 |
| SNORA22 | 0 | 0 | 0 | 0 | 0 | 0 | 1 |
| TRPV6 | 0 | 0 | 0 | 0 | 0 | 0 | 1 |
| RPL23A | 0 | 0 | 0 | 0 | 0 | 0 | 1 |
| ENSG00000217648 | 0 | 0 | 0 | 0 | 0 | 0 | 1 |
| IGHV4-39 | 0 | 0 | 0 | 0 | 0 | 0 | 1 |
| SOCS3 | 0 | 0 | 0 | 0 | 0 | 0 | 1 |
| CEBPG | 0 | 0 | 0 | 0 | 0 | 0 | 1 |
| PSTK | 0 | 0 | 0 | 0 | 0 | 0 | 1 |
| TMEM98 | 0 | 0 | 0 | 0 | 0 | 0 | 1 |
| GTF2IP1 | 0 | 0 | 0 | 0 | 0 | 0 | 1 |
| FAM110A | 0 | 0 | 0 | 0 | 0 | 0 | 1 |
| RGCC | 0 | 0 | 0 | 0 | 0 | 0 | 1 |
| SLC27A4 | 0 | 0 | 0 | 0 | 0 | 0 | 1 |
| SPACA6 | 0 | 0 | 0 | 0 | 0 | 0 | 1 |
| ERG28 | 0 | 0 | 0 | 0 | 0 | 0 | 1 |
| RHOD | 0 | 0 | 0 | 0 | 0 | 0 | 1 |
| PPIAP30 | 0 | 0 | 0 | 0 | 0 | 0 | 1 |
| CD151 | 0 | 0 | 0 | 0 | 0 | 0 | 1 |
| GIMAP1 | 0 | 0 | 0 | 0 | 0 | 0 | 1 |
| EIF2S2P4 | 0 | 0 | 0 | 0 | 0 | 0 | 1 |
| MICE | 0 | 0 | 0 | 0 | 0 | 0 | 1 |
| NPM2 | 0 | 0 | 0 | 0 | 0 | 0 | 1 |
| MIR558 | 0 | 0 | 0 | 0 | 0 | 0 | 1 |
| ESCO2 | 0 | 0 | 0 | 0 | 0 | 0 | 1 |
| ENSG00000276603 | 0 | 0 | 0 | 0 | 0 | 0 | 1 |
| PRRT2 | 0 | 0 | 0 | 0 | 0 | 0 | 1 |
| TTLL1 | 0 | 0 | 0 | 0 | 0 | 0 | 1 |
| PLEKHG5 | 0 | 0 | 0 | 0 | 0 | 0 | 1 |
| SCART1 | 0 | 0 | 0 | 0 | 0 | 0 | 1 |
| ENSG00000278434 | 0 | 0 | 0 | 0 | 0 | 0 | 1 |
| GCLM | 0 | 0 | 0 | 0 | 0 | 0 | 1 |
| ENSG00000266371 | 0 | 0 | 0 | 0 | 0 | 0 | 1 |
| COTL1 | 0 | 0 | 0 | 0 | 0 | 0 | 1 |
| ST8SIA5 | 0 | 0 | 0 | 0 | 0 | 0 | 1 |
| RPS4X | 0 | 0 | 0 | 0 | 0 | 0 | 1 |
| SLC15A2 | 0 | 0 | 0 | 0 | 0 | 0 | 1 |
| PLCL1 | 0 | 0 | 0 | 0 | 0 | 0 | 1 |
| RPS6KL1 | 0 | 0 | 0 | 0 | 0 | 0 | 1 |
| ZFTA | 0 | 0 | 0 | 0 | 0 | 0 | 1 |
| PWRN1 | 0 | 0 | 0 | 0 | 0 | 0 | 1 |
| SLC25A37 | 0 | 0 | 0 | 0 | 0 | 0 | 1 |
| SCD | 0 | 0 | 0 | 0 | 0 | 0 | 1 |
| TCEA1P4 | 0 | 0 | 0 | 0 | 0 | 0 | 1 |
| ARL5B | 0 | 0 | 0 | 0 | 0 | 0 | 1 |
| GPRC5C | 0 | 0 | 0 | 0 | 0 | 0 | 1 |
| TOB1-AS1 | 0 | 0 | 0 | 0 | 0 | 0 | 1 |
| H4C1 | 0 | 0 | 0 | 0 | 0 | 0 | 1 |
| PID1 | 0 | 0 | 0 | 0 | 0 | 0 | 1 |
| MYO16 | 0 | 0 | 0 | 0 | 0 | 0 | 1 |
| PEG10 | 0 | 0 | 0 | 0 | 0 | 0 | 1 |
| RNH1 | 0 | 0 | 0 | 0 | 0 | 0 | 1 |
| MRPL32 | 0 | 0 | 0 | 0 | 0 | 0 | 1 |
| RPL9 | 0 | 0 | 0 | 0 | 0 | 0 | 1 |
| SPA17 | 0 | 0 | 0 | 0 | 0 | 0 | 1 |
| SGSM1 | 0 | 0 | 0 | 0 | 0 | 0 | 1 |
| IYD | 0 | 0 | 0 | 0 | 0 | 0 | 1 |
| KLHL29 | 0 | 0 | 0 | 0 | 0 | 0 | 1 |
| GMFG | 0 | 0 | 0 | 0 | 0 | 0 | 1 |
| MCM5 | 0 | 0 | 0 | 0 | 0 | 0 | 1 |
| FRZB | 0 | 0 | 0 | 0 | 0 | 0 | 1 |
| CADPS | 0 | 0 | 0 | 0 | 0 | 0 | 1 |
| ADAM19 | 0 | 0 | 0 | 0 | 0 | 0 | 1 |
| VIM | 0 | 0 | 0 | 0 | 0 | 0 | 1 |
| ARHGAP23 | 0 | 0 | 0 | 0 | 0 | 0 | 1 |
| ENSG00000259407 | 0 | 0 | 0 | 0 | 0 | 0 | 1 |
| CAVIN4 | 0 | 0 | 0 | 0 | 0 | 0 | 1 |
| RPL13P5 | 0 | 0 | 0 | 0 | 0 | 0 | 1 |
| RN7SL809P | 0 | 0 | 0 | 0 | 0 | 0 | 1 |
| CCDC106 | 0 | 0 | 0 | 0 | 0 | 0 | 1 |
| ADCK2 | 0 | 0 | 0 | 0 | 0 | 0 | 1 |
| SEMA4C | 0 | 0 | 0 | 0 | 0 | 0 | 1 |
| ENSG00000273259 | 0 | 0 | 0 | 0 | 0 | 0 | 1 |
| ITGA3 | 0 | 0 | 0 | 0 | 0 | 0 | 1 |
| MIR6081 | 0 | 0 | 0 | 0 | 0 | 0 | 1 |
| RNU6-759P | 0 | 0 | 0 | 0 | 0 | 0 | 1 |
| WHAMMP1 | 0 | 0 | 0 | 0 | 0 | 0 | 1 |
| ENSG00000280351 | 0 | 0 | 0 | 0 | 0 | 0 | 1 |
| ZNF777 | 0 | 0 | 0 | 0 | 0 | 0 | 1 |
| TMEM220 | 0 | 0 | 0 | 0 | 0 | 0 | 1 |
| PLCB3 | 0 | 0 | 0 | 0 | 0 | 0 | 1 |
| CD300LB | 0 | 0 | 0 | 0 | 0 | 0 | 1 |
| MAGEE1 | 0 | 0 | 0 | 0 | 0 | 0 | 1 |
| ECI2 | 0 | 0 | 0 | 0 | 0 | 0 | 1 |
| MGAM | 0 | 0 | 0 | 0 | 0 | 0 | 1 |
| ADTRP | 0 | 0 | 0 | 0 | 0 | 0 | 1 |
| IGHV3-30 | 0 | 0 | 0 | 0 | 0 | 0 | 1 |
| RFPL3S | 0 | 0 | 0 | 0 | 0 | 0 | 1 |
| TEX30 | 0 | 0 | 0 | 0 | 0 | 0 | 1 |
| ENSG00000257531 | 0 | 0 | 0 | 0 | 0 | 0 | 1 |
| CYP2J2 | 0 | 0 | 0 | 0 | 0 | 0 | 1 |
| GALT | 0 | 0 | 0 | 0 | 0 | 0 | 1 |
| FGGY | 0 | 0 | 0 | 0 | 0 | 0 | 1 |
| PCOLCE2 | 0 | 0 | 0 | 0 | 0 | 0 | 1 |
| ENSG00000261291 | 0 | 0 | 0 | 0 | 0 | 0 | 1 |
| ENSG00000280604 | 0 | 0 | 0 | 0 | 0 | 0 | 1 |
| THBS1-IT1 | 0 | 0 | 0 | 0 | 0 | 0 | 1 |
| DNAJA3 | 0 | 0 | 0 | 0 | 0 | 0 | 1 |
| UBE2Q2 | 0 | 0 | 0 | 0 | 0 | 0 | 1 |
| CD320 | 0 | 0 | 0 | 0 | 0 | 0 | 1 |
| HIBADH | 0 | 0 | 0 | 0 | 0 | 0 | 1 |
| RTTN | 0 | 0 | 0 | 0 | 0 | 0 | 1 |
| WWP1 | 0 | 0 | 0 | 0 | 0 | 0 | 1 |
| INSC | 0 | 0 | 0 | 0 | 0 | 0 | 1 |
| RASD1 | 0 | 0 | 0 | 0 | 0 | 0 | 1 |
| DOK2 | 0 | 0 | 0 | 0 | 0 | 0 | 1 |
| MPRIPP1 | 0 | 0 | 0 | 0 | 0 | 0 | 1 |
| CCL21 | 0 | 0 | 0 | 0 | 0 | 0 | 1 |
| ENSG00000270091 | 0 | 0 | 0 | 0 | 0 | 0 | 1 |
| ABCC4 | 0 | 0 | 0 | 0 | 0 | 0 | 1 |
| ANXA2P2 | 0 | 0 | 0 | 0 | 0 | 0 | 1 |
| TRMT11 | 0 | 0 | 0 | 0 | 0 | 0 | 1 |
| CCDC17 | 0 | 0 | 0 | 0 | 0 | 0 | 1 |
| RAD52 | 0 | 0 | 0 | 0 | 0 | 0 | 1 |
| KCNS3 | 0 | 0 | 0 | 0 | 0 | 0 | 1 |
| TMEM273 | 0 | 0 | 0 | 0 | 0 | 0 | 1 |
| ENSG00000275850 | 0 | 0 | 0 | 0 | 0 | 0 | 1 |
| EMP1 | 0 | 0 | 0 | 0 | 0 | 0 | 1 |
| RCC2 | 0 | 0 | 0 | 0 | 0 | 0 | 1 |
| ZNF670 | 0 | 0 | 0 | 0 | 0 | 0 | 1 |
| TAS2R63P | 0 | 0 | 0 | 0 | 0 | 0 | 1 |
| HMGA2 | 0 | 0 | 0 | 0 | 0 | 0 | 1 |
| SCARA3 | 0 | 0 | 0 | 0 | 0 | 0 | 1 |
| ASPG | 0 | 0 | 0 | 0 | 0 | 0 | 1 |
| RAB6B | 0 | 0 | 0 | 0 | 0 | 0 | 1 |
| ENSG00000274038 | 0 | 0 | 0 | 0 | 0 | 0 | 1 |
| SLCO2B1 | 0 | 0 | 0 | 0 | 0 | 0 | 1 |
| PHETA2 | 0 | 0 | 0 | 0 | 0 | 0 | 1 |
| ARID3A | 0 | 0 | 0 | 0 | 0 | 0 | 1 |
| MDH2 | 0 | 0 | 0 | 0 | 0 | 0 | 1 |
| RPL7AP42 | 0 | 0 | 0 | 0 | 0 | 0 | 1 |
| SHTN1 | 0 | 0 | 0 | 0 | 0 | 0 | 1 |
| SULT1C4 | 0 | 0 | 0 | 0 | 0 | 0 | 1 |
| RPL7P9 | 0 | 0 | 0 | 0 | 0 | 0 | 1 |
| ENSG00000286689 | 0 | 0 | 0 | 0 | 0 | 0 | 1 |
| TBC1D30 | 0 | 0 | 0 | 0 | 0 | 0 | 1 |
| GIT1 | 0 | 0 | 0 | 0 | 0 | 0 | 1 |
| USF1P1 | 0 | 0 | 0 | 0 | 0 | 0 | 1 |
| ENSG00000288586 | 0 | 0 | 0 | 0 | 0 | 0 | 1 |
| TMEM26 | 0 | 0 | 0 | 0 | 0 | 0 | 1 |
| LARGE-IT1 | 0 | 0 | 0 | 0 | 0 | 0 | 1 |
| ENSG00000251314 | 0 | 0 | 0 | 0 | 0 | 0 | 1 |
| ENSG00000261056 | 0 | 0 | 0 | 0 | 0 | 0 | 1 |
| RN7SKP110 | 0 | 0 | 0 | 0 | 0 | 0 | 1 |
| ENSG00000273328 | 0 | 0 | 0 | 0 | 0 | 0 | 1 |
| VWA8 | 0 | 0 | 0 | 0 | 0 | 0 | 1 |
| ENSG00000279453 | 0 | 0 | 0 | 0 | 0 | 0 | 1 |
| LINC01232 | 0 | 0 | 0 | 0 | 0 | 0 | 1 |
| WFIKKN1 | 0 | 0 | 0 | 0 | 0 | 0 | 1 |
| ZNF703 | 0 | 0 | 0 | 0 | 0 | 0 | 1 |
| DNM1P47 | 0 | 0 | 0 | 0 | 0 | 0 | 1 |
| TCIM | 0 | 0 | 0 | 0 | 0 | 0 | 1 |
| GPRC5A | 0 | 0 | 0 | 0 | 0 | 0 | 1 |
| NPNT | 0 | 0 | 0 | 0 | 0 | 0 | 1 |
| SIRPA | 0 | 0 | 0 | 0 | 0 | 0 | 1 |
| ADAMTSL4-AS2 | 0 | 0 | 0 | 0 | 0 | 0 | 1 |
| CNPY4 | 0 | 0 | 0 | 0 | 0 | 0 | 1 |
| NOVA2 | 0 | 0 | 0 | 0 | 0 | 0 | 1 |
| ZNF711 | 0 | 0 | 0 | 0 | 0 | 0 | 1 |
| MXD1 | 0 | 0 | 0 | 0 | 0 | 0 | 1 |
| CATSPERG | 0 | 0 | 0 | 0 | 0 | 0 | 1 |
| ENSG00000274776 | 0 | 0 | 0 | 0 | 0 | 0 | 1 |
| NDUFB2-AS1 | 0 | 0 | 0 | 0 | 0 | 0 | 1 |
| PIP5K1B | 0 | 0 | 0 | 0 | 0 | 0 | 1 |
| BHLHE41 | 0 | 0 | 0 | 0 | 0 | 0 | 1 |
| ZFP82 | 0 | 0 | 0 | 0 | 0 | 0 | 1 |
| PEX3 | 0 | 0 | 0 | 0 | 0 | 0 | 1 |
| ENSG00000261888 | 0 | 0 | 0 | 0 | 0 | 0 | 1 |
| ENSG00000272505 | 0 | 0 | 0 | 0 | 0 | 0 | 1 |
| MMUT | 0 | 0 | 0 | 0 | 0 | 0 | 1 |
| ZNF579 | 0 | 0 | 0 | 0 | 0 | 0 | 1 |
| SCIN | 0 | 0 | 0 | 0 | 0 | 0 | 1 |
| ZNF252P-AS1 | 0 | 0 | 0 | 0 | 0 | 0 | 1 |
| SMG1P7 | 0 | 0 | 0 | 0 | 0 | 0 | 1 |
| ANXA2 | 0 | 0 | 0 | 0 | 0 | 0 | 1 |
| CACTIN | 0 | 0 | 0 | 0 | 0 | 0 | 1 |
| NBR2 | 0 | 0 | 0 | 0 | 0 | 0 | 1 |
| CRB3 | 0 | 0 | 0 | 0 | 0 | 0 | 1 |
| AP1G2-AS1 | 0 | 0 | 0 | 0 | 0 | 0 | 1 |
| B3GNT7 | 0 | 0 | 0 | 0 | 0 | 0 | 1 |
| NKD2 | 0 | 0 | 0 | 0 | 0 | 0 | 1 |
| GNA12 | 0 | 0 | 0 | 0 | 0 | 0 | 1 |
| PDGFB | 0 | 0 | 0 | 0 | 0 | 0 | 1 |
| ST7-OT4 | 0 | 0 | 0 | 0 | 0 | 0 | 1 |
| DBF4 | 0 | 0 | 0 | 0 | 0 | 0 | 1 |
| FBLN2 | 0 | 0 | 0 | 0 | 0 | 0 | 1 |
| ORMDL3 | 0 | 0 | 0 | 0 | 0 | 0 | 1 |
| ENSG00000253372 | 0 | 0 | 0 | 0 | 0 | 0 | 1 |
| ECHS1 | 0 | 0 | 0 | 0 | 0 | 0 | 1 |
| PRR4 | 0 | 0 | 0 | 0 | 0 | 0 | 1 |
| TNFSF14 | 0 | 0 | 0 | 0 | 0 | 0 | 1 |
| GSTP1 | 0 | 0 | 0 | 0 | 0 | 0 | 1 |
| ENSG00000272668 | 0 | 0 | 0 | 0 | 0 | 0 | 1 |
| ENSG00000287925 | 0 | 0 | 0 | 0 | 0 | 0 | 1 |
| BAIAP2-DT | 0 | 0 | 0 | 0 | 0 | 0 | 1 |
| ATP5F1C | 0 | 0 | 0 | 0 | 0 | 0 | 1 |
| USP32P3 | 0 | 0 | 0 | 0 | 0 | 0 | 1 |
| TNKS1BP1 | 0 | 0 | 0 | 0 | 0 | 0 | 1 |
| KMT5C | 0 | 0 | 0 | 0 | 0 | 0 | 1 |
| FAM135B | 0 | 0 | 0 | 0 | 0 | 0 | 1 |
| NECAB3 | 0 | 0 | 0 | 0 | 0 | 0 | 1 |
| MAFG-DT | 0 | 0 | 0 | 0 | 0 | 0 | 1 |
| HDHD5 | 0 | 0 | 0 | 0 | 0 | 0 | 1 |
| ST3GAL6-AS1 | 0 | 0 | 0 | 0 | 0 | 0 | 1 |
| ENSG00000288538 | 0 | 0 | 0 | 0 | 0 | 0 | 1 |
| ENSG00000228280 | 0 | 0 | 0 | 0 | 0 | 0 | 1 |
| TADA1 | 0 | 0 | 0 | 0 | 0 | 0 | 1 |
| ZNF385B | 0 | 0 | 0 | 0 | 0 | 0 | 1 |
| ENSG00000277687 | 0 | 0 | 0 | 0 | 0 | 0 | 1 |
| SNX21 | 0 | 0 | 0 | 0 | 0 | 0 | 1 |
| ENSG00000224945 | 0 | 0 | 0 | 0 | 0 | 0 | 1 |
| ENSG00000234902 | 0 | 0 | 0 | 0 | 0 | 0 | 1 |
| KLF5 | 0 | 0 | 0 | 0 | 0 | 0 | 1 |
| MB21D2 | 0 | 0 | 0 | 0 | 0 | 0 | 1 |
| PFDN2 | 0 | 0 | 0 | 0 | 0 | 0 | 1 |
| RN7SKP56 | 0 | 0 | 0 | 0 | 0 | 0 | 1 |
| ENSG00000285744 | 0 | 0 | 0 | 0 | 0 | 0 | 1 |
| TMEM254 | 0 | 0 | 0 | 0 | 0 | 0 | 1 |
| FAM151B | 0 | 0 | 0 | 0 | 0 | 0 | 1 |
| ENSG00000279026 | 0 | 0 | 0 | 0 | 0 | 0 | 1 |
| ENSG00000259767 | 0 | 0 | 0 | 0 | 0 | 0 | 1 |
| RNF213-AS1 | 0 | 0 | 0 | 0 | 0 | 0 | 1 |
| ENSG00000281469 | 0 | 0 | 0 | 0 | 0 | 0 | 1 |
| FAM78B | 0 | 0 | 0 | 0 | 0 | 0 | 1 |
| LGR4 | 0 | 0 | 0 | 0 | 0 | 0 | 1 |
| MAK | 0 | 0 | 0 | 0 | 0 | 0 | 1 |
| ENSG00000255872 | 0 | 0 | 0 | 0 | 0 | 0 | 1 |
| ENSG00000280046 | 0 | 0 | 0 | 0 | 0 | 0 | 1 |
| ZNF865 | 0 | 0 | 0 | 0 | 0 | 0 | 1 |
| ATOH8 | 0 | 0 | 0 | 0 | 0 | 0 | 1 |
| FOXQ1 | 0 | 0 | 0 | 0 | 0 | 0 | 1 |
| ENSG00000270061 | 0 | 0 | 0 | 0 | 0 | 0 | 1 |
| SCRIB | 0 | 0 | 0 | 0 | 0 | 0 | 1 |
| ANKRD33B | 0 | 0 | 0 | 0 | 0 | 0 | 1 |
| LPL | 0 | 0 | 0 | 0 | 0 | 0 | 1 |
| SLC45A4 | 0 | 0 | 0 | 0 | 0 | 0 | 1 |
| LRCOL1 | 0 | 0 | 0 | 0 | 0 | 0 | 1 |
| CPT2 | 0 | 0 | 0 | 0 | 0 | 0 | 1 |
| MYCBP2-AS2 | 0 | 0 | 0 | 0 | 0 | 0 | 1 |
| EFS | 0 | 0 | 0 | 0 | 0 | 0 | 1 |
| ENSG00000275120 | 0 | 0 | 0 | 0 | 0 | 0 | 1 |
| ZNF331 | 0 | 0 | 0 | 0 | 0 | 0 | 1 |
| FLVCR2 | 0 | 0 | 0 | 0 | 0 | 0 | 1 |
| RPLP2 | 0 | 0 | 0 | 0 | 0 | 0 | 1 |
| BMP5 | 0 | 0 | 0 | 0 | 0 | 0 | 1 |
| B4GALNT3 | 0 | 0 | 0 | 0 | 0 | 0 | 1 |
| PROCR | 0 | 0 | 0 | 0 | 0 | 0 | 1 |
| NIPAL1 | 0 | 0 | 0 | 0 | 0 | 0 | 1 |
| PAQR7 | 0 | 0 | 0 | 0 | 0 | 0 | 1 |
| ENSG00000260495 | 0 | 0 | 0 | 0 | 0 | 0 | 1 |
| ENSG00000273133 | 0 | 0 | 0 | 0 | 0 | 0 | 1 |
| TG | 0 | 0 | 0 | 0 | 0 | 0 | 1 |
| ENSG00000277595 | 0 | 0 | 0 | 0 | 0 | 0 | 1 |
| KIAA1614 | 0 | 0 | 0 | 0 | 0 | 0 | 1 |
| CIART | 0 | 0 | 0 | 0 | 0 | 0 | 1 |
| CCDC180 | 0 | 0 | 0 | 0 | 0 | 0 | 1 |
| GOLGA2P10 | 0 | 0 | 0 | 0 | 0 | 0 | 1 |
| KCNT2 | 0 | 0 | 0 | 0 | 0 | 0 | 1 |
| ZNF707 | 0 | 0 | 0 | 0 | 0 | 0 | 1 |
| JOSD2 | 0 | 0 | 0 | 0 | 0 | 0 | 1 |
| ENSG00000233674 | 0 | 0 | 0 | 0 | 0 | 0 | 1 |
| SH3BGRL | 0 | 0 | 0 | 0 | 0 | 0 | 1 |
| GPSM1 | 0 | 0 | 0 | 0 | 0 | 0 | 1 |
| MRPL54 | 0 | 0 | 0 | 0 | 0 | 0 | 1 |
| CNN1 | 0 | 0 | 0 | 0 | 0 | 0 | 1 |
| MCRIP1 | 0 | 0 | 0 | 0 | 0 | 0 | 1 |
| SMAD6 | 0 | 0 | 0 | 0 | 0 | 0 | 1 |
| COX6A1 | 0 | 0 | 0 | 0 | 0 | 0 | 1 |
| IFI27L2 | 0 | 0 | 0 | 0 | 0 | 0 | 1 |
| ENSG00000225721 | 0 | 0 | 0 | 0 | 0 | 0 | 1 |
| SALL2 | 0 | 0 | 0 | 0 | 0 | 0 | 1 |
| TMED1 | 0 | 0 | 0 | 0 | 0 | 0 | 1 |
| SCML2 | 0 | 0 | 0 | 0 | 0 | 0 | 1 |
| ENSG00000279811 | 0 | 0 | 0 | 0 | 0 | 0 | 1 |
| MARCHF9 | 0 | 0 | 0 | 0 | 0 | 0 | 1 |
| ACADS | 0 | 0 | 0 | 0 | 0 | 0 | 1 |
| SUCLG2-DT | 0 | 0 | 0 | 0 | 0 | 0 | 1 |
| MOK | 0 | 0 | 0 | 0 | 0 | 0 | 1 |
| ENSG00000275236 | 0 | 0 | 0 | 0 | 0 | 0 | 1 |
| UBXN8 | 0 | 0 | 0 | 0 | 0 | 0 | 1 |
| ENSG00000278367 | 0 | 0 | 0 | 0 | 0 | 0 | 1 |
| PRDX3 | 0 | 0 | 0 | 0 | 0 | 0 | 1 |
| INTS6-AS1 | 0 | 0 | 0 | 0 | 0 | 0 | 1 |
| ENSG00000261537 | 0 | 0 | 0 | 0 | 0 | 0 | 1 |
| SDCBP2 | 0 | 0 | 0 | 0 | 0 | 0 | 1 |
| ENSG00000287562 | 0 | 0 | 0 | 0 | 0 | 0 | 1 |
| STAP2 | 0 | 0 | 0 | 0 | 0 | 0 | 1 |
| ENSG00000273381 | 0 | 0 | 0 | 0 | 0 | 0 | 1 |
| EFEMP1 | 0 | 0 | 0 | 0 | 0 | 0 | 1 |
| ENSG00000229955 | 0 | 0 | 0 | 0 | 0 | 0 | 1 |
| RTN2 | 0 | 0 | 0 | 0 | 0 | 0 | 1 |
| KLHL32 | 0 | 0 | 0 | 0 | 0 | 0 | 1 |
| TWF2 | 0 | 0 | 0 | 0 | 0 | 0 | 1 |
| TIFA | 0 | 0 | 0 | 0 | 0 | 0 | 1 |
| APOE | 0 | 0 | 0 | 0 | 0 | 0 | 1 |
| ETFB | 0 | 0 | 0 | 0 | 0 | 0 | 1 |
| ENSG00000223916 | 0 | 0 | 0 | 0 | 0 | 0 | 1 |
| FOXF1 | 0 | 0 | 0 | 0 | 0 | 0 | 1 |
| RDH10 | 0 | 0 | 0 | 0 | 0 | 0 | 1 |
| IGSF6 | 0 | 0 | 0 | 0 | 0 | 0 | 1 |
| PLCD1 | 0 | 0 | 0 | 0 | 0 | 0 | 1 |
| ENSG00000250519 | 0 | 0 | 0 | 0 | 0 | 0 | 1 |
| ENSG00000284292 | 0 | 0 | 0 | 0 | 0 | 0 | 1 |
| NPIPP1 | 0 | 0 | 0 | 0 | 0 | 0 | 1 |
| AQP7 | 0 | 0 | 0 | 0 | 0 | 0 | 1 |
| CEP57 | 0 | 0 | 0 | 0 | 0 | 0 | 1 |
| NKILA | 0 | 0 | 0 | 0 | 0 | 0 | 1 |
| RTN4RL1 | 0 | 0 | 0 | 0 | 0 | 0 | 1 |
| OSTC | 0 | 0 | 0 | 0 | 0 | 0 | 1 |
| ENSG00000272379 | 0 | 0 | 0 | 0 | 0 | 0 | 1 |
| AGPAT5 | 0 | 0 | 0 | 0 | 0 | 0 | 1 |
| NSDHL | 0 | 0 | 0 | 0 | 0 | 0 | 1 |
| ENSG00000270804 | 0 | 0 | 0 | 0 | 0 | 0 | 1 |
| UST | 0 | 0 | 0 | 0 | 0 | 0 | 1 |
| IPMK | 0 | 0 | 0 | 0 | 0 | 0 | 1 |
| ENSG00000286817 | 0 | 0 | 0 | 0 | 0 | 0 | 1 |
| ENSG00000224593 | 0 | 0 | 0 | 0 | 0 | 0 | 1 |
| FBLN1 | 0 | 0 | 0 | 0 | 0 | 0 | 1 |
| MRPS35 | 0 | 0 | 0 | 0 | 0 | 0 | 1 |
| ENSG00000240710 | 0 | 0 | 0 | 0 | 0 | 0 | 1 |
| IL17RD | 0 | 0 | 0 | 0 | 0 | 0 | 1 |
| ENSG00000261451 | 0 | 0 | 0 | 0 | 0 | 0 | 1 |
| NUDCP1 | 0 | 0 | 0 | 0 | 0 | 0 | 1 |
| NCK2 | 0 | 0 | 0 | 0 | 0 | 0 | 1 |
| C21orf58 | 0 | 0 | 0 | 0 | 0 | 0 | 1 |
| GSTZ1 | 0 | 0 | 0 | 0 | 0 | 0 | 1 |
| CCDC88B | 0 | 0 | 0 | 0 | 0 | 0 | 1 |
| CCL19 | 0 | 0 | 0 | 0 | 0 | 0 | 1 |
| HILPDA | 0 | 0 | 0 | 0 | 0 | 0 | 1 |
| DKK2 | 0 | 0 | 0 | 0 | 0 | 0 | 1 |
| DNMT1 | 0 | 0 | 0 | 0 | 0 | 0 | 1 |
| LMOD3 | 0 | 0 | 0 | 0 | 0 | 0 | 1 |
| EEPD1 | 0 | 0 | 0 | 0 | 0 | 0 | 1 |
| ENSG00000279059 | 0 | 0 | 0 | 0 | 0 | 0 | 1 |
| DDX10P1 | 0 | 0 | 0 | 0 | 0 | 0 | 1 |
| HIC1 | 0 | 0 | 0 | 0 | 0 | 0 | 1 |
| ENSG00000286451 | 0 | 0 | 0 | 0 | 0 | 0 | 1 |
| RGS14 | 0 | 0 | 0 | 0 | 0 | 0 | 1 |
| CYC1 | 0 | 0 | 0 | 0 | 0 | 0 | 1 |
| RABGAP1L-DT | 0 | 0 | 0 | 0 | 0 | 0 | 1 |
| DERA | 0 | 0 | 0 | 0 | 0 | 0 | 1 |
| ENSG00000266498 | 0 | 0 | 0 | 0 | 0 | 0 | 1 |
| ENSG00000237094 | 0 | 0 | 0 | 0 | 0 | 0 | 1 |
| FBXL19 | 0 | 0 | 0 | 0 | 0 | 0 | 1 |
| TRAPPC4 | 0 | 0 | 0 | 0 | 0 | 0 | 1 |
| PRR15L | 0 | 0 | 0 | 0 | 0 | 0 | 1 |
| AP4B1 | 0 | 0 | 0 | 0 | 0 | 0 | 1 |
| ZEB2-AS1 | 0 | 0 | 0 | 0 | 0 | 0 | 1 |
| LRRC8D | 0 | 0 | 0 | 0 | 0 | 0 | 1 |
| ELAPOR2 | 0 | 0 | 0 | 0 | 0 | 0 | 1 |
| PCF11-AS1 | 0 | 0 | 0 | 0 | 0 | 0 | 1 |
| RPL30P13 | 0 | 0 | 0 | 0 | 0 | 0 | 1 |
| ENSG00000274297 | 0 | 0 | 0 | 0 | 0 | 0 | 1 |
| ENSG00000272033 | 0 | 0 | 0 | 0 | 0 | 0 | 1 |
| MALL | 0 | 0 | 0 | 0 | 0 | 0 | 1 |
| GRB7 | 0 | 0 | 0 | 0 | 0 | 0 | 1 |
| BTNL9 | 0 | 0 | 0 | 0 | 0 | 0 | 1 |
| PCDHB4 | 0 | 0 | 0 | 0 | 0 | 0 | 1 |
| BCL2L11 | 0 | 0 | 0 | 0 | 0 | 0 | 1 |
| ADGRG6 | 0 | 0 | 0 | 0 | 0 | 0 | 1 |
| RAI2 | 0 | 0 | 0 | 0 | 0 | 0 | 1 |
| KYAT3 | 0 | 0 | 0 | 0 | 0 | 0 | 1 |
| DIP2A-IT1 | 0 | 0 | 0 | 0 | 0 | 0 | 1 |
| SYN1 | 0 | 0 | 0 | 0 | 0 | 0 | 1 |
| GALNT3 | 0 | 0 | 0 | 0 | 0 | 0 | 1 |
| PTPRU | 0 | 0 | 0 | 0 | 0 | 0 | 1 |
| ENSG00000229618 | 0 | 0 | 0 | 0 | 0 | 0 | 1 |
| ZNF524 | 0 | 0 | 0 | 0 | 0 | 0 | 1 |
| YTHDF1P1 | 0 | 0 | 0 | 0 | 0 | 0 | 1 |
| NANOGP8 | 0 | 0 | 0 | 0 | 0 | 0 | 1 |
| LINC02918 | 0 | 0 | 0 | 0 | 0 | 0 | 1 |
| ENSG00000273151 | 0 | 0 | 0 | 0 | 0 | 0 | 1 |
| PRSS36 | 0 | 0 | 0 | 0 | 0 | 0 | 1 |
| ARL4AP5 | 0 | 0 | 0 | 0 | 0 | 0 | 1 |
| ENSG00000258311 | 0 | 0 | 0 | 0 | 0 | 0 | 1 |
| NT5E | 0 | 0 | 0 | 0 | 0 | 0 | 1 |
| AP1M2 | 0 | 0 | 0 | 0 | 0 | 0 | 1 |
| ANGPTL1 | 0 | 0 | 0 | 0 | 0 | 0 | 1 |
| DPYSL3 | 0 | 0 | 0 | 0 | 0 | 0 | 1 |
| FUOM | 0 | 0 | 0 | 0 | 0 | 0 | 1 |
| PKNOX2 | 0 | 0 | 0 | 0 | 0 | 0 | 1 |
| NCAPGP1 | 0 | 0 | 0 | 0 | 0 | 0 | 1 |
| PRSS42P | 0 | 0 | 0 | 0 | 0 | 0 | 1 |
| ENSG00000250899 | 0 | 0 | 0 | 0 | 0 | 0 | 1 |
| INKA2 | 0 | 0 | 0 | 0 | 0 | 0 | 1 |
| GIMAP8 | 0 | 0 | 0 | 0 | 0 | 0 | 1 |
| FEZ1 | 0 | 0 | 0 | 0 | 0 | 0 | 1 |
| ENSG00000260645 | 0 | 0 | 0 | 0 | 0 | 0 | 1 |
| ATN1 | 0 | 0 | 0 | 0 | 0 | 0 | 1 |
| HMOX1 | 0 | 0 | 0 | 0 | 0 | 0 | 1 |
| MMACHC | 0 | 0 | 0 | 0 | 0 | 0 | 1 |
| DZIP1L | 0 | 0 | 0 | 0 | 0 | 0 | 1 |
| ENSG00000256361 | 0 | 0 | 0 | 0 | 0 | 0 | 1 |
| INPP5J | 0 | 0 | 0 | 0 | 0 | 0 | 1 |
| CHMP4A | 0 | 0 | 0 | 0 | 0 | 0 | 1 |
| MRPL39 | 0 | 0 | 0 | 0 | 0 | 0 | 1 |
| FSBP | 0 | 0 | 0 | 0 | 0 | 0 | 1 |
| SLC28A3 | 0 | 0 | 0 | 0 | 0 | 0 | 1 |
| ENSG00000233178 | 0 | 0 | 0 | 0 | 0 | 0 | 1 |
| SOWAHCP2 | 0 | 0 | 0 | 0 | 0 | 0 | 1 |
| FRMD5 | 0 | 0 | 0 | 0 | 0 | 0 | 1 |
| FBXW9 | 0 | 0 | 0 | 0 | 0 | 0 | 1 |
| C1QTNF3 | 0 | 0 | 0 | 0 | 0 | 0 | 1 |
| NOTCH4 | 0 | 0 | 0 | 0 | 0 | 0 | 1 |
| WHAMMP4 | 0 | 0 | 0 | 0 | 0 | 0 | 1 |
| IGHV2-5 | 0 | 0 | 0 | 0 | 0 | 0 | 1 |
| BMP4 | 0 | 0 | 0 | 0 | 0 | 0 | 1 |
| PARP10 | 0 | 0 | 0 | 0 | 0 | 0 | 1 |
| B3GAT1 | 0 | 0 | 0 | 0 | 0 | 0 | 1 |
| RPL7 | 0 | 0 | 0 | 0 | 0 | 0 | 1 |
| VWA7 | 0 | 0 | 0 | 0 | 0 | 0 | 1 |
| S100A6 | 0 | 0 | 0 | 0 | 0 | 0 | 1 |
| ENSG00000270956 | 0 | 0 | 0 | 0 | 0 | 0 | 1 |
| MSANTD1 | 0 | 0 | 0 | 0 | 0 | 0 | 1 |
| MECOM | 0 | 0 | 0 | 0 | 0 | 0 | 1 |
| PCDHB3 | 0 | 0 | 0 | 0 | 0 | 0 | 1 |
| TCEAL9 | 0 | 0 | 0 | 0 | 0 | 0 | 1 |
| CPNE5 | 0 | 0 | 0 | 0 | 0 | 0 | 1 |
| CARS1 | 0 | 0 | 0 | 0 | 0 | 0 | 1 |
| LINC02482 | 0 | 0 | 0 | 0 | 0 | 0 | 1 |
| SLC25A36 | 0 | 0 | 0 | 0 | 0 | 0 | 1 |
| PPM1L | 0 | 0 | 0 | 0 | 0 | 0 | 1 |
| LGALS3 | 0 | 0 | 0 | 0 | 0 | 0 | 1 |
| ENSG00000277152 | 0 | 0 | 0 | 0 | 0 | 0 | 1 |
| RPS6P25 | 0 | 0 | 0 | 0 | 0 | 0 | 1 |
| ENSG00000272465 | 0 | 0 | 0 | 0 | 0 | 0 | 1 |
| LEPR | 0 | 0 | 0 | 0 | 0 | 0 | 1 |
| CACNA2D2 | 0 | 0 | 0 | 0 | 0 | 0 | 1 |
| ENSG00000265625 | 0 | 0 | 0 | 0 | 0 | 0 | 1 |
| RENBP | 0 | 0 | 0 | 0 | 0 | 0 | 1 |
| ENSG00000260816 | 0 | 0 | 0 | 0 | 0 | 0 | 1 |
| ENSG00000271009 | 0 | 0 | 0 | 0 | 0 | 0 | 1 |
| ENSG00000261499 | 0 | 0 | 0 | 0 | 0 | 0 | 1 |
| MTND4P14 | 0 | 0 | 0 | 0 | 0 | 0 | 1 |
| ATP13A4 | 0 | 0 | 0 | 0 | 0 | 0 | 1 |
| UQCRQ | 0 | 0 | 0 | 0 | 0 | 0 | 1 |
| HOTAIRM1 | 0 | 0 | 0 | 0 | 0 | 0 | 1 |
| ETS2 | 0 | 0 | 0 | 0 | 0 | 0 | 1 |
| ENSG00000259915 | 0 | 0 | 0 | 0 | 0 | 0 | 1 |
| MT-TW | 0 | 0 | 0 | 0 | 0 | 0 | 1 |
| ENSG00000273437 | 0 | 0 | 0 | 0 | 0 | 0 | 1 |
| SHLD3 | 0 | 0 | 0 | 0 | 0 | 0 | 1 |
| C16orf54 | 0 | 0 | 0 | 0 | 0 | 0 | 1 |
| GNA13 | 0 | 0 | 0 | 0 | 0 | 0 | 1 |
| ENSG00000260160 | 0 | 0 | 0 | 0 | 0 | 0 | 1 |
| PEX11A | 0 | 0 | 0 | 0 | 0 | 0 | 1 |
| METRN | 0 | 0 | 0 | 0 | 0 | 0 | 1 |
| AK7 | 0 | 0 | 0 | 0 | 0 | 0 | 1 |
| ENSG00000254165 | 0 | 0 | 0 | 0 | 0 | 0 | 1 |
| PPP1R16B | 0 | 0 | 0 | 0 | 0 | 0 | 1 |
| ENSG00000203647 | 0 | 0 | 0 | 0 | 0 | 0 | 1 |
| ARHGEF17 | 0 | 0 | 0 | 0 | 0 | 0 | 1 |
| ENSG00000273448 | 0 | 0 | 0 | 0 | 0 | 0 | 1 |
| ENSG00000232528 | 0 | 0 | 0 | 0 | 0 | 0 | 1 |
| PPIAP2 | 0 | 0 | 0 | 0 | 0 | 0 | 1 |
| ENSG00000273230 | 0 | 0 | 0 | 0 | 0 | 0 | 1 |
| ENSG00000218426 | 0 | 0 | 0 | 0 | 0 | 0 | 1 |
| MEX3B | 0 | 0 | 0 | 0 | 0 | 0 | 1 |
| ITGB7 | 0 | 0 | 0 | 0 | 0 | 0 | 1 |
| BCAT2 | 0 | 0 | 0 | 0 | 0 | 0 | 1 |
| KDM5C-IT1 | 0 | 0 | 0 | 0 | 0 | 0 | 1 |
| ENSG00000273893 | 0 | 0 | 0 | 0 | 0 | 0 | 1 |
| RPL5P30 | 0 | 0 | 0 | 0 | 0 | 0 | 1 |
| CDC14B | 0 | 0 | 0 | 0 | 0 | 0 | 1 |
| ZNHIT2 | 0 | 0 | 0 | 0 | 0 | 0 | 1 |
| LSMEM1 | 0 | 0 | 0 | 0 | 0 | 0 | 1 |
| HYAL1 | 0 | 0 | 0 | 0 | 0 | 0 | 1 |
| CFAP61 | 0 | 0 | 0 | 0 | 0 | 0 | 1 |
| HACD1 | 0 | 0 | 0 | 0 | 0 | 0 | 1 |
| ENSG00000281530 | 0 | 0 | 0 | 0 | 0 | 0 | 1 |
| GRHL2 | 0 | 0 | 0 | 0 | 0 | 0 | 1 |
| KIF3C | 0 | 0 | 0 | 0 | 0 | 0 | 1 |
| ENSG00000228463 | 0 | 0 | 0 | 0 | 0 | 0 | 1 |
| FILIP1 | 0 | 0 | 0 | 0 | 0 | 0 | 1 |
| ZNF747-DT | 0 | 0 | 0 | 0 | 0 | 0 | 1 |
| TWF1 | 0 | 0 | 0 | 0 | 0 | 0 | 1 |
| LNX1 | 0 | 0 | 0 | 0 | 0 | 0 | 1 |
| ATP5MC3 | 0 | 0 | 0 | 0 | 0 | 0 | 1 |
| ARHGEF15 | 0 | 0 | 0 | 0 | 0 | 0 | 1 |
| BMPR1B | 0 | 0 | 0 | 0 | 0 | 0 | 1 |
| GIPC2 | 0 | 0 | 0 | 0 | 0 | 0 | 1 |
| MIR17HG | 0 | 0 | 0 | 0 | 0 | 0 | 1 |
| ATP5PBP1 | 0 | 0 | 0 | 0 | 0 | 0 | 1 |
| MT-ND6 | 0 | 0 | 0 | 0 | 0 | 0 | 1 |
| ACKR3 | 0 | 0 | 0 | 0 | 0 | 0 | 1 |
| DCLRE1A | 0 | 0 | 0 | 0 | 0 | 0 | 1 |
| SQLE | 0 | 0 | 0 | 0 | 0 | 0 | 1 |
| WNT2 | 0 | 0 | 0 | 0 | 0 | 0 | 1 |
| ARG2 | 0 | 0 | 0 | 0 | 0 | 0 | 1 |
| LINC02969 | 0 | 0 | 0 | 0 | 0 | 0 | 1 |
| ENSG00000273792 | 0 | 0 | 0 | 0 | 0 | 0 | 1 |
| PPM1F | 0 | 0 | 0 | 0 | 0 | 0 | 1 |
| ENSG00000272529 | 0 | 0 | 0 | 0 | 0 | 0 | 1 |
| ENSG00000276997 | 0 | 0 | 0 | 0 | 0 | 0 | 1 |
| TPMT | 0 | 0 | 0 | 0 | 0 | 0 | 1 |
| KDM8 | 0 | 0 | 0 | 0 | 0 | 0 | 1 |
| SLC36A1 | 0 | 0 | 0 | 0 | 0 | 0 | 1 |
| RASL12 | 0 | 0 | 0 | 0 | 0 | 0 | 1 |
| IQANK1 | 0 | 0 | 0 | 0 | 0 | 0 | 1 |
| SORCS2 | 0 | 0 | 0 | 0 | 0 | 0 | 1 |
| EEF1E1 | 0 | 0 | 0 | 0 | 0 | 0 | 1 |
| HK1 | 0 | 0 | 0 | 0 | 0 | 0 | 1 |
| GAPDHP1 | 0 | 0 | 0 | 0 | 0 | 0 | 1 |
| RAB13 | 0 | 0 | 0 | 0 | 0 | 0 | 1 |
| GRM8 | 0 | 0 | 0 | 0 | 0 | 0 | 1 |
| RPS23 | 0 | 0 | 0 | 0 | 0 | 0 | 1 |
| ANKRD1 | 0 | 0 | 0 | 0 | 0 | 0 | 1 |
| SLC12A7 | 0 | 0 | 0 | 0 | 0 | 0 | 1 |
| SUGT1P1 | 0 | 0 | 0 | 0 | 0 | 0 | 1 |
| ENSG00000224950 | 0 | 0 | 0 | 0 | 0 | 0 | 1 |
| ENSG00000279348 | 0 | 0 | 0 | 0 | 0 | 0 | 1 |
| RAB3D | 0 | 0 | 0 | 0 | 0 | 0 | 1 |
| C18orf15 | 0 | 0 | 0 | 0 | 0 | 0 | 1 |
| LMOD1 | 0 | 0 | 0 | 0 | 0 | 0 | 1 |
| ADAMTS9 | 0 | 0 | 0 | 0 | 0 | 0 | 1 |
| TRPV2 | 0 | 0 | 0 | 0 | 0 | 0 | 1 |
| TTBK1 | 0 | 0 | 0 | 0 | 0 | 0 | 1 |
| GLCE | 0 | 0 | 0 | 0 | 0 | 0 | 1 |
| GLDC | 0 | 0 | 0 | 0 | 0 | 0 | 1 |
| EPHB3 | 0 | 0 | 0 | 0 | 0 | 0 | 1 |
| ADGRE5 | 0 | 0 | 0 | 0 | 0 | 0 | 1 |
| ITGA11 | 0 | 0 | 0 | 0 | 0 | 0 | 1 |
| ASPSCR1 | 0 | 0 | 0 | 0 | 0 | 0 | 1 |
| MAP4K2 | 0 | 0 | 0 | 0 | 0 | 0 | 1 |
| ENSG00000183171 | 0 | 0 | 0 | 0 | 0 | 0 | 1 |
| ART4 | 0 | 0 | 0 | 0 | 0 | 0 | 1 |
| ENSG00000238039 | 0 | 0 | 0 | 0 | 0 | 0 | 1 |
| SOX4 | 0 | 0 | 0 | 0 | 0 | 0 | 1 |
| B3GAT1-DT | 0 | 0 | 0 | 0 | 0 | 0 | 1 |
| PLN | 0 | 0 | 0 | 0 | 0 | 0 | 1 |
| PCDHAC2 | 0 | 0 | 0 | 0 | 0 | 0 | 1 |
| MAFK | 0 | 0 | 0 | 0 | 0 | 0 | 1 |
| STRIP2 | 0 | 0 | 0 | 0 | 0 | 0 | 1 |
| ENSG00000261582 | 0 | 0 | 0 | 0 | 0 | 0 | 1 |
| H2AC15 | 0 | 0 | 0 | 0 | 0 | 0 | 1 |
| TMPRSS4 | 0 | 0 | 0 | 0 | 0 | 0 | 1 |
| B4GALNT1 | 0 | 0 | 0 | 0 | 0 | 0 | 1 |
| ENSG00000226180 | 0 | 0 | 0 | 0 | 0 | 0 | 1 |
| ABCG2 | 0 | 0 | 0 | 0 | 0 | 0 | 1 |
| TAS2R15P | 0 | 0 | 0 | 0 | 0 | 0 | 1 |
| MAGOH2P | 0 | 0 | 0 | 0 | 0 | 0 | 1 |
| REM2 | 0 | 0 | 0 | 0 | 0 | 0 | 1 |
| KITLG | 0 | 0 | 0 | 0 | 0 | 0 | 1 |
| CXCL14 | 0 | 0 | 0 | 0 | 0 | 0 | 1 |
| PEBP1 | 0 | 0 | 0 | 0 | 0 | 0 | 1 |
| SOD3 | 0 | 0 | 0 | 0 | 0 | 0 | 1 |
| TNFRSF19 | 0 | 0 | 0 | 0 | 0 | 0 | 1 |
| LILRB5 | 0 | 0 | 0 | 0 | 0 | 0 | 1 |
| ADH1B | 0 | 0 | 0 | 0 | 0 | 0 | 1 |
| ENSG00000262558 | 0 | 0 | 0 | 0 | 0 | 0 | 1 |
| N4BP3 | 0 | 0 | 0 | 0 | 0 | 0 | 1 |
| UQCR10 | 0 | 0 | 0 | 0 | 0 | 0 | 1 |
| PHLDB2 | 0 | 0 | 0 | 0 | 0 | 0 | 1 |
| ACOX1 | 0 | 0 | 0 | 0 | 0 | 0 | 1 |
| TARS1 | 0 | 0 | 0 | 0 | 0 | 0 | 1 |
| DEPTOR | 0 | 0 | 0 | 0 | 0 | 0 | 1 |
| KLF8 | 0 | 0 | 0 | 0 | 0 | 0 | 1 |
| ENSG00000268051 | 0 | 0 | 0 | 0 | 0 | 0 | 1 |
| DRAM1 | 0 | 0 | 0 | 0 | 0 | 0 | 1 |
| LINC02193 | 0 | 0 | 0 | 0 | 0 | 0 | 1 |
| TAGAP | 0 | 0 | 0 | 0 | 0 | 0 | 1 |
| MCOLN3 | 0 | 0 | 0 | 0 | 0 | 0 | 1 |
| P3H4 | 0 | 0 | 0 | 0 | 0 | 0 | 1 |
| C11orf54 | 0 | 0 | 0 | 0 | 0 | 0 | 1 |
| CYGB | 0 | 0 | 0 | 0 | 0 | 0 | 1 |
| ENSG00000270140 | 0 | 0 | 0 | 0 | 0 | 0 | 1 |
| ARHGEF3 | 0 | 0 | 0 | 0 | 0 | 0 | 1 |
| ANKDD1B | 0 | 0 | 0 | 0 | 0 | 0 | 1 |
| THOC6 | 0 | 0 | 0 | 0 | 0 | 0 | 1 |
| PPP1R3G | 0 | 0 | 0 | 0 | 0 | 0 | 1 |
| ENSG00000254343 | 0 | 0 | 0 | 0 | 0 | 0 | 1 |
| CLPX | 0 | 0 | 0 | 0 | 0 | 0 | 1 |
| ZNF860 | 0 | 0 | 0 | 0 | 0 | 0 | 1 |
| CERS3-AS1 | 0 | 0 | 0 | 0 | 0 | 0 | 1 |
| RN7SKP30 | 0 | 0 | 0 | 0 | 0 | 0 | 1 |
| CDHR1 | 0 | 0 | 0 | 0 | 0 | 0 | 1 |
| SDHD | 0 | 0 | 0 | 0 | 0 | 0 | 1 |
| TIMP4 | 0 | 0 | 0 | 0 | 0 | 0 | 1 |
| HRC | 0 | 0 | 0 | 0 | 0 | 0 | 1 |
| MARCHF3 | 0 | 0 | 0 | 0 | 0 | 0 | 1 |
| CCDC183 | 0 | 0 | 0 | 0 | 0 | 0 | 1 |
| TMEM70 | 0 | 0 | 0 | 0 | 0 | 0 | 1 |
| IZUMO1 | 0 | 0 | 0 | 0 | 0 | 0 | 1 |
| LTK | 0 | 0 | 0 | 0 | 0 | 0 | 1 |
| ENSG00000267672 | 0 | 0 | 0 | 0 | 0 | 0 | 1 |
| KBTBD11 | 0 | 0 | 0 | 0 | 0 | 0 | 1 |
| CFAP410 | 0 | 0 | 0 | 0 | 0 | 0 | 1 |
| VASP | 0 | 0 | 0 | 0 | 0 | 0 | 1 |
| MIR570 | 0 | 0 | 0 | 0 | 0 | 0 | 1 |
| SLC25A20 | 0 | 0 | 0 | 0 | 0 | 0 | 1 |
| GUCY1A2 | 0 | 0 | 0 | 0 | 0 | 0 | 1 |
| EBP | 0 | 0 | 0 | 0 | 0 | 0 | 1 |
| BPNT2 | 0 | 0 | 0 | 0 | 0 | 0 | 1 |
| REX1BD | 0 | 0 | 0 | 0 | 0 | 0 | 1 |
| CALCB | 0 | 0 | 0 | 0 | 0 | 0 | 1 |
| ENSG00000262652 | 0 | 0 | 0 | 0 | 0 | 0 | 1 |
| ENSG00000248015 | 0 | 0 | 0 | 0 | 0 | 0 | 1 |
| TLR2 | 0 | 0 | 0 | 0 | 0 | 0 | 1 |
| DNAH17 | 0 | 0 | 0 | 0 | 0 | 0 | 1 |
| DCST2 | 0 | 0 | 0 | 0 | 0 | 0 | 1 |
| ENSG00000234389 | 0 | 0 | 0 | 0 | 0 | 0 | 1 |
| TSLP | 0 | 0 | 0 | 0 | 0 | 0 | 1 |
| SYDE1 | 0 | 0 | 0 | 0 | 0 | 0 | 1 |
| ENPP2 | 0 | 0 | 0 | 0 | 0 | 0 | 1 |
| PLCH2 | 0 | 0 | 0 | 0 | 0 | 0 | 1 |
| RNU7-40P | 0 | 0 | 0 | 0 | 0 | 0 | 1 |
| SYT15-AS1 | 0 | 0 | 0 | 0 | 0 | 0 | 1 |
| ENSG00000263120 | 0 | 0 | 0 | 0 | 0 | 0 | 1 |
| FAM83G | 0 | 0 | 0 | 0 | 0 | 0 | 1 |
| STPG3-AS1 | 0 | 0 | 0 | 0 | 0 | 0 | 1 |
| ADI1 | 0 | 0 | 0 | 0 | 0 | 0 | 1 |
| GASK1B-AS1 | 0 | 0 | 0 | 0 | 0 | 0 | 1 |
| ENSG00000274341 | 0 | 0 | 0 | 0 | 0 | 0 | 1 |
| HTR4 | 0 | 0 | 0 | 0 | 0 | 0 | 1 |
| ENSG00000277879 | 0 | 0 | 0 | 0 | 0 | 0 | 1 |
| ENSG00000259605 | 0 | 0 | 0 | 0 | 0 | 0 | 1 |
| MIR7161 | 0 | 0 | 0 | 0 | 0 | 0 | 1 |
| FENDRR | 0 | 0 | 0 | 0 | 0 | 0 | 1 |
| ENSG00000285417 | 0 | 0 | 0 | 0 | 0 | 0 | 1 |
| PDE3A | 0 | 0 | 0 | 0 | 0 | 0 | 1 |
| CD34 | 0 | 0 | 0 | 0 | 0 | 0 | 1 |
| ENSG00000279880 | 0 | 0 | 0 | 0 | 0 | 0 | 1 |
| ENSG00000261635 | 0 | 0 | 0 | 0 | 0 | 0 | 1 |
| ENSG00000279932 | 0 | 0 | 0 | 0 | 0 | 0 | 1 |
| ZNF566-AS1 | 0 | 0 | 0 | 0 | 0 | 0 | 1 |
| LINC01622 | 0 | 0 | 0 | 0 | 0 | 0 | 1 |
| ENSG00000272812 | 0 | 0 | 0 | 0 | 0 | 0 | 1 |
| THBS1 | 0 | 0 | 0 | 0 | 0 | 0 | 1 |
| TAS2R14 | 0 | 0 | 0 | 0 | 0 | 0 | 1 |
| SUCLG1 | 0 | 0 | 0 | 0 | 0 | 0 | 1 |
| ENSG00000250280 | 0 | 0 | 0 | 0 | 0 | 0 | 1 |
| CNIH1 | 0 | 0 | 0 | 0 | 0 | 0 | 1 |
| HDGFL3 | 0 | 0 | 0 | 0 | 0 | 0 | 1 |
| RABGAP1L-IT1 | 0 | 0 | 0 | 0 | 0 | 0 | 1 |
| PAM16 | 0 | 0 | 0 | 0 | 0 | 0 | 1 |
| COQ10A | 0 | 0 | 0 | 0 | 0 | 0 | 1 |
| ENSG00000278668 | 0 | 0 | 0 | 0 | 0 | 0 | 1 |
| ENSG00000285581 | 0 | 0 | 0 | 0 | 0 | 0 | 1 |
| TRABD2B | 0 | 0 | 0 | 0 | 0 | 0 | 1 |
| ZNNT1 | 0 | 0 | 0 | 0 | 0 | 0 | 1 |
| CYP4X1 | 0 | 0 | 0 | 0 | 0 | 0 | 1 |
| CRYAB | 0 | 0 | 0 | 0 | 0 | 0 | 1 |
| HPS5 | 0 | 0 | 0 | 0 | 0 | 0 | 1 |
| ZNF215 | 0 | 0 | 0 | 0 | 0 | 0 | 1 |
| MTND2P2 | 0 | 0 | 0 | 0 | 0 | 0 | 1 |
| PIGAP1 | 0 | 0 | 0 | 0 | 0 | 0 | 1 |
| APLN | 0 | 0 | 0 | 0 | 0 | 0 | 1 |
| ENSG00000250138 | 0 | 0 | 0 | 0 | 0 | 0 | 1 |
| SLC27A1 | 0 | 0 | 0 | 0 | 0 | 0 | 1 |
| ENSG00000248863 | 0 | 0 | 0 | 0 | 0 | 0 | 1 |
| BOP1 | 0 | 0 | 0 | 0 | 0 | 0 | 1 |
| CDNF | 0 | 0 | 0 | 0 | 0 | 0 | 1 |
| IER5 | 0 | 0 | 0 | 0 | 0 | 0 | 1 |
| SLC45A2 | 0 | 0 | 0 | 0 | 0 | 0 | 1 |
| CCL3L1 | 0 | 0 | 0 | 0 | 0 | 0 | 1 |
| MAPK13 | 0 | 0 | 0 | 0 | 0 | 0 | 1 |
| TP53I13 | 0 | 0 | 0 | 0 | 0 | 0 | 1 |
| PDE6B | 0 | 0 | 0 | 0 | 0 | 0 | 1 |
| CD300C | 0 | 0 | 0 | 0 | 0 | 0 | 1 |
| ENSG00000238061 | 0 | 0 | 0 | 0 | 0 | 0 | 1 |
| ENSG00000276248 | 0 | 0 | 0 | 0 | 0 | 0 | 1 |
| ITGB6 | 0 | 0 | 0 | 0 | 0 | 0 | 1 |
| MEX3A | 0 | 0 | 0 | 0 | 0 | 0 | 1 |
| FAM83H | 0 | 0 | 0 | 0 | 0 | 0 | 1 |
| TGFB1 | 0 | 0 | 0 | 0 | 0 | 0 | 1 |
| CTAGE3P | 0 | 0 | 0 | 0 | 0 | 0 | 1 |
| H3C11 | 0 | 0 | 0 | 0 | 0 | 0 | 1 |
| ENSG00000279836 | 0 | 0 | 0 | 0 | 0 | 0 | 1 |
| GJC1 | 0 | 0 | 0 | 0 | 0 | 0 | 1 |
| ENSG00000278236 | 0 | 0 | 0 | 0 | 0 | 0 | 1 |
| PKD1 | 0 | 0 | 0 | 0 | 0 | 0 | 1 |
| SCRN1 | 0 | 0 | 0 | 0 | 0 | 0 | 1 |
| BAIAP3 | 0 | 0 | 0 | 0 | 0 | 0 | 1 |
| ENSG00000279958 | 0 | 0 | 0 | 0 | 0 | 0 | 1 |
| FGGY-DT | 0 | 0 | 0 | 0 | 0 | 0 | 1 |
| MICU3 | 0 | 0 | 0 | 0 | 0 | 0 | 1 |
| DAGLA | 0 | 0 | 0 | 0 | 0 | 0 | 1 |
| CDH5 | 0 | 0 | 0 | 0 | 0 | 0 | 1 |
| CDKL2 | 0 | 0 | 0 | 0 | 0 | 0 | 1 |
| HBA1 | 0 | 0 | 0 | 0 | 0 | 0 | 1 |
| HMGB1P31 | 0 | 0 | 0 | 0 | 0 | 0 | 1 |
| OPLAH | 0 | 0 | 0 | 0 | 0 | 0 | 1 |
| CCL3 | 0 | 0 | 0 | 0 | 0 | 0 | 1 |
| STARD4 | 0 | 0 | 0 | 0 | 0 | 0 | 1 |
| COX6CP17 | 0 | 0 | 0 | 0 | 0 | 0 | 1 |
| ENSG00000200075 | 0 | 0 | 0 | 0 | 0 | 0 | 1 |
| ENSG00000285269 | 0 | 0 | 0 | 0 | 0 | 0 | 1 |
| MGP | 0 | 0 | 0 | 0 | 0 | 0 | 1 |
| U6 | 0 | 0 | 0 | 0 | 0 | 0 | 1 |
| ENSG00000287932 | 0 | 0 | 0 | 0 | 0 | 0 | 1 |
| C17orf97 | 0 | 0 | 0 | 0 | 0 | 0 | 1 |
| ENSG00000280274 | 0 | 0 | 0 | 0 | 0 | 0 | 1 |
| FOLH1 | 0 | 0 | 0 | 0 | 0 | 0 | 1 |
| RGPD3 | 0 | 0 | 0 | 0 | 0 | 0 | 1 |
| GALNT14 | 0 | 0 | 0 | 0 | 0 | 0 | 1 |
| DHX34 | 0 | 0 | 0 | 0 | 0 | 0 | 1 |
| NET1 | 0 | 0 | 0 | 0 | 0 | 0 | 1 |
| ENSG00000166104 | 0 | 0 | 0 | 0 | 0 | 0 | 1 |
| ENSG00000269867 | 0 | 0 | 0 | 0 | 0 | 0 | 1 |
| CYB5D1 | 0 | 0 | 0 | 0 | 0 | 0 | 1 |
| ENSG00000269938 | 0 | 0 | 0 | 0 | 0 | 0 | 1 |
| KCNMB4 | 0 | 0 | 0 | 0 | 0 | 0 | 1 |
| HBA2 | 0 | 0 | 0 | 0 | 0 | 0 | 1 |
| CHAC1 | 0 | 0 | 0 | 0 | 0 | 0 | 1 |
| ESYT3 | 0 | 0 | 0 | 0 | 0 | 0 | 1 |
| PTPRO | 0 | 0 | 0 | 0 | 0 | 0 | 1 |
| XYLT1 | 0 | 0 | 0 | 0 | 0 | 0 | 1 |
| FBXO32 | 0 | 0 | 0 | 0 | 0 | 0 | 1 |
| MT-ND2 | 0 | 0 | 0 | 0 | 0 | 0 | 1 |
| RASA4CP | 0 | 0 | 0 | 0 | 0 | 0 | 1 |
| SLC25A4 | 0 | 0 | 0 | 0 | 0 | 0 | 1 |
| CKAP2L | 0 | 0 | 0 | 0 | 0 | 0 | 1 |
| GJA1 | 0 | 0 | 0 | 0 | 0 | 0 | 1 |
| ENSG00000258732 | 0 | 0 | 0 | 0 | 0 | 0 | 1 |
| AMDHD2 | 0 | 0 | 0 | 0 | 0 | 0 | 1 |
| CHKB-CPT1B | 0 | 0 | 0 | 0 | 0 | 0 | 1 |
| CBR3 | 0 | 0 | 0 | 0 | 0 | 0 | 1 |
| HSPB8 | 0 | 0 | 0 | 0 | 0 | 0 | 1 |
| ABTB1 | 0 | 0 | 0 | 0 | 0 | 0 | 1 |
| MPV17L | 0 | 0 | 0 | 0 | 0 | 0 | 1 |
| PIK3CG | 0 | 0 | 0 | 0 | 0 | 0 | 1 |
| STMN1P1 | 0 | 0 | 0 | 0 | 0 | 0 | 1 |
| PIM3 | 0 | 0 | 0 | 0 | 0 | 0 | 1 |
| ZDHHC1 | 0 | 0 | 0 | 0 | 0 | 0 | 1 |
| RTL8B | 0 | 0 | 0 | 0 | 0 | 0 | 1 |
| RPLP0P9 | 0 | 0 | 0 | 0 | 0 | 0 | 1 |
| CCN3 | 0 | 0 | 0 | 0 | 0 | 0 | 1 |
| TCAP | 0 | 0 | 0 | 0 | 0 | 0 | 1 |
| ENSG00000270605 | 0 | 0 | 0 | 0 | 0 | 0 | 1 |
| PRR5L | 0 | 0 | 0 | 0 | 0 | 0 | 1 |
| RHPN1 | 0 | 0 | 0 | 0 | 0 | 0 | 1 |
| IMPDH1P8 | 0 | 0 | 0 | 0 | 0 | 0 | 1 |
| SPOCK1 | 0 | 0 | 0 | 0 | 0 | 0 | 1 |
| ENSG00000260912 | 0 | 0 | 0 | 0 | 0 | 0 | 1 |
| ENSG00000271141 | 0 | 0 | 0 | 0 | 0 | 0 | 1 |
| IGHG1 | 0 | 0 | 0 | 0 | 0 | 0 | 1 |
| ENSG00000254061 | 0 | 0 | 0 | 0 | 0 | 0 | 1 |
| RAMP1 | 0 | 0 | 0 | 0 | 0 | 0 | 1 |
| SNX29P2 | 0 | 0 | 0 | 0 | 0 | 0 | 1 |
| TIGD5 | 0 | 0 | 0 | 0 | 0 | 0 | 1 |
| LAYN | 0 | 0 | 0 | 0 | 0 | 0 | 1 |
| PRICKLE1 | 0 | 0 | 0 | 0 | 0 | 0 | 1 |
| EXTL3-AS1 | 0 | 0 | 0 | 0 | 0 | 0 | 1 |
| NPDC1 | 0 | 0 | 0 | 0 | 0 | 0 | 1 |
| CFAP91 | 0 | 0 | 0 | 0 | 0 | 0 | 1 |
| ENSG00000227066 | 0 | 0 | 0 | 0 | 0 | 0 | 1 |
| SUDS3P1 | 0 | 0 | 0 | 0 | 0 | 0 | 1 |
| MFSD6 | 0 | 0 | 0 | 0 | 0 | 0 | 1 |
| A4GALT | 0 | 0 | 0 | 0 | 0 | 0 | 1 |
| E2F1 | 0 | 0 | 0 | 0 | 0 | 0 | 1 |
| LINC02970 | 0 | 0 | 0 | 0 | 0 | 0 | 1 |
| EVC | 0 | 0 | 0 | 0 | 0 | 0 | 1 |
| SLIT3 | 0 | 0 | 0 | 0 | 0 | 0 | 1 |
| IL27RA | 0 | 0 | 0 | 0 | 0 | 0 | 1 |
| ENSG00000187904 | 0 | 0 | 0 | 0 | 0 | 0 | 1 |
| ARMC5 | 0 | 0 | 0 | 0 | 0 | 0 | 1 |
| DOK6 | 0 | 0 | 0 | 0 | 0 | 0 | 1 |
| EFNA1 | 0 | 0 | 0 | 0 | 0 | 0 | 1 |
| ENSG00000286912 | 0 | 0 | 0 | 0 | 0 | 0 | 1 |
| PCDHB2 | 0 | 0 | 0 | 0 | 0 | 0 | 1 |
| PCDH12 | 0 | 0 | 0 | 0 | 0 | 0 | 1 |
| ENSG00000267152 | 0 | 0 | 0 | 0 | 0 | 0 | 1 |
| FLT1 | 0 | 0 | 0 | 0 | 0 | 0 | 1 |
| PKN3 | 0 | 0 | 0 | 0 | 0 | 0 | 1 |
| TAGLN | 0 | 0 | 0 | 0 | 0 | 0 | 1 |
| RGPD8 | 0 | 0 | 0 | 0 | 0 | 0 | 1 |
| LAMC2 | 0 | 0 | 0 | 0 | 0 | 0 | 1 |
| MYORG | 0 | 0 | 0 | 0 | 0 | 0 | 1 |
| CELSR3 | 0 | 0 | 0 | 0 | 0 | 0 | 1 |
| LINC01132 | 0 | 0 | 0 | 0 | 0 | 0 | 1 |
| ABCA3 | 0 | 0 | 0 | 0 | 0 | 0 | 1 |
| ENSG00000242299 | 0 | 0 | 0 | 0 | 0 | 0 | 1 |
| RPS28P7 | 0 | 0 | 0 | 0 | 0 | 0 | 1 |
| LRFN4 | 0 | 0 | 0 | 0 | 0 | 0 | 1 |
| CCL14 | 0 | 0 | 0 | 0 | 0 | 0 | 1 |
| STX1A | 0 | 0 | 0 | 0 | 0 | 0 | 1 |
| LINC00240 | 0 | 0 | 0 | 0 | 0 | 0 | 1 |
| ENSG00000250568 | 0 | 0 | 0 | 0 | 0 | 0 | 1 |
| IGLV1-44 | 0 | 0 | 0 | 0 | 0 | 0 | 1 |
| ITGB1BP2 | 0 | 0 | 0 | 0 | 0 | 0 | 1 |
| ACSM3 | 0 | 0 | 0 | 0 | 0 | 0 | 1 |
| ENSG00000269514 | 0 | 0 | 0 | 0 | 0 | 0 | 1 |
| HOPX | 0 | 0 | 0 | 0 | 0 | 0 | 1 |
| ATP2A1 | 0 | 0 | 0 | 0 | 0 | 0 | 1 |
| RN7SL378P | 0 | 0 | 0 | 0 | 0 | 0 | 1 |
| ATP7B | 0 | 0 | 0 | 0 | 0 | 0 | 1 |
| ZNF511 | 0 | 0 | 0 | 0 | 0 | 0 | 1 |
| ENSG00000238966 | 0 | 0 | 0 | 0 | 0 | 0 | 1 |
| HMGB1P41 | 0 | 0 | 0 | 0 | 0 | 0 | 1 |
| CLDN10 | 0 | 0 | 0 | 0 | 0 | 0 | 1 |
| NKIRAS1 | 0 | 0 | 0 | 0 | 0 | 0 | 1 |
| IFIT1 | 0 | 0 | 0 | 0 | 0 | 0 | 1 |
| FGFR4 | 0 | 0 | 0 | 0 | 0 | 0 | 1 |
| STEAP1B | 0 | 0 | 0 | 0 | 0 | 0 | 1 |
| PMS2P6 | 0 | 0 | 0 | 0 | 0 | 0 | 1 |
| NSMCE1-DT | 0 | 0 | 0 | 0 | 0 | 0 | 1 |
| ALOX15P1 | 0 | 0 | 0 | 0 | 0 | 0 | 1 |
| GPR37 | 0 | 0 | 0 | 0 | 0 | 0 | 1 |
| ENSG00000267681 | 0 | 0 | 0 | 0 | 0 | 0 | 1 |
| ENSG00000274322 | 0 | 0 | 0 | 0 | 0 | 0 | 1 |
| BEND5 | 0 | 0 | 0 | 0 | 0 | 0 | 1 |
| SEC61G | 0 | 0 | 0 | 0 | 0 | 0 | 1 |
| KIF14 | 0 | 0 | 0 | 0 | 0 | 0 | 1 |
| ENSG00000287074 | 0 | 0 | 0 | 0 | 0 | 0 | 1 |
| ACAP2-IT1 | 0 | 0 | 0 | 0 | 0 | 0 | 1 |
| TNFAIP8L2 | 0 | 0 | 0 | 0 | 0 | 0 | 1 |
| TMEM238 | 0 | 0 | 0 | 0 | 0 | 0 | 1 |
| UPP1 | 0 | 0 | 0 | 0 | 0 | 0 | 1 |
| ENSG00000279689 | 0 | 0 | 0 | 0 | 0 | 0 | 1 |
| KIF21A | 0 | 0 | 0 | 0 | 0 | 0 | 1 |
| RCSD1 | 0 | 0 | 0 | 0 | 0 | 0 | 1 |
| LINC01948 | 0 | 0 | 0 | 0 | 0 | 0 | 1 |
| GPBAR1 | 0 | 0 | 0 | 0 | 0 | 0 | 1 |
| TEAD4 | 0 | 0 | 0 | 0 | 0 | 0 | 1 |
| RAMP3 | 0 | 0 | 0 | 0 | 0 | 0 | 1 |
| MMP24 | 0 | 0 | 0 | 0 | 0 | 0 | 1 |
| ENSG00000246225 | 0 | 0 | 0 | 0 | 0 | 0 | 1 |
| COLGALT2 | 0 | 0 | 0 | 0 | 0 | 0 | 1 |
| ENSG00000266993 | 0 | 0 | 0 | 0 | 0 | 0 | 1 |
| PLOD1 | 0 | 0 | 0 | 0 | 0 | 0 | 1 |
| GPC4 | 0 | 0 | 0 | 0 | 0 | 0 | 1 |
| ENSG00000285867 | 0 | 0 | 0 | 0 | 0 | 0 | 1 |
| CIT | 0 | 0 | 0 | 0 | 0 | 0 | 1 |
| HEATR4 | 0 | 0 | 0 | 0 | 0 | 0 | 1 |
| SNORD117 | 0 | 0 | 0 | 0 | 0 | 0 | 1 |
| CCN1 | 0 | 0 | 0 | 0 | 0 | 0 | 1 |
| RGS10 | 0 | 0 | 0 | 0 | 0 | 0 | 1 |
| ENSG00000261512 | 0 | 0 | 0 | 0 | 0 | 0 | 1 |
| PROM2 | 0 | 0 | 0 | 0 | 0 | 0 | 1 |
| SRPX | 0 | 0 | 0 | 0 | 0 | 0 | 1 |
| MAP1B | 0 | 0 | 0 | 0 | 0 | 0 | 1 |
| TMC4 | 0 | 0 | 0 | 0 | 0 | 0 | 1 |
| PLXDC2 | 0 | 0 | 0 | 0 | 0 | 0 | 1 |
| THNSL1 | 0 | 0 | 0 | 0 | 0 | 0 | 1 |
| ENSG00000231466 | 0 | 0 | 0 | 0 | 0 | 0 | 1 |
| MIR3609 | 0 | 0 | 0 | 0 | 0 | 0 | 1 |
| ARHGAP30 | 0 | 0 | 0 | 0 | 0 | 0 | 1 |
| FAM107A | 0 | 0 | 0 | 0 | 0 | 0 | 1 |
| GAS1 | 0 | 0 | 0 | 0 | 0 | 0 | 1 |
| ENSG00000273156 | 0 | 0 | 0 | 0 | 0 | 0 | 1 |
| ENSG00000237813 | 0 | 0 | 0 | 0 | 0 | 0 | 1 |
| XACT | 0 | 0 | 0 | 0 | 0 | 0 | 1 |
| DEPP1 | 0 | 0 | 0 | 0 | 0 | 0 | 1 |
| TOB1 | 0 | 0 | 0 | 0 | 0 | 0 | 1 |
| STEAP2 | 0 | 0 | 0 | 0 | 0 | 0 | 1 |
| ENSG00000227992 | 0 | 0 | 0 | 0 | 0 | 0 | 1 |
| RASL10B | 0 | 0 | 0 | 0 | 0 | 0 | 1 |
| ENSG00000254325 | 0 | 0 | 0 | 0 | 0 | 0 | 1 |
| ARHGEF4 | 0 | 0 | 0 | 0 | 0 | 0 | 1 |
| BIRC3 | 0 | 0 | 0 | 0 | 0 | 0 | 1 |
| DOCK7-DT | 0 | 0 | 0 | 0 | 0 | 0 | 1 |
| PABPC1L | 0 | 0 | 0 | 0 | 0 | 0 | 1 |
| TLNRD1 | 0 | 0 | 0 | 0 | 0 | 0 | 1 |
| SPATA2L | 0 | 0 | 0 | 0 | 0 | 0 | 1 |
| HORMAD1 | 0 | 0 | 0 | 0 | 0 | 0 | 1 |
| GNB1L | 0 | 0 | 0 | 0 | 0 | 0 | 1 |
| FAM133CP | 0 | 0 | 0 | 0 | 0 | 0 | 1 |
| LST1 | 0 | 0 | 0 | 0 | 0 | 0 | 1 |
| ADAMTS1 | 0 | 0 | 0 | 0 | 0 | 0 | 1 |
| ANKRD37 | 0 | 0 | 0 | 0 | 0 | 0 | 1 |
| ENSG00000275367 | 0 | 0 | 0 | 0 | 0 | 0 | 1 |
| ARHGEF19 | 0 | 0 | 0 | 0 | 0 | 0 | 1 |
| ENSG00000272638 | 0 | 0 | 0 | 0 | 0 | 0 | 1 |
| ADAMTS14 | 0 | 0 | 0 | 0 | 0 | 0 | 1 |
| ENSG00000274964 | 0 | 0 | 0 | 0 | 0 | 0 | 1 |
| SNORD15B | 0 | 0 | 0 | 0 | 0 | 0 | 1 |
| ZNF385A | 0 | 0 | 0 | 0 | 0 | 0 | 1 |
| CXCL1 | 0 | 0 | 0 | 0 | 0 | 0 | 1 |
| PTPRN2 | 0 | 0 | 0 | 0 | 0 | 0 | 1 |
| LIF | 0 | 0 | 0 | 0 | 0 | 0 | 1 |
| MT-TQ | 0 | 0 | 0 | 0 | 0 | 0 | 1 |
| ENSG00000283415 | 0 | 0 | 0 | 0 | 0 | 0 | 1 |
| RGS5 | 0 | 0 | 0 | 0 | 0 | 0 | 1 |
| ENSG00000260711 | 0 | 0 | 0 | 0 | 0 | 0 | 1 |
| EDIL3 | 0 | 0 | 0 | 0 | 0 | 0 | 1 |
| TMED7 | 0 | 0 | 0 | 0 | 0 | 0 | 1 |
| MUC5B | 0 | 0 | 0 | 0 | 0 | 0 | 1 |
| IGKV1-5 | 0 | 0 | 0 | 0 | 0 | 0 | 1 |
| PNPLA7 | 0 | 0 | 0 | 0 | 0 | 0 | 1 |
| DPEP2 | 0 | 0 | 0 | 0 | 0 | 0 | 1 |
| PYGO1 | 0 | 0 | 0 | 0 | 0 | 0 | 1 |
| ABHD6 | 0 | 0 | 0 | 0 | 0 | 0 | 1 |
| NAV3 | 0 | 0 | 0 | 0 | 0 | 0 | 1 |
| VNN2 | 0 | 0 | 0 | 0 | 0 | 0 | 1 |
| SPESP1 | 0 | 0 | 0 | 0 | 0 | 0 | 1 |
| PIK3R6 | 0 | 0 | 0 | 0 | 0 | 0 | 1 |
| MAN1B1-DT | 0 | 0 | 0 | 0 | 0 | 0 | 1 |
| TPM2 | 0 | 0 | 0 | 0 | 0 | 0 | 1 |
| RIMS3 | 0 | 0 | 0 | 0 | 0 | 0 | 1 |
| DUSP5 | 0 | 0 | 0 | 0 | 0 | 0 | 1 |
| MBD3 | 0 | 0 | 0 | 0 | 0 | 0 | 1 |
| SYTL1 | 0 | 0 | 0 | 0 | 0 | 0 | 1 |
| LOXHD1 | 0 | 0 | 0 | 0 | 0 | 0 | 1 |
| ENSG00000286129 | 0 | 0 | 0 | 0 | 0 | 0 | 1 |
| MINAR1 | 0 | 0 | 0 | 0 | 0 | 0 | 1 |
| GREM2 | 0 | 0 | 0 | 0 | 0 | 0 | 1 |
| NEXN | 0 | 0 | 0 | 0 | 0 | 0 | 1 |
| NMRAL2P | 0 | 0 | 0 | 0 | 0 | 0 | 1 |
| TBC1D10C | 0 | 0 | 0 | 0 | 0 | 0 | 1 |
| ADGRG1 | 0 | 0 | 0 | 0 | 0 | 0 | 1 |
| ATF3 | 0 | 0 | 0 | 0 | 0 | 0 | 1 |
| ID1 | 0 | 0 | 0 | 0 | 0 | 0 | 1 |
| ENSG00000279328 | 0 | 0 | 0 | 0 | 0 | 0 | 1 |
| FHOD1 | 0 | 0 | 0 | 0 | 0 | 0 | 1 |
| AADAT | 0 | 0 | 0 | 0 | 0 | 0 | 1 |
| ENSG00000284602 | 0 | 0 | 0 | 0 | 0 | 0 | 1 |
| SAMD5 | 0 | 0 | 0 | 0 | 0 | 0 | 1 |
| SOX7 | 0 | 0 | 0 | 0 | 0 | 0 | 1 |
| MAPK4 | 0 | 0 | 0 | 0 | 0 | 0 | 1 |
| ENSG00000232334 | 0 | 0 | 0 | 0 | 0 | 0 | 1 |
| SCTR | 0 | 0 | 0 | 0 | 0 | 0 | 1 |
| THRB-IT1 | 0 | 0 | 0 | 0 | 0 | 0 | 1 |
| ADAMTS7 | 0 | 0 | 0 | 0 | 0 | 0 | 1 |
| SPOPL-DT | 0 | 0 | 0 | 0 | 0 | 0 | 1 |
