# Supplementary material for: Potential association between COVID-19 and neurological disorders: analysis of common genes and therapeutics
Source: Front Neurol. 2024 Oct 14;15:1417183. doi: 10.3389/fneur.2024.1417183 (PMC11513677; doi:10.3389/fneur.2024.1417183)
Supplement: Supplementary file 3 [file Table_3.docx]

**Supplementary Table 3**

**Intersection Distribution**

| Genes | IS | COV-IMM | PD | EP | AD | HS | SD |
| --- | --- | --- | --- | --- | --- | --- | --- |
| CD6 | 1 | 0 | 0 | 0 | 0 | 0 | 0 |
| PDK4 | 1 | 0 | 0 | 0 | 0 | 0 | 0 |
| ARG1 | 1 | 0 | 0 | 0 | 0 | 0 | 0 |
| MAL | 1 | 0 | 0 | 0 | 0 | 0 | 1 |
| FAIM3 | 1 | 0 | 0 | 0 | 0 | 0 | 0 |
| IL7R | 1 | 0 | 0 | 0 | 0 | 0 | 0 |
| IQGAP1 | 1 | 0 | 0 | 0 | 0 | 0 | 0 |
| CCR7 | 1 | 0 | 0 | 0 | 0 | 0 | 0 |
| MMP9 | 1 | 0 | 0 | 0 | 0 | 0 | 0 |
| CA4 | 1 | 0 | 0 | 0 | 0 | 0 | 1 |
| ACSL1 | 1 | 0 | 0 | 0 | 0 | 0 | 0 |
| S100A12 | 1 | 0 | 0 | 0 | 0 | 1 | 0 |
| FOLR3 | 1 | 0 | 0 | 0 | 0 | 0 | 0 |
| LY96 | 1 | 0 | 0 | 0 | 0 | 0 | 0 |
| BNIP3L | 1 | 0 | 0 | 0 | 0 | 1 | 0 |
| APOBEC3A | 1 | 0 | 0 | 0 | 0 | 0 | 0 |
| ORM1 | 1 | 0 | 0 | 0 | 0 | 0 | 1 |
| FCGR3B | 1 | 0 | 0 | 0 | 1 | 0 | 0 |
| FTHL3 | 1 | 0 | 0 | 0 | 0 | 0 | 0 |
| FTHL11 | 1 | 0 | 0 | 0 | 0 | 0 | 0 |
| FCGR3A | 1 | 0 | 0 | 0 | 0 | 0 | 0 |
| IL6R | 0 | 1 | 0 | 0 | 0 | 0 | 0 |
| HLA-DQA1 | 0 | 1 | 0 | 0 | 0 | 0 | 1 |
| HLA-DQB1 | 0 | 1 | 0 | 0 | 0 | 0 | 1 |
| ITGB2 | 0 | 1 | 0 | 0 | 0 | 0 | 0 |
| CD19 | 0 | 1 | 1 | 0 | 0 | 0 | 1 |
| CD22 | 0 | 1 | 0 | 0 | 1 | 0 | 0 |
| MS4A1 | 0 | 1 | 1 | 0 | 0 | 0 | 0 |
| CCR5 | 0 | 1 | 0 | 0 | 0 | 0 | 0 |
| ZAP70 | 0 | 1 | 0 | 0 | 0 | 0 | 0 |
| TUBB | 0 | 1 | 0 | 0 | 0 | 0 | 0 |
| PAX5 | 0 | 1 | 0 | 0 | 0 | 0 | 0 |
| POLR1B | 0 | 1 | 0 | 0 | 0 | 0 | 0 |
| TNFRSF13C | 0 | 1 | 0 | 0 | 0 | 0 | 0 |
| ZBTB16 | 0 | 1 | 0 | 0 | 0 | 0 | 0 |
| CD79A | 0 | 1 | 1 | 0 | 0 | 0 | 0 |
| IRF5 | 0 | 1 | 0 | 0 | 0 | 0 | 0 |
| AICDA | 0 | 1 | 0 | 0 | 0 | 0 | 0 |
| IL1RL2 | 0 | 1 | 0 | 0 | 1 | 0 | 0 |
| CX3CR1 | 0 | 1 | 0 | 0 | 0 | 0 | 0 |
| CCR1 | 0 | 1 | 0 | 0 | 0 | 0 | 1 |
| IL12B | 0 | 1 | 0 | 0 | 0 | 0 | 0 |
| IL1RAP | 0 | 1 | 0 | 0 | 0 | 1 | 1 |
| FCER1A | 0 | 1 | 0 | 0 | 0 | 0 | 0 |
| C4BPA | 0 | 1 | 0 | 0 | 0 | 0 | 1 |
| HLA-DRA | 0 | 1 | 0 | 1 | 0 | 0 | 0 |
| LAG3 | 0 | 1 | 0 | 0 | 0 | 0 | 0 |
| C2 | 0 | 1 | 0 | 0 | 0 | 0 | 0 |
| CD1A | 0 | 1 | 0 | 0 | 0 | 0 | 0 |
| NOS2 | 0 | 1 | 0 | 0 | 0 | 0 | 0 |
| EBI3 | 0 | 1 | 0 | 0 | 0 | 0 | 0 |
| STAT1 | 0 | 1 | 0 | 0 | 0 | 0 | 0 |
| PDCD1 | 0 | 1 | 0 | 0 | 0 | 0 | 0 |
| IL4 | 0 | 1 | 0 | 0 | 0 | 0 | 0 |
| BST2 | 0 | 1 | 0 | 0 | 0 | 0 | 0 |
| IFIH1 | 0 | 1 | 0 | 0 | 0 | 0 | 0 |
| GP1BB | 0 | 1 | 0 | 0 | 0 | 0 | 0 |
| IL22RA2 | 0 | 1 | 0 | 0 | 0 | 0 | 0 |
| THY1 | 0 | 1 | 0 | 0 | 0 | 0 | 0 |
| LILRA6 | 0 | 1 | 0 | 0 | 0 | 0 | 0 |
| CCL15 | 0 | 1 | 0 | 0 | 0 | 0 | 0 |
| SERPING1 | 0 | 1 | 0 | 0 | 0 | 0 | 0 |
| KIR_Activating_Subgroup_1 | 0 | 1 | 0 | 0 | 0 | 0 | 0 |
| CCBP2 | 0 | 1 | 0 | 0 | 0 | 0 | 0 |
| GNLY | 0 | 1 | 0 | 0 | 0 | 0 | 0 |
| GBP1 | 0 | 1 | 0 | 0 | 0 | 0 | 0 |
| C8B | 0 | 1 | 0 | 0 | 0 | 0 | 0 |
| SOCS1 | 0 | 1 | 0 | 0 | 0 | 0 | 0 |
| KIR_Activating_Subgroup_2 | 0 | 1 | 0 | 0 | 0 | 0 | 0 |
| IL18RAP | 0 | 1 | 0 | 0 | 0 | 0 | 0 |
| IL18R1 | 0 | 1 | 0 | 0 | 0 | 0 | 0 |
| KLRB1 | 0 | 1 | 0 | 0 | 0 | 0 | 0 |
| CEACAM1 | 0 | 1 | 0 | 0 | 0 | 0 | 1 |
| IRF7 | 0 | 1 | 0 | 0 | 0 | 0 | 0 |
| IFI35 | 0 | 1 | 0 | 0 | 0 | 0 | 1 |
| KLRC1 | 0 | 1 | 0 | 0 | 0 | 0 | 0 |
| ITLN2 | 0 | 1 | 0 | 0 | 0 | 0 | 0 |
| CCL8 | 0 | 1 | 0 | 0 | 0 | 0 | 0 |
| MX1 | 0 | 1 | 0 | 0 | 0 | 0 | 1 |
| CLEC5A | 0 | 1 | 0 | 0 | 0 | 0 | 0 |
| CXCL10 | 0 | 1 | 0 | 0 | 0 | 0 | 0 |
| IFIT2 | 0 | 1 | 0 | 0 | 0 | 1 | 0 |
| CTSG | 0 | 1 | 0 | 0 | 0 | 0 | 0 |
| LILRA3 | 0 | 1 | 0 | 0 | 0 | 1 | 1 |
| TLR10 | 0 | 0 | 1 | 0 | 0 | 0 | 0 |
| OSBPL10 | 0 | 0 | 1 | 0 | 0 | 0 | 0 |
| SNORA28 | 0 | 0 | 1 | 0 | 0 | 0 | 0 |
| FOS | 0 | 0 | 1 | 0 | 0 | 0 | 1 |
| FCRLA | 0 | 0 | 1 | 0 | 0 | 0 | 0 |
| PDXDC2P | 0 | 0 | 1 | 0 | 0 | 0 | 0 |
| CD24 | 0 | 0 | 1 | 0 | 0 | 0 | 0 |
| ABCA7 | 0 | 0 | 1 | 0 | 0 | 0 | 0 |
| PTPRC | 0 | 0 | 1 | 0 | 0 | 0 | 0 |
| LOC90925 | 0 | 0 | 1 | 0 | 0 | 0 | 0 |
| SNX29 | 0 | 0 | 1 | 0 | 0 | 0 | 0 |
| FOSB | 0 | 0 | 1 | 0 | 0 | 1 | 0 |
| JUP | 0 | 0 | 1 | 0 | 0 | 0 | 0 |
| AMFR | 0 | 0 | 1 | 0 | 0 | 0 | 0 |
| POU2AF1 | 0 | 0 | 1 | 0 | 1 | 0 | 0 |
| EGR1 | 0 | 0 | 1 | 0 | 0 | 0 | 0 |
| C16orf7 | 0 | 0 | 1 | 0 | 0 | 0 | 0 |
| TBC1D10B | 0 | 0 | 1 | 0 | 0 | 0 | 1 |
| KLF4 | 0 | 0 | 1 | 0 | 0 | 0 | 0 |
| CXCR4 | 0 | 0 | 1 | 0 | 0 | 0 | 0 |
| STAB1 | 0 | 0 | 1 | 0 | 0 | 0 | 0 |
| AL359560 | 0 | 0 | 1 | 0 | 0 | 0 | 0 |
| LOC728153 | 0 | 0 | 1 | 0 | 0 | 0 | 0 |
| RRP12 | 0 | 0 | 1 | 0 | 0 | 0 | 0 |
| LOC338817 | 0 | 0 | 1 | 0 | 0 | 0 | 0 |
| POU2F2 | 0 | 0 | 1 | 0 | 0 | 0 | 0 |
| BANK1 | 0 | 0 | 1 | 0 | 0 | 0 | 0 |
| E2F5 | 0 | 0 | 1 | 0 | 0 | 0 | 0 |
| RP2 | 0 | 0 | 1 | 0 | 0 | 0 | 0 |
| MOV10 | 0 | 0 | 1 | 0 | 0 | 0 | 0 |
| TSPAN13 | 0 | 0 | 1 | 0 | 0 | 0 | 0 |
| WARS | 0 | 0 | 1 | 0 | 0 | 1 | 0 |
| SF3A1 | 0 | 0 | 1 | 0 | 0 | 1 | 0 |
| TNFAIP2 | 0 | 0 | 1 | 0 | 0 | 1 | 0 |
| ID3 | 0 | 0 | 1 | 0 | 0 | 0 | 0 |
| LOC284837 | 0 | 0 | 1 | 0 | 0 | 0 | 0 |
| BLK | 0 | 0 | 1 | 0 | 0 | 0 | 0 |
| NAG8 | 0 | 0 | 1 | 0 | 0 | 0 | 0 |
| AK129699 | 0 | 0 | 1 | 0 | 0 | 0 | 0 |
| ATG16L2 | 0 | 0 | 1 | 0 | 0 | 0 | 0 |
| GNG7 | 0 | 0 | 1 | 0 | 0 | 0 | 0 |
| P2RX1 | 0 | 0 | 1 | 0 | 0 | 0 | 1 |
| KLHL14 | 0 | 0 | 1 | 0 | 0 | 0 | 0 |
| MDFIC | 0 | 0 | 1 | 0 | 0 | 0 | 0 |
| CD1C | 0 | 0 | 1 | 0 | 0 | 0 | 0 |
| CPT1B | 0 | 0 | 1 | 0 | 0 | 0 | 0 |
| ZNF679 | 0 | 0 | 1 | 0 | 0 | 0 | 0 |
| DUSP1 | 0 | 0 | 1 | 0 | 0 | 0 | 0 |
| PARP12 | 0 | 0 | 1 | 0 | 0 | 0 | 0 |
| X69637 | 0 | 0 | 1 | 0 | 0 | 0 | 0 |
| MORC3 | 0 | 0 | 1 | 0 | 0 | 0 | 0 |
| ERAP2 | 0 | 0 | 1 | 0 | 0 | 0 | 1 |
| STAT2 | 0 | 0 | 1 | 0 | 0 | 0 | 0 |
| STAP1 | 0 | 0 | 1 | 0 | 0 | 0 | 0 |
| TYMP | 0 | 0 | 1 | 0 | 0 | 1 | 0 |
| SAMD9L | 0 | 0 | 1 | 0 | 0 | 0 | 0 |
| VPREB3 | 0 | 0 | 1 | 0 | 0 | 0 | 0 |
| FCRL5 | 0 | 0 | 1 | 0 | 0 | 0 | 0 |
| BBS10 | 0 | 0 | 1 | 0 | 0 | 0 | 0 |
| AK024852 | 0 | 0 | 1 | 0 | 0 | 0 | 0 |
| GABBR1 | 0 | 0 | 1 | 0 | 0 | 0 | 0 |
| FREM1 | 0 | 0 | 0 | 1 | 0 | 0 | 0 |
| SUSD2 | 0 | 0 | 0 | 1 | 0 | 0 | 0 |
| FRAS1 | 0 | 0 | 0 | 1 | 0 | 0 | 0 |
| ERICH1-AS1 | 0 | 0 | 0 | 1 | 0 | 0 | 0 |
| RP11-627G23.1 | 0 | 0 | 0 | 1 | 0 | 0 | 0 |
| CTD-2047H16.4 | 0 | 0 | 0 | 1 | 0 | 0 | 0 |
| COLQ | 0 | 0 | 0 | 1 | 0 | 0 | 0 |
| MIR143HG | 0 | 0 | 0 | 1 | 0 | 1 | 0 |
| AC131025.8 | 0 | 0 | 0 | 1 | 0 | 0 | 0 |
| RBPMS | 0 | 0 | 0 | 1 | 0 | 0 | 0 |
| MUC19 | 0 | 0 | 0 | 1 | 0 | 0 | 0 |
| LAMA5 | 0 | 0 | 0 | 1 | 0 | 0 | 0 |
| TTC21A | 0 | 0 | 0 | 1 | 0 | 0 | 0 |
| CTD-2349P21.5 | 0 | 0 | 0 | 1 | 0 | 0 | 0 |
| LIMS2 | 0 | 0 | 0 | 1 | 0 | 0 | 0 |
| IQCJ | 0 | 0 | 0 | 1 | 0 | 0 | 0 |
| ANKRD19P | 0 | 0 | 0 | 1 | 0 | 0 | 0 |
| MIR145 | 0 | 0 | 0 | 1 | 0 | 0 | 0 |
| HSPG2 | 0 | 0 | 0 | 1 | 0 | 0 | 0 |
| COL14A1 | 0 | 0 | 0 | 1 | 0 | 0 | 0 |
| AC093642.3 | 0 | 0 | 0 | 1 | 0 | 0 | 0 |
| MIAT | 0 | 0 | 0 | 1 | 0 | 0 | 0 |
| FAM118A | 0 | 0 | 0 | 1 | 0 | 0 | 1 |
| AKT3-IT1 | 0 | 0 | 0 | 1 | 0 | 0 | 0 |
| RP4-673D20.3 | 0 | 0 | 0 | 1 | 0 | 0 | 0 |
| CASQ2 | 0 | 0 | 0 | 1 | 0 | 0 | 0 |
| RP4-555D20.2 | 0 | 0 | 0 | 1 | 0 | 0 | 0 |
| MYOCD | 0 | 0 | 0 | 1 | 0 | 0 | 0 |
| RP11-159D12.2 | 0 | 0 | 0 | 1 | 0 | 0 | 0 |
| CAPG | 0 | 0 | 0 | 1 | 0 | 0 | 1 |
| RP6-99M1.2 | 0 | 0 | 0 | 1 | 0 | 0 | 0 |
| GAPDHP66 | 0 | 0 | 0 | 1 | 0 | 0 | 0 |
| FGF14-IT1 | 0 | 0 | 0 | 1 | 0 | 0 | 0 |
| AC144833.1 | 0 | 0 | 0 | 1 | 0 | 0 | 0 |
| ROCK1P1 | 0 | 0 | 0 | 1 | 0 | 0 | 0 |
| C20orf203 | 0 | 0 | 0 | 1 | 0 | 0 | 0 |
| HNRNPCP6 | 0 | 0 | 0 | 1 | 0 | 0 | 0 |
| RP11-989F5.3 | 0 | 0 | 0 | 1 | 0 | 0 | 0 |
| CRYZ | 0 | 0 | 0 | 1 | 0 | 0 | 0 |
| COPS8P2 | 0 | 0 | 0 | 1 | 0 | 0 | 0 |
| AOC3 | 0 | 0 | 0 | 1 | 0 | 0 | 0 |
| ZNF410 | 0 | 0 | 0 | 1 | 0 | 0 | 0 |
| MIR770 | 0 | 0 | 0 | 1 | 0 | 0 | 0 |
| CEACAM19 | 0 | 0 | 0 | 1 | 0 | 0 | 0 |
| FOXL1 | 0 | 0 | 0 | 1 | 0 | 0 | 0 |
| RP11-379B18.5 | 0 | 0 | 0 | 1 | 0 | 0 | 0 |
| PVT1 | 0 | 0 | 0 | 1 | 0 | 0 | 0 |
| ADIRF-AS1 | 0 | 0 | 0 | 1 | 0 | 0 | 0 |
| RP11-981G7.2 | 0 | 0 | 0 | 1 | 0 | 0 | 0 |
| RP11-54A4.2 | 0 | 0 | 0 | 1 | 0 | 0 | 0 |
| GJA6P | 0 | 0 | 0 | 1 | 0 | 0 | 0 |
| SCUBE3 | 0 | 0 | 0 | 1 | 0 | 0 | 0 |
| RP11-768G7.2 | 0 | 0 | 0 | 1 | 0 | 0 | 0 |
| SLC25A34 | 0 | 0 | 0 | 1 | 0 | 1 | 0 |
| AC019118.2 | 0 | 0 | 0 | 1 | 0 | 0 | 0 |
| AC011747.4 | 0 | 0 | 0 | 1 | 0 | 0 | 0 |
| H3F3AP4 | 0 | 0 | 0 | 1 | 0 | 0 | 0 |
| LINC00599 | 0 | 0 | 0 | 1 | 0 | 0 | 0 |
| RP11-509E10.1 | 0 | 0 | 0 | 1 | 0 | 0 | 0 |
| VWCE | 0 | 0 | 0 | 1 | 0 | 0 | 1 |
| BTBD19 | 0 | 0 | 0 | 1 | 0 | 0 | 0 |
| CTD-2281E23.2 | 0 | 0 | 0 | 1 | 0 | 0 | 0 |
| RP11-535M15.2 | 0 | 0 | 0 | 1 | 0 | 0 | 0 |
| RP11-981G7.1 | 0 | 0 | 0 | 1 | 0 | 0 | 0 |
| MIR137HG | 0 | 0 | 0 | 1 | 0 | 0 | 0 |
| RNF139-AS1 | 0 | 0 | 0 | 1 | 0 | 0 | 0 |
| PTCH2 | 0 | 0 | 0 | 1 | 0 | 0 | 0 |
| ADAMTS15 | 0 | 0 | 0 | 1 | 0 | 0 | 0 |
| CTA-407F11.6 | 0 | 0 | 0 | 1 | 0 | 0 | 0 |
| SMYD3-IT1 | 0 | 0 | 0 | 1 | 0 | 0 | 0 |
| ELN | 0 | 0 | 0 | 1 | 1 | 0 | 0 |
| MKRN5P | 0 | 0 | 0 | 1 | 0 | 0 | 0 |
| GNB3 | 0 | 0 | 0 | 1 | 0 | 0 | 0 |
| RP11-282K24.3 | 0 | 0 | 0 | 1 | 0 | 0 | 0 |
| XXbac-BPG154L12.4 | 0 | 0 | 0 | 1 | 0 | 0 | 0 |
| C1orf132 | 0 | 0 | 0 | 1 | 0 | 0 | 0 |
| AF131215.3 | 0 | 0 | 0 | 1 | 0 | 0 | 0 |
| RP11-53B2.2 | 0 | 0 | 0 | 1 | 0 | 0 | 0 |
| RP11-981G7.6 | 0 | 0 | 0 | 1 | 0 | 0 | 0 |
| CORO6 | 0 | 0 | 0 | 1 | 0 | 0 | 0 |
| MEG3 | 0 | 0 | 0 | 1 | 0 | 0 | 0 |
| DNAJC5G | 0 | 0 | 0 | 1 | 1 | 0 | 0 |
| LAMA3 | 0 | 0 | 0 | 1 | 0 | 0 | 0 |
| RP11-16E23.4 | 0 | 0 | 0 | 1 | 0 | 0 | 0 |
| FAM153B | 0 | 0 | 0 | 1 | 0 | 0 | 1 |
| GET4 | 0 | 0 | 0 | 1 | 0 | 0 | 0 |
| LINC00595 | 0 | 0 | 0 | 1 | 0 | 0 | 0 |
| TPCN2 | 0 | 0 | 0 | 1 | 0 | 0 | 0 |
| RP11-367O10.1 | 0 | 0 | 0 | 1 | 0 | 0 | 0 |
| HIF3A | 0 | 0 | 0 | 1 | 1 | 0 | 0 |
| RP11-397O4.1 | 0 | 0 | 0 | 1 | 0 | 0 | 0 |
| AL132709.8 | 0 | 0 | 0 | 1 | 0 | 0 | 0 |
| GOLGA8A | 0 | 0 | 0 | 1 | 0 | 0 | 0 |
| DSCAM-IT1 | 0 | 0 | 0 | 1 | 0 | 0 | 0 |
| C6orf163 | 0 | 0 | 0 | 1 | 0 | 0 | 0 |
| C10orf113 | 0 | 0 | 0 | 1 | 0 | 0 | 0 |
| FGF17 | 0 | 0 | 0 | 1 | 0 | 0 | 0 |
| MSS51 | 0 | 0 | 0 | 1 | 0 | 0 | 0 |
| LINC00106 | 0 | 0 | 0 | 1 | 0 | 0 | 0 |
| RP11-572C21.1 | 0 | 0 | 0 | 1 | 0 | 0 | 0 |
| RP11-359E3.4 | 0 | 0 | 0 | 1 | 0 | 0 | 0 |
| C1RL-AS1 | 0 | 0 | 0 | 1 | 0 | 0 | 0 |
| CATSPER2 | 0 | 0 | 0 | 1 | 0 | 0 | 0 |
| GHRLOS | 0 | 0 | 0 | 1 | 0 | 0 | 0 |
| KLF15 | 0 | 0 | 0 | 0 | 1 | 0 | 0 |
| VGF | 0 | 0 | 0 | 0 | 1 | 0 | 0 |
| ADCYAP1 | 0 | 0 | 0 | 0 | 1 | 0 | 0 |
| SLC6A9 | 0 | 0 | 0 | 0 | 1 | 0 | 0 |
| NRN1 | 0 | 0 | 0 | 0 | 1 | 0 | 0 |
| SGO1 | 0 | 0 | 0 | 0 | 1 | 0 | 0 |
| NEUROD6 | 0 | 0 | 0 | 0 | 1 | 0 | 0 |
| PRKX | 0 | 0 | 0 | 0 | 1 | 1 | 0 |
| PRMT8 | 0 | 0 | 0 | 0 | 1 | 0 | 0 |
| PPEF1 | 0 | 0 | 0 | 0 | 1 | 0 | 0 |
| RPH3A | 0 | 0 | 0 | 0 | 1 | 0 | 0 |
| PCSK1 | 0 | 0 | 0 | 0 | 1 | 0 | 0 |
| JADE3 | 0 | 0 | 0 | 0 | 1 | 0 | 0 |
| C3orf80 | 0 | 0 | 0 | 0 | 1 | 0 | 0 |
| MID1IP1 | 0 | 0 | 0 | 0 | 1 | 0 | 1 |
| LOC102724596 | 0 | 0 | 0 | 0 | 1 | 0 | 0 |
| DNAH11 | 0 | 0 | 0 | 0 | 1 | 0 | 0 |
| GNRH1 | 0 | 0 | 0 | 0 | 1 | 0 | 0 |
| MAS1 | 0 | 0 | 0 | 0 | 1 | 0 | 0 |
| SOWAHB | 0 | 0 | 0 | 0 | 1 | 0 | 0 |
| FAM53B-AS1 | 0 | 0 | 0 | 0 | 1 | 0 | 0 |
| MSC | 0 | 0 | 0 | 0 | 1 | 0 | 0 |
| MDH1B | 0 | 0 | 0 | 0 | 1 | 0 | 0 |
| CLDN15 | 0 | 0 | 0 | 0 | 1 | 0 | 0 |
| CRH | 0 | 0 | 0 | 0 | 1 | 0 | 0 |
| FOXO4 | 0 | 0 | 0 | 0 | 1 | 0 | 1 |
| ABCC12 | 0 | 0 | 0 | 0 | 1 | 0 | 0 |
| TAC1 | 0 | 0 | 0 | 0 | 1 | 0 | 0 |
| OTOGL | 0 | 0 | 0 | 0 | 1 | 0 | 0 |
| USP2-AS1 | 0 | 0 | 0 | 0 | 1 | 0 | 0 |
| GCNT4 | 0 | 0 | 0 | 0 | 1 | 0 | 0 |
| BEX5 | 0 | 0 | 0 | 0 | 1 | 0 | 0 |
| NPFF | 0 | 0 | 0 | 0 | 1 | 0 | 0 |
| CPM | 0 | 0 | 0 | 0 | 1 | 0 | 0 |
| ALOX12B | 0 | 0 | 0 | 0 | 1 | 0 | 0 |
| LINC01546 | 0 | 0 | 0 | 0 | 1 | 0 | 0 |
| GJD2 | 0 | 0 | 0 | 0 | 1 | 0 | 0 |
| MGC16025 | 0 | 0 | 0 | 0 | 1 | 0 | 0 |
| FAM222A | 0 | 0 | 0 | 0 | 1 | 0 | 0 |
| LBX2 | 0 | 0 | 0 | 0 | 1 | 0 | 0 |
| HSPB3 | 0 | 0 | 0 | 0 | 1 | 0 | 0 |
| PCDHGC5 | 0 | 0 | 0 | 0 | 1 | 0 | 0 |
| KRT5 | 0 | 0 | 0 | 0 | 1 | 0 | 0 |
| STAT4 | 0 | 0 | 0 | 0 | 1 | 0 | 0 |
| ANKRD18DP | 0 | 0 | 0 | 0 | 1 | 0 | 0 |
| MCHR2 | 0 | 0 | 0 | 0 | 1 | 0 | 0 |
| PNMA3 | 0 | 0 | 0 | 0 | 1 | 0 | 0 |
| TNRC6C-AS1 | 0 | 0 | 0 | 0 | 1 | 0 | 0 |
| OVOL3 | 0 | 0 | 0 | 0 | 1 | 0 | 0 |
| C10orf62 | 0 | 0 | 0 | 0 | 1 | 0 | 0 |
| PPFIBP2 | 0 | 0 | 0 | 0 | 1 | 0 | 0 |
| LINC01561 | 0 | 0 | 0 | 0 | 1 | 0 | 0 |
| MPO | 0 | 0 | 0 | 0 | 1 | 0 | 0 |
| NAP1L2 | 0 | 0 | 0 | 0 | 1 | 0 | 0 |
| LOC100129316 | 0 | 0 | 0 | 0 | 1 | 0 | 0 |
| NAP1L5 | 0 | 0 | 0 | 0 | 1 | 0 | 0 |
| STON1 | 0 | 0 | 0 | 0 | 1 | 0 | 0 |
| ADRA2B | 0 | 0 | 0 | 0 | 1 | 0 | 0 |
| LINC01202 | 0 | 0 | 0 | 0 | 1 | 0 | 0 |
| THCAT155 | 0 | 0 | 0 | 0 | 1 | 0 | 0 |
| SST | 0 | 0 | 0 | 0 | 1 | 0 | 0 |
| NAT16 | 0 | 0 | 0 | 0 | 1 | 0 | 0 |
| CHML | 0 | 0 | 0 | 0 | 1 | 0 | 0 |
| ZBBX | 0 | 0 | 0 | 0 | 1 | 0 | 0 |
| MSC-AS1 | 0 | 0 | 0 | 0 | 1 | 0 | 0 |
| PAK1 | 0 | 0 | 0 | 0 | 1 | 1 | 0 |
| VSNL1 | 0 | 0 | 0 | 0 | 1 | 0 | 0 |
| FBLN7 | 0 | 0 | 0 | 0 | 1 | 0 | 0 |
| LOC100507194 | 0 | 0 | 0 | 0 | 1 | 0 | 0 |
| MCM7 | 0 | 0 | 0 | 0 | 1 | 0 | 0 |
| ADCY10P1 | 0 | 0 | 0 | 0 | 1 | 0 | 0 |
| SCG2 | 0 | 0 | 0 | 0 | 1 | 0 | 0 |
| HIP1 | 0 | 0 | 0 | 0 | 1 | 0 | 0 |
| PVRIG | 0 | 0 | 0 | 0 | 1 | 0 | 1 |
| RGS4 | 0 | 0 | 0 | 0 | 1 | 0 | 0 |
| LINC00898 | 0 | 0 | 0 | 0 | 1 | 0 | 0 |
| SH2D5 | 0 | 0 | 0 | 0 | 1 | 0 | 0 |
| CARTPT | 0 | 0 | 0 | 0 | 1 | 0 | 0 |
| SAP25 | 0 | 0 | 0 | 0 | 1 | 0 | 0 |
| CCDC184 | 0 | 0 | 0 | 0 | 1 | 0 | 0 |
| LDLRAP1 | 0 | 0 | 0 | 0 | 1 | 0 | 0 |
| ZNF652 | 0 | 0 | 0 | 0 | 1 | 0 | 0 |
| KIF1C | 0 | 0 | 0 | 0 | 1 | 0 | 0 |
| LIN28B-AS1 | 0 | 0 | 0 | 0 | 1 | 0 | 0 |
| PKMYT1 | 0 | 0 | 0 | 0 | 1 | 0 | 0 |
| SH2D6 | 0 | 0 | 0 | 0 | 1 | 0 | 0 |
| GAP43 | 0 | 0 | 0 | 0 | 1 | 0 | 0 |
| BEX1 | 0 | 0 | 0 | 0 | 1 | 0 | 0 |
| DTHD1 | 0 | 0 | 0 | 0 | 1 | 0 | 0 |
| SPATC1 | 0 | 0 | 0 | 0 | 1 | 0 | 0 |
| SPTSSB | 0 | 0 | 0 | 0 | 1 | 0 | 0 |
| ZBTB20-AS1 | 0 | 0 | 0 | 0 | 1 | 0 | 0 |
| LINC00463 | 0 | 0 | 0 | 0 | 1 | 0 | 0 |
| CRYM | 0 | 0 | 0 | 0 | 1 | 0 | 0 |
| UCKL1-AS1 | 0 | 0 | 0 | 0 | 1 | 1 | 0 |
| BLID | 0 | 0 | 0 | 0 | 1 | 0 | 0 |
| OLFM3 | 0 | 0 | 0 | 0 | 1 | 0 | 0 |
| LOC101926975 | 0 | 0 | 0 | 0 | 1 | 0 | 0 |
| SERTAD4-AS1 | 0 | 0 | 0 | 0 | 1 | 0 | 0 |
| LOC102724484 | 0 | 0 | 0 | 0 | 1 | 0 | 0 |
| MORN3 | 0 | 0 | 0 | 0 | 1 | 0 | 1 |
| LCN6 | 0 | 0 | 0 | 0 | 1 | 0 | 0 |
| ZDHHC23 | 0 | 0 | 0 | 0 | 1 | 0 | 0 |
| HMGCS1 | 0 | 0 | 0 | 0 | 1 | 0 | 0 |
| SERPINF2 | 0 | 0 | 0 | 0 | 1 | 0 | 0 |
| TLDC2 | 0 | 0 | 0 | 0 | 1 | 0 | 0 |
| NEAT1 | 0 | 0 | 0 | 0 | 1 | 0 | 0 |
| ITPKB-IT1 | 0 | 0 | 0 | 0 | 1 | 0 | 0 |
| LOC101929715 | 0 | 0 | 0 | 0 | 1 | 0 | 0 |
| PLA2G4B | 0 | 0 | 0 | 0 | 1 | 0 | 0 |
| LOC285762 | 0 | 0 | 0 | 0 | 1 | 0 | 0 |
| ATAD3C | 0 | 0 | 0 | 0 | 1 | 1 | 0 |
| CYP4B1 | 0 | 0 | 0 | 0 | 1 | 0 | 0 |
| CALY | 0 | 0 | 0 | 0 | 1 | 0 | 0 |
| LOC102723493 | 0 | 0 | 0 | 0 | 1 | 0 | 0 |
| SYP | 0 | 0 | 0 | 0 | 1 | 0 | 0 |
| TMPRSS5 | 0 | 0 | 0 | 0 | 1 | 0 | 0 |
| KIF19 | 0 | 0 | 0 | 0 | 1 | 0 | 0 |
| ARRDC2 | 0 | 0 | 0 | 0 | 1 | 0 | 0 |
| MYOT | 0 | 0 | 0 | 0 | 1 | 0 | 0 |
| FZD10-AS1 | 0 | 0 | 0 | 0 | 1 | 0 | 0 |
| LLGL2 | 0 | 0 | 0 | 0 | 1 | 0 | 0 |
| TUBB2A | 0 | 0 | 0 | 0 | 1 | 0 | 1 |
| PCA3 | 0 | 0 | 0 | 0 | 1 | 0 | 0 |
| MCHR1 | 0 | 0 | 0 | 0 | 1 | 0 | 0 |
| OLMALINC | 0 | 0 | 0 | 0 | 1 | 0 | 0 |
| SERTM1 | 0 | 0 | 0 | 0 | 1 | 0 | 0 |
| CLDN16 | 0 | 0 | 0 | 0 | 1 | 0 | 0 |
| PCP4L1 | 0 | 0 | 0 | 0 | 1 | 0 | 0 |
| SLC10A1 | 0 | 0 | 0 | 0 | 1 | 0 | 0 |
| LYRM9 | 0 | 0 | 0 | 0 | 1 | 0 | 0 |
| PTOV1-AS2 | 0 | 0 | 0 | 0 | 1 | 0 | 0 |
| OPTC | 0 | 0 | 0 | 0 | 1 | 0 | 0 |
| CPLX1 | 0 | 0 | 0 | 0 | 1 | 0 | 0 |
| VCAN-AS1 | 0 | 0 | 0 | 0 | 1 | 0 | 0 |
| FANCB | 0 | 0 | 0 | 0 | 1 | 0 | 0 |
| HTR3B | 0 | 0 | 0 | 0 | 1 | 0 | 0 |
| STYK1 | 0 | 0 | 0 | 0 | 1 | 0 | 0 |
| TUBB3 | 0 | 0 | 0 | 0 | 1 | 0 | 1 |
| OR7A5 | 0 | 0 | 0 | 0 | 1 | 0 | 0 |
| LINC01168 | 0 | 0 | 0 | 0 | 1 | 0 | 0 |
| LOC728084 | 0 | 0 | 0 | 0 | 1 | 0 | 0 |
| ARL4D | 0 | 0 | 0 | 0 | 1 | 0 | 0 |
| NRON | 0 | 0 | 0 | 0 | 1 | 0 | 0 |
| MIR6717 | 0 | 0 | 0 | 0 | 1 | 0 | 0 |
| PSG8 | 0 | 0 | 0 | 0 | 1 | 0 | 0 |
| ATOH7 | 0 | 0 | 0 | 0 | 1 | 0 | 0 |
| MAP4K4 | 0 | 0 | 0 | 0 | 1 | 0 | 0 |
| KMO | 0 | 0 | 0 | 0 | 1 | 0 | 0 |
| BAALC-AS1 | 0 | 0 | 0 | 0 | 1 | 0 | 0 |
| LINCR-0002 | 0 | 0 | 0 | 0 | 1 | 0 | 0 |
| CXCR1 | 0 | 0 | 0 | 0 | 1 | 0 | 1 |
| KCNB2 | 0 | 0 | 0 | 0 | 1 | 0 | 0 |
| C6orf223 | 0 | 0 | 0 | 0 | 1 | 0 | 0 |
| LINC01007 | 0 | 0 | 0 | 0 | 1 | 0 | 0 |
| LINC00460 | 0 | 0 | 0 | 0 | 1 | 0 | 0 |
| LOC100288637 | 0 | 0 | 0 | 0 | 1 | 0 | 0 |
| HES5 | 0 | 0 | 0 | 0 | 1 | 0 | 0 |
| LOC100130548 | 0 | 0 | 0 | 0 | 1 | 0 | 0 |
| SAMD11 | 0 | 0 | 0 | 0 | 1 | 0 | 0 |
| GABRA1 | 0 | 0 | 0 | 0 | 1 | 0 | 0 |
| GAD1 | 0 | 0 | 0 | 0 | 1 | 0 | 0 |
| GFRA2 | 0 | 0 | 0 | 0 | 1 | 0 | 0 |
| SCHLAP1 | 0 | 0 | 0 | 0 | 1 | 0 | 0 |
| GAD2 | 0 | 0 | 0 | 0 | 1 | 0 | 0 |
| RIIAD1 | 0 | 0 | 0 | 0 | 1 | 0 | 0 |
| LRRC73 | 0 | 0 | 0 | 0 | 1 | 0 | 0 |
| FAM222A-AS1 | 0 | 0 | 0 | 0 | 1 | 0 | 0 |
| IGF1 | 0 | 0 | 0 | 0 | 1 | 0 | 0 |
| ADAMTS3 | 0 | 0 | 0 | 0 | 1 | 0 | 0 |
| SLC23A3 | 0 | 0 | 0 | 0 | 1 | 0 | 0 |
| MRGPRF | 0 | 0 | 0 | 0 | 1 | 0 | 0 |
| FREM3 | 0 | 0 | 0 | 0 | 1 | 0 | 0 |
| LINC01164 | 0 | 0 | 0 | 0 | 1 | 0 | 0 |
| BOK | 0 | 0 | 0 | 0 | 1 | 0 | 0 |
| LINC01445 | 0 | 0 | 0 | 0 | 1 | 0 | 0 |
| LINC00601 | 0 | 0 | 0 | 0 | 1 | 0 | 0 |
| LOC105378385 | 0 | 0 | 0 | 0 | 1 | 0 | 0 |
| QDPR | 0 | 0 | 0 | 0 | 1 | 0 | 0 |
| KIRREL3-AS3 | 0 | 0 | 0 | 0 | 1 | 0 | 0 |
| PRSS8 | 0 | 0 | 0 | 0 | 1 | 0 | 0 |
| PTPRD-AS2 | 0 | 0 | 0 | 0 | 1 | 0 | 0 |
| LOC339685 | 0 | 0 | 0 | 0 | 1 | 0 | 0 |
| SNAP25 | 0 | 0 | 0 | 0 | 1 | 0 | 0 |
| CCKBR | 0 | 0 | 0 | 0 | 1 | 0 | 0 |
| PLPP2 | 0 | 0 | 0 | 0 | 1 | 0 | 0 |
| CDK18 | 0 | 0 | 0 | 0 | 1 | 0 | 0 |
| MDH1 | 0 | 0 | 0 | 0 | 1 | 0 | 0 |
| COL5A2 | 0 | 0 | 0 | 0 | 1 | 0 | 0 |
| MAMDC4 | 0 | 0 | 0 | 0 | 1 | 0 | 0 |
| FAM81A | 0 | 0 | 0 | 0 | 1 | 0 | 0 |
| OLAH | 0 | 0 | 0 | 0 | 1 | 0 | 0 |
| SLC12A1 | 0 | 0 | 0 | 0 | 1 | 0 | 0 |
| ABCA6 | 0 | 0 | 0 | 0 | 1 | 0 | 0 |
| ENC1 | 0 | 0 | 0 | 0 | 1 | 0 | 0 |
| AMIGO2 | 0 | 0 | 0 | 0 | 1 | 0 | 0 |
| EGFR-AS1 | 0 | 0 | 0 | 0 | 1 | 0 | 0 |
| BMP3 | 0 | 0 | 0 | 0 | 1 | 0 | 0 |
| CA3-AS1 | 0 | 0 | 0 | 0 | 1 | 0 | 0 |
| MAN2A1 | 0 | 0 | 0 | 0 | 1 | 0 | 0 |
| PPP1R2P3 | 0 | 0 | 0 | 0 | 1 | 0 | 0 |
| TRPA1 | 0 | 0 | 0 | 0 | 1 | 0 | 0 |
| CLDN34 | 0 | 0 | 0 | 0 | 1 | 0 | 0 |
| SMIM5 | 0 | 0 | 0 | 0 | 1 | 0 | 1 |
| C9orf139 | 0 | 0 | 0 | 0 | 1 | 0 | 0 |
| INHBA-AS1 | 0 | 0 | 0 | 0 | 1 | 0 | 0 |
| F3 | 0 | 0 | 0 | 0 | 1 | 0 | 0 |
| MIR5690 | 0 | 0 | 0 | 0 | 1 | 0 | 0 |
| CD244 | 0 | 0 | 0 | 0 | 1 | 0 | 0 |
| GZMM | 0 | 0 | 0 | 0 | 1 | 0 | 0 |
| MDGA1 | 0 | 0 | 0 | 0 | 1 | 0 | 0 |
| LINC01257 | 0 | 0 | 0 | 0 | 1 | 0 | 0 |
| CLEC18A | 0 | 0 | 0 | 0 | 1 | 0 | 0 |
| DLX6-AS1 | 0 | 0 | 0 | 0 | 1 | 0 | 0 |
| OLFM4 | 0 | 0 | 0 | 0 | 1 | 0 | 1 |
| LOC101929754 | 0 | 0 | 0 | 0 | 1 | 0 | 0 |
| NPY2R | 0 | 0 | 0 | 0 | 1 | 0 | 0 |
| MIR3619 | 0 | 0 | 0 | 0 | 1 | 0 | 0 |
| RAD21L1 | 0 | 0 | 0 | 0 | 1 | 0 | 0 |
| MIR1250 | 0 | 0 | 0 | 0 | 1 | 0 | 0 |
| SELE | 0 | 0 | 0 | 0 | 1 | 0 | 0 |
| PRSS35 | 0 | 0 | 0 | 0 | 1 | 0 | 0 |
| SFTPA1 | 0 | 0 | 0 | 0 | 1 | 0 | 0 |
| LOC100505912 | 0 | 0 | 0 | 0 | 1 | 0 | 0 |
| LINC01336 | 0 | 0 | 0 | 0 | 1 | 0 | 0 |
| TGFBR3L | 0 | 0 | 0 | 0 | 1 | 0 | 0 |
| LOC339529 | 0 | 0 | 0 | 0 | 1 | 0 | 0 |
| LOC101927043 | 0 | 0 | 0 | 0 | 1 | 0 | 0 |
| ANO3 | 0 | 0 | 0 | 0 | 1 | 0 | 0 |
| PRKCG | 0 | 0 | 0 | 0 | 1 | 0 | 0 |
| LOC101929151 | 0 | 0 | 0 | 0 | 1 | 0 | 0 |
| LINC01107 | 0 | 0 | 0 | 0 | 1 | 0 | 0 |
| SGCA | 0 | 0 | 0 | 0 | 1 | 0 | 0 |
| ABCA8 | 0 | 0 | 0 | 0 | 1 | 0 | 0 |
| SNORA104 | 0 | 0 | 0 | 0 | 1 | 0 | 0 |
| FAAHP1 | 0 | 0 | 0 | 0 | 1 | 0 | 0 |
| RAB3A | 0 | 0 | 0 | 0 | 1 | 0 | 0 |
| LOC100506136 | 0 | 0 | 0 | 0 | 1 | 0 | 0 |
| SNX10 | 0 | 0 | 0 | 0 | 1 | 0 | 0 |
| NGFR | 0 | 0 | 0 | 0 | 1 | 0 | 0 |
| CCR6 | 0 | 0 | 0 | 0 | 1 | 0 | 0 |
| SYT10 | 0 | 0 | 0 | 0 | 1 | 0 | 0 |
| DHH | 0 | 0 | 0 | 0 | 1 | 0 | 0 |
| ANKRD40 | 0 | 0 | 0 | 0 | 1 | 0 | 0 |
| MIR340 | 0 | 0 | 0 | 0 | 1 | 0 | 0 |
| C11orf87 | 0 | 0 | 0 | 0 | 1 | 0 | 0 |
| SMTN | 0 | 0 | 0 | 0 | 1 | 0 | 0 |
| MND1 | 0 | 0 | 0 | 0 | 1 | 0 | 0 |
| IL33 | 0 | 0 | 0 | 0 | 1 | 0 | 0 |
| LRRC46 | 0 | 0 | 0 | 0 | 1 | 0 | 0 |
| LGALS2 | 0 | 0 | 0 | 0 | 1 | 0 | 0 |
| DOCK5 | 0 | 0 | 0 | 0 | 1 | 0 | 0 |
| PRR34-AS1 | 0 | 0 | 0 | 0 | 1 | 0 | 0 |
| CXCR2 | 0 | 0 | 0 | 0 | 1 | 0 | 0 |
| DLX6 | 0 | 0 | 0 | 0 | 1 | 0 | 0 |
| PART1 | 0 | 0 | 0 | 0 | 1 | 0 | 0 |
| LOC101927139 | 0 | 0 | 0 | 0 | 1 | 0 | 0 |
| THRSP | 0 | 0 | 0 | 0 | 1 | 0 | 0 |
| SNORD114-21 | 0 | 0 | 0 | 0 | 1 | 0 | 0 |
| CLCA4 | 0 | 0 | 0 | 0 | 1 | 0 | 0 |
| CLMN | 0 | 0 | 0 | 0 | 1 | 0 | 0 |
| ADORA2A | 0 | 0 | 0 | 0 | 1 | 0 | 0 |
| CHRNA7 | 0 | 0 | 0 | 0 | 1 | 0 | 0 |
| TCP11 | 0 | 0 | 0 | 0 | 1 | 0 | 0 |
| HILS1 | 0 | 0 | 0 | 0 | 1 | 0 | 0 |
| BGLAP | 0 | 0 | 0 | 0 | 1 | 0 | 0 |
| BOK-AS1 | 0 | 0 | 0 | 0 | 1 | 0 | 0 |
| GADD45A | 0 | 0 | 0 | 0 | 1 | 0 | 0 |
| OVCH2 | 0 | 0 | 0 | 0 | 1 | 0 | 0 |
| RDH12 | 0 | 0 | 0 | 0 | 1 | 0 | 0 |
| SH2D3A | 0 | 0 | 0 | 0 | 1 | 0 | 0 |
| LOC100506274 | 0 | 0 | 0 | 0 | 1 | 0 | 0 |
| IRX2 | 0 | 0 | 0 | 0 | 1 | 0 | 0 |
| GMNC | 0 | 0 | 0 | 0 | 1 | 0 | 0 |
| MUC3A | 0 | 0 | 0 | 0 | 1 | 0 | 0 |
| KCNV1 | 0 | 0 | 0 | 0 | 1 | 0 | 0 |
| MIR2467 | 0 | 0 | 0 | 0 | 1 | 0 | 0 |
| LMNTD2 | 0 | 0 | 0 | 0 | 1 | 0 | 0 |
| FAM21EP | 0 | 0 | 0 | 0 | 1 | 0 | 0 |
| SALRNA2 | 0 | 0 | 0 | 0 | 1 | 0 | 0 |
| FOXD2 | 0 | 0 | 0 | 0 | 1 | 0 | 0 |
| C11orf94 | 0 | 0 | 0 | 0 | 1 | 0 | 0 |
| LINC00323 | 0 | 0 | 0 | 0 | 1 | 0 | 0 |
| BMP7-AS1 | 0 | 0 | 0 | 0 | 1 | 0 | 0 |
| PRTN3 | 0 | 0 | 0 | 0 | 1 | 0 | 0 |
| GABRG2 | 0 | 0 | 0 | 0 | 1 | 0 | 0 |
| SLC16A6 | 0 | 0 | 0 | 0 | 1 | 0 | 0 |
| LOC108783645 | 0 | 0 | 0 | 0 | 1 | 0 | 0 |
| FOXD1 | 0 | 0 | 0 | 0 | 1 | 0 | 0 |
| SPDEF | 0 | 0 | 0 | 0 | 1 | 0 | 0 |
| LINC00484 | 0 | 0 | 0 | 0 | 1 | 0 | 0 |
| SLC30A3 | 0 | 0 | 0 | 0 | 1 | 0 | 0 |
| PIK3C2G | 0 | 0 | 0 | 0 | 1 | 0 | 0 |
| LOC101928766 | 0 | 0 | 0 | 0 | 1 | 0 | 0 |
| CPAMD8 | 0 | 0 | 0 | 0 | 1 | 0 | 0 |
| NLRP14 | 0 | 0 | 0 | 0 | 1 | 0 | 0 |
| LINC01476 | 0 | 0 | 0 | 0 | 1 | 0 | 0 |
| RND1 | 0 | 0 | 0 | 0 | 1 | 0 | 0 |
| FMO3 | 0 | 0 | 0 | 0 | 1 | 0 | 0 |
| LOC100288866 | 0 | 0 | 0 | 0 | 1 | 0 | 0 |
| LOC101927406 | 0 | 0 | 0 | 0 | 1 | 0 | 0 |
| TP73 | 0 | 0 | 0 | 0 | 1 | 0 | 0 |
| LINC01119 | 0 | 0 | 0 | 0 | 1 | 0 | 0 |
| C1QL3 | 0 | 0 | 0 | 0 | 1 | 0 | 0 |
| FIBCD1 | 0 | 0 | 0 | 0 | 1 | 0 | 0 |
| CLDN9 | 0 | 0 | 0 | 0 | 1 | 0 | 0 |
| LOC105377448 | 0 | 0 | 0 | 0 | 1 | 0 | 0 |
| UHRF1 | 0 | 0 | 0 | 0 | 1 | 0 | 0 |
| LINC01296 | 0 | 0 | 0 | 0 | 1 | 0 | 0 |
| ROR1-AS1 | 0 | 0 | 0 | 0 | 1 | 0 | 0 |
| TSG1 | 0 | 0 | 0 | 0 | 1 | 0 | 0 |
| GRP | 0 | 0 | 0 | 0 | 1 | 0 | 0 |
| NEFL | 0 | 0 | 0 | 0 | 1 | 0 | 0 |
| FFAR4 | 0 | 0 | 0 | 0 | 1 | 0 | 0 |
| CHGB | 0 | 0 | 0 | 0 | 1 | 0 | 0 |
| HPN-AS1 | 0 | 0 | 0 | 0 | 1 | 0 | 0 |
| FERMT1 | 0 | 0 | 0 | 0 | 1 | 0 | 0 |
| NEK2 | 0 | 0 | 0 | 0 | 1 | 1 | 0 |
| LOC101929473 | 0 | 0 | 0 | 0 | 1 | 0 | 0 |
| ANLN | 0 | 0 | 0 | 0 | 1 | 0 | 0 |
| GSN-AS1 | 0 | 0 | 0 | 0 | 1 | 0 | 0 |
| HPN | 0 | 0 | 0 | 0 | 1 | 0 | 0 |
| FHL5 | 0 | 0 | 0 | 0 | 1 | 0 | 0 |
| CACNG1 | 0 | 0 | 0 | 0 | 1 | 0 | 0 |
| FAM86B3P | 0 | 0 | 0 | 0 | 1 | 0 | 0 |
| TMEM215 | 0 | 0 | 0 | 0 | 1 | 0 | 0 |
| MIR1914 | 0 | 0 | 0 | 0 | 1 | 0 | 0 |
| LOC149684 | 0 | 0 | 0 | 0 | 1 | 0 | 0 |
| VGLL3 | 0 | 0 | 0 | 0 | 1 | 0 | 0 |
| SOX8 | 0 | 0 | 0 | 0 | 1 | 0 | 0 |
| ZNF90 | 0 | 0 | 0 | 0 | 1 | 0 | 0 |
| TMC6 | 0 | 0 | 0 | 0 | 1 | 0 | 0 |
| HIPK2 | 0 | 0 | 0 | 0 | 1 | 0 | 0 |
| LINC00567 | 0 | 0 | 0 | 0 | 1 | 0 | 0 |
| MAGI1-AS1 | 0 | 0 | 0 | 0 | 1 | 0 | 0 |
| TINAG | 0 | 0 | 0 | 0 | 1 | 0 | 0 |
| LOC100505635 | 0 | 0 | 0 | 0 | 1 | 0 | 0 |
| NSF | 0 | 0 | 0 | 0 | 1 | 0 | 0 |
| LOC101927410 | 0 | 0 | 0 | 0 | 1 | 0 | 0 |
| LOC100507616 | 0 | 0 | 0 | 0 | 1 | 0 | 0 |
| C10orf128 | 0 | 0 | 0 | 0 | 1 | 0 | 0 |
| DKFZP434K028 | 0 | 0 | 0 | 0 | 1 | 0 | 0 |
| SLC26A5 | 0 | 0 | 0 | 0 | 1 | 0 | 0 |
| INHA | 0 | 0 | 0 | 0 | 1 | 0 | 0 |
| ALG1L | 0 | 0 | 0 | 0 | 1 | 0 | 0 |
| KRT19 | 0 | 0 | 0 | 0 | 1 | 0 | 0 |
| TFCP2L1 | 0 | 0 | 0 | 0 | 1 | 0 | 0 |
| ELMO1-AS1 | 0 | 0 | 0 | 0 | 1 | 0 | 0 |
| C5orf38 | 0 | 0 | 0 | 0 | 1 | 0 | 0 |
| ANKRD22 | 0 | 0 | 0 | 0 | 1 | 0 | 0 |
| SHISA2 | 0 | 0 | 0 | 0 | 1 | 1 | 0 |
| FAM19A4 | 0 | 0 | 0 | 0 | 1 | 0 | 0 |
| ACP7 | 0 | 0 | 0 | 0 | 1 | 0 | 0 |
| C10orf90 | 0 | 0 | 0 | 0 | 1 | 0 | 0 |
| LOC100128554 | 0 | 0 | 0 | 0 | 1 | 0 | 0 |
| C7orf61 | 0 | 0 | 0 | 0 | 1 | 0 | 0 |
| CLCN1 | 0 | 0 | 0 | 0 | 1 | 0 | 0 |
| LOC441052 | 0 | 0 | 0 | 0 | 1 | 0 | 0 |
| KRT81 | 0 | 0 | 0 | 0 | 1 | 0 | 0 |
| LOC642943 | 0 | 0 | 0 | 0 | 1 | 0 | 0 |
| LOC400997 | 0 | 0 | 0 | 0 | 1 | 0 | 0 |
| SGK1 | 0 | 0 | 0 | 0 | 1 | 1 | 0 |
| TPTEP1 | 0 | 0 | 0 | 0 | 1 | 0 | 0 |
| DEPDC1 | 0 | 0 | 0 | 0 | 1 | 0 | 0 |
| VIP | 0 | 0 | 0 | 0 | 1 | 0 | 0 |
| LINCR-0003 | 0 | 0 | 0 | 0 | 1 | 0 | 0 |
| PROK2 | 0 | 0 | 0 | 0 | 1 | 0 | 0 |
| IL16 | 0 | 0 | 0 | 0 | 1 | 0 | 0 |
| LOC388780 | 0 | 0 | 0 | 0 | 1 | 0 | 0 |
| IRX1 | 0 | 0 | 0 | 0 | 1 | 0 | 0 |
| LOC105376360 | 0 | 0 | 0 | 0 | 1 | 0 | 0 |
| LOC100507472 | 0 | 0 | 0 | 0 | 1 | 0 | 0 |
| ADAMTS2 | 0 | 0 | 0 | 0 | 1 | 0 | 0 |
| SLC45A3 | 0 | 0 | 0 | 0 | 1 | 0 | 0 |
| PTER | 0 | 0 | 0 | 0 | 1 | 0 | 0 |
| LOC284933 | 0 | 0 | 0 | 0 | 1 | 0 | 0 |
| MROH5 | 0 | 0 | 0 | 0 | 1 | 0 | 0 |
| LINC01108 | 0 | 0 | 0 | 0 | 1 | 0 | 0 |
| IFNL2 | 0 | 0 | 0 | 0 | 1 | 0 | 0 |
| SPINT1 | 0 | 0 | 0 | 0 | 1 | 0 | 0 |
| CHADL | 0 | 0 | 0 | 0 | 1 | 0 | 0 |
| CCDC189 | 0 | 0 | 0 | 0 | 1 | 0 | 0 |
| ASB9P1 | 0 | 0 | 0 | 0 | 1 | 0 | 0 |
| H1FNT | 0 | 0 | 0 | 0 | 1 | 0 | 0 |
| MUC16 | 0 | 0 | 0 | 0 | 1 | 0 | 0 |
| CTD-2201E9.1 | 0 | 0 | 0 | 0 | 1 | 0 | 0 |
| CDR1 | 0 | 0 | 0 | 0 | 1 | 0 | 0 |
| MIR3612 | 0 | 0 | 0 | 0 | 1 | 0 | 0 |
| DLX5 | 0 | 0 | 0 | 0 | 1 | 0 | 0 |
| PNOC | 0 | 0 | 0 | 0 | 1 | 0 | 0 |
| IGF2 | 0 | 0 | 0 | 0 | 1 | 0 | 0 |
| ENPP6 | 0 | 0 | 0 | 0 | 1 | 0 | 0 |
| AZGP1 | 0 | 0 | 0 | 0 | 1 | 0 | 0 |
| RPLP0P2 | 0 | 0 | 0 | 0 | 1 | 0 | 0 |
| KIF2B | 0 | 0 | 0 | 0 | 1 | 0 | 0 |
| ZFPM2-AS1 | 0 | 0 | 0 | 0 | 1 | 0 | 0 |
| LOC100507144 | 0 | 0 | 0 | 0 | 1 | 0 | 0 |
| PCDH8 | 0 | 0 | 0 | 0 | 1 | 0 | 0 |
| SLED1 | 0 | 0 | 0 | 0 | 1 | 0 | 0 |
| LCN10 | 0 | 0 | 0 | 0 | 1 | 0 | 0 |
| LMNTD1 | 0 | 0 | 0 | 0 | 1 | 0 | 0 |
| SELENOP | 0 | 0 | 0 | 0 | 1 | 0 | 0 |
| LINC01470 | 0 | 0 | 0 | 0 | 1 | 0 | 0 |
| SFRP1 | 0 | 0 | 0 | 0 | 1 | 0 | 0 |
| CLDN7 | 0 | 0 | 0 | 0 | 1 | 0 | 0 |
| MYBPC2 | 0 | 0 | 0 | 0 | 1 | 0 | 0 |
| CBX3P2 | 0 | 0 | 0 | 0 | 1 | 0 | 0 |
| LINC01361 | 0 | 0 | 0 | 0 | 1 | 0 | 0 |
| TESPA1 | 0 | 0 | 0 | 0 | 1 | 0 | 0 |
| G0S2 | 0 | 0 | 0 | 0 | 1 | 0 | 0 |
| HECTD2-AS1 | 0 | 0 | 0 | 0 | 1 | 0 | 0 |
| ADORA2A-AS1 | 0 | 0 | 0 | 0 | 1 | 0 | 0 |
| LINC00390 | 0 | 0 | 0 | 0 | 1 | 0 | 0 |
| LINC01378 | 0 | 0 | 0 | 0 | 1 | 0 | 0 |
| LOC101929719 | 0 | 0 | 0 | 0 | 1 | 0 | 0 |
| RAB27B | 0 | 0 | 0 | 0 | 1 | 0 | 0 |
| RASL11B | 0 | 0 | 0 | 0 | 1 | 0 | 0 |
| SLC30A8 | 0 | 0 | 0 | 0 | 1 | 0 | 0 |
| ZNF341-AS1 | 0 | 0 | 0 | 0 | 1 | 0 | 0 |
| LMX1B | 0 | 0 | 0 | 0 | 1 | 0 | 0 |
| IP6K3 | 0 | 0 | 0 | 0 | 1 | 0 | 0 |
| LCN2 | 0 | 0 | 0 | 0 | 1 | 0 | 1 |
| RNASE2 | 0 | 0 | 0 | 0 | 1 | 0 | 0 |
| TAC3 | 0 | 0 | 0 | 0 | 1 | 0 | 0 |
| C17orf102 | 0 | 0 | 0 | 0 | 1 | 0 | 0 |
| LOC101927844 | 0 | 0 | 0 | 0 | 1 | 0 | 0 |
| TPH1 | 0 | 0 | 0 | 0 | 1 | 0 | 0 |
| EGR3 | 0 | 0 | 0 | 0 | 1 | 0 | 0 |
| TPO | 0 | 0 | 0 | 0 | 1 | 0 | 0 |
| LCN15 | 0 | 0 | 0 | 0 | 1 | 0 | 0 |
| SPTBN5 | 0 | 0 | 0 | 0 | 1 | 0 | 0 |
| TSPAN10 | 0 | 0 | 0 | 0 | 1 | 0 | 0 |
| VNN1 | 0 | 0 | 0 | 0 | 1 | 0 | 0 |
| MIR4534 | 0 | 0 | 0 | 0 | 1 | 0 | 0 |
| PKHD1L1 | 0 | 0 | 0 | 0 | 1 | 0 | 0 |
| LENEP | 0 | 0 | 0 | 0 | 1 | 0 | 0 |
| SLC5A11 | 0 | 0 | 0 | 0 | 1 | 0 | 0 |
| MIR3139 | 0 | 0 | 0 | 0 | 1 | 0 | 0 |
| DLX4 | 0 | 0 | 0 | 0 | 1 | 0 | 0 |
| IQGAP3 | 0 | 0 | 0 | 0 | 1 | 0 | 0 |
| LRP2 | 0 | 0 | 0 | 0 | 1 | 0 | 0 |
| RTKN | 0 | 0 | 0 | 0 | 1 | 0 | 0 |
| TPH2 | 0 | 0 | 0 | 0 | 1 | 0 | 0 |
| KLHDC7B | 0 | 0 | 0 | 0 | 1 | 0 | 0 |
| LOC101059915 | 0 | 0 | 0 | 0 | 1 | 0 | 0 |
| LINC01512 | 0 | 0 | 0 | 0 | 1 | 0 | 0 |
| HAPLN2 | 0 | 0 | 0 | 0 | 1 | 0 | 0 |
| OR2D3 | 0 | 0 | 0 | 0 | 1 | 0 | 0 |
| CR1 | 0 | 0 | 0 | 0 | 1 | 0 | 0 |
| TNN | 0 | 0 | 0 | 0 | 1 | 0 | 0 |
| LINC00926 | 0 | 0 | 0 | 0 | 1 | 0 | 0 |
| ADGRG5 | 0 | 0 | 0 | 0 | 1 | 0 | 0 |
| LAMA5-AS1 | 0 | 0 | 0 | 0 | 1 | 0 | 0 |
| NKX6-2 | 0 | 0 | 0 | 0 | 1 | 0 | 0 |
| LOC340090 | 0 | 0 | 0 | 0 | 1 | 0 | 0 |
| LRRC63 | 0 | 0 | 0 | 0 | 1 | 0 | 0 |
| MIR657 | 0 | 0 | 0 | 0 | 1 | 0 | 0 |
| F2 | 0 | 0 | 0 | 0 | 1 | 0 | 0 |
| ZIC3 | 0 | 0 | 0 | 0 | 1 | 0 | 0 |
| GRK7 | 0 | 0 | 0 | 0 | 1 | 0 | 0 |
| SCUBE1 | 0 | 0 | 0 | 0 | 1 | 0 | 0 |
| GPA33 | 0 | 0 | 0 | 0 | 1 | 0 | 1 |
| CCKAR | 0 | 0 | 0 | 0 | 1 | 0 | 0 |
| LOC100129940 | 0 | 0 | 0 | 0 | 1 | 0 | 0 |
| C4orf26 | 0 | 0 | 0 | 0 | 1 | 1 | 0 |
| SLN | 0 | 0 | 0 | 0 | 1 | 0 | 0 |
| GDNF | 0 | 0 | 0 | 0 | 1 | 0 | 0 |
| LGR5 | 0 | 0 | 0 | 0 | 1 | 0 | 0 |
| MIR4296 | 0 | 0 | 0 | 0 | 1 | 0 | 0 |
| ZBTB46-AS1 | 0 | 0 | 0 | 0 | 1 | 0 | 0 |
| PTPRQ | 0 | 0 | 0 | 0 | 1 | 0 | 0 |
| LOC105371789 | 0 | 0 | 0 | 0 | 1 | 0 | 0 |
| RAB3C | 0 | 0 | 0 | 0 | 1 | 0 | 0 |
| SFTPA2 | 0 | 0 | 0 | 0 | 1 | 0 | 0 |
| PTF1A | 0 | 0 | 0 | 0 | 1 | 0 | 0 |
| MIR4300HG | 0 | 0 | 0 | 0 | 1 | 0 | 0 |
| MIR4311 | 0 | 0 | 0 | 0 | 1 | 0 | 0 |
| LOC100506271 | 0 | 0 | 0 | 0 | 1 | 0 | 0 |
| C16orf90 | 0 | 0 | 0 | 0 | 1 | 0 | 0 |
| PRKXP1 | 0 | 0 | 0 | 0 | 1 | 0 | 0 |
| GDF2 | 0 | 0 | 0 | 0 | 1 | 0 | 0 |
| CA9 | 0 | 0 | 0 | 0 | 1 | 0 | 0 |
| LINC01088 | 0 | 0 | 0 | 0 | 1 | 0 | 0 |
| PRIMA1 | 0 | 0 | 0 | 0 | 1 | 0 | 0 |
| ST18 | 0 | 0 | 0 | 0 | 1 | 0 | 0 |
| OR7C1 | 0 | 0 | 0 | 0 | 1 | 0 | 0 |
| LOC100287072 | 0 | 0 | 0 | 0 | 1 | 0 | 0 |
| OR14I1 | 0 | 0 | 0 | 0 | 1 | 0 | 0 |
| LINC00514 | 0 | 0 | 0 | 0 | 1 | 0 | 0 |
| SNRPD2P2 | 0 | 0 | 0 | 0 | 1 | 0 | 0 |
| LINC00937 | 0 | 0 | 0 | 0 | 1 | 0 | 1 |
| KANK4 | 0 | 0 | 0 | 0 | 1 | 0 | 0 |
| FAM95A | 0 | 0 | 0 | 0 | 1 | 0 | 0 |
| ARL5C | 0 | 0 | 0 | 0 | 1 | 0 | 0 |
| PIEZO2 | 0 | 0 | 0 | 0 | 1 | 0 | 0 |
| MCCD1 | 0 | 0 | 0 | 0 | 1 | 0 | 0 |
| FOXR1 | 0 | 0 | 0 | 0 | 1 | 0 | 0 |
| PCSK6-AS1 | 0 | 0 | 0 | 0 | 1 | 0 | 0 |
| LOC100130331 | 0 | 0 | 0 | 0 | 1 | 0 | 0 |
| ADAM29 | 0 | 0 | 0 | 0 | 1 | 0 | 0 |
| DUSP4 | 0 | 0 | 0 | 0 | 1 | 0 | 0 |
| PCSK6 | 0 | 0 | 0 | 0 | 1 | 0 | 0 |
| C1orf195 | 0 | 0 | 0 | 0 | 1 | 0 | 0 |
| MIR6758 | 0 | 0 | 0 | 0 | 1 | 0 | 0 |
| FAM163A | 0 | 0 | 0 | 0 | 1 | 0 | 0 |
| CT55 | 0 | 0 | 0 | 0 | 1 | 0 | 0 |
| LINC00664 | 0 | 0 | 0 | 0 | 1 | 0 | 0 |
| TMEM235 | 0 | 0 | 0 | 0 | 1 | 0 | 0 |
| LOC101929613 | 0 | 0 | 0 | 0 | 1 | 0 | 0 |
| MIR6871 | 0 | 0 | 0 | 0 | 1 | 0 | 0 |
| LINC00320 | 0 | 0 | 0 | 0 | 1 | 0 | 0 |
| GFRA3 | 0 | 0 | 0 | 0 | 1 | 0 | 0 |
| MIR4479 | 0 | 0 | 0 | 0 | 1 | 0 | 0 |
| LINC01219 | 0 | 0 | 0 | 0 | 1 | 0 | 0 |
| MEIS1-AS2 | 0 | 0 | 0 | 0 | 1 | 0 | 0 |
| LDHAL6B | 0 | 0 | 0 | 0 | 1 | 0 | 0 |
| KCNJ15 | 0 | 0 | 0 | 0 | 1 | 0 | 0 |
| LOC101928738 | 0 | 0 | 0 | 0 | 1 | 0 | 0 |
| ASB2 | 0 | 0 | 0 | 0 | 1 | 0 | 0 |
| LOC102723895 | 0 | 0 | 0 | 0 | 1 | 0 | 0 |
| LINC01117 | 0 | 0 | 0 | 0 | 1 | 0 | 0 |
| ELANE | 0 | 0 | 0 | 0 | 1 | 0 | 0 |
| FAM95C | 0 | 0 | 0 | 0 | 1 | 0 | 0 |
| CALHM3 | 0 | 0 | 0 | 0 | 1 | 0 | 0 |
| CHRM5 | 0 | 0 | 0 | 0 | 1 | 0 | 0 |
| FERD3L | 0 | 0 | 0 | 0 | 1 | 0 | 0 |
| DHRS2 | 0 | 0 | 0 | 0 | 1 | 0 | 0 |
| NFE2 | 0 | 0 | 0 | 0 | 1 | 0 | 1 |
| C1orf94 | 0 | 0 | 0 | 0 | 1 | 0 | 0 |
| JAML | 0 | 0 | 0 | 0 | 1 | 0 | 0 |
| SNORD128 | 0 | 0 | 0 | 0 | 1 | 0 | 0 |
| LPO | 0 | 0 | 0 | 0 | 1 | 0 | 0 |
| MIR194-2HG | 0 | 0 | 0 | 0 | 1 | 0 | 0 |
| LINC00504 | 0 | 0 | 0 | 0 | 1 | 0 | 0 |
| BAAT | 0 | 0 | 0 | 0 | 1 | 0 | 0 |
| LOC100507388 | 0 | 0 | 0 | 0 | 1 | 0 | 0 |
| LOC440982 | 0 | 0 | 0 | 0 | 1 | 0 | 0 |
| RASGRP3 | 0 | 0 | 0 | 0 | 1 | 0 | 0 |
| LINC00609 | 0 | 0 | 0 | 0 | 1 | 0 | 0 |
| LOC101927960 | 0 | 0 | 0 | 0 | 1 | 0 | 0 |
| LINC01331 | 0 | 0 | 0 | 0 | 1 | 0 | 0 |
| LOC101927168 | 0 | 0 | 0 | 0 | 1 | 0 | 0 |
| NMRK2 | 0 | 0 | 0 | 0 | 1 | 0 | 0 |
| HOXD1 | 0 | 0 | 0 | 0 | 1 | 0 | 0 |
| LOC100130698 | 0 | 0 | 0 | 0 | 1 | 0 | 0 |
| CLEC18B | 0 | 0 | 0 | 0 | 1 | 0 | 0 |
| CEL | 0 | 0 | 0 | 0 | 1 | 0 | 0 |
| ANXA2P3 | 0 | 0 | 0 | 0 | 1 | 0 | 0 |
| LOC101929284 | 0 | 0 | 0 | 0 | 1 | 0 | 0 |
| DIO3OS | 0 | 0 | 0 | 0 | 1 | 0 | 0 |
| LOC101929341 | 0 | 0 | 0 | 0 | 1 | 0 | 0 |
| FAM151A | 0 | 0 | 0 | 0 | 1 | 0 | 0 |
| FAM66E | 0 | 0 | 0 | 0 | 1 | 0 | 0 |
| NXPH2 | 0 | 0 | 0 | 0 | 1 | 0 | 0 |
| LINC01494 | 0 | 0 | 0 | 0 | 1 | 0 | 0 |
| LOC100130370 | 0 | 0 | 0 | 0 | 1 | 0 | 0 |
| CBLN4 | 0 | 0 | 0 | 0 | 1 | 0 | 0 |
| LINC00398 | 0 | 0 | 0 | 0 | 1 | 0 | 0 |
| GIPR | 0 | 0 | 0 | 0 | 1 | 0 | 0 |
| C21orf91-OT1 | 0 | 0 | 0 | 0 | 1 | 0 | 0 |
| NMUR2 | 0 | 0 | 0 | 0 | 1 | 0 | 0 |
| BCRP3 | 0 | 0 | 0 | 0 | 1 | 0 | 0 |
| TSPAN18 | 0 | 0 | 0 | 0 | 1 | 0 | 0 |
| RORC | 0 | 0 | 0 | 0 | 1 | 0 | 0 |
| CD300LG | 0 | 0 | 0 | 0 | 1 | 0 | 0 |
| HIST1H1T | 0 | 0 | 0 | 0 | 1 | 0 | 0 |
| MUC5AC | 0 | 0 | 0 | 0 | 1 | 0 | 0 |
| SLC17A6 | 0 | 0 | 0 | 0 | 1 | 0 | 0 |
| KRT8P41 | 0 | 0 | 0 | 0 | 1 | 0 | 0 |
| CD38 | 0 | 0 | 0 | 0 | 1 | 0 | 1 |
| MIR4513 | 0 | 0 | 0 | 0 | 1 | 0 | 0 |
| MTRNR2L10 | 0 | 0 | 0 | 0 | 0 | 1 | 0 |
| INMT | 0 | 0 | 0 | 0 | 0 | 1 | 0 |
| PPIEL | 0 | 0 | 0 | 0 | 0 | 1 | 0 |
| RAB3B | 0 | 0 | 0 | 0 | 0 | 1 | 0 |
| IKZF3 | 0 | 0 | 0 | 0 | 0 | 1 | 0 |
| GATM-AS1 | 0 | 0 | 0 | 0 | 0 | 1 | 0 |
| PDE6A | 0 | 0 | 0 | 0 | 0 | 1 | 0 |
| MREG | 0 | 0 | 0 | 0 | 0 | 1 | 0 |
| IDS | 0 | 0 | 0 | 0 | 0 | 1 | 0 |
| LINC00649 | 0 | 0 | 0 | 0 | 0 | 1 | 0 |
| METTL21A | 0 | 0 | 0 | 0 | 0 | 1 | 0 |
| SLC43A2 | 0 | 0 | 0 | 0 | 0 | 1 | 0 |
| EEF2K | 0 | 0 | 0 | 0 | 0 | 1 | 0 |
| FXN | 0 | 0 | 0 | 0 | 0 | 1 | 0 |
| ACBD7 | 0 | 0 | 0 | 0 | 0 | 1 | 0 |
| IFNLR1 | 0 | 0 | 0 | 0 | 0 | 1 | 0 |
| ZNF483 | 0 | 0 | 0 | 0 | 0 | 1 | 0 |
| FGF5 | 0 | 0 | 0 | 0 | 0 | 1 | 0 |
| FBLIM1 | 0 | 0 | 0 | 0 | 0 | 1 | 0 |
| ATP5E | 0 | 0 | 0 | 0 | 0 | 1 | 0 |
| MTRNR2L2 | 0 | 0 | 0 | 0 | 0 | 1 | 0 |
| GNG4 | 0 | 0 | 0 | 0 | 0 | 1 | 0 |
| LOC284260 | 0 | 0 | 0 | 0 | 0 | 1 | 0 |
| CCL5 | 0 | 0 | 0 | 0 | 0 | 1 | 0 |
| GLIPR1L2 | 0 | 0 | 0 | 0 | 0 | 1 | 0 |
| PRELP | 0 | 0 | 0 | 0 | 0 | 1 | 0 |
| SIX4 | 0 | 0 | 0 | 0 | 0 | 1 | 0 |
| CSDE1 | 0 | 0 | 0 | 0 | 0 | 1 | 0 |
| C21orf62 | 0 | 0 | 0 | 0 | 0 | 1 | 0 |
| TMEM154 | 0 | 0 | 0 | 0 | 0 | 1 | 0 |
| LOC286186 | 0 | 0 | 0 | 0 | 0 | 1 | 0 |
| CACNG8 | 0 | 0 | 0 | 0 | 0 | 1 | 0 |
| VSIG1 | 0 | 0 | 0 | 0 | 0 | 1 | 0 |
| MOG | 0 | 0 | 0 | 0 | 0 | 1 | 0 |
| UBE2Q2P1 | 0 | 0 | 0 | 0 | 0 | 1 | 0 |
| CCDC122 | 0 | 0 | 0 | 0 | 0 | 1 | 0 |
| PPIL6 | 0 | 0 | 0 | 0 | 0 | 1 | 0 |
| ELMOD1 | 0 | 0 | 0 | 0 | 0 | 1 | 0 |
| CYP4V2 | 0 | 0 | 0 | 0 | 0 | 1 | 0 |
| NXN | 0 | 0 | 0 | 0 | 0 | 1 | 0 |
| ZFP42 | 0 | 0 | 0 | 0 | 0 | 1 | 0 |
| SPRED1 | 0 | 0 | 0 | 0 | 0 | 1 | 0 |
| ZYG11A | 0 | 0 | 0 | 0 | 0 | 1 | 0 |
| TBXA2R | 0 | 0 | 0 | 0 | 0 | 1 | 0 |
| KIAA1456 | 0 | 0 | 0 | 0 | 0 | 1 | 0 |
| ZNF665 | 0 | 0 | 0 | 0 | 0 | 1 | 0 |
| MTRNR2L4 | 0 | 0 | 0 | 0 | 0 | 1 | 0 |
| PJA2 | 0 | 0 | 0 | 0 | 0 | 1 | 0 |
| LOC283335 | 0 | 0 | 0 | 0 | 0 | 1 | 0 |
| HTRA4 | 0 | 0 | 0 | 0 | 0 | 1 | 0 |
| IRF2BPL | 0 | 0 | 0 | 0 | 0 | 1 | 0 |
| LOC100287314 | 0 | 0 | 0 | 0 | 0 | 1 | 0 |
| FKBP14 | 0 | 0 | 0 | 0 | 0 | 1 | 0 |
| LOC90834 | 0 | 0 | 0 | 0 | 0 | 1 | 0 |
| LOC100129269 | 0 | 0 | 0 | 0 | 0 | 1 | 0 |
| AP1S3 | 0 | 0 | 0 | 0 | 0 | 1 | 0 |
| LOC100288069 | 0 | 0 | 0 | 0 | 0 | 1 | 0 |
| BVES | 0 | 0 | 0 | 0 | 0 | 1 | 0 |
| CEP41 | 0 | 0 | 0 | 0 | 0 | 1 | 0 |
| PTPN14 | 0 | 0 | 0 | 0 | 0 | 1 | 0 |
| ZNF713 | 0 | 0 | 0 | 0 | 0 | 1 | 0 |
| AFMID | 0 | 0 | 0 | 0 | 0 | 1 | 0 |
| OR7D2 | 0 | 0 | 0 | 0 | 0 | 1 | 0 |
| MAP1LC3C | 0 | 0 | 0 | 0 | 0 | 1 | 0 |
| LOC100128288 | 0 | 0 | 0 | 0 | 0 | 1 | 0 |
| ZNF793 | 0 | 0 | 0 | 0 | 0 | 1 | 1 |
| PTK6 | 0 | 0 | 0 | 0 | 0 | 1 | 0 |
| NEK5 | 0 | 0 | 0 | 0 | 0 | 1 | 0 |
| TMEM213 | 0 | 0 | 0 | 0 | 0 | 1 | 0 |
| ATCAY | 0 | 0 | 0 | 0 | 0 | 1 | 0 |
| ATP5I | 0 | 0 | 0 | 0 | 0 | 1 | 0 |
| C1orf210 | 0 | 0 | 0 | 0 | 0 | 1 | 0 |
| MAPRE1 | 0 | 0 | 0 | 0 | 0 | 1 | 0 |
| LRRN4CL | 0 | 0 | 0 | 0 | 0 | 1 | 0 |
| LOC100506085 | 0 | 0 | 0 | 0 | 0 | 1 | 0 |
| METTL8 | 0 | 0 | 0 | 0 | 0 | 1 | 0 |
| MTRNR2L3 | 0 | 0 | 0 | 0 | 0 | 1 | 0 |
| RNF207 | 0 | 0 | 0 | 0 | 0 | 1 | 0 |
| RABL5 | 0 | 0 | 0 | 0 | 0 | 1 | 0 |
| TMEM236 | 0 | 0 | 0 | 0 | 0 | 1 | 0 |
| JPX | 0 | 0 | 0 | 0 | 0 | 1 | 0 |
| ZNF716 | 0 | 0 | 0 | 0 | 0 | 1 | 0 |
| TFDP2 | 0 | 0 | 0 | 0 | 0 | 1 | 0 |
| NCMAP | 0 | 0 | 0 | 0 | 0 | 1 | 0 |
| RAB42 | 0 | 0 | 0 | 0 | 0 | 1 | 0 |
| ZNF818P | 0 | 0 | 0 | 0 | 0 | 1 | 0 |
| ZC3H12D | 0 | 0 | 0 | 0 | 0 | 1 | 0 |
| LOC440300 | 0 | 0 | 0 | 0 | 0 | 1 | 0 |
| PXMP4 | 0 | 0 | 0 | 0 | 0 | 1 | 0 |
| PAPL | 0 | 0 | 0 | 0 | 0 | 1 | 0 |
| ZFAS1 | 0 | 0 | 0 | 0 | 0 | 1 | 0 |
| PNPO | 0 | 0 | 0 | 0 | 0 | 1 | 0 |
| PTPRG-AS1 | 0 | 0 | 0 | 0 | 0 | 1 | 0 |
| WDR1 | 0 | 0 | 0 | 0 | 0 | 1 | 0 |
| SEPT14 | 0 | 0 | 0 | 0 | 0 | 1 | 0 |
| BCAP31 | 0 | 0 | 0 | 0 | 0 | 1 | 0 |
| ANKRD16 | 0 | 0 | 0 | 0 | 0 | 1 | 0 |
| SCD5 | 0 | 0 | 0 | 0 | 0 | 1 | 0 |
| C17orf75 | 0 | 0 | 0 | 0 | 0 | 1 | 0 |
| FAM227A | 0 | 0 | 0 | 0 | 0 | 1 | 0 |
| PNMA2 | 0 | 0 | 0 | 0 | 0 | 1 | 0 |
| ZNF667 | 0 | 0 | 0 | 0 | 0 | 1 | 0 |
| EMP2 | 0 | 0 | 0 | 0 | 0 | 1 | 0 |
| RFPL1-AS1 | 0 | 0 | 0 | 0 | 0 | 1 | 0 |
| SCAI | 0 | 0 | 0 | 0 | 0 | 1 | 0 |
| KIAA1324 | 0 | 0 | 0 | 0 | 0 | 1 | 0 |
| PACS2 | 0 | 0 | 0 | 0 | 0 | 1 | 0 |
| EXPH5 | 0 | 0 | 0 | 0 | 0 | 1 | 0 |
| DYNC1LI1 | 0 | 0 | 0 | 0 | 0 | 1 | 0 |
| DNAL1 | 0 | 0 | 0 | 0 | 0 | 1 | 0 |
| TRIM65 | 0 | 0 | 0 | 0 | 0 | 1 | 0 |
| PRND | 0 | 0 | 0 | 0 | 0 | 1 | 0 |
| CHRNB1 | 0 | 0 | 0 | 0 | 0 | 1 | 0 |
| ZNF492 | 0 | 0 | 0 | 0 | 0 | 1 | 0 |
| PGM5P2 | 0 | 0 | 0 | 0 | 0 | 1 | 1 |
| BRIP1 | 0 | 0 | 0 | 0 | 0 | 1 | 0 |
| IBA57 | 0 | 0 | 0 | 0 | 0 | 1 | 0 |
| POU5F1 | 0 | 0 | 0 | 0 | 0 | 1 | 0 |
| SLC5A5 | 0 | 0 | 0 | 0 | 0 | 1 | 0 |
| GDPD1 | 0 | 0 | 0 | 0 | 0 | 1 | 0 |
| PDP2 | 0 | 0 | 0 | 0 | 0 | 1 | 0 |
| KCNJ5 | 0 | 0 | 0 | 0 | 0 | 1 | 0 |
| PLEKHH2 | 0 | 0 | 0 | 0 | 0 | 1 | 0 |
| LRRC2 | 0 | 0 | 0 | 0 | 0 | 1 | 0 |
| LOC284950 | 0 | 0 | 0 | 0 | 0 | 1 | 0 |
| C2orf91 | 0 | 0 | 0 | 0 | 0 | 1 | 0 |
| AK3 | 0 | 0 | 0 | 0 | 0 | 1 | 0 |
| GGT6 | 0 | 0 | 0 | 0 | 0 | 1 | 0 |
| SEC14L1 | 0 | 0 | 0 | 0 | 0 | 1 | 0 |
| PGAM5 | 0 | 0 | 0 | 0 | 0 | 1 | 0 |
| KIAA1875 | 0 | 0 | 0 | 0 | 0 | 1 | 0 |
| SPG21 | 0 | 0 | 0 | 0 | 0 | 1 | 0 |
| LOC100128338 | 0 | 0 | 0 | 0 | 0 | 1 | 0 |
| CYFIP2 | 0 | 0 | 0 | 0 | 0 | 1 | 0 |
| UBL5 | 0 | 0 | 0 | 0 | 0 | 1 | 0 |
| C1orf56 | 0 | 0 | 0 | 0 | 0 | 1 | 0 |
| CCNI | 0 | 0 | 0 | 0 | 0 | 1 | 1 |
| SGCB | 0 | 0 | 0 | 0 | 0 | 1 | 0 |
| ARHGEF26-AS1 | 0 | 0 | 0 | 0 | 0 | 1 | 0 |
| FPR2 | 0 | 0 | 0 | 0 | 0 | 1 | 0 |
| ARF1 | 0 | 0 | 0 | 0 | 0 | 1 | 0 |
| OPA3 | 0 | 0 | 0 | 0 | 0 | 1 | 0 |
| SLC4A8 | 0 | 0 | 0 | 0 | 0 | 1 | 0 |
| AARS2 | 0 | 0 | 0 | 0 | 0 | 1 | 0 |
| LOC283299 | 0 | 0 | 0 | 0 | 0 | 1 | 0 |
| LIN28A | 0 | 0 | 0 | 0 | 0 | 1 | 0 |
| MTRNR2L8 | 0 | 0 | 0 | 0 | 0 | 1 | 0 |
| TMEM136 | 0 | 0 | 0 | 0 | 0 | 1 | 0 |
| NR1H2 | 0 | 0 | 0 | 0 | 0 | 1 | 1 |
| MYLK3 | 0 | 0 | 0 | 0 | 0 | 1 | 0 |
| CA5B | 0 | 0 | 0 | 0 | 0 | 1 | 0 |
| C3orf72 | 0 | 0 | 0 | 0 | 0 | 1 | 0 |
| CRX | 0 | 0 | 0 | 0 | 0 | 1 | 0 |
| FAM73A | 0 | 0 | 0 | 0 | 0 | 1 | 0 |
| MANEAL | 0 | 0 | 0 | 0 | 0 | 1 | 0 |
| LINC00294 | 0 | 0 | 0 | 0 | 0 | 1 | 0 |
| WDR92 | 0 | 0 | 0 | 0 | 0 | 1 | 0 |
| FOXK1 | 0 | 0 | 0 | 0 | 0 | 1 | 0 |
| TSTD3 | 0 | 0 | 0 | 0 | 0 | 1 | 0 |
| SIGLEC8 | 0 | 0 | 0 | 0 | 0 | 1 | 0 |
| LOC728606 | 0 | 0 | 0 | 0 | 0 | 1 | 0 |
| ZNF714 | 0 | 0 | 0 | 0 | 0 | 1 | 0 |
| WDR17 | 0 | 0 | 0 | 0 | 0 | 1 | 0 |
| MAP7D3 | 0 | 0 | 0 | 0 | 0 | 1 | 0 |
| TYW5 | 0 | 0 | 0 | 0 | 0 | 1 | 0 |
| AQP6 | 0 | 0 | 0 | 0 | 0 | 1 | 0 |
| GLUD1P7 | 0 | 0 | 0 | 0 | 0 | 1 | 0 |
| TMEM130 | 0 | 0 | 0 | 0 | 0 | 1 | 0 |
| LARS2-AS1 | 0 | 0 | 0 | 0 | 0 | 1 | 0 |
| PPARA | 0 | 0 | 0 | 0 | 0 | 1 | 0 |
| DZIP3 | 0 | 0 | 0 | 0 | 0 | 1 | 0 |
| MTX3 | 0 | 0 | 0 | 0 | 0 | 1 | 0 |
| LOC728558 | 0 | 0 | 0 | 0 | 0 | 1 | 0 |
| LOC100287792 | 0 | 0 | 0 | 0 | 0 | 1 | 0 |
| C7orf55 | 0 | 0 | 0 | 0 | 0 | 1 | 0 |
| UTY | 0 | 0 | 0 | 0 | 0 | 1 | 0 |
| IFITM3 | 0 | 0 | 0 | 0 | 0 | 1 | 0 |
| PCDHB9 | 0 | 0 | 0 | 0 | 0 | 1 | 0 |
| CD93 | 0 | 0 | 0 | 0 | 0 | 1 | 0 |
| NOS1 | 0 | 0 | 0 | 0 | 0 | 1 | 0 |
| PLEK | 0 | 0 | 0 | 0 | 0 | 1 | 1 |
| LOC100128531 | 0 | 0 | 0 | 0 | 0 | 1 | 0 |
| ANKS4B | 0 | 0 | 0 | 0 | 0 | 1 | 0 |
| ARNTL2 | 0 | 0 | 0 | 0 | 0 | 1 | 0 |
| SLC7A14 | 0 | 0 | 0 | 0 | 0 | 1 | 0 |
| AKIP1 | 0 | 0 | 0 | 0 | 0 | 1 | 0 |
| CYP27C1 | 0 | 0 | 0 | 0 | 0 | 1 | 0 |
| FKBP8 | 0 | 0 | 0 | 0 | 0 | 1 | 1 |
| RAD23B | 0 | 0 | 0 | 0 | 0 | 1 | 0 |
| ORC6 | 0 | 0 | 0 | 0 | 0 | 1 | 1 |
| FBXO45 | 0 | 0 | 0 | 0 | 0 | 1 | 0 |
| FAM200B | 0 | 0 | 0 | 0 | 0 | 1 | 0 |
| NUP43 | 0 | 0 | 0 | 0 | 0 | 1 | 0 |
| SMAD5 | 0 | 0 | 0 | 0 | 0 | 1 | 0 |
| BMP8A | 0 | 0 | 0 | 0 | 0 | 1 | 0 |
| HEXIM1 | 0 | 0 | 0 | 0 | 0 | 1 | 0 |
| DNAJB12 | 0 | 0 | 0 | 0 | 0 | 1 | 1 |
| S1PR2 | 0 | 0 | 0 | 0 | 0 | 1 | 0 |
| ZNF542 | 0 | 0 | 0 | 0 | 0 | 1 | 0 |
| ZNF69 | 0 | 0 | 0 | 0 | 0 | 1 | 0 |
| KIAA1467 | 0 | 0 | 0 | 0 | 0 | 1 | 0 |
| EXOSC6 | 0 | 0 | 0 | 0 | 0 | 1 | 0 |
| DNAJC22 | 0 | 0 | 0 | 0 | 0 | 1 | 0 |
| MTRNR2L1 | 0 | 0 | 0 | 0 | 0 | 1 | 0 |
| VSTM4 | 0 | 0 | 0 | 0 | 0 | 1 | 0 |
| ADAM17 | 0 | 0 | 0 | 0 | 0 | 1 | 0 |
| SBF2-AS1 | 0 | 0 | 0 | 0 | 0 | 1 | 0 |
| DUSP19 | 0 | 0 | 0 | 0 | 0 | 1 | 0 |
| PCDH11Y | 0 | 0 | 0 | 0 | 0 | 1 | 0 |
| NUDT19 | 0 | 0 | 0 | 0 | 0 | 1 | 0 |
| DTD2 | 0 | 0 | 0 | 0 | 0 | 1 | 0 |
| BHMT2 | 0 | 0 | 0 | 0 | 0 | 1 | 0 |
| TMEM241 | 0 | 0 | 0 | 0 | 0 | 1 | 1 |
| CALCOCO2 | 0 | 0 | 0 | 0 | 0 | 1 | 0 |
| AMPD2 | 0 | 0 | 0 | 0 | 0 | 1 | 0 |
| FTH1P3 | 0 | 0 | 0 | 0 | 0 | 1 | 0 |
| EIF4H | 0 | 0 | 0 | 0 | 0 | 1 | 0 |
| GNPNAT1 | 0 | 0 | 0 | 0 | 0 | 1 | 0 |
| RPS6KA6 | 0 | 0 | 0 | 0 | 0 | 1 | 0 |
| FOXRED2 | 0 | 0 | 0 | 0 | 0 | 1 | 0 |
| PCDHA9 | 0 | 0 | 0 | 0 | 0 | 1 | 0 |
| ICA1L | 0 | 0 | 0 | 0 | 0 | 1 | 0 |
| S100A8 | 0 | 0 | 0 | 0 | 0 | 1 | 0 |
| DNAJB6 | 0 | 0 | 0 | 0 | 0 | 1 | 1 |
| DSCR6 | 0 | 0 | 0 | 0 | 0 | 1 | 0 |
| IL10RB | 0 | 0 | 0 | 0 | 0 | 1 | 0 |
| THBD | 0 | 0 | 0 | 0 | 0 | 1 | 0 |
| SLC25A15 | 0 | 0 | 0 | 0 | 0 | 1 | 0 |
| IGFBPL1 | 0 | 0 | 0 | 0 | 0 | 1 | 0 |
| LRIF1 | 0 | 0 | 0 | 0 | 0 | 1 | 0 |
| KATNBL1 | 0 | 0 | 0 | 0 | 0 | 1 | 0 |
| ZNF566 | 0 | 0 | 0 | 0 | 0 | 1 | 0 |
| ATP8B1 | 0 | 0 | 0 | 0 | 0 | 1 | 1 |
| C1orf86 | 0 | 0 | 0 | 0 | 0 | 1 | 0 |
| LRRFIP1 | 0 | 0 | 0 | 0 | 0 | 1 | 0 |
| LOC284023 | 0 | 0 | 0 | 0 | 0 | 1 | 0 |
| CCDC113 | 0 | 0 | 0 | 0 | 0 | 1 | 0 |
| PDIA3 | 0 | 0 | 0 | 0 | 0 | 1 | 0 |
| SKAP2 | 0 | 0 | 0 | 0 | 0 | 1 | 0 |
| BCL2L15 | 0 | 0 | 0 | 0 | 0 | 1 | 0 |
| CTSS | 0 | 0 | 0 | 0 | 0 | 1 | 0 |
| FLNA | 0 | 0 | 0 | 0 | 0 | 1 | 0 |
| IGBP1 | 0 | 0 | 0 | 0 | 0 | 1 | 0 |
| LINC00663 | 0 | 0 | 0 | 0 | 0 | 1 | 0 |
| DBT | 0 | 0 | 0 | 0 | 0 | 1 | 0 |
| AKIRIN1 | 0 | 0 | 0 | 0 | 0 | 1 | 0 |
| IL1B | 0 | 0 | 0 | 0 | 0 | 1 | 1 |
| PLEKHO2 | 0 | 0 | 0 | 0 | 0 | 1 | 0 |
| VAMP3 | 0 | 0 | 0 | 0 | 0 | 1 | 0 |
| RAD1 | 0 | 0 | 0 | 0 | 0 | 1 | 0 |
| TNFAIP6 | 0 | 0 | 0 | 0 | 0 | 1 | 1 |
| TNIP1 | 0 | 0 | 0 | 0 | 0 | 1 | 0 |
| COX7B | 0 | 0 | 0 | 0 | 0 | 1 | 0 |
| TMF1 | 0 | 0 | 0 | 0 | 0 | 1 | 0 |
| HIST2H2AC | 0 | 0 | 0 | 0 | 0 | 1 | 0 |
| HNRNPL | 0 | 0 | 0 | 0 | 0 | 1 | 0 |
| CHP2 | 0 | 0 | 0 | 0 | 0 | 1 | 0 |
| PSME3 | 0 | 0 | 0 | 0 | 0 | 1 | 1 |
| PUM2 | 0 | 0 | 0 | 0 | 0 | 1 | 0 |
| LOC400891 | 0 | 0 | 0 | 0 | 0 | 1 | 0 |
| MRI1 | 0 | 0 | 0 | 0 | 0 | 1 | 0 |
| GP2 | 0 | 0 | 0 | 0 | 0 | 1 | 0 |
| GPLD1 | 0 | 0 | 0 | 0 | 0 | 1 | 0 |
| MTRNR2L6 | 0 | 0 | 0 | 0 | 0 | 1 | 0 |
| GRM6 | 0 | 0 | 0 | 0 | 0 | 1 | 0 |
| CD177 | 0 | 0 | 0 | 0 | 0 | 1 | 0 |
| AKAP5 | 0 | 0 | 0 | 0 | 0 | 1 | 0 |
| SYNGR2 | 0 | 0 | 0 | 0 | 0 | 1 | 0 |
| BAG6 | 0 | 0 | 0 | 0 | 0 | 1 | 1 |
| SLC35E2 | 0 | 0 | 0 | 0 | 0 | 1 | 0 |
| ELL | 0 | 0 | 0 | 0 | 0 | 1 | 0 |
| TNFAIP8L3 | 0 | 0 | 0 | 0 | 0 | 1 | 0 |
| NUP98 | 0 | 0 | 0 | 0 | 0 | 1 | 0 |
| DKFZP434L187 | 0 | 0 | 0 | 0 | 0 | 1 | 0 |
| TRIM59 | 0 | 0 | 0 | 0 | 0 | 1 | 0 |
| ABHD2 | 0 | 0 | 0 | 0 | 0 | 1 | 1 |
| OR51E2 | 0 | 0 | 0 | 0 | 0 | 1 | 0 |
| C3orf33 | 0 | 0 | 0 | 0 | 0 | 1 | 0 |
| ATP6V0C | 0 | 0 | 0 | 0 | 0 | 1 | 1 |
| ATP6AP2 | 0 | 0 | 0 | 0 | 0 | 1 | 0 |
| SF3B2 | 0 | 0 | 0 | 0 | 0 | 1 | 0 |
| TMEM192 | 0 | 0 | 0 | 0 | 0 | 1 | 0 |
| PTGS2 | 0 | 0 | 0 | 0 | 0 | 1 | 0 |
| TUBA3FP | 0 | 0 | 0 | 0 | 0 | 1 | 0 |
| INADL | 0 | 0 | 0 | 0 | 0 | 1 | 0 |
| DBNL | 0 | 0 | 0 | 0 | 0 | 1 | 1 |
| IL10 | 0 | 0 | 0 | 0 | 0 | 1 | 0 |
| ITIH5 | 0 | 0 | 0 | 0 | 0 | 1 | 0 |
| FTH1 | 0 | 0 | 0 | 0 | 0 | 1 | 0 |
| TPM4 | 0 | 0 | 0 | 0 | 0 | 1 | 0 |
| PPP1R15B | 0 | 0 | 0 | 0 | 0 | 1 | 0 |
| BRAT1 | 0 | 0 | 0 | 0 | 0 | 1 | 1 |
| LINC00598 | 0 | 0 | 0 | 0 | 0 | 1 | 0 |
| PARK2 | 0 | 0 | 0 | 0 | 0 | 1 | 0 |
| RNF13 | 0 | 0 | 0 | 0 | 0 | 1 | 0 |
| ZNF260 | 0 | 0 | 0 | 0 | 0 | 1 | 0 |
| TPTE2P1 | 0 | 0 | 0 | 0 | 0 | 1 | 0 |
| C14orf2 | 0 | 0 | 0 | 0 | 0 | 1 | 0 |
| LOC284551 | 0 | 0 | 0 | 0 | 0 | 1 | 0 |
| DUSP6 | 0 | 0 | 0 | 0 | 0 | 1 | 0 |
| ZNF681 | 0 | 0 | 0 | 0 | 0 | 1 | 0 |
| LPIN3 | 0 | 0 | 0 | 0 | 0 | 1 | 0 |
| C9orf85 | 0 | 0 | 0 | 0 | 0 | 1 | 0 |
| TMEM181 | 0 | 0 | 0 | 0 | 0 | 1 | 0 |
| TMEM19 | 0 | 0 | 0 | 0 | 0 | 1 | 0 |
| STX4 | 0 | 0 | 0 | 0 | 0 | 1 | 0 |
| LOC151475 | 0 | 0 | 0 | 0 | 0 | 1 | 0 |
| HCAR2 | 0 | 0 | 0 | 0 | 0 | 1 | 0 |
| TTC39C | 0 | 0 | 0 | 0 | 0 | 1 | 0 |
| KDM6A | 0 | 0 | 0 | 0 | 0 | 1 | 0 |
| CD82 | 0 | 0 | 0 | 0 | 0 | 1 | 0 |
| CEBPD | 0 | 0 | 0 | 0 | 0 | 1 | 0 |
| KIF3A | 0 | 0 | 0 | 0 | 0 | 1 | 0 |
| LINC00665 | 0 | 0 | 0 | 0 | 0 | 1 | 0 |
| ARHGAP15 | 0 | 0 | 0 | 0 | 0 | 1 | 0 |
| EIF4A1 | 0 | 0 | 0 | 0 | 0 | 1 | 0 |
| ATP1B2 | 0 | 0 | 0 | 0 | 0 | 1 | 0 |
| XRCC6 | 0 | 0 | 0 | 0 | 0 | 1 | 0 |
| MAPK14 | 0 | 0 | 0 | 0 | 0 | 1 | 1 |
| DCUN1D2 | 0 | 0 | 0 | 0 | 0 | 1 | 0 |
| LOC100130954 | 0 | 0 | 0 | 0 | 0 | 1 | 0 |
| RNF40 | 0 | 0 | 0 | 0 | 0 | 1 | 0 |
| NFKB2 | 0 | 0 | 0 | 0 | 0 | 1 | 0 |
| FAM157B | 0 | 0 | 0 | 0 | 0 | 1 | 0 |
| HCAR3 | 0 | 0 | 0 | 0 | 0 | 1 | 0 |
| CST7 | 0 | 0 | 0 | 0 | 0 | 1 | 0 |
| AXL | 0 | 0 | 0 | 0 | 0 | 1 | 0 |
| CARD16 | 0 | 0 | 0 | 0 | 0 | 1 | 0 |
| PTAFR | 0 | 0 | 0 | 0 | 0 | 1 | 1 |
| METTL2B | 0 | 0 | 0 | 0 | 0 | 1 | 0 |
| NFE2L2 | 0 | 0 | 0 | 0 | 0 | 1 | 0 |
| FGL2 | 0 | 0 | 0 | 0 | 0 | 1 | 0 |
| SNW1 | 0 | 0 | 0 | 0 | 0 | 1 | 0 |
| SWSAP1 | 0 | 0 | 0 | 0 | 0 | 1 | 0 |
| ENTPD3-AS1 | 0 | 0 | 0 | 0 | 0 | 1 | 0 |
| CNEP1R1 | 0 | 0 | 0 | 0 | 0 | 1 | 0 |
| GLUL | 0 | 0 | 0 | 0 | 0 | 1 | 1 |
| DNASE1 | 0 | 0 | 0 | 0 | 0 | 1 | 0 |
| SURF4 | 0 | 0 | 0 | 0 | 0 | 1 | 0 |
| C19orf35 | 0 | 0 | 0 | 0 | 0 | 1 | 0 |
| LOH12CR2 | 0 | 0 | 0 | 0 | 0 | 1 | 0 |
| P4HB | 0 | 0 | 0 | 0 | 0 | 1 | 0 |
| ROMO1 | 0 | 0 | 0 | 0 | 0 | 1 | 0 |
| ADAM10 | 0 | 0 | 0 | 0 | 0 | 1 | 0 |
| PTMA | 0 | 0 | 0 | 0 | 0 | 1 | 0 |
| GTPBP3 | 0 | 0 | 0 | 0 | 0 | 1 | 0 |
| CNNM3 | 0 | 0 | 0 | 0 | 0 | 1 | 0 |
| DPYSL5 | 0 | 0 | 0 | 0 | 0 | 1 | 0 |
| CHPF2 | 0 | 0 | 0 | 0 | 0 | 1 | 0 |
| HSPA8 | 0 | 0 | 0 | 0 | 0 | 1 | 0 |
| LOC727896 | 0 | 0 | 0 | 0 | 0 | 1 | 0 |
| RAB11FIP4 | 0 | 0 | 0 | 0 | 0 | 1 | 0 |
| SULT2A1 | 0 | 0 | 0 | 0 | 0 | 1 | 0 |
| ATP2B1 | 0 | 0 | 0 | 0 | 0 | 1 | 0 |
| DPY19L1P1 | 0 | 0 | 0 | 0 | 0 | 1 | 0 |
| CIB1 | 0 | 0 | 0 | 0 | 0 | 1 | 0 |
| SCAMP2 | 0 | 0 | 0 | 0 | 0 | 1 | 0 |
| HDLBP | 0 | 0 | 0 | 0 | 0 | 1 | 0 |
| SOD2 | 0 | 0 | 0 | 0 | 0 | 1 | 0 |
| TIMP1 | 0 | 0 | 0 | 0 | 0 | 1 | 1 |
| EIF3A | 0 | 0 | 0 | 0 | 0 | 1 | 0 |
| SLC22A18 | 0 | 0 | 0 | 0 | 0 | 1 | 1 |
| LPCAT1 | 0 | 0 | 0 | 0 | 0 | 1 | 0 |
| GAK | 0 | 0 | 0 | 0 | 0 | 1 | 0 |
| LOC100505876 | 0 | 0 | 0 | 0 | 0 | 1 | 0 |
| PPIG | 0 | 0 | 0 | 0 | 0 | 1 | 1 |
| IL1RN | 0 | 0 | 0 | 0 | 0 | 1 | 0 |
| IL8 | 0 | 0 | 0 | 0 | 0 | 1 | 0 |
| BCDIN3D-AS1 | 0 | 0 | 0 | 0 | 0 | 1 | 0 |
| ATP6V0D2 | 0 | 0 | 0 | 0 | 0 | 1 | 0 |
| BTF3 | 0 | 0 | 0 | 0 | 0 | 1 | 0 |
| PPCDC | 0 | 0 | 0 | 0 | 0 | 1 | 0 |
| ZC3H12A | 0 | 0 | 0 | 0 | 0 | 1 | 1 |
| TNFRSF14 | 0 | 0 | 0 | 0 | 0 | 1 | 0 |
| CMTM6 | 0 | 0 | 0 | 0 | 0 | 1 | 0 |
| ARSG | 0 | 0 | 0 | 0 | 0 | 1 | 1 |
| NPC2 | 0 | 0 | 0 | 0 | 0 | 1 | 0 |
| DNAJB14 | 0 | 0 | 0 | 0 | 0 | 1 | 0 |
| IRAK1 | 0 | 0 | 0 | 0 | 0 | 1 | 0 |
| RAB2B | 0 | 0 | 0 | 0 | 0 | 1 | 1 |
| CCDC90A | 0 | 0 | 0 | 0 | 0 | 1 | 0 |
| SNRNP200 | 0 | 0 | 0 | 0 | 0 | 1 | 0 |
| TMCC3 | 0 | 0 | 0 | 0 | 0 | 1 | 0 |
| PAR-SN | 0 | 0 | 0 | 0 | 0 | 1 | 0 |
| HSP90B1 | 0 | 0 | 0 | 0 | 0 | 1 | 0 |
| GPCPD1 | 0 | 0 | 0 | 0 | 0 | 1 | 0 |
| EPS15L1 | 0 | 0 | 0 | 0 | 0 | 1 | 0 |
| HIST1H2BC | 0 | 0 | 0 | 0 | 0 | 1 | 0 |
| CHD1 | 0 | 0 | 0 | 0 | 0 | 1 | 0 |
| GBP5 | 0 | 0 | 0 | 0 | 0 | 1 | 1 |
| RRP15 | 0 | 0 | 0 | 0 | 0 | 1 | 0 |
| INSIG1 | 0 | 0 | 0 | 0 | 0 | 1 | 0 |
| KDM4A-AS1 | 0 | 0 | 0 | 0 | 0 | 1 | 0 |
| THAP9-AS1 | 0 | 0 | 0 | 0 | 0 | 1 | 0 |
| TUBA1A | 0 | 0 | 0 | 0 | 0 | 1 | 0 |
| SPRYD7 | 0 | 0 | 0 | 0 | 0 | 1 | 0 |
| ESRG | 0 | 0 | 0 | 0 | 0 | 1 | 0 |
| PGM2L1 | 0 | 0 | 0 | 0 | 0 | 1 | 0 |
| NUCB1 | 0 | 0 | 0 | 0 | 0 | 1 | 1 |
| CEBPB | 0 | 0 | 0 | 0 | 0 | 1 | 0 |
| CCDC30 | 0 | 0 | 0 | 0 | 0 | 1 | 0 |
| FAM41C | 0 | 0 | 0 | 0 | 0 | 1 | 0 |
| DNAJC27-AS1 | 0 | 0 | 0 | 0 | 0 | 1 | 0 |
| ARAF | 0 | 0 | 0 | 0 | 0 | 1 | 0 |
| PLAUR | 0 | 0 | 0 | 0 | 0 | 1 | 1 |
| SMARCA5 | 0 | 0 | 0 | 0 | 0 | 1 | 0 |
| ZNF526 | 0 | 0 | 0 | 0 | 0 | 1 | 1 |
| TMBIM1 | 0 | 0 | 0 | 0 | 0 | 1 | 0 |
| CANT1 | 0 | 0 | 0 | 0 | 0 | 1 | 0 |
| CNIH4 | 0 | 0 | 0 | 0 | 0 | 1 | 0 |
| PDE4B | 0 | 0 | 0 | 0 | 0 | 1 | 0 |
| SUPT20H | 0 | 0 | 0 | 0 | 0 | 1 | 0 |
| BCAS2 | 0 | 0 | 0 | 0 | 0 | 1 | 0 |
| AZIN1 | 0 | 0 | 0 | 0 | 0 | 1 | 0 |
| COPB1 | 0 | 0 | 0 | 0 | 0 | 1 | 0 |
| SERINC3 | 0 | 0 | 0 | 0 | 0 | 1 | 0 |
| SLC3A2 | 0 | 0 | 0 | 0 | 0 | 1 | 0 |
| MX2 | 0 | 0 | 0 | 0 | 0 | 1 | 0 |
| EIF5A2 | 0 | 0 | 0 | 0 | 0 | 1 | 0 |
| SQSTM1 | 0 | 0 | 0 | 0 | 0 | 1 | 0 |
| HIST2H2BF | 0 | 0 | 0 | 0 | 0 | 1 | 0 |
| LOC338758 | 0 | 0 | 0 | 0 | 0 | 1 | 0 |
| EIF4A3 | 0 | 0 | 0 | 0 | 0 | 1 | 0 |
| TPT1 | 0 | 0 | 0 | 0 | 0 | 1 | 0 |
| STRN3 | 0 | 0 | 0 | 0 | 0 | 1 | 0 |
| TM9SF1 | 0 | 0 | 0 | 0 | 0 | 1 | 0 |
| USP16 | 0 | 0 | 0 | 0 | 0 | 1 | 1 |
| AP2M1 | 0 | 0 | 0 | 0 | 0 | 1 | 1 |
| HMGN2 | 0 | 0 | 0 | 0 | 0 | 1 | 0 |
| CSRNP1 | 0 | 0 | 0 | 0 | 0 | 1 | 1 |
| NLN | 0 | 0 | 0 | 0 | 0 | 1 | 0 |
| CASP10 | 0 | 0 | 0 | 0 | 0 | 1 | 0 |
| ALDOA | 0 | 0 | 0 | 0 | 0 | 1 | 0 |
| SSFA2 | 0 | 0 | 0 | 0 | 0 | 1 | 0 |
| CDK5RAP3 | 0 | 0 | 0 | 0 | 0 | 1 | 0 |
| SLC4A1 | 0 | 0 | 0 | 0 | 0 | 1 | 1 |
| TREM1 | 0 | 0 | 0 | 0 | 0 | 1 | 0 |
| HPSE | 0 | 0 | 0 | 0 | 0 | 1 | 1 |
| CD44 | 0 | 0 | 0 | 0 | 0 | 1 | 0 |
| RPL10 | 0 | 0 | 0 | 0 | 0 | 1 | 0 |
| LOC606724 | 0 | 0 | 0 | 0 | 0 | 1 | 0 |
| VPS26A | 0 | 0 | 0 | 0 | 0 | 1 | 0 |
| CHMP2B | 0 | 0 | 0 | 0 | 0 | 1 | 0 |
| MED25 | 0 | 0 | 0 | 0 | 0 | 1 | 1 |
| PPIF | 0 | 0 | 0 | 0 | 0 | 1 | 0 |
| FCHO2 | 0 | 0 | 0 | 0 | 0 | 1 | 0 |
| APLP2 | 0 | 0 | 0 | 0 | 0 | 1 | 0 |
| ICAM1 | 0 | 0 | 0 | 0 | 0 | 1 | 0 |
| PGK1 | 0 | 0 | 0 | 0 | 0 | 1 | 0 |
| ACOT13 | 0 | 0 | 0 | 0 | 0 | 1 | 0 |
| RPL3 | 0 | 0 | 0 | 0 | 0 | 1 | 0 |
| SYTL3 | 0 | 0 | 0 | 0 | 0 | 1 | 0 |
| HSPA5 | 0 | 0 | 0 | 0 | 0 | 1 | 0 |
| ZNF780B | 0 | 0 | 0 | 0 | 0 | 1 | 0 |
| ATP7A | 0 | 0 | 0 | 0 | 0 | 1 | 0 |
| RHBDD2 | 0 | 0 | 0 | 0 | 0 | 1 | 0 |
| SIPA1L1 | 0 | 0 | 0 | 0 | 0 | 1 | 0 |
| PAXBP1-AS1 | 0 | 0 | 0 | 0 | 0 | 1 | 0 |
| IRS2 | 0 | 0 | 0 | 0 | 0 | 1 | 0 |
| CSTA | 0 | 0 | 0 | 0 | 0 | 1 | 0 |
| LOC100505783 | 0 | 0 | 0 | 0 | 0 | 1 | 0 |
| VPS37B | 0 | 0 | 0 | 0 | 0 | 1 | 0 |
| MATR3 | 0 | 0 | 0 | 0 | 0 | 1 | 0 |
| KDM5B | 0 | 0 | 0 | 0 | 0 | 1 | 0 |
| ADPGK | 0 | 0 | 0 | 0 | 0 | 1 | 0 |
| SNX13 | 0 | 0 | 0 | 0 | 0 | 1 | 0 |
| PSMC4 | 0 | 0 | 0 | 0 | 0 | 1 | 0 |
| H6PD | 0 | 0 | 0 | 0 | 0 | 1 | 0 |
| ATP6V0A2 | 0 | 0 | 0 | 0 | 0 | 1 | 0 |
| ZNF548 | 0 | 0 | 0 | 0 | 0 | 1 | 0 |
| SNN | 0 | 0 | 0 | 0 | 0 | 1 | 1 |
| HSH2D | 0 | 0 | 0 | 0 | 0 | 1 | 0 |
| ZFP36L2 | 0 | 0 | 0 | 0 | 0 | 1 | 0 |
| FXR1 | 0 | 0 | 0 | 0 | 0 | 1 | 0 |
| RNASET2 | 0 | 0 | 0 | 0 | 0 | 1 | 0 |
| KLF10 | 0 | 0 | 0 | 0 | 0 | 1 | 0 |
| NUB1 | 0 | 0 | 0 | 0 | 0 | 1 | 0 |
| GTF2H2B | 0 | 0 | 0 | 0 | 0 | 1 | 1 |
| ZSWIM1 | 0 | 0 | 0 | 0 | 0 | 1 | 0 |
| NAIP | 0 | 0 | 0 | 0 | 0 | 1 | 0 |
| MRVI1 | 0 | 0 | 0 | 0 | 0 | 1 | 0 |
| ARIH2 | 0 | 0 | 0 | 0 | 0 | 1 | 0 |
| LDHA | 0 | 0 | 0 | 0 | 0 | 1 | 0 |
| PMAIP1 | 0 | 0 | 0 | 0 | 0 | 1 | 0 |
| TSPO | 0 | 0 | 0 | 0 | 0 | 1 | 0 |
| FFAR2 | 0 | 0 | 0 | 0 | 0 | 1 | 0 |
| POLDIP3 | 0 | 0 | 0 | 0 | 0 | 1 | 0 |
| MASTL | 0 | 0 | 0 | 0 | 0 | 1 | 0 |
| NCOA4 | 0 | 0 | 0 | 0 | 0 | 1 | 0 |
| TRIM66 | 0 | 0 | 0 | 0 | 0 | 1 | 0 |
| CD68 | 0 | 0 | 0 | 0 | 0 | 1 | 0 |
| MICU1 | 0 | 0 | 0 | 0 | 0 | 1 | 1 |
| RELA | 0 | 0 | 0 | 0 | 0 | 1 | 1 |
| SERPINB9 | 0 | 0 | 0 | 0 | 0 | 1 | 0 |
| ARF4 | 0 | 0 | 0 | 0 | 0 | 1 | 0 |
| TMED9 | 0 | 0 | 0 | 0 | 0 | 1 | 0 |
| TMEM45B | 0 | 0 | 0 | 0 | 0 | 1 | 1 |
| AFTPH | 0 | 0 | 0 | 0 | 0 | 1 | 0 |
| MIF | 0 | 0 | 0 | 0 | 0 | 1 | 0 |
| SUPT6H | 0 | 0 | 0 | 0 | 0 | 1 | 0 |
| LPIN2 | 0 | 0 | 0 | 0 | 0 | 1 | 1 |
| MRPL30 | 0 | 0 | 0 | 0 | 0 | 1 | 0 |
| DNAJB11 | 0 | 0 | 0 | 0 | 0 | 1 | 0 |
| PBXIP1 | 0 | 0 | 0 | 0 | 0 | 1 | 0 |
| ZNF527 | 0 | 0 | 0 | 0 | 0 | 1 | 0 |
| RPL14 | 0 | 0 | 0 | 0 | 0 | 1 | 0 |
| TNFRSF1B | 0 | 0 | 0 | 0 | 0 | 1 | 0 |
| RPL11 | 0 | 0 | 0 | 0 | 0 | 1 | 0 |
| IVNS1ABP | 0 | 0 | 0 | 0 | 0 | 1 | 0 |
| KDELR2 | 0 | 0 | 0 | 0 | 0 | 1 | 0 |
| KLHL2 | 0 | 0 | 0 | 0 | 0 | 1 | 0 |
| ZNF234 | 0 | 0 | 0 | 0 | 0 | 1 | 0 |
| RLIM | 0 | 0 | 0 | 0 | 0 | 1 | 0 |
| CXCL16 | 0 | 0 | 0 | 0 | 0 | 1 | 1 |
| OCLN | 0 | 0 | 0 | 0 | 0 | 1 | 0 |
| METTL6 | 0 | 0 | 0 | 0 | 0 | 1 | 0 |
| SCN3B | 0 | 0 | 0 | 0 | 0 | 1 | 0 |
| POLR2J3 | 0 | 0 | 0 | 0 | 0 | 1 | 0 |
| SH3BP5 | 0 | 0 | 0 | 0 | 0 | 1 | 0 |
| ARL17A | 0 | 0 | 0 | 0 | 0 | 1 | 0 |
| ARFIP1 | 0 | 0 | 0 | 0 | 0 | 1 | 0 |
| C17orf103 | 0 | 0 | 0 | 0 | 0 | 1 | 0 |
| KLHL24 | 0 | 0 | 0 | 0 | 0 | 1 | 0 |
| ODF3B | 0 | 0 | 0 | 0 | 0 | 1 | 0 |
| CPSF7 | 0 | 0 | 0 | 0 | 0 | 1 | 0 |
| RPL6 | 0 | 0 | 0 | 0 | 0 | 1 | 0 |
| GAPDH | 0 | 0 | 0 | 0 | 0 | 1 | 0 |
| MORF4L2 | 0 | 0 | 0 | 0 | 0 | 1 | 0 |
| GAA | 0 | 0 | 0 | 0 | 0 | 1 | 0 |
| NFKBID | 0 | 0 | 0 | 0 | 0 | 1 | 0 |
| PTBP1 | 0 | 0 | 0 | 0 | 0 | 1 | 0 |
| PRKACA | 0 | 0 | 0 | 0 | 0 | 1 | 0 |
| TIMP2 | 0 | 0 | 0 | 0 | 0 | 1 | 0 |
| USMG5 | 0 | 0 | 0 | 0 | 0 | 1 | 0 |
| GNB4 | 0 | 0 | 0 | 0 | 0 | 1 | 0 |
| HIAT1 | 0 | 0 | 0 | 0 | 0 | 1 | 0 |
| PER2 | 0 | 0 | 0 | 0 | 0 | 1 | 0 |
| LOC100129250 | 0 | 0 | 0 | 0 | 0 | 1 | 0 |
| PHOSPHO1 | 0 | 0 | 0 | 0 | 0 | 1 | 1 |
| KDM3A | 0 | 0 | 0 | 0 | 0 | 1 | 0 |
| RPS16 | 0 | 0 | 0 | 0 | 0 | 1 | 0 |
| MAB21L3 | 0 | 0 | 0 | 0 | 0 | 1 | 0 |
| MGC2752 | 0 | 0 | 0 | 0 | 0 | 1 | 0 |
| PRKDC | 0 | 0 | 0 | 0 | 0 | 1 | 0 |
| SYAP1 | 0 | 0 | 0 | 0 | 0 | 1 | 0 |
| RLF | 0 | 0 | 0 | 0 | 0 | 1 | 0 |
| AP2A1 | 0 | 0 | 0 | 0 | 0 | 1 | 0 |
| CANX | 0 | 0 | 0 | 0 | 0 | 1 | 0 |
| SNRPB | 0 | 0 | 0 | 0 | 0 | 1 | 0 |
| SLC26A8 | 0 | 0 | 0 | 0 | 0 | 1 | 0 |
| ETF1 | 0 | 0 | 0 | 0 | 0 | 1 | 0 |
| HIVEP1 | 0 | 0 | 0 | 0 | 0 | 1 | 0 |
| RANBP2 | 0 | 0 | 0 | 0 | 0 | 1 | 0 |
| NDUFB3 | 0 | 0 | 0 | 0 | 0 | 1 | 1 |
| PNPLA8 | 0 | 0 | 0 | 0 | 0 | 1 | 0 |
| CDK5RAP2 | 0 | 0 | 0 | 0 | 0 | 1 | 0 |
| ENO1 | 0 | 0 | 0 | 0 | 0 | 1 | 0 |
| RAB22A | 0 | 0 | 0 | 0 | 0 | 1 | 0 |
| NBN | 0 | 0 | 0 | 0 | 0 | 1 | 0 |
| CPPED1 | 0 | 0 | 0 | 0 | 0 | 1 | 0 |
| EOGT | 0 | 0 | 0 | 0 | 0 | 1 | 0 |
| DHRSX | 0 | 0 | 0 | 0 | 0 | 1 | 1 |
| UBE2L6 | 0 | 0 | 0 | 0 | 0 | 1 | 1 |
| CCDC71L | 0 | 0 | 0 | 0 | 0 | 1 | 0 |
| NAPA | 0 | 0 | 0 | 0 | 0 | 1 | 1 |
| HMGN1 | 0 | 0 | 0 | 0 | 0 | 1 | 0 |
| DMXL2 | 0 | 0 | 0 | 0 | 0 | 1 | 0 |
| MCOLN1 | 0 | 0 | 0 | 0 | 0 | 1 | 1 |
| RPS29 | 0 | 0 | 0 | 0 | 0 | 1 | 0 |
| PRPF4 | 0 | 0 | 0 | 0 | 0 | 1 | 1 |
| IFIT3 | 0 | 0 | 0 | 0 | 0 | 1 | 1 |
| ARHGDIA | 0 | 0 | 0 | 0 | 0 | 1 | 0 |
| ZNF440 | 0 | 0 | 0 | 0 | 0 | 1 | 0 |
| ZNF554 | 0 | 0 | 0 | 0 | 0 | 1 | 0 |
| MAPKAPK3 | 0 | 0 | 0 | 0 | 0 | 1 | 0 |
| EMD | 0 | 0 | 0 | 0 | 0 | 1 | 0 |
| SERTAD1 | 0 | 0 | 0 | 0 | 0 | 1 | 0 |
| KCTD20 | 0 | 0 | 0 | 0 | 0 | 1 | 0 |
| MSMO1 | 0 | 0 | 0 | 0 | 0 | 1 | 0 |
| PECAM1 | 0 | 0 | 0 | 0 | 0 | 1 | 0 |
| MGAT4A | 0 | 0 | 0 | 0 | 0 | 1 | 0 |
| SLMO2 | 0 | 0 | 0 | 0 | 0 | 1 | 0 |
| LOC284454 | 0 | 0 | 0 | 0 | 0 | 1 | 0 |
| AP2B1 | 0 | 0 | 0 | 0 | 0 | 1 | 1 |
| FAM210B | 0 | 0 | 0 | 0 | 0 | 1 | 1 |
| ATXN2L | 0 | 0 | 0 | 0 | 0 | 1 | 0 |
| GGA1 | 0 | 0 | 0 | 0 | 0 | 1 | 0 |
| ZNF37A | 0 | 0 | 0 | 0 | 0 | 1 | 0 |
| EMB | 0 | 0 | 0 | 0 | 0 | 1 | 0 |
| LOC100506710 | 0 | 0 | 0 | 0 | 0 | 1 | 0 |
| DNTTIP2 | 0 | 0 | 0 | 0 | 0 | 1 | 1 |
| GZF1 | 0 | 0 | 0 | 0 | 0 | 1 | 0 |
| OXSR1 | 0 | 0 | 0 | 0 | 0 | 1 | 0 |
| TPI1 | 0 | 0 | 0 | 0 | 0 | 1 | 0 |
| NKIRAS2 | 0 | 0 | 0 | 0 | 0 | 1 | 1 |
| DDX21 | 0 | 0 | 0 | 0 | 0 | 1 | 0 |
| RBM41 | 0 | 0 | 0 | 0 | 0 | 1 | 0 |
| GTPBP1 | 0 | 0 | 0 | 0 | 0 | 1 | 0 |
| CPD | 0 | 0 | 0 | 0 | 0 | 1 | 0 |
| IRAK3 | 0 | 0 | 0 | 0 | 0 | 1 | 0 |
| GPX4 | 0 | 0 | 0 | 0 | 0 | 1 | 0 |
| SIGLEC10 | 0 | 0 | 0 | 0 | 0 | 1 | 1 |
| ZEB1 | 0 | 0 | 0 | 0 | 0 | 1 | 0 |
| HEXB | 0 | 0 | 0 | 0 | 0 | 1 | 0 |
| USP14 | 0 | 0 | 0 | 0 | 0 | 1 | 0 |
| GPR65 | 0 | 0 | 0 | 0 | 0 | 1 | 0 |
| ZNF516 | 0 | 0 | 0 | 0 | 0 | 1 | 0 |
| EML4 | 0 | 0 | 0 | 0 | 0 | 1 | 0 |
| GBP6 | 0 | 0 | 0 | 0 | 0 | 1 | 0 |
| HIF1A | 0 | 0 | 0 | 0 | 0 | 1 | 0 |
| AGPAT6 | 0 | 0 | 0 | 0 | 0 | 1 | 0 |
| FAM174A | 0 | 0 | 0 | 0 | 0 | 1 | 0 |
| RNF169 | 0 | 0 | 0 | 0 | 0 | 1 | 0 |
| SESN2 | 0 | 0 | 0 | 0 | 0 | 1 | 0 |
| S100A4 | 0 | 0 | 0 | 0 | 0 | 1 | 0 |
| SIN3A | 0 | 0 | 0 | 0 | 0 | 1 | 0 |
| RABGEF1 | 0 | 0 | 0 | 0 | 0 | 1 | 0 |
| RGS2 | 0 | 0 | 0 | 0 | 0 | 1 | 0 |
| QSOX1 | 0 | 0 | 0 | 0 | 0 | 1 | 0 |
| ATG2A | 0 | 0 | 0 | 0 | 0 | 1 | 0 |
| HSP90AB1 | 0 | 0 | 0 | 0 | 0 | 1 | 0 |
| DNAJC14 | 0 | 0 | 0 | 0 | 0 | 1 | 0 |
| RHEB | 0 | 0 | 0 | 0 | 0 | 1 | 0 |
| KCNE3 | 0 | 0 | 0 | 0 | 0 | 1 | 1 |
| RPS8 | 0 | 0 | 0 | 0 | 0 | 1 | 0 |
| APH1B | 0 | 0 | 0 | 0 | 0 | 1 | 0 |
| STOM | 0 | 0 | 0 | 0 | 0 | 1 | 0 |
| MOAP1 | 0 | 0 | 0 | 0 | 0 | 1 | 0 |
| CD97 | 0 | 0 | 0 | 0 | 0 | 1 | 0 |
| MOCS3 | 0 | 0 | 0 | 0 | 0 | 1 | 0 |
| SEC16A | 0 | 0 | 0 | 0 | 0 | 1 | 0 |
| LINC00476 | 0 | 0 | 0 | 0 | 0 | 1 | 0 |
| PLK3 | 0 | 0 | 0 | 0 | 0 | 1 | 0 |
| SLC20A1 | 0 | 0 | 0 | 0 | 0 | 1 | 0 |
| SULF2 | 0 | 0 | 0 | 0 | 0 | 1 | 1 |
| NXPE3 | 0 | 0 | 0 | 0 | 0 | 1 | 0 |
| CD83 | 0 | 0 | 0 | 0 | 0 | 1 | 0 |
| TRAF3IP3 | 0 | 0 | 0 | 0 | 0 | 1 | 1 |
| CLEC2D | 0 | 0 | 0 | 0 | 0 | 1 | 0 |
| RASA4 | 0 | 0 | 0 | 0 | 0 | 1 | 0 |
| POLB | 0 | 0 | 0 | 0 | 0 | 1 | 0 |
| MAP3K8 | 0 | 0 | 0 | 0 | 0 | 1 | 0 |
| PURB | 0 | 0 | 0 | 0 | 0 | 1 | 0 |
| GADD45B | 0 | 0 | 0 | 0 | 0 | 1 | 0 |
| PLEKHB2 | 0 | 0 | 0 | 0 | 0 | 1 | 0 |
| MLH3 | 0 | 0 | 0 | 0 | 0 | 0 | 1 |
| TMEM176A | 0 | 0 | 0 | 0 | 0 | 0 | 1 |
| SCGB3A1 | 0 | 0 | 0 | 0 | 0 | 0 | 1 |
| TMEM176B | 0 | 0 | 0 | 0 | 0 | 0 | 1 |
| HSPC102 | 0 | 0 | 0 | 0 | 0 | 0 | 1 |
| CHP1 | 0 | 0 | 0 | 0 | 0 | 0 | 1 |
| NATD1 | 0 | 0 | 0 | 0 | 0 | 0 | 1 |
| APOM | 0 | 0 | 0 | 0 | 0 | 0 | 1 |
| LOC101928343 | 0 | 0 | 0 | 0 | 0 | 0 | 1 |
| PRR11 | 0 | 0 | 0 | 0 | 0 | 0 | 1 |
| CUTALP | 0 | 0 | 0 | 0 | 0 | 0 | 1 |
| ACSM2A | 0 | 0 | 0 | 0 | 0 | 0 | 1 |
| SMAGP | 0 | 0 | 0 | 0 | 0 | 0 | 1 |
| CDK5 | 0 | 0 | 0 | 0 | 0 | 0 | 1 |
| EEF1AKMT3 | 0 | 0 | 0 | 0 | 0 | 0 | 1 |
| CD99P1 | 0 | 0 | 0 | 0 | 0 | 0 | 1 |
| HNRNPH3 | 0 | 0 | 0 | 0 | 0 | 0 | 1 |
| INSIG1-DT | 0 | 0 | 0 | 0 | 0 | 0 | 1 |
| SIGLEC9 | 0 | 0 | 0 | 0 | 0 | 0 | 1 |
| SLC24A3 | 0 | 0 | 0 | 0 | 0 | 0 | 1 |
| SERPINB9P1 | 0 | 0 | 0 | 0 | 0 | 0 | 1 |
| CCEPR | 0 | 0 | 0 | 0 | 0 | 0 | 1 |
| LOC100996756 | 0 | 0 | 0 | 0 | 0 | 0 | 1 |
| SLC22A3 | 0 | 0 | 0 | 0 | 0 | 0 | 1 |
| LINC02802 | 0 | 0 | 0 | 0 | 0 | 0 | 1 |
| SIRT6 | 0 | 0 | 0 | 0 | 0 | 0 | 1 |
| DEPDC5 | 0 | 0 | 0 | 0 | 0 | 0 | 1 |
| TRIB3 | 0 | 0 | 0 | 0 | 0 | 0 | 1 |
| SLC10A3 | 0 | 0 | 0 | 0 | 0 | 0 | 1 |
| SENCR | 0 | 0 | 0 | 0 | 0 | 0 | 1 |
| ELAPOR1 | 0 | 0 | 0 | 0 | 0 | 0 | 1 |
| RITA1 | 0 | 0 | 0 | 0 | 0 | 0 | 1 |
| MAX | 0 | 0 | 0 | 0 | 0 | 0 | 1 |
| CASTOR3 | 0 | 0 | 0 | 0 | 0 | 0 | 1 |
| KHNYN | 0 | 0 | 0 | 0 | 0 | 0 | 1 |
| ANKRD36B | 0 | 0 | 0 | 0 | 0 | 0 | 1 |
| PKIG | 0 | 0 | 0 | 0 | 0 | 0 | 1 |
| LOC105371215 | 0 | 0 | 0 | 0 | 0 | 0 | 1 |
| CMAS | 0 | 0 | 0 | 0 | 0 | 0 | 1 |
| ZNF595 | 0 | 0 | 0 | 0 | 0 | 0 | 1 |
| ARF3 | 0 | 0 | 0 | 0 | 0 | 0 | 1 |
| NCR3 | 0 | 0 | 0 | 0 | 0 | 0 | 1 |
| AGPAT1 | 0 | 0 | 0 | 0 | 0 | 0 | 1 |
| DHFR2 | 0 | 0 | 0 | 0 | 0 | 0 | 1 |
| LRRC42 | 0 | 0 | 0 | 0 | 0 | 0 | 1 |
| ACTR1B | 0 | 0 | 0 | 0 | 0 | 0 | 1 |
| BEND2 | 0 | 0 | 0 | 0 | 0 | 0 | 1 |
| ELOA-AS1 | 0 | 0 | 0 | 0 | 0 | 0 | 1 |
| MIX23 | 0 | 0 | 0 | 0 | 0 | 0 | 1 |
| ELP5 | 0 | 0 | 0 | 0 | 0 | 0 | 1 |
| DUBR | 0 | 0 | 0 | 0 | 0 | 0 | 1 |
| UGT2B28 | 0 | 0 | 0 | 0 | 0 | 0 | 1 |
| LOC100289333 | 0 | 0 | 0 | 0 | 0 | 0 | 1 |
| ENTPD1-AS1 | 0 | 0 | 0 | 0 | 0 | 0 | 1 |
| RAMP2-AS1 | 0 | 0 | 0 | 0 | 0 | 0 | 1 |
| DYNLL2 | 0 | 0 | 0 | 0 | 0 | 0 | 1 |
| MRPL49 | 0 | 0 | 0 | 0 | 0 | 0 | 1 |
| ADAM33 | 0 | 0 | 0 | 0 | 0 | 0 | 1 |
| RAB37 | 0 | 0 | 0 | 0 | 0 | 0 | 1 |
| LOC105375547 | 0 | 0 | 0 | 0 | 0 | 0 | 1 |
| ACADVL | 0 | 0 | 0 | 0 | 0 | 0 | 1 |
| LOC105369228 | 0 | 0 | 0 | 0 | 0 | 0 | 1 |
| BRD3OS | 0 | 0 | 0 | 0 | 0 | 0 | 1 |
| PRPF38B | 0 | 0 | 0 | 0 | 0 | 0 | 1 |
| RIC8A | 0 | 0 | 0 | 0 | 0 | 0 | 1 |
| ECRG4 | 0 | 0 | 0 | 0 | 0 | 0 | 1 |
| DDX11L2 | 0 | 0 | 0 | 0 | 0 | 0 | 1 |
| H2BC21 | 0 | 0 | 0 | 0 | 0 | 0 | 1 |
| LOC114224 | 0 | 0 | 0 | 0 | 0 | 0 | 1 |
| TMEM185A | 0 | 0 | 0 | 0 | 0 | 0 | 1 |
| ARHGEF1 | 0 | 0 | 0 | 0 | 0 | 0 | 1 |
| TASL | 0 | 0 | 0 | 0 | 0 | 0 | 1 |
| POM121L8P | 0 | 0 | 0 | 0 | 0 | 0 | 1 |
| GNG5 | 0 | 0 | 0 | 0 | 0 | 0 | 1 |
| ZNF747 | 0 | 0 | 0 | 0 | 0 | 0 | 1 |
| ACTR1A | 0 | 0 | 0 | 0 | 0 | 0 | 1 |
| TBC1D25 | 0 | 0 | 0 | 0 | 0 | 0 | 1 |
| ATG13 | 0 | 0 | 0 | 0 | 0 | 0 | 1 |
| NIPSNAP1 | 0 | 0 | 0 | 0 | 0 | 0 | 1 |
| FKBP1A | 0 | 0 | 0 | 0 | 0 | 0 | 1 |
| BRF2 | 0 | 0 | 0 | 0 | 0 | 0 | 1 |
| TP53I3 | 0 | 0 | 0 | 0 | 0 | 0 | 1 |
| PVALB | 0 | 0 | 0 | 0 | 0 | 0 | 1 |
| SH3BGRL3 | 0 | 0 | 0 | 0 | 0 | 0 | 1 |
| CREB3L4 | 0 | 0 | 0 | 0 | 0 | 0 | 1 |
| NOTCH2NLA | 0 | 0 | 0 | 0 | 0 | 0 | 1 |
| BRAF | 0 | 0 | 0 | 0 | 0 | 0 | 1 |
| DHRS1 | 0 | 0 | 0 | 0 | 0 | 0 | 1 |
| ERMAP | 0 | 0 | 0 | 0 | 0 | 0 | 1 |
| LOC105378577 | 0 | 0 | 0 | 0 | 0 | 0 | 1 |
| ELOVL7 | 0 | 0 | 0 | 0 | 0 | 0 | 1 |
| HIF1AN | 0 | 0 | 0 | 0 | 0 | 0 | 1 |
| H2BC8 | 0 | 0 | 0 | 0 | 0 | 0 | 1 |
| TNFAIP8L1 | 0 | 0 | 0 | 0 | 0 | 0 | 1 |
| SMIM24 | 0 | 0 | 0 | 0 | 0 | 0 | 1 |
| KIR2DL2 | 0 | 0 | 0 | 0 | 0 | 0 | 1 |
| ECSIT | 0 | 0 | 0 | 0 | 0 | 0 | 1 |
| HOMER1 | 0 | 0 | 0 | 0 | 0 | 0 | 1 |
| CAVIN3 | 0 | 0 | 0 | 0 | 0 | 0 | 1 |
| TTC7B | 0 | 0 | 0 | 0 | 0 | 0 | 1 |
| CNN2 | 0 | 0 | 0 | 0 | 0 | 0 | 1 |
| SWI5 | 0 | 0 | 0 | 0 | 0 | 0 | 1 |
| COL9A3 | 0 | 0 | 0 | 0 | 0 | 0 | 1 |
| ACD | 0 | 0 | 0 | 0 | 0 | 0 | 1 |
| TMEM109 | 0 | 0 | 0 | 0 | 0 | 0 | 1 |
| C8orf82 | 0 | 0 | 0 | 0 | 0 | 0 | 1 |
| MRPL51 | 0 | 0 | 0 | 0 | 0 | 0 | 1 |
| MPV17 | 0 | 0 | 0 | 0 | 0 | 0 | 1 |
| VPS72 | 0 | 0 | 0 | 0 | 0 | 0 | 1 |
| LOC105379362 | 0 | 0 | 0 | 0 | 0 | 0 | 1 |
| MIF4GD | 0 | 0 | 0 | 0 | 0 | 0 | 1 |
| TMEM212 | 0 | 0 | 0 | 0 | 0 | 0 | 1 |
| SNRPN | 0 | 0 | 0 | 0 | 0 | 0 | 1 |
| PPP1R18 | 0 | 0 | 0 | 0 | 0 | 0 | 1 |
| DUSP14 | 0 | 0 | 0 | 0 | 0 | 0 | 1 |
| ZNF721 | 0 | 0 | 0 | 0 | 0 | 0 | 1 |
| SRXN1 | 0 | 0 | 0 | 0 | 0 | 0 | 1 |
| DET1 | 0 | 0 | 0 | 0 | 0 | 0 | 1 |
| CTDSP1 | 0 | 0 | 0 | 0 | 0 | 0 | 1 |
| TKTL1 | 0 | 0 | 0 | 0 | 0 | 0 | 1 |
| SNAI3 | 0 | 0 | 0 | 0 | 0 | 0 | 1 |
| HADHA | 0 | 0 | 0 | 0 | 0 | 0 | 1 |
| RSRP1 | 0 | 0 | 0 | 0 | 0 | 0 | 1 |
| LOC100507642 | 0 | 0 | 0 | 0 | 0 | 0 | 1 |
| MACF1 | 0 | 0 | 0 | 0 | 0 | 0 | 1 |
| GUSBP14 | 0 | 0 | 0 | 0 | 0 | 0 | 1 |
| PDLIM1 | 0 | 0 | 0 | 0 | 0 | 0 | 1 |
| TNFSF12 | 0 | 0 | 0 | 0 | 0 | 0 | 1 |
| SSBP3-AS1 | 0 | 0 | 0 | 0 | 0 | 0 | 1 |
| PRSS33 | 0 | 0 | 0 | 0 | 0 | 0 | 1 |
| TAL1 | 0 | 0 | 0 | 0 | 0 | 0 | 1 |
| GCHFR | 0 | 0 | 0 | 0 | 0 | 0 | 1 |
| GPS2 | 0 | 0 | 0 | 0 | 0 | 0 | 1 |
| POLR2J | 0 | 0 | 0 | 0 | 0 | 0 | 1 |
| CPTP | 0 | 0 | 0 | 0 | 0 | 0 | 1 |
| CAPNS1 | 0 | 0 | 0 | 0 | 0 | 0 | 1 |
| DEFB108B | 0 | 0 | 0 | 0 | 0 | 0 | 1 |
| ZSWIM3 | 0 | 0 | 0 | 0 | 0 | 0 | 1 |
| GOLGA8IP | 0 | 0 | 0 | 0 | 0 | 0 | 1 |
| CHID1 | 0 | 0 | 0 | 0 | 0 | 0 | 1 |
| LIPA | 0 | 0 | 0 | 0 | 0 | 0 | 1 |
| SPARC | 0 | 0 | 0 | 0 | 0 | 0 | 1 |
| RANGRF | 0 | 0 | 0 | 0 | 0 | 0 | 1 |
| LOC101927770 | 0 | 0 | 0 | 0 | 0 | 0 | 1 |
| LINC01809 | 0 | 0 | 0 | 0 | 0 | 0 | 1 |
| KPNA1 | 0 | 0 | 0 | 0 | 0 | 0 | 1 |
| SLC29A3 | 0 | 0 | 0 | 0 | 0 | 0 | 1 |
| POLDIP2 | 0 | 0 | 0 | 0 | 0 | 0 | 1 |
| CHI3L2 | 0 | 0 | 0 | 0 | 0 | 0 | 1 |
| WIF1 | 0 | 0 | 0 | 0 | 0 | 0 | 1 |
| LOC729732 | 0 | 0 | 0 | 0 | 0 | 0 | 1 |
| MMP24OS | 0 | 0 | 0 | 0 | 0 | 0 | 1 |
| DESI1 | 0 | 0 | 0 | 0 | 0 | 0 | 1 |
| GPX7 | 0 | 0 | 0 | 0 | 0 | 0 | 1 |
| ASB6 | 0 | 0 | 0 | 0 | 0 | 0 | 1 |
| FBXW12 | 0 | 0 | 0 | 0 | 0 | 0 | 1 |
| SHISA4 | 0 | 0 | 0 | 0 | 0 | 0 | 1 |
| COX2 | 0 | 0 | 0 | 0 | 0 | 0 | 1 |
| H2AC11 | 0 | 0 | 0 | 0 | 0 | 0 | 1 |
| PI4KB | 0 | 0 | 0 | 0 | 0 | 0 | 1 |
| RETREG2 | 0 | 0 | 0 | 0 | 0 | 0 | 1 |
| SLC9B1 | 0 | 0 | 0 | 0 | 0 | 0 | 1 |
| OR52K3P | 0 | 0 | 0 | 0 | 0 | 0 | 1 |
| ND4 | 0 | 0 | 0 | 0 | 0 | 0 | 1 |
| MAP1S | 0 | 0 | 0 | 0 | 0 | 0 | 1 |
| RILP | 0 | 0 | 0 | 0 | 0 | 0 | 1 |
| YY1AP1 | 0 | 0 | 0 | 0 | 0 | 0 | 1 |
| PCLAF | 0 | 0 | 0 | 0 | 0 | 0 | 1 |
| ALDOC | 0 | 0 | 0 | 0 | 0 | 0 | 1 |
| MEN1 | 0 | 0 | 0 | 0 | 0 | 0 | 1 |
| CIDEB | 0 | 0 | 0 | 0 | 0 | 0 | 1 |
| RNF185 | 0 | 0 | 0 | 0 | 0 | 0 | 1 |
| CMTM5 | 0 | 0 | 0 | 0 | 0 | 0 | 1 |
| LINC00328 | 0 | 0 | 0 | 0 | 0 | 0 | 1 |
| TSHZ3 | 0 | 0 | 0 | 0 | 0 | 0 | 1 |
| LOC105376486 | 0 | 0 | 0 | 0 | 0 | 0 | 1 |
| TRIM26 | 0 | 0 | 0 | 0 | 0 | 0 | 1 |
| COPS6 | 0 | 0 | 0 | 0 | 0 | 0 | 1 |
| ZNF394 | 0 | 0 | 0 | 0 | 0 | 0 | 1 |
| RBM22 | 0 | 0 | 0 | 0 | 0 | 0 | 1 |
| CNPPD1 | 0 | 0 | 0 | 0 | 0 | 0 | 1 |
| RAB43 | 0 | 0 | 0 | 0 | 0 | 0 | 1 |
| ORAI3 | 0 | 0 | 0 | 0 | 0 | 0 | 1 |
| MPST | 0 | 0 | 0 | 0 | 0 | 0 | 1 |
| ELF4 | 0 | 0 | 0 | 0 | 0 | 0 | 1 |
| CCDC159 | 0 | 0 | 0 | 0 | 0 | 0 | 1 |
| SIRPB1 | 0 | 0 | 0 | 0 | 0 | 0 | 1 |
| EFNA4 | 0 | 0 | 0 | 0 | 0 | 0 | 1 |
| XNDC1N | 0 | 0 | 0 | 0 | 0 | 0 | 1 |
| CDC42EP2 | 0 | 0 | 0 | 0 | 0 | 0 | 1 |
| MXRA7 | 0 | 0 | 0 | 0 | 0 | 0 | 1 |
| RCOR3 | 0 | 0 | 0 | 0 | 0 | 0 | 1 |
| GMDS-DT | 0 | 0 | 0 | 0 | 0 | 0 | 1 |
| LYSMD2 | 0 | 0 | 0 | 0 | 0 | 0 | 1 |
| UTP4 | 0 | 0 | 0 | 0 | 0 | 0 | 1 |
| CYB561D2 | 0 | 0 | 0 | 0 | 0 | 0 | 1 |
| RTL8C | 0 | 0 | 0 | 0 | 0 | 0 | 1 |
| LOC100505874 | 0 | 0 | 0 | 0 | 0 | 0 | 1 |
| H3C10 | 0 | 0 | 0 | 0 | 0 | 0 | 1 |
| KLHDC3 | 0 | 0 | 0 | 0 | 0 | 0 | 1 |
| TBC1D20 | 0 | 0 | 0 | 0 | 0 | 0 | 1 |
| HEXIM2 | 0 | 0 | 0 | 0 | 0 | 0 | 1 |
| LOC105377458 | 0 | 0 | 0 | 0 | 0 | 0 | 1 |
| ESF1 | 0 | 0 | 0 | 0 | 0 | 0 | 1 |
| LOC100190986 | 0 | 0 | 0 | 0 | 0 | 0 | 1 |
| PFAS | 0 | 0 | 0 | 0 | 0 | 0 | 1 |
| SHISA5 | 0 | 0 | 0 | 0 | 0 | 0 | 1 |
| RNASEH2A | 0 | 0 | 0 | 0 | 0 | 0 | 1 |
| H2AC8 | 0 | 0 | 0 | 0 | 0 | 0 | 1 |
| LINC02754 | 0 | 0 | 0 | 0 | 0 | 0 | 1 |
| ND2 | 0 | 0 | 0 | 0 | 0 | 0 | 1 |
| VPS26B | 0 | 0 | 0 | 0 | 0 | 0 | 1 |
| CLK4 | 0 | 0 | 0 | 0 | 0 | 0 | 1 |
| ANKRD20A12P | 0 | 0 | 0 | 0 | 0 | 0 | 1 |
| TOX2 | 0 | 0 | 0 | 0 | 0 | 0 | 1 |
| TTI2 | 0 | 0 | 0 | 0 | 0 | 0 | 1 |
| LRRC75A | 0 | 0 | 0 | 0 | 0 | 0 | 1 |
| USP30 | 0 | 0 | 0 | 0 | 0 | 0 | 1 |
| ZNF831 | 0 | 0 | 0 | 0 | 0 | 0 | 1 |
| MIR3682 | 0 | 0 | 0 | 0 | 0 | 0 | 1 |
| RMDN3 | 0 | 0 | 0 | 0 | 0 | 0 | 1 |
| TAGLN2 | 0 | 0 | 0 | 0 | 0 | 0 | 1 |
| PRDX6 | 0 | 0 | 0 | 0 | 0 | 0 | 1 |
| ACTR3BP2 | 0 | 0 | 0 | 0 | 0 | 0 | 1 |
| NDUFB1 | 0 | 0 | 0 | 0 | 0 | 0 | 1 |
| SNIP1 | 0 | 0 | 0 | 0 | 0 | 0 | 1 |
| PPP5D1P | 0 | 0 | 0 | 0 | 0 | 0 | 1 |
| DENND4A | 0 | 0 | 0 | 0 | 0 | 0 | 1 |
| TRIM35 | 0 | 0 | 0 | 0 | 0 | 0 | 1 |
| C9orf64 | 0 | 0 | 0 | 0 | 0 | 0 | 1 |
| RASEF | 0 | 0 | 0 | 0 | 0 | 0 | 1 |
| CCDC18-AS1 | 0 | 0 | 0 | 0 | 0 | 0 | 1 |
| TUBA3D | 0 | 0 | 0 | 0 | 0 | 0 | 1 |
| ACKR4 | 0 | 0 | 0 | 0 | 0 | 0 | 1 |
| LGALS3BP | 0 | 0 | 0 | 0 | 0 | 0 | 1 |
| TSPAN33 | 0 | 0 | 0 | 0 | 0 | 0 | 1 |
| TIPARP | 0 | 0 | 0 | 0 | 0 | 0 | 1 |
| DDX41 | 0 | 0 | 0 | 0 | 0 | 0 | 1 |
| HLA-DRB4 | 0 | 0 | 0 | 0 | 0 | 0 | 1 |
| S1PR1-DT | 0 | 0 | 0 | 0 | 0 | 0 | 1 |
| NOP58 | 0 | 0 | 0 | 0 | 0 | 0 | 1 |
| ALG8 | 0 | 0 | 0 | 0 | 0 | 0 | 1 |
| LOC389834 | 0 | 0 | 0 | 0 | 0 | 0 | 1 |
| STARD10 | 0 | 0 | 0 | 0 | 0 | 0 | 1 |
| TIMM8A | 0 | 0 | 0 | 0 | 0 | 0 | 1 |
| TUBB4B | 0 | 0 | 0 | 0 | 0 | 0 | 1 |
| TRA2A | 0 | 0 | 0 | 0 | 0 | 0 | 1 |
| CDK4 | 0 | 0 | 0 | 0 | 0 | 0 | 1 |
| SLC25A11 | 0 | 0 | 0 | 0 | 0 | 0 | 1 |
| F8 | 0 | 0 | 0 | 0 | 0 | 0 | 1 |
| TUSC2 | 0 | 0 | 0 | 0 | 0 | 0 | 1 |
| PELI3 | 0 | 0 | 0 | 0 | 0 | 0 | 1 |
| MCUR1 | 0 | 0 | 0 | 0 | 0 | 0 | 1 |
| SUV39H1 | 0 | 0 | 0 | 0 | 0 | 0 | 1 |
| MIPEPP3 | 0 | 0 | 0 | 0 | 0 | 0 | 1 |
| LOC101927018 | 0 | 0 | 0 | 0 | 0 | 0 | 1 |
| PIP5K1C | 0 | 0 | 0 | 0 | 0 | 0 | 1 |
| COX3 | 0 | 0 | 0 | 0 | 0 | 0 | 1 |
| MAP3K1 | 0 | 0 | 0 | 0 | 0 | 0 | 1 |
| RUNDC3A | 0 | 0 | 0 | 0 | 0 | 0 | 1 |
| IP6K2 | 0 | 0 | 0 | 0 | 0 | 0 | 1 |
| SIGLEC5 | 0 | 0 | 0 | 0 | 0 | 0 | 1 |
| TMEM164 | 0 | 0 | 0 | 0 | 0 | 0 | 1 |
| CARM1 | 0 | 0 | 0 | 0 | 0 | 0 | 1 |
| FRG1BP | 0 | 0 | 0 | 0 | 0 | 0 | 1 |
| FHL3 | 0 | 0 | 0 | 0 | 0 | 0 | 1 |
| MALAT1 | 0 | 0 | 0 | 0 | 0 | 0 | 1 |
| MED8 | 0 | 0 | 0 | 0 | 0 | 0 | 1 |
| PRAF2 | 0 | 0 | 0 | 0 | 0 | 0 | 1 |
| GBGT1 | 0 | 0 | 0 | 0 | 0 | 0 | 1 |
| HEY1 | 0 | 0 | 0 | 0 | 0 | 0 | 1 |
| FKSG49 | 0 | 0 | 0 | 0 | 0 | 0 | 1 |
| GFI1B | 0 | 0 | 0 | 0 | 0 | 0 | 1 |
| MLLT11 | 0 | 0 | 0 | 0 | 0 | 0 | 1 |
| ZSCAN22 | 0 | 0 | 0 | 0 | 0 | 0 | 1 |
| CIZ1 | 0 | 0 | 0 | 0 | 0 | 0 | 1 |
| XKR8 | 0 | 0 | 0 | 0 | 0 | 0 | 1 |
| CHAMP1 | 0 | 0 | 0 | 0 | 0 | 0 | 1 |
| TMEM141 | 0 | 0 | 0 | 0 | 0 | 0 | 1 |
| MGLL | 0 | 0 | 0 | 0 | 0 | 0 | 1 |
| GNAZ | 0 | 0 | 0 | 0 | 0 | 0 | 1 |
| GOLGA6L2 | 0 | 0 | 0 | 0 | 0 | 0 | 1 |
| NEK2-DT | 0 | 0 | 0 | 0 | 0 | 0 | 1 |
| UCP2 | 0 | 0 | 0 | 0 | 0 | 0 | 1 |
| DHRS7B | 0 | 0 | 0 | 0 | 0 | 0 | 1 |
| TMEM9B | 0 | 0 | 0 | 0 | 0 | 0 | 1 |
| WASHC5 | 0 | 0 | 0 | 0 | 0 | 0 | 1 |
| ARFIP2 | 0 | 0 | 0 | 0 | 0 | 0 | 1 |
| LOC100505915 | 0 | 0 | 0 | 0 | 0 | 0 | 1 |
| SSNA1 | 0 | 0 | 0 | 0 | 0 | 0 | 1 |
| OS9 | 0 | 0 | 0 | 0 | 0 | 0 | 1 |
| LOC654780 | 0 | 0 | 0 | 0 | 0 | 0 | 1 |
| ZBED5-AS1 | 0 | 0 | 0 | 0 | 0 | 0 | 1 |
| VAT1 | 0 | 0 | 0 | 0 | 0 | 0 | 1 |
| KLF3 | 0 | 0 | 0 | 0 | 0 | 0 | 1 |
| PTDSS1 | 0 | 0 | 0 | 0 | 0 | 0 | 1 |
| KAT5 | 0 | 0 | 0 | 0 | 0 | 0 | 1 |
| XRCC2 | 0 | 0 | 0 | 0 | 0 | 0 | 1 |
| PNISR | 0 | 0 | 0 | 0 | 0 | 0 | 1 |
| BAP1 | 0 | 0 | 0 | 0 | 0 | 0 | 1 |
| MAP2K2 | 0 | 0 | 0 | 0 | 0 | 0 | 1 |
| PRMT5 | 0 | 0 | 0 | 0 | 0 | 0 | 1 |
| TMEM41B | 0 | 0 | 0 | 0 | 0 | 0 | 1 |
| TCTA | 0 | 0 | 0 | 0 | 0 | 0 | 1 |
| ADRM1 | 0 | 0 | 0 | 0 | 0 | 0 | 1 |
| MICALL1 | 0 | 0 | 0 | 0 | 0 | 0 | 1 |
| DUSP23 | 0 | 0 | 0 | 0 | 0 | 0 | 1 |
| MZT2B | 0 | 0 | 0 | 0 | 0 | 0 | 1 |
| SLC48A1 | 0 | 0 | 0 | 0 | 0 | 0 | 1 |
| ALOX12 | 0 | 0 | 0 | 0 | 0 | 0 | 1 |
| LRRC69 | 0 | 0 | 0 | 0 | 0 | 0 | 1 |
| TMEM179B | 0 | 0 | 0 | 0 | 0 | 0 | 1 |
| SYNGR3 | 0 | 0 | 0 | 0 | 0 | 0 | 1 |
| ZNF213 | 0 | 0 | 0 | 0 | 0 | 0 | 1 |
| BIK | 0 | 0 | 0 | 0 | 0 | 0 | 1 |
| MEIS1 | 0 | 0 | 0 | 0 | 0 | 0 | 1 |
| IL32 | 0 | 0 | 0 | 0 | 0 | 0 | 1 |
| TMEM185B | 0 | 0 | 0 | 0 | 0 | 0 | 1 |
| PTGS1 | 0 | 0 | 0 | 0 | 0 | 0 | 1 |
| SH3GLB2 | 0 | 0 | 0 | 0 | 0 | 0 | 1 |
| DEDD2 | 0 | 0 | 0 | 0 | 0 | 0 | 1 |
| KCTD21 | 0 | 0 | 0 | 0 | 0 | 0 | 1 |
| FANCG | 0 | 0 | 0 | 0 | 0 | 0 | 1 |
| TCP11L2 | 0 | 0 | 0 | 0 | 0 | 0 | 1 |
| ZFYVE27 | 0 | 0 | 0 | 0 | 0 | 0 | 1 |
| HSP90AA1 | 0 | 0 | 0 | 0 | 0 | 0 | 1 |
| INPP5K | 0 | 0 | 0 | 0 | 0 | 0 | 1 |
| ASF1B | 0 | 0 | 0 | 0 | 0 | 0 | 1 |
| TMEM158 | 0 | 0 | 0 | 0 | 0 | 0 | 1 |
| H2BC7 | 0 | 0 | 0 | 0 | 0 | 0 | 1 |
| ABHD14A | 0 | 0 | 0 | 0 | 0 | 0 | 1 |
| CHMP6 | 0 | 0 | 0 | 0 | 0 | 0 | 1 |
| SRGAP2B | 0 | 0 | 0 | 0 | 0 | 0 | 1 |
| LGALSL | 0 | 0 | 0 | 0 | 0 | 0 | 1 |
| ZNF296 | 0 | 0 | 0 | 0 | 0 | 0 | 1 |
| MGC70870 | 0 | 0 | 0 | 0 | 0 | 0 | 1 |
| GET3 | 0 | 0 | 0 | 0 | 0 | 0 | 1 |
| FCGR2B | 0 | 0 | 0 | 0 | 0 | 0 | 1 |
| PPM1M | 0 | 0 | 0 | 0 | 0 | 0 | 1 |
| NEMF | 0 | 0 | 0 | 0 | 0 | 0 | 1 |
| LYL1 | 0 | 0 | 0 | 0 | 0 | 0 | 1 |
| TRAPPC1 | 0 | 0 | 0 | 0 | 0 | 0 | 1 |
| NDUFAF3 | 0 | 0 | 0 | 0 | 0 | 0 | 1 |
| NGDN | 0 | 0 | 0 | 0 | 0 | 0 | 1 |
| CAPN12 | 0 | 0 | 0 | 0 | 0 | 0 | 1 |
| SLC35A4 | 0 | 0 | 0 | 0 | 0 | 0 | 1 |
| BCAS3 | 0 | 0 | 0 | 0 | 0 | 0 | 1 |
| SH3GL3 | 0 | 0 | 0 | 0 | 0 | 0 | 1 |
| SLC38A5 | 0 | 0 | 0 | 0 | 0 | 0 | 1 |
| KCTD2 | 0 | 0 | 0 | 0 | 0 | 0 | 1 |
| PPP1R15A | 0 | 0 | 0 | 0 | 0 | 0 | 1 |
| DCTN1 | 0 | 0 | 0 | 0 | 0 | 0 | 1 |
| RNF41 | 0 | 0 | 0 | 0 | 0 | 0 | 1 |
| TANGO2 | 0 | 0 | 0 | 0 | 0 | 0 | 1 |
| ZNFX1 | 0 | 0 | 0 | 0 | 0 | 0 | 1 |
| ORMDL2 | 0 | 0 | 0 | 0 | 0 | 0 | 1 |
| NUDT2 | 0 | 0 | 0 | 0 | 0 | 0 | 1 |
| NT5M | 0 | 0 | 0 | 0 | 0 | 0 | 1 |
| VKORC1 | 0 | 0 | 0 | 0 | 0 | 0 | 1 |
| LINC01949 | 0 | 0 | 0 | 0 | 0 | 0 | 1 |
| CYP27A1 | 0 | 0 | 0 | 0 | 0 | 0 | 1 |
| LINC00597 | 0 | 0 | 0 | 0 | 0 | 0 | 1 |
| H2AW | 0 | 0 | 0 | 0 | 0 | 0 | 1 |
| POLL | 0 | 0 | 0 | 0 | 0 | 0 | 1 |
| CBX3 | 0 | 0 | 0 | 0 | 0 | 0 | 1 |
| TRIM10 | 0 | 0 | 0 | 0 | 0 | 0 | 1 |
| COG7 | 0 | 0 | 0 | 0 | 0 | 0 | 1 |
| ARHGAP6 | 0 | 0 | 0 | 0 | 0 | 0 | 1 |
| SMIM3 | 0 | 0 | 0 | 0 | 0 | 0 | 1 |
| POLA1 | 0 | 0 | 0 | 0 | 0 | 0 | 1 |
| MAP2K3 | 0 | 0 | 0 | 0 | 0 | 0 | 1 |
| PCGF5 | 0 | 0 | 0 | 0 | 0 | 0 | 1 |
| TNFAIP3 | 0 | 0 | 0 | 0 | 0 | 0 | 1 |
| TADA2B | 0 | 0 | 0 | 0 | 0 | 0 | 1 |
| NRGN | 0 | 0 | 0 | 0 | 0 | 0 | 1 |
| UBAC1 | 0 | 0 | 0 | 0 | 0 | 0 | 1 |
| TSPYL5 | 0 | 0 | 0 | 0 | 0 | 0 | 1 |
| PODNL1 | 0 | 0 | 0 | 0 | 0 | 0 | 1 |
| MCAT | 0 | 0 | 0 | 0 | 0 | 0 | 1 |
| CDKN1A | 0 | 0 | 0 | 0 | 0 | 0 | 1 |
| CYB5R3 | 0 | 0 | 0 | 0 | 0 | 0 | 1 |
| SLC25A39 | 0 | 0 | 0 | 0 | 0 | 0 | 1 |
| WDTC1 | 0 | 0 | 0 | 0 | 0 | 0 | 1 |
| MIR181A2HG | 0 | 0 | 0 | 0 | 0 | 0 | 1 |
| RTL6 | 0 | 0 | 0 | 0 | 0 | 0 | 1 |
| RNF19B | 0 | 0 | 0 | 0 | 0 | 0 | 1 |
| SHARPIN | 0 | 0 | 0 | 0 | 0 | 0 | 1 |
| FBXO7 | 0 | 0 | 0 | 0 | 0 | 0 | 1 |
| HECTD3 | 0 | 0 | 0 | 0 | 0 | 0 | 1 |
| GYPC | 0 | 0 | 0 | 0 | 0 | 0 | 1 |
| BAK1 | 0 | 0 | 0 | 0 | 0 | 0 | 1 |
| HPS1 | 0 | 0 | 0 | 0 | 0 | 0 | 1 |
| IMP4 | 0 | 0 | 0 | 0 | 0 | 0 | 1 |
| PGAP2 | 0 | 0 | 0 | 0 | 0 | 0 | 1 |
| TSPAN2 | 0 | 0 | 0 | 0 | 0 | 0 | 1 |
| POLD4 | 0 | 0 | 0 | 0 | 0 | 0 | 1 |
| TNFSF4 | 0 | 0 | 0 | 0 | 0 | 0 | 1 |
| ENDOD1 | 0 | 0 | 0 | 0 | 0 | 0 | 1 |
| SMIM1 | 0 | 0 | 0 | 0 | 0 | 0 | 1 |
| CRTC2 | 0 | 0 | 0 | 0 | 0 | 0 | 1 |
| DMTN | 0 | 0 | 0 | 0 | 0 | 0 | 1 |
| MARCHF2 | 0 | 0 | 0 | 0 | 0 | 0 | 1 |
| SIGLEC17P | 0 | 0 | 0 | 0 | 0 | 0 | 1 |
| ZFYVE21 | 0 | 0 | 0 | 0 | 0 | 0 | 1 |
| STK11 | 0 | 0 | 0 | 0 | 0 | 0 | 1 |
| IPO11 | 0 | 0 | 0 | 0 | 0 | 0 | 1 |
| IRF2 | 0 | 0 | 0 | 0 | 0 | 0 | 1 |
| TST | 0 | 0 | 0 | 0 | 0 | 0 | 1 |
| GCDH | 0 | 0 | 0 | 0 | 0 | 0 | 1 |
| KRT18 | 0 | 0 | 0 | 0 | 0 | 0 | 1 |
| IP6K1 | 0 | 0 | 0 | 0 | 0 | 0 | 1 |
| ZBED6CL | 0 | 0 | 0 | 0 | 0 | 0 | 1 |
| NELFE | 0 | 0 | 0 | 0 | 0 | 0 | 1 |
| CALCOCO1 | 0 | 0 | 0 | 0 | 0 | 0 | 1 |
| CPA3 | 0 | 0 | 0 | 0 | 0 | 0 | 1 |
| MYG1 | 0 | 0 | 0 | 0 | 0 | 0 | 1 |
| DHPS | 0 | 0 | 0 | 0 | 0 | 0 | 1 |
| TICAM1 | 0 | 0 | 0 | 0 | 0 | 0 | 1 |
| B9D2 | 0 | 0 | 0 | 0 | 0 | 0 | 1 |
| TOMM40L | 0 | 0 | 0 | 0 | 0 | 0 | 1 |
| POTEM | 0 | 0 | 0 | 0 | 0 | 0 | 1 |
| TUBG1 | 0 | 0 | 0 | 0 | 0 | 0 | 1 |
| MYADM | 0 | 0 | 0 | 0 | 0 | 0 | 1 |
| GPR160 | 0 | 0 | 0 | 0 | 0 | 0 | 1 |
| CXCR6 | 0 | 0 | 0 | 0 | 0 | 0 | 1 |
| OST4 | 0 | 0 | 0 | 0 | 0 | 0 | 1 |
| KIAA0040 | 0 | 0 | 0 | 0 | 0 | 0 | 1 |
| TAFA1 | 0 | 0 | 0 | 0 | 0 | 0 | 1 |
| NRM | 0 | 0 | 0 | 0 | 0 | 0 | 1 |
| C2orf88 | 0 | 0 | 0 | 0 | 0 | 0 | 1 |
| PLA2G15 | 0 | 0 | 0 | 0 | 0 | 0 | 1 |
| TPST2 | 0 | 0 | 0 | 0 | 0 | 0 | 1 |
| CCRL2 | 0 | 0 | 0 | 0 | 0 | 0 | 1 |
| TMEM44-AS1 | 0 | 0 | 0 | 0 | 0 | 0 | 1 |
| STIMATE | 0 | 0 | 0 | 0 | 0 | 0 | 1 |
| HEBP1 | 0 | 0 | 0 | 0 | 0 | 0 | 1 |
| TTC14 | 0 | 0 | 0 | 0 | 0 | 0 | 1 |
| CYSTM1 | 0 | 0 | 0 | 0 | 0 | 0 | 1 |
| SLAMF6 | 0 | 0 | 0 | 0 | 0 | 0 | 1 |
| DPF2 | 0 | 0 | 0 | 0 | 0 | 0 | 1 |
| PKIA | 0 | 0 | 0 | 0 | 0 | 0 | 1 |
| SURF2 | 0 | 0 | 0 | 0 | 0 | 0 | 1 |
| TMCO6 | 0 | 0 | 0 | 0 | 0 | 0 | 1 |
| STING1 | 0 | 0 | 0 | 0 | 0 | 0 | 1 |
| CCR3 | 0 | 0 | 0 | 0 | 0 | 0 | 1 |
| TM7SF2 | 0 | 0 | 0 | 0 | 0 | 0 | 1 |
| ARL2BP | 0 | 0 | 0 | 0 | 0 | 0 | 1 |
| LRRTM2 | 0 | 0 | 0 | 0 | 0 | 0 | 1 |
| CHTF8 | 0 | 0 | 0 | 0 | 0 | 0 | 1 |
| CDC34 | 0 | 0 | 0 | 0 | 0 | 0 | 1 |
| PIN4 | 0 | 0 | 0 | 0 | 0 | 0 | 1 |
| LINC00919 | 0 | 0 | 0 | 0 | 0 | 0 | 1 |
| BMX | 0 | 0 | 0 | 0 | 0 | 0 | 1 |
| CSNK2A1 | 0 | 0 | 0 | 0 | 0 | 0 | 1 |
| GBAP1 | 0 | 0 | 0 | 0 | 0 | 0 | 1 |
| LINC00342 | 0 | 0 | 0 | 0 | 0 | 0 | 1 |
| DENND1A | 0 | 0 | 0 | 0 | 0 | 0 | 1 |
| NEU1 | 0 | 0 | 0 | 0 | 0 | 0 | 1 |
| SNHG16 | 0 | 0 | 0 | 0 | 0 | 0 | 1 |
| FAM117A | 0 | 0 | 0 | 0 | 0 | 0 | 1 |
| TMEM140 | 0 | 0 | 0 | 0 | 0 | 0 | 1 |
| LINC02256 | 0 | 0 | 0 | 0 | 0 | 0 | 1 |
| ABCD1 | 0 | 0 | 0 | 0 | 0 | 0 | 1 |
| MICOS10P1 | 0 | 0 | 0 | 0 | 0 | 0 | 1 |
| NINJ1 | 0 | 0 | 0 | 0 | 0 | 0 | 1 |
| SHMT2 | 0 | 0 | 0 | 0 | 0 | 0 | 1 |
| AGAP9 | 0 | 0 | 0 | 0 | 0 | 0 | 1 |
| DUXAP8 | 0 | 0 | 0 | 0 | 0 | 0 | 1 |
| GFUS | 0 | 0 | 0 | 0 | 0 | 0 | 1 |
| PEF1 | 0 | 0 | 0 | 0 | 0 | 0 | 1 |
| TGFB1I1 | 0 | 0 | 0 | 0 | 0 | 0 | 1 |
| TREML2 | 0 | 0 | 0 | 0 | 0 | 0 | 1 |
| GNPDA1 | 0 | 0 | 0 | 0 | 0 | 0 | 1 |
| TSSC4 | 0 | 0 | 0 | 0 | 0 | 0 | 1 |
| C15orf39 | 0 | 0 | 0 | 0 | 0 | 0 | 1 |
| ZFAND3 | 0 | 0 | 0 | 0 | 0 | 0 | 1 |
| RNF10 | 0 | 0 | 0 | 0 | 0 | 0 | 1 |
| SH3RF3 | 0 | 0 | 0 | 0 | 0 | 0 | 1 |
| EPHA1-AS1 | 0 | 0 | 0 | 0 | 0 | 0 | 1 |
| ADIPOR1 | 0 | 0 | 0 | 0 | 0 | 0 | 1 |
| UVRAG | 0 | 0 | 0 | 0 | 0 | 0 | 1 |
| KAT7 | 0 | 0 | 0 | 0 | 0 | 0 | 1 |
| ZC3HC1 | 0 | 0 | 0 | 0 | 0 | 0 | 1 |
| COPZ1 | 0 | 0 | 0 | 0 | 0 | 0 | 1 |
| LINC02076 | 0 | 0 | 0 | 0 | 0 | 0 | 1 |
| DAD1 | 0 | 0 | 0 | 0 | 0 | 0 | 1 |
| PINK1 | 0 | 0 | 0 | 0 | 0 | 0 | 1 |
| BCKDK | 0 | 0 | 0 | 0 | 0 | 0 | 1 |
| FBXW4 | 0 | 0 | 0 | 0 | 0 | 0 | 1 |
| BCR | 0 | 0 | 0 | 0 | 0 | 0 | 1 |
| RBCK1 | 0 | 0 | 0 | 0 | 0 | 0 | 1 |
| EPM2A-DT | 0 | 0 | 0 | 0 | 0 | 0 | 1 |
| MXI1 | 0 | 0 | 0 | 0 | 0 | 0 | 1 |
| AIFM1 | 0 | 0 | 0 | 0 | 0 | 0 | 1 |
| GPKOW | 0 | 0 | 0 | 0 | 0 | 0 | 1 |
| MEIS3P1 | 0 | 0 | 0 | 0 | 0 | 0 | 1 |
| TFEB | 0 | 0 | 0 | 0 | 0 | 0 | 1 |
| SF3A2 | 0 | 0 | 0 | 0 | 0 | 0 | 1 |
| SEM1 | 0 | 0 | 0 | 0 | 0 | 0 | 1 |
| CHST7 | 0 | 0 | 0 | 0 | 0 | 0 | 1 |
| BAG1 | 0 | 0 | 0 | 0 | 0 | 0 | 1 |
| MPND | 0 | 0 | 0 | 0 | 0 | 0 | 1 |
| GPR146 | 0 | 0 | 0 | 0 | 0 | 0 | 1 |
| ABL1 | 0 | 0 | 0 | 0 | 0 | 0 | 1 |
| MLF2 | 0 | 0 | 0 | 0 | 0 | 0 | 1 |
| HSPB1 | 0 | 0 | 0 | 0 | 0 | 0 | 1 |
| STRN4 | 0 | 0 | 0 | 0 | 0 | 0 | 1 |
| PCGF1 | 0 | 0 | 0 | 0 | 0 | 0 | 1 |
| APRT | 0 | 0 | 0 | 0 | 0 | 0 | 1 |
| TCL1A | 0 | 0 | 0 | 0 | 0 | 0 | 1 |
| GDE1 | 0 | 0 | 0 | 0 | 0 | 0 | 1 |
| S100A11 | 0 | 0 | 0 | 0 | 0 | 0 | 1 |
| TPRG1L | 0 | 0 | 0 | 0 | 0 | 0 | 1 |
| AK1 | 0 | 0 | 0 | 0 | 0 | 0 | 1 |
| LOC441666 | 0 | 0 | 0 | 0 | 0 | 0 | 1 |
| BANF1 | 0 | 0 | 0 | 0 | 0 | 0 | 1 |
| UBE2M | 0 | 0 | 0 | 0 | 0 | 0 | 1 |
| CPQ | 0 | 0 | 0 | 0 | 0 | 0 | 1 |
| PARN | 0 | 0 | 0 | 0 | 0 | 0 | 1 |
| GSEC | 0 | 0 | 0 | 0 | 0 | 0 | 1 |
| LTBP2 | 0 | 0 | 0 | 0 | 0 | 0 | 1 |
| GLB1 | 0 | 0 | 0 | 0 | 0 | 0 | 1 |
| KIR2DS2 | 0 | 0 | 0 | 0 | 0 | 0 | 1 |
| SRM | 0 | 0 | 0 | 0 | 0 | 0 | 1 |
| RAB5C | 0 | 0 | 0 | 0 | 0 | 0 | 1 |
| NCAPD2 | 0 | 0 | 0 | 0 | 0 | 0 | 1 |
| TMEM86B | 0 | 0 | 0 | 0 | 0 | 0 | 1 |
| CDK2AP2 | 0 | 0 | 0 | 0 | 0 | 0 | 1 |
| PDIA5 | 0 | 0 | 0 | 0 | 0 | 0 | 1 |
| ST6GALNAC4 | 0 | 0 | 0 | 0 | 0 | 0 | 1 |
| H2BC12 | 0 | 0 | 0 | 0 | 0 | 0 | 1 |
| F13A1 | 0 | 0 | 0 | 0 | 0 | 0 | 1 |
| SGO2 | 0 | 0 | 0 | 0 | 0 | 0 | 1 |
| GIMAP7 | 0 | 0 | 0 | 0 | 0 | 0 | 1 |
| CTDSP2 | 0 | 0 | 0 | 0 | 0 | 0 | 1 |
| UBXN6 | 0 | 0 | 0 | 0 | 0 | 0 | 1 |
| MARCKSL1 | 0 | 0 | 0 | 0 | 0 | 0 | 1 |
| RNF182 | 0 | 0 | 0 | 0 | 0 | 0 | 1 |
| NUDT16L2P | 0 | 0 | 0 | 0 | 0 | 0 | 1 |
| RNF123 | 0 | 0 | 0 | 0 | 0 | 0 | 1 |
| ACRBP | 0 | 0 | 0 | 0 | 0 | 0 | 1 |
| C6orf136 | 0 | 0 | 0 | 0 | 0 | 0 | 1 |
| NFKBIE | 0 | 0 | 0 | 0 | 0 | 0 | 1 |
| CDCA4 | 0 | 0 | 0 | 0 | 0 | 0 | 1 |
| ATP6 | 0 | 0 | 0 | 0 | 0 | 0 | 1 |
| SLC6A8 | 0 | 0 | 0 | 0 | 0 | 0 | 1 |
| SLC25A1 | 0 | 0 | 0 | 0 | 0 | 0 | 1 |
| PPM1G | 0 | 0 | 0 | 0 | 0 | 0 | 1 |
| KTN1 | 0 | 0 | 0 | 0 | 0 | 0 | 1 |
| GRAP2 | 0 | 0 | 0 | 0 | 0 | 0 | 1 |
| ETHE1 | 0 | 0 | 0 | 0 | 0 | 0 | 1 |
| PGRMC1 | 0 | 0 | 0 | 0 | 0 | 0 | 1 |
| PIP4P1 | 0 | 0 | 0 | 0 | 0 | 0 | 1 |
| PIM1 | 0 | 0 | 0 | 0 | 0 | 0 | 1 |
| GPSM3 | 0 | 0 | 0 | 0 | 0 | 0 | 1 |
| CTSD | 0 | 0 | 0 | 0 | 0 | 0 | 1 |
| TLR4 | 0 | 0 | 0 | 0 | 0 | 0 | 1 |
| ARRB2 | 0 | 0 | 0 | 0 | 0 | 0 | 1 |
| GYS1 | 0 | 0 | 0 | 0 | 0 | 0 | 1 |
| ADA2 | 0 | 0 | 0 | 0 | 0 | 0 | 1 |
| AHSA2P | 0 | 0 | 0 | 0 | 0 | 0 | 1 |
| PCK2 | 0 | 0 | 0 | 0 | 0 | 0 | 1 |
| WDR54 | 0 | 0 | 0 | 0 | 0 | 0 | 1 |
| USP39 | 0 | 0 | 0 | 0 | 0 | 0 | 1 |
| KIFBP | 0 | 0 | 0 | 0 | 0 | 0 | 1 |
| ALAS1 | 0 | 0 | 0 | 0 | 0 | 0 | 1 |
| ISG20 | 0 | 0 | 0 | 0 | 0 | 0 | 1 |
| TCN1 | 0 | 0 | 0 | 0 | 0 | 0 | 1 |
| AP1M1 | 0 | 0 | 0 | 0 | 0 | 0 | 1 |
| MYL4 | 0 | 0 | 0 | 0 | 0 | 0 | 1 |
| TUBB6 | 0 | 0 | 0 | 0 | 0 | 0 | 1 |
| NPRL3 | 0 | 0 | 0 | 0 | 0 | 0 | 1 |
| ABCG1 | 0 | 0 | 0 | 0 | 0 | 0 | 1 |
| ASGR1 | 0 | 0 | 0 | 0 | 0 | 0 | 1 |
| RXRA | 0 | 0 | 0 | 0 | 0 | 0 | 1 |
| ADGRE1 | 0 | 0 | 0 | 0 | 0 | 0 | 1 |
| CORO1B | 0 | 0 | 0 | 0 | 0 | 0 | 1 |
| BAZ2B-AS1 | 0 | 0 | 0 | 0 | 0 | 0 | 1 |
| LOC100310756 | 0 | 0 | 0 | 0 | 0 | 0 | 1 |
| DPM1 | 0 | 0 | 0 | 0 | 0 | 0 | 1 |
| PRR5 | 0 | 0 | 0 | 0 | 0 | 0 | 1 |
| ABCF1 | 0 | 0 | 0 | 0 | 0 | 0 | 1 |
| MPP1 | 0 | 0 | 0 | 0 | 0 | 0 | 1 |
| ACKR1 | 0 | 0 | 0 | 0 | 0 | 0 | 1 |
| PLPPR2 | 0 | 0 | 0 | 0 | 0 | 0 | 1 |
| SAC3D1 | 0 | 0 | 0 | 0 | 0 | 0 | 1 |
| USP7-AS1 | 0 | 0 | 0 | 0 | 0 | 0 | 1 |
| CHPT1 | 0 | 0 | 0 | 0 | 0 | 0 | 1 |
| GAS2L1 | 0 | 0 | 0 | 0 | 0 | 0 | 1 |
| RBIS | 0 | 0 | 0 | 0 | 0 | 0 | 1 |
| FABP5 | 0 | 0 | 0 | 0 | 0 | 0 | 1 |
| FAXDC2 | 0 | 0 | 0 | 0 | 0 | 0 | 1 |
| KIR3DL1 | 0 | 0 | 0 | 0 | 0 | 0 | 1 |
| H2BC9 | 0 | 0 | 0 | 0 | 0 | 0 | 1 |
| LOC105379250 | 0 | 0 | 0 | 0 | 0 | 0 | 1 |
| MTX1 | 0 | 0 | 0 | 0 | 0 | 0 | 1 |
| LILRA1 | 0 | 0 | 0 | 0 | 0 | 0 | 1 |
| JAZF1 | 0 | 0 | 0 | 0 | 0 | 0 | 1 |
| RNPEPL1 | 0 | 0 | 0 | 0 | 0 | 0 | 1 |
| R3HDM4 | 0 | 0 | 0 | 0 | 0 | 0 | 1 |
| CBWD2 | 0 | 0 | 0 | 0 | 0 | 0 | 1 |
| LINC01857 | 0 | 0 | 0 | 0 | 0 | 0 | 1 |
| ATP5ME | 0 | 0 | 0 | 0 | 0 | 0 | 1 |
| SH2D2A | 0 | 0 | 0 | 0 | 0 | 0 | 1 |
| MYOM2 | 0 | 0 | 0 | 0 | 0 | 0 | 1 |
| MTLN | 0 | 0 | 0 | 0 | 0 | 0 | 1 |
| BTBD11 | 0 | 0 | 0 | 0 | 0 | 0 | 1 |
| OSGEP | 0 | 0 | 0 | 0 | 0 | 0 | 1 |
| CPNE2 | 0 | 0 | 0 | 0 | 0 | 0 | 1 |
| THTPA | 0 | 0 | 0 | 0 | 0 | 0 | 1 |
| FKBP1B | 0 | 0 | 0 | 0 | 0 | 0 | 1 |
| CHRAC1 | 0 | 0 | 0 | 0 | 0 | 0 | 1 |
| FLJ32255 | 0 | 0 | 0 | 0 | 0 | 0 | 1 |
| TGM2 | 0 | 0 | 0 | 0 | 0 | 0 | 1 |
| CA2 | 0 | 0 | 0 | 0 | 0 | 0 | 1 |
| STMP1 | 0 | 0 | 0 | 0 | 0 | 0 | 1 |
| TRAV13-1 | 0 | 0 | 0 | 0 | 0 | 0 | 1 |
| TAF15 | 0 | 0 | 0 | 0 | 0 | 0 | 1 |
| TMEM250 | 0 | 0 | 0 | 0 | 0 | 0 | 1 |
| ZNF479 | 0 | 0 | 0 | 0 | 0 | 0 | 1 |
| GALNT6 | 0 | 0 | 0 | 0 | 0 | 0 | 1 |
| MICB | 0 | 0 | 0 | 0 | 0 | 0 | 1 |
| FCRL4 | 0 | 0 | 0 | 0 | 0 | 0 | 1 |
| DBN1 | 0 | 0 | 0 | 0 | 0 | 0 | 1 |
| GUCY1B1 | 0 | 0 | 0 | 0 | 0 | 0 | 1 |
| H4C8 | 0 | 0 | 0 | 0 | 0 | 0 | 1 |
| HPCAL1 | 0 | 0 | 0 | 0 | 0 | 0 | 1 |
| MEST | 0 | 0 | 0 | 0 | 0 | 0 | 1 |
| OAZ2 | 0 | 0 | 0 | 0 | 0 | 0 | 1 |
| SELP | 0 | 0 | 0 | 0 | 0 | 0 | 1 |
| FBP1 | 0 | 0 | 0 | 0 | 0 | 0 | 1 |
| NCF4 | 0 | 0 | 0 | 0 | 0 | 0 | 1 |
| TRAV21 | 0 | 0 | 0 | 0 | 0 | 0 | 1 |
| SAP130 | 0 | 0 | 0 | 0 | 0 | 0 | 1 |
| HYMAI | 0 | 0 | 0 | 0 | 0 | 0 | 1 |
| ASPHD2 | 0 | 0 | 0 | 0 | 0 | 0 | 1 |
| KEAP1 | 0 | 0 | 0 | 0 | 0 | 0 | 1 |
| PDZK1IP1 | 0 | 0 | 0 | 0 | 0 | 0 | 1 |
| NEURL4 | 0 | 0 | 0 | 0 | 0 | 0 | 1 |
| CDA | 0 | 0 | 0 | 0 | 0 | 0 | 1 |
| LSP1 | 0 | 0 | 0 | 0 | 0 | 0 | 1 |
| DNAJB2 | 0 | 0 | 0 | 0 | 0 | 0 | 1 |
| UBALD2 | 0 | 0 | 0 | 0 | 0 | 0 | 1 |
| SFRP2 | 0 | 0 | 0 | 0 | 0 | 0 | 1 |
| HBM | 0 | 0 | 0 | 0 | 0 | 0 | 1 |
| EIF2AK1 | 0 | 0 | 0 | 0 | 0 | 0 | 1 |
| TREX1 | 0 | 0 | 0 | 0 | 0 | 0 | 1 |
| ROGDI | 0 | 0 | 0 | 0 | 0 | 0 | 1 |
| MMD | 0 | 0 | 0 | 0 | 0 | 0 | 1 |
| GTSF1 | 0 | 0 | 0 | 0 | 0 | 0 | 1 |
| PDCD6P1 | 0 | 0 | 0 | 0 | 0 | 0 | 1 |
| SLC20A2 | 0 | 0 | 0 | 0 | 0 | 0 | 1 |
| ELOF1 | 0 | 0 | 0 | 0 | 0 | 0 | 1 |
| GUCD1 | 0 | 0 | 0 | 0 | 0 | 0 | 1 |
| INAFM1 | 0 | 0 | 0 | 0 | 0 | 0 | 1 |
| PUDP | 0 | 0 | 0 | 0 | 0 | 0 | 1 |
| RUVBL1 | 0 | 0 | 0 | 0 | 0 | 0 | 1 |
| MSRB1 | 0 | 0 | 0 | 0 | 0 | 0 | 1 |
| STRADB | 0 | 0 | 0 | 0 | 0 | 0 | 1 |
| ANKRD9 | 0 | 0 | 0 | 0 | 0 | 0 | 1 |
| LOC105379173 | 0 | 0 | 0 | 0 | 0 | 0 | 1 |
| POLR1D | 0 | 0 | 0 | 0 | 0 | 0 | 1 |
| ZBTB8OS | 0 | 0 | 0 | 0 | 0 | 0 | 1 |
| SSB | 0 | 0 | 0 | 0 | 0 | 0 | 1 |
| RHOG | 0 | 0 | 0 | 0 | 0 | 0 | 1 |
| NOMO3 | 0 | 0 | 0 | 0 | 0 | 0 | 1 |
| PSMC6 | 0 | 0 | 0 | 0 | 0 | 0 | 1 |
| DCXR | 0 | 0 | 0 | 0 | 0 | 0 | 1 |
| GPAT2 | 0 | 0 | 0 | 0 | 0 | 0 | 1 |
| ALDOAP2 | 0 | 0 | 0 | 0 | 0 | 0 | 1 |
| WDR45 | 0 | 0 | 0 | 0 | 0 | 0 | 1 |
| PGM1 | 0 | 0 | 0 | 0 | 0 | 0 | 1 |
| CR1L | 0 | 0 | 0 | 0 | 0 | 0 | 1 |
| RBM23 | 0 | 0 | 0 | 0 | 0 | 0 | 1 |
| MAGOH | 0 | 0 | 0 | 0 | 0 | 0 | 1 |
| FGF7P3 | 0 | 0 | 0 | 0 | 0 | 0 | 1 |
| C12orf43 | 0 | 0 | 0 | 0 | 0 | 0 | 1 |
| YIPF3 | 0 | 0 | 0 | 0 | 0 | 0 | 1 |
| STAT6 | 0 | 0 | 0 | 0 | 0 | 0 | 1 |
| RASSF10 | 0 | 0 | 0 | 0 | 0 | 0 | 1 |
| PSENEN | 0 | 0 | 0 | 0 | 0 | 0 | 1 |
| U2AF1L4 | 0 | 0 | 0 | 0 | 0 | 0 | 1 |
| C2orf42 | 0 | 0 | 0 | 0 | 0 | 0 | 1 |
| SLC15A3 | 0 | 0 | 0 | 0 | 0 | 0 | 1 |
| CRYL1 | 0 | 0 | 0 | 0 | 0 | 0 | 1 |
| HOXC6 | 0 | 0 | 0 | 0 | 0 | 0 | 1 |
| KIR3DL3 | 0 | 0 | 0 | 0 | 0 | 0 | 1 |
| MST1L | 0 | 0 | 0 | 0 | 0 | 0 | 1 |
| UBAP1 | 0 | 0 | 0 | 0 | 0 | 0 | 1 |
| STX5 | 0 | 0 | 0 | 0 | 0 | 0 | 1 |
| PLD3 | 0 | 0 | 0 | 0 | 0 | 0 | 1 |
| PNP | 0 | 0 | 0 | 0 | 0 | 0 | 1 |
| ATIC | 0 | 0 | 0 | 0 | 0 | 0 | 1 |
| ASCC2 | 0 | 0 | 0 | 0 | 0 | 0 | 1 |
| SHKBP1 | 0 | 0 | 0 | 0 | 0 | 0 | 1 |
| ELMO1 | 0 | 0 | 0 | 0 | 0 | 0 | 1 |
| MT1X | 0 | 0 | 0 | 0 | 0 | 0 | 1 |
| HECW2-AS1 | 0 | 0 | 0 | 0 | 0 | 0 | 1 |
| ZNF542P | 0 | 0 | 0 | 0 | 0 | 0 | 1 |
| ZC3H15 | 0 | 0 | 0 | 0 | 0 | 0 | 1 |
| PSMB9 | 0 | 0 | 0 | 0 | 0 | 0 | 1 |
| LRFN1 | 0 | 0 | 0 | 0 | 0 | 0 | 1 |
| KBTBD3 | 0 | 0 | 0 | 0 | 0 | 0 | 1 |
| H2BC10 | 0 | 0 | 0 | 0 | 0 | 0 | 1 |
| MAP7D1 | 0 | 0 | 0 | 0 | 0 | 0 | 1 |
| LOC105371967 | 0 | 0 | 0 | 0 | 0 | 0 | 1 |
| ITGA2B | 0 | 0 | 0 | 0 | 0 | 0 | 1 |
| H1-2 | 0 | 0 | 0 | 0 | 0 | 0 | 1 |
| UQCRB | 0 | 0 | 0 | 0 | 0 | 0 | 1 |
| CXCL8 | 0 | 0 | 0 | 0 | 0 | 0 | 1 |
| ZNF628 | 0 | 0 | 0 | 0 | 0 | 0 | 1 |
| RECQL | 0 | 0 | 0 | 0 | 0 | 0 | 1 |
| KLHDC8B | 0 | 0 | 0 | 0 | 0 | 0 | 1 |
| EVA1C | 0 | 0 | 0 | 0 | 0 | 0 | 1 |
| PRKAR2B | 0 | 0 | 0 | 0 | 0 | 0 | 1 |
| RRAGD | 0 | 0 | 0 | 0 | 0 | 0 | 1 |
| IPO4 | 0 | 0 | 0 | 0 | 0 | 0 | 1 |
| ROPN1L | 0 | 0 | 0 | 0 | 0 | 0 | 1 |
| TIGD3 | 0 | 0 | 0 | 0 | 0 | 0 | 1 |
| RABAC1 | 0 | 0 | 0 | 0 | 0 | 0 | 1 |
| P2RY12 | 0 | 0 | 0 | 0 | 0 | 0 | 1 |
| SRRD | 0 | 0 | 0 | 0 | 0 | 0 | 1 |
| HBQ1 | 0 | 0 | 0 | 0 | 0 | 0 | 1 |
| SIRPB2 | 0 | 0 | 0 | 0 | 0 | 0 | 1 |
| CLU | 0 | 0 | 0 | 0 | 0 | 0 | 1 |
| HTT | 0 | 0 | 0 | 0 | 0 | 0 | 1 |
| UBL7 | 0 | 0 | 0 | 0 | 0 | 0 | 1 |
| TESC | 0 | 0 | 0 | 0 | 0 | 0 | 1 |
| TMEM268 | 0 | 0 | 0 | 0 | 0 | 0 | 1 |
| VPS51 | 0 | 0 | 0 | 0 | 0 | 0 | 1 |
| MFSD5 | 0 | 0 | 0 | 0 | 0 | 0 | 1 |
| GBP4 | 0 | 0 | 0 | 0 | 0 | 0 | 1 |
| CCNJL | 0 | 0 | 0 | 0 | 0 | 0 | 1 |
| LOC101927166 | 0 | 0 | 0 | 0 | 0 | 0 | 1 |
| SLC43A1 | 0 | 0 | 0 | 0 | 0 | 0 | 1 |
| PEX6 | 0 | 0 | 0 | 0 | 0 | 0 | 1 |
| FXYD6 | 0 | 0 | 0 | 0 | 0 | 0 | 1 |
| RAD23A | 0 | 0 | 0 | 0 | 0 | 0 | 1 |
| TRAPPC10 | 0 | 0 | 0 | 0 | 0 | 0 | 1 |
| CHI3L1 | 0 | 0 | 0 | 0 | 0 | 0 | 1 |
| HAGH | 0 | 0 | 0 | 0 | 0 | 0 | 1 |
| ANK1 | 0 | 0 | 0 | 0 | 0 | 0 | 1 |
| FLOT2 | 0 | 0 | 0 | 0 | 0 | 0 | 1 |
| LBH | 0 | 0 | 0 | 0 | 0 | 0 | 1 |
| VAMP2 | 0 | 0 | 0 | 0 | 0 | 0 | 1 |
| RPF2 | 0 | 0 | 0 | 0 | 0 | 0 | 1 |
| PUSL1 | 0 | 0 | 0 | 0 | 0 | 0 | 1 |
| SPDYE1 | 0 | 0 | 0 | 0 | 0 | 0 | 1 |
| TRAPPC5 | 0 | 0 | 0 | 0 | 0 | 0 | 1 |
| SNRPE | 0 | 0 | 0 | 0 | 0 | 0 | 1 |
| TRIM21 | 0 | 0 | 0 | 0 | 0 | 0 | 1 |
| PPP2R5B | 0 | 0 | 0 | 0 | 0 | 0 | 1 |
| ATP6V1B2 | 0 | 0 | 0 | 0 | 0 | 0 | 1 |
| DPP4 | 0 | 0 | 0 | 0 | 0 | 0 | 1 |
| SIAH2 | 0 | 0 | 0 | 0 | 0 | 0 | 1 |
| GUK1 | 0 | 0 | 0 | 0 | 0 | 0 | 1 |
| FRMD3 | 0 | 0 | 0 | 0 | 0 | 0 | 1 |
| ZNF581 | 0 | 0 | 0 | 0 | 0 | 0 | 1 |
| TMOD1 | 0 | 0 | 0 | 0 | 0 | 0 | 1 |
| ZNF808 | 0 | 0 | 0 | 0 | 0 | 0 | 1 |
| HDGF | 0 | 0 | 0 | 0 | 0 | 0 | 1 |
| LINC00339 | 0 | 0 | 0 | 0 | 0 | 0 | 1 |
| CTSA | 0 | 0 | 0 | 0 | 0 | 0 | 1 |
| HAUS4 | 0 | 0 | 0 | 0 | 0 | 0 | 1 |
| MT1F | 0 | 0 | 0 | 0 | 0 | 0 | 1 |
| DSPP | 0 | 0 | 0 | 0 | 0 | 0 | 1 |
| CD7 | 0 | 0 | 0 | 0 | 0 | 0 | 1 |
| RANBP10 | 0 | 0 | 0 | 0 | 0 | 0 | 1 |
| TRIM58 | 0 | 0 | 0 | 0 | 0 | 0 | 1 |
| GATA1 | 0 | 0 | 0 | 0 | 0 | 0 | 1 |
| PNPLA6 | 0 | 0 | 0 | 0 | 0 | 0 | 1 |
| ENO2 | 0 | 0 | 0 | 0 | 0 | 0 | 1 |
| PGLYRP1 | 0 | 0 | 0 | 0 | 0 | 0 | 1 |
| ADORA3 | 0 | 0 | 0 | 0 | 0 | 0 | 1 |
| HLA-DMA | 0 | 0 | 0 | 0 | 0 | 0 | 1 |
| TMTC1 | 0 | 0 | 0 | 0 | 0 | 0 | 1 |
| CLEC1B | 0 | 0 | 0 | 0 | 0 | 0 | 1 |
| HSF5 | 0 | 0 | 0 | 0 | 0 | 0 | 1 |
| LRRC25 | 0 | 0 | 0 | 0 | 0 | 0 | 1 |
| YPEL4 | 0 | 0 | 0 | 0 | 0 | 0 | 1 |
| SH3BGRL2 | 0 | 0 | 0 | 0 | 0 | 0 | 1 |
| POR | 0 | 0 | 0 | 0 | 0 | 0 | 1 |
| CHURC1 | 0 | 0 | 0 | 0 | 0 | 0 | 1 |
| OCEL1 | 0 | 0 | 0 | 0 | 0 | 0 | 1 |
| AMIGO1 | 0 | 0 | 0 | 0 | 0 | 0 | 1 |
| NDUFS5 | 0 | 0 | 0 | 0 | 0 | 0 | 1 |
| SPATC1L | 0 | 0 | 0 | 0 | 0 | 0 | 1 |
| GRAMD1C | 0 | 0 | 0 | 0 | 0 | 0 | 1 |
| TGM3 | 0 | 0 | 0 | 0 | 0 | 0 | 1 |
| ACCS | 0 | 0 | 0 | 0 | 0 | 0 | 1 |
| BCL2L1 | 0 | 0 | 0 | 0 | 0 | 0 | 1 |
| SLC31A2 | 0 | 0 | 0 | 0 | 0 | 0 | 1 |
| FAHD1 | 0 | 0 | 0 | 0 | 0 | 0 | 1 |
| RAD21-AS1 | 0 | 0 | 0 | 0 | 0 | 0 | 1 |
| CAVIN2 | 0 | 0 | 0 | 0 | 0 | 0 | 1 |
| GPX1 | 0 | 0 | 0 | 0 | 0 | 0 | 1 |
| S100P | 0 | 0 | 0 | 0 | 0 | 0 | 1 |
| PRDX5 | 0 | 0 | 0 | 0 | 0 | 0 | 1 |
| TRANK1 | 0 | 0 | 0 | 0 | 0 | 0 | 1 |
| WBP2 | 0 | 0 | 0 | 0 | 0 | 0 | 1 |
| HMBS | 0 | 0 | 0 | 0 | 0 | 0 | 1 |
| NOP2 | 0 | 0 | 0 | 0 | 0 | 0 | 1 |
| PRKCD | 0 | 0 | 0 | 0 | 0 | 0 | 1 |
| H2BC6 | 0 | 0 | 0 | 0 | 0 | 0 | 1 |
| CD3E | 0 | 0 | 0 | 0 | 0 | 0 | 1 |
| GMPPA | 0 | 0 | 0 | 0 | 0 | 0 | 1 |
| MAF1 | 0 | 0 | 0 | 0 | 0 | 0 | 1 |
| PSMF1 | 0 | 0 | 0 | 0 | 0 | 0 | 1 |
| TREML1 | 0 | 0 | 0 | 0 | 0 | 0 | 1 |
| DPEP3 | 0 | 0 | 0 | 0 | 0 | 0 | 1 |
| DANCR | 0 | 0 | 0 | 0 | 0 | 0 | 1 |
| TNS1 | 0 | 0 | 0 | 0 | 0 | 0 | 1 |
| H2AC6 | 0 | 0 | 0 | 0 | 0 | 0 | 1 |
| MFSD13A | 0 | 0 | 0 | 0 | 0 | 0 | 1 |
| KRT1 | 0 | 0 | 0 | 0 | 0 | 0 | 1 |
| PIGU | 0 | 0 | 0 | 0 | 0 | 0 | 1 |
| TBCA | 0 | 0 | 0 | 0 | 0 | 0 | 1 |
| PLEKHF1 | 0 | 0 | 0 | 0 | 0 | 0 | 1 |
| RAB5B | 0 | 0 | 0 | 0 | 0 | 0 | 1 |
| ANKRD55 | 0 | 0 | 0 | 0 | 0 | 0 | 1 |
| IFRD2 | 0 | 0 | 0 | 0 | 0 | 0 | 1 |
| ITPRIPL1 | 0 | 0 | 0 | 0 | 0 | 0 | 1 |
| GLT1D1 | 0 | 0 | 0 | 0 | 0 | 0 | 1 |
| DCAF12 | 0 | 0 | 0 | 0 | 0 | 0 | 1 |
| POLD2 | 0 | 0 | 0 | 0 | 0 | 0 | 1 |
| ZER1 | 0 | 0 | 0 | 0 | 0 | 0 | 1 |
| SLC27A3 | 0 | 0 | 0 | 0 | 0 | 0 | 1 |
| SAMD12 | 0 | 0 | 0 | 0 | 0 | 0 | 1 |
| MICAL2 | 0 | 0 | 0 | 0 | 0 | 0 | 1 |
| ITLN1 | 0 | 0 | 0 | 0 | 0 | 0 | 1 |
| MYL6B | 0 | 0 | 0 | 0 | 0 | 0 | 1 |
| BBOF1 | 0 | 0 | 0 | 0 | 0 | 0 | 1 |
| KIR2DS5 | 0 | 0 | 0 | 0 | 0 | 0 | 1 |
| ASPRV1 | 0 | 0 | 0 | 0 | 0 | 0 | 1 |
| IL15RA | 0 | 0 | 0 | 0 | 0 | 0 | 1 |
| MBNL3 | 0 | 0 | 0 | 0 | 0 | 0 | 1 |
| CSF1R | 0 | 0 | 0 | 0 | 0 | 0 | 1 |
| CD274 | 0 | 0 | 0 | 0 | 0 | 0 | 1 |
| EIF2D | 0 | 0 | 0 | 0 | 0 | 0 | 1 |
| KLF1 | 0 | 0 | 0 | 0 | 0 | 0 | 1 |
| MYH9 | 0 | 0 | 0 | 0 | 0 | 0 | 1 |
| CWC15 | 0 | 0 | 0 | 0 | 0 | 0 | 1 |
| CFP | 0 | 0 | 0 | 0 | 0 | 0 | 1 |
| ITPRIP | 0 | 0 | 0 | 0 | 0 | 0 | 1 |
| SNORA21 | 0 | 0 | 0 | 0 | 0 | 0 | 1 |
| TENT5C | 0 | 0 | 0 | 0 | 0 | 0 | 1 |
| GSTM2 | 0 | 0 | 0 | 0 | 0 | 0 | 1 |
| EPB42 | 0 | 0 | 0 | 0 | 0 | 0 | 1 |
| DHRS3 | 0 | 0 | 0 | 0 | 0 | 0 | 1 |
| LOC389831 | 0 | 0 | 0 | 0 | 0 | 0 | 1 |
| DHRS13 | 0 | 0 | 0 | 0 | 0 | 0 | 1 |
| LOC105377782 | 0 | 0 | 0 | 0 | 0 | 0 | 1 |
| DYSF | 0 | 0 | 0 | 0 | 0 | 0 | 1 |
| PFDN4 | 0 | 0 | 0 | 0 | 0 | 0 | 1 |
| PDZD11 | 0 | 0 | 0 | 0 | 0 | 0 | 1 |
| NDUFA1 | 0 | 0 | 0 | 0 | 0 | 0 | 1 |
| FAM214B | 0 | 0 | 0 | 0 | 0 | 0 | 1 |
| HMGN3 | 0 | 0 | 0 | 0 | 0 | 0 | 1 |
| FIS1 | 0 | 0 | 0 | 0 | 0 | 0 | 1 |
| FCMR | 0 | 0 | 0 | 0 | 0 | 0 | 1 |
| ZNF600 | 0 | 0 | 0 | 0 | 0 | 0 | 1 |
| HSPE1 | 0 | 0 | 0 | 0 | 0 | 0 | 1 |
| ZNF57 | 0 | 0 | 0 | 0 | 0 | 0 | 1 |
| ABHD14B | 0 | 0 | 0 | 0 | 0 | 0 | 1 |
| BPI | 0 | 0 | 0 | 0 | 0 | 0 | 1 |
| OXTR | 0 | 0 | 0 | 0 | 0 | 0 | 1 |
| HBG2 | 0 | 0 | 0 | 0 | 0 | 0 | 1 |
| EEF1B2 | 0 | 0 | 0 | 0 | 0 | 0 | 1 |
| CTNNAL1 | 0 | 0 | 0 | 0 | 0 | 0 | 1 |
| YBX3 | 0 | 0 | 0 | 0 | 0 | 0 | 1 |
| MED22 | 0 | 0 | 0 | 0 | 0 | 0 | 1 |
| DGAT2 | 0 | 0 | 0 | 0 | 0 | 0 | 1 |
| CLEC4D | 0 | 0 | 0 | 0 | 0 | 0 | 1 |
| LOC101928893 | 0 | 0 | 0 | 0 | 0 | 0 | 1 |
| GMPR | 0 | 0 | 0 | 0 | 0 | 0 | 1 |
| PCSK1N | 0 | 0 | 0 | 0 | 0 | 0 | 1 |
| SHCBP1 | 0 | 0 | 0 | 0 | 0 | 0 | 1 |
| BABAM1 | 0 | 0 | 0 | 0 | 0 | 0 | 1 |
| HLX | 0 | 0 | 0 | 0 | 0 | 0 | 1 |
| BLVRB | 0 | 0 | 0 | 0 | 0 | 0 | 1 |
| STX11 | 0 | 0 | 0 | 0 | 0 | 0 | 1 |
| LRG1 | 0 | 0 | 0 | 0 | 0 | 0 | 1 |
| OSBP2 | 0 | 0 | 0 | 0 | 0 | 0 | 1 |
| TATDN1 | 0 | 0 | 0 | 0 | 0 | 0 | 1 |
| LSM8 | 0 | 0 | 0 | 0 | 0 | 0 | 1 |
| OPTN | 0 | 0 | 0 | 0 | 0 | 0 | 1 |
| ABHD15 | 0 | 0 | 0 | 0 | 0 | 0 | 1 |
| PCTP | 0 | 0 | 0 | 0 | 0 | 0 | 1 |
| SELENOM | 0 | 0 | 0 | 0 | 0 | 0 | 1 |
| H2BC5 | 0 | 0 | 0 | 0 | 0 | 0 | 1 |
| PDCD4-AS1 | 0 | 0 | 0 | 0 | 0 | 0 | 1 |
| NFU1 | 0 | 0 | 0 | 0 | 0 | 0 | 1 |
| SMIM30 | 0 | 0 | 0 | 0 | 0 | 0 | 1 |
| AK6 | 0 | 0 | 0 | 0 | 0 | 0 | 1 |
| ADGRE3 | 0 | 0 | 0 | 0 | 0 | 0 | 1 |
| SLPI | 0 | 0 | 0 | 0 | 0 | 0 | 1 |
| WLS | 0 | 0 | 0 | 0 | 0 | 0 | 1 |
| CETN3 | 0 | 0 | 0 | 0 | 0 | 0 | 1 |
| NME4 | 0 | 0 | 0 | 0 | 0 | 0 | 1 |
| BATF2 | 0 | 0 | 0 | 0 | 0 | 0 | 1 |
| C2orf74 | 0 | 0 | 0 | 0 | 0 | 0 | 1 |
| LINC01527 | 0 | 0 | 0 | 0 | 0 | 0 | 1 |
| ODC1 | 0 | 0 | 0 | 0 | 0 | 0 | 1 |
| PIP4K2A | 0 | 0 | 0 | 0 | 0 | 0 | 1 |
| HAT1 | 0 | 0 | 0 | 0 | 0 | 0 | 1 |
| RGL4 | 0 | 0 | 0 | 0 | 0 | 0 | 1 |
| KLRG1 | 0 | 0 | 0 | 0 | 0 | 0 | 1 |
| PCED1B | 0 | 0 | 0 | 0 | 0 | 0 | 1 |
| TMEM91 | 0 | 0 | 0 | 0 | 0 | 0 | 1 |
| XK | 0 | 0 | 0 | 0 | 0 | 0 | 1 |
| LINC02481 | 0 | 0 | 0 | 0 | 0 | 0 | 1 |
| E2F2 | 0 | 0 | 0 | 0 | 0 | 0 | 1 |
| B3GNT8 | 0 | 0 | 0 | 0 | 0 | 0 | 1 |
| PRXL2B | 0 | 0 | 0 | 0 | 0 | 0 | 1 |
| PLEK2 | 0 | 0 | 0 | 0 | 0 | 0 | 1 |
| RELB | 0 | 0 | 0 | 0 | 0 | 0 | 1 |
| EIF1AY | 0 | 0 | 0 | 0 | 0 | 0 | 1 |
| MAP3K7CL | 0 | 0 | 0 | 0 | 0 | 0 | 1 |
| NFKB1 | 0 | 0 | 0 | 0 | 0 | 0 | 1 |
| CLEC12B | 0 | 0 | 0 | 0 | 0 | 0 | 1 |
| FHIT | 0 | 0 | 0 | 0 | 0 | 0 | 1 |
| ADGRG3 | 0 | 0 | 0 | 0 | 0 | 0 | 1 |
| AHSP | 0 | 0 | 0 | 0 | 0 | 0 | 1 |
| FHL2 | 0 | 0 | 0 | 0 | 0 | 0 | 1 |
| FAM104A | 0 | 0 | 0 | 0 | 0 | 0 | 1 |
| RHBDF2 | 0 | 0 | 0 | 0 | 0 | 0 | 1 |
| LHFPL2 | 0 | 0 | 0 | 0 | 0 | 0 | 1 |
| PSMA4 | 0 | 0 | 0 | 0 | 0 | 0 | 1 |
| SCN1B | 0 | 0 | 0 | 0 | 0 | 0 | 1 |
| PLVAP | 0 | 0 | 0 | 0 | 0 | 0 | 1 |
| DPM2 | 0 | 0 | 0 | 0 | 0 | 0 | 1 |
| ERV3-1 | 0 | 0 | 0 | 0 | 0 | 0 | 1 |
| TSPAN5 | 0 | 0 | 0 | 0 | 0 | 0 | 1 |
| SNRPD1 | 0 | 0 | 0 | 0 | 0 | 0 | 1 |
| OTUD6B-AS1 | 0 | 0 | 0 | 0 | 0 | 0 | 1 |
| TPRKB | 0 | 0 | 0 | 0 | 0 | 0 | 1 |
| GYPB | 0 | 0 | 0 | 0 | 0 | 0 | 1 |
| MAD2L1BP | 0 | 0 | 0 | 0 | 0 | 0 | 1 |
| LOC105375492 | 0 | 0 | 0 | 0 | 0 | 0 | 1 |
| SARNP | 0 | 0 | 0 | 0 | 0 | 0 | 1 |
| TNFSF13 | 0 | 0 | 0 | 0 | 0 | 0 | 1 |
| RWDD3 | 0 | 0 | 0 | 0 | 0 | 0 | 1 |
| ARMCX2 | 0 | 0 | 0 | 0 | 0 | 0 | 1 |
| SELENBP1 | 0 | 0 | 0 | 0 | 0 | 0 | 1 |
| RPL26L1 | 0 | 0 | 0 | 0 | 0 | 0 | 1 |
| LRRC4 | 0 | 0 | 0 | 0 | 0 | 0 | 1 |
| RPL22L1 | 0 | 0 | 0 | 0 | 0 | 0 | 1 |
| TMA7 | 0 | 0 | 0 | 0 | 0 | 0 | 1 |
| CASC3 | 0 | 0 | 0 | 0 | 0 | 0 | 1 |
| CMC1 | 0 | 0 | 0 | 0 | 0 | 0 | 1 |
| UQCRH | 0 | 0 | 0 | 0 | 0 | 0 | 1 |
| P2RX7 | 0 | 0 | 0 | 0 | 0 | 0 | 1 |
| CTSB | 0 | 0 | 0 | 0 | 0 | 0 | 1 |
| SECTM1 | 0 | 0 | 0 | 0 | 0 | 0 | 1 |
| ABCC13 | 0 | 0 | 0 | 0 | 0 | 0 | 1 |
| P2RY14 | 0 | 0 | 0 | 0 | 0 | 0 | 1 |
| MARCHF8 | 0 | 0 | 0 | 0 | 0 | 0 | 1 |
| NINJ2 | 0 | 0 | 0 | 0 | 0 | 0 | 1 |
| MRC2 | 0 | 0 | 0 | 0 | 0 | 0 | 1 |
| WARS1 | 0 | 0 | 0 | 0 | 0 | 0 | 1 |
| OASL | 0 | 0 | 0 | 0 | 0 | 0 | 1 |
| NAPSB | 0 | 0 | 0 | 0 | 0 | 0 | 1 |
| FBXO6 | 0 | 0 | 0 | 0 | 0 | 0 | 1 |
| FAM106A | 0 | 0 | 0 | 0 | 0 | 0 | 1 |
| KIR3DL2 | 0 | 0 | 0 | 0 | 0 | 0 | 1 |
| SLC7A5 | 0 | 0 | 0 | 0 | 0 | 0 | 1 |
| RBM38 | 0 | 0 | 0 | 0 | 0 | 0 | 1 |
| CEACAM21 | 0 | 0 | 0 | 0 | 0 | 0 | 1 |
| COX16 | 0 | 0 | 0 | 0 | 0 | 0 | 1 |
| SNRPG | 0 | 0 | 0 | 0 | 0 | 0 | 1 |
| MKRN1 | 0 | 0 | 0 | 0 | 0 | 0 | 1 |
| BSG | 0 | 0 | 0 | 0 | 0 | 0 | 1 |
| LTF | 0 | 0 | 0 | 0 | 0 | 0 | 1 |
| COX6C | 0 | 0 | 0 | 0 | 0 | 0 | 1 |
| RWDD1 | 0 | 0 | 0 | 0 | 0 | 0 | 1 |
| CTSL | 0 | 0 | 0 | 0 | 0 | 0 | 1 |
| PI3 | 0 | 0 | 0 | 0 | 0 | 0 | 1 |
| GLRX5 | 0 | 0 | 0 | 0 | 0 | 0 | 1 |
| PF4 | 0 | 0 | 0 | 0 | 0 | 0 | 1 |
| EMC2 | 0 | 0 | 0 | 0 | 0 | 0 | 1 |
| RSL24D1 | 0 | 0 | 0 | 0 | 0 | 0 | 1 |
| BTNL3 | 0 | 0 | 0 | 0 | 0 | 0 | 1 |
| LAMP3 | 0 | 0 | 0 | 0 | 0 | 0 | 1 |
| EPHX2 | 0 | 0 | 0 | 0 | 0 | 0 | 1 |
| RPIA | 0 | 0 | 0 | 0 | 0 | 0 | 1 |
| CEACAM8 | 0 | 0 | 0 | 0 | 0 | 0 | 1 |
| SDCBPP2 | 0 | 0 | 0 | 0 | 0 | 0 | 1 |
| FECH | 0 | 0 | 0 | 0 | 0 | 0 | 1 |
| RPS7 | 0 | 0 | 0 | 0 | 0 | 0 | 1 |
| TUBB1 | 0 | 0 | 0 | 0 | 0 | 0 | 1 |
| LY6E | 0 | 0 | 0 | 0 | 0 | 0 | 1 |
| MRPS28 | 0 | 0 | 0 | 0 | 0 | 0 | 1 |
| BCL2A1 | 0 | 0 | 0 | 0 | 0 | 0 | 1 |
| DPPA3 | 0 | 0 | 0 | 0 | 0 | 0 | 1 |
| SNCA | 0 | 0 | 0 | 0 | 0 | 0 | 1 |
| METTL18 | 0 | 0 | 0 | 0 | 0 | 0 | 1 |
| BPGM | 0 | 0 | 0 | 0 | 0 | 0 | 1 |
| FAM3B | 0 | 0 | 0 | 0 | 0 | 0 | 1 |
| SLC1A5 | 0 | 0 | 0 | 0 | 0 | 0 | 1 |
| RPL34 | 0 | 0 | 0 | 0 | 0 | 0 | 1 |
| KDM7A-DT | 0 | 0 | 0 | 0 | 0 | 0 | 1 |
| C9orf78 | 0 | 0 | 0 | 0 | 0 | 0 | 1 |
| HAUS1 | 0 | 0 | 0 | 0 | 0 | 0 | 1 |
| HEMGN | 0 | 0 | 0 | 0 | 0 | 0 | 1 |
| KANSL1-AS1 | 0 | 0 | 0 | 0 | 0 | 0 | 1 |
| CHMP5 | 0 | 0 | 0 | 0 | 0 | 0 | 1 |
| EPSTI1 | 0 | 0 | 0 | 0 | 0 | 0 | 1 |
| SLIRP | 0 | 0 | 0 | 0 | 0 | 0 | 1 |
| FCGR1B | 0 | 0 | 0 | 0 | 0 | 0 | 1 |
| RSAD2 | 0 | 0 | 0 | 0 | 0 | 0 | 1 |
| FRG1JP | 0 | 0 | 0 | 0 | 0 | 0 | 1 |
| CAMP | 0 | 0 | 0 | 0 | 0 | 0 | 1 |
| MRPL1 | 0 | 0 | 0 | 0 | 0 | 0 | 1 |
