# Supplementary material for: Potential association between COVID-19 and neurological disorders: analysis of common genes and therapeutics
Source: Front Neurol. 2024 Oct 14;15:1417183. doi: 10.3389/fneur.2024.1417183 (PMC11513677; doi:10.3389/fneur.2024.1417183)
Supplement: Supplementary file 4 [file Table_4.docx]

**Supplementary Table 4**

**Co-DEGs_1**

| COVID-SD | COVID-HS | COVID-AD | COVID-EP | COVID-PD |
| --- | --- | --- | --- | --- |
| ABHD2 | ABHD2 |  |  |  |
| CAPG |  |  | CAPG |  |
| CD19 |  |  |  | CD19 |
| CSRNP1 | CSRNP1 |  |  |  |
| FKBP8 | FKBP8 |  |  |  |
| FOS |  |  |  | FOS |
| FOXO4 |  | FOXO4 |  |  |
| IL1RAP | IL1RAP |  |  |  |
| KCNE3 | KCNE3 |  |  |  |
| LCN2 |  | LCN2 |  |  |
| LILRA3 | LILRA3 |  |  |  |
| MAPK14 | MAPK14 |  |  |  |
| SIGLEC10 | SIGLEC10 |  |  |  |
| SLC22A18 | SLC22A18 |  |  |  |
| SMIM5 |  | SMIM5 |  |  |
| TIMP1 | TIMP1 |  |  |  |
| TMEM45B | TMEM45B |  |  |  |
| TNFAIP6 | TNFAIP6 |  |  |  |
|  | FOSB |  |  | FOSB |

**Co-DEGs_2**

| Gene | Disease Group |
| --- | --- |
| ABHD2 | COVID-SD |
| CAPG | COVID-SD |
| CD19 | COVID-SD |
| CSRNP1 | COVID-SD |
| FKBP8 | COVID-SD |
| FOS | COVID-SD |
| FOXO4 | COVID-SD |
| IL1RAP | COVID-SD |
| KCNE3 | COVID-SD |
| LCN2 | COVID-SD |
| LILRA3 | COVID-SD |
| MAPK14 | COVID-SD |
| SIGLEC10 | COVID-SD |
| SLC22A18 | COVID-SD |
| SMIM5 | COVID-SD |
| TIMP1 | COVID-SD |
| TMEM45B | COVID-SD |
| TNFAIP6 | COVID-SD |
| ABHD2 | COVID-HS |
| CSRNP1 | COVID-HS |
| FKBP8 | COVID-HS |
| FOSB | COVID-HS |
| IL1RAP | COVID-HS |
| KCNE3 | COVID-HS |
| LILRA3 | COVID-HS |
| MAPK14 | COVID-HS |
| SIGLEC10 | COVID-HS |
| SLC22A18 | COVID-HS |
| TIMP1 | COVID-HS |
| TMEM45B | COVID-HS |
| TNFAIP6 | COVID-HS |
| FOXO4 | COVID-AD |
| LCN2 | COVID-AD |
| SMIM5 | COVID-AD |
| CAPG | COVID-EP |
| CD19 | COVID-PD |
| FOS | COVID-PD |
| FOSB | COVID-PD |
