# Supplementary material for: Potential association between COVID-19 and neurological disorders: analysis of common genes and therapeutics
Source: Front Neurol. 2024 Oct 14;15:1417183. doi: 10.3389/fneur.2024.1417183 (PMC11513677; doi:10.3389/fneur.2024.1417183)
Supplement: Supplementary file 5 [file Table_5.docx]

**Supplementary Table 5**

**Neuro_COVID**

| COVID_SD | COVID_HS | COVID_AD | COVID_EP | COVID_PD | COVID_IS |
| --- | --- | --- | --- | --- | --- |
| CEACAM1 | BHMT2 | BAAT | SMYD3-IT1 | FOSB | ARG1 |
| FABP5 | EMP2 | IGF1 | LAMA3 | FOS | ACSL1 |
| FOS | FOSB | AZGP1 | HNRNPCP6 | EGR1 | IQGAP1 |
| APOM | PPIL6 | VNN1 | MEG3 | ID3 |  |
| CAVIN2 | HEXIM1 | SERPINF2 | SUSD2 | KLF4 |  |
| BRF2 | SPRYD7 | ADORA2A-AS1 | HLA-DRA | TSPAN13 |  |
| MYADM | PBXIP1 | AMIGO2 | AKT3-IT1 | GNG7 |  |
| TMEM176A | INMT | PCSK6 | CAPG | DUSP1 |  |
| ASGR1 | SLC25A15 | MUC3A | CRYZ | BANK1 |  |
| CHI3L1 | ZNF440 | MDH1B | LAMA5 | STAB1 |  |
| BAG1 | PTPN14 | NGFR | AOC3 | CD19 |  |
| LRG1 | INSIG1 | FOXO4 | LIMS2 | MS4A1 |  |
| DESI1 | RPS16 | LCN2 | CASQ2 | CD79A |  |
| CPA3 | TTC39C | HIPK2 | ADIRF-AS1 |  |  |
| SAC3D1 | TMEM45B | PTPRQ |  |  |  |
| DGAT2 | RAB11FIP4 | KMO |  |  |  |
| TMEM45B | SOD2 | CPM |  |  |  |
| TMEM176B | WDR17 | HMGCS1 |  |  |  |
| PRDX5 | ITIH5 | FMO3 |  |  |  |
| CDA | PTMA | SLED1 |  |  |  |
| FOXO4 | S100A4 | OLAH |  |  |  |
| HMGN3 | MAPK14 | CLMN |  |  |  |
| LCN2 | SURF4 | IP6K3 |  |  |  |
| SLC43A1 | CD177 | IL1RL2 |  |  |  |
| HEY1 | SLC22A18 | ANKRD22 |  |  |  |
| DANCR | IBA57 | NAP1L5 |  |  |  |
| MAPK14 | DNAL1 | RASGRP3 |  |  |  |
| MT1F | TYW5 | DTHD1 |  |  |  |
| CAVIN3 | FAM174A | SFRP1 |  |  |  |
| TMEM140 | SMARCA5 | BEX5 |  |  |  |
| GLT1D1 | KIF3A | G0S2 |  |  |  |
| SNCA | MSMO1 | MAMDC4 |  |  |  |
| SLC22A18 | THBD | HIP1 |  |  |  |
| RPIA | PXMP4 | MIR2467 |  |  |  |
| LGALSL | FKBP8 | LLGL2 |  |  |  |
| TUBB4B | VPS37B | KRT19 |  |  |  |
| ALDOC | PCDHB9 | PCSK1 |  |  |  |
| RMDN3 | SPRED1 | MSC-AS1 |  |  |  |
| SHMT2 | PNPO | SMIM5 |  |  |  |
| PLEKHF1 | GPLD1 | GIPR |  |  |  |
| RANGRF | KDELR2 | SELENOP |  |  |  |
| VKORC1 | UBL5 | NRN1 |  |  |  |
| FRMD3 | PPIF | KCNJ15 |  |  |  |
| NINJ1 | SH3BP5 | TPTEP1 |  |  |  |
| TRAPPC1 | CEBPD | CR1 |  |  |  |
| FKBP8 | RAB3B | BAALC-AS1 |  |  |  |
| VAT1 | TM9SF1 | SH2D3A |  |  |  |
| SLPI | NLN | PKHD1L1 |  |  |  |
| PFDN4 | SCD5 | SPTBN5 |  |  |  |
| PELI3 | ACBD7 | ADCY10P1 |  |  |  |
| SNRPD1 | CSRNP1 | ZNF90 |  |  |  |
| CLU | TIMP1 | PRSS8 |  |  |  |
| NIPSNAP1 | SCAI | ABCA6 |  |  |  |
| GIMAP7 | SLC4A8 | RAB3C |  |  |  |
| TRIM58 | CYP4V2 | TFCP2L1 |  |  |  |
| LBH | HIF1A | SFTPA2 |  |  |  |
| B9D2 | TRIM65 | SELE |  |  |  |
| BANF1 | LDHA | USP2-AS1 |  |  |  |
| POLDIP2 | HCAR2 | MUC16 |  |  |  |
| SURF2 | HMGN2 | STAT4 |  |  |  |
| STRADB | SIGLEC10 | VGLL3 |  |  |  |
| ALAS1 | GDPD1 | CD22 |  |  |  |
| AIFM1 | ZNF714 |  |  |  |  |
| CSRNP1 | APLP2 |  |  |  |  |
| CRYL1 | ZNF713 |  |  |  |  |
| WLS | SLC20A1 |  |  |  |  |
| TIMP1 | TFDP2 |  |  |  |  |
| SH3GLB2 | SERTAD1 |  |  |  |  |
| AGAP9 | AK3 |  |  |  |  |
| C4BPA | CPD |  |  |  |  |
| SIGLEC10 | EEF2K |  |  |  |  |
| MT1X | AXL |  |  |  |  |
| HSP90AA1 | LRIF1 |  |  |  |  |
| PRKAR2B | ABHD2 |  |  |  |  |
| MIX23 | IL1RAP |  |  |  |  |
| NOP2 | ZNF483 |  |  |  |  |
| CHP1 | NXN |  |  |  |  |
| CHMP6 | TREM1 |  |  |  |  |
| TAGLN2 | FGL2 |  |  |  |  |
| TST | RGS2 |  |  |  |  |
| ACADVL | DMXL2 |  |  |  |  |
| PKIG | LPCAT1 |  |  |  |  |
| PCTP | NPC2 |  |  |  |  |
| ABHD2 | SLC26A8 |  |  |  |  |
| MPND | ROMO1 |  |  |  |  |
| IL1RAP | H6PD |  |  |  |  |
| ORAI3 | FXN |  |  |  |  |
| SLC27A3 | FLNA |  |  |  |  |
| TMOD1 | NCMAP |  |  |  |  |
| TMEM86B | GADD45B |  |  |  |  |
| BCKDK | EXPH5 |  |  |  |  |
| HLA-DQA1 | GAPDH |  |  |  |  |
| SMIM30 | QSOX1 |  |  |  |  |
| NELFE | TRIM59 |  |  |  |  |
| C6orf136 | PGK1 |  |  |  |  |
| USP30 | GNPNAT1 |  |  |  |  |
| INAFM1 | CHRNB1 |  |  |  |  |
| RTL6 | TPM4 |  |  |  |  |
| GALNT6 | PNMA2 |  |  |  |  |
| TFEB | IRAK3 |  |  |  |  |
| POR | DUSP19 |  |  |  |  |
| DHRS13 | PLEKHH2 |  |  |  |  |
| CAPG | KCNJ5 |  |  |  |  |
| EEF1AKMT3 | COX7B |  |  |  |  |
| PPP1R15A | SESN2 |  |  |  |  |
| EPHX2 | KCNE3 |  |  |  |  |
| DHFR2 | PRELP |  |  |  |  |
| ELAPOR1 | TNFAIP6 |  |  |  |  |
| PCK2 | IFIT2 |  |  |  |  |
| GUCD1 | LILRA3 |  |  |  |  |
| GCDH |  |  |  |  |  |
| CR1L |  |  |  |  |  |
| MXI1 |  |  |  |  |  |
| SMIM5 |  |  |  |  |  |
| DCXR |  |  |  |  |  |
| TGM2 |  |  |  |  |  |
| HOMER1 |  |  |  |  |  |
| PRDX6 |  |  |  |  |  |
| ABCG1 |  |  |  |  |  |
| LSP1 |  |  |  |  |  |
| NRGN |  |  |  |  |  |
| YBX3 |  |  |  |  |  |
| CDCA4 |  |  |  |  |  |
| ARHGAP6 |  |  |  |  |  |
| ROPN1L |  |  |  |  |  |
| RASEF |  |  |  |  |  |
| RAMP2-AS1 |  |  |  |  |  |
| CFP |  |  |  |  |  |
| MST1L |  |  |  |  |  |
| PLA2G15 |  |  |  |  |  |
| ZNF213 |  |  |  |  |  |
| LTBP2 |  |  |  |  |  |
| PNPLA6 |  |  |  |  |  |
| HSPE1 |  |  |  |  |  |
| CHPT1 |  |  |  |  |  |
| TUBB6 |  |  |  |  |  |
| HSPB1 |  |  |  |  |  |
| PCED1B |  |  |  |  |  |
| PLEK2 |  |  |  |  |  |
| IFI35 |  |  |  |  |  |
| ANKRD55 |  |  |  |  |  |
| MLLT11 |  |  |  |  |  |
| CAPN12 |  |  |  |  |  |
| TSPAN2 |  |  |  |  |  |
| PGRMC1 |  |  |  |  |  |
| GNAZ |  |  |  |  |  |
| KCNE3 |  |  |  |  |  |
| LILRA1 |  |  |  |  |  |
| TSPAN33 |  |  |  |  |  |
| TNFAIP6 |  |  |  |  |  |
| PPM1M |  |  |  |  |  |
| HLA-DQB1 |  |  |  |  |  |
| CD19 |  |  |  |  |  |
| CCR1 |  |  |  |  |  |
| MX1 |  |  |  |  |  |
| LILRA3 |  |  |  |  |  |

**DEGs unions**

| DEGs_union | logFC | Disease |
| --- | --- | --- |
| NRN1 | 1.277835519 | Cov-AD |
| PCSK1 | 1.910706808 | Cov-AD |
| MDH1B | 1.149415212 | Cov-AD |
| FOXO4 | -1.271735756 | Cov-AD |
| USP2-AS1 | 1.477922923 | Cov-AD |
| BEX5 | 1.32815609 | Cov-AD |
| CPM | -1.111543826 | Cov-AD |
| STAT4 | 1.473842465 | Cov-AD |
| IL1RL2 | 1.215908974 | Cov-AD |
| NAP1L5 | 1.060849699 | Cov-AD |
| MSC-AS1 | 1.250837283 | Cov-AD |
| ADCY10P1 | -1.014370235 | Cov-AD |
| HIP1 | -1.075069202 | Cov-AD |
| DTHD1 | -1.508680683 | Cov-AD |
| HMGCS1 | 1.044773659 | Cov-AD |
| SERPINF2 | -1.284460365 | Cov-AD |
| LLGL2 | -1.003055055 | Cov-AD |
| KMO | 1.1683805 | Cov-AD |
| BAALC-AS1 | 1.302449277 | Cov-AD |
| IGF1 | 1.09955993 | Cov-AD |
| PRSS8 | -1.099096101 | Cov-AD |
| MAMDC4 | -1.208769727 | Cov-AD |
| OLAH | -1.486919538 | Cov-AD |
| ABCA6 | -1.163989915 | Cov-AD |
| AMIGO2 | 1.007422023 | Cov-AD |
| SMIM5 | -1.196507977 | Cov-AD |
| SELE | 2.350541733 | Cov-AD |
| NGFR | -1.543036102 | Cov-AD |
| CLMN | -1.114101012 | Cov-AD |
| SH2D3A | -1.416826074 | Cov-AD |
| MUC3A | 1.268324828 | Cov-AD |
| MIR2467 | -1.07323979 | Cov-AD |
| FMO3 | -1.053544878 | Cov-AD |
| VGLL3 | -1.027857269 | Cov-AD |
| ZNF90 | -1.027823574 | Cov-AD |
| HIPK2 | -1.101720836 | Cov-AD |
| KRT19 | -1.240473231 | Cov-AD |
| TFCP2L1 | -1.108412504 | Cov-AD |
| ANKRD22 | 2.031370349 | Cov-AD |
| TPTEP1 | -1.1078093 | Cov-AD |
| MUC16 | 1.857594206 | Cov-AD |
| AZGP1 | -1.211526638 | Cov-AD |
| SLED1 | -1.010865827 | Cov-AD |
| SELENOP | -1.071987793 | Cov-AD |
| SFRP1 | -1.247561787 | Cov-AD |
| G0S2 | -1.125267969 | Cov-AD |
| ADORA2A-AS1 | -1.18375853 | Cov-AD |
| IP6K3 | -1.607075868 | Cov-AD |
| LCN2 | 2.571978443 | Cov-AD |
| SPTBN5 | -1.05255696 | Cov-AD |
| VNN1 | -1.888598297 | Cov-AD |
| PKHD1L1 | -1.641416657 | Cov-AD |
| CR1 | -1.081485745 | Cov-AD |
| PTPRQ | 1.035884176 | Cov-AD |
| RAB3C | 1.001344497 | Cov-AD |
| SFTPA2 | 1.136076839 | Cov-AD |
| CD22 | -1.25109533 | Cov-AD |
| PCSK6 | -1.169181193 | Cov-AD |
| KCNJ15 | -1.234541067 | Cov-AD |
| BAAT | 1.104642193 | Cov-AD |
| RASGRP3 | -1.056234743 | Cov-AD |
| GIPR | -1.165318727 | Cov-AD |
| SUSD2 | -1.888866551 | Cov-EP |
| LAMA5 | -1.179878767 | Cov-EP |
| LIMS2 | -1.195497703 | Cov-EP |
| AKT3-IT1 | -1.943344322 | Cov-EP |
| CASQ2 | -1.677966452 | Cov-EP |
| CAPG | 1.282049854 | Cov-EP |
| HNRNPCP6 | -1.903450893 | Cov-EP |
| CRYZ | 1.119413993 | Cov-EP |
| AOC3 | -2.142040435 | Cov-EP |
| ADIRF-AS1 | -1.786016874 | Cov-EP |
| SMYD3-IT1 | -2.045274934 | Cov-EP |
| MEG3 | -1.538810231 | Cov-EP |
| LAMA3 | -1.009472321 | Cov-EP |
| HLA-DRA | 1.303145752 | Cov-EP |
| INMT | -1.920583992 | Cov-HS |
| RAB3B | -2.204701686 | Cov-HS |
| EEF2K | -1.498264259 | Cov-HS |
| FXN | -1.425383483 | Cov-HS |
| ACBD7 | -1.563976139 | Cov-HS |
| ZNF483 | -1.456663678 | Cov-HS |
| PRELP | -1.789813961 | Cov-HS |
| PPIL6 | -1.738142413 | Cov-HS |
| CYP4V2 | -1.165049132 | Cov-HS |
| NXN | -2.228938181 | Cov-HS |
| SPRED1 | -1.826234443 | Cov-HS |
| PTPN14 | -1.392186318 | Cov-HS |
| ZNF713 | -1.352450904 | Cov-HS |
| TFDP2 | -1.066456826 | Cov-HS |
| NCMAP | -1.218892101 | Cov-HS |
| PXMP4 | -1.05243311 | Cov-HS |
| PNPO | -1.256885542 | Cov-HS |
| SCD5 | -1.099571695 | Cov-HS |
| PNMA2 | -1.689225109 | Cov-HS |
| EMP2 | -1.091433494 | Cov-HS |
| SCAI | -1.174207268 | Cov-HS |
| EXPH5 | -1.163880503 | Cov-HS |
| DNAL1 | -1.009158414 | Cov-HS |
| TRIM65 | -1.349275039 | Cov-HS |
| CHRNB1 | -1.305448004 | Cov-HS |
| IBA57 | -1.137334186 | Cov-HS |
| GDPD1 | -1.015931323 | Cov-HS |
| KCNJ5 | -1.675169761 | Cov-HS |
| PLEKHH2 | -1.894099419 | Cov-HS |
| AK3 | -1.461146798 | Cov-HS |
| UBL5 | -1.024232877 | Cov-HS |
| SLC4A8 | -1.313959817 | Cov-HS |
| ZNF714 | -1.295910547 | Cov-HS |
| WDR17 | -1.158579977 | Cov-HS |
| TYW5 | -2.501355829 | Cov-HS |
| PCDHB9 | -1.113684858 | Cov-HS |
| FKBP8 | 1.381652629 | Cov-HS |
| HEXIM1 | 1.831873465 | Cov-HS |
| DUSP19 | -1.234604299 | Cov-HS |
| BHMT2 | -1.12802344 | Cov-HS |
| GNPNAT1 | -1.376144707 | Cov-HS |
| THBD | 3.028614875 | Cov-HS |
| SLC25A15 | -1.252189559 | Cov-HS |
| LRIF1 | 1.910395228 | Cov-HS |
| FLNA | 3.195771639 | Cov-HS |
| TNFAIP6 | 1.471054104 | Cov-HS |
| COX7B | -1.036149308 | Cov-HS |
| GPLD1 | -1.213339997 | Cov-HS |
| CD177 | -2.541808996 | Cov-HS |
| TRIM59 | -1.281296905 | Cov-HS |
| ABHD2 | 1.017476403 | Cov-HS |
| ITIH5 | -1.275649434 | Cov-HS |
| TPM4 | 2.57154279 | Cov-HS |
| IL1RAP | 1.944630641 | Cov-HS |
| HCAR2 | 1.635657319 | Cov-HS |
| TTC39C | -1.010416917 | Cov-HS |
| CEBPD | -1.440061294 | Cov-HS |
| KIF3A | -1.292178826 | Cov-HS |
| MAPK14 | -1.537426115 | Cov-HS |
| AXL | -1.076715157 | Cov-HS |
| FGL2 | 2.377185269 | Cov-HS |
| SURF4 | 1.563097703 | Cov-HS |
| ROMO1 | -1.266599281 | Cov-HS |
| PTMA | 1.942931296 | Cov-HS |
| RAB11FIP4 | 1.272578981 | Cov-HS |
| SOD2 | 1.084670302 | Cov-HS |
| TIMP1 | 2.277138947 | Cov-HS |
| SLC22A18 | 1.413804463 | Cov-HS |
| LPCAT1 | 2.975754016 | Cov-HS |
| NPC2 | 1.274538039 | Cov-HS |
| INSIG1 | 2.452486483 | Cov-HS |
| SPRYD7 | -1.086689988 | Cov-HS |
| SMARCA5 | 1.313690824 | Cov-HS |
| FOSB | 1.50531554 | Cov-HS |
| TM9SF1 | 1.298401536 | Cov-HS |
| HMGN2 | 1.016771273 | Cov-HS |
| CSRNP1 | 1.259102475 | Cov-HS |
| NLN | -1.573935308 | Cov-HS |
| TREM1 | 1.042798751 | Cov-HS |
| PPIF | 1.80101845 | Cov-HS |
| APLP2 | 1.63735637 | Cov-HS |
| PGK1 | 1.344246935 | Cov-HS |
| VPS37B | 1.099909637 | Cov-HS |
| H6PD | -1.483969357 | Cov-HS |
| LDHA | 2.172311402 | Cov-HS |
| TMEM45B | 1.51186118 | Cov-HS |
| PBXIP1 | 1.388005682 | Cov-HS |
| KDELR2 | 1.224724944 | Cov-HS |
| SH3BP5 | 1.414223609 | Cov-HS |
| GAPDH | 1.625518389 | Cov-HS |
| RPS16 | 1.168156477 | Cov-HS |
| SLC26A8 | -1.518586678 | Cov-HS |
| LILRA3 | 1.344142826 | Cov-HS |
| IFIT2 | 1.368907137 | Cov-HS |
| DMXL2 | 1.117977414 | Cov-HS |
| ZNF440 | -1.312502919 | Cov-HS |
| SERTAD1 | 1.220287692 | Cov-HS |
| MSMO1 | -1.31827627 | Cov-HS |
| CPD | 1.363988095 | Cov-HS |
| IRAK3 | -1.103432855 | Cov-HS |
| SIGLEC10 | 1.117310891 | Cov-HS |
| HIF1A | 1.140208905 | Cov-HS |
| FAM174A | 1.035034819 | Cov-HS |
| SESN2 | 1.64971839 | Cov-HS |
| S100A4 | -1.129838013 | Cov-HS |
| RGS2 | 1.09079962 | Cov-HS |
| QSOX1 | 1.065065019 | Cov-HS |
| KCNE3 | -1.047015768 | Cov-HS |
| SLC20A1 | 1.169343837 | Cov-HS |
| GADD45B | 1.432424556 | Cov-HS |
| ARG1 | 1.585532372 | Cov-IS |
| IQGAP1 | 1.007538257 | Cov-IS |
| ACSL1 | 1.004878988 | Cov-IS |
| FOS | -2.42616234 | Cov-PD |
| FOSB | -3.187027693 | Cov-PD |
| EGR1 | -2.202921582 | Cov-PD |
| MS4A1 | 1.513432992 | Cov-PD |
| KLF4 | -1.033070325 | Cov-PD |
| STAB1 | -1.121769974 | Cov-PD |
| BANK1 | 1.358733227 | Cov-PD |
| TSPAN13 | 1.211528289 | Cov-PD |
| CD79A | 1.508810452 | Cov-PD |
| ID3 | 1.008736706 | Cov-PD |
| GNG7 | 1.074285755 | Cov-PD |
| DUSP1 | -1.1394244 | Cov-PD |
| CD19 | 1.333873082 | Cov-PD |
| TMEM176A | 3.36084828 | Cov-SD |
| TMEM176B | 2.180979836 | Cov-SD |
| CHP1 | 1.380782098 | Cov-SD |
| APOM | 1.141071417 | Cov-SD |
| HLA-DQA1 | 4.102475965 | Cov-SD |
| HLA-DQB1 | 2.349899831 | Cov-SD |
| EEF1AKMT3 | 1.957695785 | Cov-SD |
| ELAPOR1 | 1.682884304 | Cov-SD |
| C4BPA | 4.135892144 | Cov-SD |
| PKIG | 1.55754086 | Cov-SD |
| IL1RAP | 1.330555563 | Cov-SD |
| DHFR2 | -1.327675331 | Cov-SD |
| MIX23 | -2.561063021 | Cov-SD |
| RAMP2-AS1 | -1.378110267 | Cov-SD |
| ACADVL | 1.059127413 | Cov-SD |
| NIPSNAP1 | 1.106531892 | Cov-SD |
| BRF2 | 1.009089652 | Cov-SD |
| HOMER1 | -1.035837528 | Cov-SD |
| CAVIN3 | 1.302204249 | Cov-SD |
| RANGRF | 1.078126784 | Cov-SD |
| POLDIP2 | 1.374760638 | Cov-SD |
| DESI1 | 1.497457909 | Cov-SD |
| ALDOC | 2.198108073 | Cov-SD |
| ORAI3 | 1.205473784 | Cov-SD |
| CAPG | 2.034529578 | Cov-SD |
| LILRA3 | -2.256185461 | Cov-SD |
| USP30 | 1.067006603 | Cov-SD |
| RMDN3 | 1.121905894 | Cov-SD |
| TAGLN2 | 1.869309047 | Cov-SD |
| PRDX6 | 1.033735363 | Cov-SD |
| RASEF | -1.646836779 | Cov-SD |
| TSPAN33 | 1.82474904 | Cov-SD |
| TUBB4B | 1.709434531 | Cov-SD |
| PELI3 | 1.318002347 | Cov-SD |
| TIMP1 | 1.356531967 | Cov-SD |
| HEY1 | -2.372299518 | Cov-SD |
| MLLT11 | 1.02580915 | Cov-SD |
| GNAZ | 1.226532815 | Cov-SD |
| VAT1 | 1.178224076 | Cov-SD |
| ZNF213 | 1.709045748 | Cov-SD |
| SH3GLB2 | 1.201562294 | Cov-SD |
| HSP90AA1 | -1.807360933 | Cov-SD |
| CHMP6 | 1.254924991 | Cov-SD |
| LGALSL | 1.093862252 | Cov-SD |
| PPM1M | 1.06104272 | Cov-SD |
| TRAPPC1 | 1.752107579 | Cov-SD |
| CAPN12 | 1.070393181 | Cov-SD |
| MAPK14 | 1.362137811 | Cov-SD |
| PPP1R15A | 1.860071277 | Cov-SD |
| VKORC1 | 1.284142472 | Cov-SD |
| ARHGAP6 | 1.174574309 | Cov-SD |
| NRGN | 2.638243243 | Cov-SD |
| RTL6 | 1.150967584 | Cov-SD |
| TSPAN2 | 1.076352451 | Cov-SD |
| TST | 1.756962462 | Cov-SD |
| GCDH | 1.036015127 | Cov-SD |
| NELFE | 1.028896009 | Cov-SD |
| CPA3 | 3.995468205 | Cov-SD |
| B9D2 | 1.034584803 | Cov-SD |
| MYADM | 1.313668076 | Cov-SD |
| CD19 | 1.188661756 | Cov-SD |
| PLA2G15 | 1.060141947 | Cov-SD |
| SURF2 | 1.198481194 | Cov-SD |
| FKBP8 | 1.878081882 | Cov-SD |
| TMEM140 | 2.02920891 | Cov-SD |
| NINJ1 | 1.355191914 | Cov-SD |
| SHMT2 | 1.064235311 | Cov-SD |
| AGAP9 | -1.332511555 | Cov-SD |
| FOS | 1.761457474 | Cov-SD |
| BCKDK | 1.30015319 | Cov-SD |
| MXI1 | 1.817780828 | Cov-SD |
| AIFM1 | 1.026475567 | Cov-SD |
| SMIM5 | 2.853428651 | Cov-SD |
| TFEB | 1.008297039 | Cov-SD |
| BAG1 | 1.919353489 | Cov-SD |
| MPND | 1.473562704 | Cov-SD |
| HSPB1 | 1.788451134 | Cov-SD |
| BANF1 | 1.175123175 | Cov-SD |
| LTBP2 | 1.430314404 | Cov-SD |
| TMEM86B | 2.42122533 | Cov-SD |
| GIMAP7 | -1.571725843 | Cov-SD |
| C6orf136 | 1.188176025 | Cov-SD |
| CDCA4 | 1.302409593 | Cov-SD |
| PGRMC1 | 1.173168649 | Cov-SD |
| PCK2 | 1.102384021 | Cov-SD |
| ALAS1 | 1.513823332 | Cov-SD |
| TUBB6 | 1.290119092 | Cov-SD |
| ABCG1 | 1.054650442 | Cov-SD |
| ASGR1 | 1.100294157 | Cov-SD |
| SAC3D1 | 1.028610333 | Cov-SD |
| CHPT1 | 1.062416772 | Cov-SD |
| FABP5 | -1.099953011 | Cov-SD |
| LILRA1 | 1.116139225 | Cov-SD |
| TGM2 | 1.5147841 | Cov-SD |
| GALNT6 | 1.10747026 | Cov-SD |
| TMEM45B | 1.443109746 | Cov-SD |
| CDA | 1.662885473 | Cov-SD |
| LSP1 | 1.108588426 | Cov-SD |
| GUCD1 | 2.259174809 | Cov-SD |
| INAFM1 | 1.633705268 | Cov-SD |
| STRADB | 2.305107608 | Cov-SD |
| DCXR | 1.387967242 | Cov-SD |
| CR1L | 2.893295046 | Cov-SD |
| CRYL1 | 1.057941862 | Cov-SD |
| MST1L | -1.354252479 | Cov-SD |
| MT1X | 1.174533914 | Cov-SD |
| PRKAR2B | 1.535731541 | Cov-SD |
| ROPN1L | 1.167228962 | Cov-SD |
| SIGLEC10 | 1.138349302 | Cov-SD |
| CLU | 1.204510452 | Cov-SD |
| KCNE3 | 1.10863047 | Cov-SD |
| SLC43A1 | 1.002611713 | Cov-SD |
| CHI3L1 | 1.033457366 | Cov-SD |
| LBH | 1.002284098 | Cov-SD |
| FRMD3 | 1.24209141 | Cov-SD |
| TMOD1 | 3.094187714 | Cov-SD |
| ABHD2 | 1.167848421 | Cov-SD |
| MT1F | 1.134956847 | Cov-SD |
| TRIM58 | 3.141983669 | Cov-SD |
| PNPLA6 | 1.244418245 | Cov-SD |
| POR | 1.360489204 | Cov-SD |
| CAVIN2 | 1.555997153 | Cov-SD |
| PRDX5 | 1.040720535 | Cov-SD |
| NOP2 | 1.037567466 | Cov-SD |
| CCR1 | 1.387178943 | Cov-SD |
| DANCR | 1.066201899 | Cov-SD |
| PLEKHF1 | 1.021113203 | Cov-SD |
| ANKRD55 | 1.451017754 | Cov-SD |
| GLT1D1 | 1.085836989 | Cov-SD |
| SLC27A3 | 1.175819871 | Cov-SD |
| CSRNP1 | 1.198182402 | Cov-SD |
| CFP | 1.029743835 | Cov-SD |
| DHRS13 | 1.112237948 | Cov-SD |
| PFDN4 | -1.170839191 | Cov-SD |
| HMGN3 | -1.046221233 | Cov-SD |
| HSPE1 | -1.06430045 | Cov-SD |
| YBX3 | 1.060468427 | Cov-SD |
| DGAT2 | 1.17357628 | Cov-SD |
| LRG1 | 1.53713238 | Cov-SD |
| PCTP | 1.220277443 | Cov-SD |
| SMIM30 | -1.088353497 | Cov-SD |
| FOXO4 | 1.336051094 | Cov-SD |
| SLPI | 1.397990653 | Cov-SD |
| WLS | 1.375544176 | Cov-SD |
| PCED1B | 1.086881732 | Cov-SD |
| PLEK2 | 2.539685512 | Cov-SD |
| TNFAIP6 | 1.601860493 | Cov-SD |
| SNRPD1 | -1.185683945 | Cov-SD |
| CEACAM1 | 1.073051972 | Cov-SD |
| LCN2 | 2.407306812 | Cov-SD |
| SLC22A18 | 1.036181404 | Cov-SD |
| MX1 | 1.307521326 | Cov-SD |
| EPHX2 | 1.06249137 | Cov-SD |
| RPIA | 1.017431855 | Cov-SD |
| SNCA | 1.49055675 | Cov-SD |
| IFI35 | 1.282114523 | Cov-SD |
